# Hyperglycemia‐induced Sirt3 downregulation increases microglial aerobic glycolysis and inflammation in diabetic neuropathic pain pathogenesis

**DOI:** 10.1111/cns.14913

**Published:** 2024-08-09

**Authors:** Yongchang Li, Erliang Kong, Ruifeng Ding, Ruitong Chu, Jinfang Lu, Mengqiu Deng, Tong Hua, Mei Yang, Haowei Wang, Dashuang Chen, Honghao Song, Huawei Wei, Ping Zhang, Chaofeng Han, Hongbin Yuan

**Affiliations:** ^1^ Department of Anesthesiology, Shanghai Changzheng Hospital Second Affiliated Hospital of Naval Medical University Shanghai China; ^2^ Department of Anesthesiology The 988th Hospital of Joint Logistic Support Force of Chinese People's Liberation Army Zhengzhou Henan China; ^3^ Department of Neurology, Naval Medical Center of PLA Naval Medical University Shanghai China; ^4^ Department of Histology and Embryology, Shanghai Key Laboratory of Cell Engineering Naval Medical University Shanghai China; ^5^ National Key Laboratory of Immunity & Inflammation Naval Medical University Shanghai China

**Keywords:** Akt/FoxO1, diabetic neuropathic pain, glycolysis, metformin, neuroinflammation, Sirt3

## Abstract

**Background:**

Hyperglycemia‐induced neuroinflammation significantly contributes to diabetic neuropathic pain (DNP), but the underlying mechanisms remain unclear.

**Objective:**

To investigate the role of Sirt3, a mitochondrial deacetylase, in hyperglycemia‐induced neuroinflammation and DNP and to explore potential therapeutic interventions.

**Method and Results:**

Here, we found that Sirt3 was downregulated in spinal dorsal horn (SDH) of diabetic mice by RNA‐sequencing, which was further confirmed at the mRNA and protein level. Sirt3 deficiency exacerbated hyperglycemia‐induced neuroinflammation and DNP by enhancing microglial aerobic glycolysis in vivo and in vitro. Overexpression of Sirt3 in microglia alleviated inflammation by reducing aerobic glycolysis. Mechanistically, high‐glucose stimulation activated Akt, which phosphorylates and inactivates FoxO1. The inactivation of FoxO1 diminished the transcription of Sirt3. Besides that, we also found that hyperglycemia induced Sirt3 degradation via the mitophagy‐lysosomal pathway. Blocking Akt activation by GSK69093 or metformin rescued the degradation of Sirt3 protein and transcription inhibition of Sirt3 mRNA, which substantially diminished hyperglycemia‐induced inflammation. Metformin in vivo treatment alleviated neuroinflammation and diabetic neuropathic pain by rescuing hyperglycemia‐induced Sirt3 downregulation.

**Conclusion:**

Hyperglycemia induces metabolic reprogramming and inflammatory activation in microglia through the regulation of Sirt3 transcription and degradation. This novel mechanism identifies Sirt3 as a potential drug target for treating DNP.

## Graphical abstract

Li et al. have addressed the critical role of hyperglycemia‐induced neuroinflammation in the pathogenesis of diabetic neuropathic pain (DNP), a significant and growing global health concern due to the rising prevalence of diabetes. Metformin can decrease neuroinflammation and DNP, and this effect depends on its regulation of Sirt3 protein level.

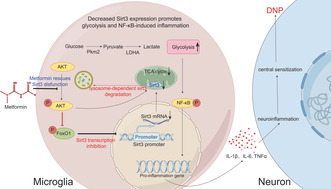

## 1 INTRODUCTION

As of 2021, it is estimated that there are 537 million individuals afflicted with diabetes worldwide, projections indicate that this number is anticipated to escalate to 643 million by the year 2030, and further surge to 783 million by 2045.^1^ Diabetic neuropathic pain (DNP) stands as a prevalent complication of diabetes mellitus, marked by aberrations in pain perception, manifested through spontaneous pain, allodynia, and hyperalgesia. Typically, approximately half of diabetes patients develop neurological complications, with about 30%–40% of these cases experiencing pain symptoms.^2^ The etiology of DNP is multifaceted, encompassing metabolic reprogramming, toxin‐induced effects, disturbances in nutritional provisioning, inflammatory processes, as well as genetic predispositions, among other factors.^3^ In terms of sexual dimorphism, sex‐specific decreases in neuroactive steroid levels in mice may lead to alterations in mitochondrial function, which in turn could impact axonal transport. This mechanism may contribute to the observed sex differences in DNP.^4^ Additionally, studies have shown that ovariectomy, but not orchidectomy, can alleviate DNP in mice by counteracting alterations in nerve conduction velocity (NCV), Na^+^, K^+^‐ATPase activity, and myelin protein expression.^5^ However, it is worth noting that approximately 40% of individuals suffering from DNP do not have a definite underlying cause.^2^

Hyperglycemia‐induced metabolic reprogramming stands as a pivotal driver in the onset of various diabetic complications, including DNP.^6^ Microglia, as resident immune cells in the brain and spinal cord, play a vital role in immune surveillance. However, the overactivation of microglia is generally considered to be neurotoxic.^7^ Much like macrophages in the peripheral nervous system (PNS),^8^ activated microglia in the context of inflammation persist in utilizing glucose through aerobic glycolysis for energy production, even under conditions of ample oxygen availability.^9^ While glycolysis yields ATP less efficiently compared to oxidative phosphorylation (OXPHOS), it provides a rapid source of energy to meet the demands of microglial proliferation, polarization, chemotaxis, and other activities during inflammatory responses.^10^, ^11^ During the pathological course of overactive inflammation in the central nervous system, a spectrum of enzyme and metabolic product alterations provides robust evidence for the existence of glucose metabolism reprogramming. HIF‐1α, also known as hypoxia‐inducible factor‐1 alpha, serves as a pivotal hallmark of excessive inflammation activation in the organism.^12^ In its capacity as a transcription factor, HIF‐1α exerts precise regulatory control over a spectrum of pivotal glycolytic enzymes, such as Glut1, LDHA, and various others.^13^ Metabolic reprogramming arises not only from the augmented glycolytic pathway but also significantly hinges on the pivotal suppression of the OXPHOS pathway. In pro‐inflammatory activated microglial cells, there is increased mitochondrial fission, accompanied by reduced activities of OXPHOS‐related enzymes such as isocitrate dehydrogenase.^14^, ^15^ This leads to the hindrance in the tricarboxylic acid (TCA) cycle, resulting in the accumulation of intermediate products like succinate. Succinate plays dual roles in inflammation. It drives extensive ROS generation through reverse electron transport at mitochondrial complex I. Additionally, it functions as an inflammatory signal, stabilizing HIF‐1α and subsequently upregulating key enzymes associated with glycolysis.^16^, ^17^ The reciprocal correlation and mechanism between suppressed OXPHOS and activated glycolysis in inflammatory states needs to be further explored.

Sirtuin 3 of the sirtuins family is a major mitochondrial NAD^+^‐dependent deacetylase with a broad range of functions, such as regulation of oxidative stress, reprogramming of tumor cell energy pathways, and metabolic homeostasis.^18^ Sirt3, among the three Sirtuins family proteins localized in the mitochondria, possesses the most robust deacetylase activity. It regulates over a hundred downstream proteins within the mitochondria, with a majority of them being activated through its deacetylation function.^19^ Sirt3 could enhance the TCA cycle and electron transport chain functions while reducing oxidative stress.^20^, ^21^ The absence of Sirt3 could initiate oxidative damage, reactive oxygen species (ROS)‐mediated signaling, and metabolic reprogramming to enhance cellular proliferation.^22^ Recent studies have indicated that Sirt3, through the reduction of ROS production, which is known to stabilize HIF1α, could facilitate the degradation of HIF1α.^23^ Consequently, this process leads to decreased cellular glycolysis levels. Given the close relationship between aerobic glycolysis and inflammation, many studies have confirmed that Sirt3 could control excessive inflammatory responses by regulating cellular metabolism.^24^, ^25^ Besides this, sirt3 could also attenuate inflammation through many non‐metabolism‐dependent mechanisms, such as directly influence the activation of inflammasomes or the NF‐κB pathway.^26^, ^27^ Despite the abundant research on the anti‐inflammatory effects of Sirt3 by deacetylating mitochondrial or non‐mitochondrial proteins, such as IDH and SOD,^28^, ^29^ its specific role in central nervous system inflammation induced by hyperglycemia remains unclear.

In this study, we demonstrated that the decreased expression of Sirt3 was related to increased glycolysis and neuroinflammation. The deficiency of Sirt3 aggravated neuroinflammation and DNP aerobic glycolysis. Akt/FoxO1 signaling to downregulate the transcription of Sirt3 and lysosomal degradation of Sirt3 upon high glucose stimulation were important mechanisms for the neuroinflammation of DNP. This pivotal signaling cascade plays a critical role in initiating microglial glycolytic reprogramming, marking a crucial step in the pathogenesis of DNP. Interventions targeting the Akt/FoxO1‐Sirt3 axis hold promise in alleviating neuropathic pain associated with diabetes.

## 2 MATERIALS AND METHODS

### 2.1 Mice

Male C57BL/6J wild‐type mice, aged 6–8 weeks and weighing between 20 and 25 g, were procured from the Experimental Animal Center at Naval Medical University (Shanghai, China). The 129‐Sirt3tm1.1Fwa/J (Sirt3^−/−^) mice (Stock number: 012755) were purchased from The Jackson Laboratory, USA. All experimental animals were housed in a specific pathogen‐free (SPF) environment at a temperature of 24°C, with a humidity level of 50%, under a 12‐h light–dark cycle. Animal experiments were conducted in accordance with the Guidelines for the Care and Use of Laboratory Animals of the National Institutes of Health and were approved by the Scientific Research Committee of Naval Medical University.

### 2.2 DNP model establishment

In this study, we induced a type 1 diabetes model through intraperitoneal injection of streptozotocin (STZ). Mice were subjected to a 12‐h fast, followed by a one‐time intraperitoneal injection of STZ at a dosage of 150 mg/kg. Preparation of STZ Solution: 2.1 g of citric acid (Molecular Weight: 210.14) was weighed and dissolved in 100 mL of double‐distilled water to form Solution A. 2.94 g of sodium citrate (Molecular Weight: 294.10) was weighed and dissolved in 100 mL of double‐distilled water to form Solution B. Solutions A and B were mixed in a 1:1 ratio. The pH was measured and adjusted to pH = 4.2–4.5. The solution was then sterilized by passing it through a 0.22 μm filter. This resulted in the preparation of a buffered solution. A 5% STZ solution was prepared using the citric acid/sodium citrate buffer. The solution was placed on ice and administered via intraperitoneal injection into mice within 10 min.

The littermates of 8‐week‐old male Sirt3^+/+^ (*n* = 20) and Sirt3^−/−^ (*n* = 20) mice were randomly involved in DNP group (*n* = 10) or Control group (*n* = 10). To investigate effect of metformin/2‐DG, 8‐week‐old C57BL/6J males were randomly divided into four groups: Control+ vehicle group (*n* = 10), Control+ metformin/2‐DG group (*n* = 10), DNP+ vehicle group (*n* = 10), DNP+ metformin/2‐DG group (*n* = 10).

### 2.3 Reagents

The following reagents were used in the experiments: STZ (Selleck, s1312, 150 mg/kg) was administered via intraperitoneal injection to induce DNP model. The glycolytic inhibitor 2‐Deoxy‐D‐glucose (2‐DG, Selleck, s4701, 250 mg/kg) was administered via intraperitoneal injection for 21 consecutive days after STZ injection. The Akt inhibitor (GSK690693, Selleck, s1113), FoxO1 inhibitor (AS1842856, Selleck, s8222), Metformin (Selleck, s5958), chloroquine (CQ) (Selleck, s6999), and MG132 (Selleck, s2619) were administered as cell culture reagents. Metformin (Selleck, s5958, 200 mg/kg) was administered via intraperitoneal injection for 21 consecutive days after STZ injection. The flowing antibodies were used: anti‐Sirt3 antibody (1:1000, ABclonal, A5718), anti‐Iba‐1 antibody (1:200, Cell Signaling Technology, #17198), anti‐GFAP antibody (1:200, Cell Signaling Technology, #80788), anti‐NeuN antibody (1:200, Cell Signaling Technology, #24307), anti‐NF‐κB p65 antibody (1:1000, Cell Signaling Technology, #8242), anti‐Phospho‐NF‐κB p65 antibody (1:1000, Cell Signaling Technology, #3033), anti‐p38 MAPK antibody (1:1000, Cell Signaling Technology, #8690), anti‐ Phospho‐p38 MAPK antibody (1:1000, Cell Signaling Technology, #4511), anti‐SAPK/JNK antibody (1:1000, Cell Signaling Technology, #9252), anti‐ Phospho‐SAPK/JNK antibody (1:1000, Cell Signaling Technology, #9255), anti‐p44/42 MAPK(Erk1/2) antibody (1:1000, Cell Signaling Technology, #4695), anti‐Phospho‐p44/42 MAPK(Erk1/2) antibody (1:1000, Cell Signaling Technology, #4370), anti‐Hexokinase II antibody (1:1000, Cell Signaling Technology, #2867), anti‐PKM2 antibody (1:1000, Cell Signaling Technology, #4053), anti‐LDHA antibody (1:1000, Cell Signaling Technology, #2012), anti‐Akt(pan) antibody (1:1000, Cell Signaling Technology, #4685), anti‐Phospho‐Akt antibody (1:1000, Cell Signaling Technology, #4060), anti‐FoxO1 antibody (1:1000, Cell Signaling Technology, #2880), and anti‐Phospho‐FoxO1 antibody (1:1000, Cell Signaling Technology, #9461).

### 2.4 Behavioral tests

Tactile allodynia (von Frey Test): Tactile allodynia was evaluated utilizing von Frey filaments (North Coast Medical, Gilroy, CA, USA) of 0.07 and 0.4 g in strength. Each mouse underwent 10 trials on the plantar surface of both hind paws, with an interval of 10 min between each trial. The instances of paw withdrawal were recorded and the percentage was subsequently calculated. Before the commencement of the experiments, animals were acclimated to the experimental environment for a period of 30 min.

Thermal Hyperalgesia: mice were placed in individual transparent enclosures on top of a Hargreaves radiant heat apparatus (IITC/Life Science, USA). The heat source was adjusted to emit a focused beam of radiant heat onto the plantar surface of the hind paw. The latency to withdraw the paw (paw withdrawal latency, PWL) was recorded automatically when the mouse exhibited a clear, reflexive paw lift. Each mouse was tested three times with a 10‐min interval between tests. The average of these three measurements was used for analysis.

### 2.5 Measure of blood glucose

The blood glucose levels were measured using the Accu‐Chek Active glucometer. Blood glucose was determined on days 3, 7, 14, and 21 after STZ administration.

### 2.6 RNA sequencing and analysis

RNA sequencing and analysis were performed on samples extracted from the SDH of mice. Immediately following extraction, the samples were rapidly frozen in liquid nitrogen to preserve RNA integrity for subsequent mRNA microarray analysis. Reverse transcription of mRNA was conducted to generate cDNA, and an amplified cDNA library was constructed. The mRNA expression levels were calculated using FPKM method, which refers to the number of fragments per kilobase in length from a protein‐coding gene per million fragments. The differentially expressed genes (DEGs) were carried out by “limma” package, with significance defined as adj. *p* < 0.05 and a fold change (FC) ≥ 2. Gene Ontology (GO) annotation and Kyoto Encyclopedia of Genes and Genomes (KEGG) pathway enrichment analyses were carried out using the R package clusterProfiler. Co‐expressed DEGs were imported to the online STRING database for protein–protein interaction (PPI) analysis. Use the online analysis website (www.xiantaozi.com) for visualization. RNA sequencing data have been deposited in the SRA database with the accession number PRJNA1049613.

### 2.7 Primary microglia and BV‐2 cell culture

The newborn mice were anesthetized and euthanized, followed by thorough disinfection using 75% ethanol. The mice brains were harvested and immersed in pre‐cold PBS to facilitate the removal of meninges and blood vessels. The brain tissue was minced using precision micro‐scissors and introduced into a 0.125% trypsin solution for 15 min at 37°C. DMEM medium supplemented with 10% FBS was applied to halt the digestion. Following a centrifugation step at 1000 rpm for 5 min, the supernatant was decanted and the cell pellet was resuspended in a culture medium containing 10% FBS. The resulting cell suspension was then carefully transferred into T25 culture flasks and situated within a 37°C incubator with 5% CO_2_ for an overnight incubation period. On the subsequent day, the culture medium was changed, and this process was reiterated every 5 days. Following a fortnight of cultivation, the culture flasks were oscillated at 180 rpm and 37°C with a constant temperature shaker. This procedure facilitated the separation of microglia from astrocytes and neurons. The supernatant was collected and subsequently introduced into new culture flasks, culminating in the isolation of a refined population of microglial cells.

The BV‐2 cell line was purchased from Quicell company (Shanghai, China) and cultured in a 37°C incubator with 5% CO_2_. The medium was refreshed daily until reaching 80% confluence for subculturing.

### 2.8 Western blotting

Protein samples were extracted from the spinal cord dorsal horn and cultured microglia using a RIPA lysis buffer (Epizyme, shanghai, China) supplemented with protease and phosphatase inhibitors (Epizyme, shanghai, China). The protein concentration was determined using a BCA Protein Assay Kit (Epizyme, shanghai, China). Equal amounts of protein (20 μg) were separated on a 10% SDS‐PAGE gel and then transferred onto a polyvinylidene fluoride membrane (Millipore, Sigma, USA). The membrane was blocked with 5% non‐fat milk in Tris‐buffered saline with 0.1% Tween‐20 (TBST) for 1 h at room temperature.

Primary antibodies were diluted according to the manufacturer's instructions and incubated with the membrane overnight at 4°C. After thorough washing with TBST, the membrane was incubated with the corresponding HRP‐conjugated secondary antibodies (1:5000) for 1 h at room temperature. Protein bands were visualized using an enhanced chemiluminescence reagent (Bio‐Rad, USA).

### 2.9 Q‐PCR

Total RNA was extracted from the spinal cord dorsal horn and cultured microglia using TRIzol reagent (Vazyme, Nanjing, China) according to the manufacturer's instructions. The RNA concentration and purity were determined using a Nanodrop spectrophotometer (Thermo Fisher Scientific, USA). Complementary DNA (cDNA) was synthesized from 1 μg of total RNA using the reverse reagent kit (Vazyme, Nanjing, China). Real‐time qPCR was performed using the QuantStudio5 (Thermo Fisher Scientific, USA) with SYBR Green PCR Master Mix (Vazyme, Nanjing, China). Specific primer pairs for the target gene were designed and synthesized by Sangon Biotech (Shanghai, China). The relative expression levels of target genes were calculated using the 2^−ΔΔCt^ method, normalizing the expression of the reference gene. The primer sequences used in the experiments can be found in Table 1.

**TABLE 1:** Primer sequences.

Gene	Forward	Reverse
Mouse TNF‐α	5′‐GTAGCCCACGTCGTAGCAAA‐3′	5′‐ACAAGGTACAACCCATCGGC‐3′
Mouse IL‐1β	5′‐AGAGCCCATCCTCTGTGACT‐3′	5′‐GCTCATATGGGTCCGACAGC‐3′
Mouse IL‐6	5′‐TAGTCCTTCCTACCCCAATTTCC‐3′	5′‐TAGTCCTTCCTACCCCAATTTCC‐3′
Mouse Sirt3	5′‐ATCCCGGACTTCAGATCCCC‐3′	5′‐CAACATGAAAAAGGGCTTGGG‐3′
Mouse Sirt3 promoter	5′‐ACAGCGTCAACTCCCACT‐3′	5′‐ATCCGTTTCTTCACATTAGG‐3′
Mouse β‐Actin	5′‐AACAGTCCGCCTAGAAGCAC‐3′	5′‐CGTTGACATCCGTAAAGACC‐3′

### 2.10 Immunofluorescence

Following fixation, the L4‐L6 spinal cord segments were dissected. Tissues were permeabilized with 0.3% Triton X‐100 in PBS for 30 min, followed by blocking with 5% bovine serum albumin (BSA) for 1 h at room temperature. Primary antibodies were applied overnight at 4°C.

For immunofluorescence analysis of cultured cells, cells were first grown on coverslips in appropriate culture dishes. Upon reaching the desired confluence, cells were fixed with 4% paraformaldehyde for 15 min at room temperature. Cells were permeabilized with 0.3% Triton X‐100 in PBS for 10 min, followed by blocking with 5% BSA for 1 h at room temperature. Primary antibodies were applied overnight at 4°C.

Subsequently, tissues or cells were washed with PBS and incubated with corresponding secondary antibodies conjugated with fluorophores for 2 h at room temperature. DAPI counterstaining was performed to visualize nuclei. Immunofluorescent images were captured using a high‐resolution CCD Spot camera equipped with appropriate filters for each fluorophore.

### 2.11 Transmission electron microscopy

For ultrastructural analysis of spinal cord tissue, small sections (approximately 1 mm^3^) were carefully excised and immediately fixed with 2.5% glutaraldehyde in 0.1 M phosphate buffer (pH 7.4) at 4°C for 2 h. Samples were then washed three times with phosphate buffer and post‐fixed with 1% osmium tetroxide in phosphate buffer for 1.5 h. The specimens were then dehydrated in a graded ethanol series (30%, 50%, 70%, 90%, and 100%) and subsequently infiltrated with epoxy resin. Polymerization was carried out at 60°C for 48 h. Ultrathin sections of 60 nm were cut using an ultramicrotome and contrasted with uranyl acetate and lead citrate. The sections were examined under a transmission electron microscope (Thermo Scientific Talos F200S) operating at suitable accelerating voltages.

### 2.12 Lentiviral transduction

For lentiviral transduction, BV‐2 cells were seeded in a 6‐well plate at a density of 1 × 10^5^ cells per well and allowed to adhere overnight. Polybrene (8 μg/mL) was included in the culture medium to enhance viral transduction efficiency. The cells were then incubated with the lentiviral vectors containing the target gene for 24 h. After incubation, the viral supernatant was replaced with a fresh complete DMEM medium, and the cells were allowed to recover for an additional 24 h. Puromycin (5 μg/mL) was added to the culture medium. The culture medium containing the antibiotic was refreshed every 3 days to maintain consistent selection pressure. This process continued for a period of 2 weeks until resistant colonies emerged.

### 2.13 Metabolite measurement

The SDH, cultured primary microglia, and BV‐2 cell are collected for metabolite analysis. Lactate production was determined using colorimetric method lactic acid (LA) content assay kit (Sangon Biotec, Shanghai, China) according to the manufacturer's instructions. Pyruvate production was determined using colorimetric method pyruvate (PA) content assay kit (Sangon Biotec, Shanghai, China), according to the manufacturer's instructions.

### 2.14 Extracellular flux assay

Primary microglia or Bv‐2 cells were plated in Seahorse XF24 microplates at a density of 5 × 10^4^ cells per well. The Seahorse XFe24 Analyzer (Agilent Technologies Co, USA) was employed to assess the extracellular acidification rate (ECAR) and oxygen consumption rate (OCR). The culture medium was substituted with XF assay medium comprising 10 mmol/L glucose, 2 mmol/L L‐glutamine, and 1 mmol/L sodium pyruvate. Subsequently, plates were incubated at 37°C in a non‐CO_2_ incubator for 1 h before being loaded into the XF24 Analyzer. For the ECAR determination, initial acidification levels were recorded, followed by injections of antimycin A/rotenone (Rot/AA) and 2‐DG to assess compensatory glycolysis and subsequent post‐2‐DG acidification. In the assessment of OCR, initial baseline measurements were recorded. Subsequently, oligomycin was introduced to calculate ATP‐linked OCR, FCCP for determining maximal OCR, and Rot/AA for determining reserve capacity.

### 2.15 Cell proliferation assay using CCK‐8

Cell proliferation rate was determined using the Cell Counting Kit‐8 (CCK‐8) reagent (Beyotime Institute of Biotechnology, China). BV‐2 cells were evenly distributed in a 96‐well culture plate at a density of 1 × 10^4^ cells per well. Prepare a working solution by mixing the CCK‐8 reagent with the culture medium at a ratio of 1:10 and replace the old medium with the working solution containing CCK‐8. Incubate at 37°C in the cell culture incubator for 2 h. Measure the absorbance at 450 nanometers using a microplate reader.

### 2.16 The dual‐luciferase reporter assay

293T cell lines were seeded in 24‐well plates and co‐transfected with the luciferase reporter plasmid containing the target promoter region (pGL4.10‐ Sirt3 WT promotor, pGL4.10‐ Sirt3 MUT promotor, pcDNA3.1(+)‐3xFLAG‐P2A‐EGFP, pcDNA3.1(+)‐Foxo1‐3xFLAG‐P2A‐EGFP) and the Renilla luciferase plasmid as an internal control. After 48 h, the cells were harvested, and luciferase activity was measured using the Microplate Reader (Spark 10 MTECAN) following the manufacturer's instructions. The relative luciferase activity was calculated by normalizing the firefly luciferase activity to Renilla luciferase activity. The experiments were performed in triplicate.

### 2.17 The chromatin immunoprecipitation (Ch‐IP) assay

BV‐2 cell lines were cross‐linked with 1% formaldehyde for 10 min at room temperature to stabilize protein‐DNA interactions. Glycine was added to a final concentration of 0.125 M to halt cross‐linking. The cells were incubated for an additional 5 min. Cells were harvested, lysed, and sonicated to shear chromatin into fragments averaging 200–500 base pairs. Protein‐DNA complexes were incubated with specific antibodies overnight at 4°C on a rotator to selectively isolate target DNA sequences. Antibody‐bound complexes were captured using Protein A/G beads to facilitate separation of DNA from non‐specific binding. The beads underwent a series of stringent washes with low‐salt, high‐salt, LiCl, and TE buffers to remove non‐specifically bound proteins. Cross‐links were reversed by heating at 65°C overnight. This released the DNA from the protein complexes. The DNA was used for quantitative PCR or high‐throughput sequencing to analyze the enrichment of specific DNA sequences. Results were quantified and presented as fold enrichment compared to input controls.

### 2.18 Statistical analysis

We conducted statistical analyses using IBM SPSS Statistics 23.0, and the data are expressed as mean ± SEM (standard error of the mean). To compare the blood glucose and behavioral data across various groups at different time points, we employed a two‐way repeated‐measure analysis of variance (ANOVA). Differences between the two groups were assessed using an unpaired *t*‐test, and variations among multiple groups were evaluated through one‐way ANOVA, followed by Dunnett's t‐test for post hoc analysis. Before conducting these analyses, we assessed the normality of the data distributions using the Shapiro–Wilk test. For data that did not exhibit a normal distribution, we utilized non‐parametric equivalents. A significance level of *p* < 0.05 was employed to determine statistical significance.

## 3 RESULTS

### 3.1 Decreased Sirt3 expression contributed to the pathology of DNP

**FIGURE 1:**
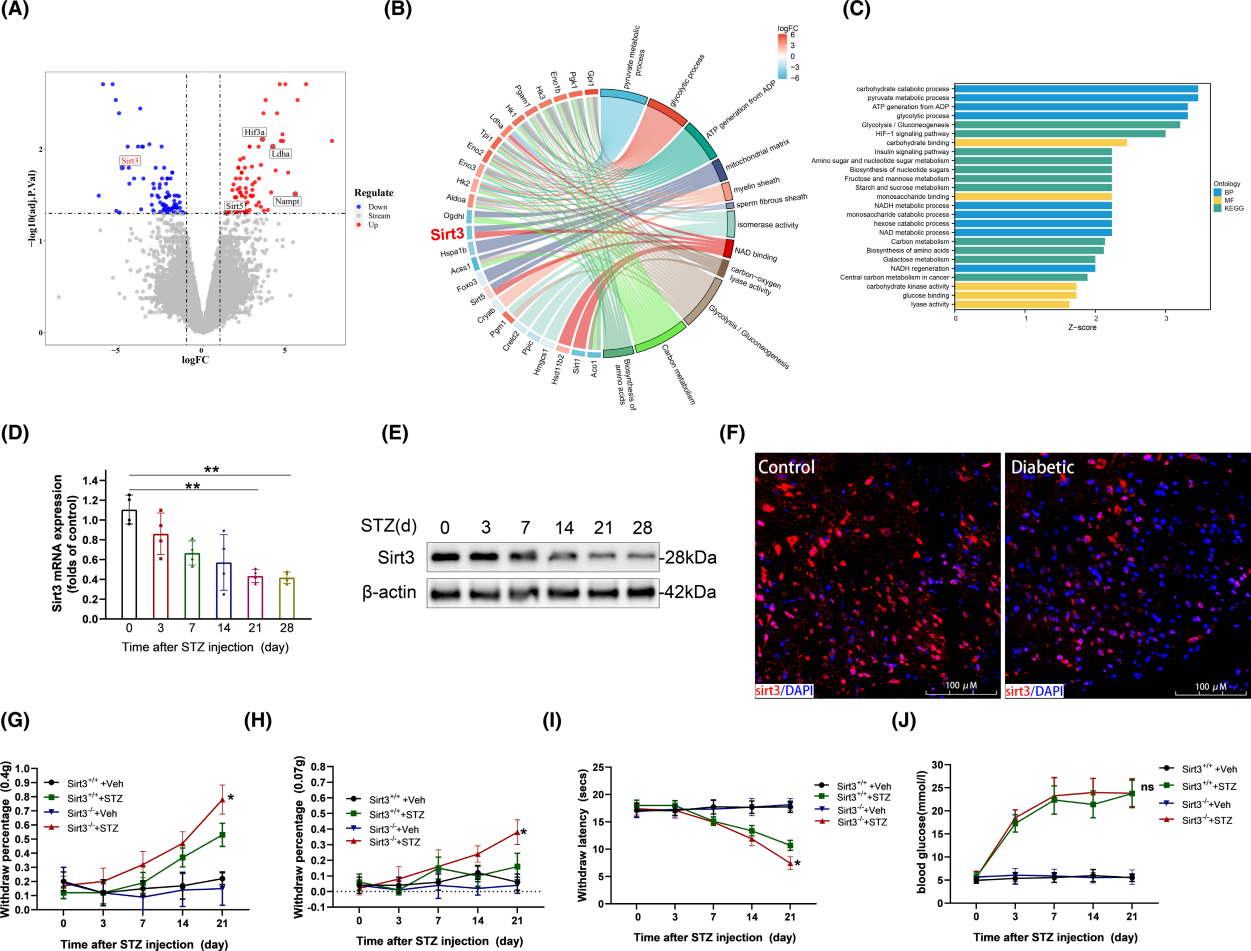
Identification of the role of Sirt3 in DNP Pathogenesis. (A) Volcano plot shows DEGs between SDH of diabetic mice and control; *n* = 3. (B) Chord plot shows DEGs related to Glycolysis/Gluconeogenesis. (C) GO terms and KEGG enrichment of the DEGs. (D–F) the transcription (D) and protein production (E, F) of Sirt3 in spinal dorsal horn after STZ injection (*n* = 4, **p < 0.01). (G, H) Paw withdrawal percentage in response to von Frey filament (0.4 g, G; 0.07 g, H) in Sirt3^−/−^ group and Sirt3^+/+^ group after STZ infection; *n* = 10, *p < 0.05 compared Sirt3^−/−^ DNP group versus Sirt3^+/+^ DNP group. (I) Paw withdrawal latency in response to heat stimulus in Sirt3^−/−^ group and Sirt3^+/+^ group after STZ infection; *n* = 10, *p < 0.05 compared Sirt3^−/−^ DNP group versus Sirt3^+/+^ DNP group. (J) Blood glucose in Sirt3^−/−^ group and Sirt3^+/+^ group after STZ infection; *n* = 10, ns *p* > 0.05 compared Sirt3^−/−^ DNP group versus Sirt3^+/+^ DNP group.

To investigate the molecular mechanisms underlying DNP, we performed mRNA microarray analysis on SDHs obtained from DNP mice (*n* = 3) and control counterparts (*n* = 3). The volcano plot illustrated distinctive gene expression profiles in murine DNP and control cohorts, revealing 180 DEGs, comprising 91 upregulated genes and 89 downregulated genes (Figure 1A). The GO analysis results revealed that the DEGs were primarily associated with biological processes related to carbohydrate catabolism and pyruvate metabolism and the enriched KEGG pathways primarily involve Glycolysis/Gluconeogenesis and the HIF‐1 signaling pathway. Furthermore, to better characterize the role of Sirt3 in the progression of DNP, we used the chord plot that showed significant Sirt3‐related GO and KEGG pathways (Figure 1B,C). Sirt3, the most potent deacetylase in vivo, plays a crucial role in maintaining mitochondrial homeostasis and regulating glucose metabolism. We observed a significant time‐dependent decrease in both Sirt3 mRNA and protein levels in the SDHs of type 1 diabetic mice (Figure 1D–F). To investigate the role of Sirt3 in pathology of DNP, we generated Sirt3‐deficient mice (Sirt3^−/−^) as previously reported.^30^ Sirt3 deficiency was confirmed through DNA gel electrophoresis and Q‐PCR (Figure S1A,B). At 21 days post‐STZ injection, Sirt3^−/−^ mice exhibited significantly increased levels of mechanical pain allodynia and thermal hyperalgesia compared to their Sirt3^+/+^ counterparts (Figure 1G–I). Importantly, Sirt3 deficiency did not significantly influence blood glucose levels in either normal or diabetic mice (Figure 1J), highlighting the specificity of changes in pain phenotype in this context. These findings underscore the pivotal role of Sirt3 in modulating pain responses in DNP.

### 3.2 Sirt3 deficiency enhanced neuroinflammation in DNP mice by increasing glycolysis

**FIGURE 2:**
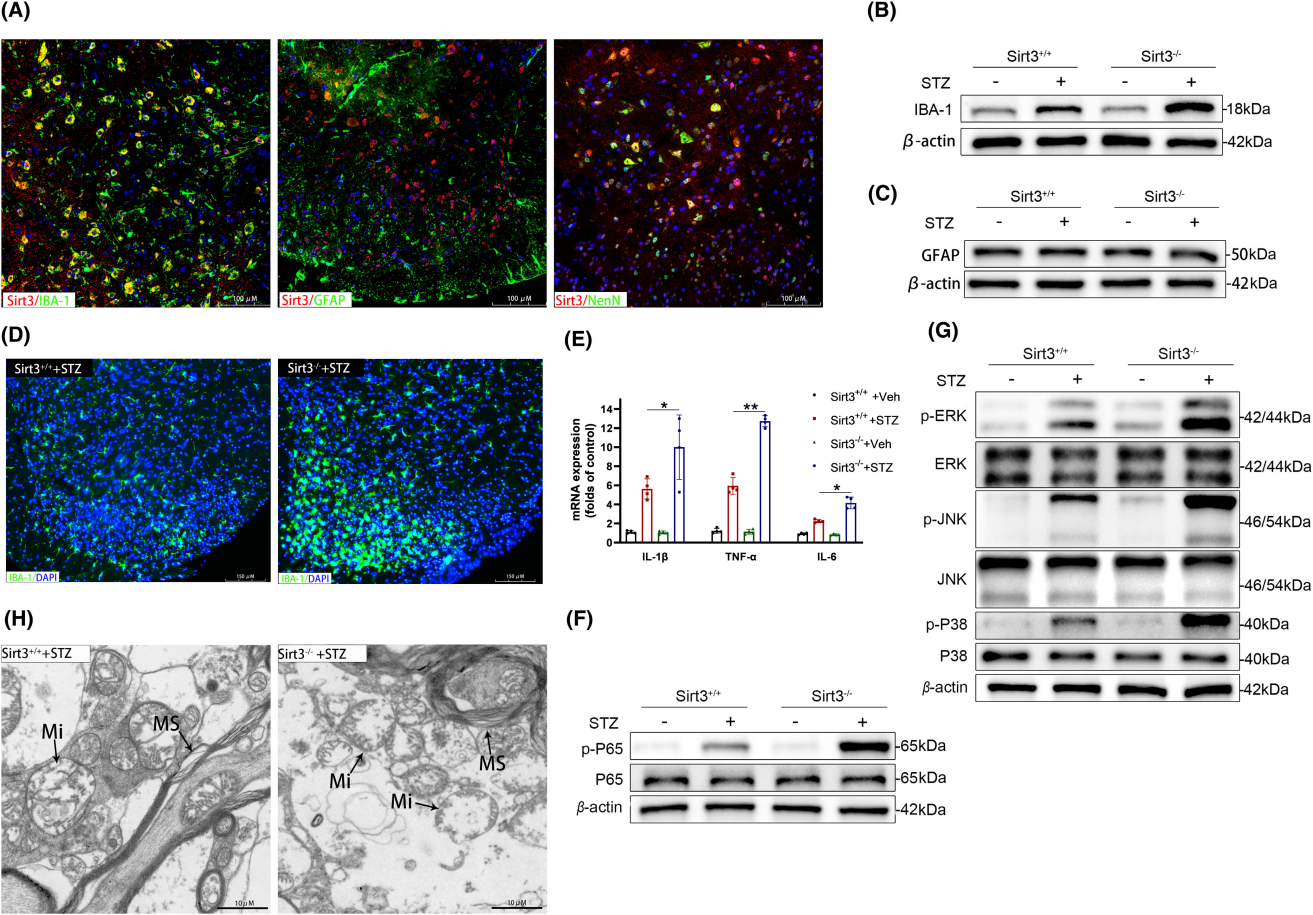
Sirt3 deficiency exacerbated neuroinflammation in the SDH in vivo. (A) Co‐expression of Sirt3 and IBA‐1, GFAP, and NeuN in SDH after STZ infection. (B, C) Protein level of IBA‐1 (B) and GFAP (C) in spinal dorsal horn (SDH) of Sirt3 deficient DNP mice. (D) Immunofluorescence shows the upregulation of IBA‐1 in SDH of Sirt3 deficient DNP mice. (E) Transcriptions of IL‐1β, TNF‐α and IL‐6 in SDH of Sirt3 deficient DNP mice (*n* = 4, *p < 0.05, **p < 0.01). (F, G) Activation of MAPK (F) and NF‐κb (G) pathway in SDH of Sirt3 deficient DNP mice. (H) Transmission electron microscopy shows myelin sheath (MS) and mitochondria (Mi) in the SDH of Sirt3 deficient DNP mice.

To explore the function of Sirt3 in DNP, we used fluorescence immunohistochemical staining by IBA‐1(a specific microglia marker), GFAP (a specific astrocyte marker), and NeuN (a specific neuron marker) to detect the major cells that express Sirt3. Sirt3 is expressed in microglia and neurons, but not in astrocyte cells in the SDH of normal raised WT mice (Figure 2A). At 21 days post‐STZ injection, Sirt3 deficiency led to an elevation in the protein expression of IBA‐1 in the SDH, while having no effect on the expression of GFAP (Figure 2B,C). The upregulation of IBA‐1 expression was further validated through immunofluorescence analysis (Figure 2D), which indicated that Sirt3 deficiency increased microglia proliferation in SDH. As a result of microglial activation, the transcription of inflammatory cytokines (IL‐6, IL‐1β, TNF‐α) within the SDH of Sirt3‐deficient mice significantly increased compared to that of control mice (Figure 2E). Given the well‐established roles of activated NF‐κB and MAPK signaling pathways in microglial activation and neuroinflammation, we investigated the influence of Sirt3 deficiency on their activation status. The phosphorylation levels of P65, ERK, JNK, and P38 were all elevated in Sirt3^−/−^ mice compared to their Sirt3^+/+^ counterparts (Figure 2F,G). Transmission electron microscopy revealed that in the SDH of Sirt3^+/+^ diabetic mice, myelin sheaths displayed a concentric arrangement with occasional areas showing slight relaxation and separation. Some individual mitochondria showed mild swelling. In Sirt3‐deficient diabetic mice, the structure of the myelin sheath lamellae in the SDH appeared loose, distorted, folded, or even fragmented and disintegrated. In Sirt3‐deficient diabetic mice, the demyelination changes were observed, and the mitochondria showed a higher degree of swelling compared to that of control mice (Figure 2H).

**FIGURE 3:**
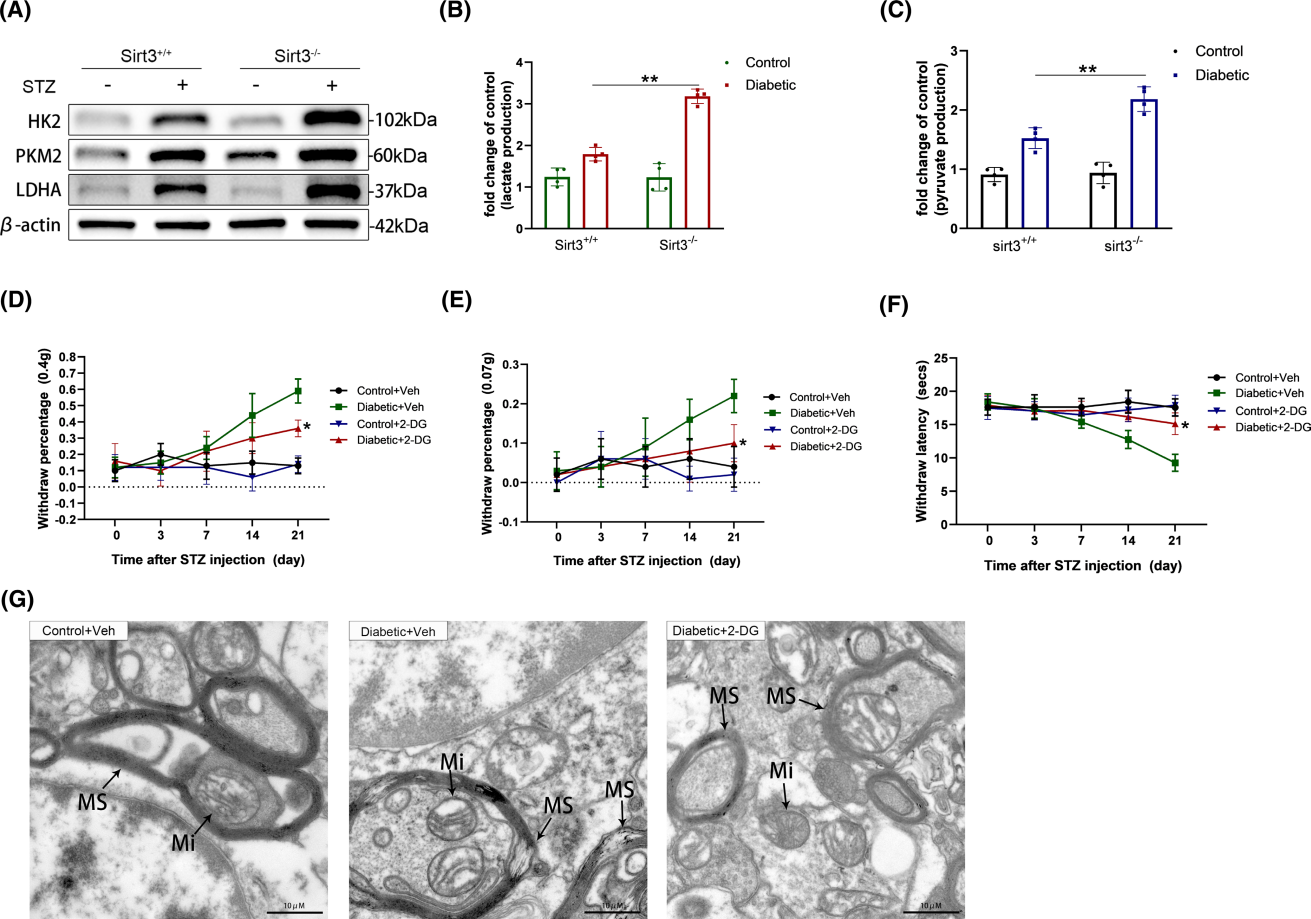
Sirt3 deficiency enhanced glycolysis in the SDH in vivo. (A) Protein expression levels of key enzymes (HK2, PKM2, LDHA) in glycolysis in SDH of Sirt3 deficient DNP mice. (B, C) glucose metabolism intermediates were changed in the SDH. Lactate levels (B) and pyruvate levels (C) increased (*n* = 4, **p < 0.01). (D, E) Paw withdrawal percentage in response to von Frey filament (0.4 g, D; 0.07 g, E) was measured in DNP mice treated intraperitoneally with 2‐DG; *n* = 10 *p < 0.05 compared 2‐DG DNP group versus vehicle DNP group. (F) Paw withdrawal latency in response to heat stimulus was measured in DNP mice treated intraperitoneally with 2‐DG; *n* = 10 *p < 0.05 compared 2‐DG DNP group versus vehicle DNP group. (G) Transmission electron microscopy shows myelin sheath (MS) and mitochondria (Mi) in the SDH of DNP mice treated intraperitoneally with 2‐DG.

Glycolysis plays a crucial role in the activation of microglia. Next, we aim to investigate whether the regulatory effect of Sirt3 on inflammation in the SDH is associated with glycolysis. The key enzymes in glycolysis (HK2, PKM2, LDHA) were utilized to assess glycolytic activity in SDH during DNP. Western blot analysis confirmed a substantial increase in the protein expression of these key enzymes on day 21 after STZ injection, and their expression was further elevated in the SDH of Sirt3‐deficient mice (Figure 3A). Furthermore, Sirt3 deficiency further increased glycolytic products lactate and pyruvate levels in the SDH of diabetic mice (Figure 3B,C). Then we investigated the effect of inhibiting diabetes‐induced glycolysis enhancement with 2‐DG on the pain phenotype and inflammatory damage in the SDH of DNP mice. Following STZ injection, intraperitoneal administration of 2‐DG led to a reduction in mechanical allodynia (Figure 3D,E) and thermal hyperalgesia (Figure 3F) in the plantar region. Transmission electron microscopy revealed that intraperitoneal administration of 2‐DG alleviated axon demyelination and reduced swollen mitochondria in the SDHs of DNP mice (Figure 3G). Importantly, administration of 2‐DG did not significantly influence blood glucose levels in either normal or diabetic mice (Figure S2A). Collectively, the glycolysis inhibitor 2‐DG attenuates diabetes‐induced neuroinflammation and pain. Indeed, this suggests that Sirt3 deficiency aggravates DNP by increasing glycolysis and neuroinflammation in vivo.

### 3.3 Sirt3 deficiency amplified aerobic glycolysis and inflammation

**FIGURE 4:**
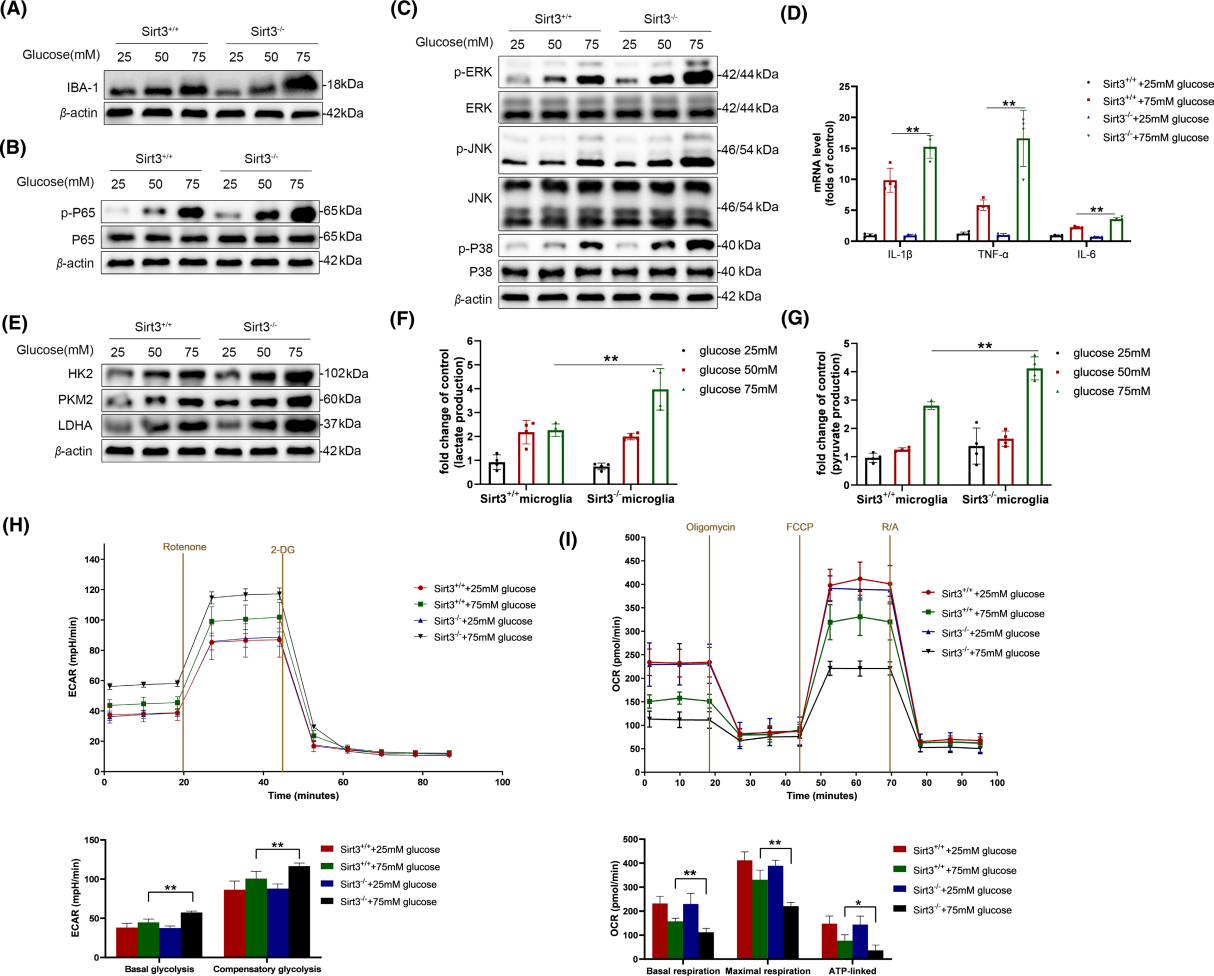
Sirt3 deficiency amplified glycolytic activity in primary microglia and intensifies their inflammatory activation in vitro. (A) Protein level of IBA‐1 in Sirt3 deficient primary microglia. (B, C) Activation of NF‐κb (B) and MAPK (C) pathway in Sirt3 deficient primary microglia. (D) Transcriptions of IL‐1β, TNF‐α and IL‐6 in Sirt3 deficient primary microglia (*n* = 4, *p < 0.05, **p < 0.01). (E) Protein expression levels of key enzymes (HK2, PKM2, LDHA) in glycolysis in Sirt3 deficient primary microglia. (F, G) Lactate levels (F) and pyruvate levels (G) in Sirt3‐deficient primary microglia (*n* = 4, *p < 0.05, **p < 0.01). (H, I) The experimental program of extracellular acidification rate (ECAR, H) and the oxygen consumption rate (OCR, I) of primary microglia, measured by Seahorse XFe24 Extracellular Flux Analyzer; *n* = 5, *p < 0.05, **p < 0.01 compared Sirt3^−/−^ high glucose group versus Sirt3^+/+^ high glucose group.

To confirm the involvement of microglial Sirt3 in glycolysis and inflammatory responses, we isolated primary microglia from both Sirt3^−/−^ and SIRT3^+/+^ mice and subsequently cultured them. Sirt3 deficiency increased the IBA‐1 expression upon high glucose stimulation in primary microglia (Figure 4A). Moreover, Sirt3 deficiency further enhanced the activation of NF‐κB and MAPK signaling pathways induced by high glucose. Compared to Sirt3^+/+^ microglia, the phosphorylation levels of P65, P38, JNK, and ERK were elevated in sirt3^−/−^ microglia under high glucose conditions (Figure 4B,C). As a consequence, Sirt3‐deficient microglia demonstrated heightened transcription of pro‐inflammatory cytokines under high glucose conditions (Figure 4D). Numerous studies have illuminated the connection between the metabolic status and microglia activation.^10^, ^31^ Metabolic reprogramming plays a pivotal role in driving microglia toward a pro‐inflammatory phenotype. To determine the involvement of Sirt3 in regulating metabolic reprogramming induced by high glucose, we examined the expression of HK2, PKM2, and LDHA, as well as lactate and pyruvate production in primary microglia. In the context of high glucose conditions, Sirt3 deficiency enhanced the upregulation of HK2, PKM2, and LDHA (Figure 4E), concomitant with increased levels of pyruvate and lactate production (Figure 4F,G). Additionally, we assessed the ECAR and the OCR in primary microglia exposed to varying concentrations of glucose. As is shown in Figure 4H, the levels of basal glycolysis and compensatory glycolytic exhibited an augmentation in response to elevated glucose levels, with Sirt3‐deficient cells displaying an even more pronounced increase. In contrast, Sirt3‐deficient cells demonstrated a more substantial decrease in basal, ATP‐linked, and maximal OCR when subjected to high glucose, as compared to Sirt3^+/+^ counterparts (Figure 4I). These results indicate that Sirt3 deficiency increases inflammation by enhancing high‐glucose‐induced aerobic glycolysis in microglia.

### 3.4 Sirt3 overexpression reduced aerobic glycolysis and inflammation in the BV‐2 cell

**FIGURE 5:**
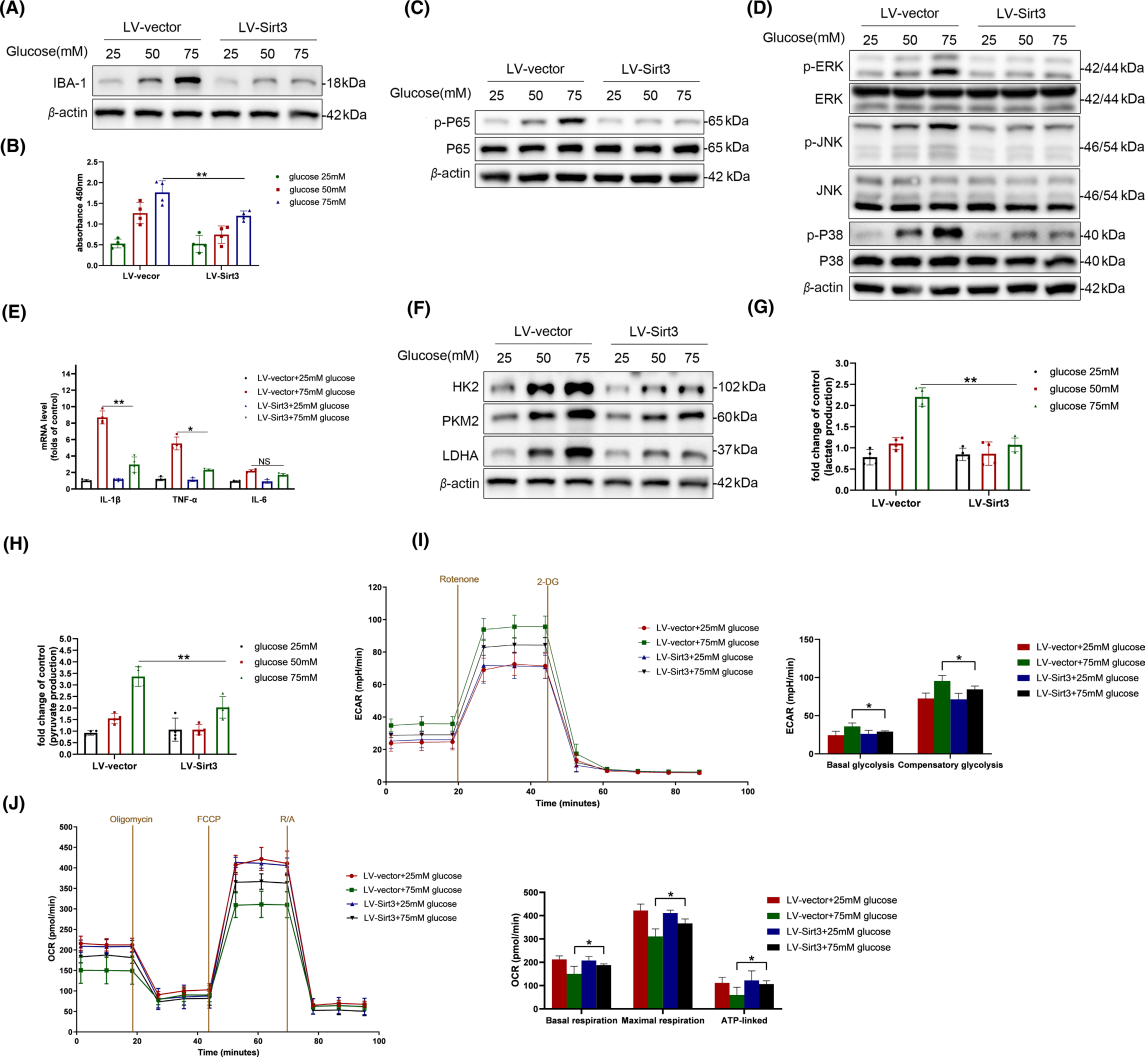
Sirt3 overexpression in BV‐2 microglia cells reduced glycolysis and reversed their inflammatory activation in vitro. (A) Protein level of IBA‐1 in Sirt3‐overexpressed BV‐2 cells. (B) cell proliferation was tested by colorimetric determination of CCK8 in Sirt3‐overexpressed BV‐2 cells; *n* = 4 **p < 0.01. (C, D) Activation of NF‐κb (C) and MAPK (D) pathway in Sirt3‐overexpressed BV‐2 cells. (E) Transcriptions of IL‐1β, TNF‐α and IL‐6 in Sirt3‐overexpressed BV‐2 cells; *n* = 4 **p < 0.01. (F) Protein expression levels of key enzymes (HK2, PKM2, LDHA) in glycolysis in Sirt3‐overexpressed BV‐2 cells. (G, H) Lactate levels (G) and pyruvate levels (H) in Sirt3‐overexpressed BV‐2 cells; *n* = 4 **p < 0.01. (I, J) Extracellular acidification rate (ECAR, I) and the experimental program of the oxygen consumption rate (OCR, J) of BV‐2 cells, measured by Seahorse XFe24 Extracellular Flux Analyzer; *n* = 5 *p < 0.05 compared LV‐Sirt3 high glucose group versus LV‐vector high glucose group.

We overexpressed Sirt3 in the BV‐2 cell line using a lentiviral vector. The overexpression efficiency of Sirt3 was confirmed by western blotting and Q‐PCR (Figure S1C,D). The Western blot results demonstrated that overexpression of Sirt3 could alleviate the upregulation of IBA‐1 induced by high glucose (Figure 5A). Additionally, under high glucose conditions, microglial proliferation was more pronounced, Sirt3‐overexpressed BV‐2 cells displayed a robust decrease in proliferation compared to their counterparts (Figure 5B). In terms of inflammation‐related signaling pathways, the overexpression of Sirt3 decreased the activation of NF‐κB and MAPK signaling pathways induced by high glucose (Figure 5C,D). Consequently, this led to a decrease in the transcription of pro‐inflammatory cytokines, including IL‐1β and TNF‐α (Figure 5E). Similar to primary microglia, BV‐2 cell line exhibited increased glycolytic activity under high glucose conditions. Overexpression of Sirt3 alleviated the upregulation of key enzymes associated with glycolysis induced by high glucose stimulation (Figure 5F). Regarding metabolites, Sirt3 overexpressed BV‐2 cells showed lower levels of pyruvate and lactate production compared to the control under high glucose conditions (Figure 5G,H). ECAR and OCR are both employed to assess the glycolytic and oxidative metabolism in BV‐2 cells as mentioned before. Overexpression of Sirt3 substantially decreased the basal glycolysis and compensatory glycolytic induced by high glucose in BV‐2 cells (Figure 5I). Additionally, lentivirus‐mediated reintroduction of Sirt3 into high glucose‐treated BV‐2 cells restored the reduction in basal, ATP‐linked, and maximal OCR (Figure 5J). These results indicate that overexpression of Sirt3 can decrease the glycolysis and inflammation in microglia upon high glucose stimulation.

### 3.5 Inactivation of FoxO1 decreased Sirt3 transcription upon high glucose stimulation

**FIGURE 6:**
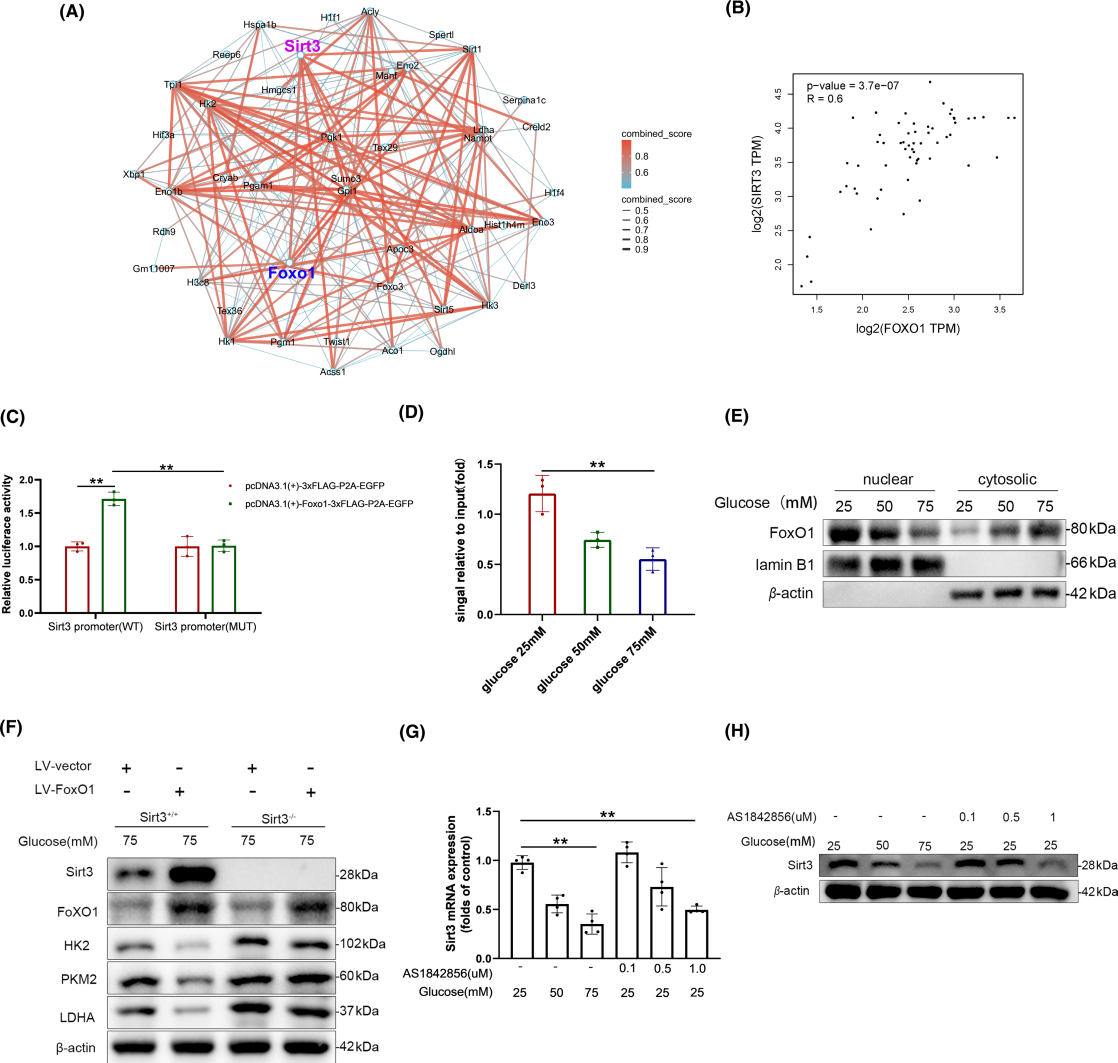
Inactivation of FoxO1 decreased Sirt3 transcription upon high glucose stimulation. (A) Network model describing protein–protein interactions between the FOXO1 and Sirt3. (B) Correlation analysis of FoxO1 and Sirt3 targets in human spinal cord tissue with GEPIA database (http://gepia.cancer‐pku.cn). (C) Luciferase reporting system evaluates the targeting effects of 293 T cells after co‐transfection with Sirt3 promoter plasmids and overexpressing‐FoxO1 plasmids; *n* = 3 **p < 0.01. (D) Ch‐IP analysis shows that the Sirt3 promoter sequence was pulled down by the anti‐FoxO1 antibody; *n* = 4 **p < 0.01. (E) Western blotting shows nuclear exclusion of FoxO1 in primary microglia under high glucose stimulation. (F) Western blotting reveals the Sirt3‐dependent impact of FoxO1 on microglial glycolysis under high glucose conditions. (G, H) the transcription (G) and protein production (H) of Sirt3 in primary microglia after AS1842856 treatment; *n* = 4 **p < 0.01.

Many studies have demonstrated that Sirt3 could maintain mitochondrial homeostasis, reduce ROS production, and the loss of Sirt3 would lead to the stabilization of HIF‐1α, thereby inducing the Warburg effect.^32^ Therefore, we are particularly interested in understanding how high glucose stimulation regulates the expression and degradation of Sirt3. We then performed network analysis to confirm the protein–protein interaction network of Sirt3 under high‐glucose conditions (Figure 6A). Next, we investigated the relationship between FoxO1 and Sirt3 targets in human spinal cord tissue with GEPIA database.^33^ Correlation analysis based on GEPIA analysis revealed a positive correlation between FoxO1 and Sirt3 (Figure 6B). To identify the precise mechanism through which FoxO1 induces the expression of Sirt3 mRNA, we examined the promoter sequence of the mouse Sirt3 gene and cloned this region into a luciferase reporter plasmid. Exogenously expressed FoxO1 proteins demonstrated the ability to activate Sirt3 luciferase reporter activity in 293 T cells. Subsequently, we performed site‐directed mutagenesis on the predicted FoxO1 binding sites within the Sirt3 promoter, leading to a significant reduction in luciferase reporter activity induced by exogenously expressed FoxO1 proteins (Figure 6C). Considering the pivotal role of FoxO1 in regulating Sirt3 expression, we conducted ChIP‐qPCR analysis and observed a notable decrease in FoxO1 enrichment on the Sirt3 promoter in primary microglia subjected to high glucose stimulation compared to the control group (Figure 6D). Phosphorylation of FoxO1 is often accompanied by cytoplasmic translocation and subsequent loss of its transcriptional activity.^34^ We investigated the cellular localization of FoxO1 in microglia by western blot and observed that with increasing glucose concentrations in the environment, there was an increased cytosolic translocation and reduced nuclear localization of FoxO1 (Figure 6E). Moreover, under high glucose stimulation, heightened glycolysis in microglia was attenuated by the overexpression of FoxO1, as reflected by decreased protein levels of key enzymes including HK2, PKM2, and LDHA. This effect was abolished upon the knockout of Sirt3, suggesting that the regulatory impact of FoxO1 on glycolysis is dependent on the presence of Sirt3 (Figure 6F). AS1842856 is a cell‐permeable inhibitor designed to target and inhibit the transcriptional activity of FoxO1. This inhibitor functions by directly binding to activated FoxO1, thereby suppressing its transcriptional activity.^35^ Under a glucose concentration of 25 mM, we observed a significant concentration‐dependent reduction in both Sirt3 mRNA and protein levels in microglia treated with AS1842856 (Figure 6G,H). These results suggest that the downregulation of Sirt3 induced by high glucose is dependent on FoxO1.

### 3.6 Akt inactivated FoxO1 upon high glucose stimulation in microglia

**FIGURE 7:**
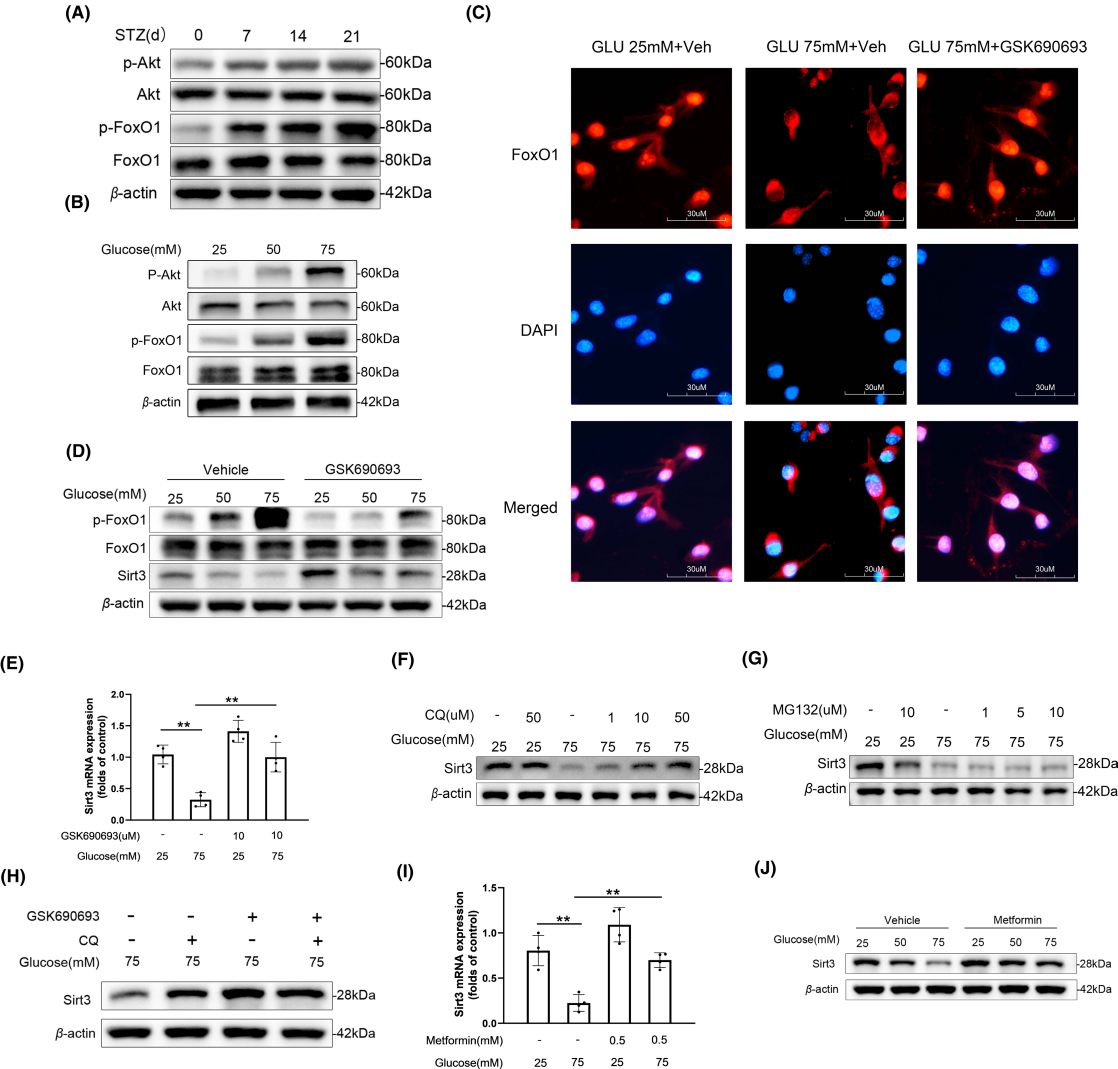
Akt inactivated FoxO1 upon high glucose stimulation in microglia. (A) Time‐dependent phosphorylation of Akt and FoxO1 in the spinal dorsal horn of DNP mice. (B) Phosphorylation of Akt and FoxO1 in primary microglia under high glucose conditions. (C) Immunofluorescence analysis of FoxO1 translocation in microglia upon treatment with GSK690693. (D) Western blot shows the impact of GSK690693 on FoxO1 phosphorylation and Sirt3 downregulation under high glucose stimulation. (E) quantitative PCR analysis of Sirt3 expression under high glucose stimulation with and without GSK690693 treatment; *n* = 4 **p < 0.01. (F, G) Western blot shows impact of CQ (F) and MG132 (G) on Sirt3 expression in microglia under high glucose stimulation. (H) Effects of chloroquine on Sirt3 protein levels in microglial cells under high glucose conditions with GSK690693 treatment. (I, J) The transcription (I) and protein level (J) of Sirt3 in microglia after metformin treatment; *n* = 4 **p < 0.01.

Many studies have confirmed the pivotal role of Akt as a key enzyme in the regulation of intracellular glucose metabolism.^36^ In addition to directly phosphorylate several metabolic enzymes, Akt also serves to regulate the FoxO family of transcription factors.^37^ So, we wonder if the impact of high glucose on FoxO1 may be connected to Akt activity. The Western blot results revealed a substantial increase in the phosphorylation of Akt at Ser‐473 in the SDH of DNP mice (Figure 7A). Consistently, similar outcomes were observed in vitro (Figure 7B). GSK690693 is a pan‐Akt inhibitor targeting Akt1/2/3.^38^ Under basal conditions, FoxO1 is primarily localized in the nucleus. Treatment with GSK690693 could reverse the high glucose‐induced translocation of FoxO1 from the nucleus to the cytoplasm in microglial cells, as indicated by immunofluorescence results (Figure 7C). Similarly, we validated these findings using Western blotting, demonstrating that GSK690693 could alleviate the phosphorylation of FoxO1 and the corresponding downregulation of Sirt3 induced by high glucose stimulation (Figure 7D). Building on these findings, we employed Q‐PCR to further confirm at the transcriptional level the regulatory effect of GSK690693 on Sirt3 expression (Figure 7E). The mitophagy‐lysosomal pathway (ALP) and the ubiquitin‐proteasome system (UPS) are two primary mechanisms for intracellular protein degradation.^39^ To further investigate the mode of Sirt3 degradation under high glucose stimulation, we treated microglia in a high‐glucose environment with the lysosome acidification inhibitor, CQ, and the proteasome inhibitor, MG132. The Western blot results indicate that the downregulation of Sirt3 expression can be blocked by CQ but not MG132 (Figure 7F,G). This suggests that downregulation of Sirt3 is induced by both Akt‐FoxO1 signaling and the ALP upon high glucose. Besides this, in microglial cells stimulated with high glucose and treated with GSK690693, CQ was unable to further increase the protein levels of Sirt3 (Figure 7H). Metformin is an oral medication commonly used to treat type 2 diabetes. The main mechanism of metformin involves activating AMP‐activated protein kinase (AMPK), which can impact the Akt signaling pathway.^40^ So, we wonder whether metformin has the same effects as the Akt inhibitor GSK690693. We have shown that metformin effectively alleviated the high glucose‐induced reduction in Sirt3 expression, as evidenced by both mRNA and protein level (Figure 7I,J). This suggests that hyperglycemia‐activated Akt signaling is critical for the decreased expression of Sirt3, which implies that dairy oral take metformin may sustain Sirt3 expression and inhibit DNP.

### 3.7 Metformin alleviated neuroinflammation and diabetic neuropathic pain by rescuing hyperglycemia‐induced Sirt3 downregulation

**FIGURE 8:**
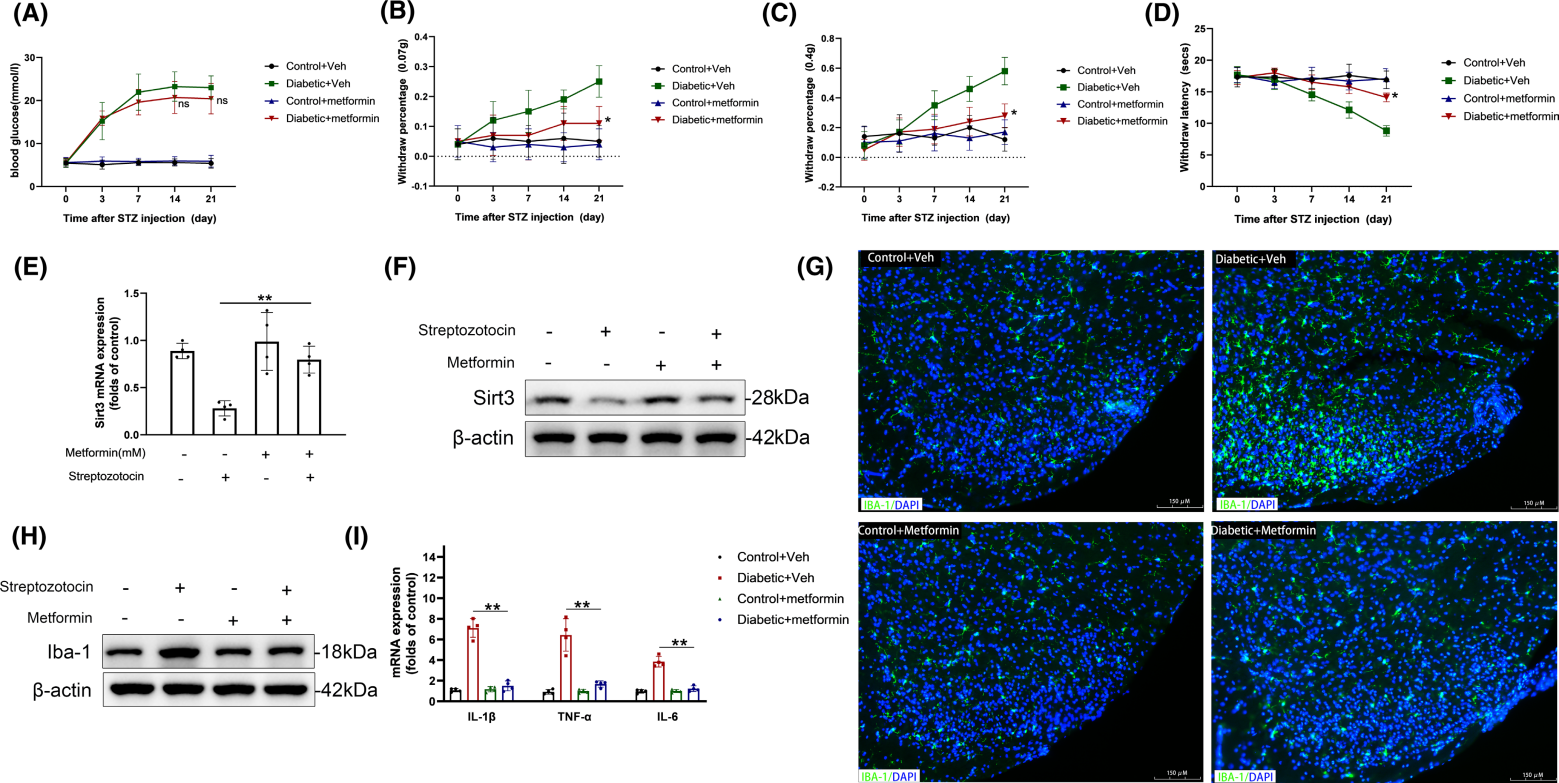
Metformin alleviated neuroinflammation and DNP by regulating Sirt3 expression. (A) Blood glucose was measured in DNP mice treated intraperitoneally with metformin; *n* = 10, ns *p* > 0.05 compared metformin DNP group versus vehicle DNP group. (B, C) Paw withdrawal percentage in response to von Frey filament (0.07 g, B; 0.4 g, C) was measured in DNP mice treated intraperitoneally with metformin; *n* = 10 *p < 0.05, **p < 0.01 compared metformin diabetic group versus vehicle diabetic group. (D) Paw withdrawal latency in response to heat stimulus was measured in DNP mice treated intraperitoneally with metformin; *n* = 10 *p < 0.05 compared metformin diabetic group versus vehicle diabetic group. (E, F) The transcription (E) and protein level (F) of Sirt3 in spinal dorsal horn (SDH) after metformin treatment; *n* = 4 **p < 0.01. (G) Immunofluorescence shows the upregulation of IBA‐1 in SDH of DNP mice treated intraperitoneally with metformin. (H) Protein level of IBA‐1 in SDH of DNP mice treated intraperitoneally with metformin. (I) Transcriptions of inflammatory cytokines (IL‐1β, TNF‐α, IL‐6) in SDH of DNP mice treated intraperitoneally with metformin; *n* = 4 **p < 0.01.

Finally, we explored the therapeutic potential of metformin in addressing DNP through daily intraperitoneal administration. Metformin exhibited the capability to reduce blood glucose levels; however, the observed differences compared to the control group did not reach statistical significance (Figure 8A). After STZ injection, metformin treatment significantly alleviated the mechanical pain allodynia and thermal hyperalgesia in the plantar region (Figure 8B–D). Additionally, we observed that intraperitoneal administration of metformin rescued the hyperglycemia‐induced downregulation of Sirt3 at both mRNA and protein levels (Figure 8E,F). Given the established association between Sirt3 and microglial inflammatory activation, we further explored the impact of metformin on the expression of IBA‐1 within the SDH. Immunofluorescence analysis shows that metformin reduced the hyperglycemia‐induced increase in IBA‐1 expression, suggesting its role in modulating microglial activation (Figure 8G). These observations were further confirmed by Western blot (Figure 8H). As a result of attenuated microglial activation, Metformin alleviated the upregulation of inflammatory cytokine transcription caused by hyperglycemia (Figure 8I). To further confirm the analgesia and neuroprotective effect depends on regulation of Sirt3, we explored whether metformin can exert the same effects in Sirt3^−/−^ DNP mice as it does in wild‐type mice. In Sirt3^−/−^ DNP mice, metformin failed to alleviate the thermal hyperalgesia and tactile allodynia induced by hyperglycemia (Figure S2B–D). In terms of neuroinflammation, the administration of metformin in Sirt3^−/−^ DNP mice had no effect on the expression of IBA‐1 in the SDH (Figure S2E). As a consequence, the administration of metformin did not significantly influence mRNA levels of inflammatory cytokines in Sirt3^−/−^ DNP mice (Figure S2F). Collectively, metformin can decrease neuroinflammation and DNP, and this effect depends on its regulation of Sirt3 protein level.

## 4 DISCUSSION

Sirt3, a mitochondrial deacetylase, is primarily enriched in metabolically active tissues such as the brain, heart, liver, brown adipose tissue, and others.^41^ It plays a crucial role in nearly all mitochondrial biological processes, including mitochondrial dynamics maintenance, OXPHOS, ATP generation, and the elimination of ROS.^42^ However, the role of Sirt3 in neuroinflammation induced by hyperglycemia remains unexplored. Here, we report for the first time that in the SDH of DNP mice, high glucose stimulation leads to the downregulation of Sirt3 protein level in microglia through the Akt/FoxO1 pathway. This downregulation results in impaired OXPHOS, a shift in metabolic pathways toward glycolysis, and subsequently, contributes to spinal inflammation damage and neuropathic pain. Mechanistically, in microglia, Akt recognizes alterations in energy signals induced by high glucose, leading to phosphorylation and nuclear exclusion of FoxO1. The reduced binding of FoxO1 to the Sirt3 promoter results in decreased mRNA transcription of Sirt3. Additionally, the lysosomal degradation of Sirt3 in a high glucose environment further decreases Sirt3 protein expression. Collectively, high glucose induces the decrease in Sirt3 expression through the Akt/FoxO1 pathway and the increase of Sirt3 degradation via the lysosome‐dependent pathway, these are crucial checkpoints in glycolytic reprogramming and neuroinflammation. Thus, our study offered novel insights into the pathology of DNP and provided clues for its therapeutic interventions.

Previous research on DNP has often been confined to the damage inflicted by high blood sugar on peripheral neurons. However, the brain and spinal cord are also crucial components in the generation of neuropathic pain. Harmful stimuli stimulate the peripheral terminals of nociceptors, these excitatory signals are transmitted through Aδ and C fibers to the SDH, where they activate nociceptive projection neurons in lamina I.^43^ Therefore, we conducted transcriptome sequencing on spinal cord tissues from mice with DNP and compared it with the control group. Our analysis revealed that Sirt3 might act as a key molecule in this pathological process. Sirt3 is synthesized in the cytoplasm as a 44 kDa form and is subsequently transported to the nucleus, where it is stored. After being transported to the mitochondria, it undergoes cleavage by matrix processing peptidase to be a 28 kDa form, playing a role in NAD‐dependent deacetylation.^44^, ^45^ Numerous studies have identified that Sirt3 can directly interact with various enzymes within the mitochondria, enhancing their activity through deacetylation, such as PDH, IDH2, and others.^46^ The results of this study unveil a critical role for Sirt3 in the pathogenesis of DNP. The observed time‐dependent decrease in Sirt3 expression in the SDHss of diabetic mice implies a potential link between Sirt3 deficiency and the development of neuropathic pain. The heightened mechanical pain allodynia and thermal hyperalgesia in Sirt3‐deficient mice further emphasize Sirt3's protective role against DNP, independent of its influence on blood glucose levels. This aligns with previous studies highlighting the protective role of Sirt3 in neuropathic pain.^47^ It's worth noting that recent studies have elucidated the involvement of Sirt3 in modulating inflammatory responses within the PNS. In a murine model of type 2 diabetes, upregulation of Sirt3 has been shown to increase motor nerve conduction velocity and alleviate hyperglycemia‐induced sciatic nerve inflammation.^48^ Activation of Sirt3 expression has also been shown to exert neuroprotective effects in models of traumatic optic nerve injury.^49^

Neuroinflammation is a key contributor to the pathogenesis of DNP, and our findings implicate Sirt3 as a crucial modulator of this inflammatory response. Sirt3 deficiency leads to microglial activation, as evidenced by increased IBA‐1 expression and elevated levels of pro‐inflammatory cytokines. The activation of NF‐κB and MAPK signaling pathways further suggests a mechanism through which Sirt3 deficiency amplifies neuroinflammation. Notably, the ultrastructural changes observed in the SDH of Sirt3‐deficient diabetic mice, including demyelination and mitochondrial swelling, provide additional insights into the impact of Sirt3 on neuroinflammatory processes in DNP.

Glycolysis emerges as a central player in the modulation of neuroinflammation, and there are reports indicating that microglia can undergo a metabolic shift from mitochondrial OXPHOS to aerobic glycolysis in response to stress conditions.^50^ Many studies have confirmed that the product of glycolysis, lactate, can enhance the microglial release of pro‐inflammatory cytokines, including tumor necrosis factor alpha (TNF‐α), interleukin‐6 (IL‐6), and interleukin‐1β (IL‐1β).^51^ Elevated glycolytic enzyme expression and increased glycolytic products in the SDH of Sirt3‐deficient diabetic mice suggest a glycolysis‐dependent mechanism in Sirt3‐mediated neuroinflammation. The attenuation of pain and neuroinflammation by the glycolysis inhibitor 2‐DG further supports the link between glycolysis and DNP pathogenesis.

Our study extends these findings to primary microglia and BV‐2 cell line cultures, demonstrating that Sirt3 deficiency amplifies glycolytic activity and inflammatory responses under high glucose conditions. The restoration of OXPHOS by Sirt3 overexpression in BV‐2 cells indicates a direct regulatory role of Sirt3 on cellular metabolism. These in vitro results complement our in vivo findings, further underscoring the importance of Sirt3 in modulating neuroinflammation and metabolic shifts associated with DNP.

FoxO1, a transcription factor categorized within the FoxO subfamily of Forkhead box (Fox) transcription factors, is crucial in modulating glucose metabolism.^52^ Specifically, it stimulates gluconeogenesis in hepatic tissues and impedes glucose uptake in insulin‐responsive tissues.^53^, ^54^ Phosphorylation events, orchestrated by kinases like Akt (protein kinase B), induce the relocation of FoxO1 from the nucleus to the cytoplasm, lead to the suppression of its transcriptional activity.^55^ Here, we also identified FoxO1 phosphorylation as the initiator of high glucose‐induced downregulation of Sirt3 expression. The decreased FoxO1 enrichment on the Sirt3 promoter and the reduction in luciferase reporter activity suggest a regulatory role of FoxO1 in Sirt3 expression. Additionally, Akt activation was identified as driving FoxO1 phosphorylation and cytoplasmic translocation, subsequently influencing Sirt3 expression. Furthermore, we investigated the mechanism underlying the degradation of Sirt3 in the context of high glucose conditions. Our findings reveal that the downregulation of Sirt3 relies on the ALP, and suggest a potential interaction between Akt signaling and lysosome‐dependent Sirt3 degradation. These findings provide additional layers to our understanding of the complex regulatory network involving FoxO1, Akt, and Sirt3 in the context of DNP. Building on these findings, we further discovered that metformin, an activator of the AMPK pathway, can rescue the reduction of Sirt3 expression in microglial cells upon high glucose stimulation. So, we investigated the therapeutic effect of metformin to assess the potential clinical translation of our findings. We demonstrated that intraperitoneal administration of metformin decreases hyperglycemia‐induced neuroinflammation and neuropathic pain, even though its blood glucose control effects are not as ideal as insulin. This indicates that, beyond its glycemic effects, metformin may have an important role in neuroinflammation and DNP.

In summary, this study unveils the interplay between Sirt3, glycolysis, and neuroinflammation in the pathological progression of DNP, targeting Sirt3 or modulating Akt/FoxO1 pathways may offer promising strategies to control neuroinflammation and alleviate pain in DNP patients.

## AUTHOR CONTRIBUTIONS

The idea was conceived by H.Y., P.Z. and C.H.; Y.L. designed and performed the in vitro experiments; E.K., R.C., and J.L. designed and performed the in vivo experiments; R.D. performed the behavioral experiments; T.H. analyzed the RNA‐seq data; M.Y. and H.W. wrote the manuscript; D.C., H.S., M.D., and H.W. supervised the study and edited the manuscript. All the authors commented on the manuscript.

## CONFLICT OF INTEREST STATEMENT

The authors declare that there are no competing interests.

## CONSENT FOR PUBLICATION

Not applicable.

## Supporting information


Figure S1.

Figure S2.

Data S1.

## Data Availability

The data that support this study are available from the corresponding author upon reasonable request. RNA sequencing data have been deposited in the SRA database with the accession number PRJNA1049613. Source data are provided with this paper.
