# Supplementary figures and images for: Hyperglycemia‐induced Sirt3 downregulation increases microglial aerobic glycolysis and inflammation in diabetic neuropathic pain pathogenesis
Source: CNS Neurosci Ther. 2024 Aug 9;30(8):e14913. doi: 10.1111/cns.14913 (PMC11315676; doi:10.1111/cns.14913)

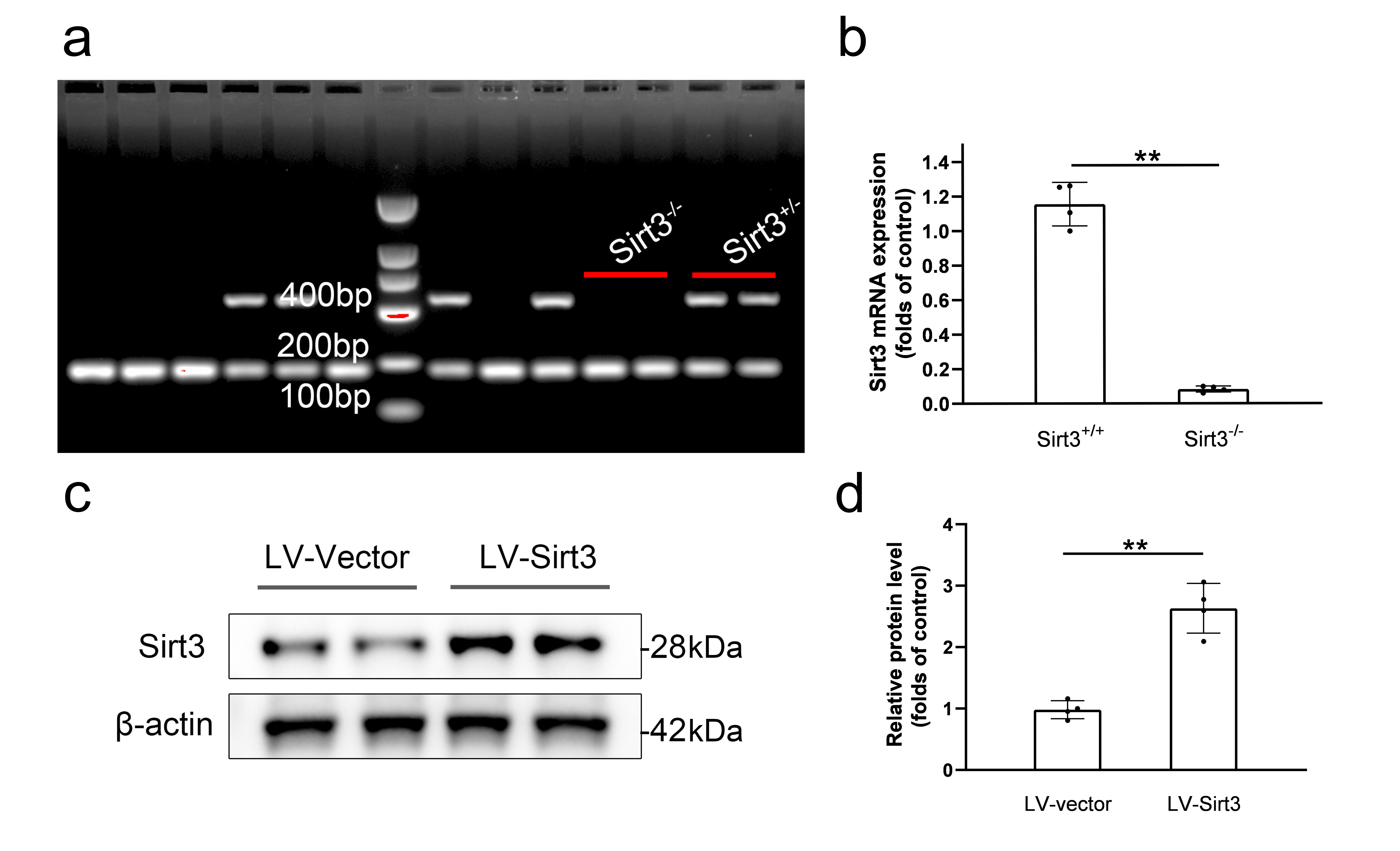

Supplement: Supplementary file 1 — Figure S1. [file CNS-30-e14913-s002.tif]

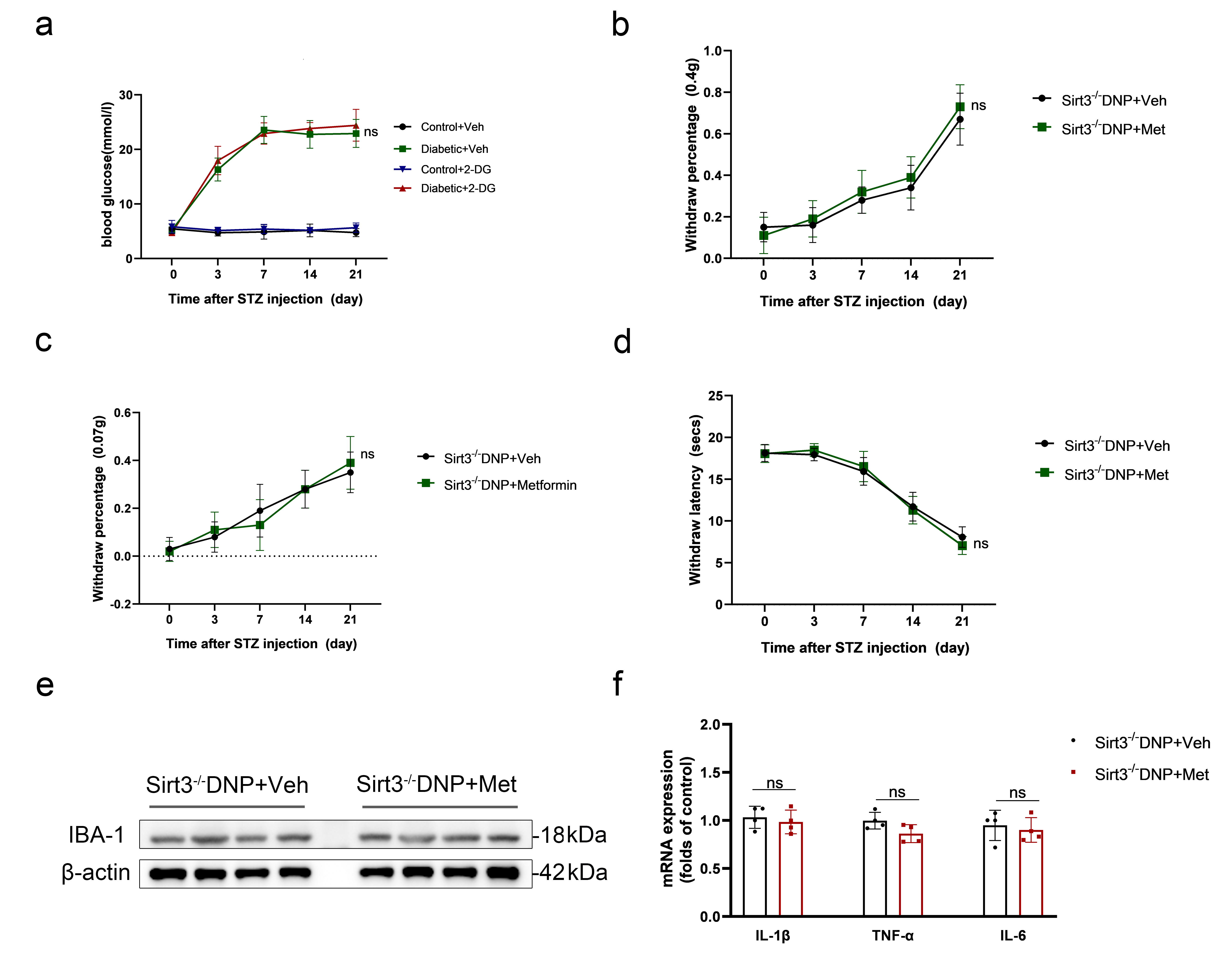

Supplement: Supplementary file 2 — Figure S2. [file CNS-30-e14913-s003.tif]
