# Supplementary material for: Hyperglycemia‐induced Sirt3 downregulation increases microglial aerobic glycolysis and inflammation in diabetic neuropathic pain pathogenesis
Source: CNS Neurosci Ther. 2024 Aug 9;30(8):e14913. doi: 10.1111/cns.14913 (PMC11315676; doi:10.1111/cns.14913)

Fig.1e

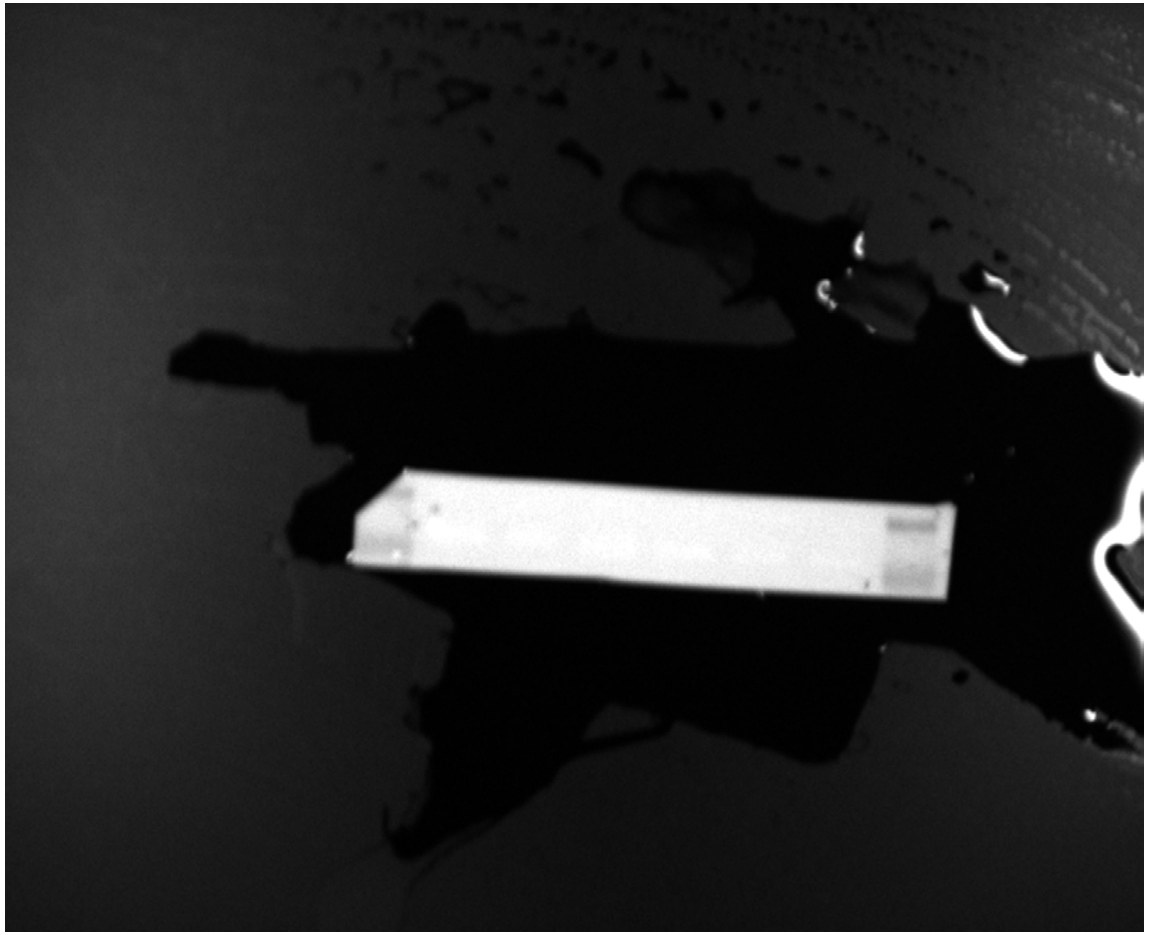

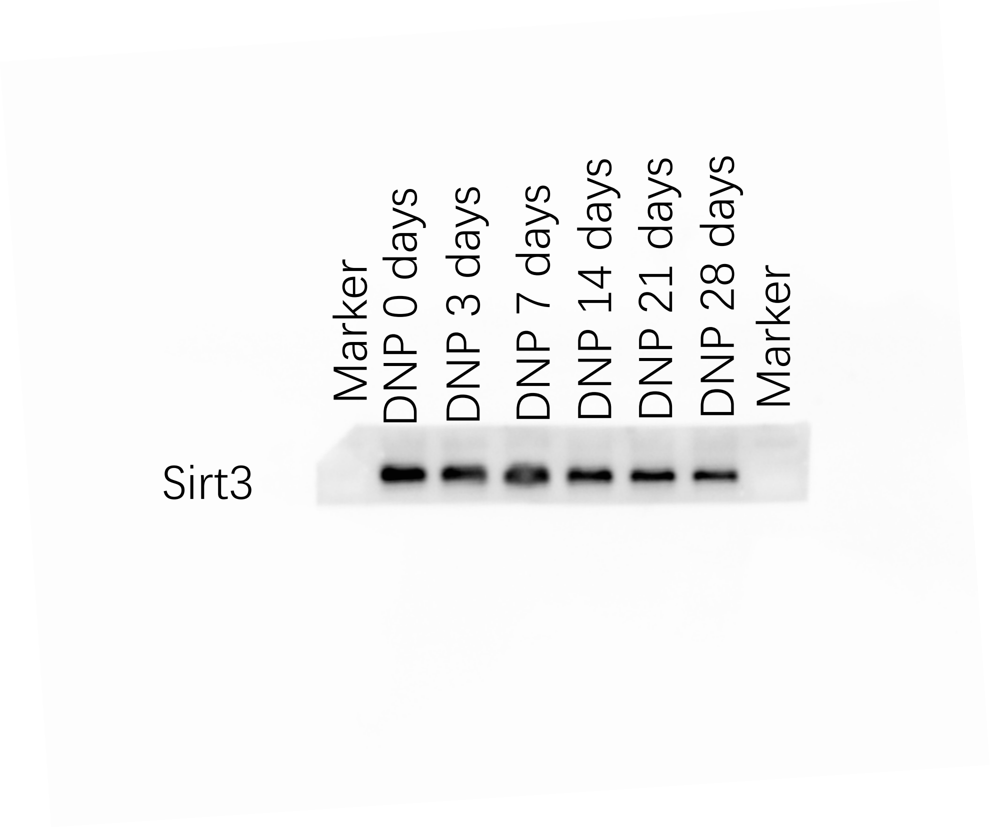
Marker
DNP 0 days
DNP 3 days
DNP 7 days
DNP 14 days
DNP 21 days
DNP 28 days
Marker
Sirt3

Fig.1e

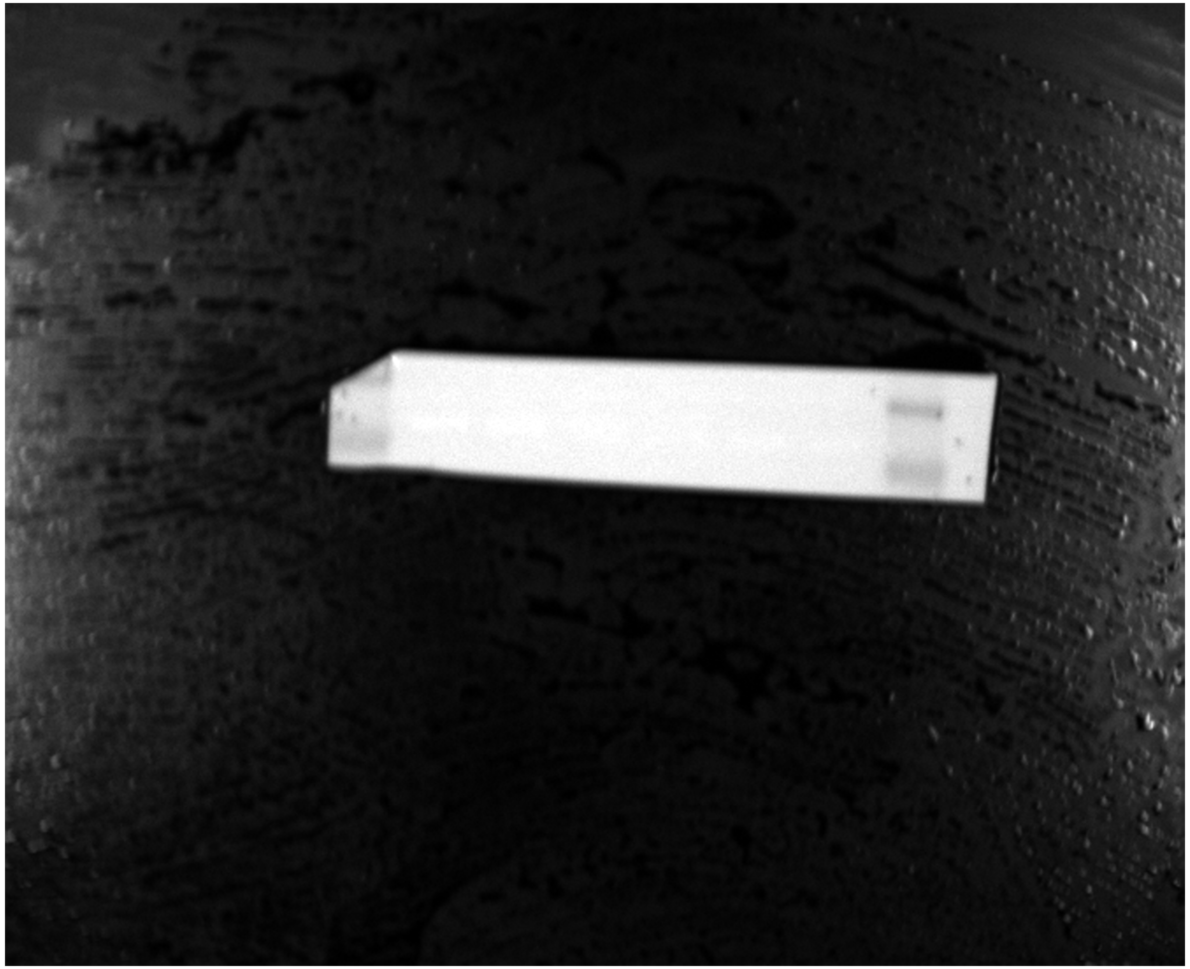

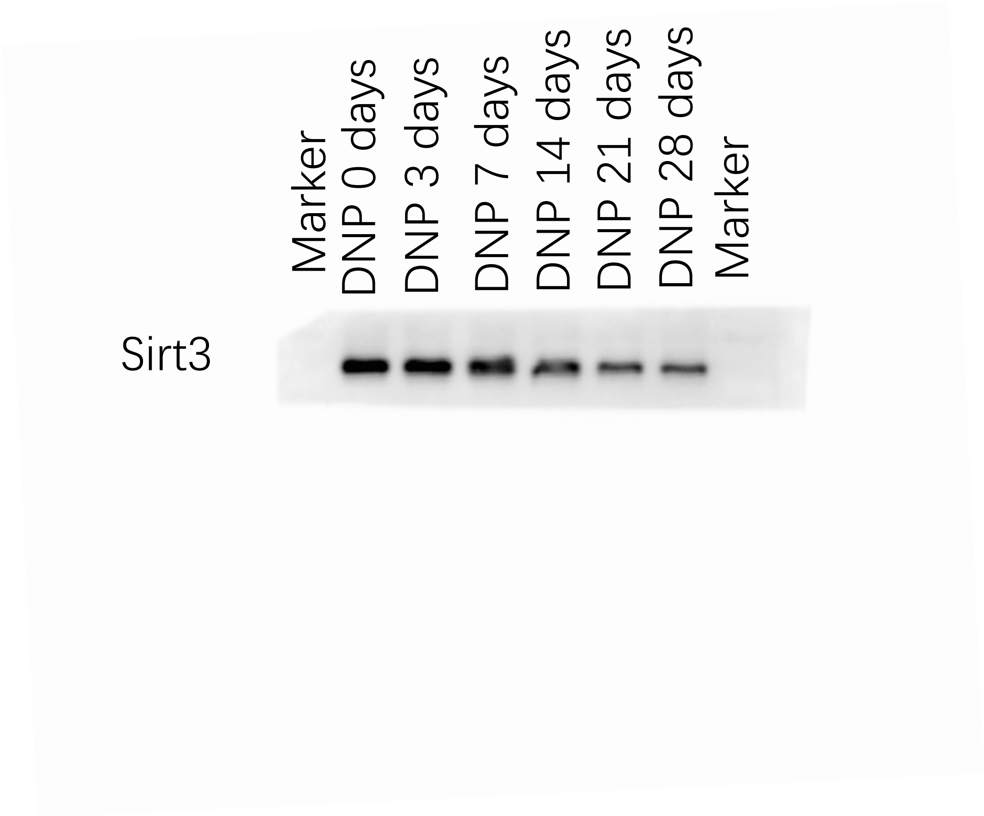
Marker
DNP 0 days
DNP 3 days
DNP 7 days
DNP 14 days
DNP 21 days
DNP 28 days
Marker
Sirt3

Fig.1e

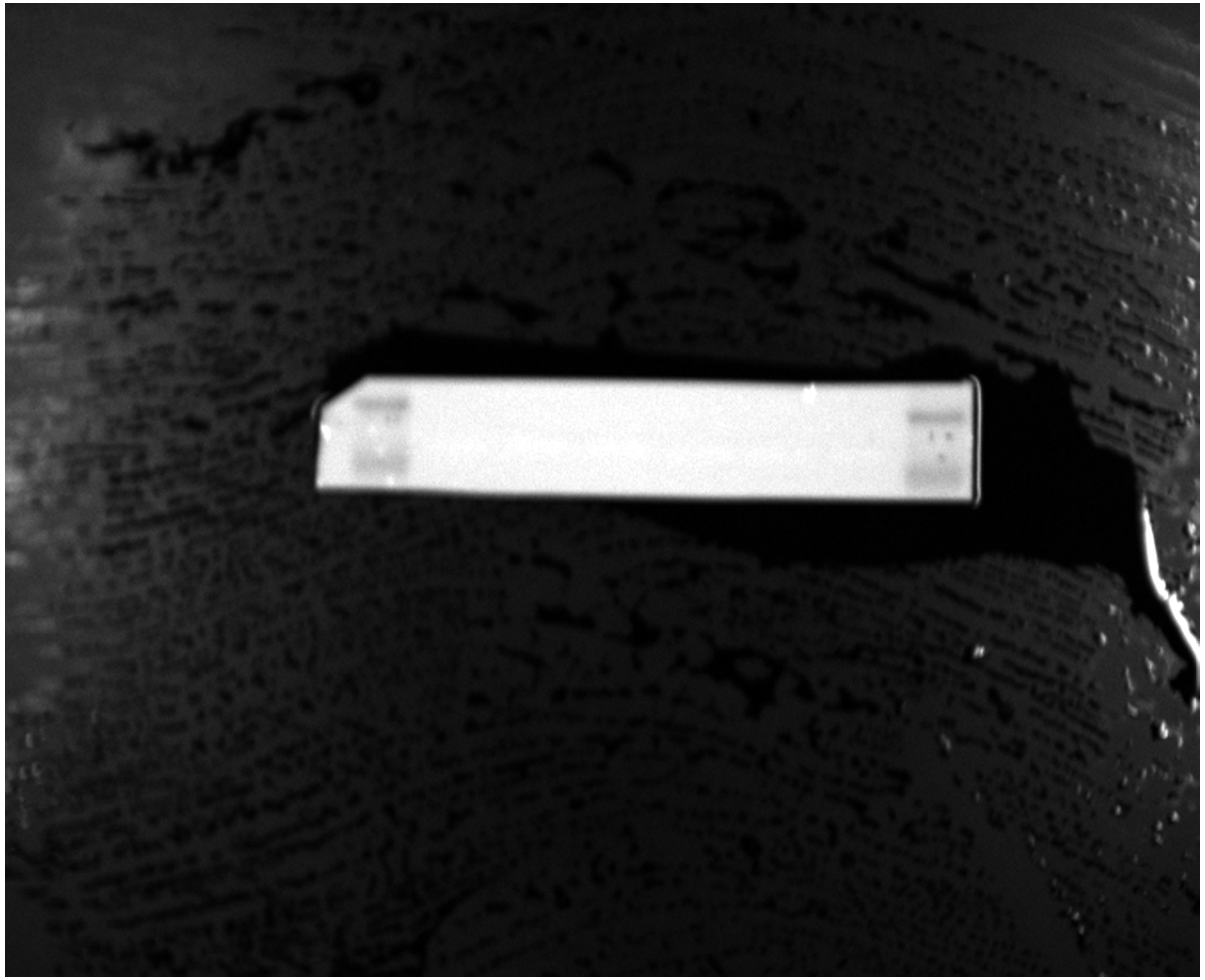

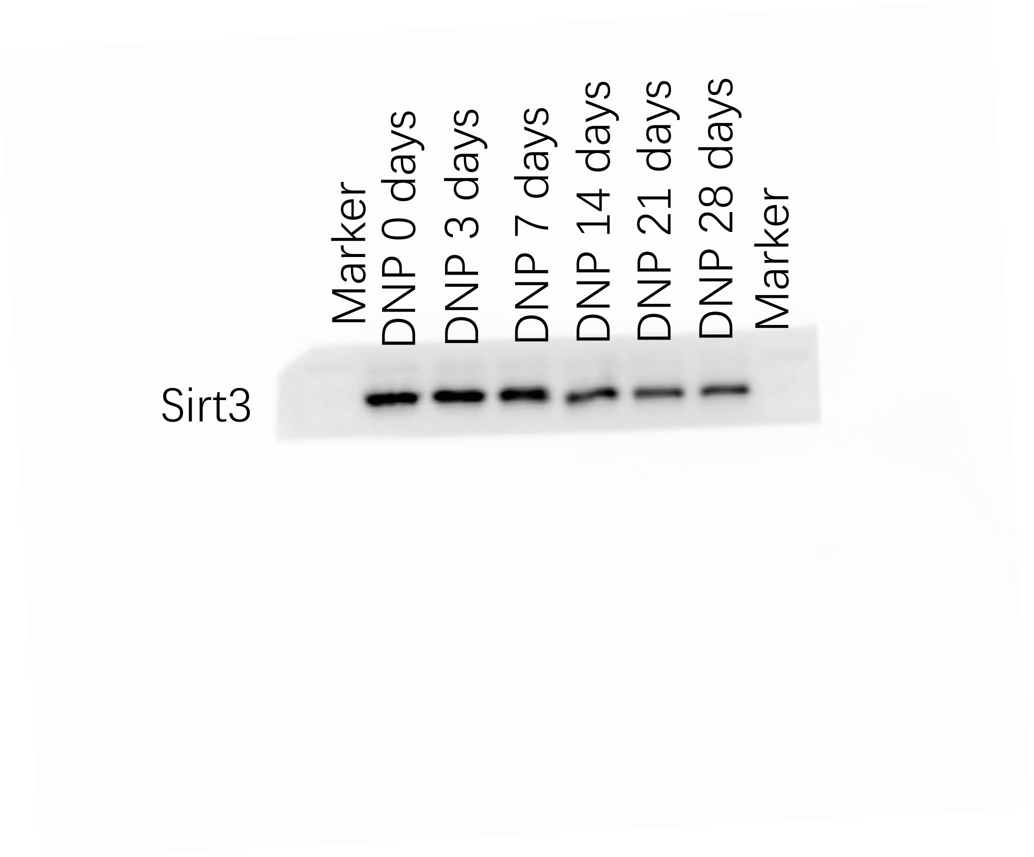
Marker
DNP 0 days
DNP 3 days
DNP 7 days
DNP 14 days
DNP 21 days
DNP 28 days
Marker
Sirt3

Fig.1e

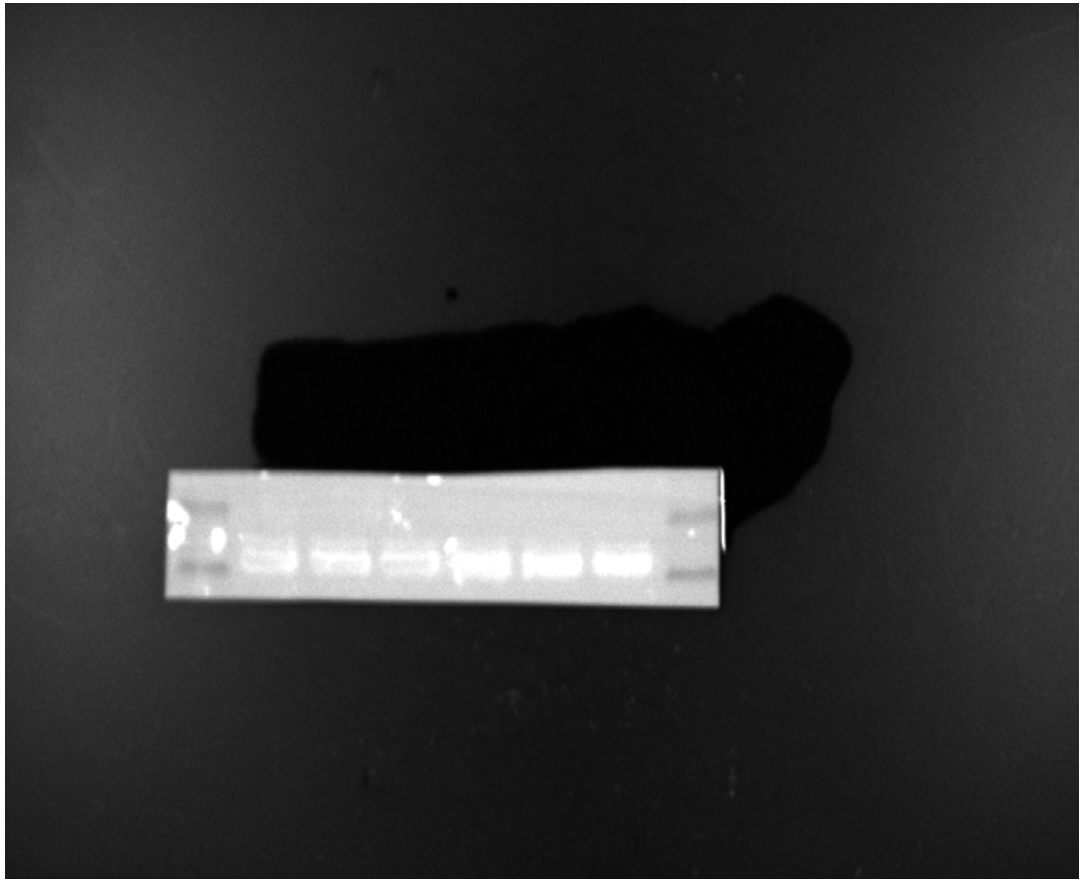

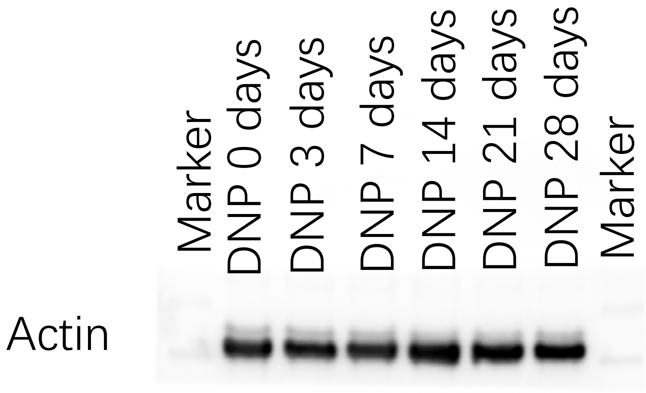
Marker
DNP 0 days
DNP 3 days
DNP 7 days
DNP 14 days
DNP 21 days
DNP 28 days
Marker
Actin

Fig.1e

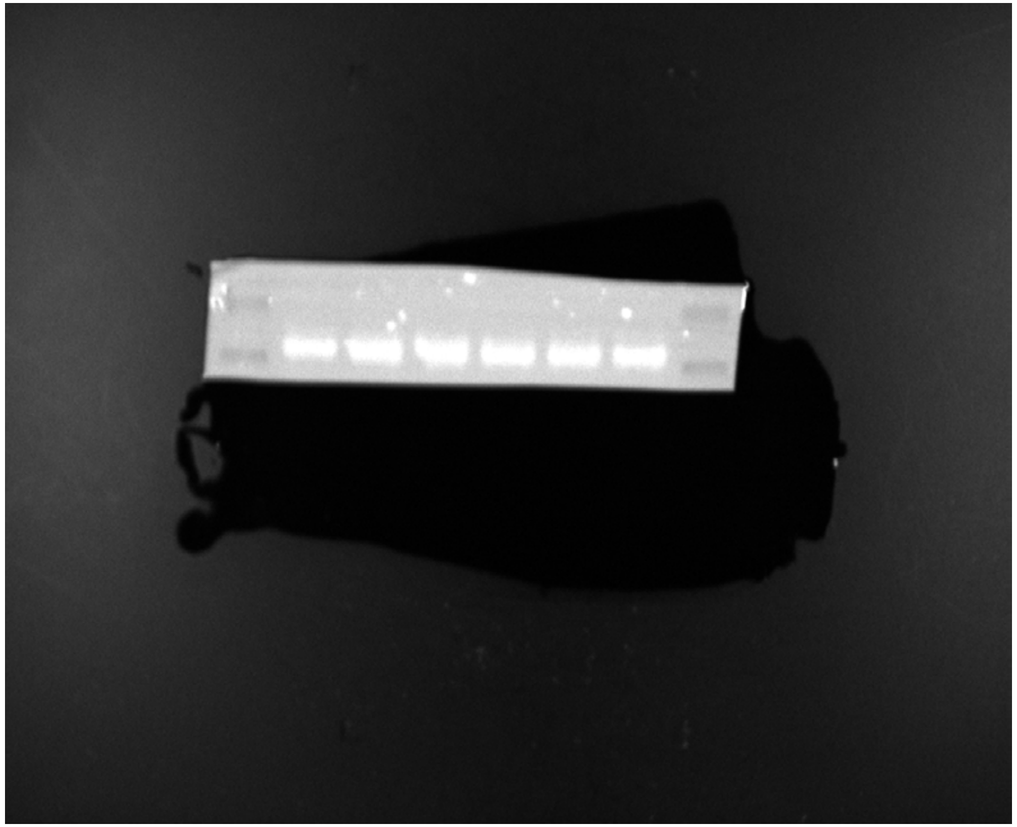

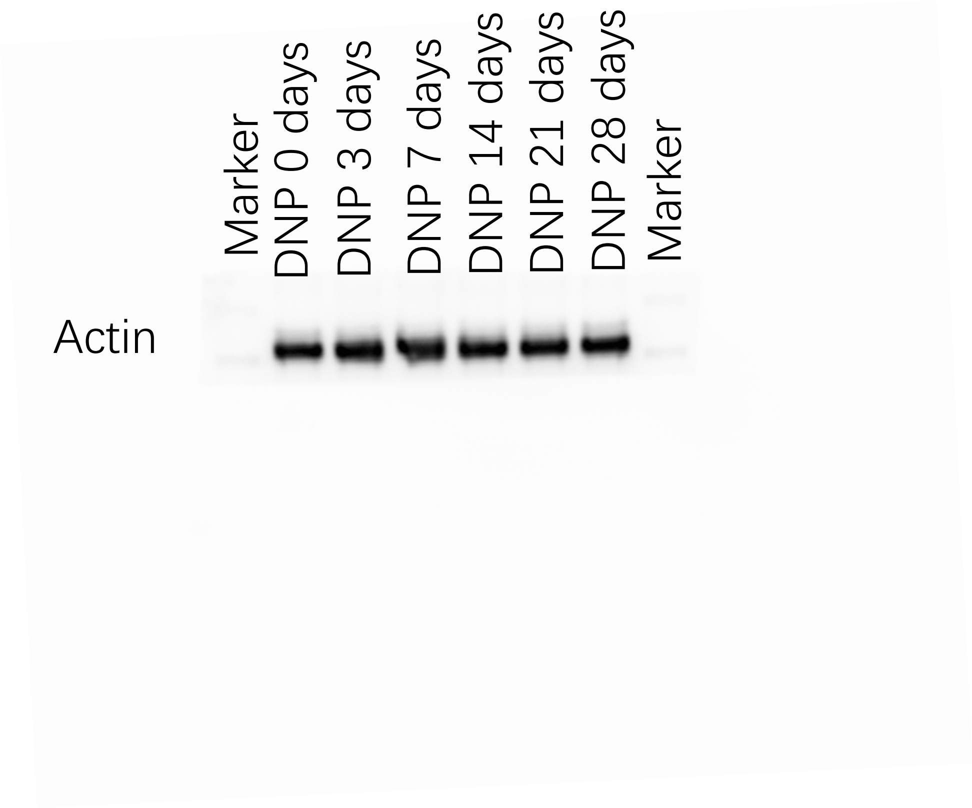
Marker
DNP 0 days
DNP 3 days
DNP 7 days
DNP 14 days
DNP 21 days
DNP 28 days
Marker
Actin

Fig.1e

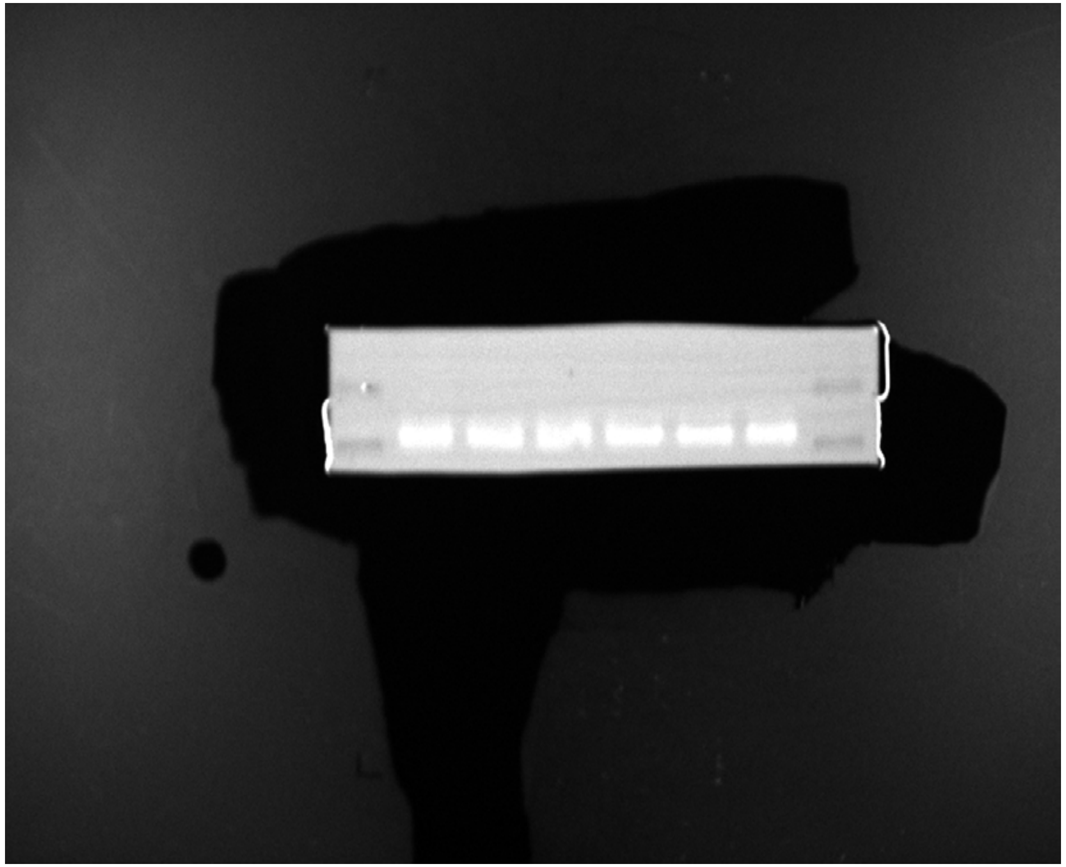

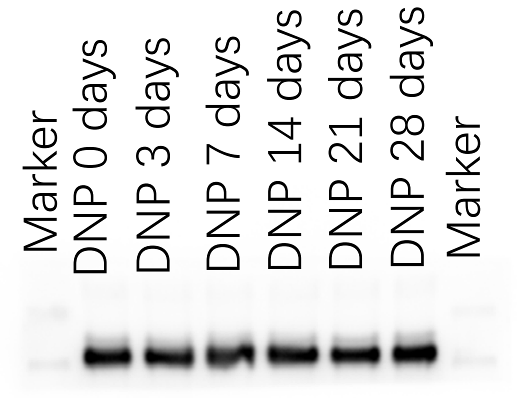
Actin
Marker
DNP 0 days
DNP 3 days
DNP 7 days
DNP 14 days
DNP 21 days
DNP 28 days
Marker

Fig.2b

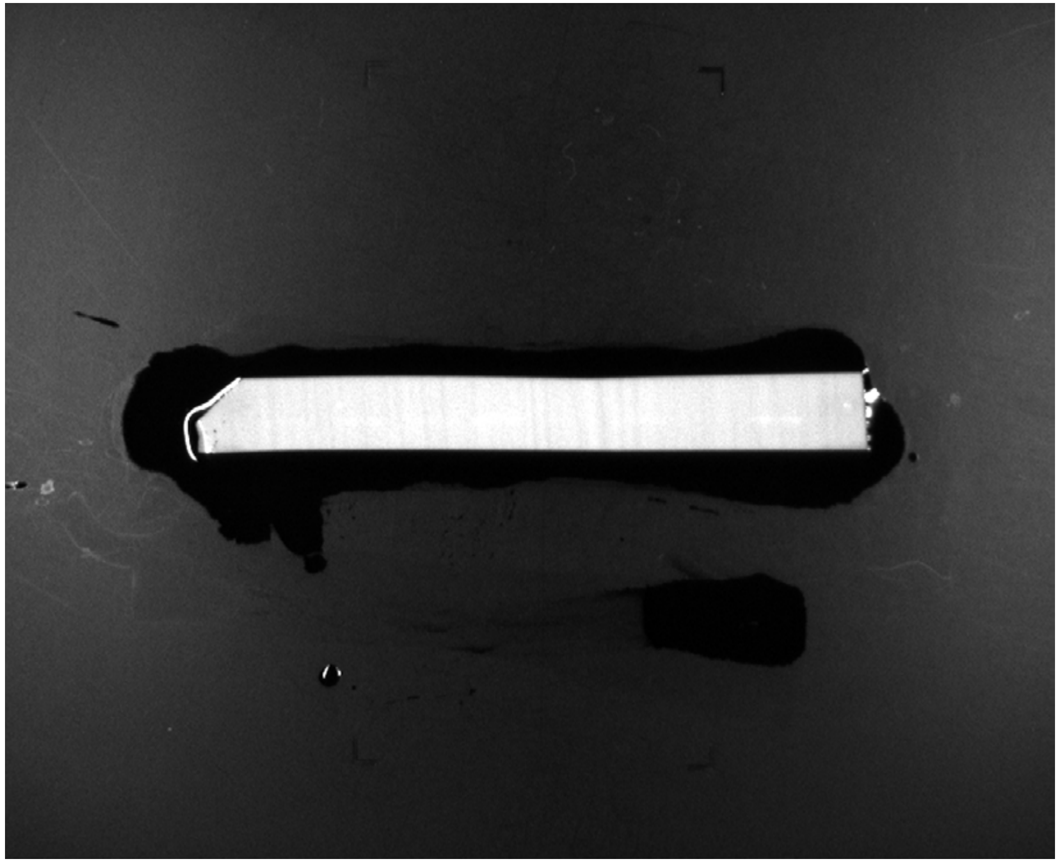

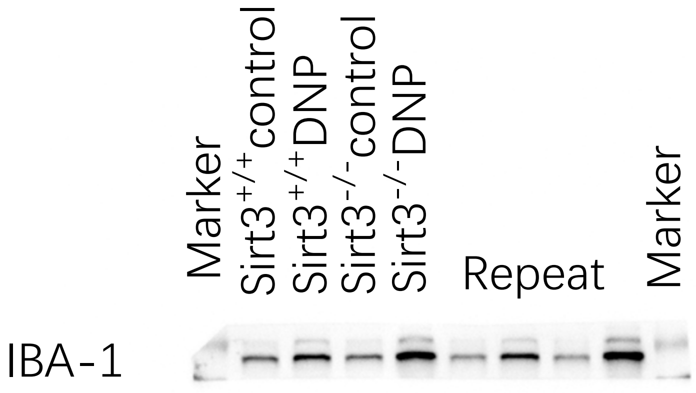
Marker
Sirt3+/+control
Sirt3+/+DNP
Sirt3-/-control
Sirt3-/-DNP
Repeat
Marker
IBA-1

Fig.2b

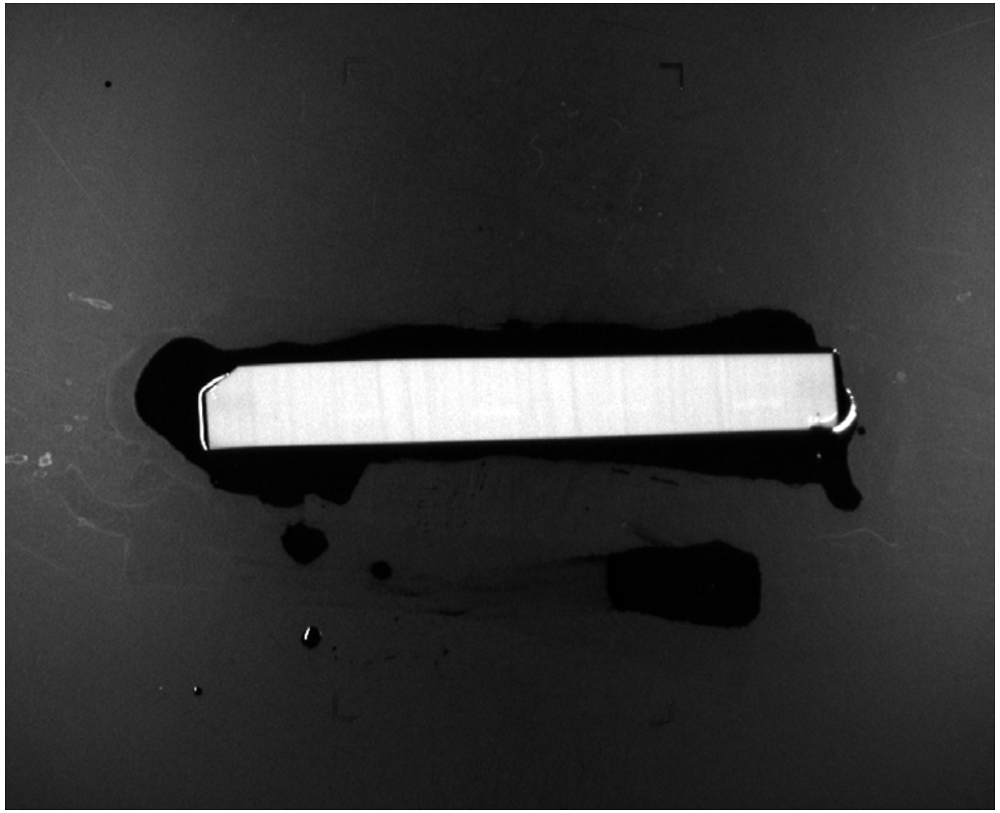

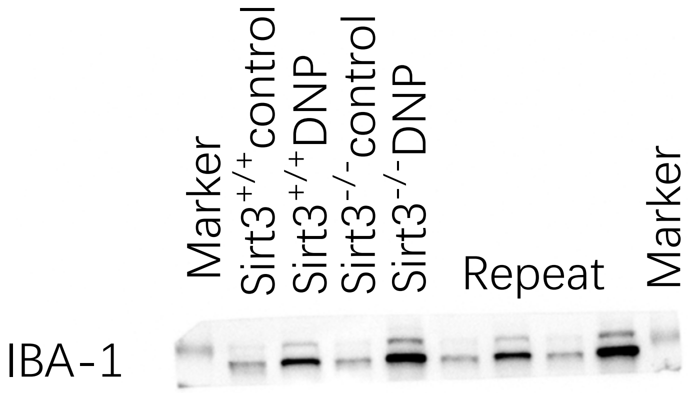
Marker
Sirt3+/+control
Sirt3+/+DNP
Sirt3-/-control
Sirt3-/-DNP
Repeat
Marker
IBA-1

Fig.2b

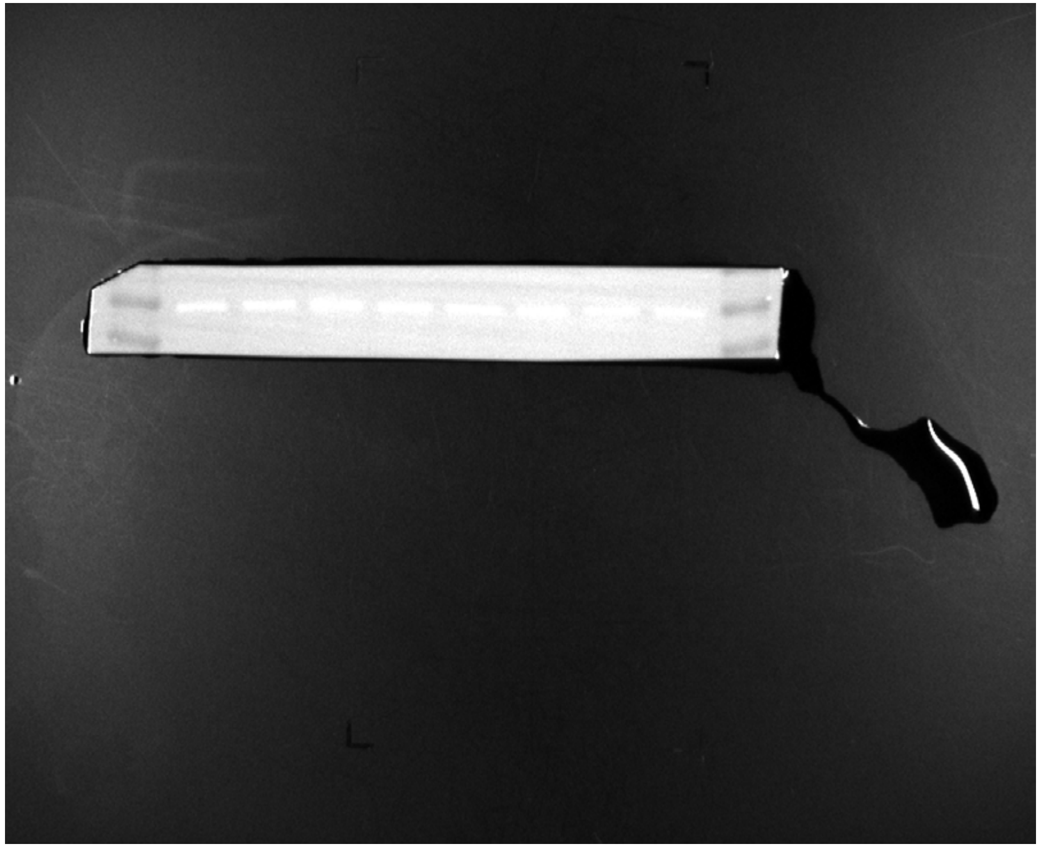

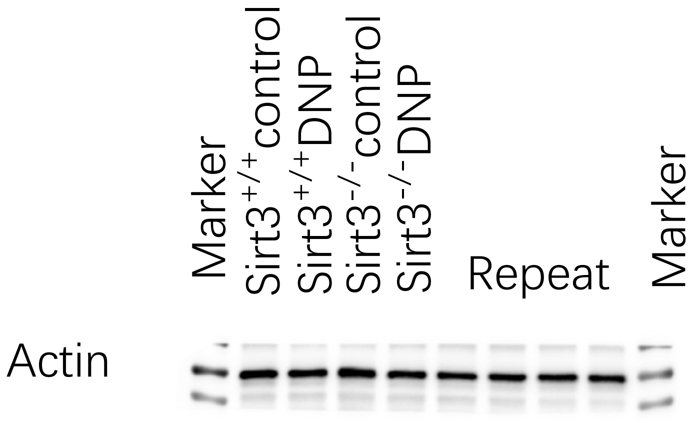

Marker
Sirt3+/+control
Sirt3+/+DNP
Sirt3-/-control
Sirt3-/-DNP
Repeat
Marker
Actin

Fig.2b

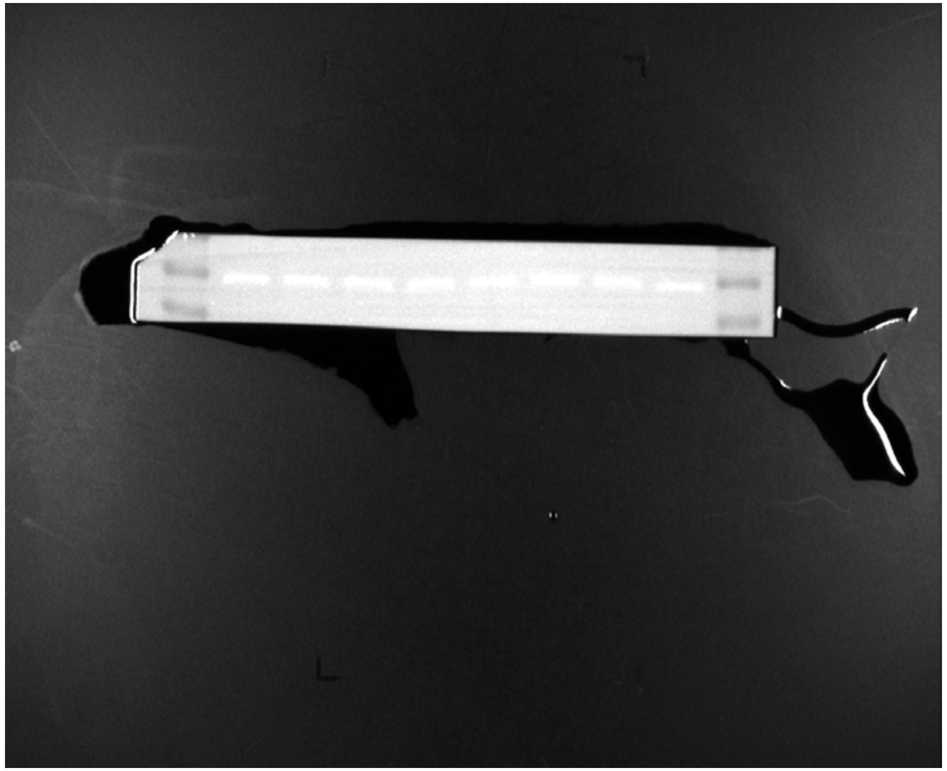

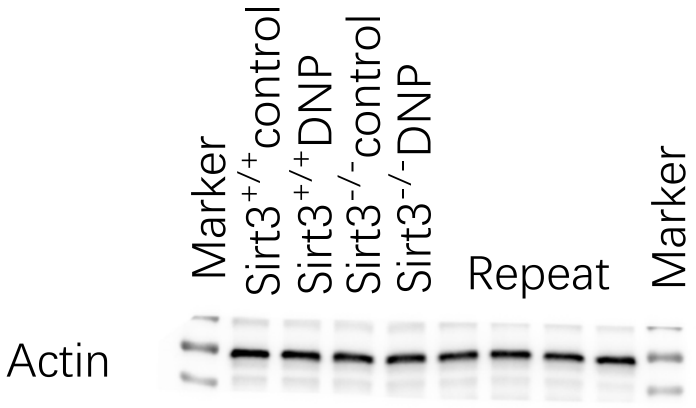

Marker
Sirt3$^{+/+}$control
Sirt3$^{+/+}$DNP
Sirt3$^{-/-}$control
Sirt3$^{-/-}$DNP
Repeat
Marker
Actin

Fig.2c

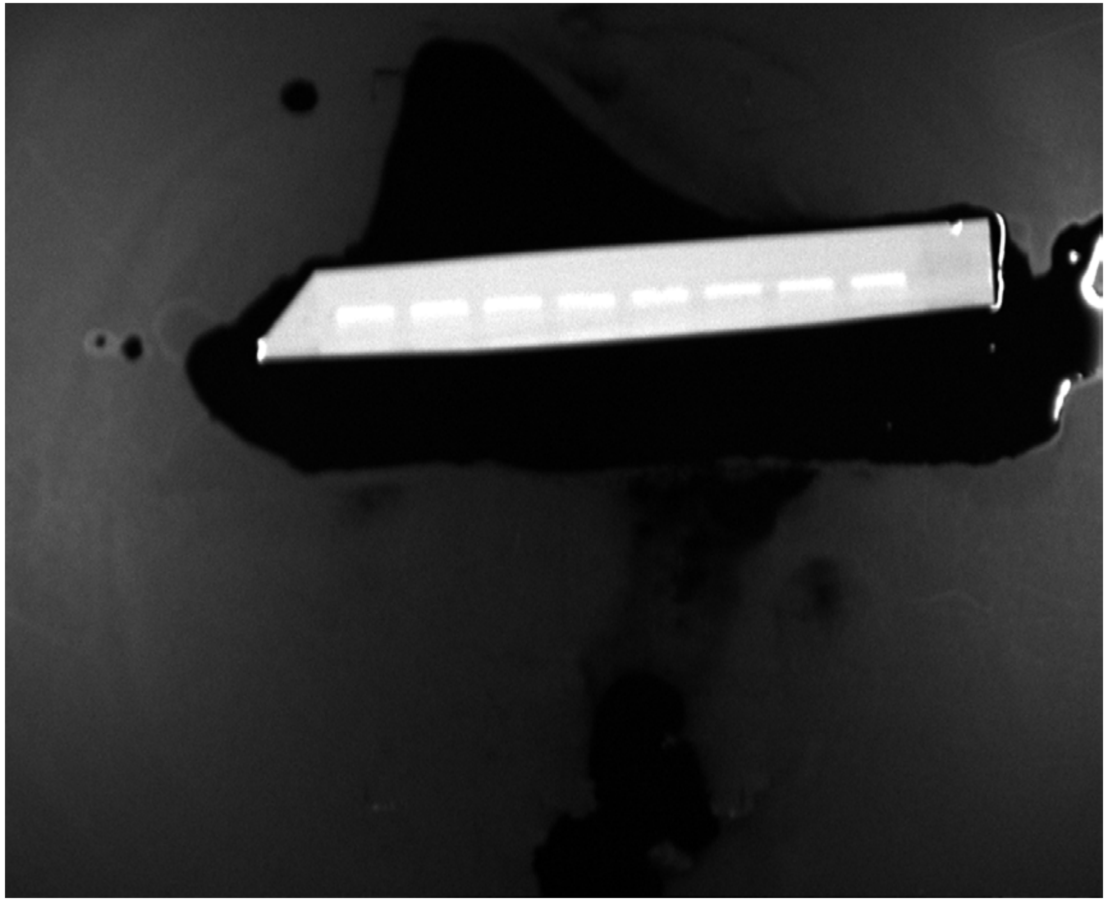

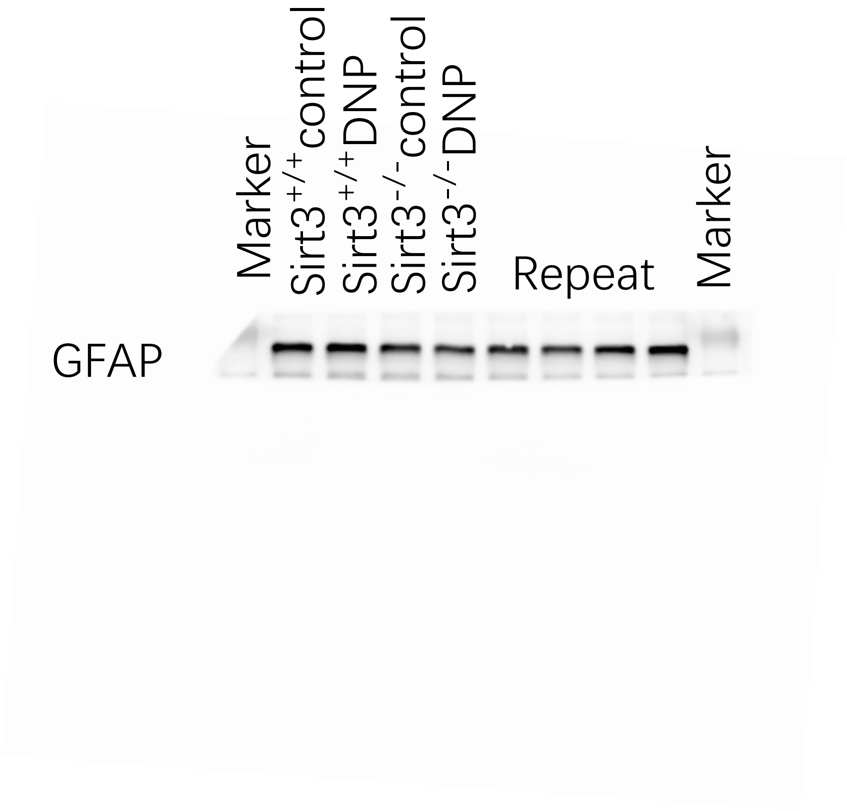
Marker
Sirt3+/+control
Sirt3+/+DNP
Sirt3-/-control
Sirt3-/-DNP
Repeat
Marker
GFAP

Fig.2c

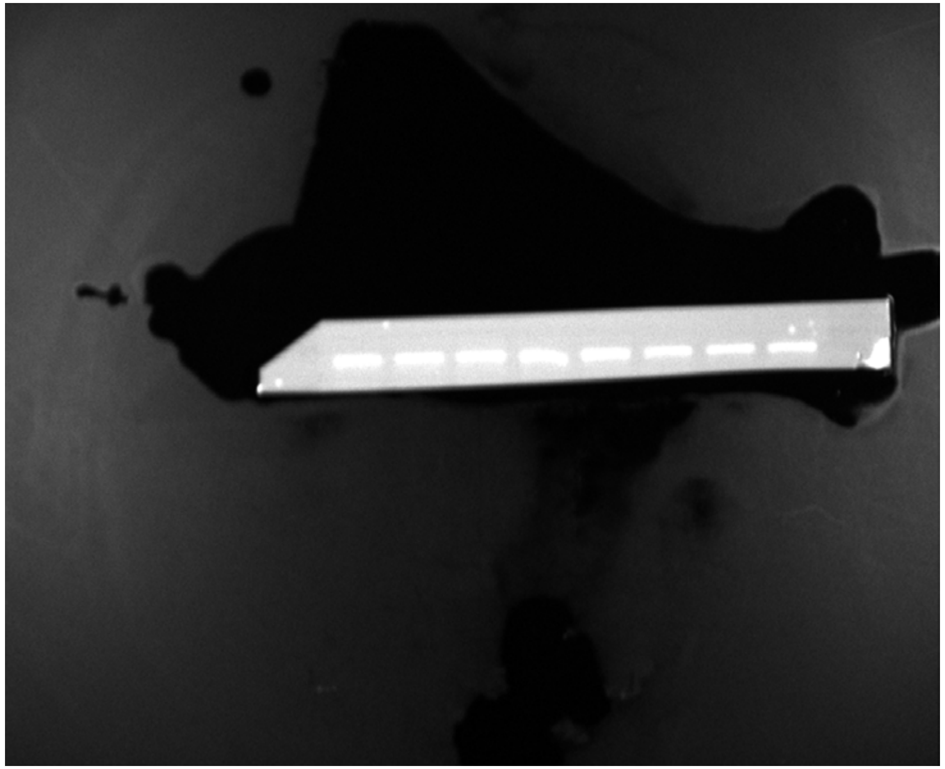

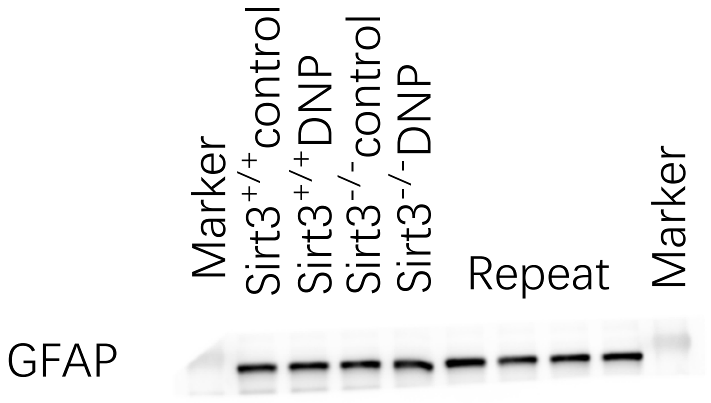

Marker
Sirt3+/+control
Sirt3+/+DNP
Sirt3-/-control
Sirt3-/-DNP
Repeat
Marker
GFAP

Fig.2f

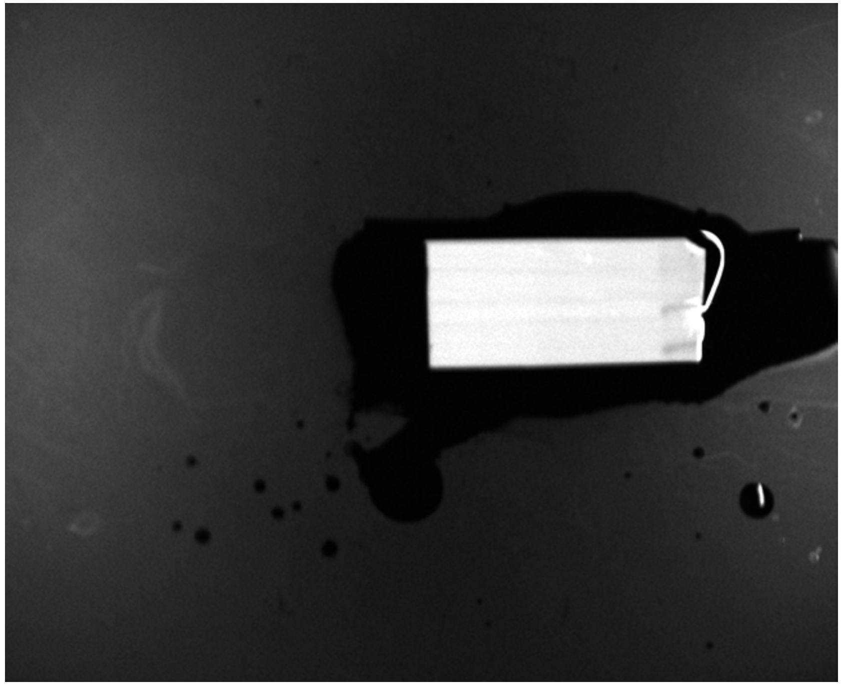

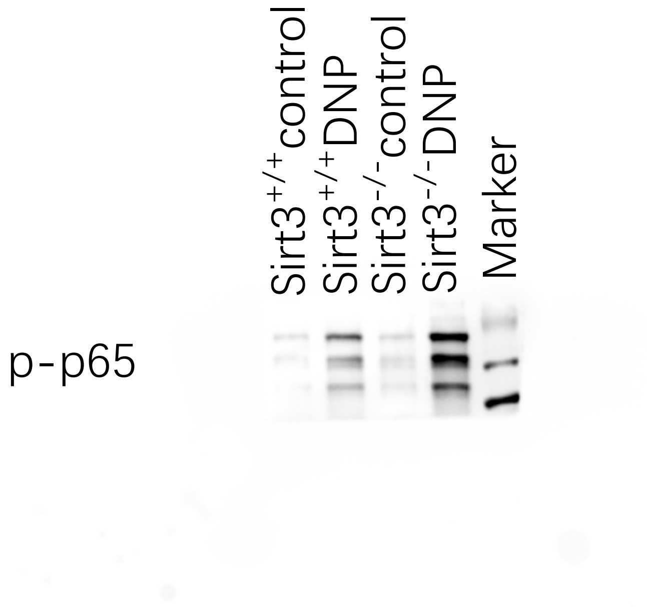
Sirt3+/+control
Sirt3+/+DNP
Sirt3-/-control
Sirt3-/-DNP
Marker
p-p65

Fig.2f

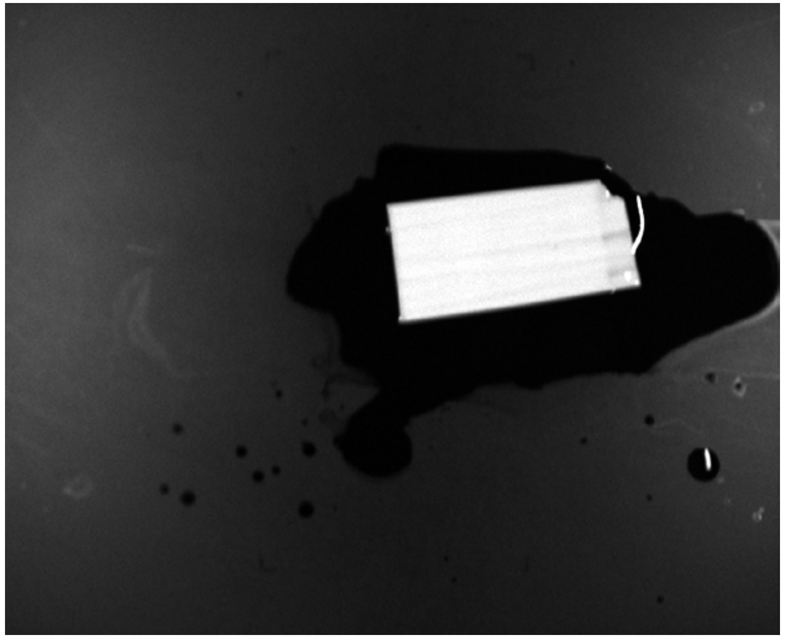

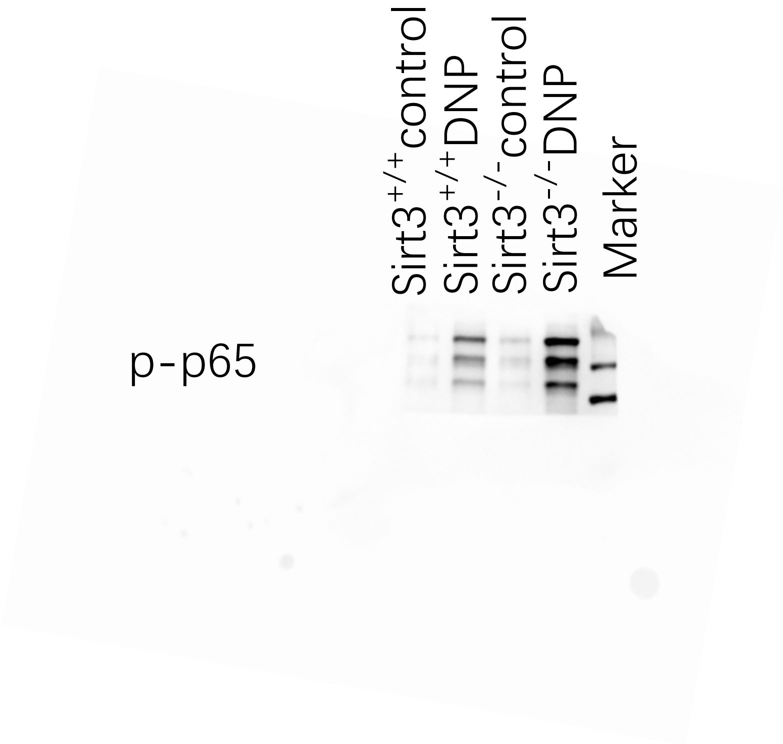
Sirt3+/+control
Sirt3+/+DNP
Sirt3-/-control
Sirt3-/-DNP
Marker
p-p65

Fig.2f

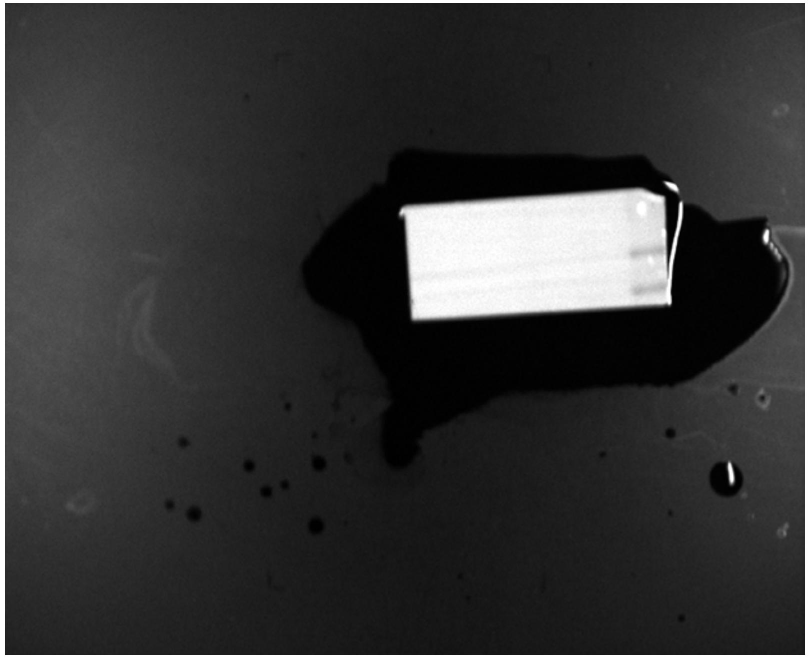

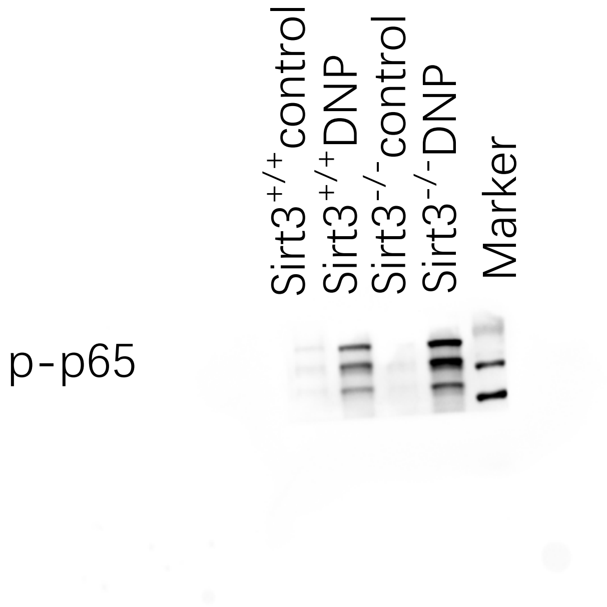
Sirt3$^{+/+}$control
Sirt3$^{+/+}$DNP
Sirt3$^{-/-}$control
Sirt3$^{-/-}$DNP
Marker
p-p65

Fig.2f

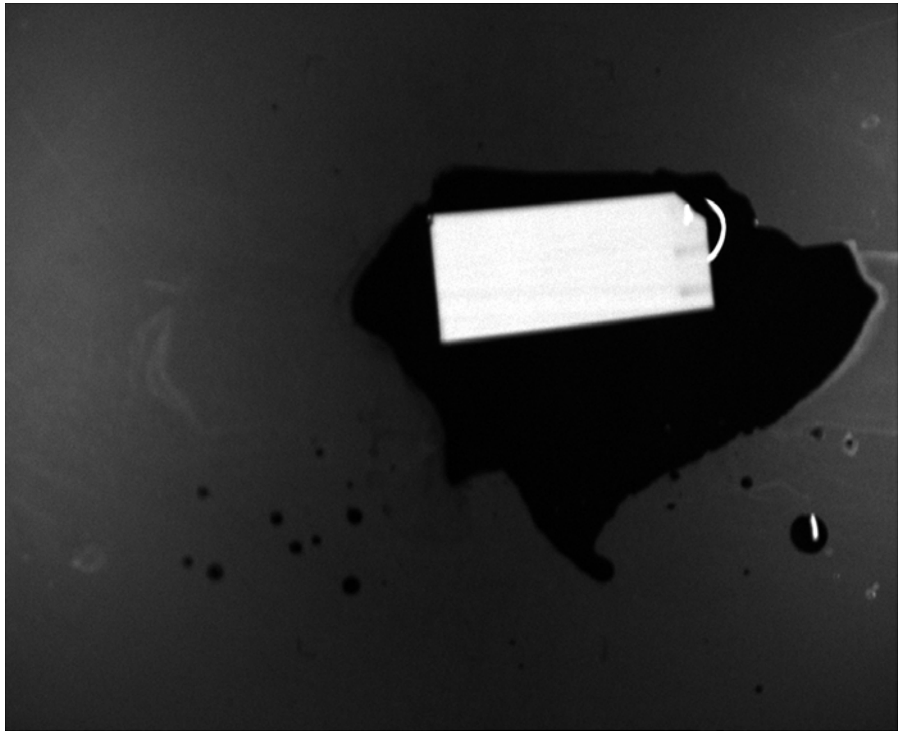

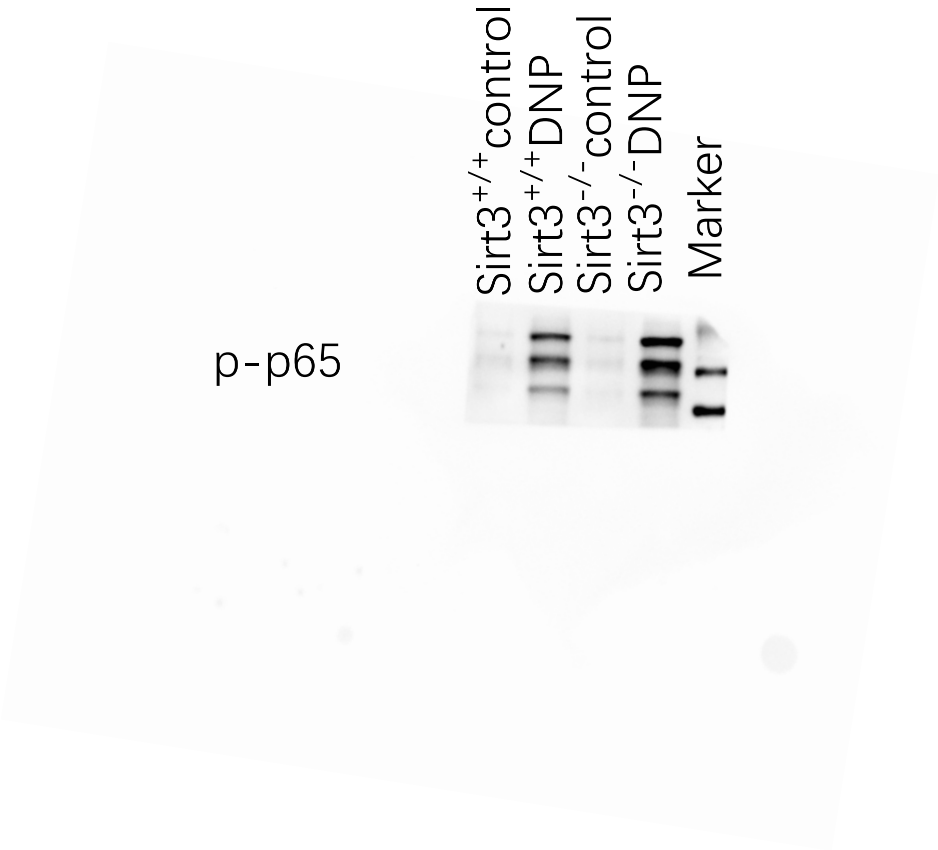
Sirt3+/+control
Sirt3+/+DNP
Sirt3-/-control
Sirt3-/-DNP
Marker
p-p65

Fig.2f

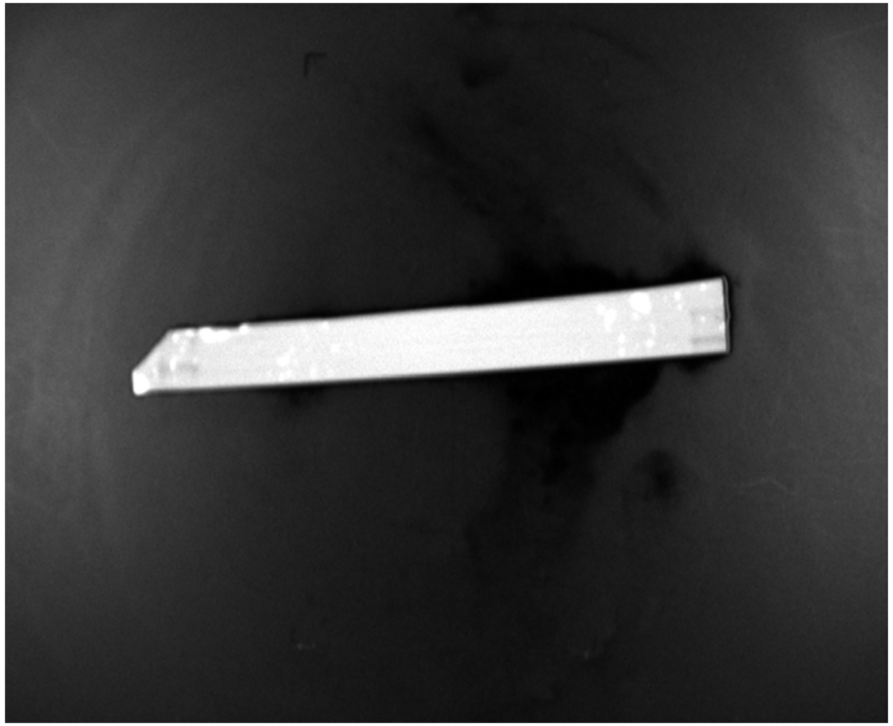

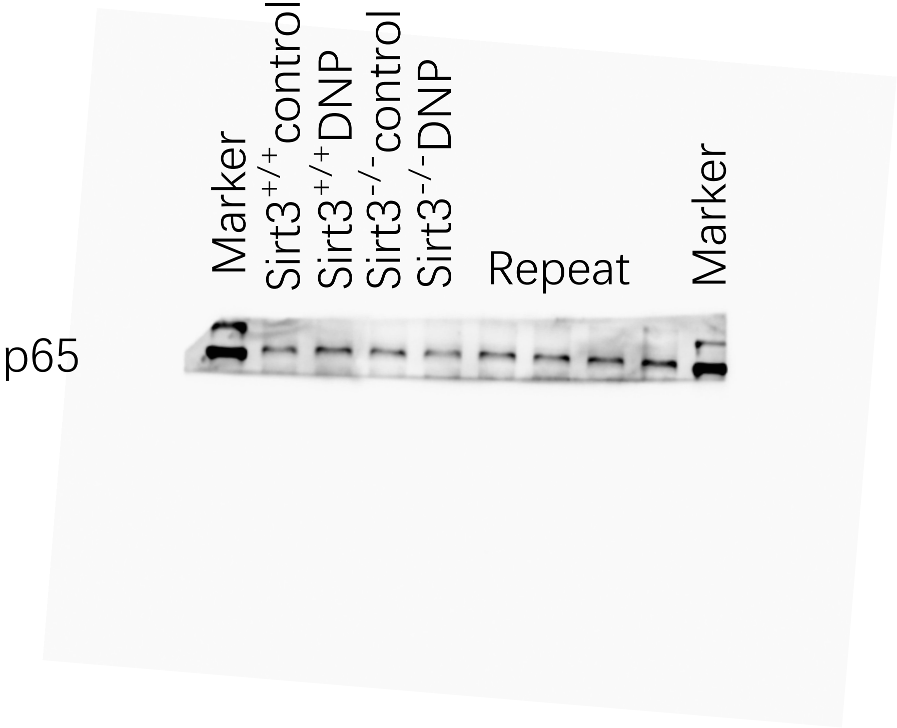
Marker
Sirt3+/+control
Sirt3+/+DNP
Sirt3-/-control
Sirt3-/-DNP
Repeat
Marker
p65

Fig.2f

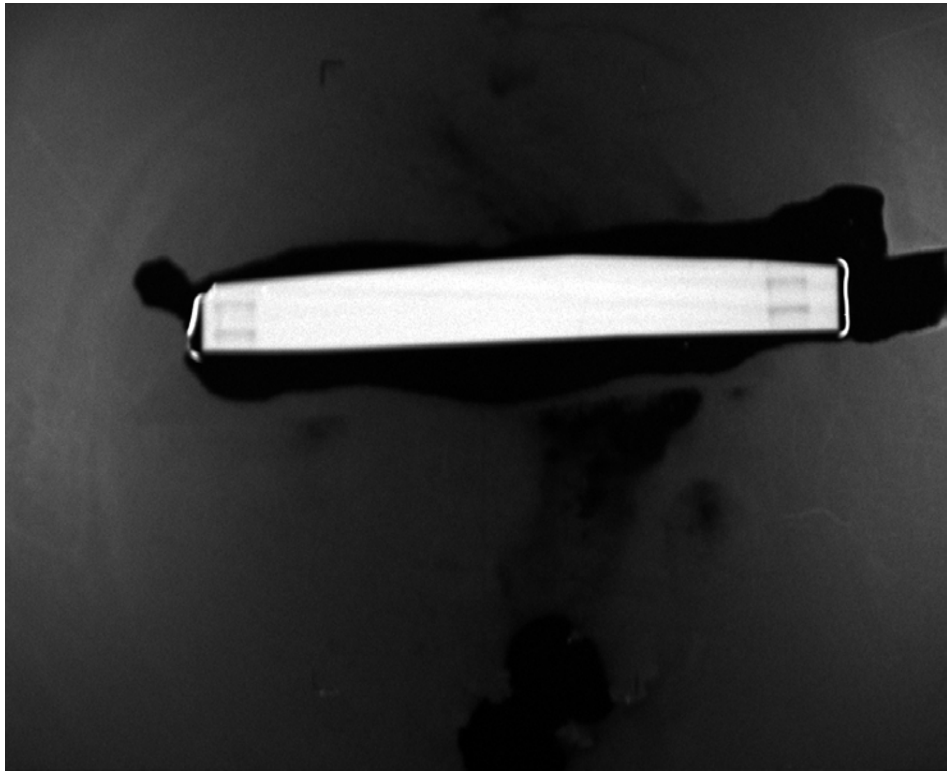

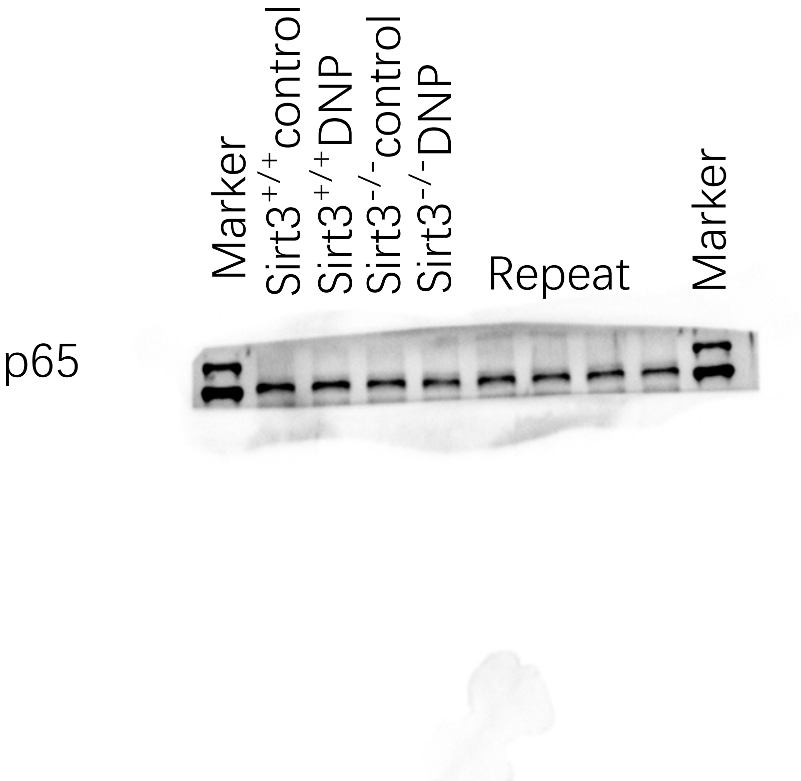
Marker
Sirt3+/+control
Sirt3+/+DNP
Sirt3-/-control
Sirt3-/-DNP
Repeat
Marker
p65

Fig.2g

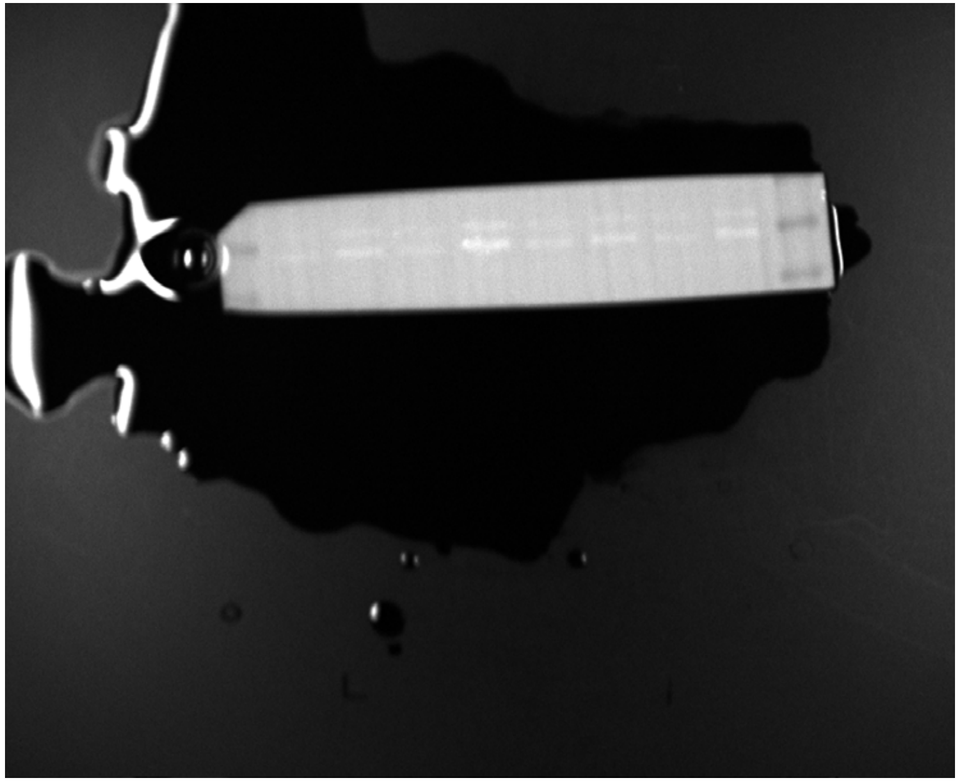

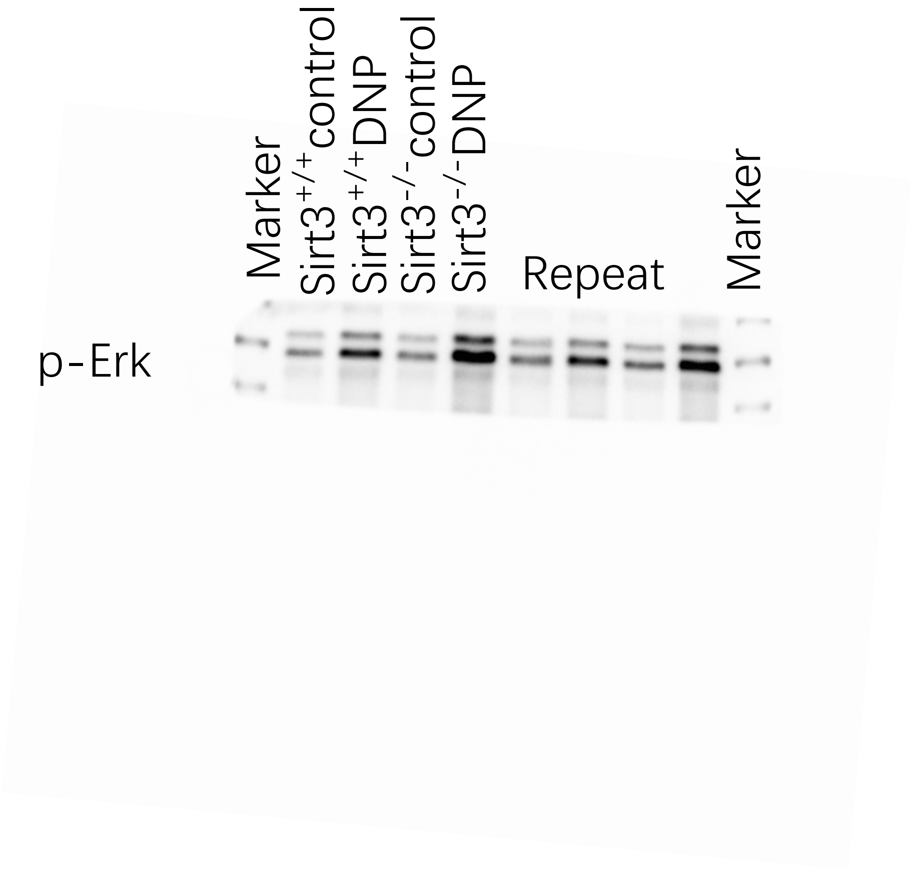

Marker
Sirt3+/+control
Sirt3+/+DNP
Sirt3-/-control
Sirt3-/-DNP
Repeat
Marker
p-Erk

Fig.2g

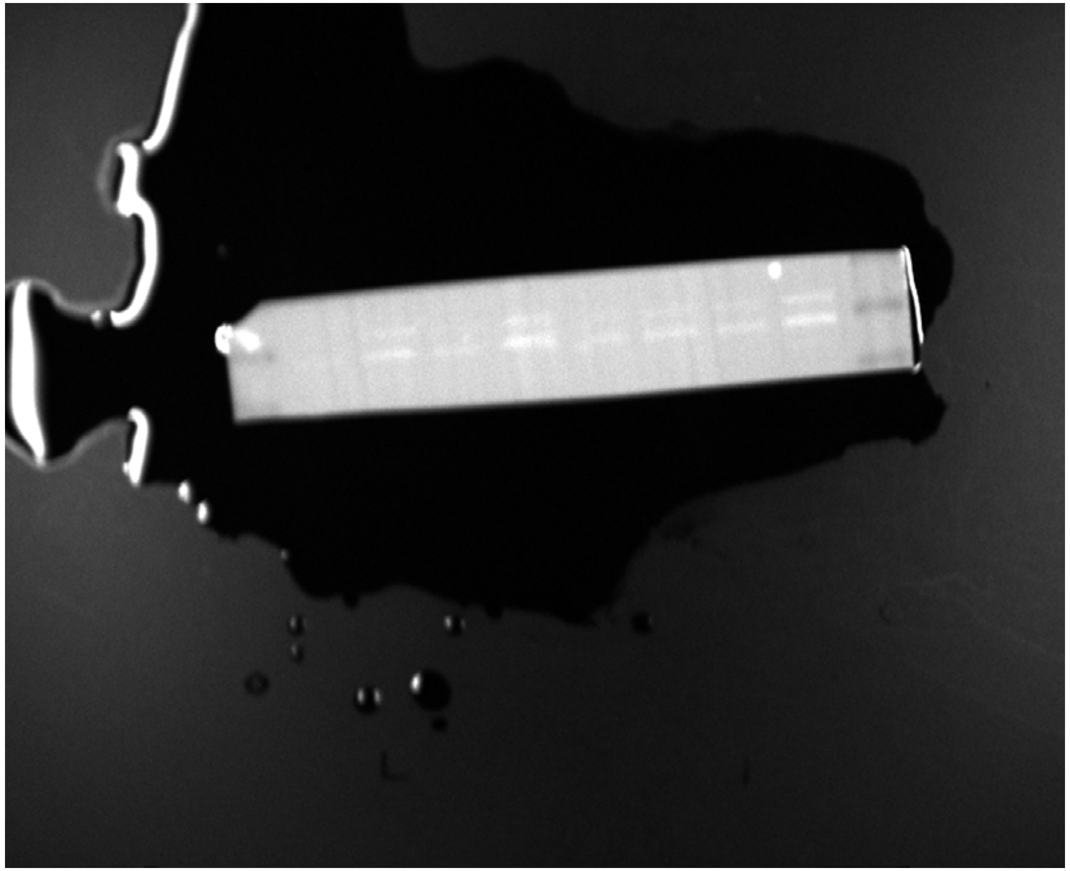

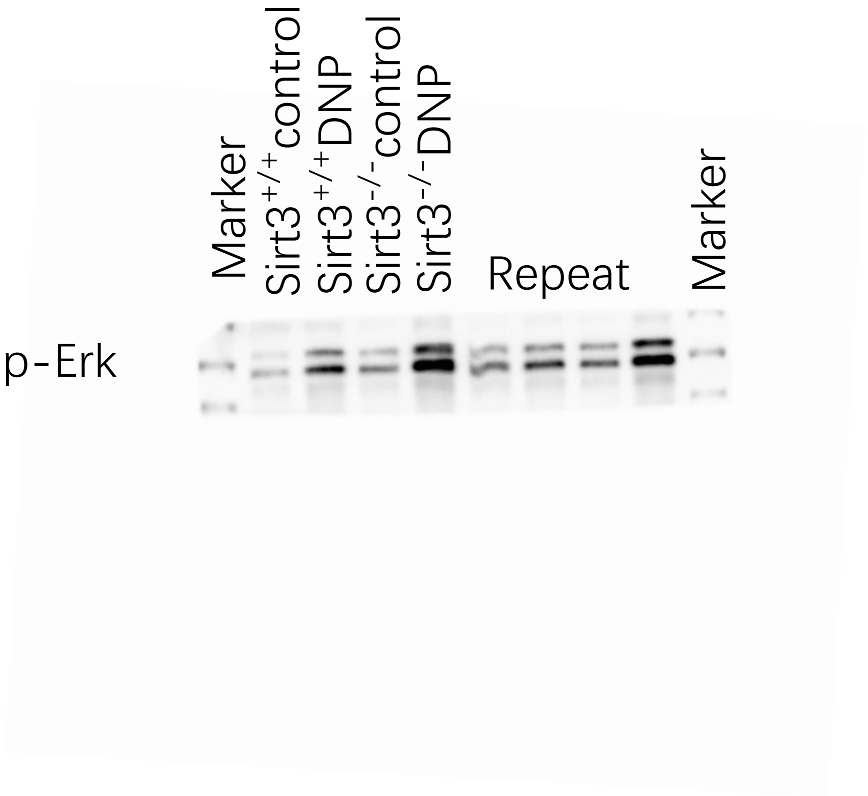
Marker
Sirt3+/+control
Sirt3+/+DNP
Sirt3-/-control
Sirt3-/-DNP
Repeat
Marker
p-Erk

Fig.2g

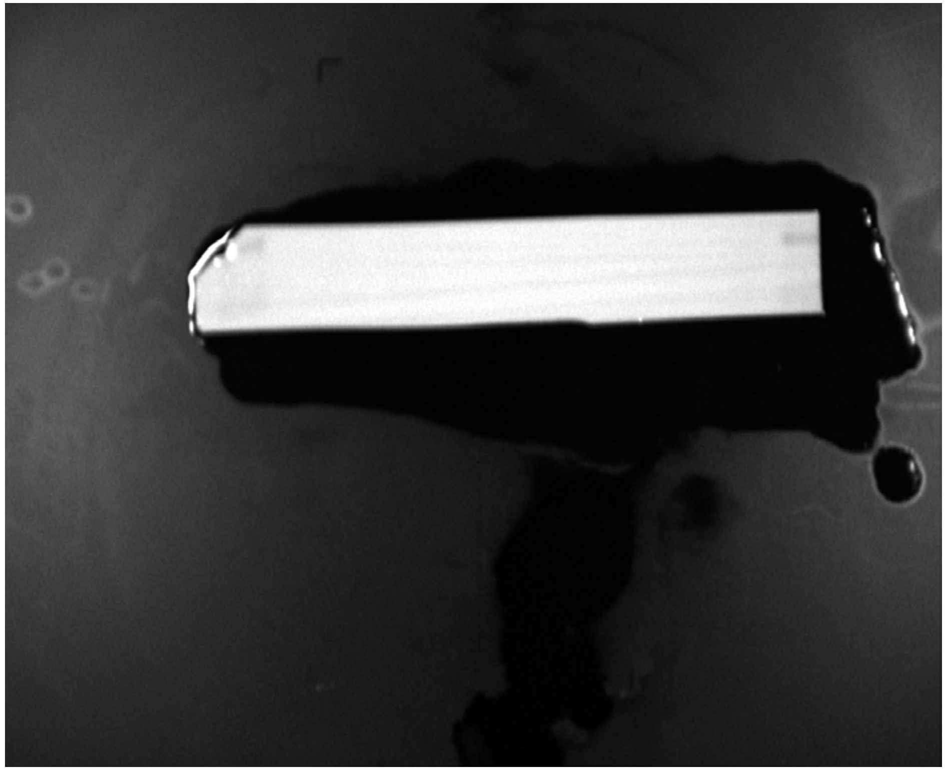

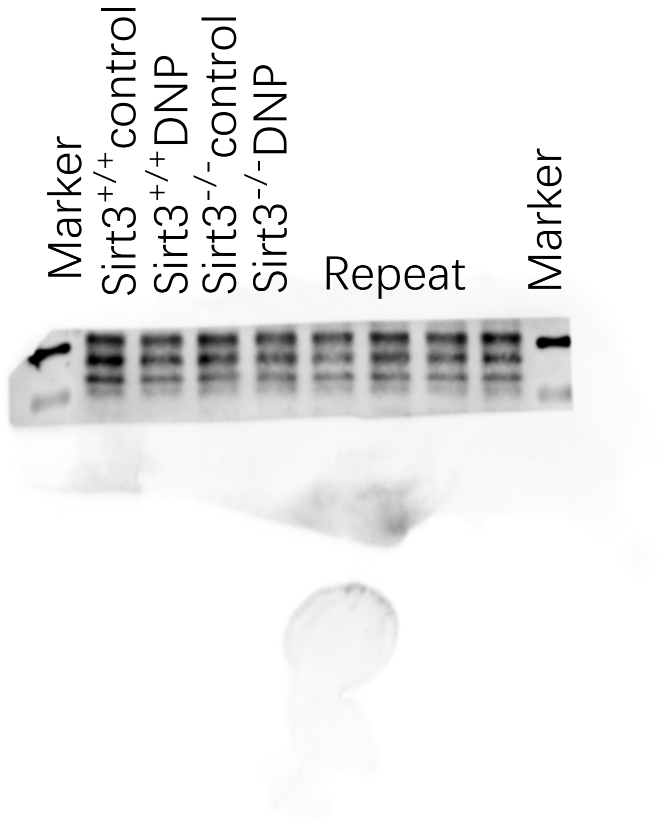
Marker
Sirt3+/+control
Sirt3+/+DNP
Sirt3-/-control
Sirt3-/-DNP
Repeat
Marker
Erk

Fig.2g

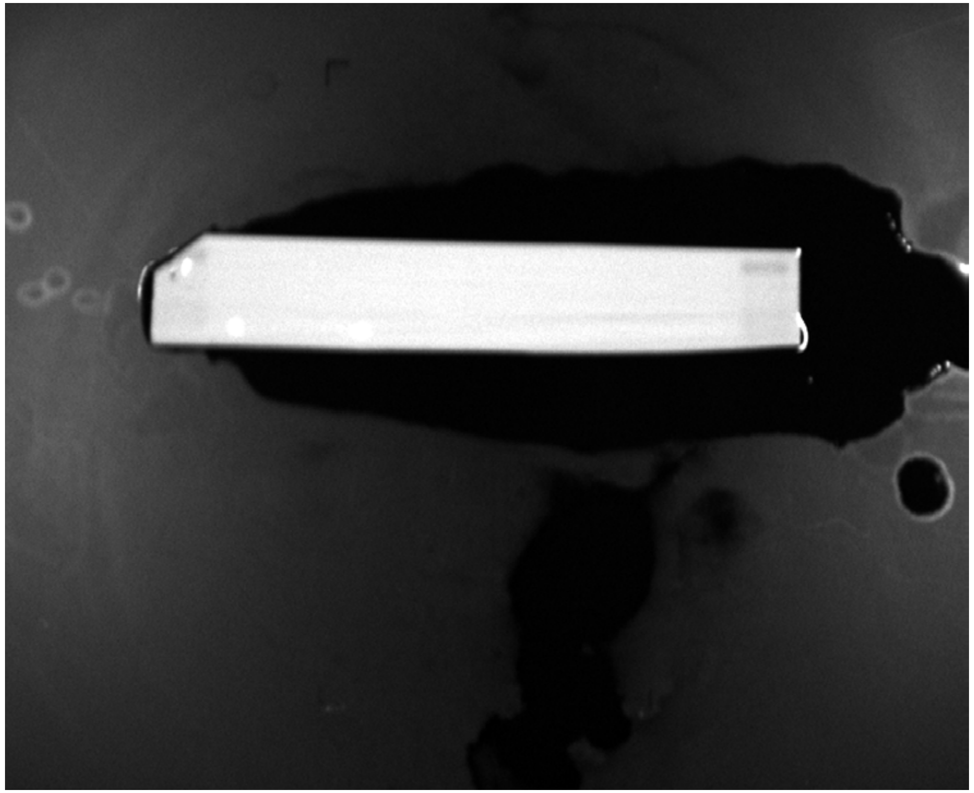

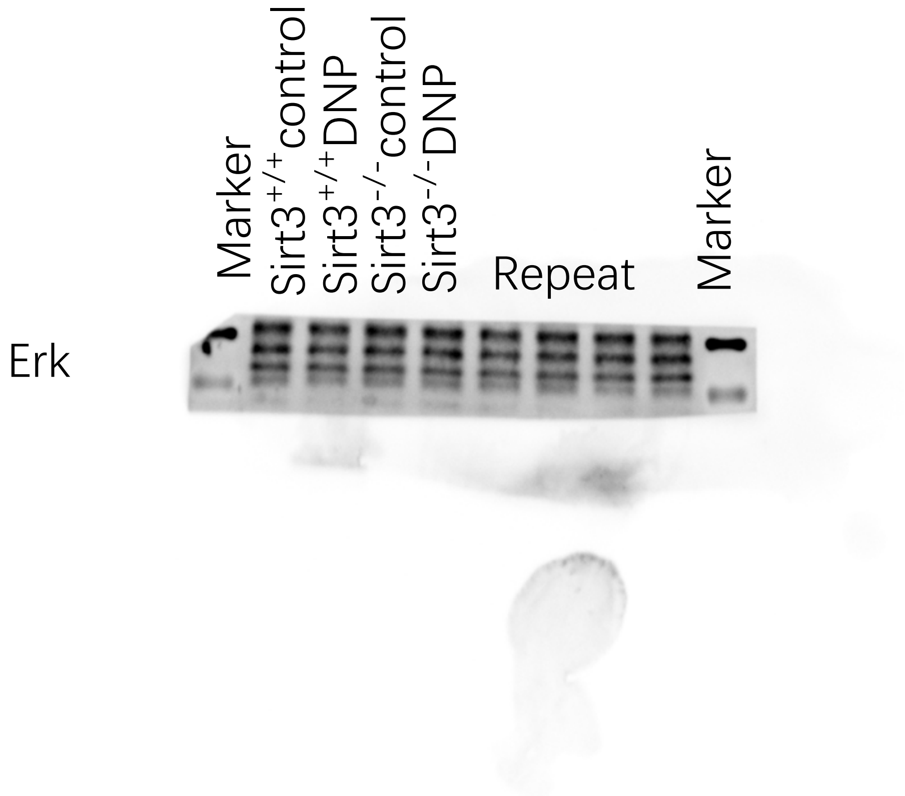
Marker
Sirt3+/+control
Sirt3+/+DNP
Sirt3-/-control
Sirt3-/-DNP
Repeat
Marker
Erk

Fig.2f

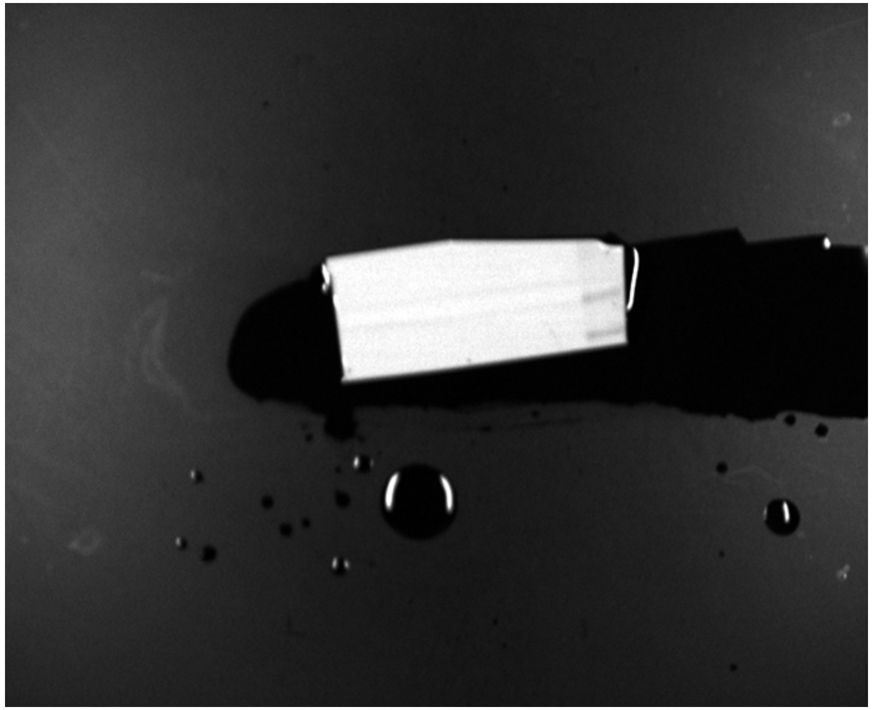

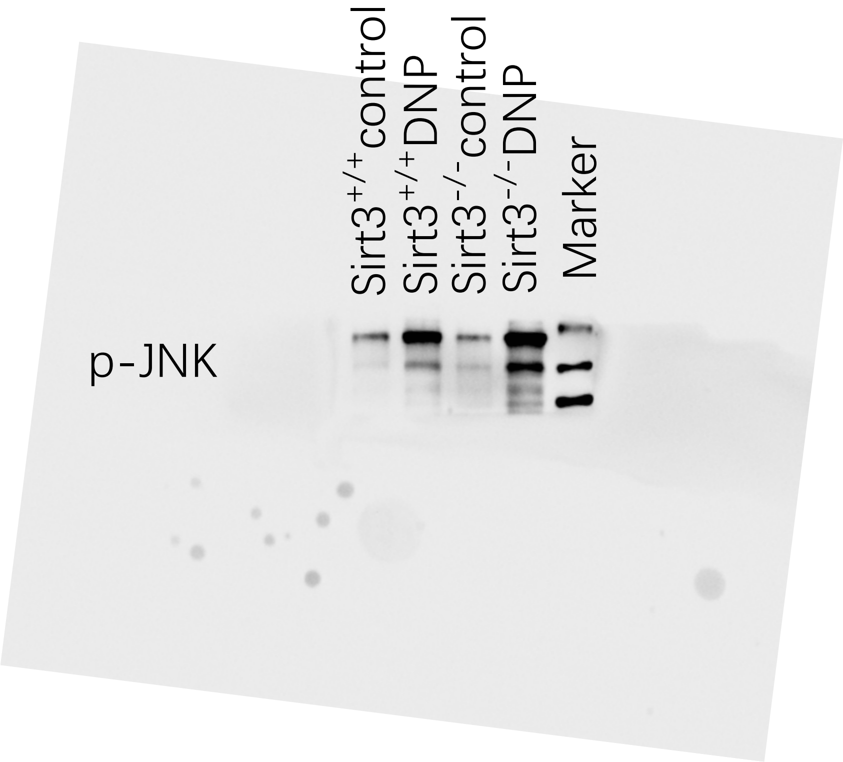

Sirt3+/+control
Sirt3+/+DNP
Sirt3-/-control
Sirt3-/-DNP
Marker
p-JNK

Fig.2f

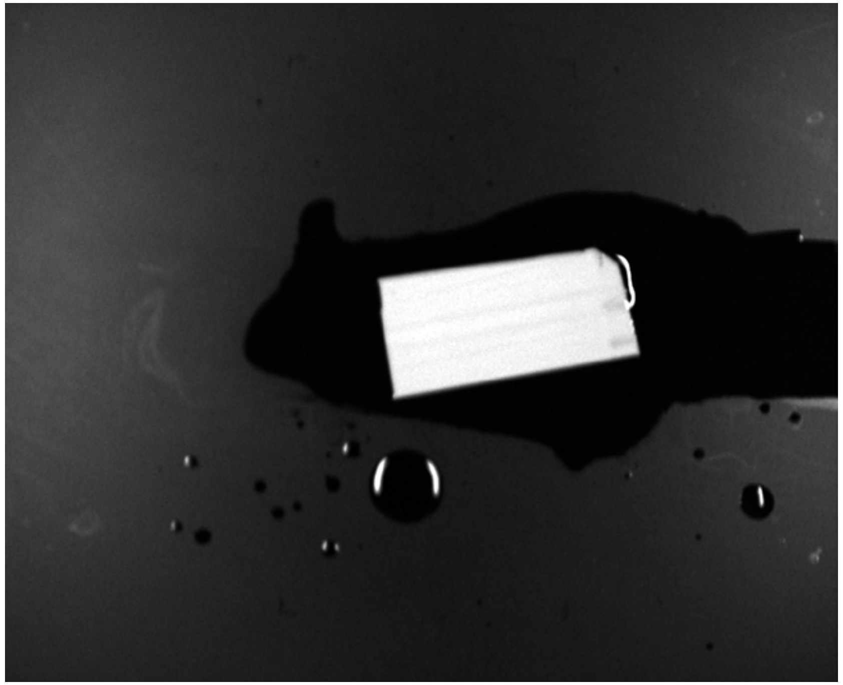

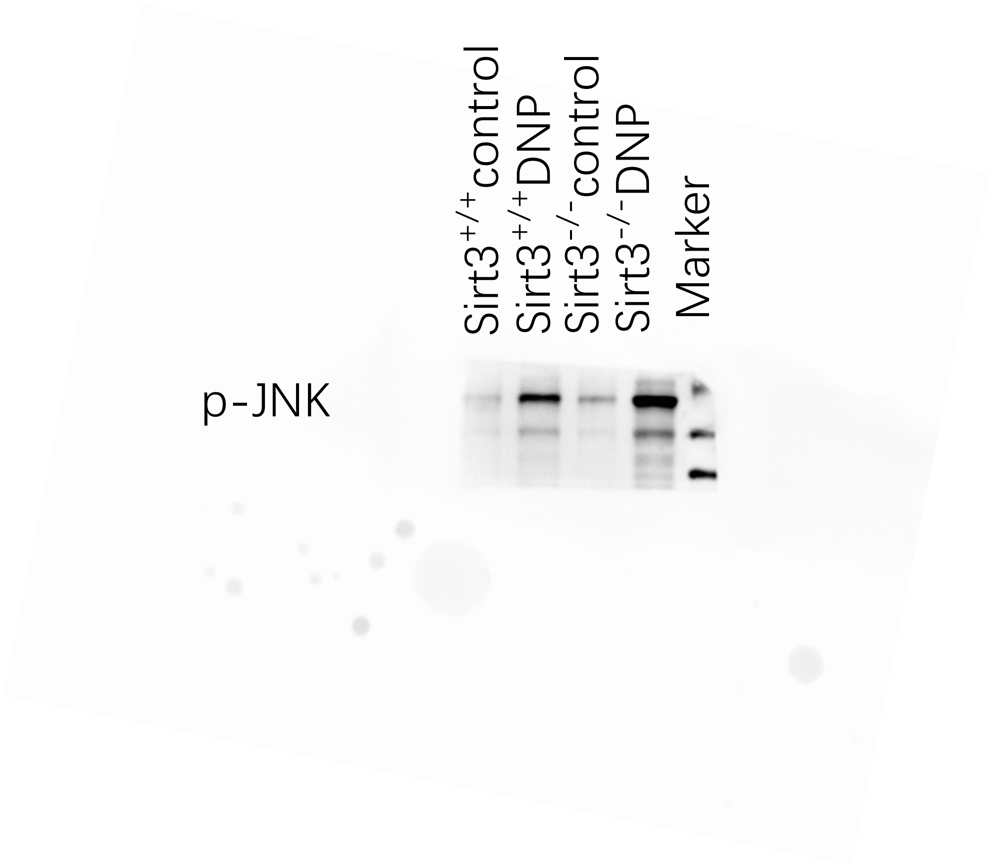

Sirt3$^{+/+}$control
Sirt3$^{+/+}$DNP
Sirt3$^{-/-}$control
Sirt3$^{-/-}$DNP
Marker
p-JNK

Fig.2f

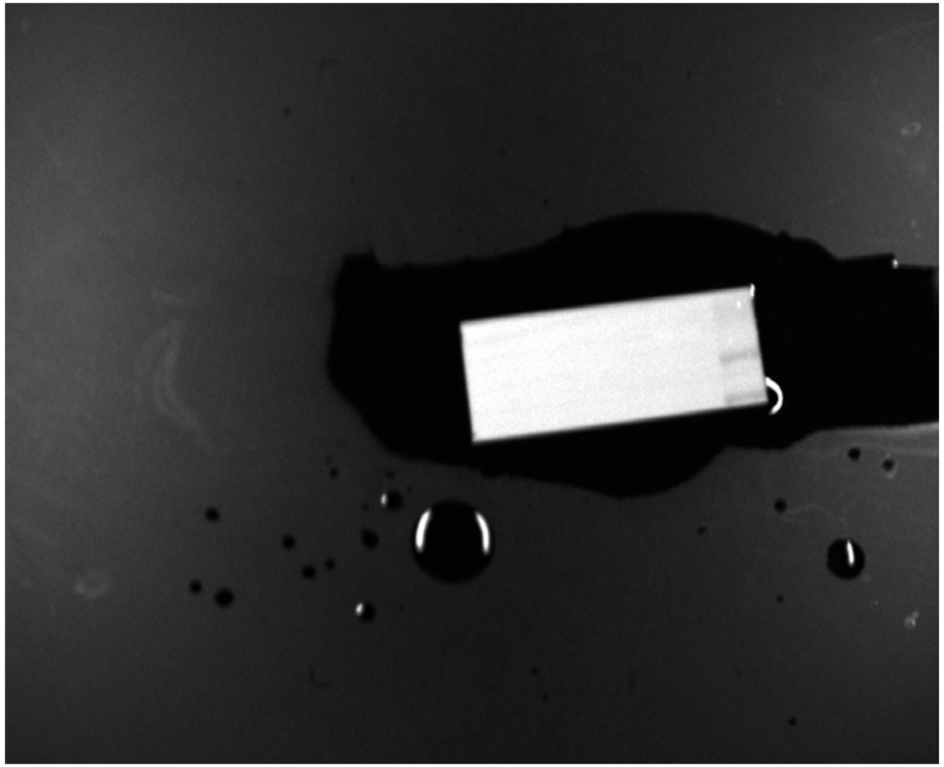

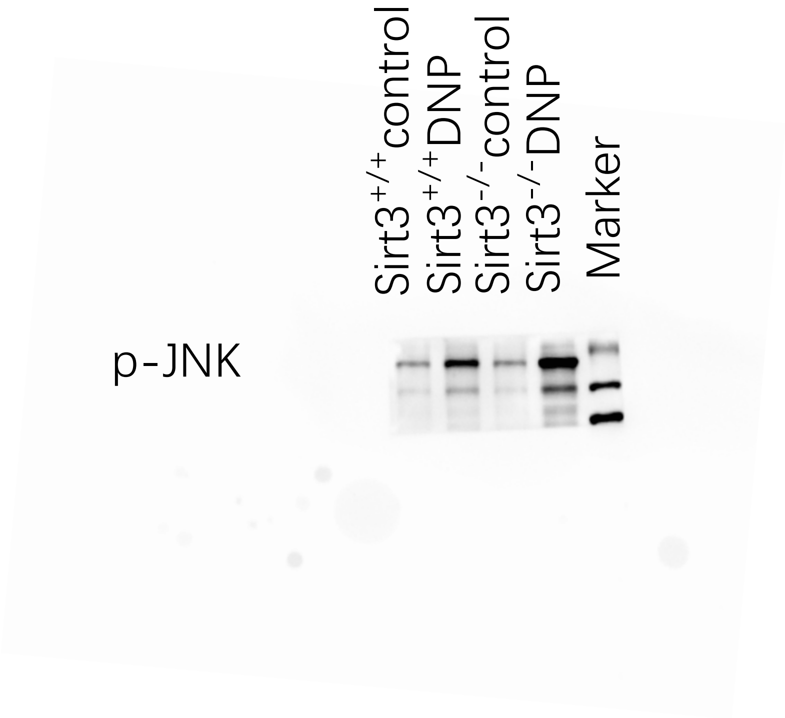

Sirt3+/+control
Sirt3+/+DNP
Sirt3-/-control
Sirt3-/-DNP
Marker
p-JNK

Fig.2f

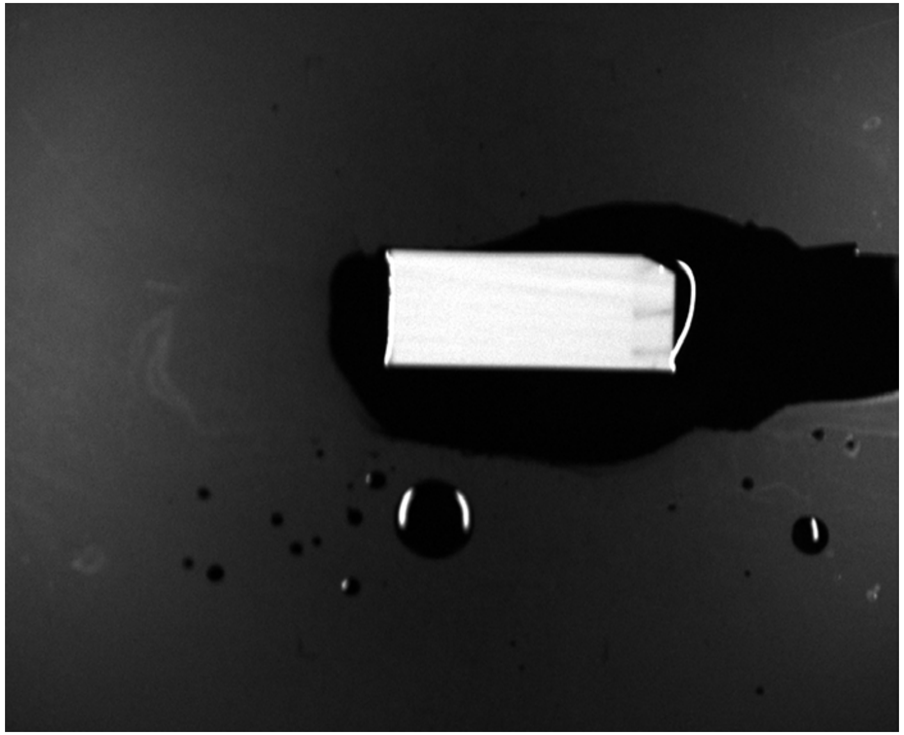

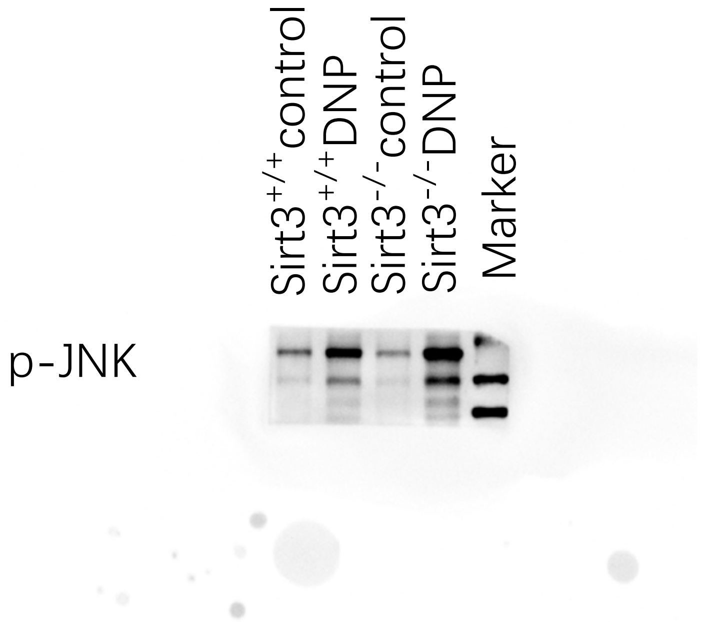
Sirt3+/+control
Sirt3+/+DNP
Sirt3-/-control
Sirt3-/-DNP
Marker
p-JNK

Fig.2g

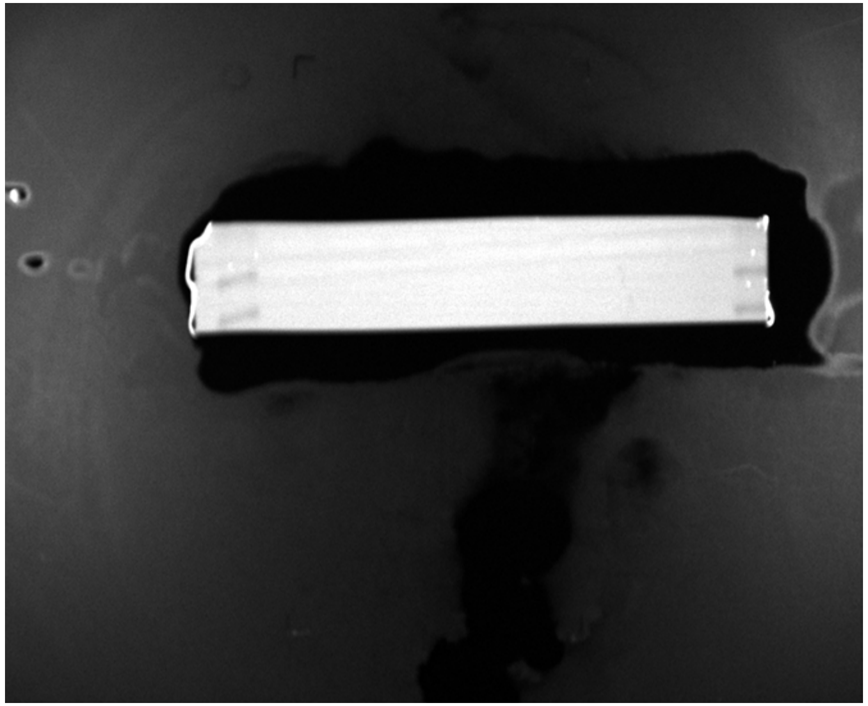

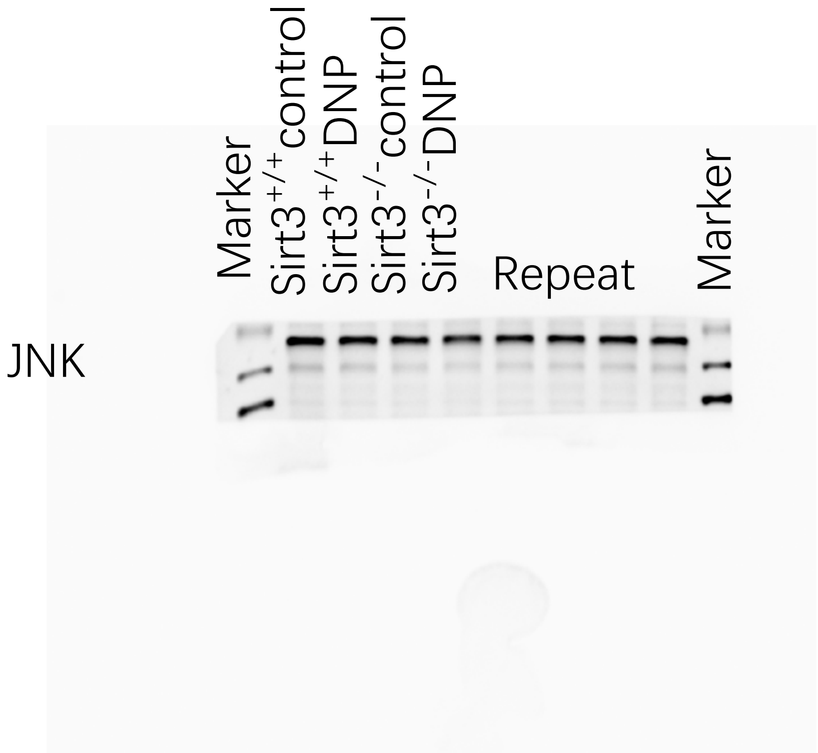
Marker
Sirt3+/+control
Sirt3+/+DNP
Sirt3-/-control
Sirt3-/-DNP
Repeat
Marker
JNK

Fig.2g

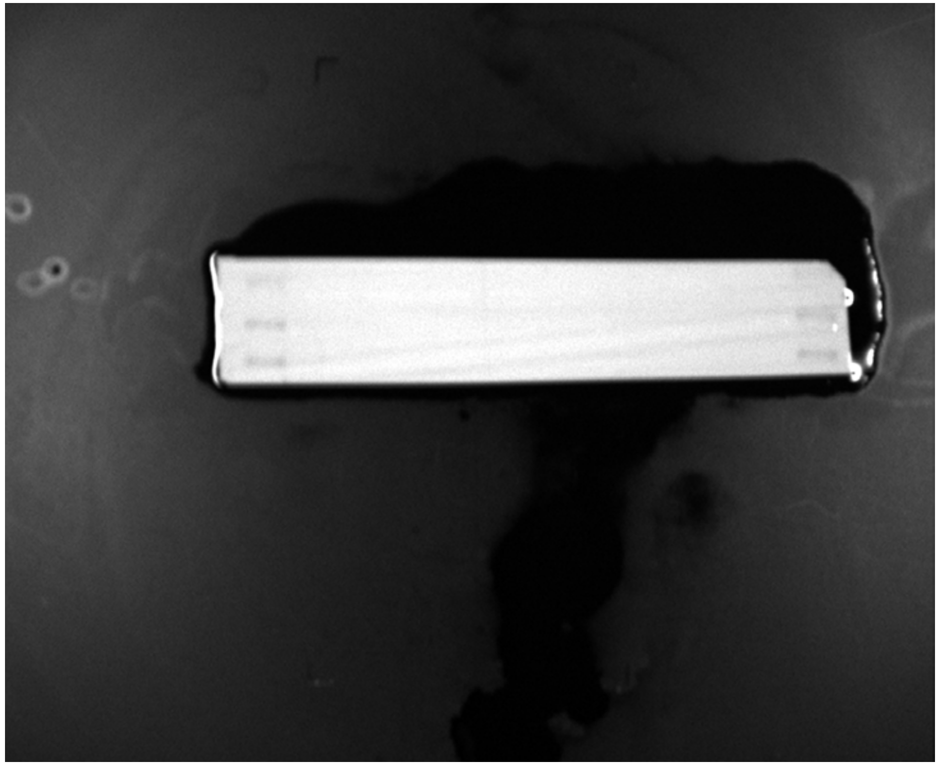

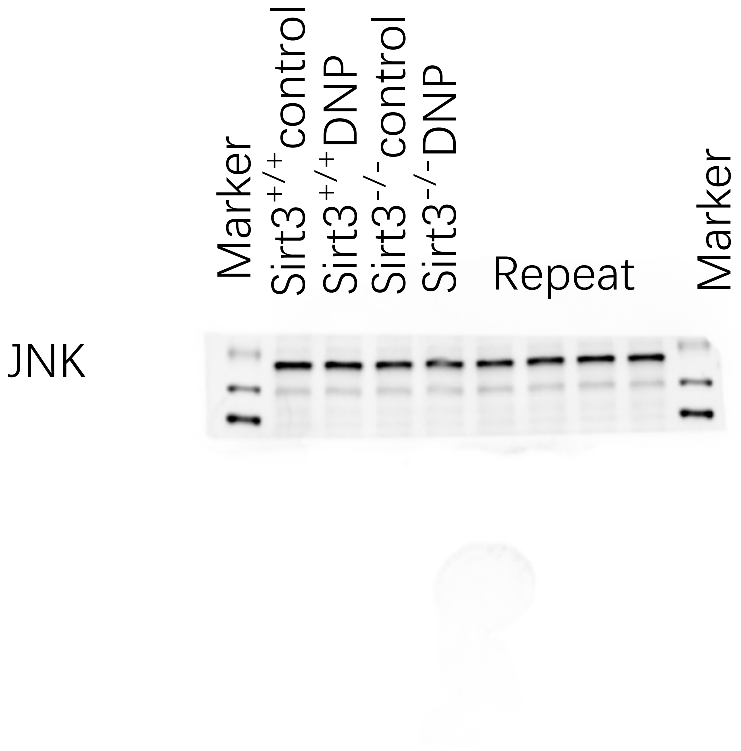

Marker
Sirt3+/+control
Sirt3+/+DNP
Sirt3-/-control
Sirt3-/-DNP
Repeat
Marker
JNK

Fig.2g

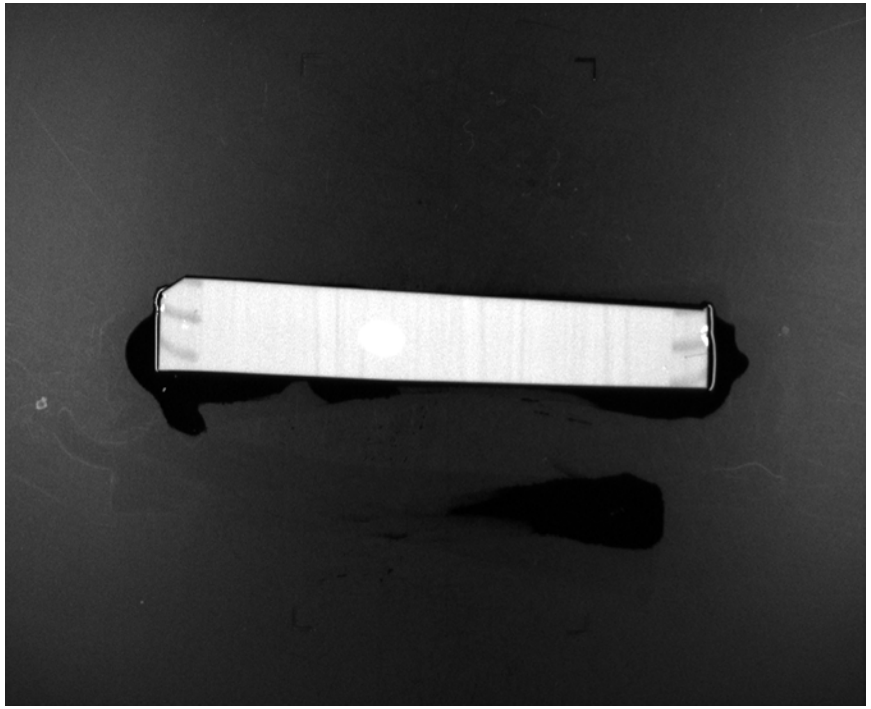

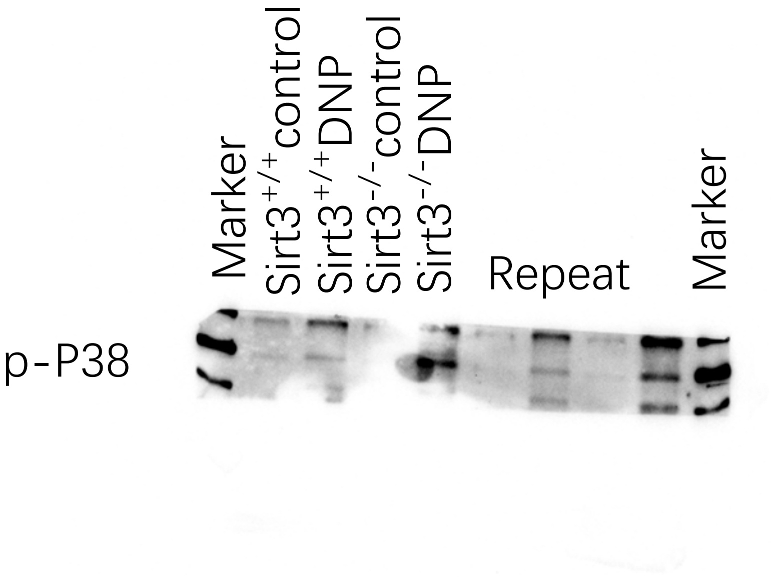

Marker
Sirt3+/+control
Sirt3+/+DNP
Sirt3-/-control
Sirt3-/-DNP
Repeat
Marker
p-P38

Fig.2g

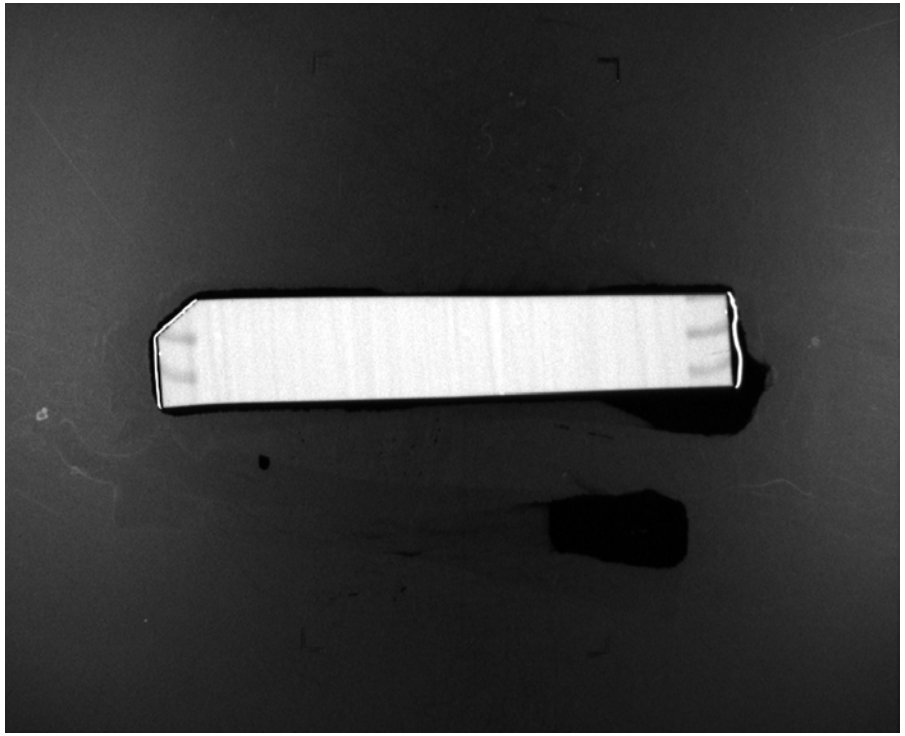

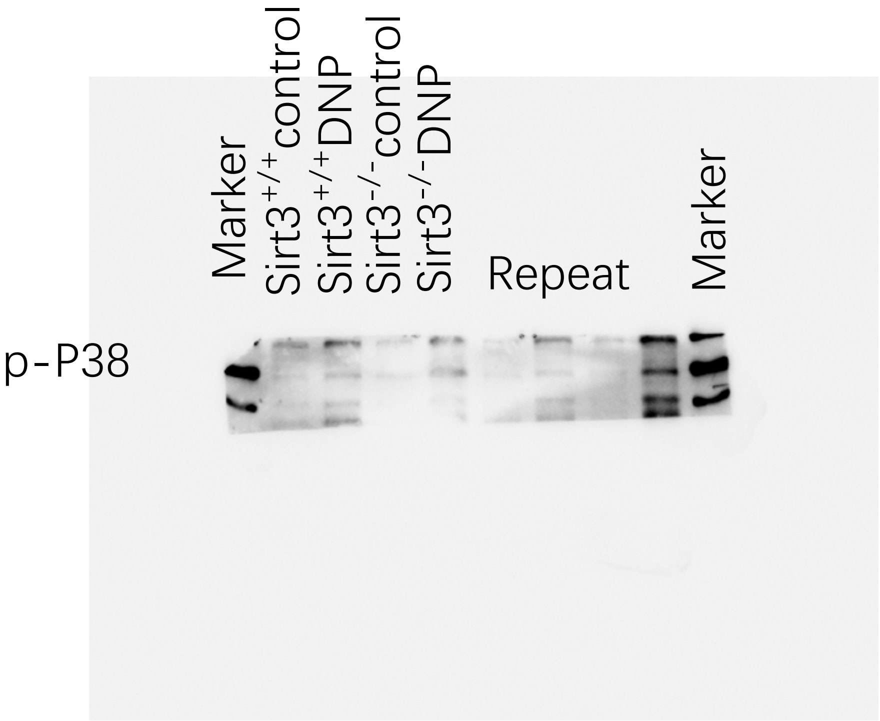

Marker
Sirt3+/+control
Sirt3+/+DNP
Sirt3-/-control
Sirt3-/-DNP
Repeat
Marker
p-P38

Fig.2g

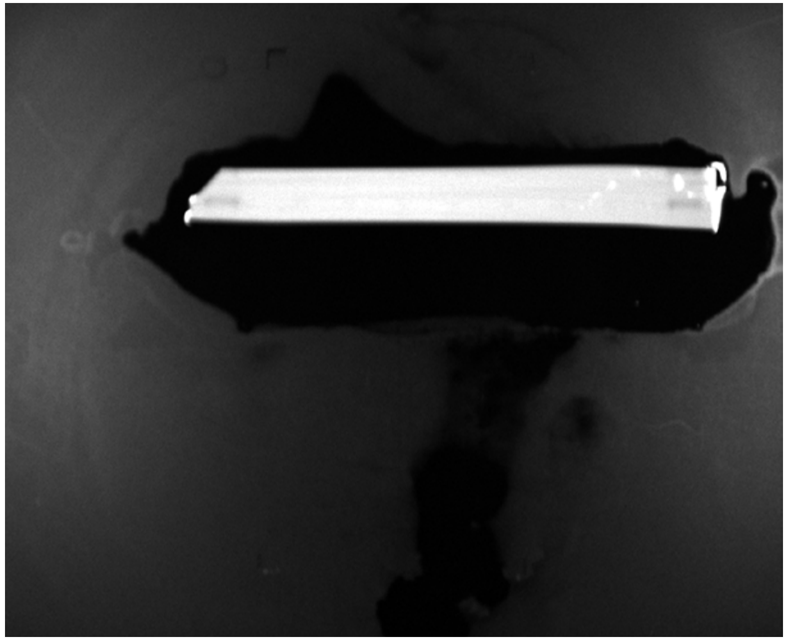

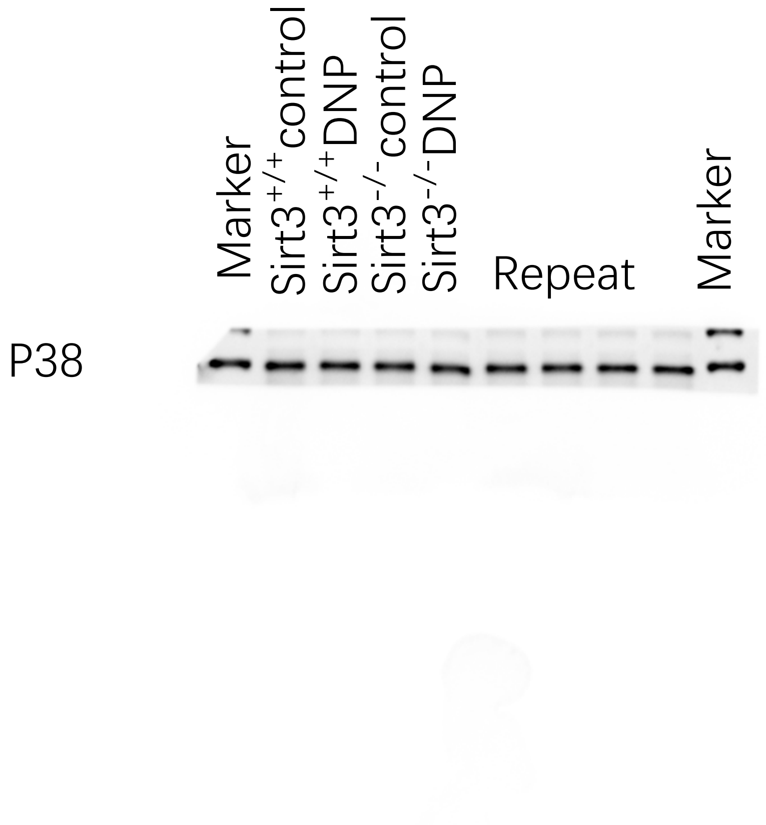
Marker
Sirt3+/+control
Sirt3+/+DNP
Sirt3-/-control
Sirt3-/-DNP
Repeat
Marker
P38

Fig.2g

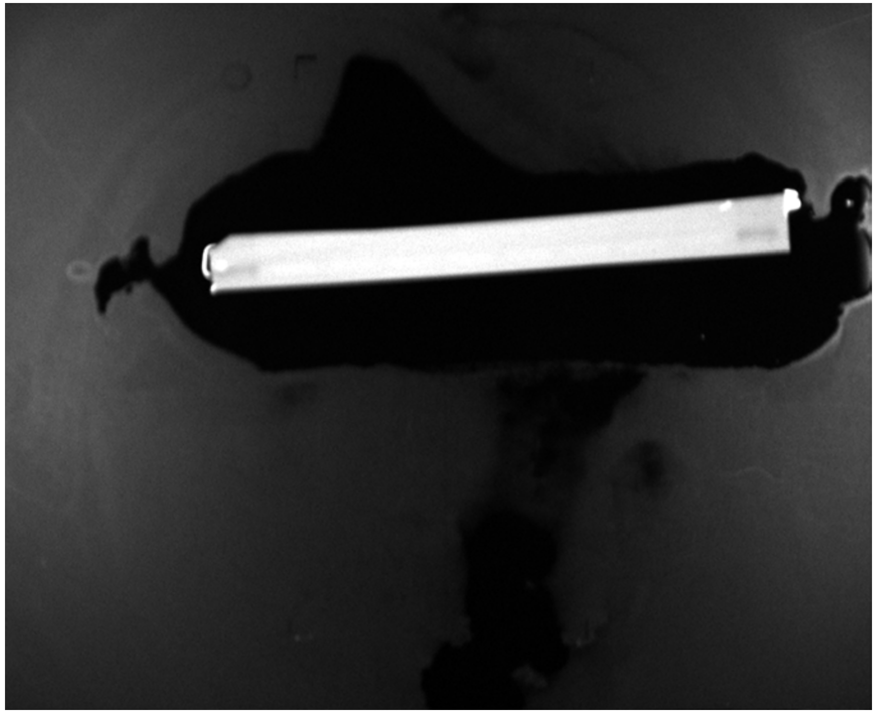

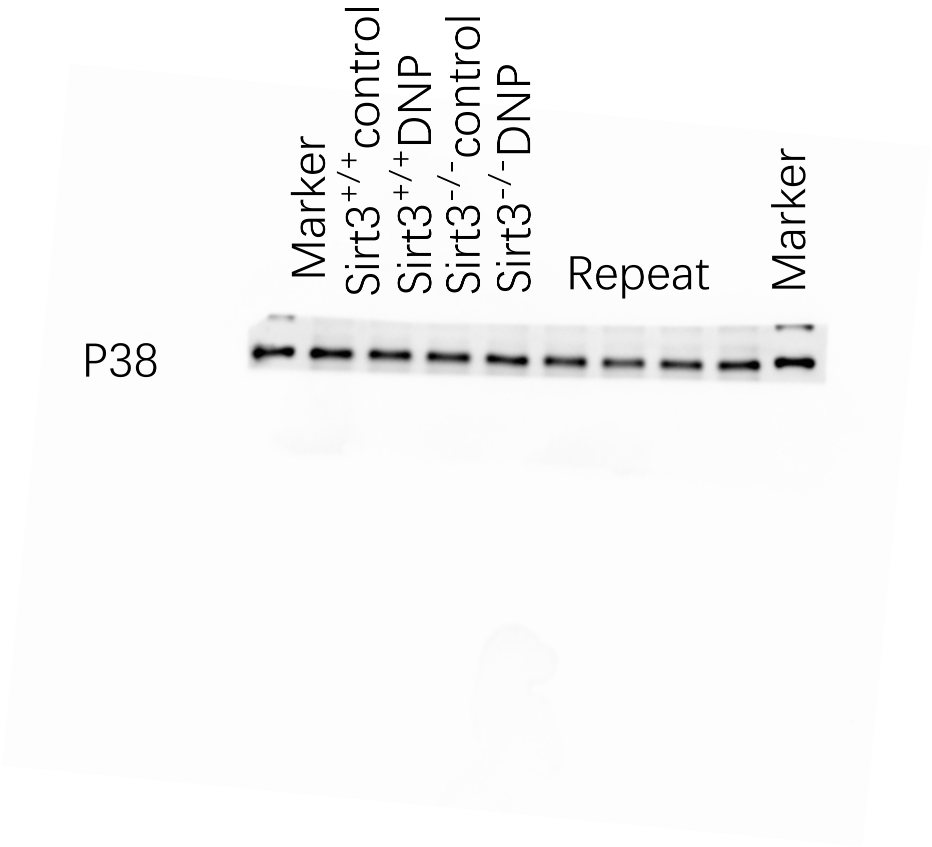
Marker
Sirt3+/+control
Sirt3+/+DNP
Sirt3-/-control
Sirt3-/-DNP
Repeat
Marker
P38

Fig.3a

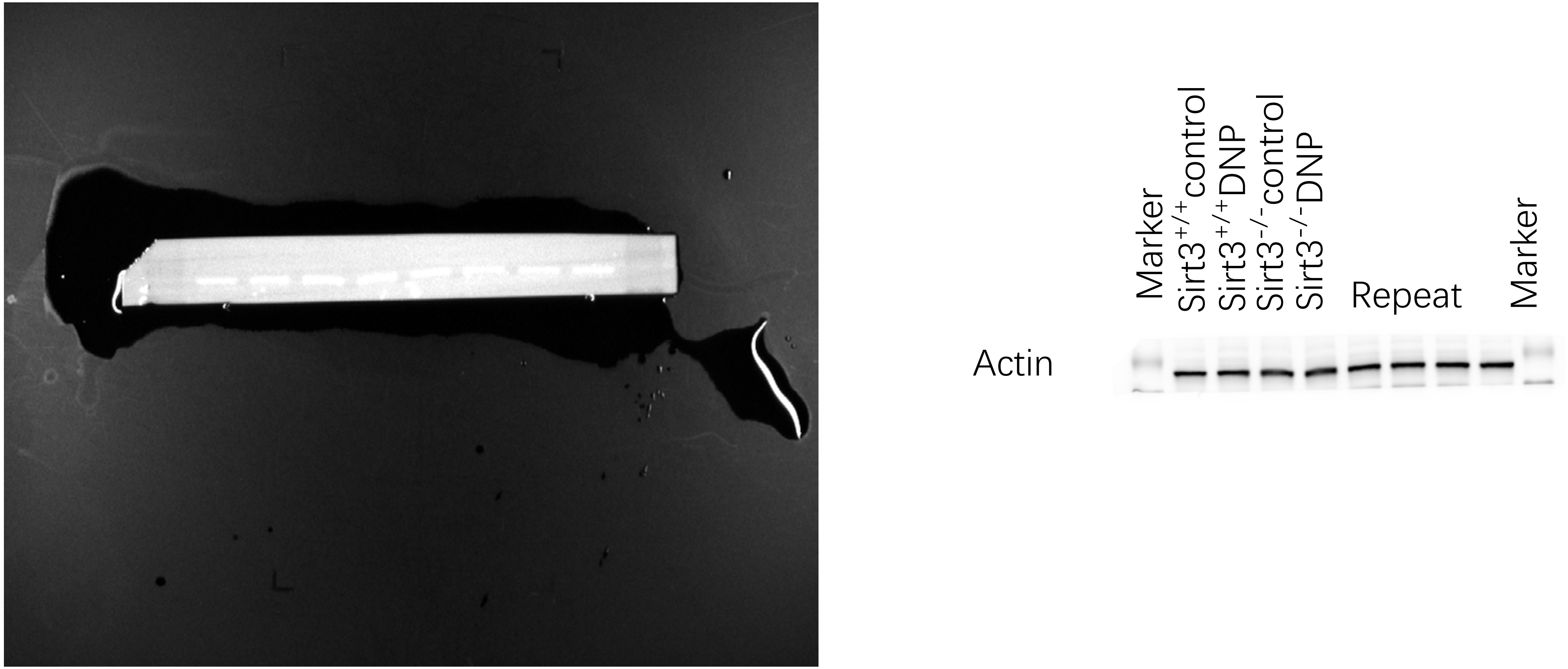
Marker
Sirt3$^{+/+}$control
Sirt3$^{+/+}$DNP
Sirt3$^{-/-}$control
Sirt3$^{-/-}$DNP
Repeat
Marker
Actin

Fig.3a

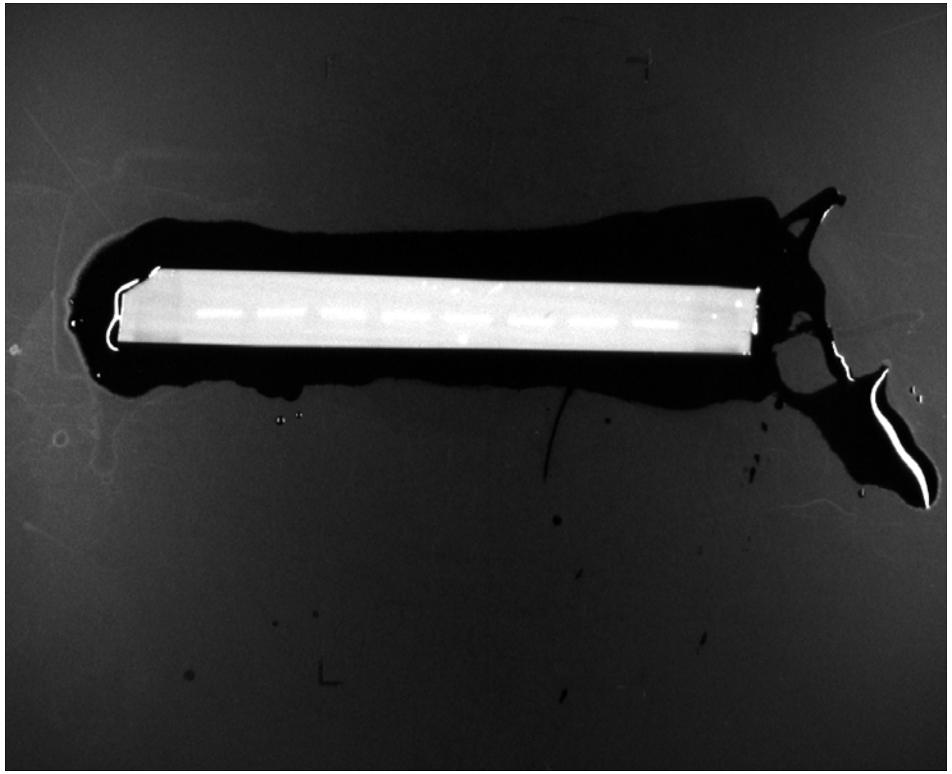

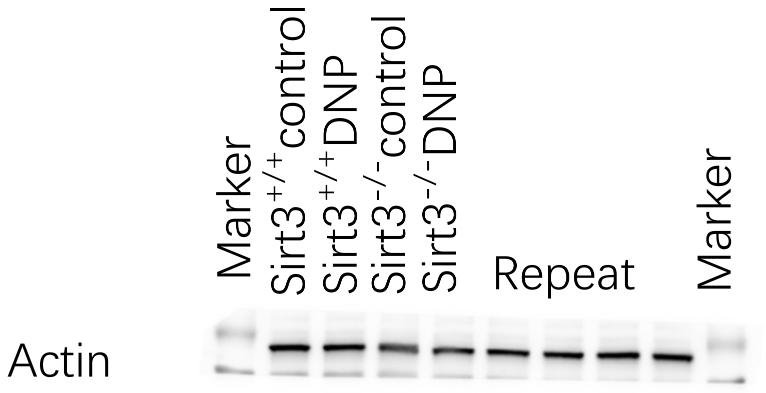
Marker
Sirt3+/+control
Sirt3+/+DNP
Sirt3-/-control
Sirt3-/-DNP
Repeat
Marker
Actin

Fig.3a

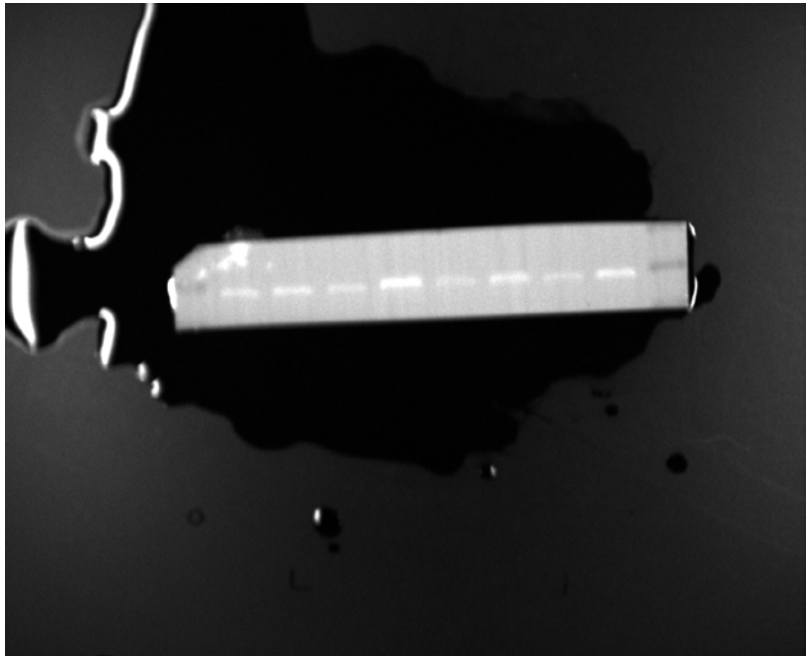

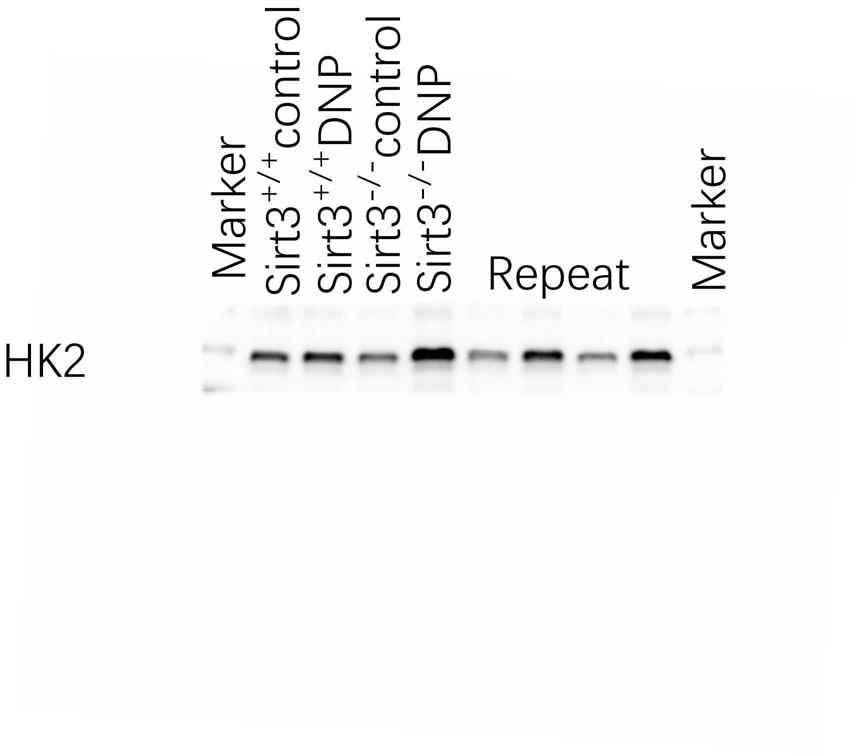
Marker
Sirt3+/+control
Sirt3+/+DNP
Sirt3-/-control
Sirt3-/-DNP
Repeat
Marker
HK2

Fig.3a

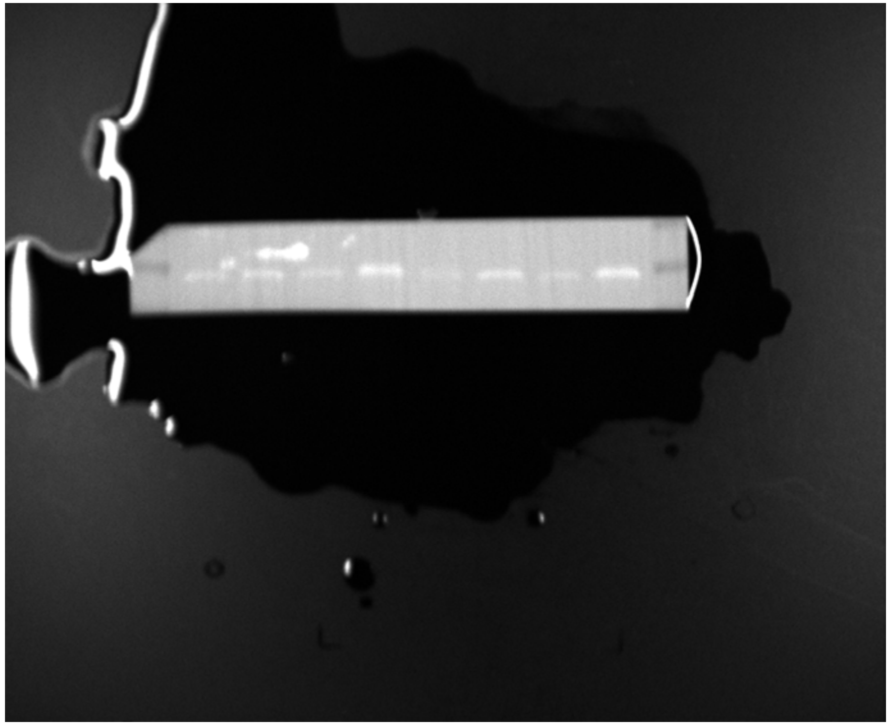

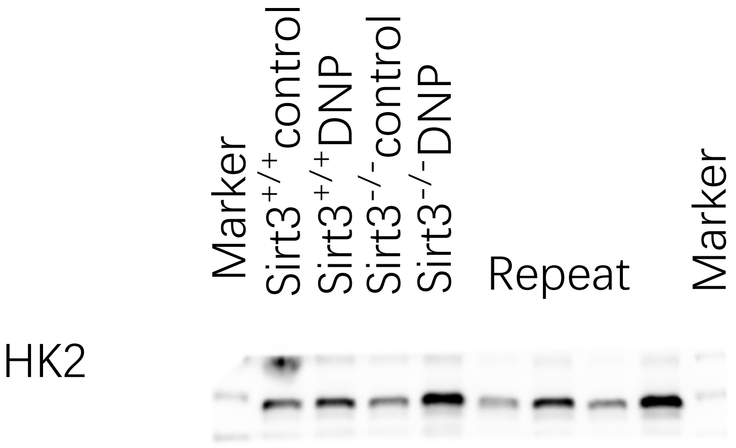
Marker
Sirt3+/+control
Sirt3+/+DNP
Sirt3-/-control
Sirt3-/-DNP
Repeat
Marker
HK2

Fig.3a

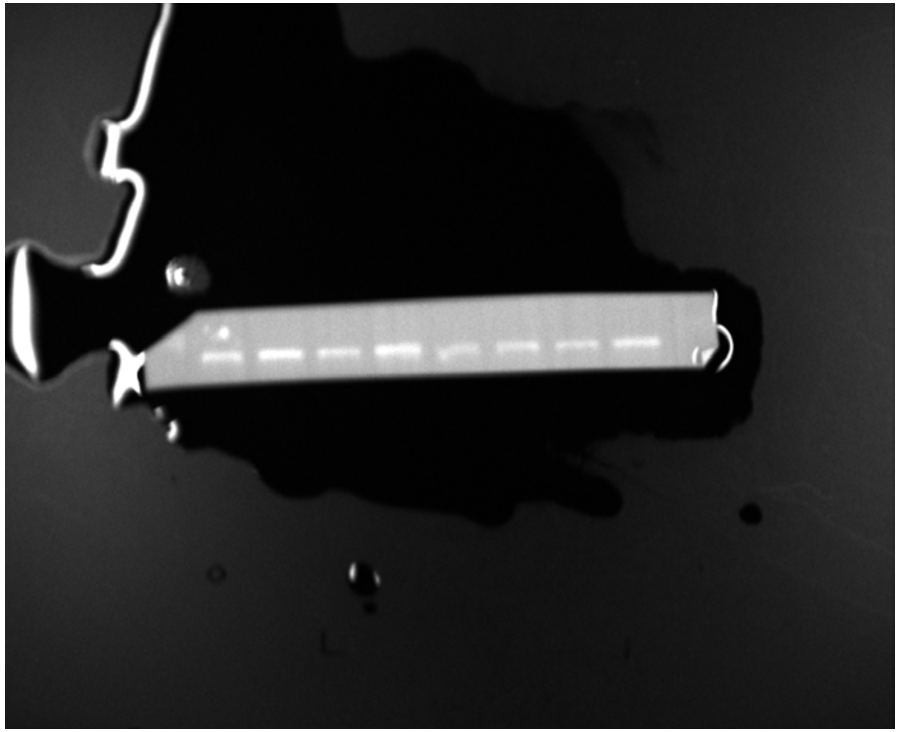

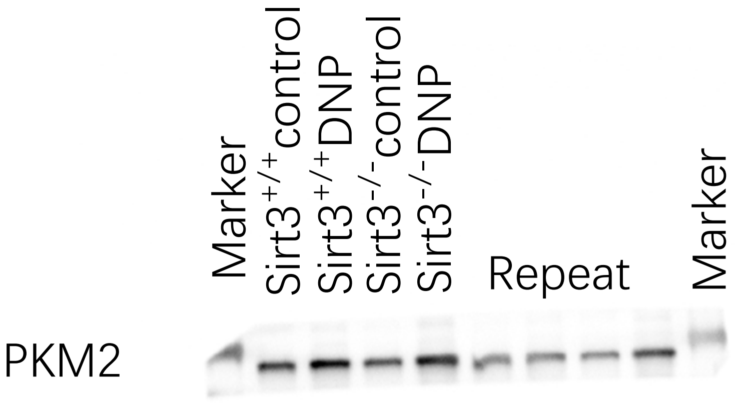
Marker
Sirt3+/+control
Sirt3+/+DNP
Sirt3-/-control
Sirt3-/-DNP
Repeat
Marker
PKM2

Fig.3a

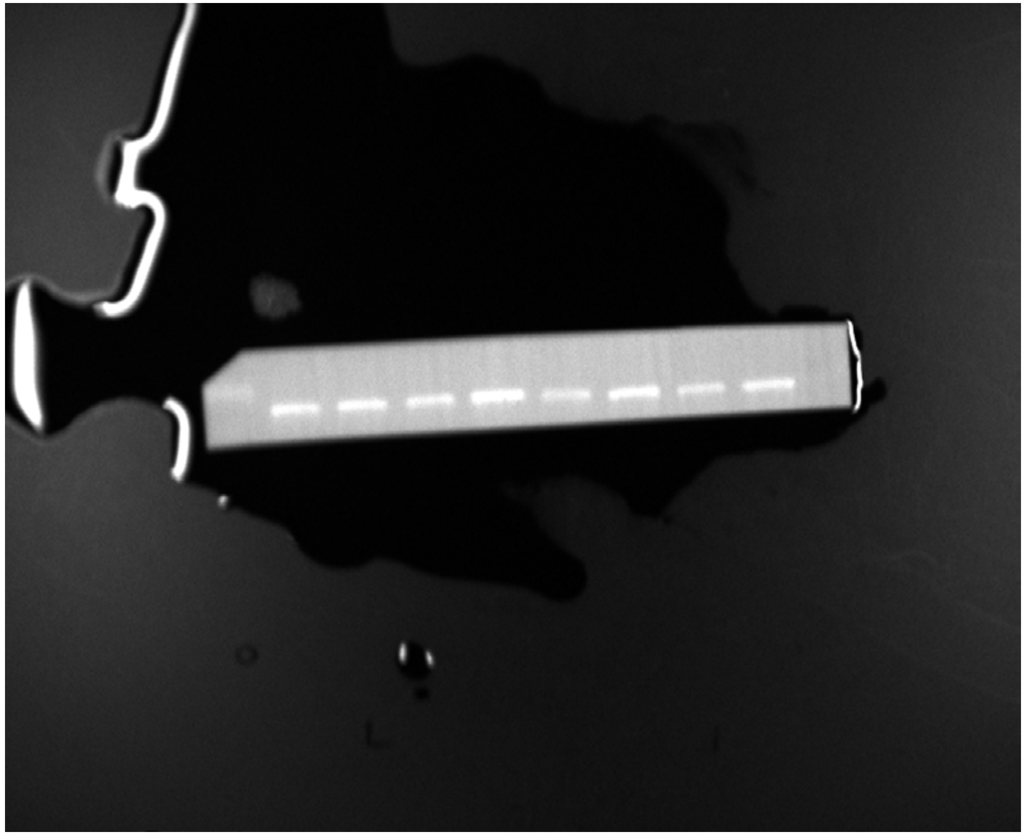

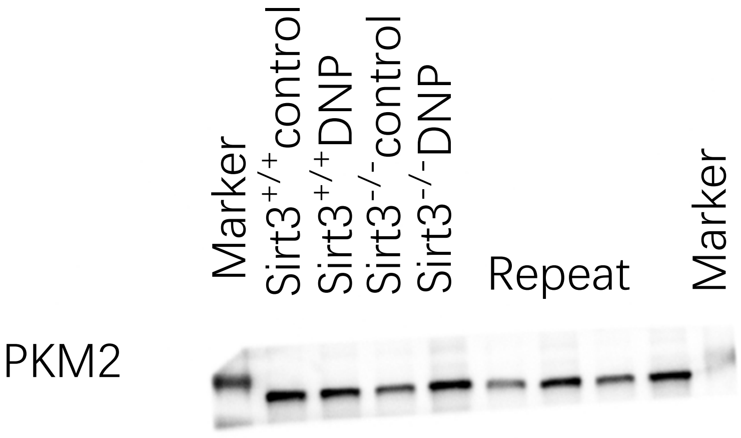
Marker
$Sirt3^{+/+}$control
$Sirt3^{+/+}$DNP
$Sirt3^{-/-}$control
$Sirt3^{-/-}$DNP
Repeat
Marker
PKM2

Fig.3a

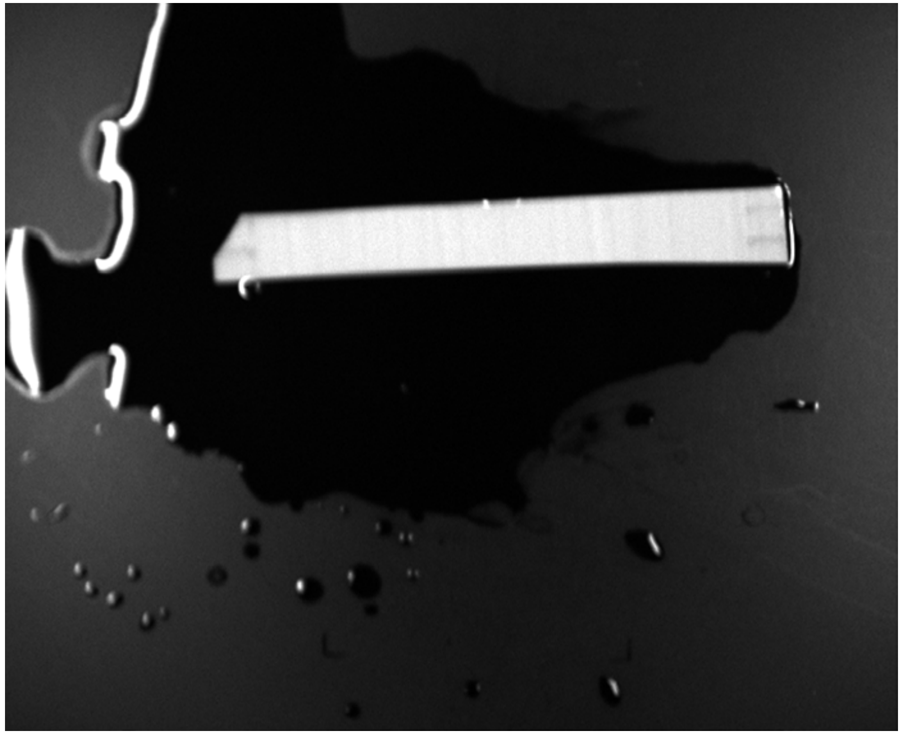

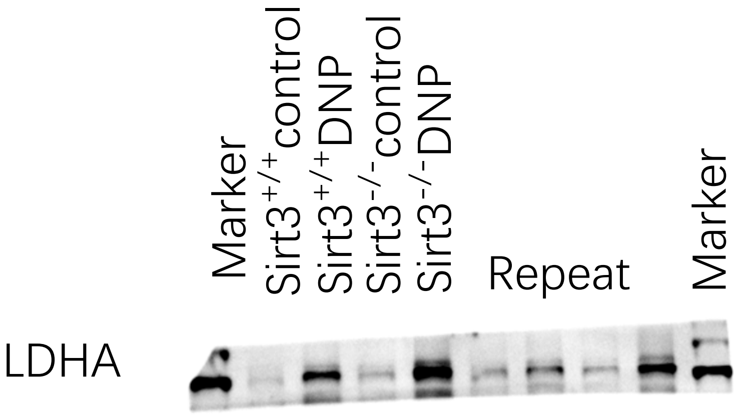
Marker
Sirt3+/+control
Sirt3+/+DNP
Sirt3-/-control
Sirt3-/-DNP
Repeat
Marker
LDHA

Fig.3a

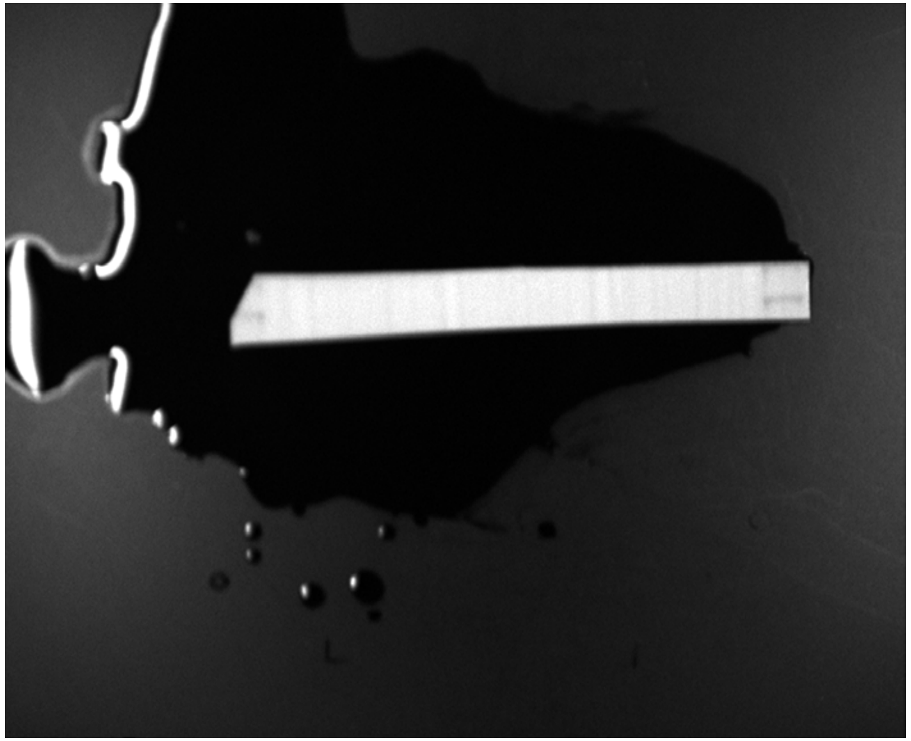

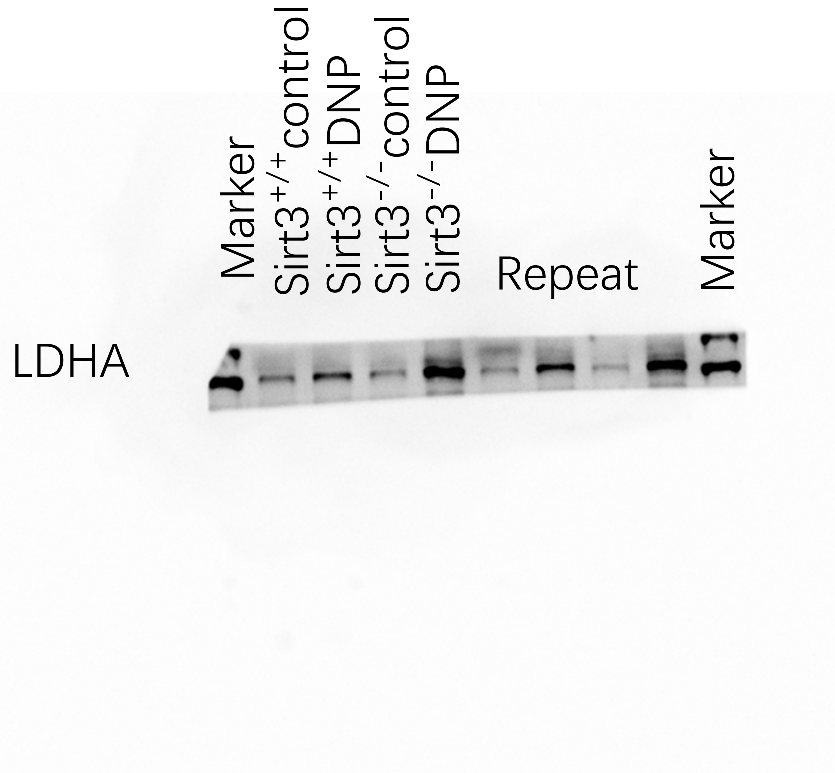
Marker
Sirt3+/+control
Sirt3+/+DNP
Sirt3-/-control
Sirt3-/-DNP
Repeat
Marker
LDHA

Fig.4a

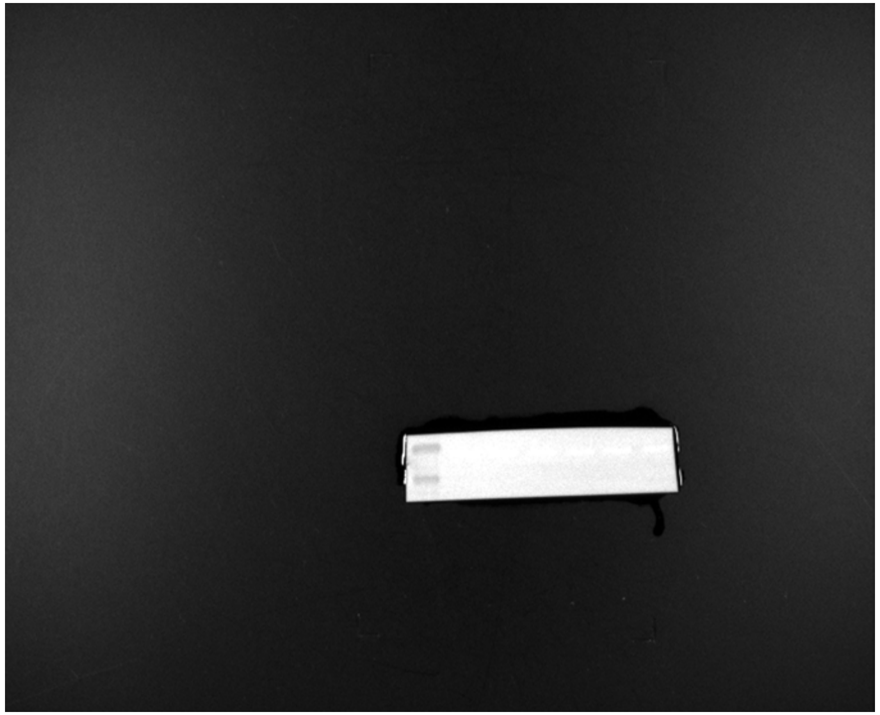

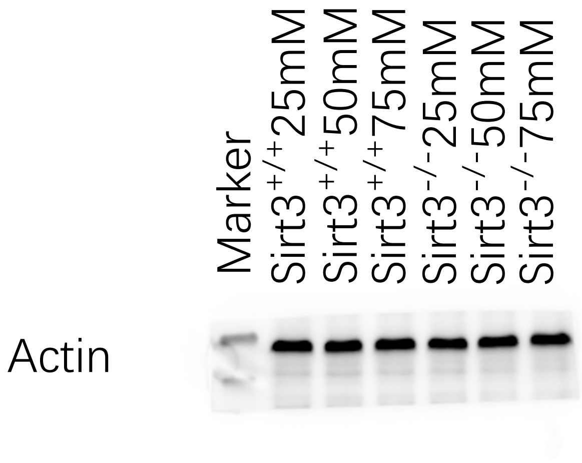
Marker
Sirt3+/+25mM
Sirt3+/+50mM
Sirt3+/+75mM
Sirt3-/-25mM
Sirt3-/-50mM
Sirt3-/-75mM
Actin

Fig.4a

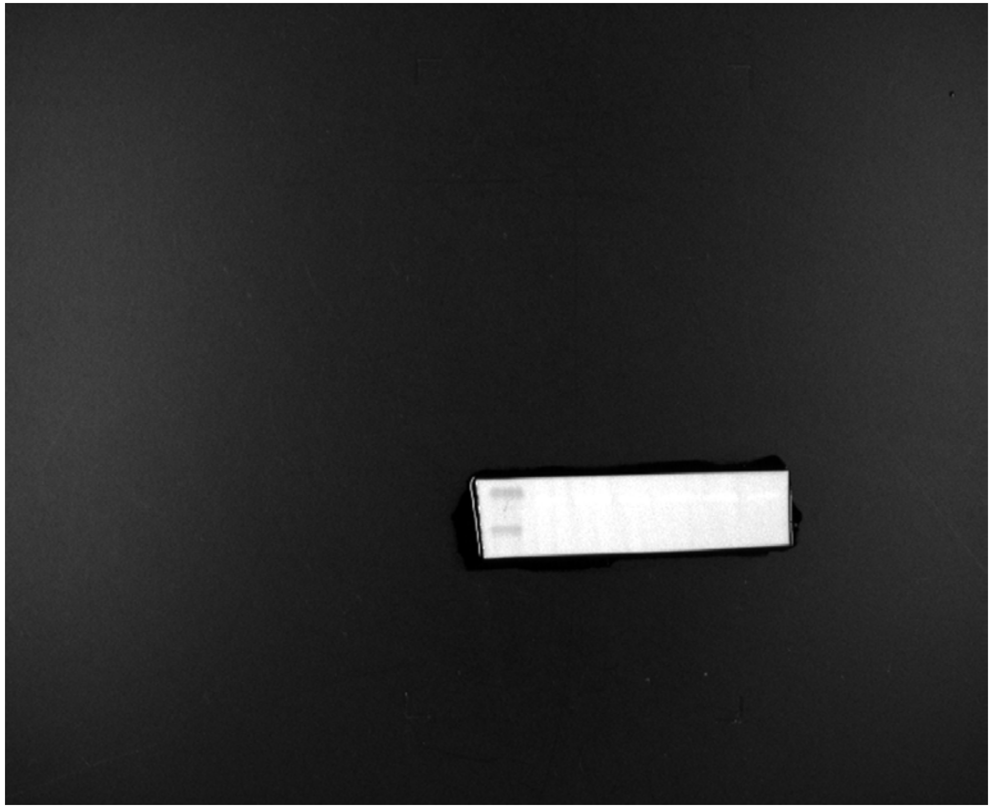

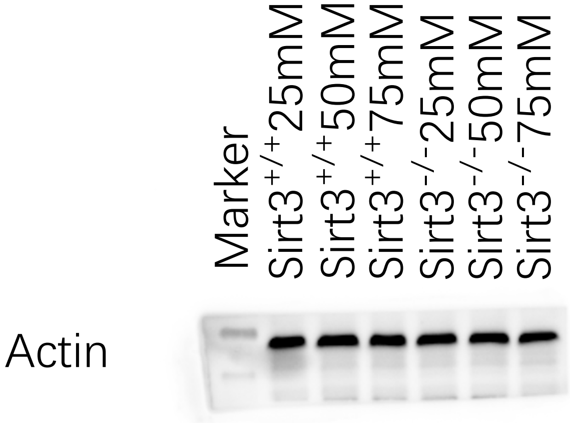

Marker
Sirt3+/+25mM
Sirt3+/+50mM
Sirt3+/+75mM
Sirt3-/-25mM
Sirt3-/-50mM
Sirt3-/-75mM
Actin

Fig.4a

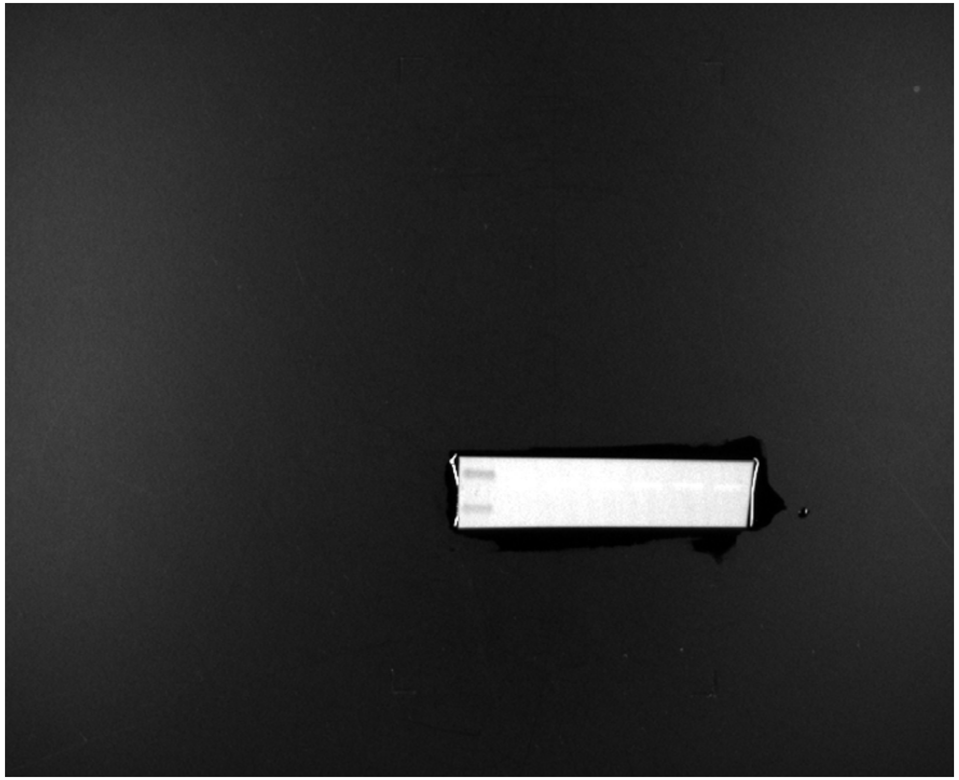

Actin

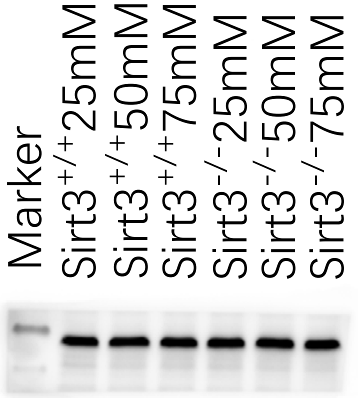
Marker
Sirt3+/+25mM
Sirt3+/+50mM
Sirt3+/+75mM
Sirt3-/-25mM
Sirt3-/-50mM
Sirt3-/-75mM

Fig.4a

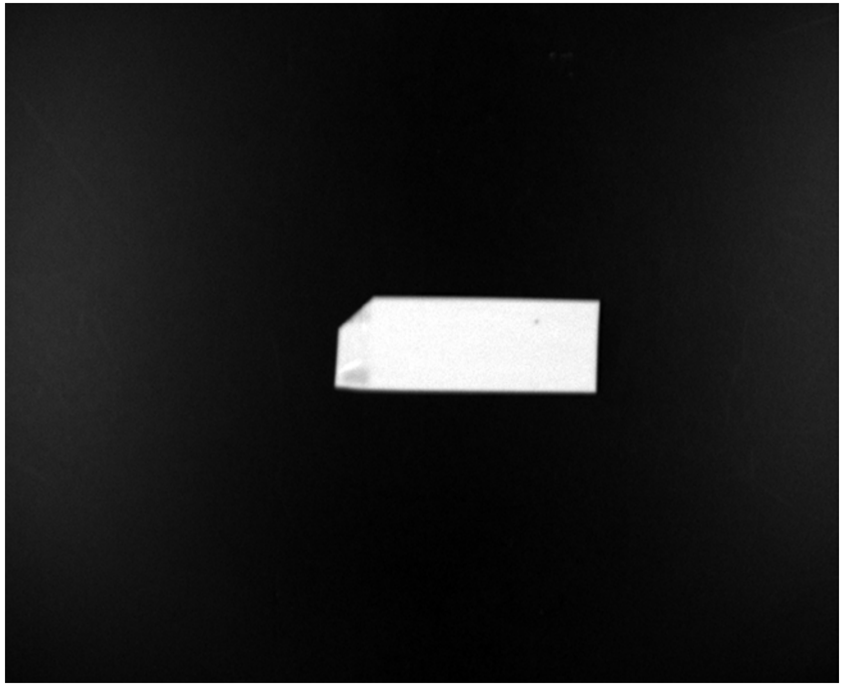

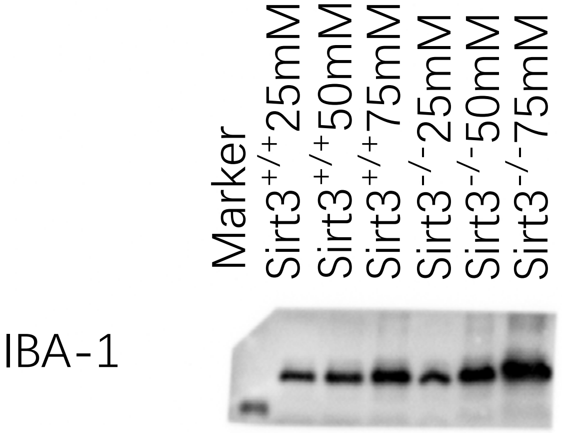
Marker
Sirt3+/+25mM
Sirt3+/+50mM
Sirt3+/+75mM
Sirt3-/-25mM
Sirt3-/-50mM
Sirt3-/-75mM
IBA-1

Fig.4a

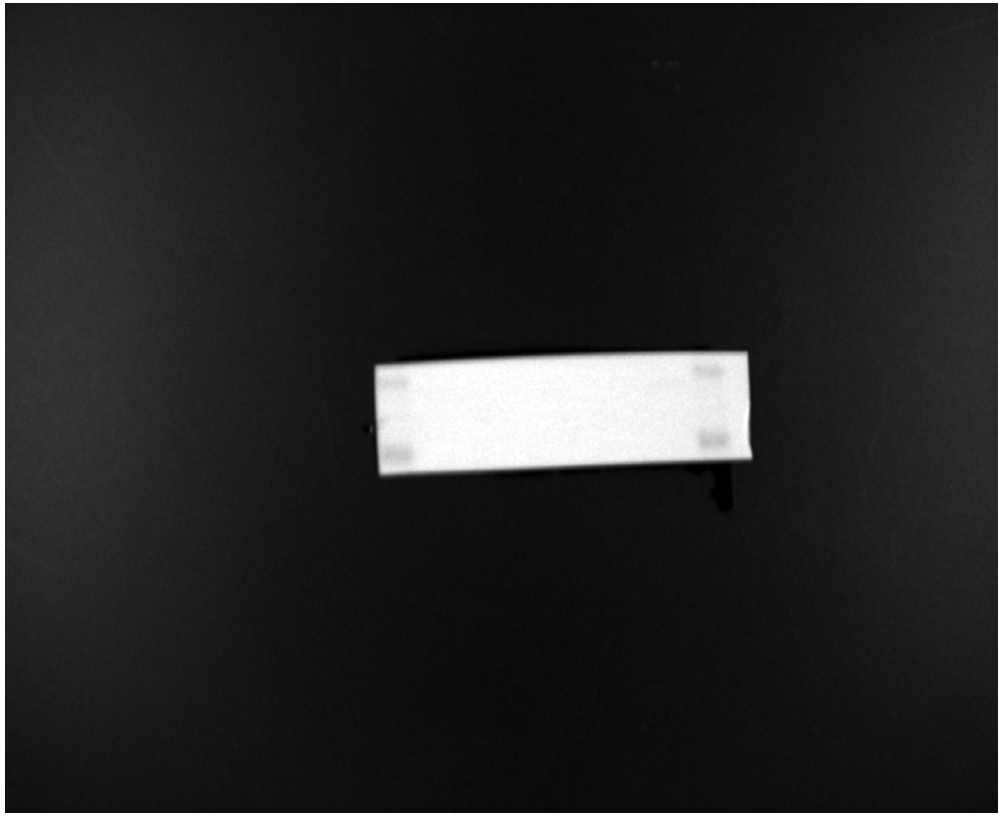

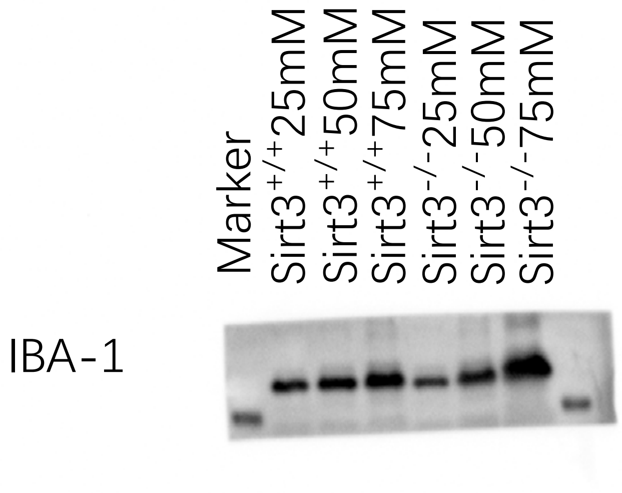
Marker
Sirt3+/+25mM
Sirt3+/+50mM
Sirt3+/+75mM
Sirt3-/-25mM
Sirt3-/-50mM
Sirt3-/-75mM
IBA-1

Fig.4a

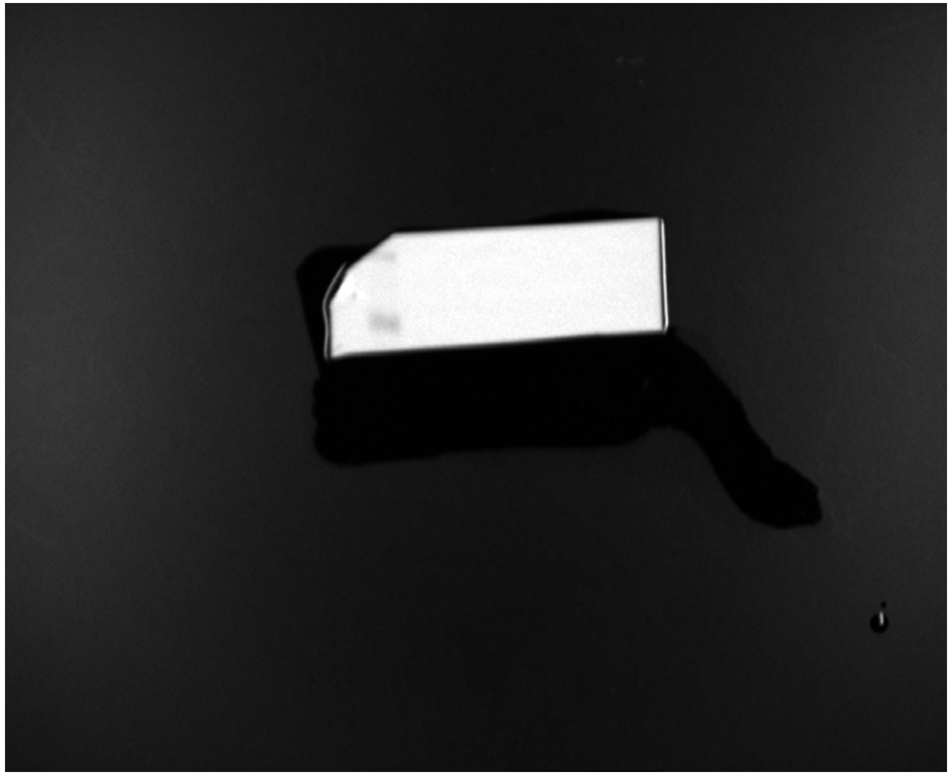

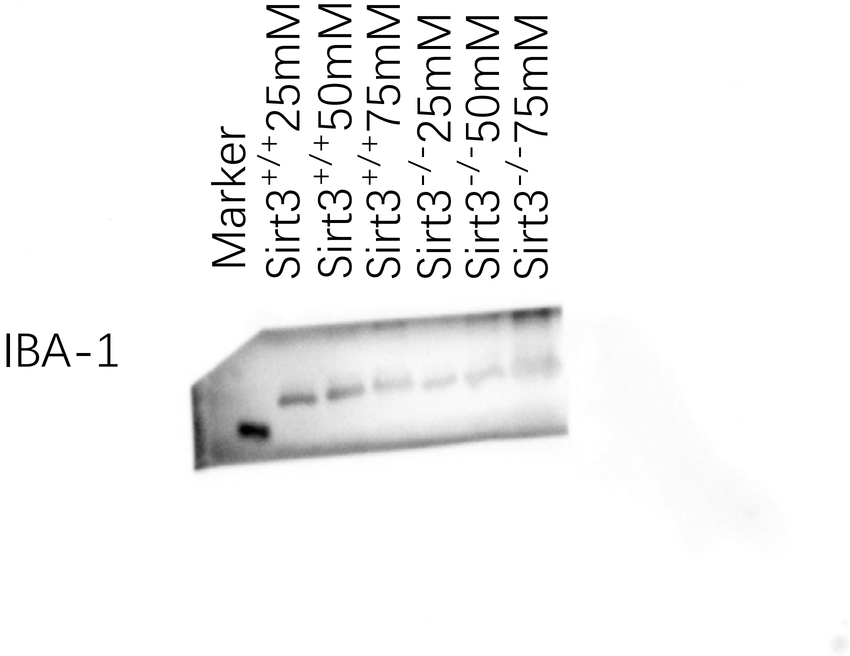
Marker
Sirt3$^{+/+}$25mM
Sirt3$^{+/+}$50mM
Sirt3$^{+/+}$75mM
Sirt3$^{-/-}$25mM
Sirt3$^{-/-}$50mM
Sirt3$^{-/-}$75mM
IBA-1

Fig.4a

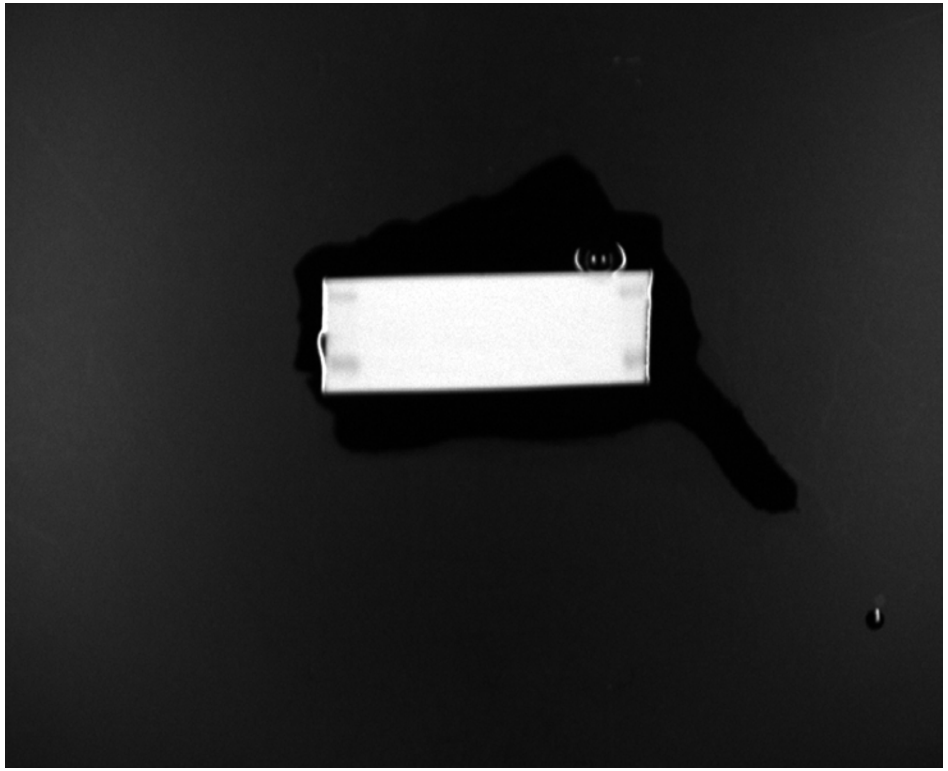

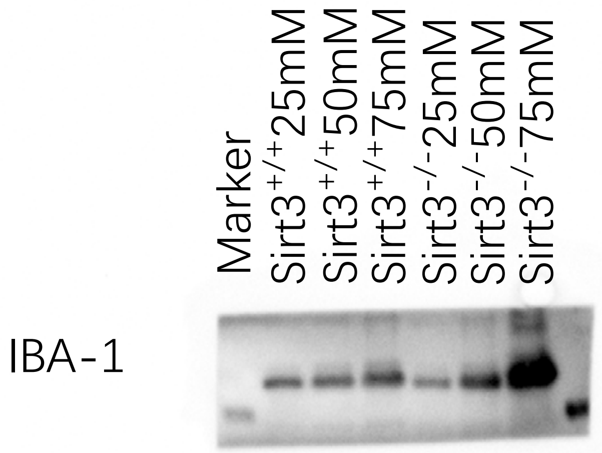
Marker
Sirt3+/+25mM
Sirt3+/+50mM
Sirt3+/+75mM
Sirt3-/-25mM
Sirt3-/-50mM
Sirt3-/-75mM
IBA-1

Fig.4b

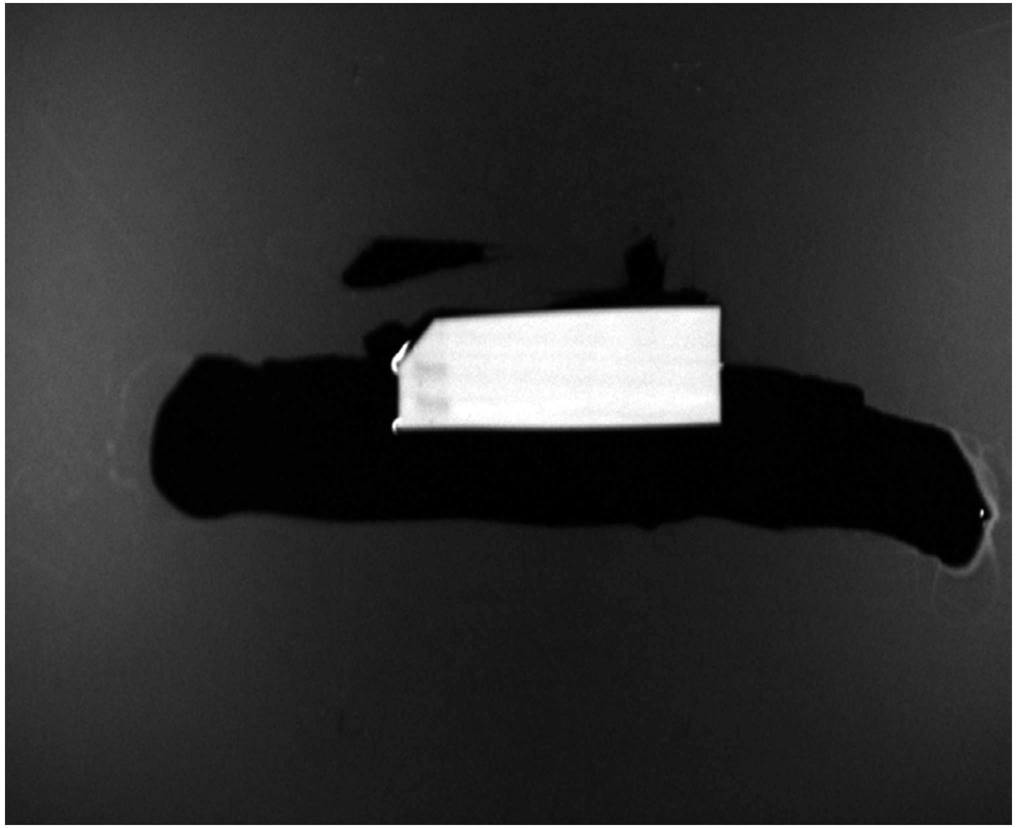

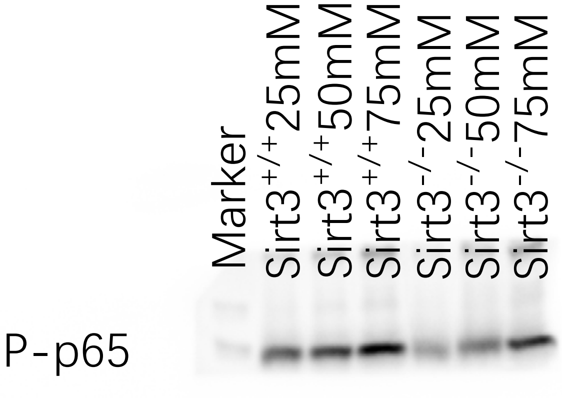
Marker
Sirt3+/+25mM
Sirt3+/+50mM
Sirt3+/+75mM
Sirt3-/-25mM
Sirt3-/-50mM
Sirt3-/-75mM
P-p65

Fig.4b

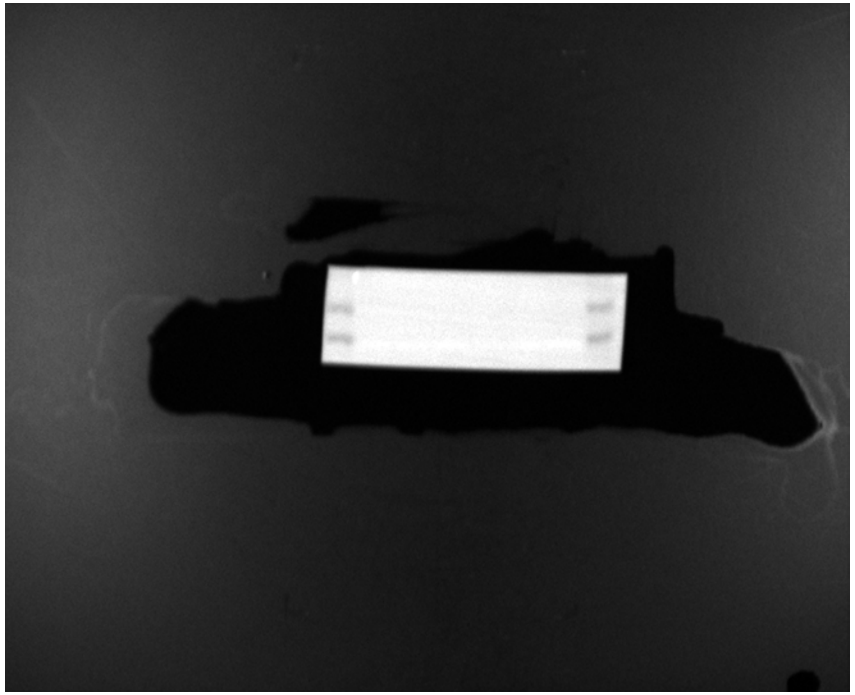

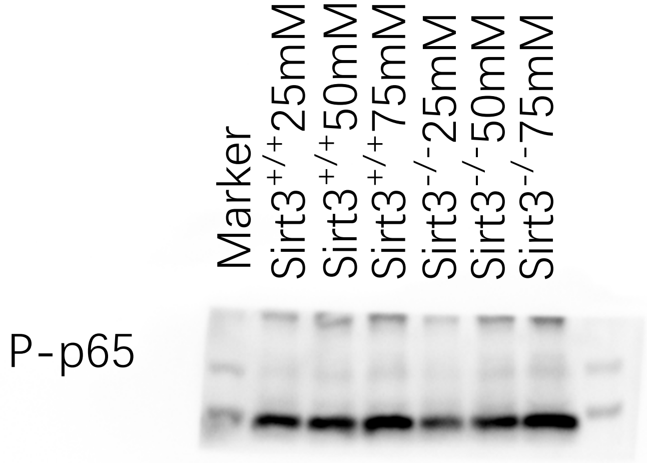
Marker
Sirt3$^{+/+}$25mM
Sirt3$^{+/+}$50mM
Sirt3$^{+/+}$75mM
Sirt3$^{-/-}$25mM
Sirt3$^{-/-}$50mM
Sirt3$^{-/-}$75mM
P-p65

Fig.4b

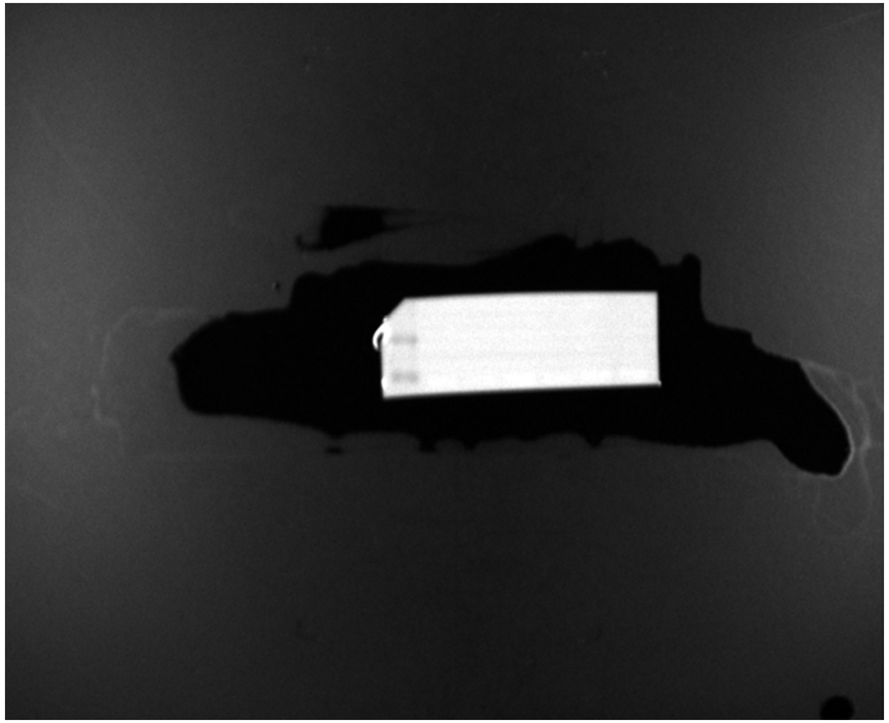

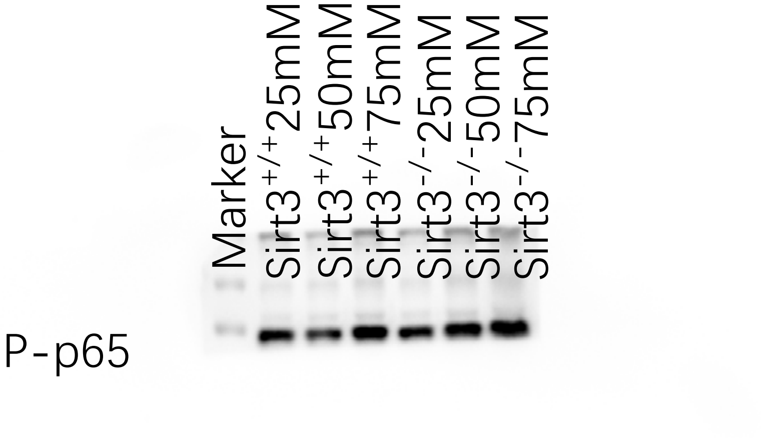
Marker
Sirt3+/+25mM
Sirt3+/+50mM
Sirt3+/+75mM
Sirt3-/-25mM
Sirt3-/-50mM
Sirt3-/-75mM
P-p65

Fig.4b

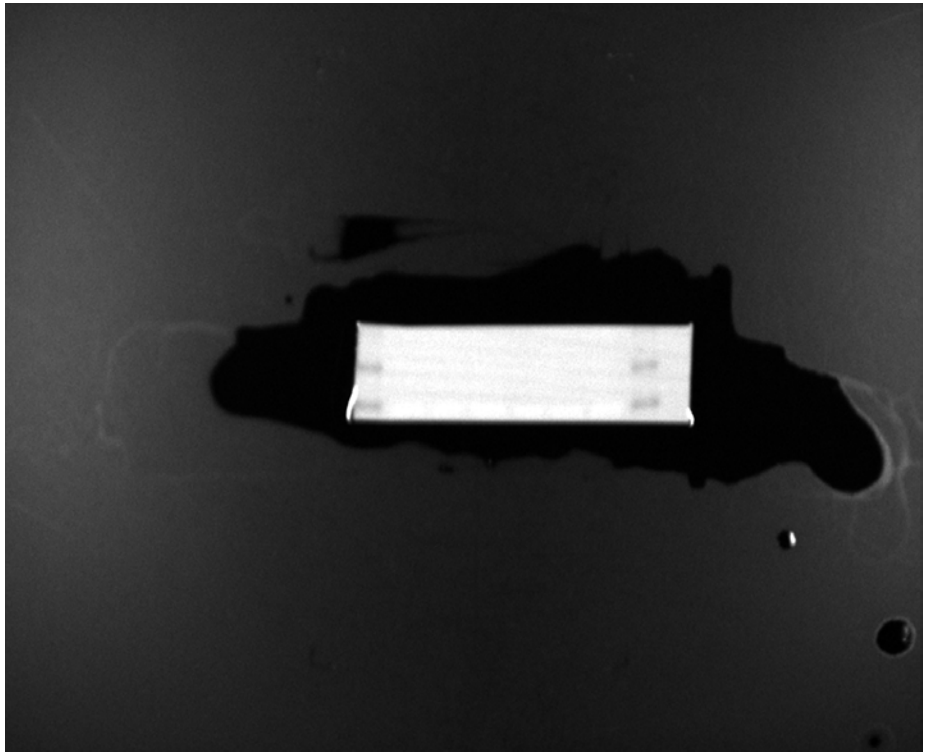

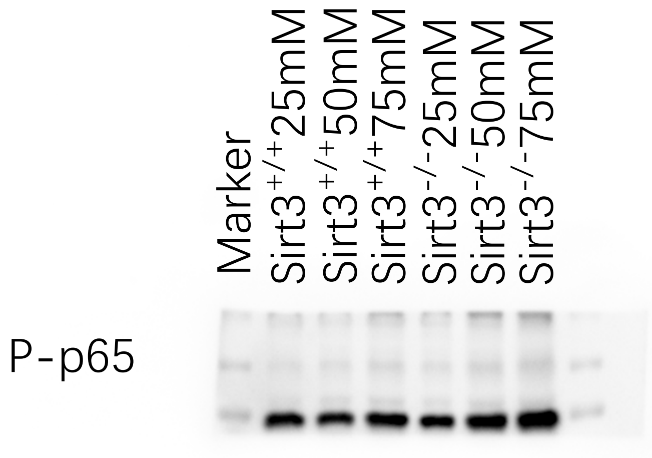

Marker
Sirt3+/+25mM
Sirt3+/+50mM
Sirt3+/+75mM
Sirt3-/-25mM
Sirt3-/-50mM
Sirt3-/-75mM
P-p65

Fig.4b

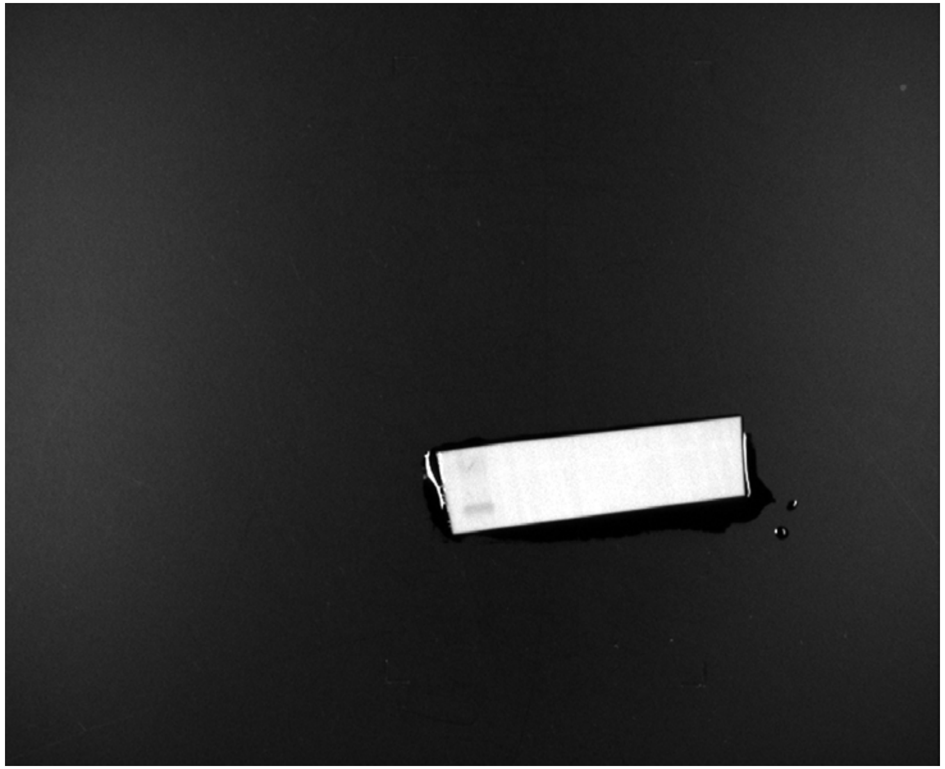

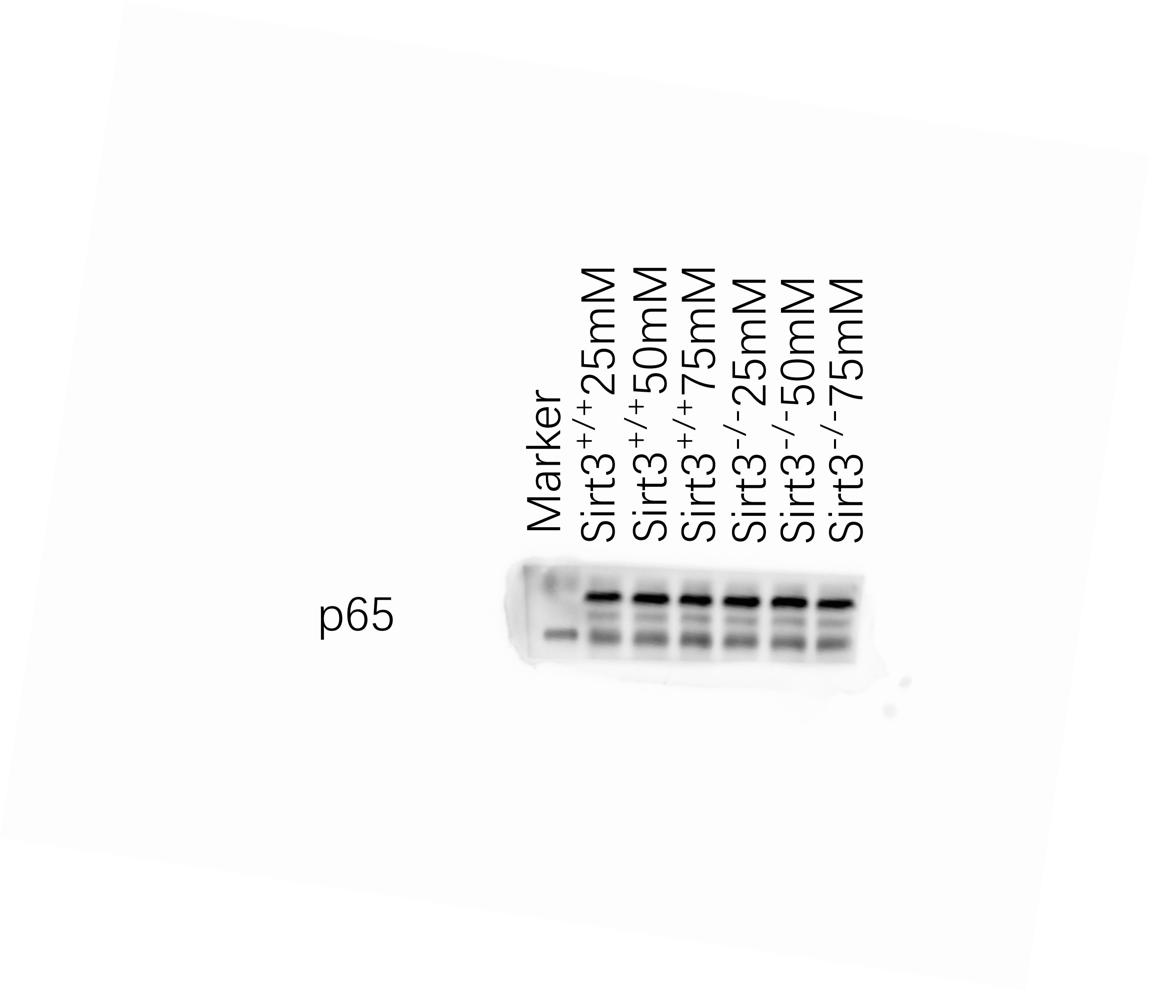

Marker
Sirt3+/+25mM
Sirt3+/+50mM
Sirt3+/+75mM
Sirt3-/-25mM
Sirt3-/-50mM
Sirt3-/-75mM
p65

Fig.4b

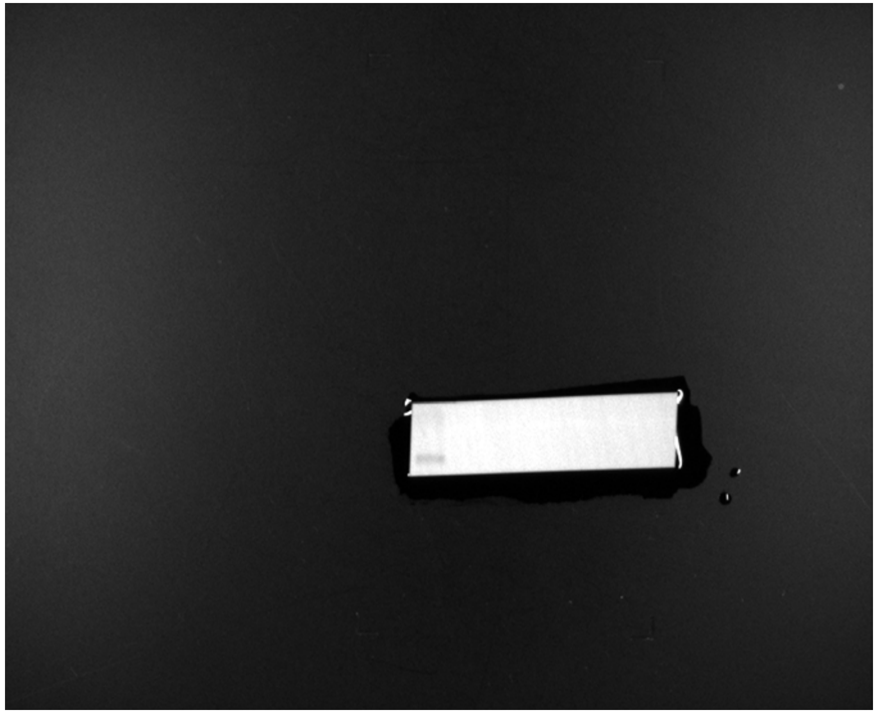

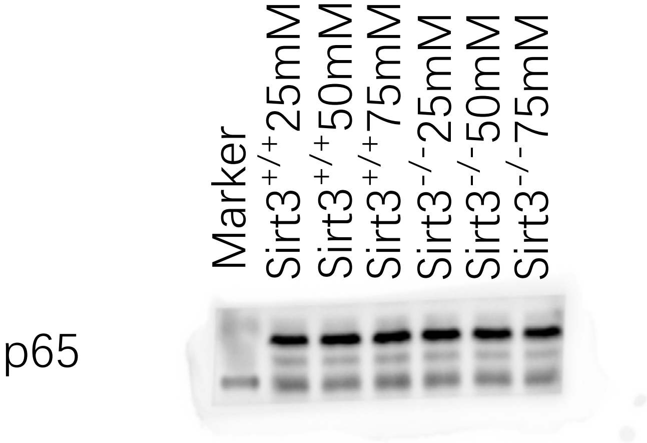
Marker
Sirt3+/+25mM
Sirt3+/+50mM
Sirt3+/+75mM
Sirt3-/-25mM
Sirt3-/-50mM
Sirt3-/-75mM
p65

Fig.4b

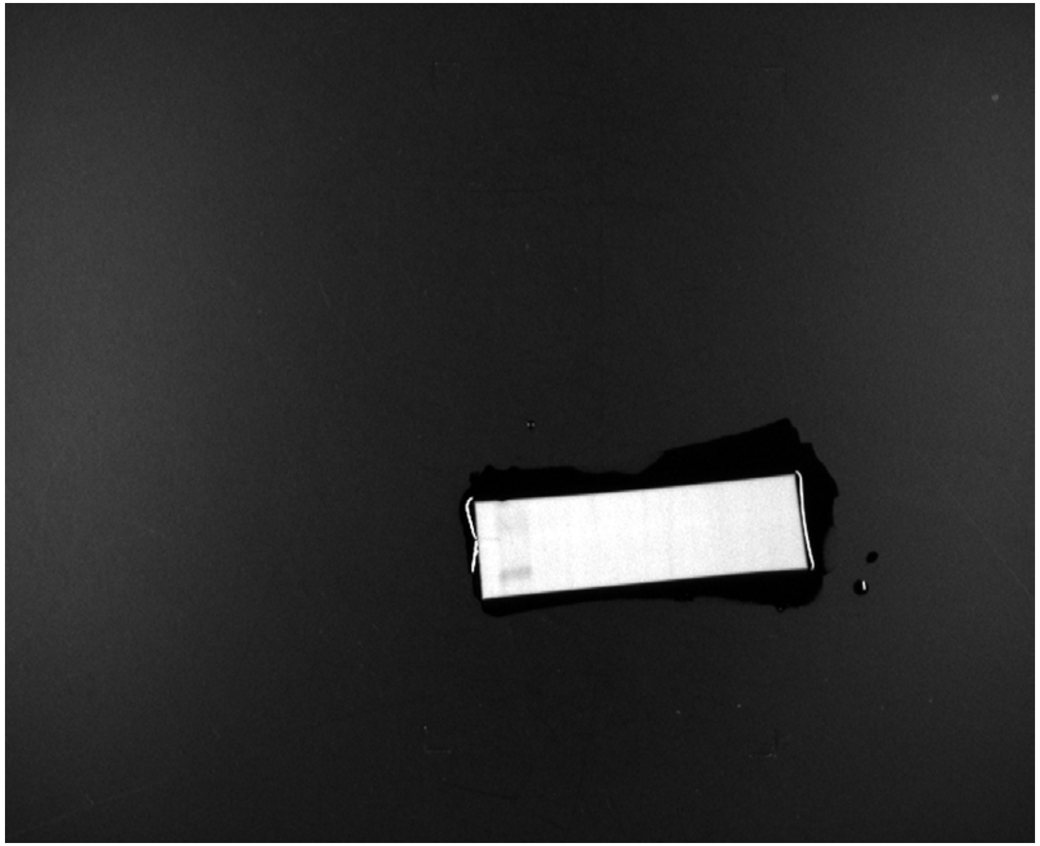

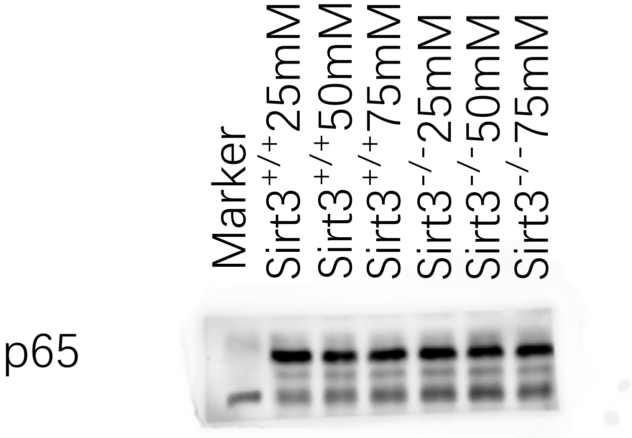
Marker
Sirt3+/+25mM
Sirt3+/+50mM
Sirt3+/+75mM
Sirt3-/-25mM
Sirt3-/-50mM
Sirt3-/-75mM
p65

Fig.4b

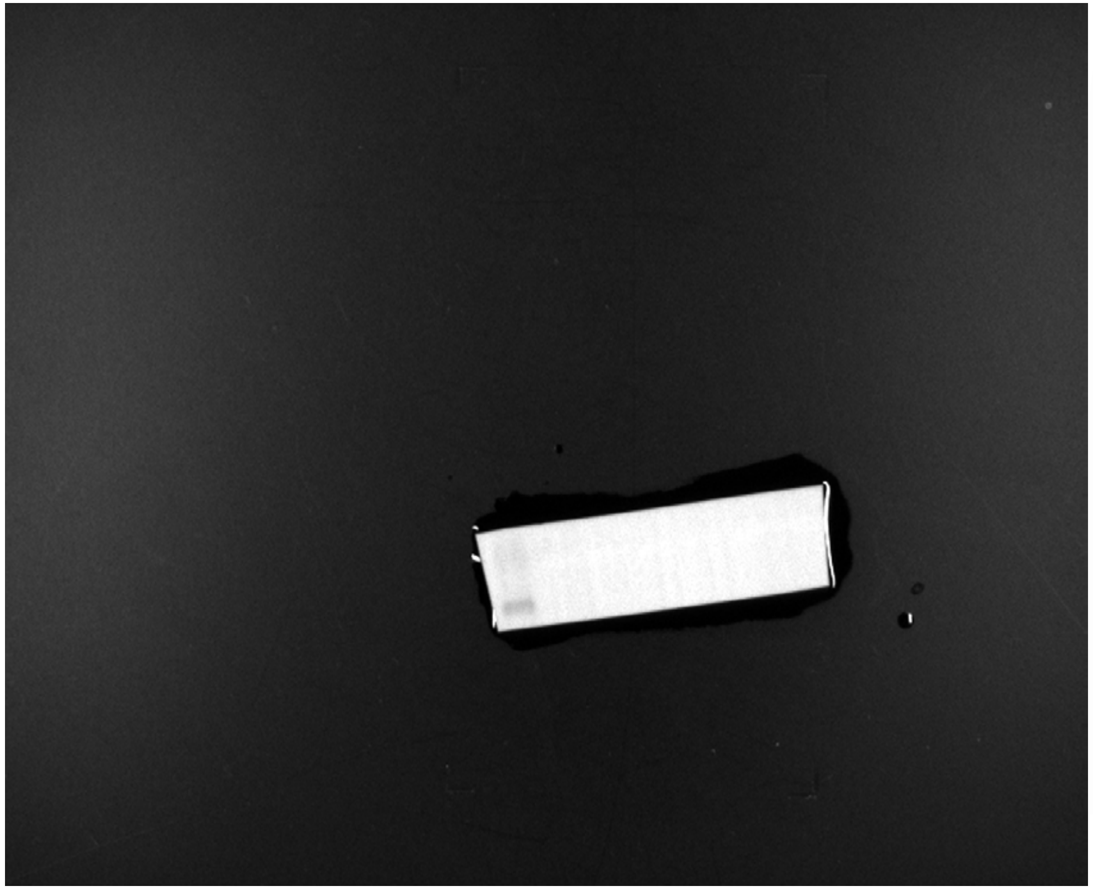

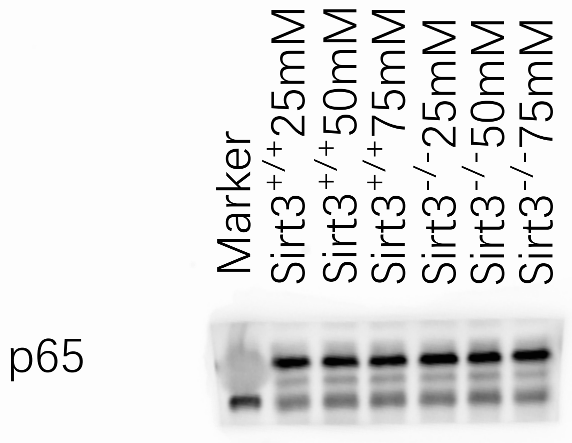
Marker
Sirt3+/+25mM
Sirt3+/+50mM
Sirt3+/+75mM
Sirt3-/-25mM
Sirt3-/-50mM
Sirt3-/-75mM
p65

Fig.4c

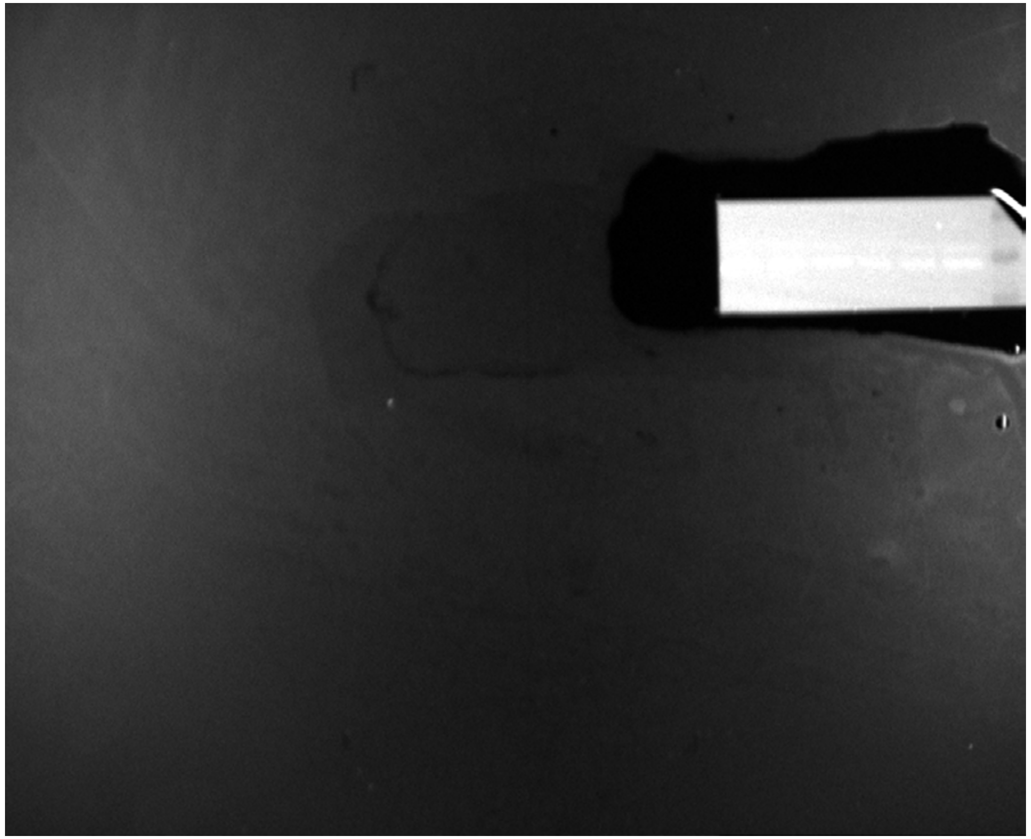

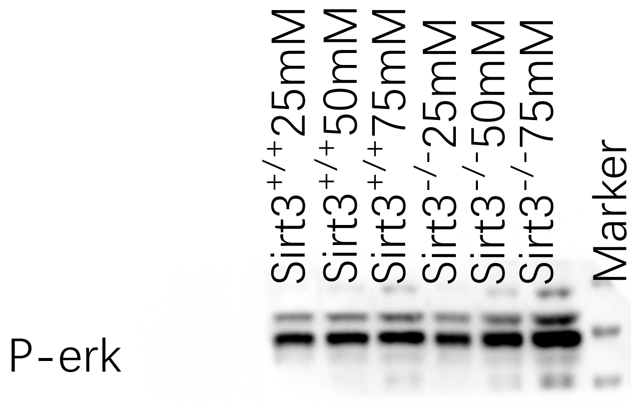

Sirt3+/+25mM
Sirt3+/+50mM
Sirt3+/+75mM
Sirt3-/-25mM
Sirt3-/-50mM
Sirt3-/-75mM
Marker
P-erk

Fig.4c

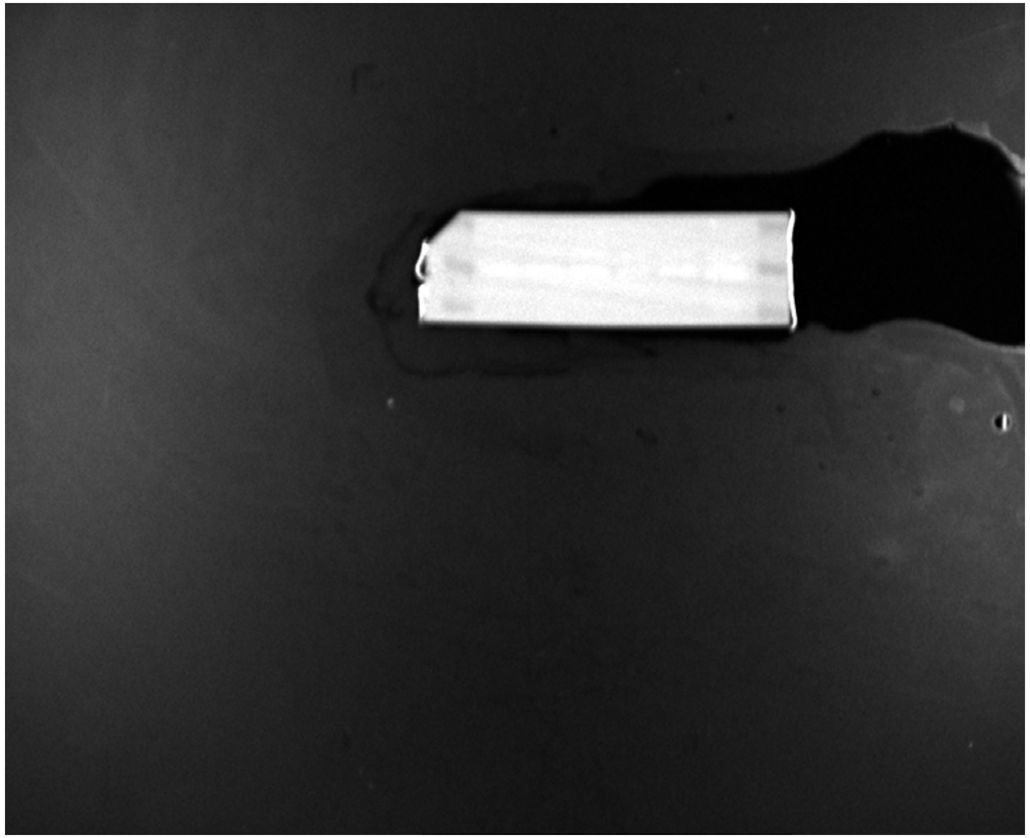

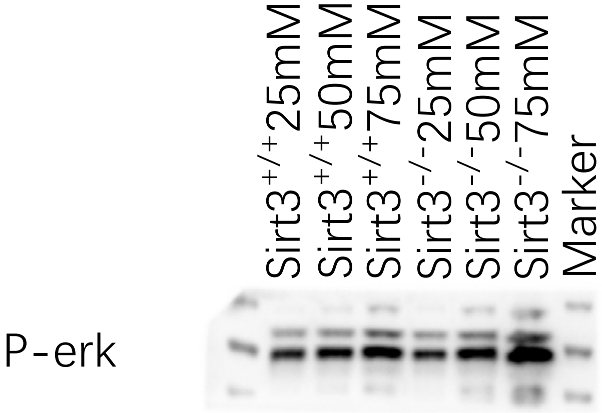
$Sirt3^{+/+}$25mM
$Sirt3^{+/+}$50mM
$Sirt3^{+/+}$75mM
$Sirt3^{-/-}$25mM
$Sirt3^{-/-}$50mM
$Sirt3^{-/-}$75mM
Marker
P-erk

Fig.4c

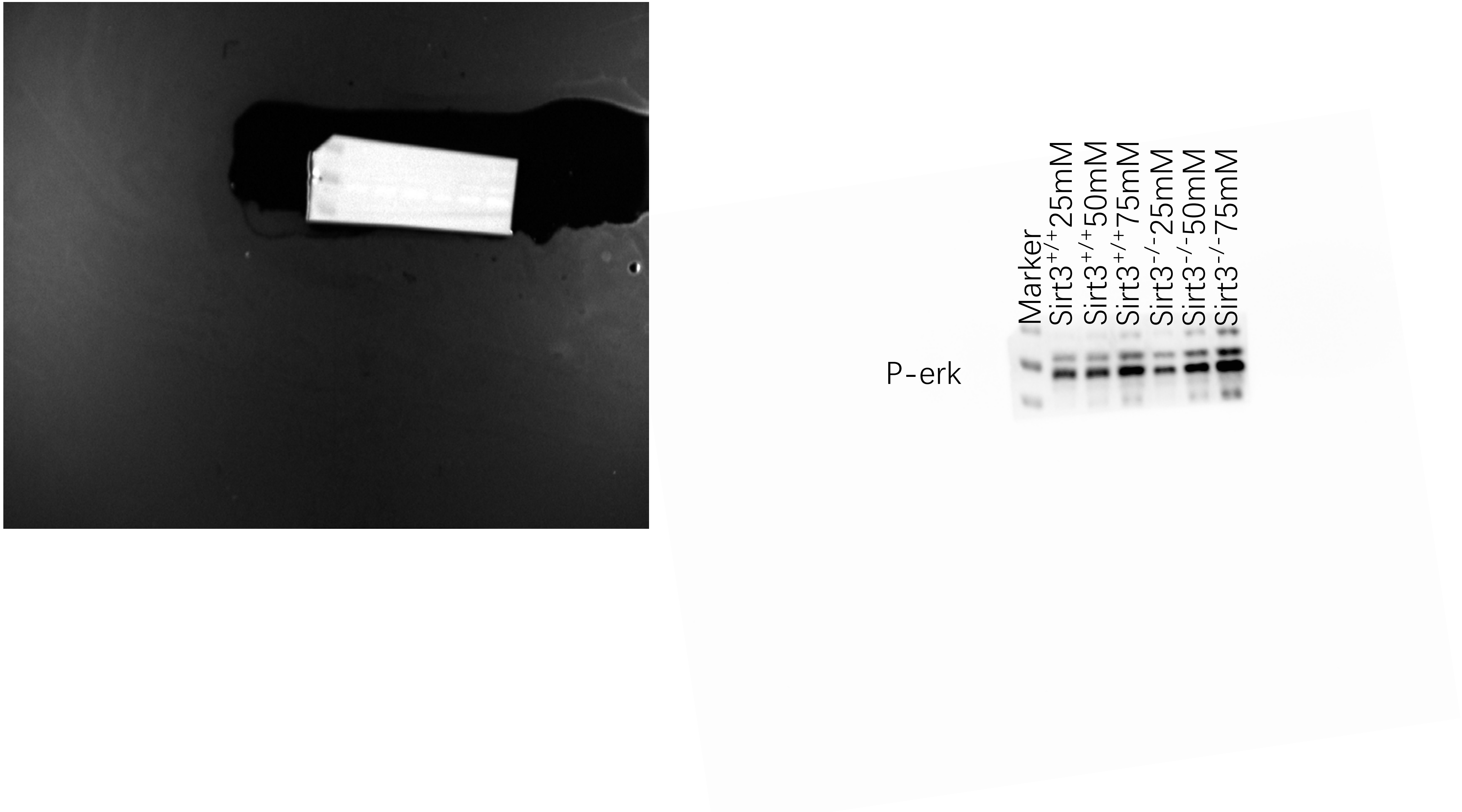
Marker
Sirt3$^{+/+}$25mM
Sirt3$^{+/+}$50mM
Sirt3$^{+/+}$75mM
Sirt3$^{-/-}$25mM
Sirt3$^{-/-}$50mM
Sirt3$^{-/-}$75mM
P-erk

Fig.4c

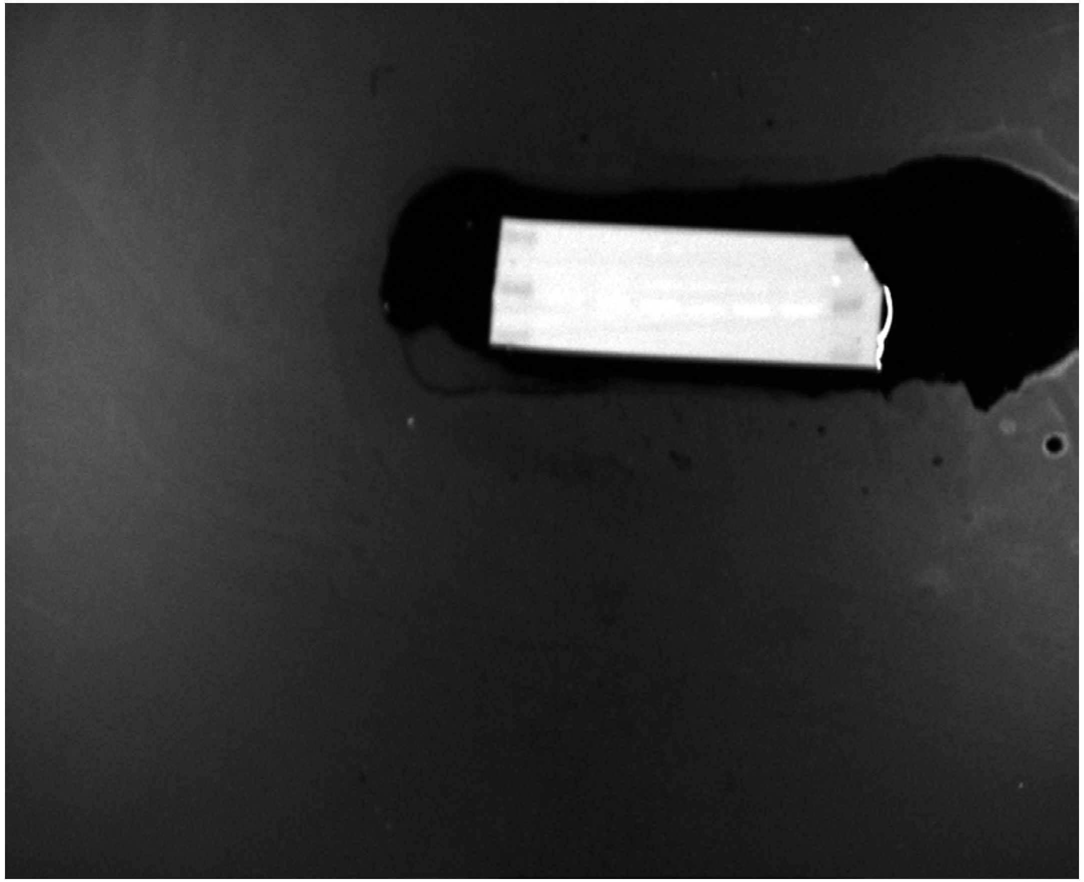

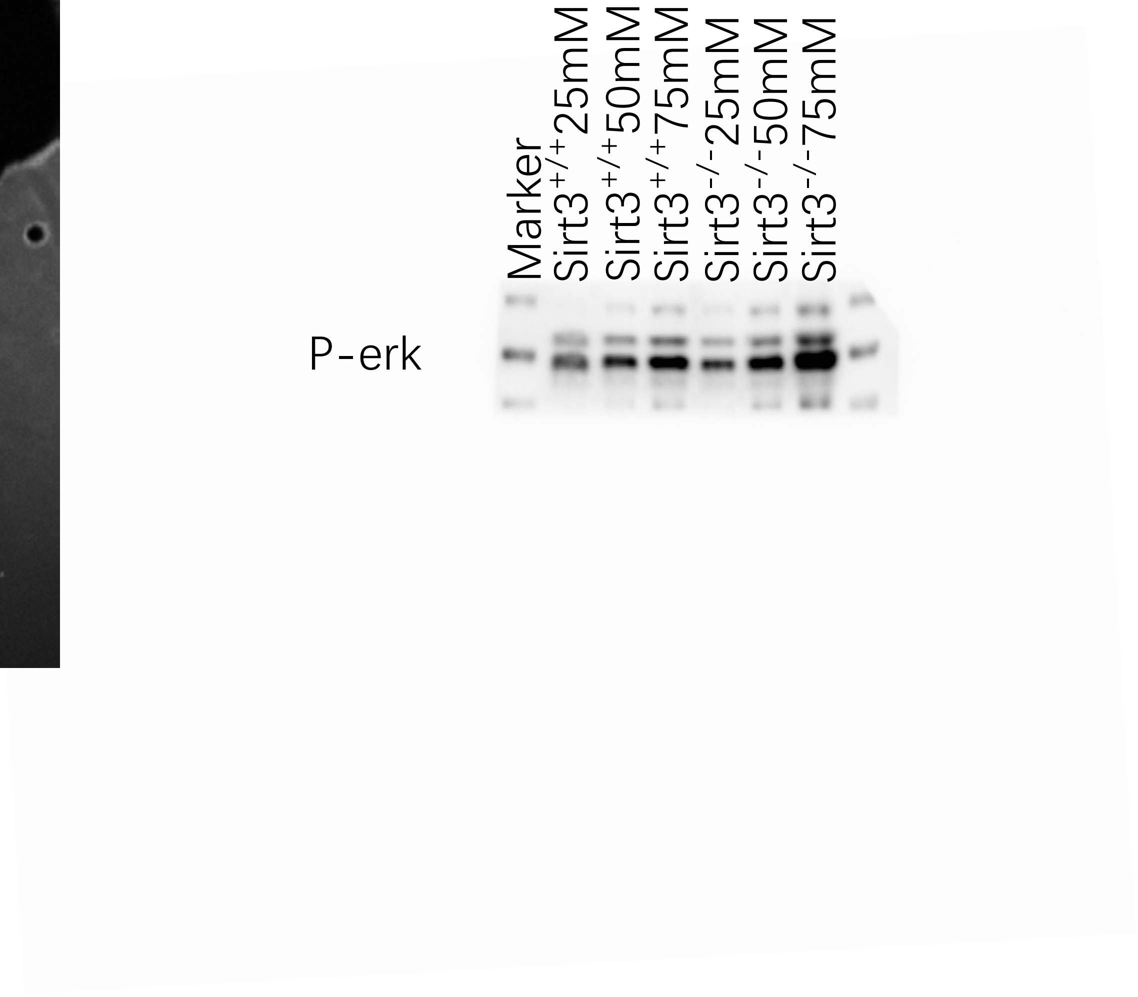
Marker
Sirt3+/+25mM
Sirt3+/+50mM
Sirt3+/+75mM
Sirt3-/-25mM
Sirt3-/-50mM
Sirt3-/-75mM
P-erk

Fig.4c

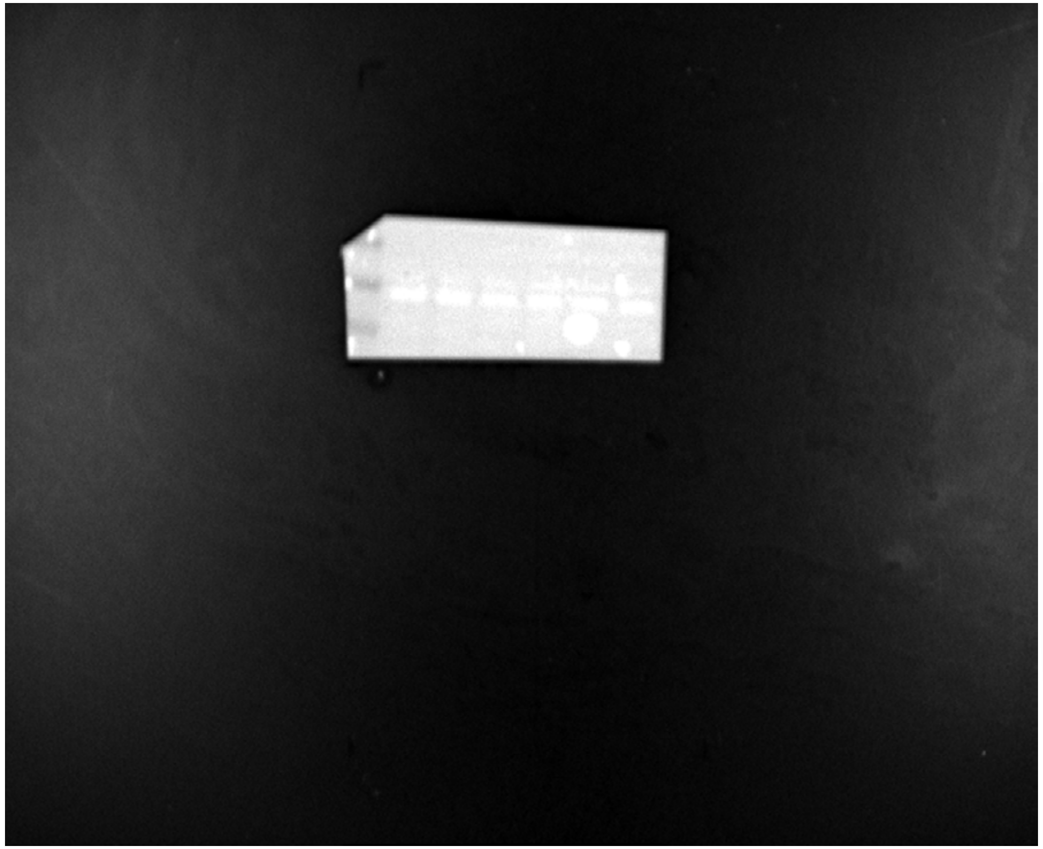

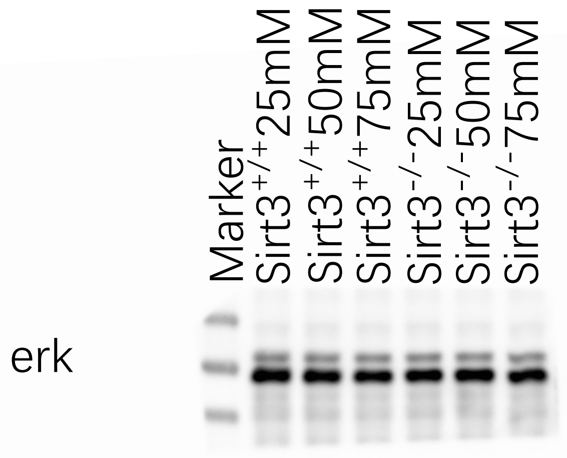
Marker
Sirt3+/+25mM
Sirt3+/+50mM
Sirt3+/+75mM
Sirt3-/-25mM
Sirt3-/-50mM
Sirt3-/-75mM
erk

Fig.4c

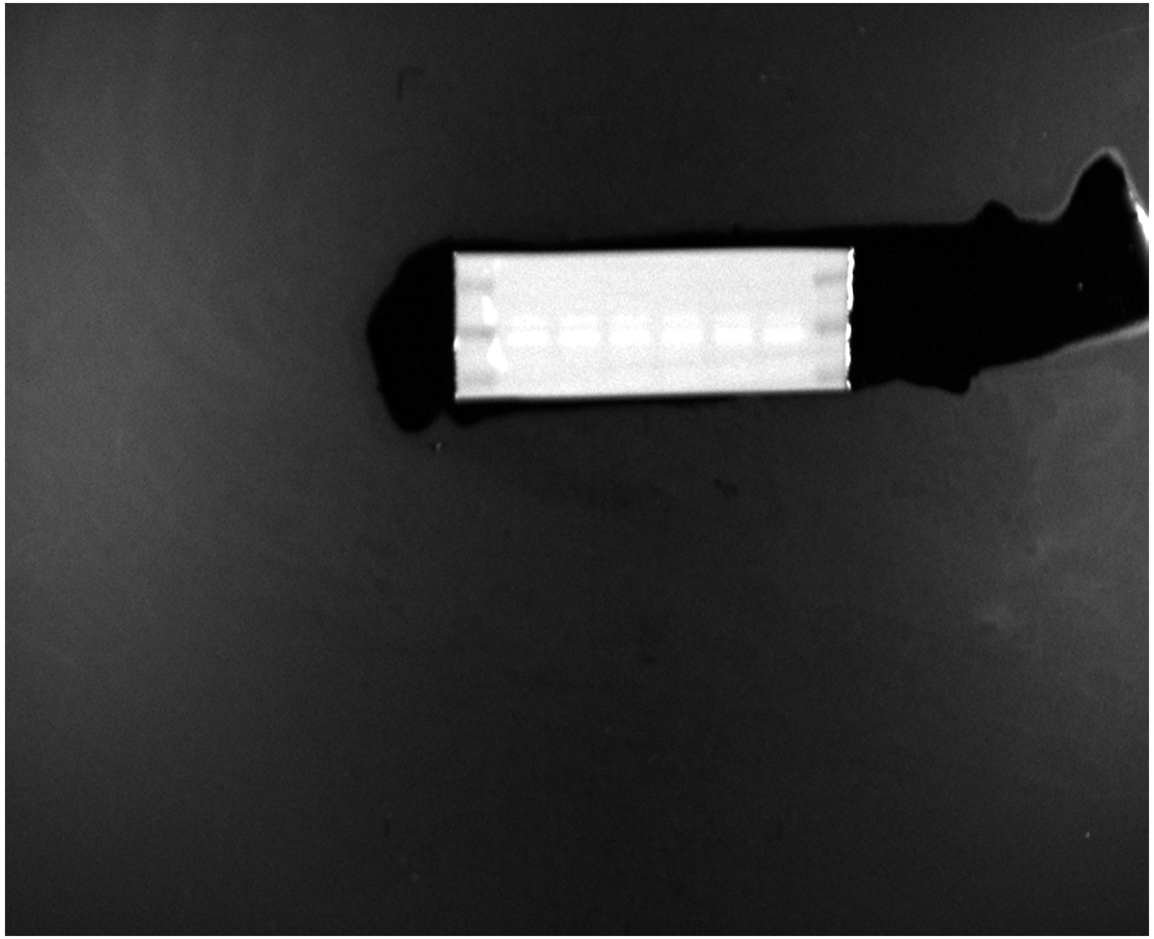

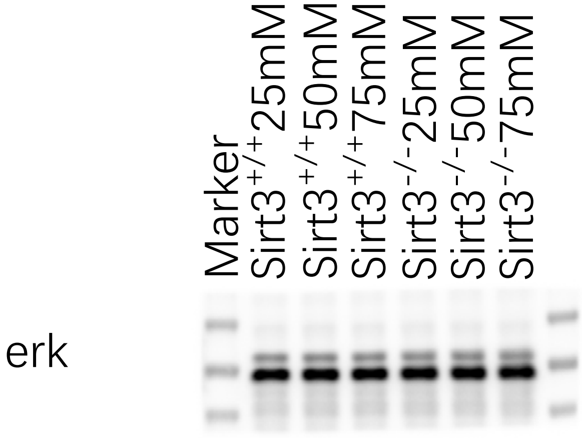
erk
Marker
Sirt3+/+25mM
Sirt3+/+50mM
Sirt3+/+75mM
Sirt3-/-25mM
Sirt3-/-50mM
Sirt3-/-75mM

Fig.4c

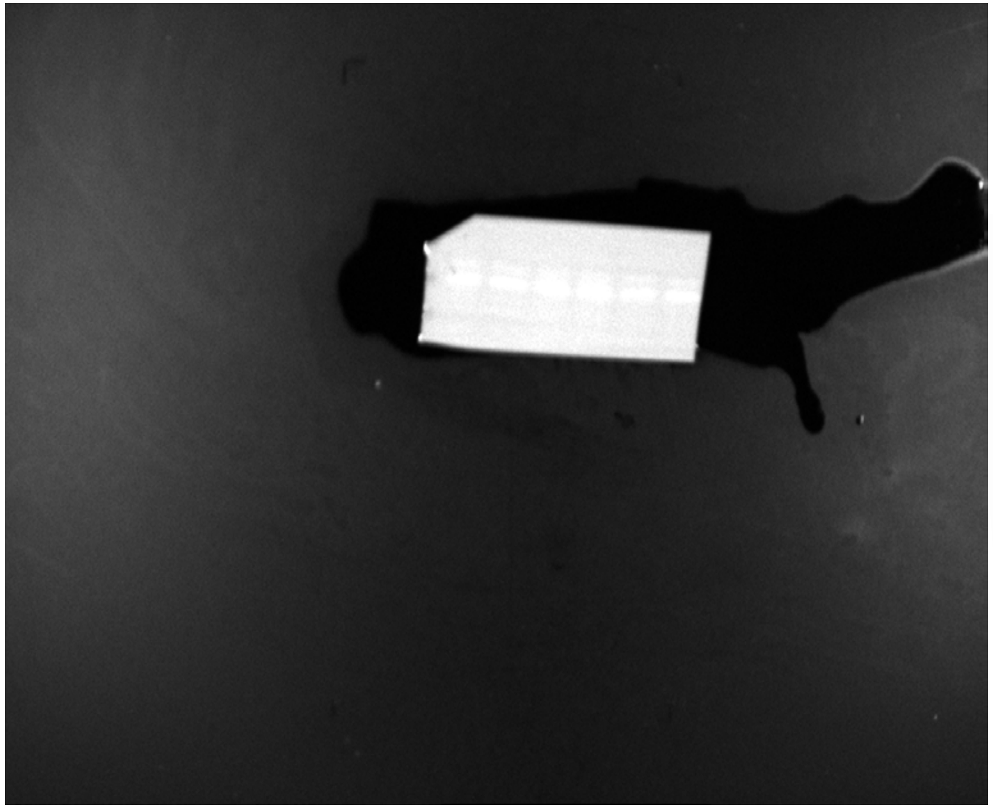

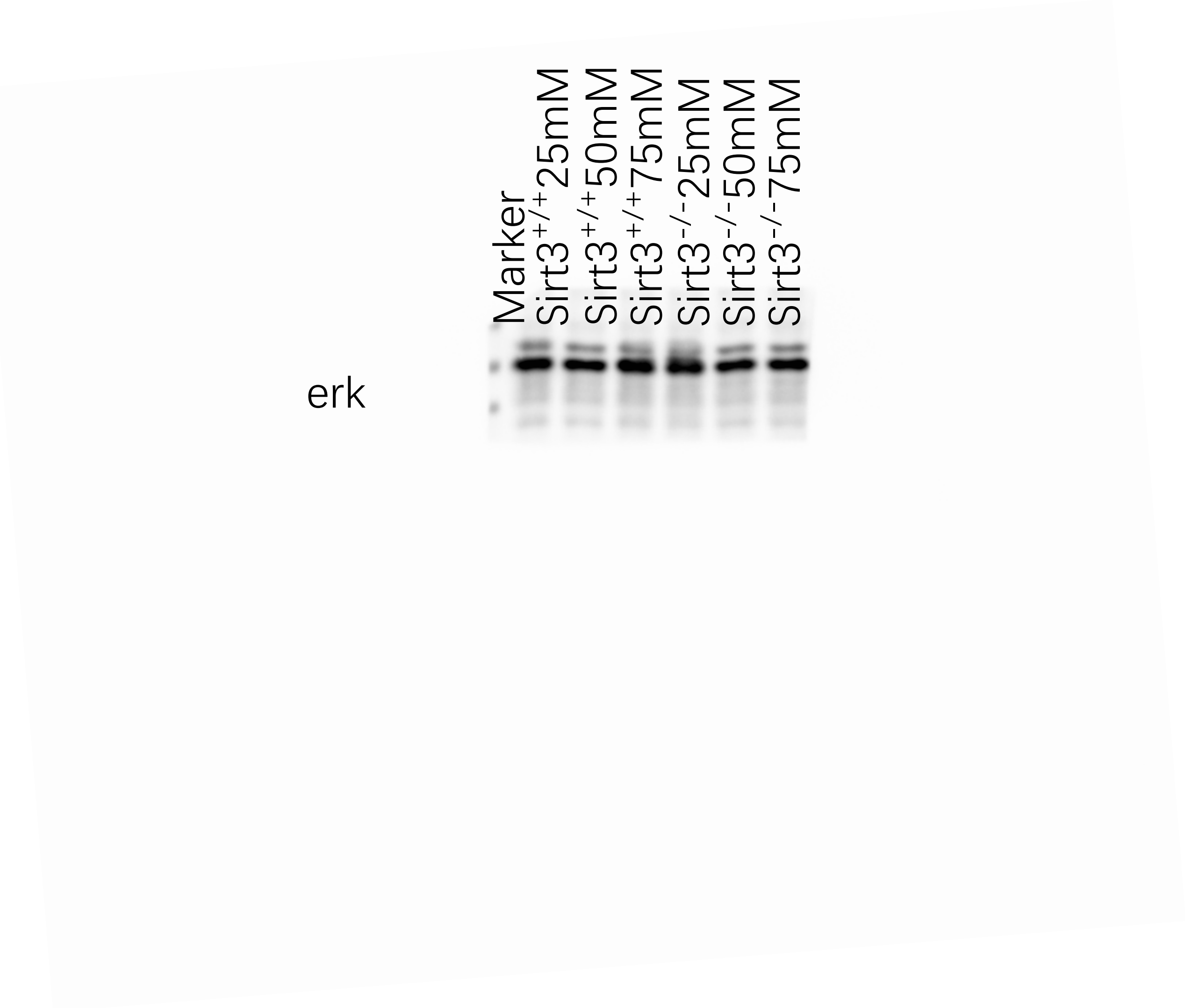
Marker
Sirt3+/+25mM
Sirt3+/+50mM
Sirt3+/+75mM
Sirt3-/-25mM
Sirt3-/-50mM
Sirt3-/-75mM
erk

Fig.4c

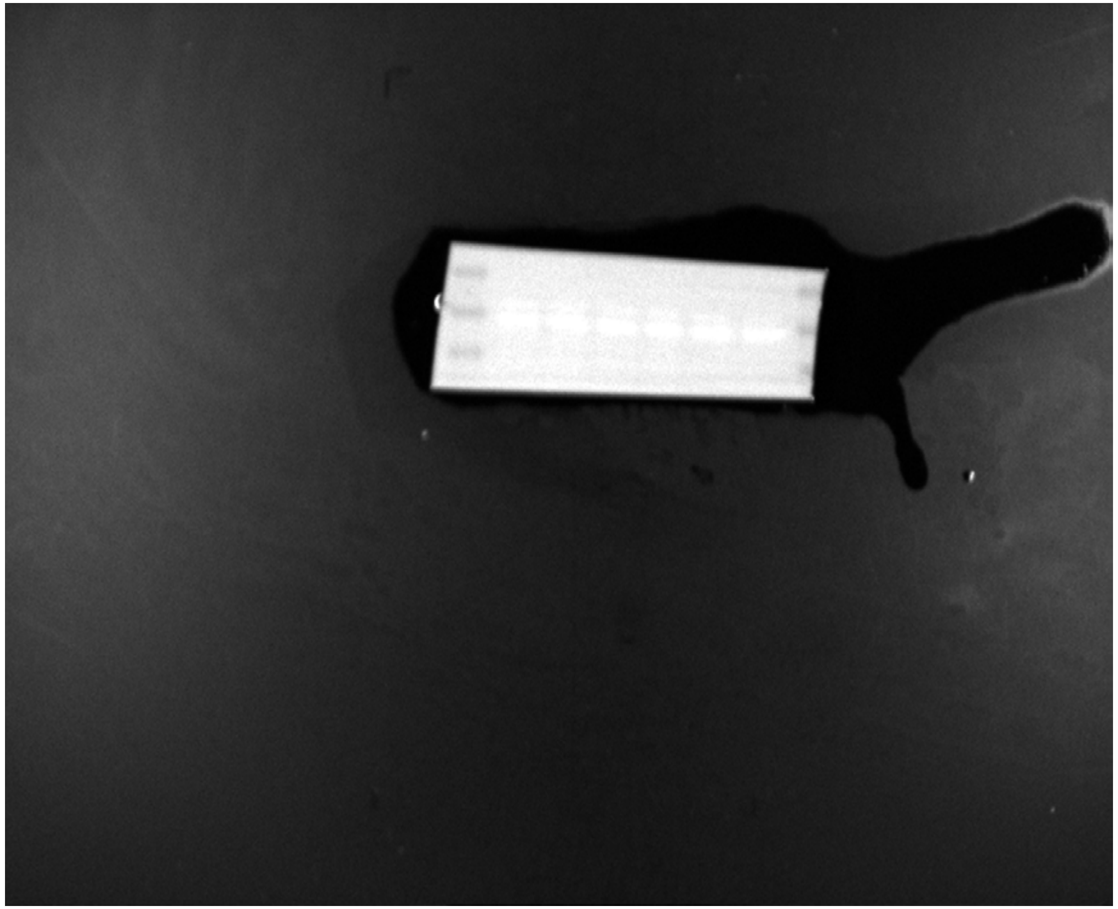

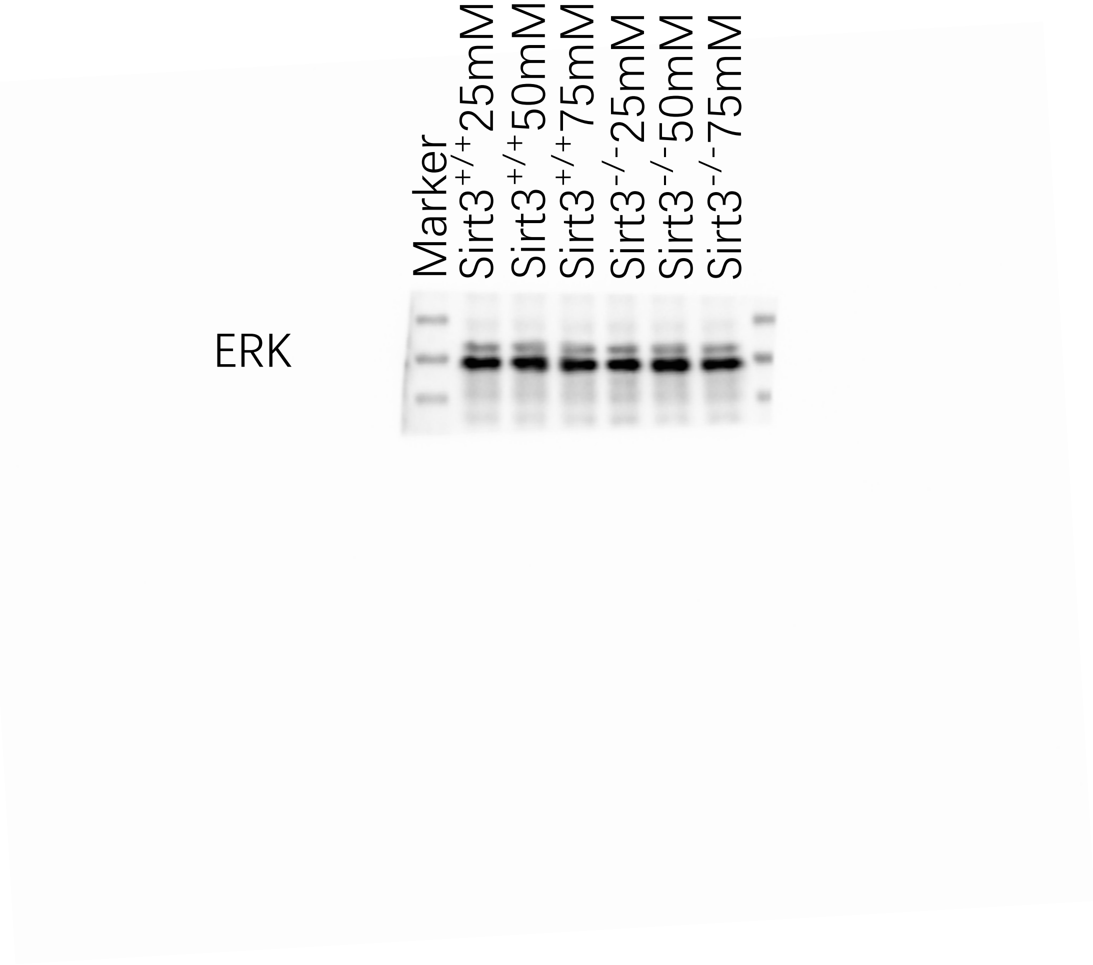
Marker
Sirt3+/+25mM
Sirt3+/+50mM
Sirt3+/+75mM
Sirt3-/-25mM
Sirt3-/-50mM
Sirt3-/-75mM
ERK

Fig.4c

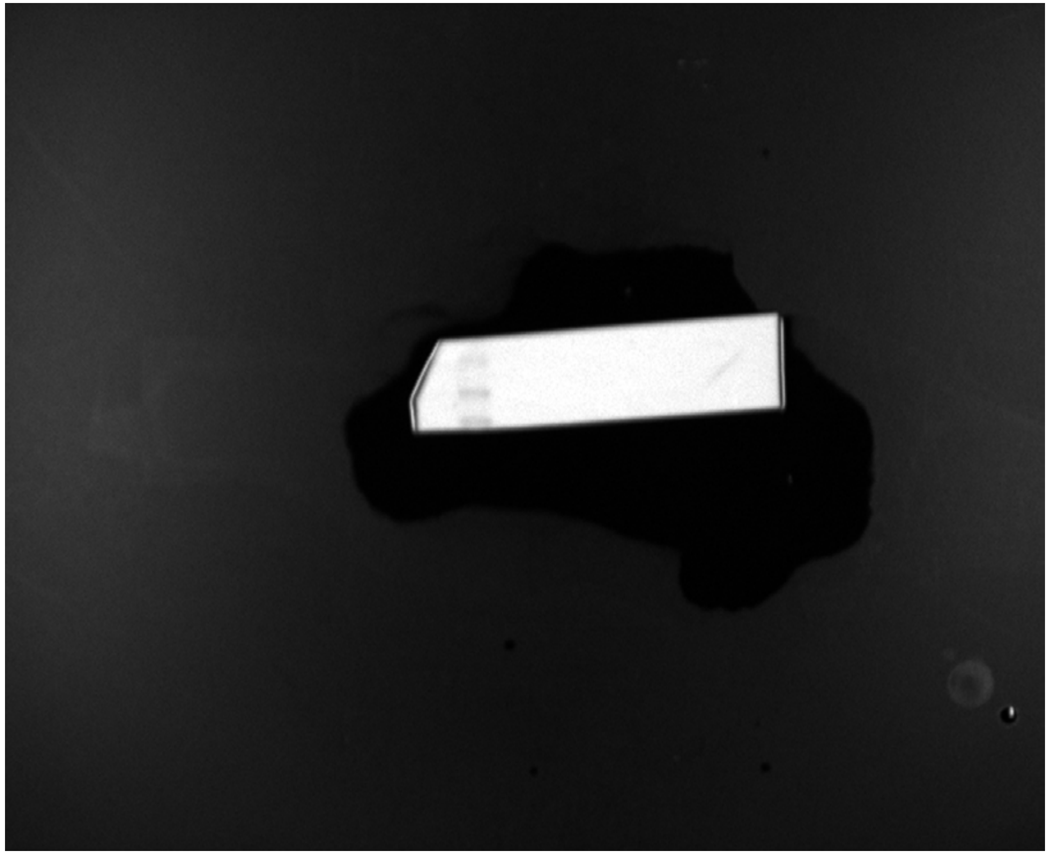

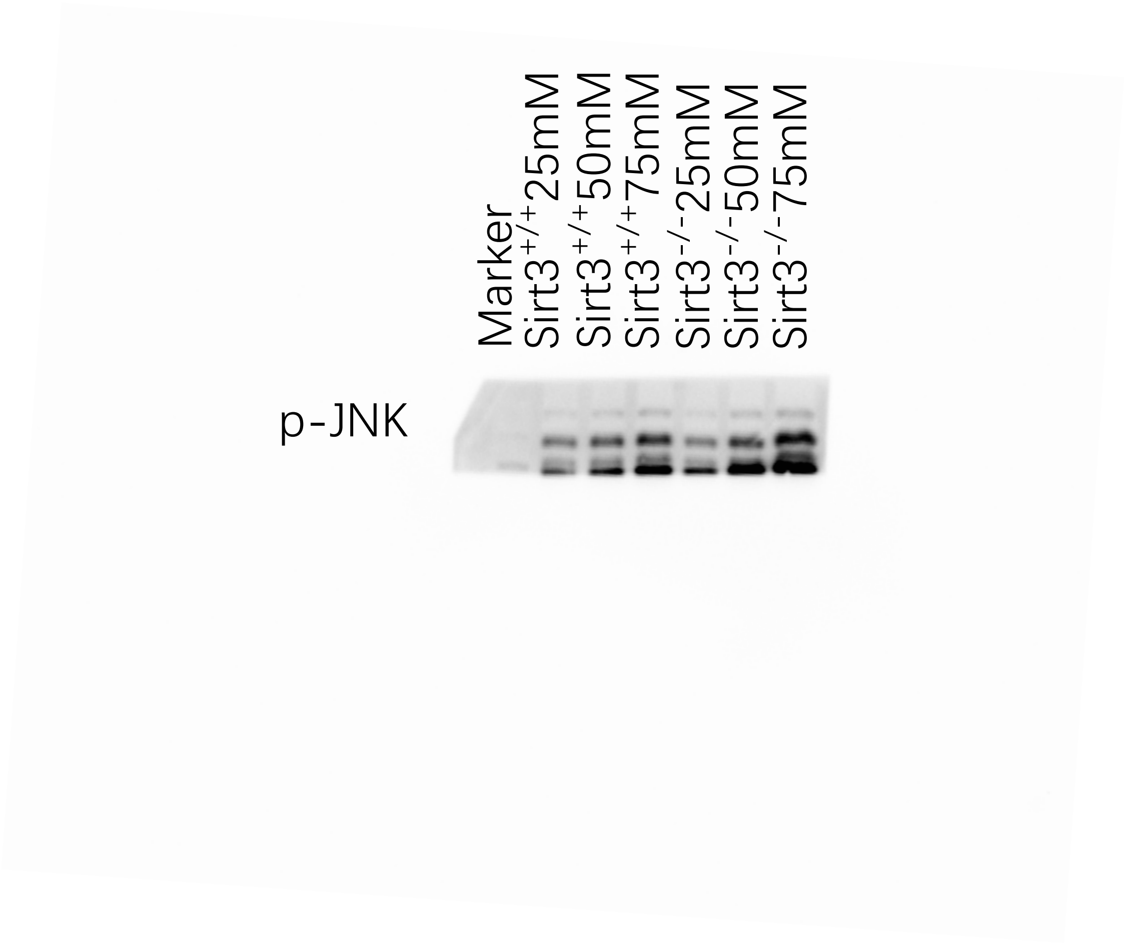
Marker
Sirt3+/+25mM
Sirt3+/+50mM
Sirt3+/+75mM
Sirt3-/-25mM
Sirt3-/-50mM
Sirt3-/-75mM
p-JNK

Fig.4c

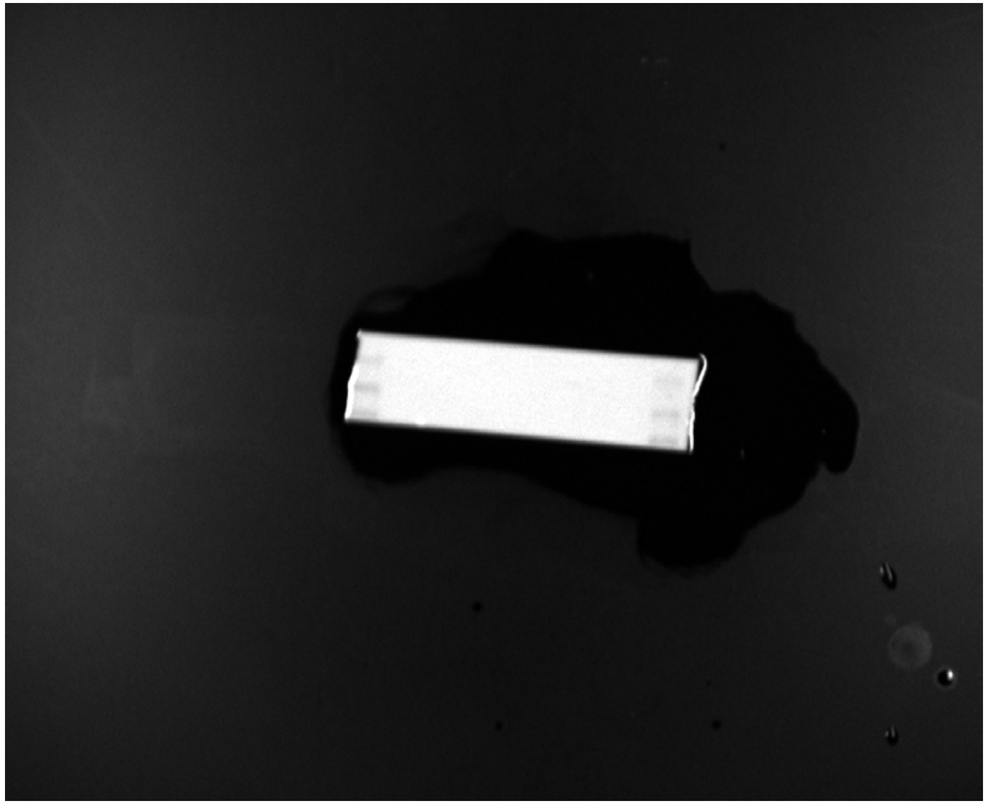

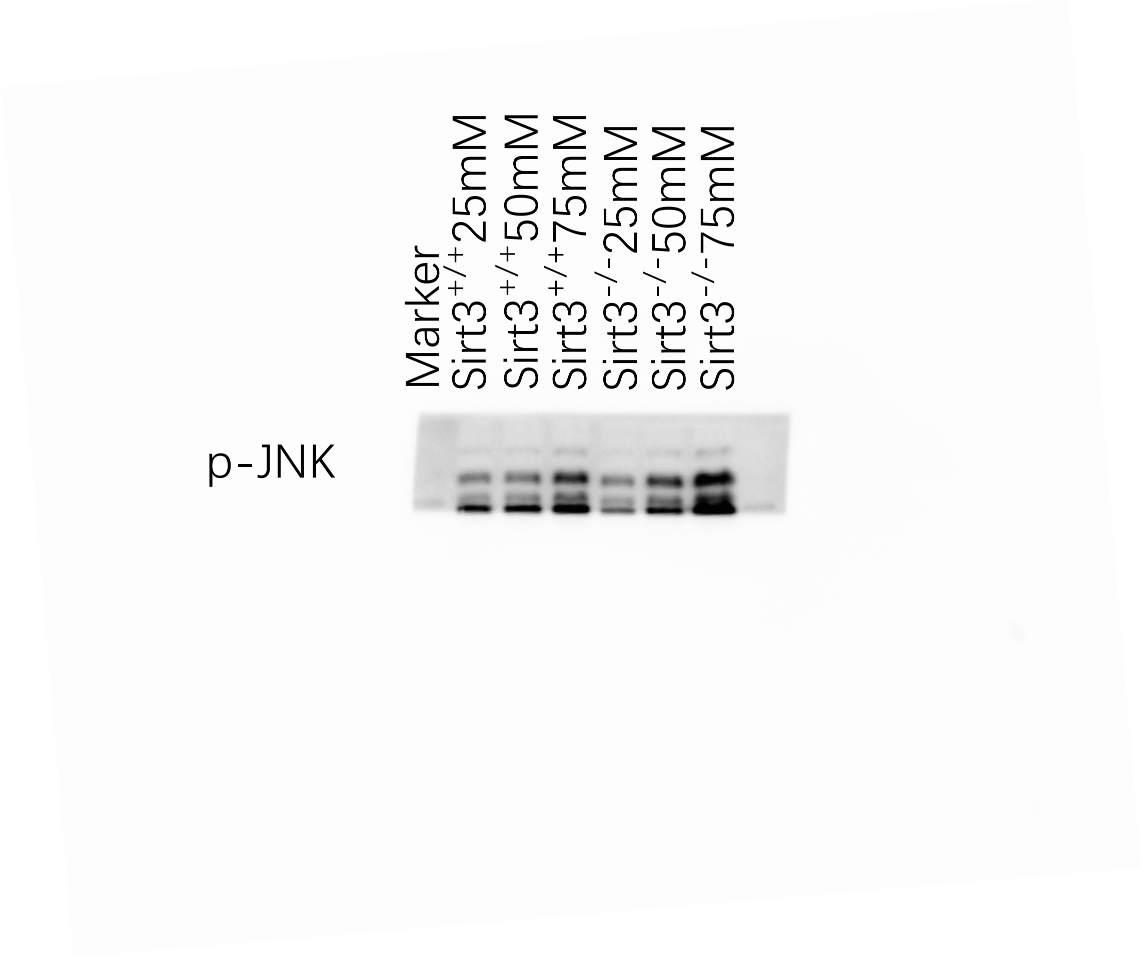
Marker
Sirt3+/+25mM
Sirt3+/+50mM
Sirt3+/+75mM
Sirt3-/-25mM
Sirt3-/-50mM
Sirt3-/-75mM
p-JNK

Fig.4c

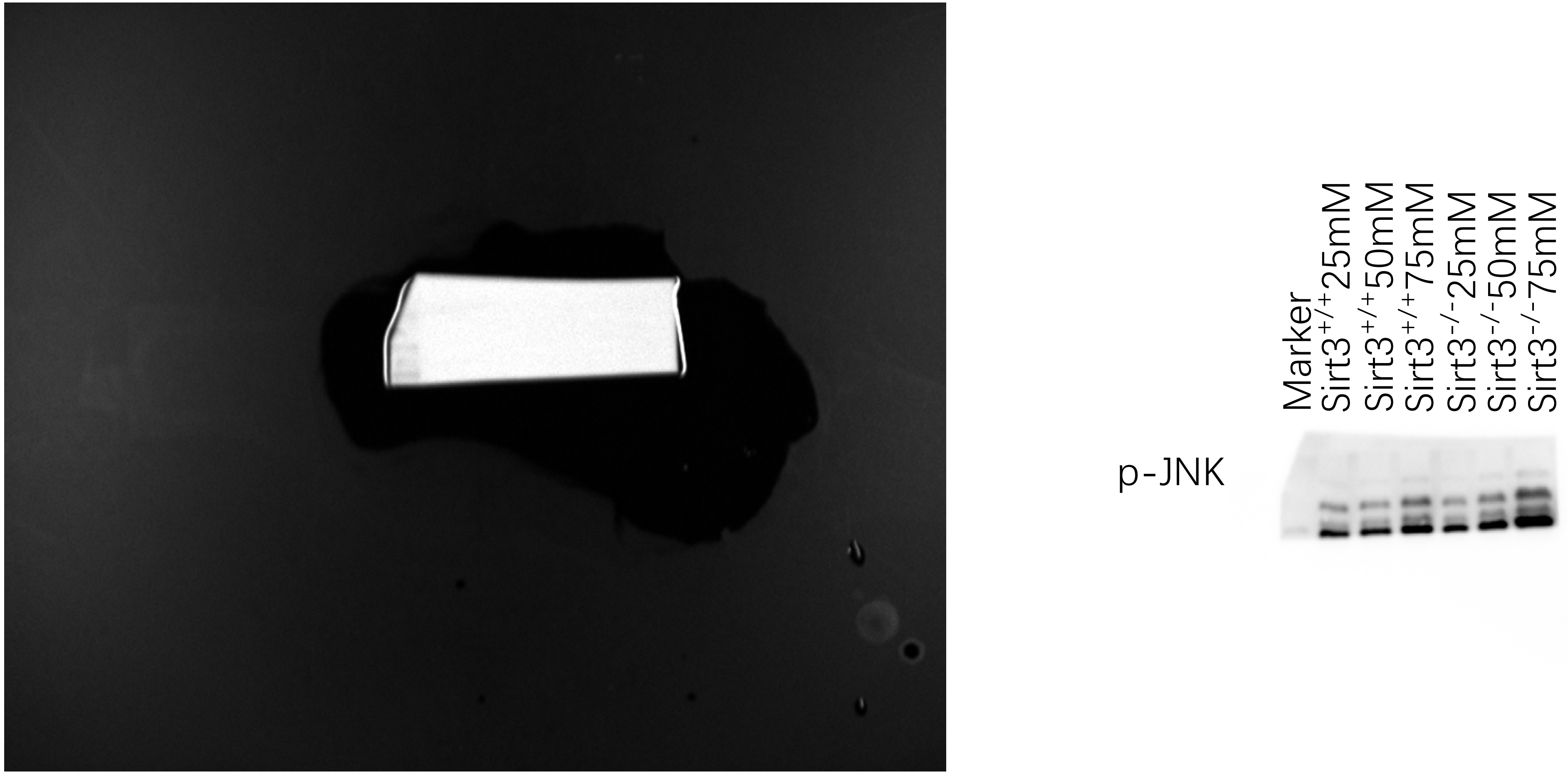
Marker
Sirt3$^{+/+}$25mM
Sirt3$^{+/+}$50mM
Sirt3$^{+/+}$75mM
Sirt3$^{-/-}$25mM
Sirt3$^{-/-}$50mM
Sirt3$^{-/-}$75mM
p-JNK

Fig.4c

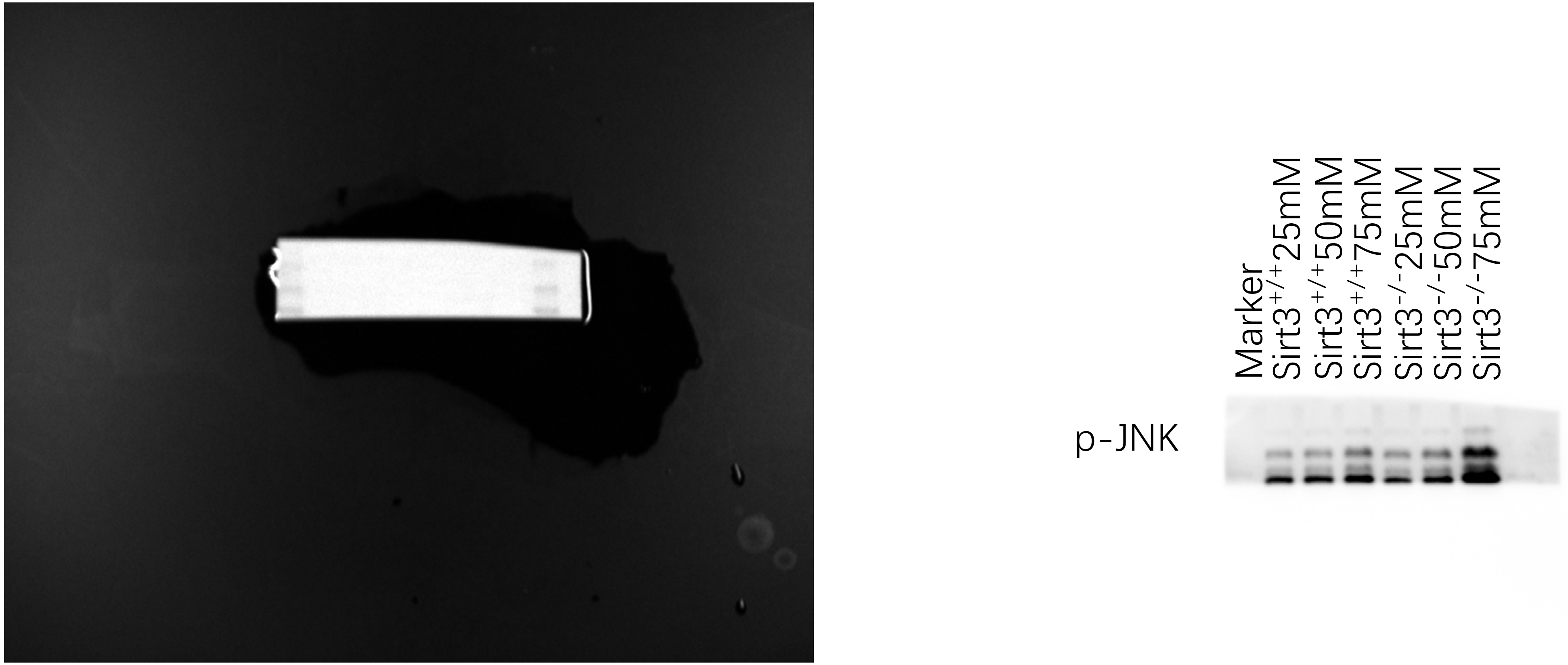
Marker
Sirt3+/+25mM
Sirt3+/+50mM
Sirt3+/+75mM
Sirt3-/-25mM
Sirt3-/-50mM
Sirt3-/-75mM
p-JNK

Fig.4c

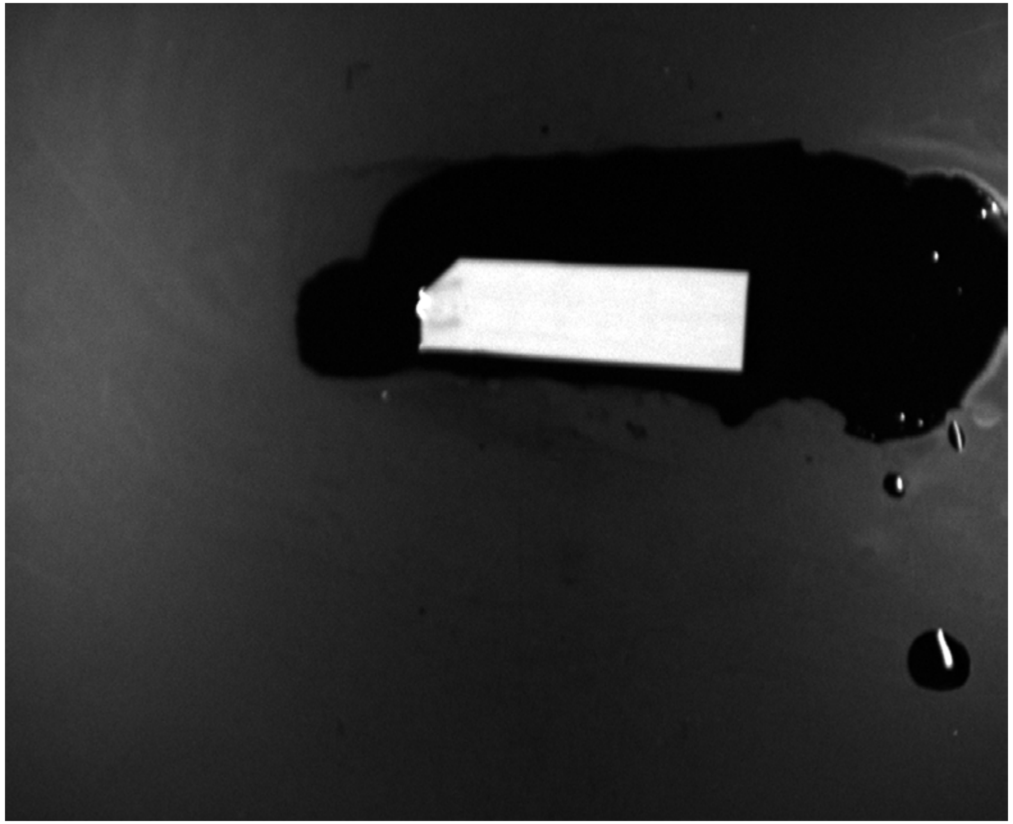

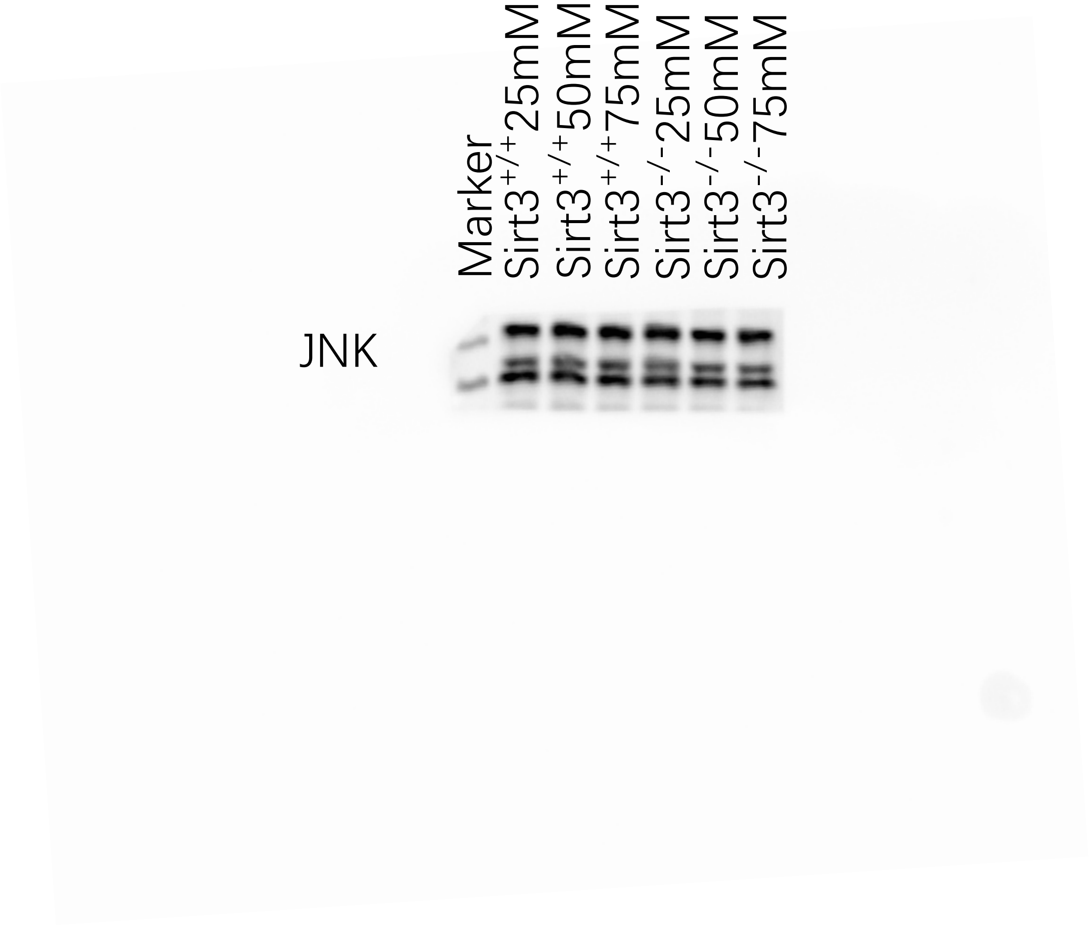
Marker
Sirt3$^{+/+}$25mM
Sirt3$^{+/+}$50mM
Sirt3$^{+/+}$75mM
Sirt3$^{-/-}$25mM
Sirt3$^{-/-}$50mM
Sirt3$^{-/-}$75mM
JNK

Fig.4c

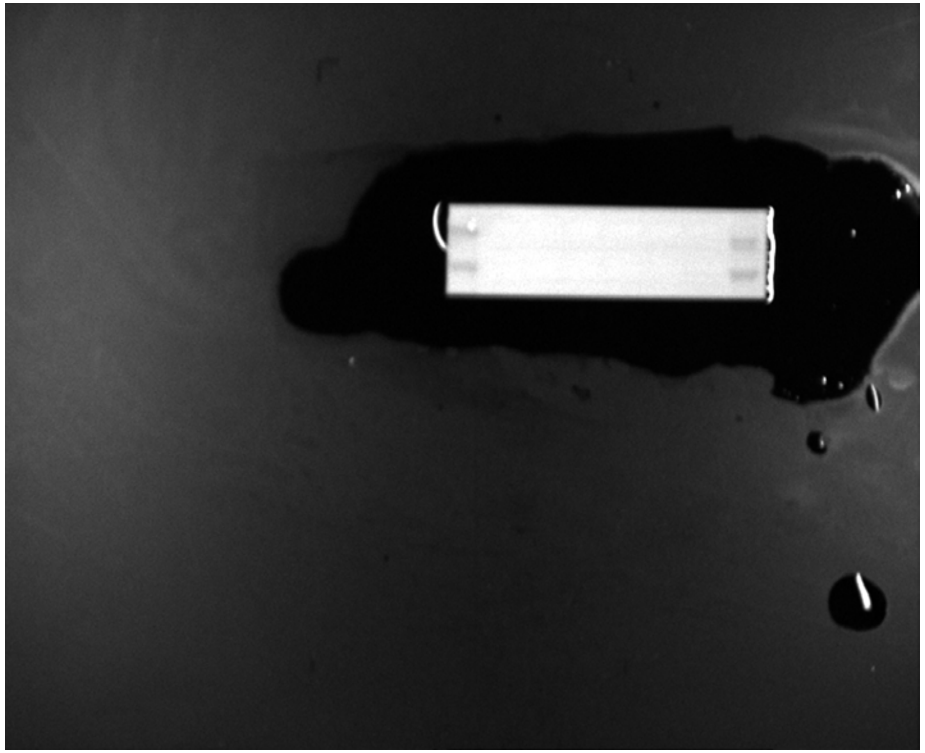

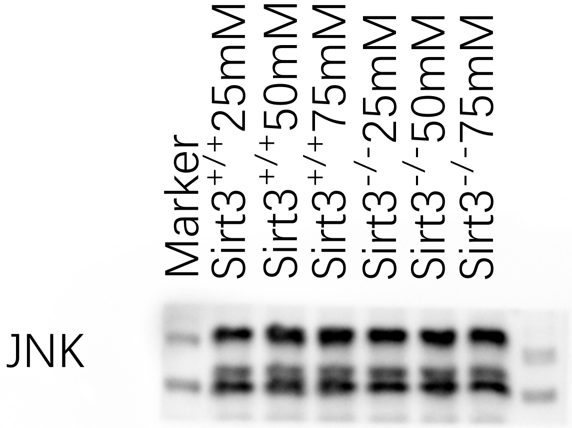

Marker
Sirt3+/+25mM
Sirt3+/+50mM
Sirt3+/+75mM
Sirt3-/-25mM
Sirt3-/-50mM
Sirt3-/-75mM
JNK

Fig.4c

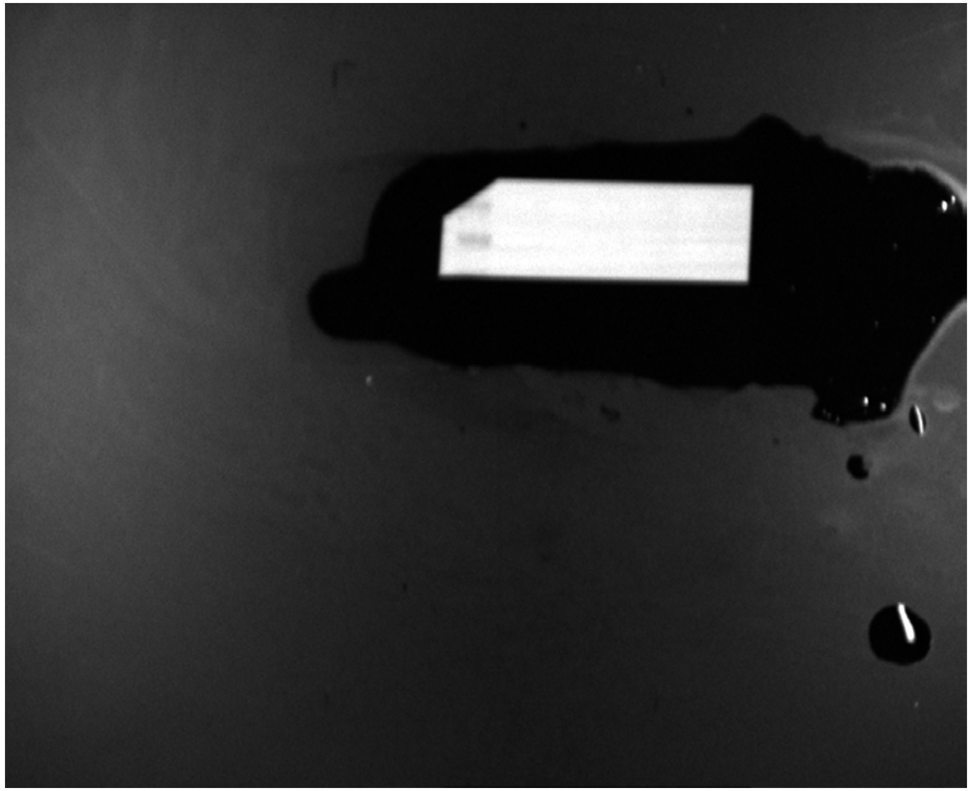

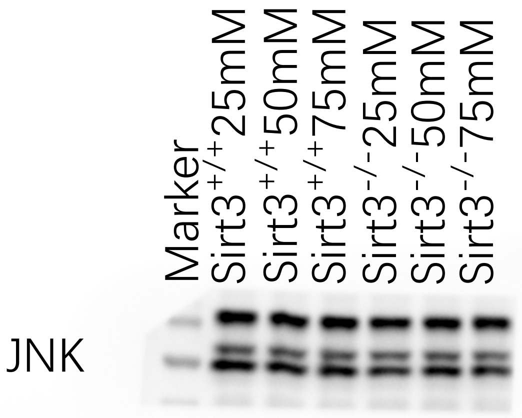
Marker
Sirt3+/+25mM
Sirt3+/+50mM
Sirt3+/+75mM
Sirt3-/-25mM
Sirt3-/-50mM
Sirt3-/-75mM
JNK

Fig.4c

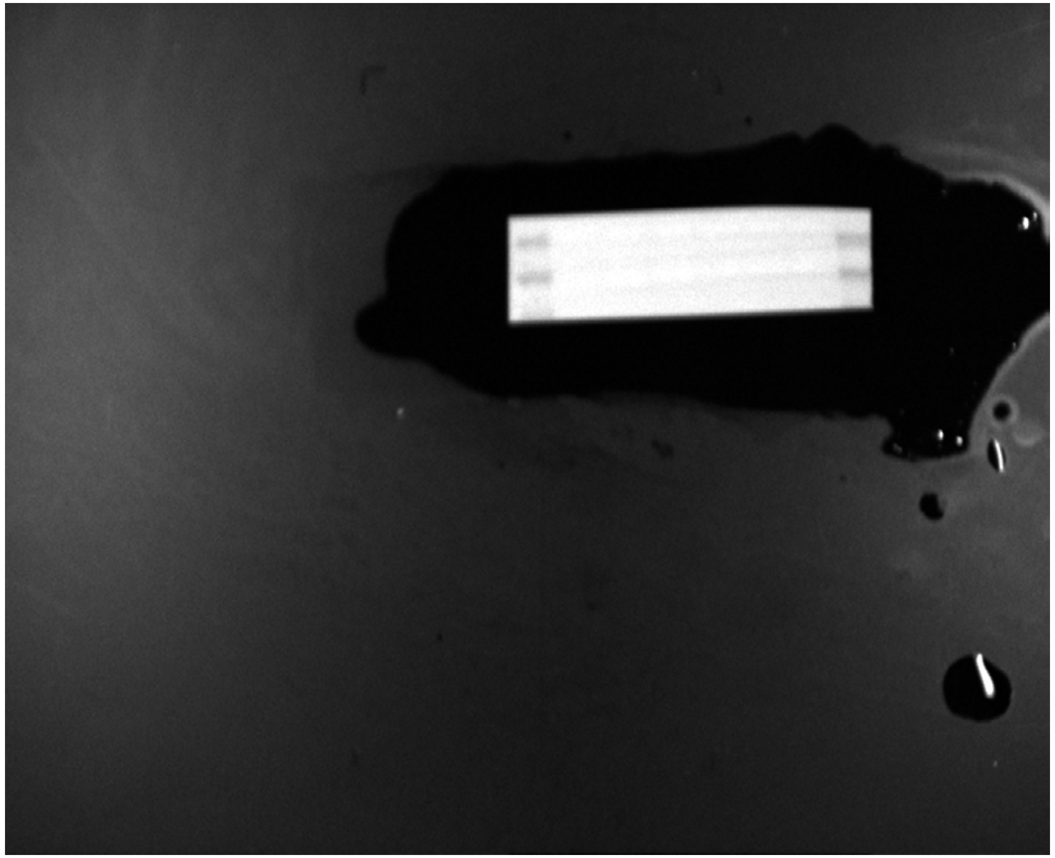

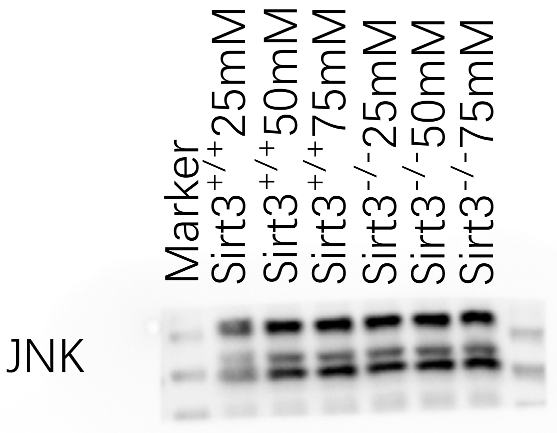
Marker
Sirt3+/+25mM
Sirt3+/+50mM
Sirt3+/+75mM
Sirt3-/-25mM
Sirt3-/-50mM
Sirt3-/-75mM
JNK

Fig.4c

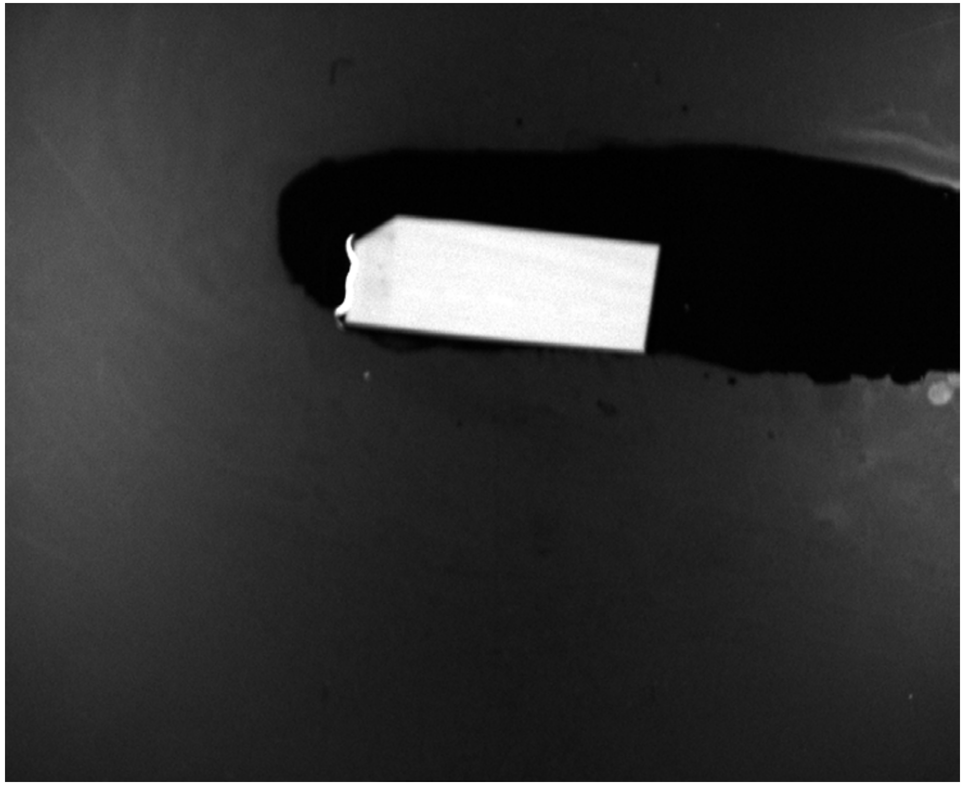

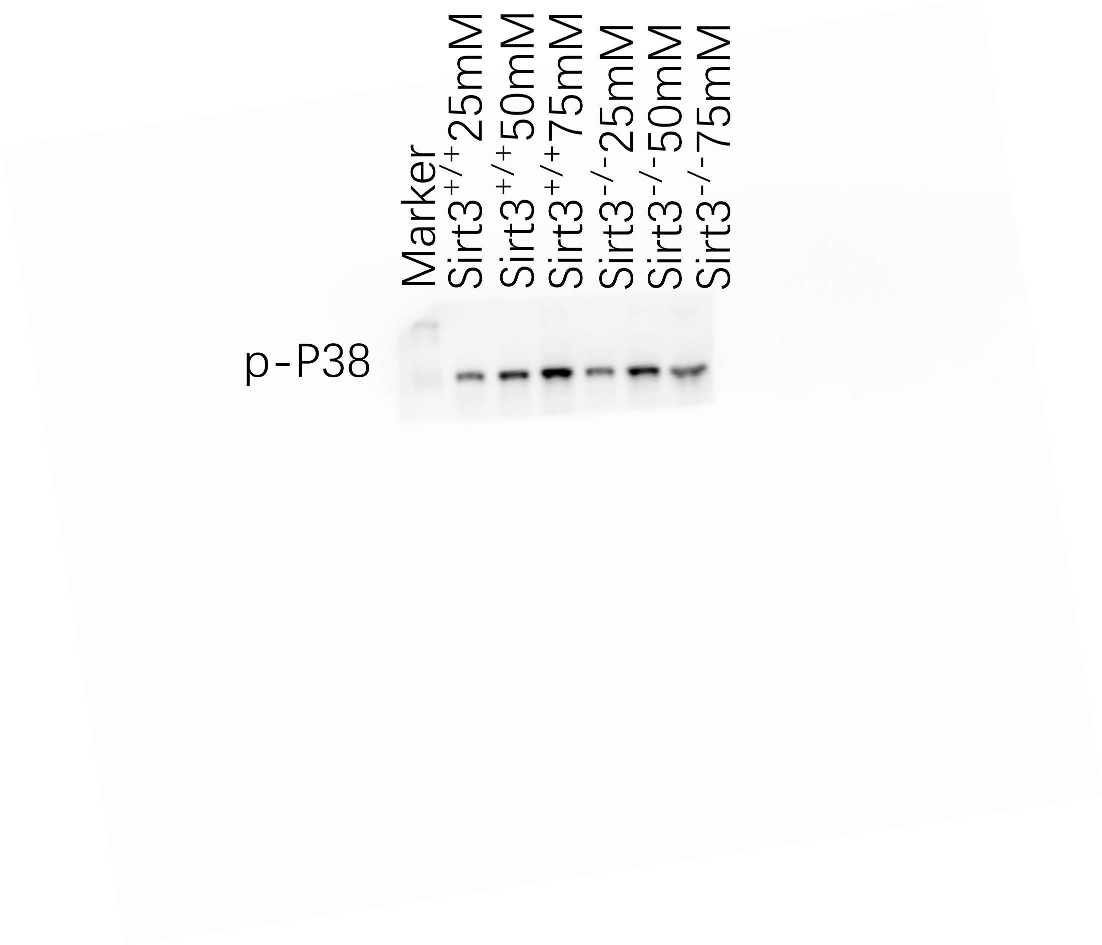
Marker
Sirt3+/+25mM
Sirt3+/+50mM
Sirt3+/+75mM
Sirt3-/-25mM
Sirt3-/-50mM
Sirt3-/-75mM
p-P38

Fig.4c

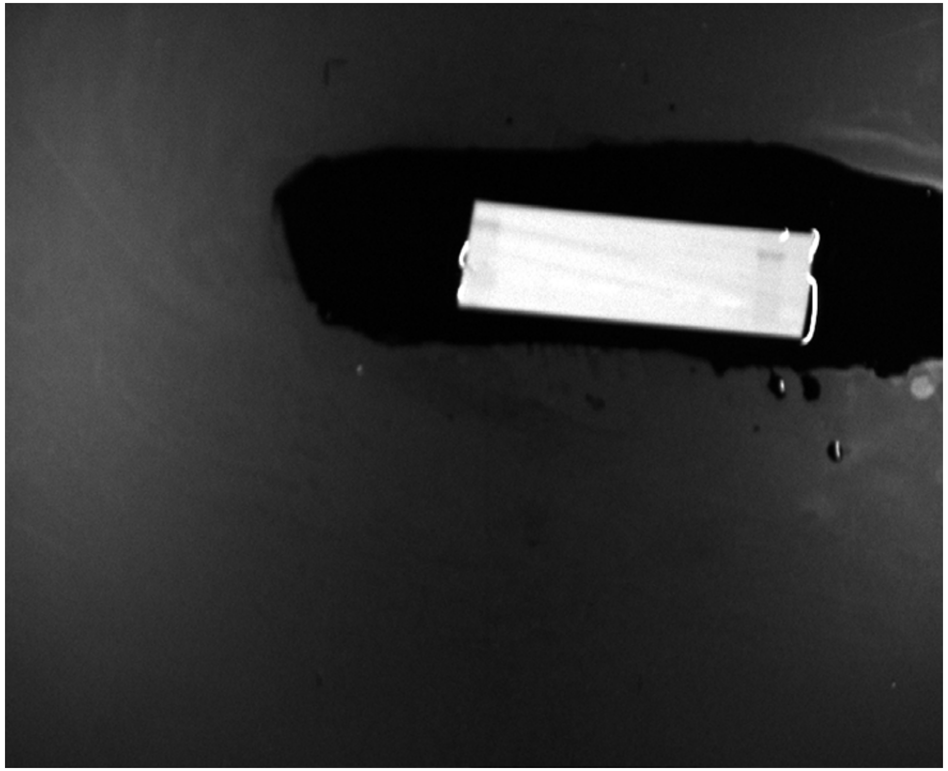

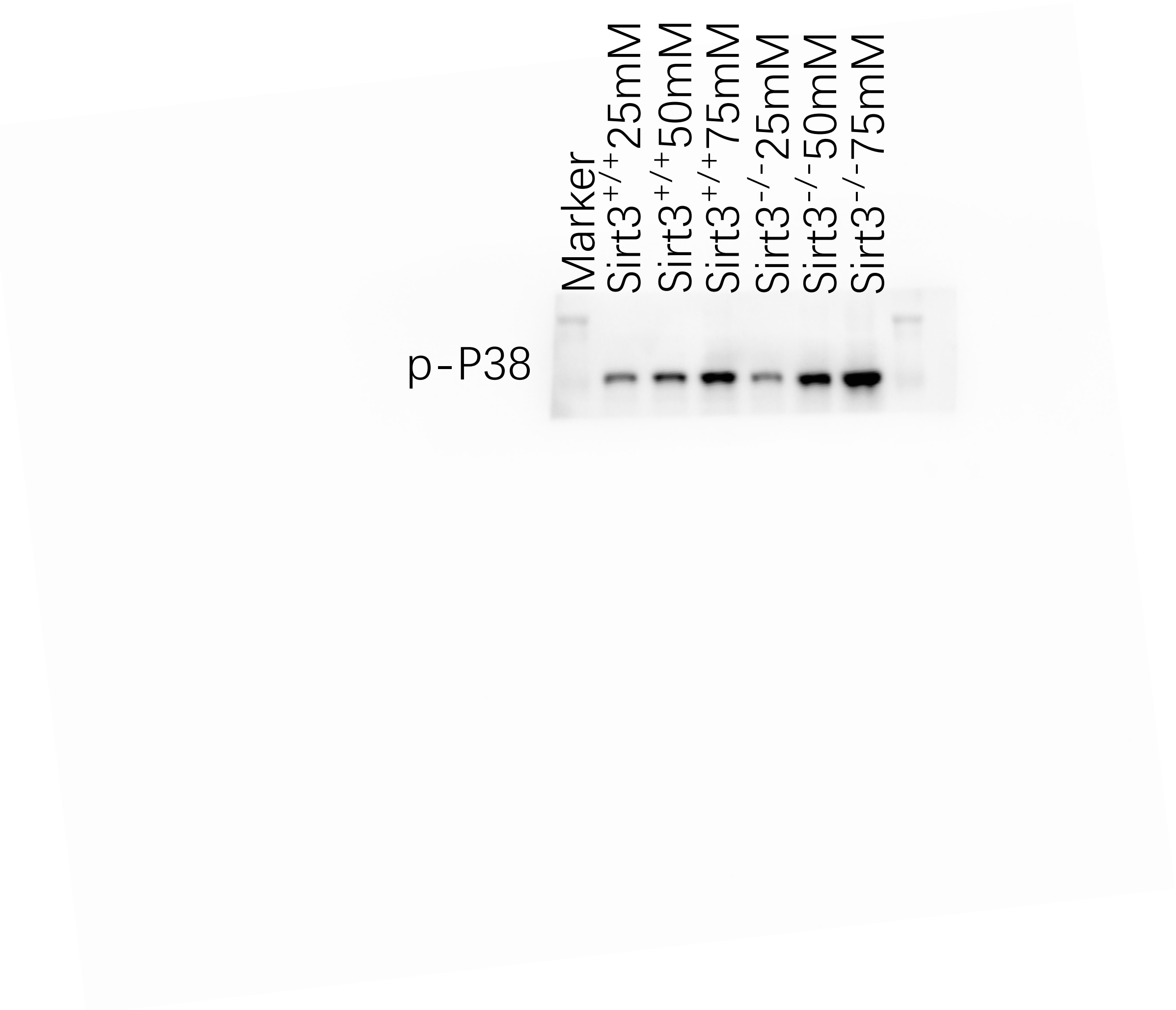
Marker
Sirt3+/+25mM
Sirt3+/+50mM
Sirt3+/+75mM
Sirt3-/-25mM
Sirt3-/-50mM
Sirt3-/-75mM
p-P38

Fig.4c

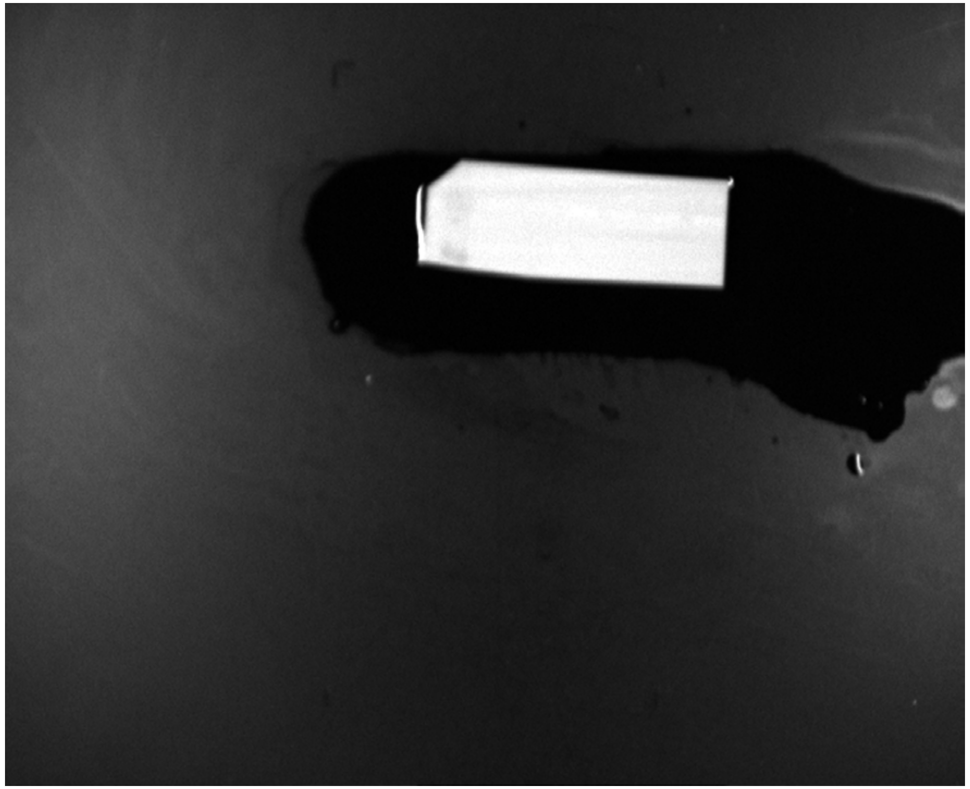

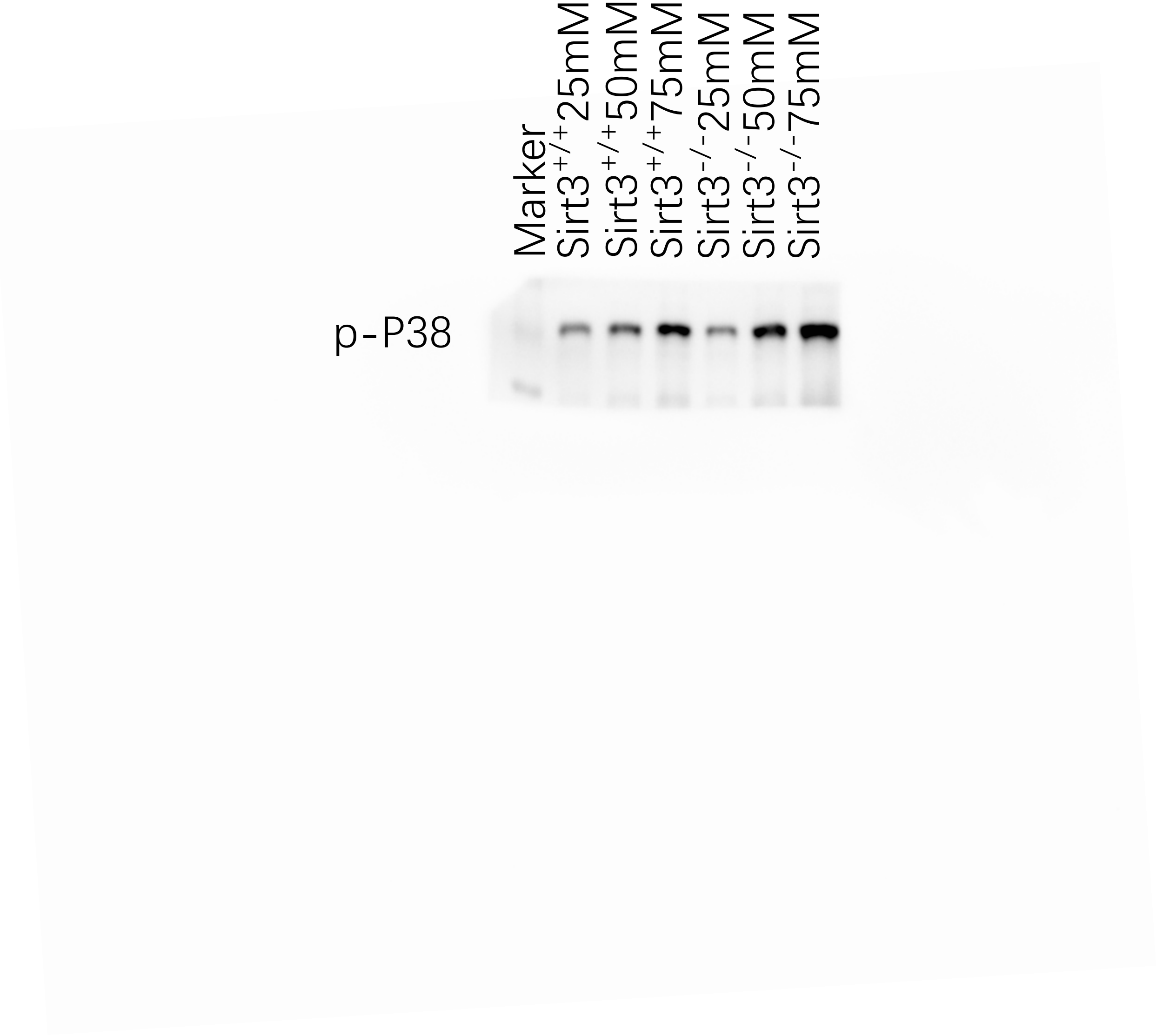
Marker
Sirt3+/+25mM
Sirt3+/+50mM
Sirt3+/+75mM
Sirt3-/-25mM
Sirt3-/-50mM
Sirt3-/-75mM
p-P38

Fig.4c

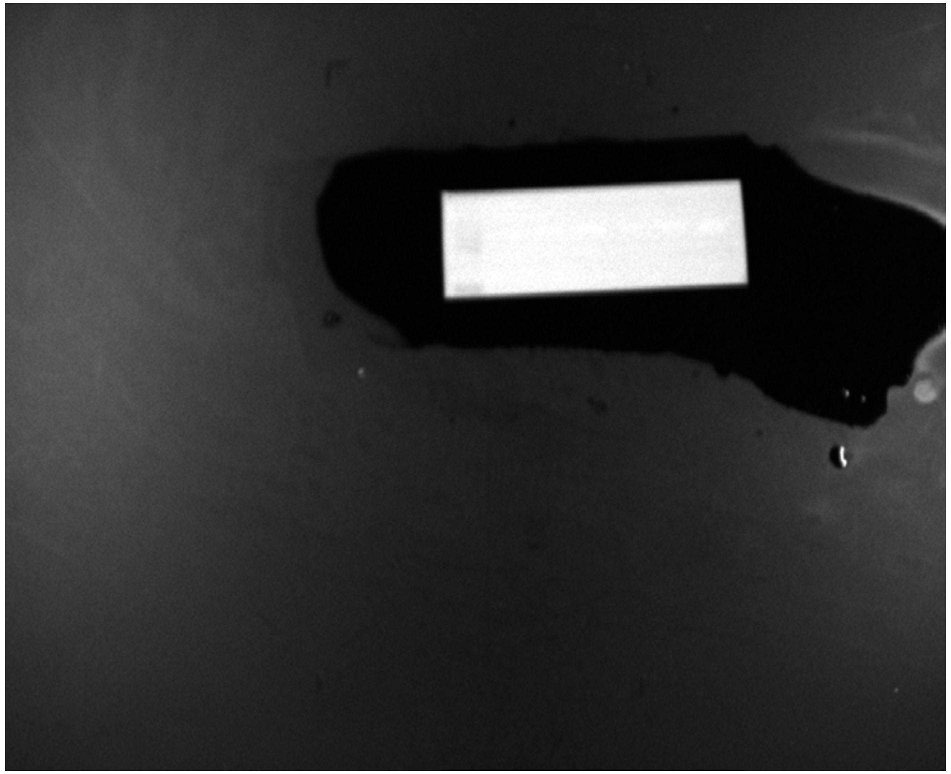

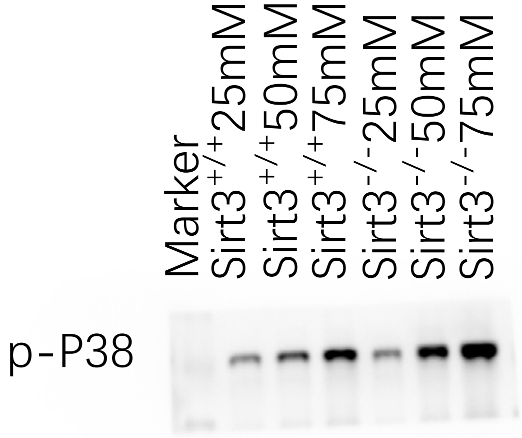
Marker
Sirt3+/+25mM
Sirt3+/+50mM
Sirt3+/+75mM
Sirt3-/-25mM
Sirt3-/-50mM
Sirt3-/-75mM
p-P38

Fig.4c

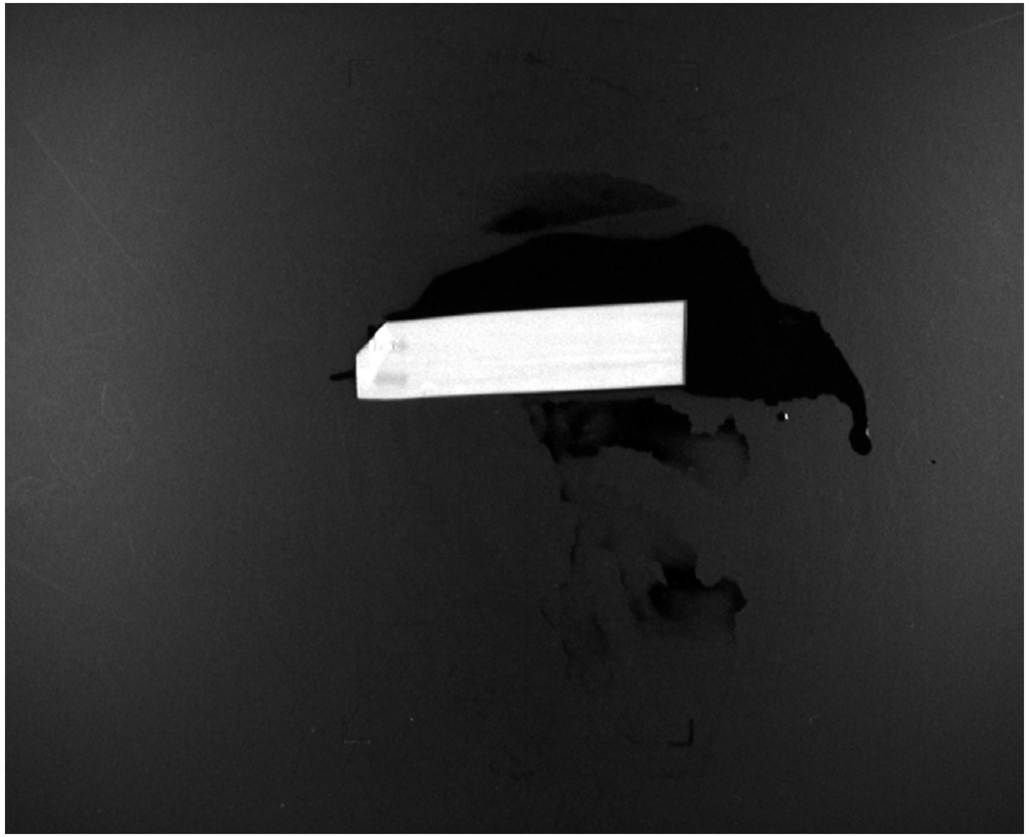

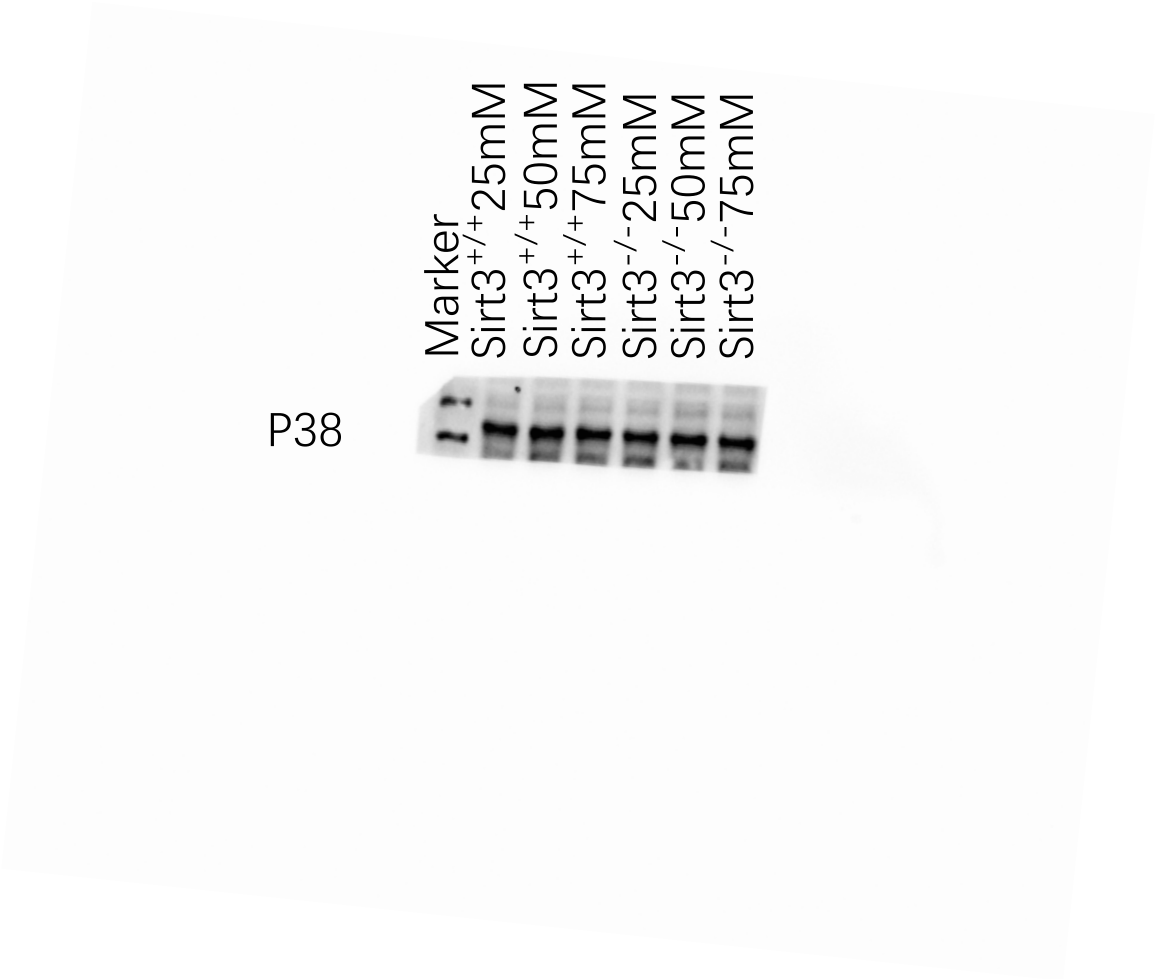
Marker
Sirt3$^{+/+}$25mM
Sirt3$^{+/+}$50mM
Sirt3$^{+/+}$75mM
Sirt3$^{-/-}$25mM
Sirt3$^{-/-}$50mM
Sirt3$^{-/-}$75mM
P38

Fig.4c

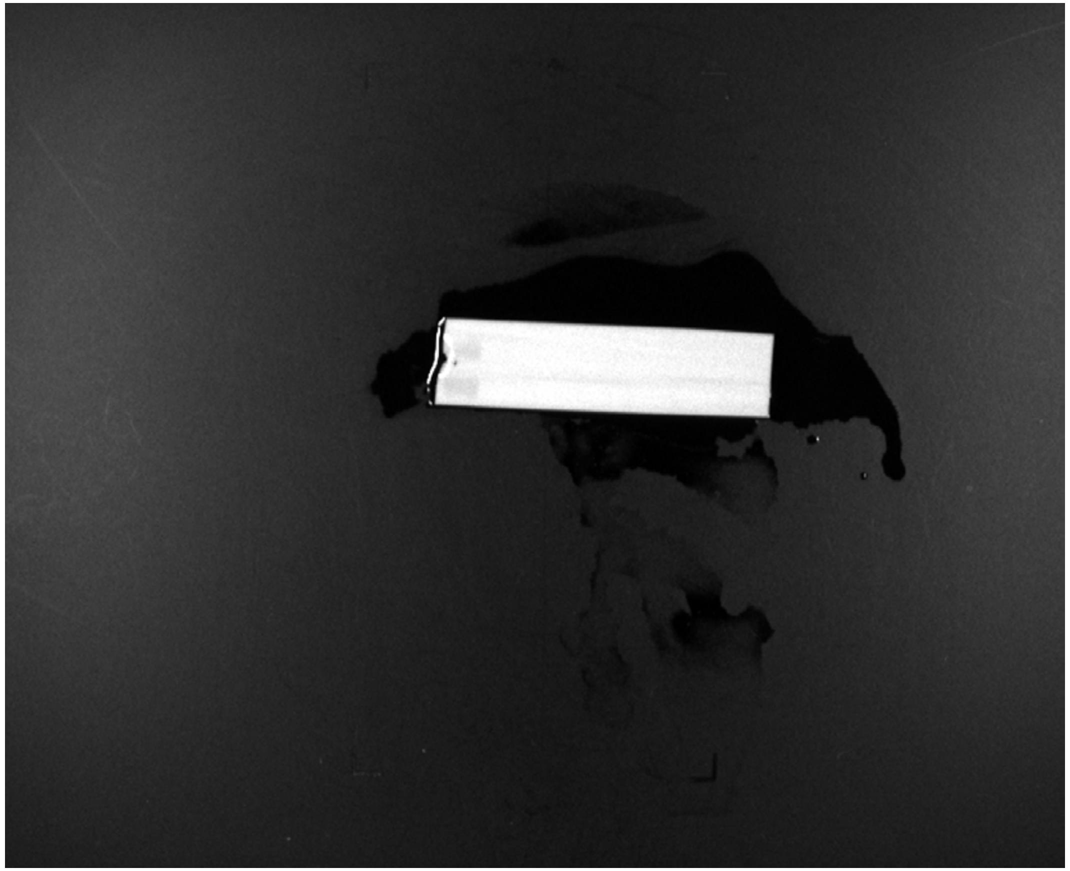

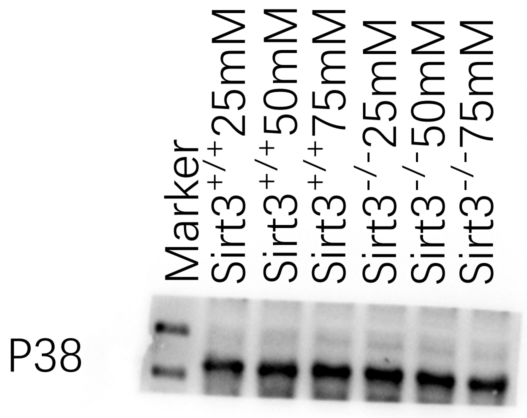

Marker
Sirt3+/+25mM
Sirt3+/+50mM
Sirt3+/+75mM
Sirt3-/-25mM
Sirt3-/-50mM
Sirt3-/-75mM
P38

Fig.4c

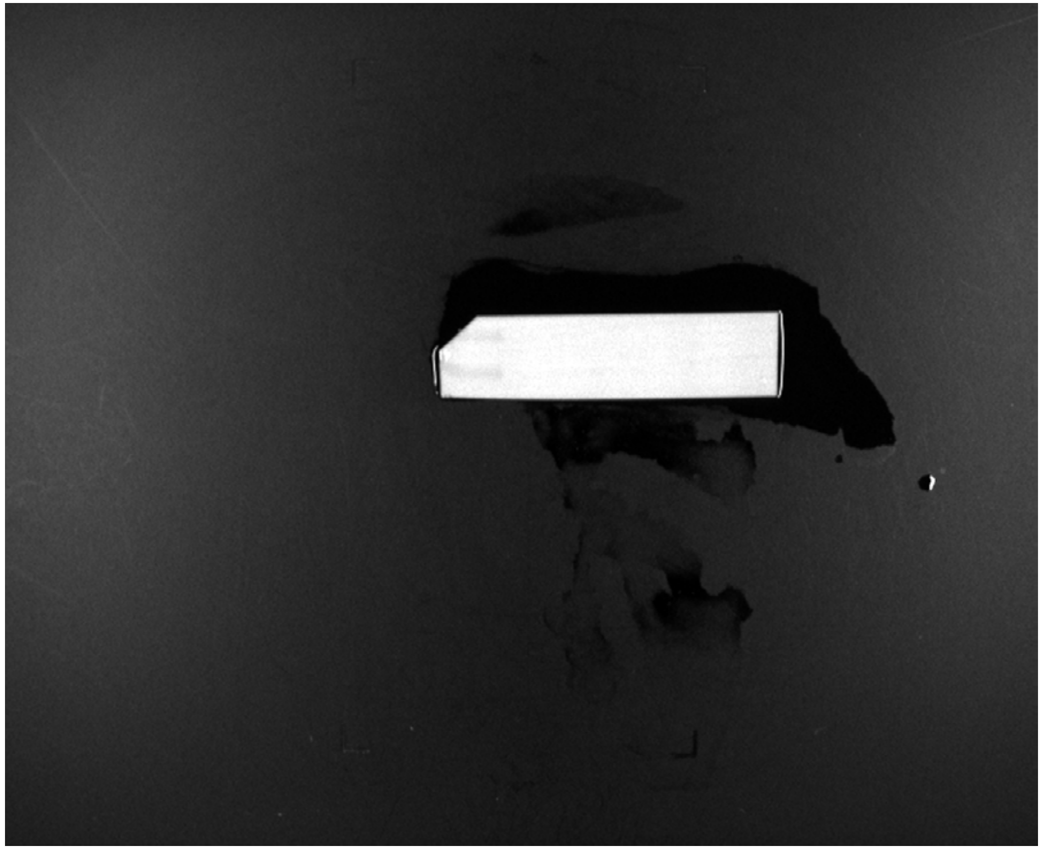

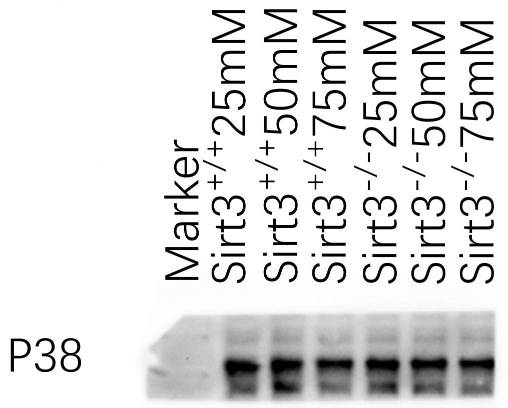
Marker
Sirt3+/+25mM
Sirt3+/+50mM
Sirt3+/+75mM
Sirt3-/-25mM
Sirt3-/-50mM
Sirt3-/-75mM
P38

Fig.4c

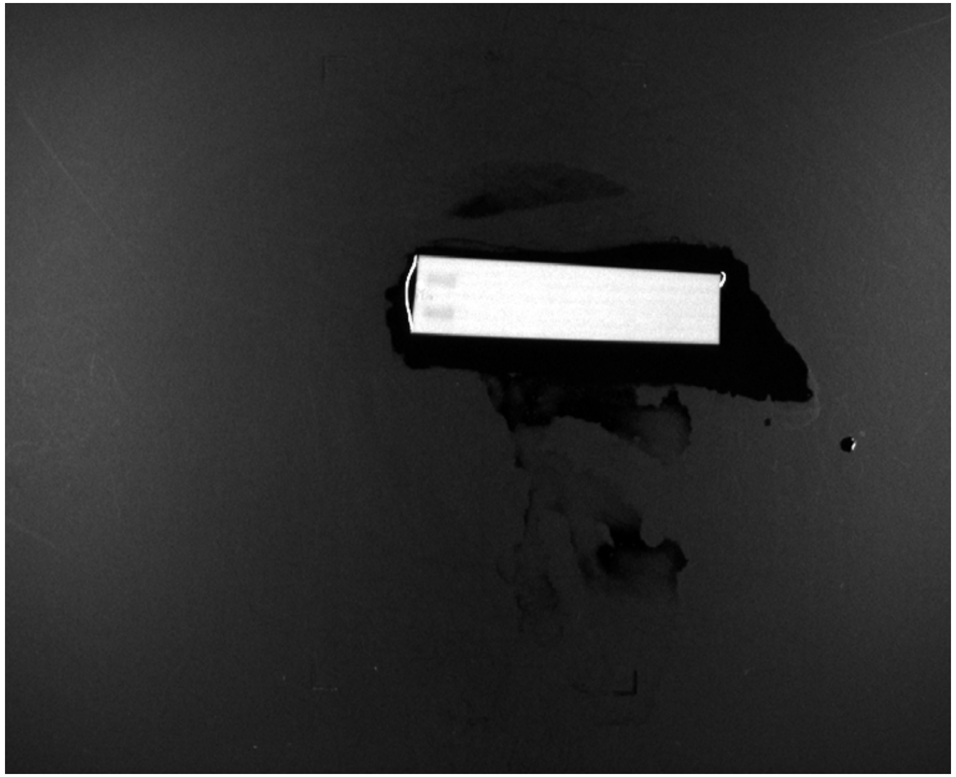

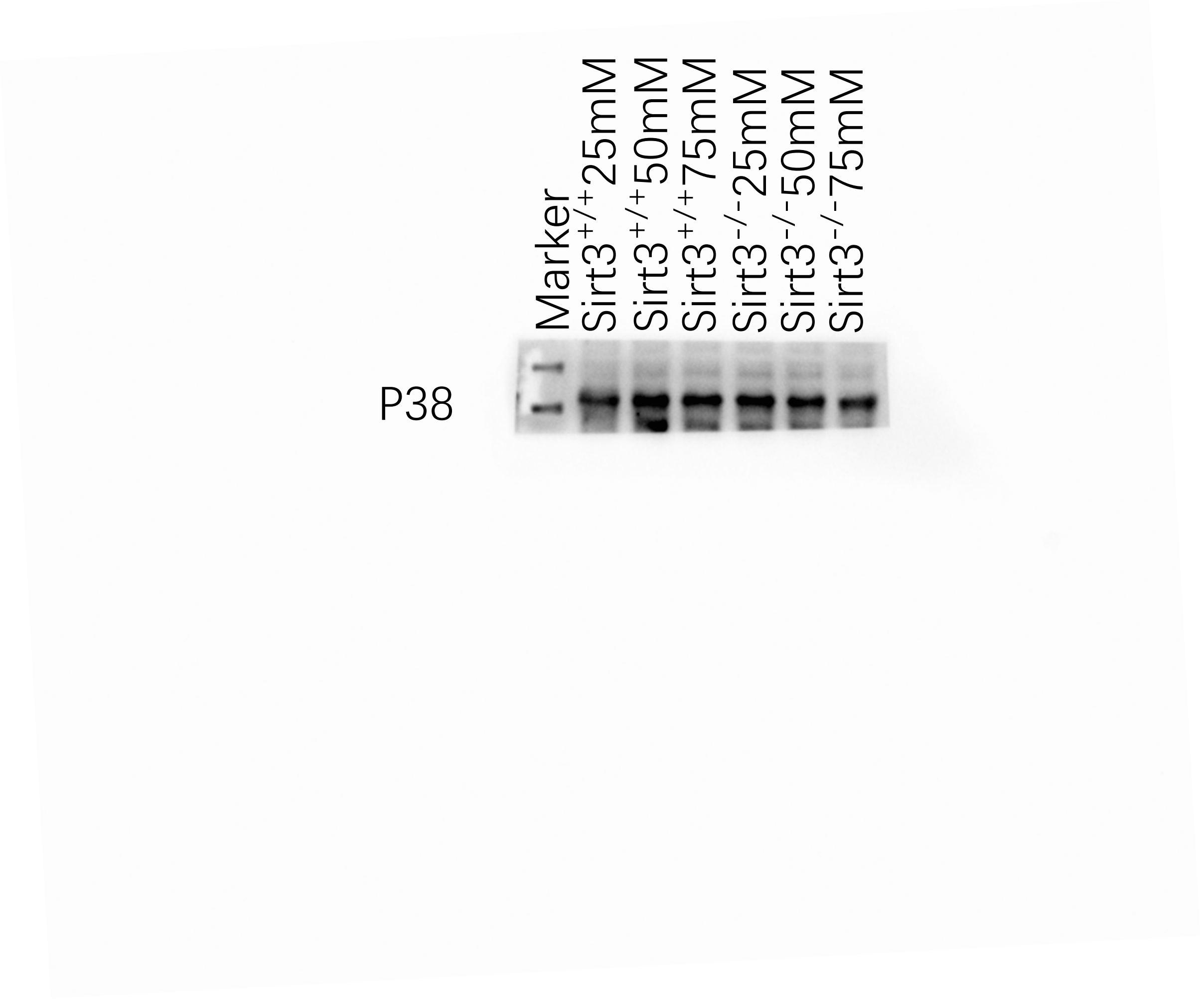
Marker
Sirt3+/+25mM
Sirt3+/+50mM
Sirt3+/+75mM
Sirt3-/-25mM
Sirt3-/-50mM
Sirt3-/-75mM
P38

Fig.4e

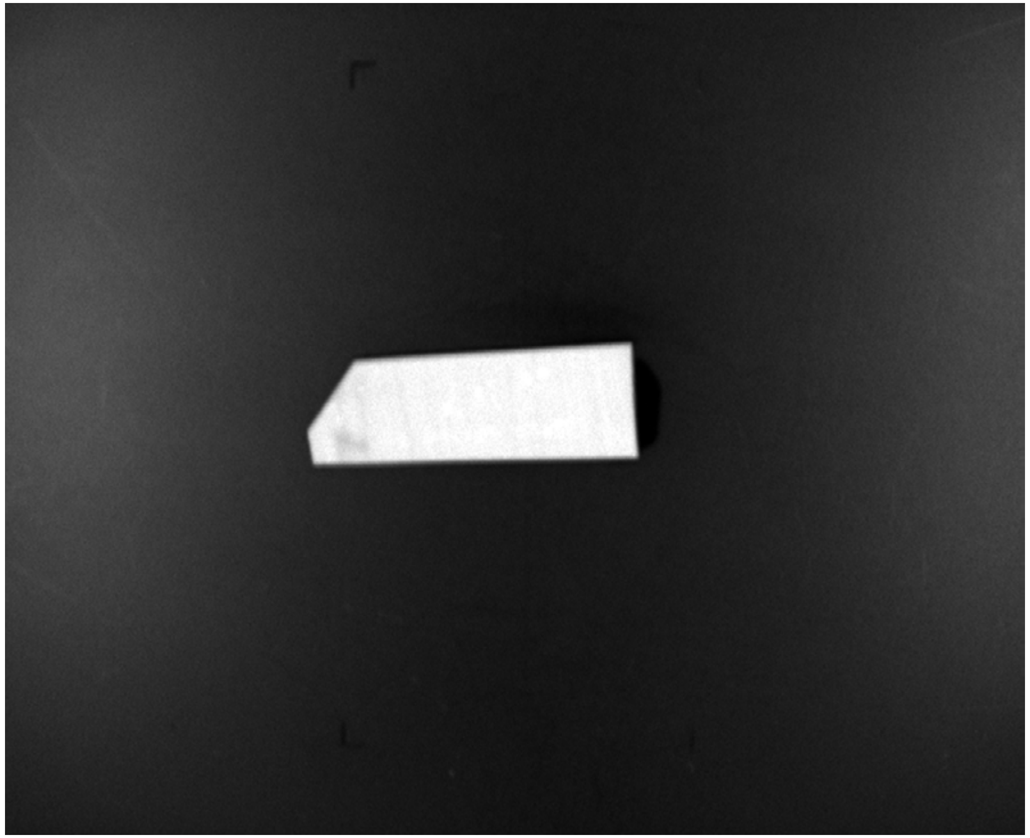

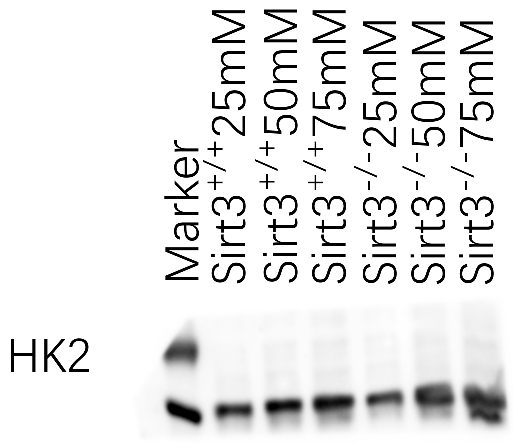
Marker
Sirt3+/+25mM
Sirt3+/+50mM
Sirt3+/+75mM
Sirt3-/-25mM
Sirt3-/-50mM
Sirt3-/-75mM
HK2

Fig.4e

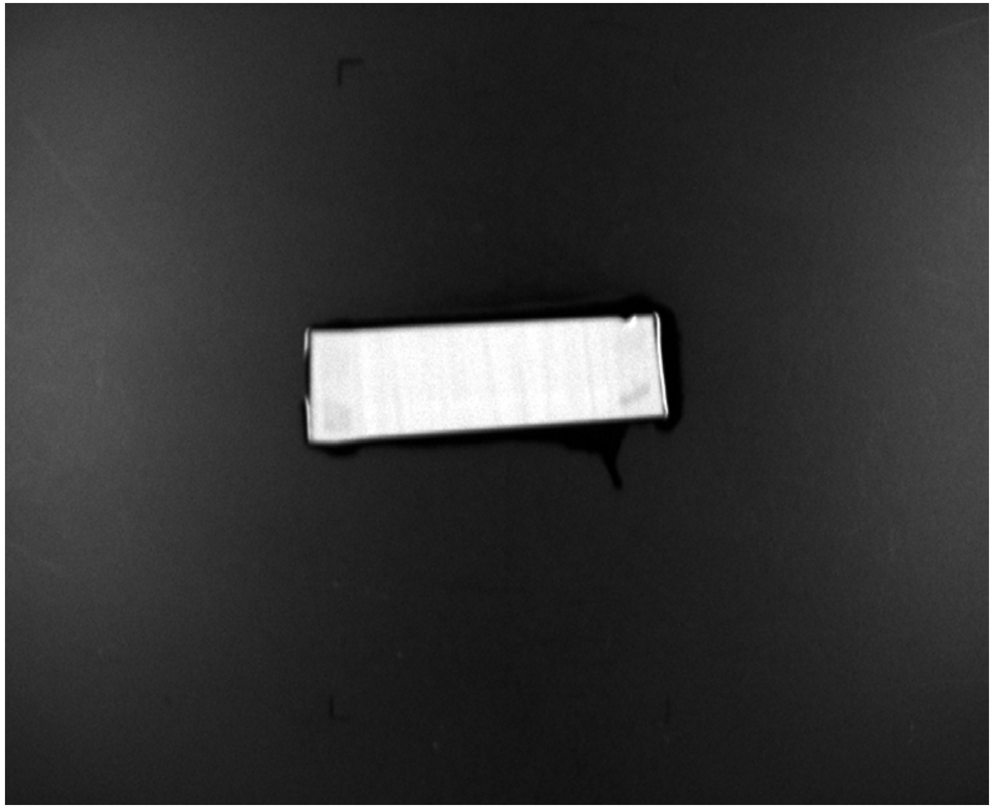

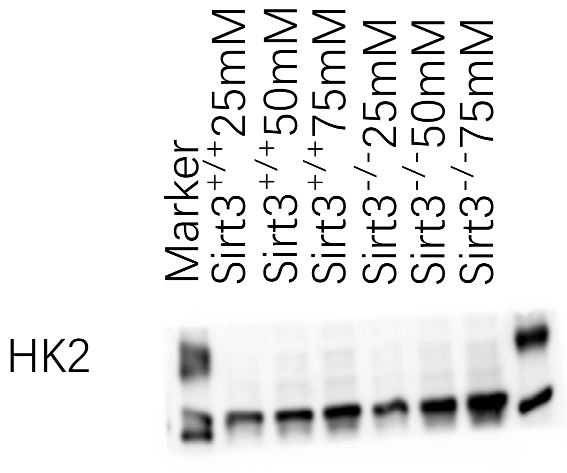

HK2
Marker
Sirt3+/+25mM
Sirt3+/+50mM
Sirt3+/+75mM
Sirt3-/-25mM
Sirt3-/-50mM
Sirt3-/-75mM

Fig.4e

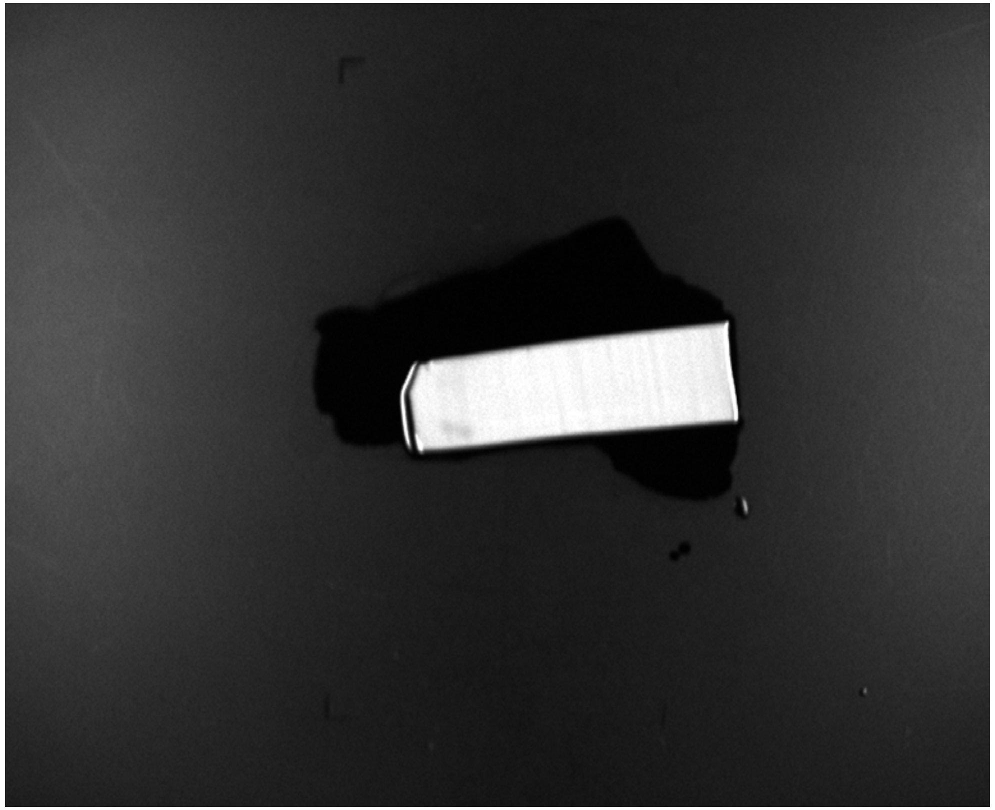

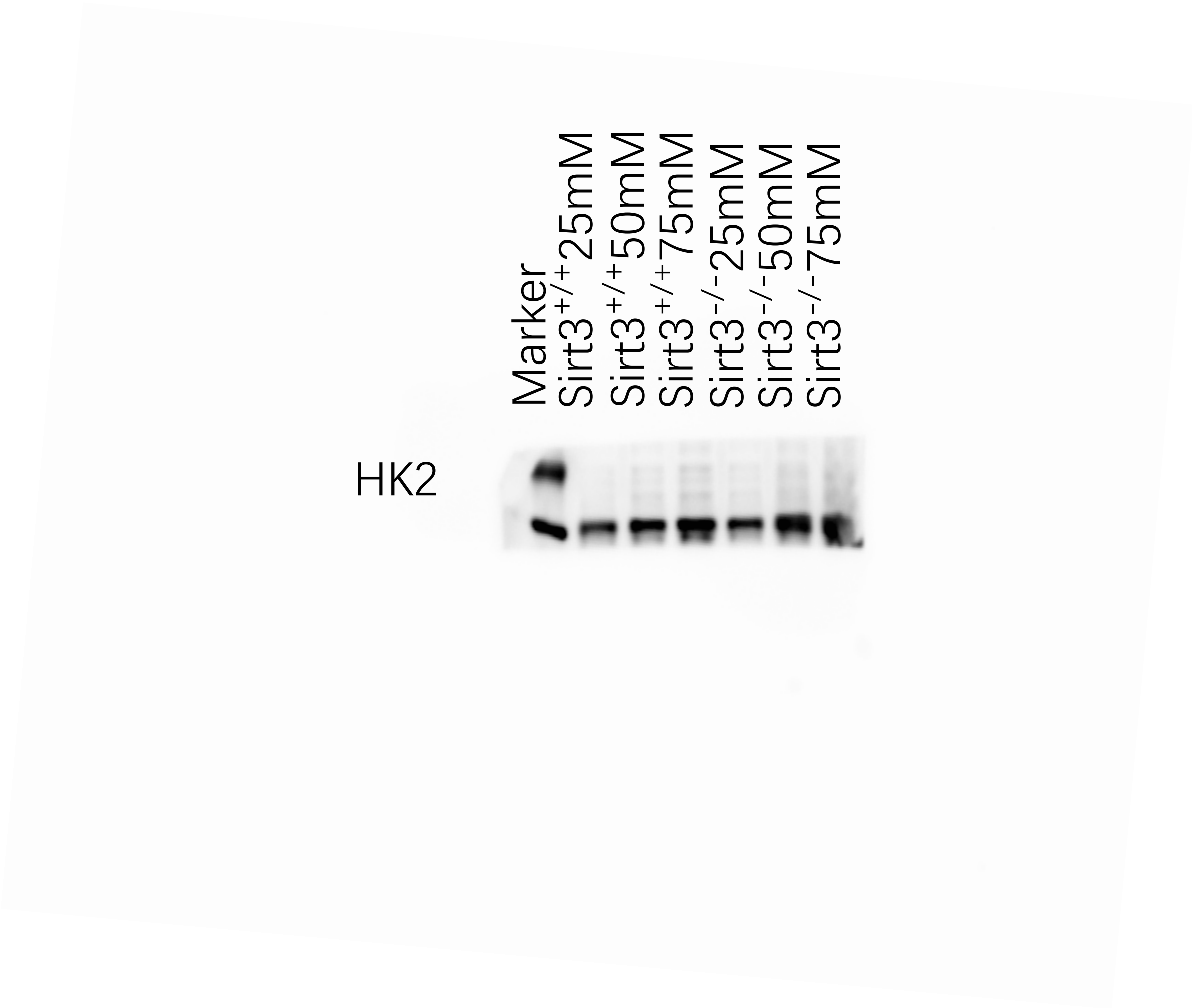
Marker
Sirt3+/+25mM
Sirt3+/+50mM
Sirt3+/+75mM
Sirt3-/-25mM
Sirt3-/-50mM
Sirt3-/-75mM
HK2

Fig.4e

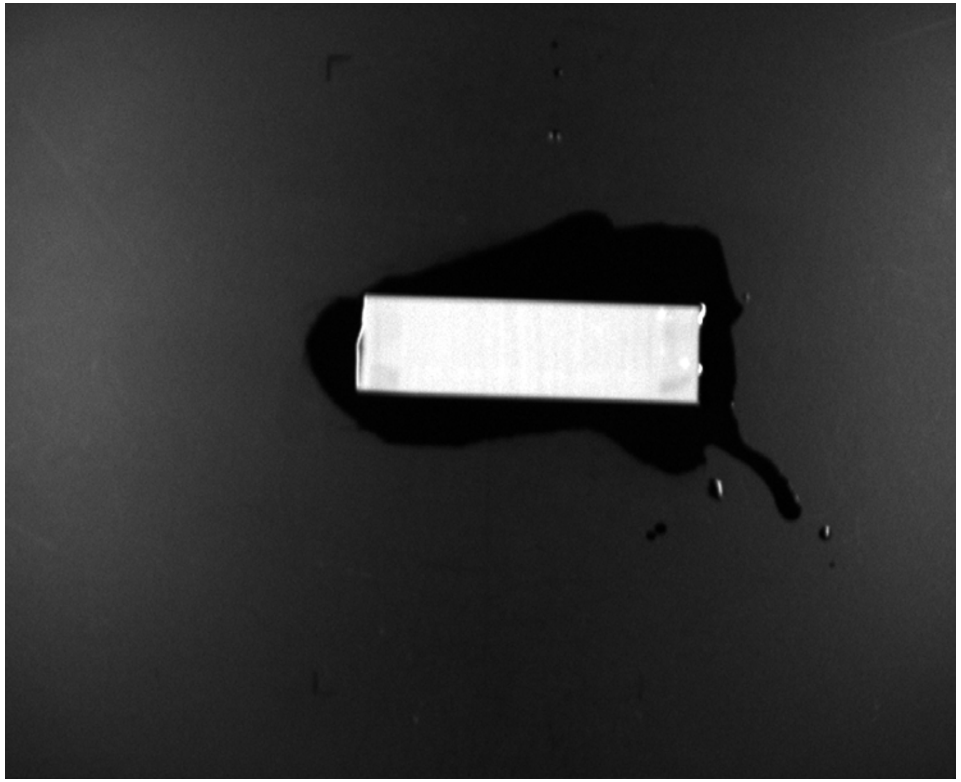

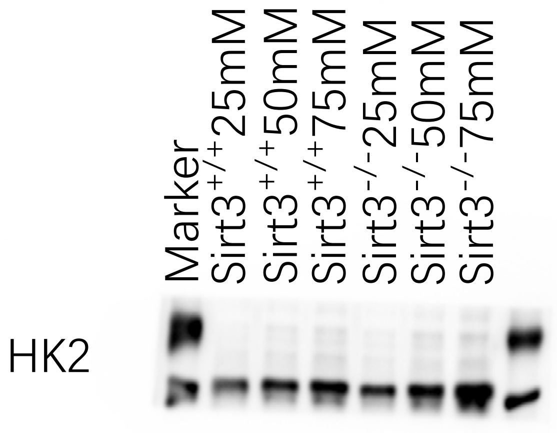
Marker
Sirt3$^{+/+}$25mM
Sirt3$^{+/+}$50mM
Sirt3$^{+/+}$75mM
Sirt3$^{-/-}$25mM
Sirt3$^{-/-}$50mM
Sirt3$^{-/-}$75mM
HK2

Fig.4e

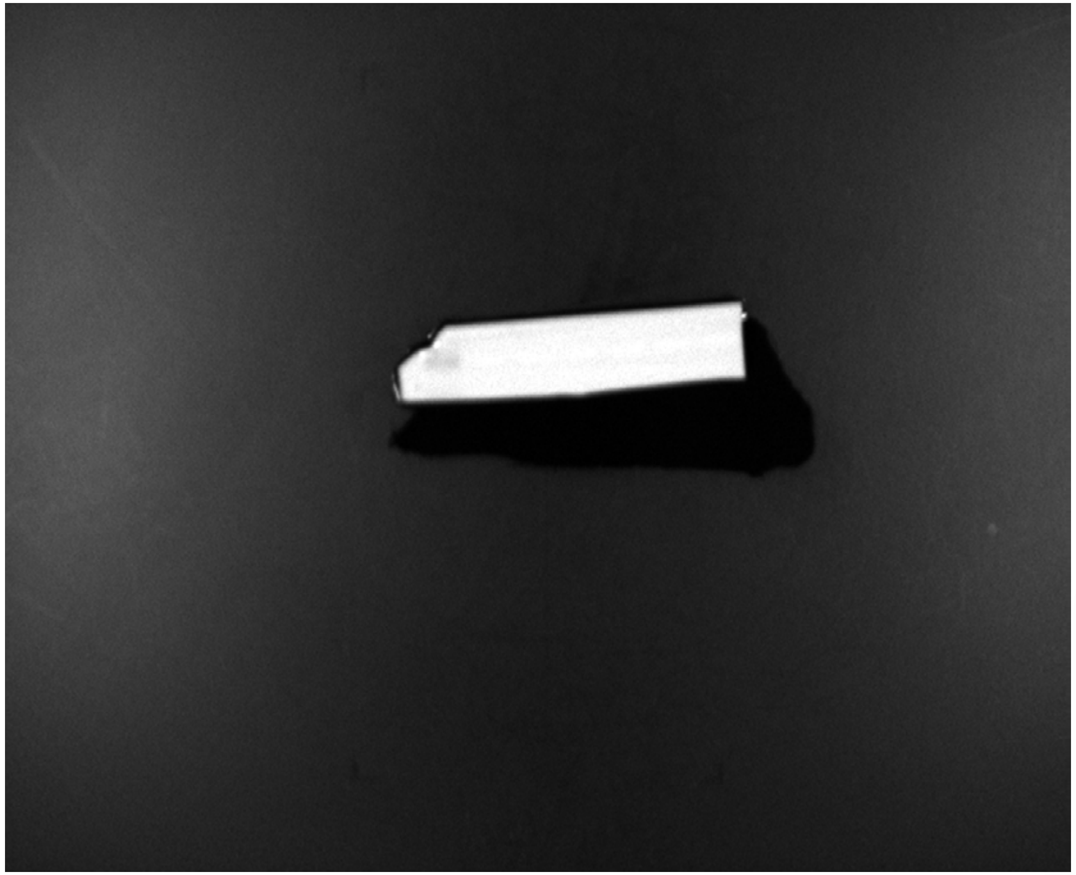

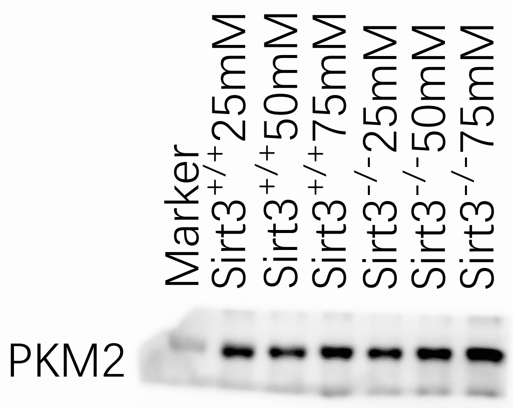
Marker
Sirt3+/+25mM
Sirt3+/+50mM
Sirt3+/+75mM
Sirt3-/-25mM
Sirt3-/-50mM
Sirt3-/-75mM
PKM2

Fig.4e

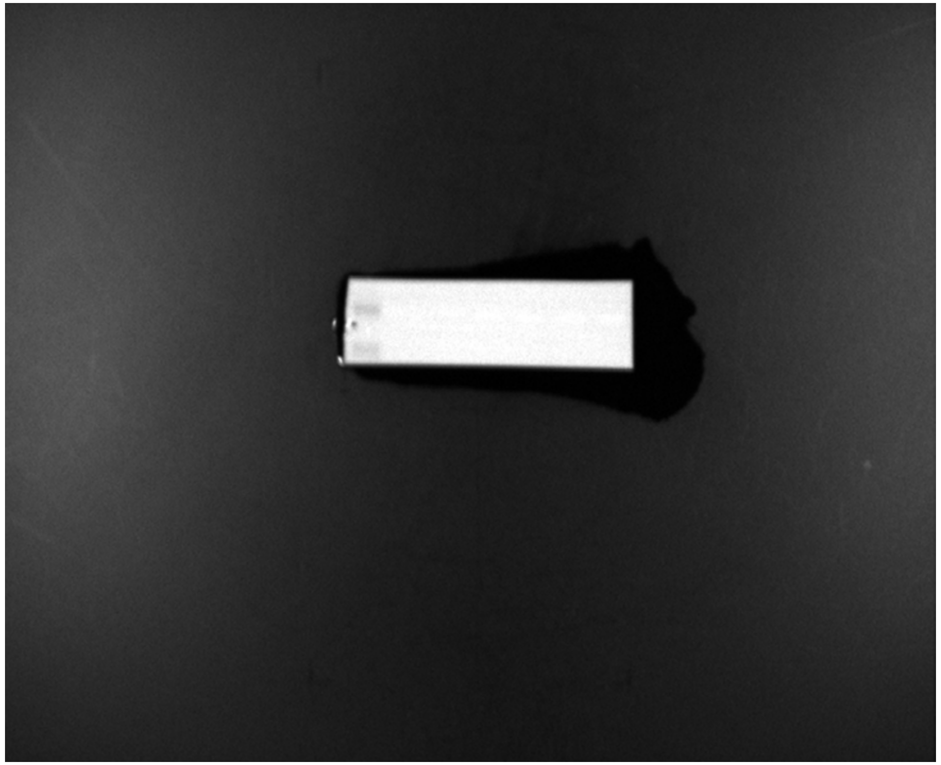

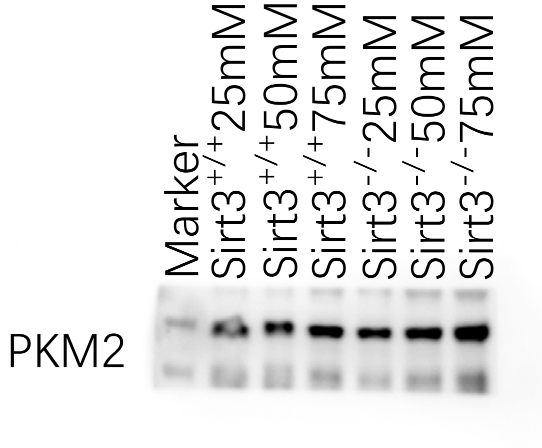
Marker
Sirt3$^{+/+}$25mM
Sirt3$^{+/+}$50mM
Sirt3$^{+/+}$75mM
Sirt3$^{-/-}$25mM
Sirt3$^{-/-}$50mM
Sirt3$^{-/-}$75mM
PKM2

Fig.4e

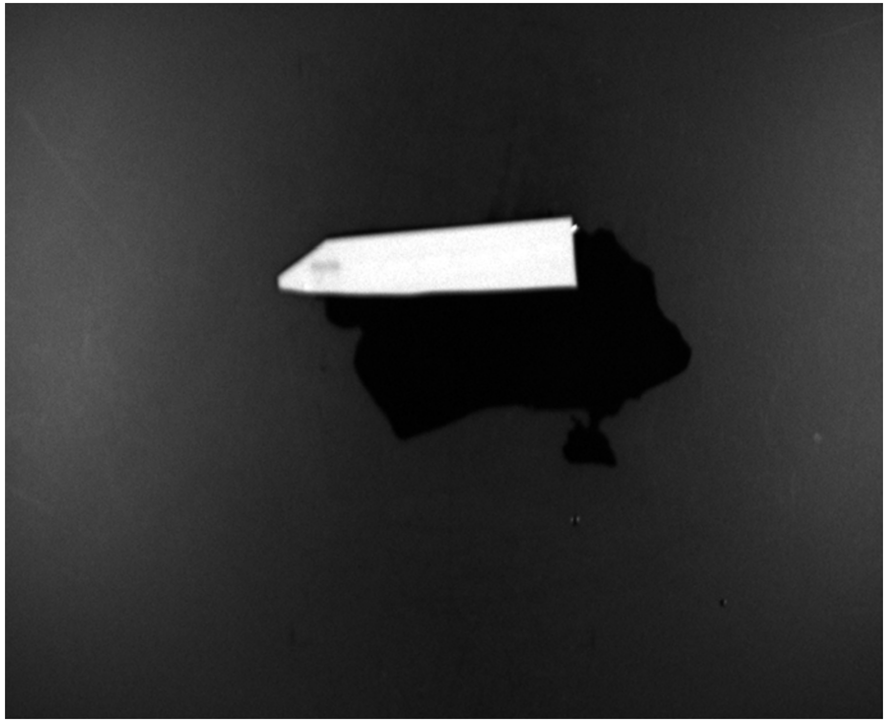

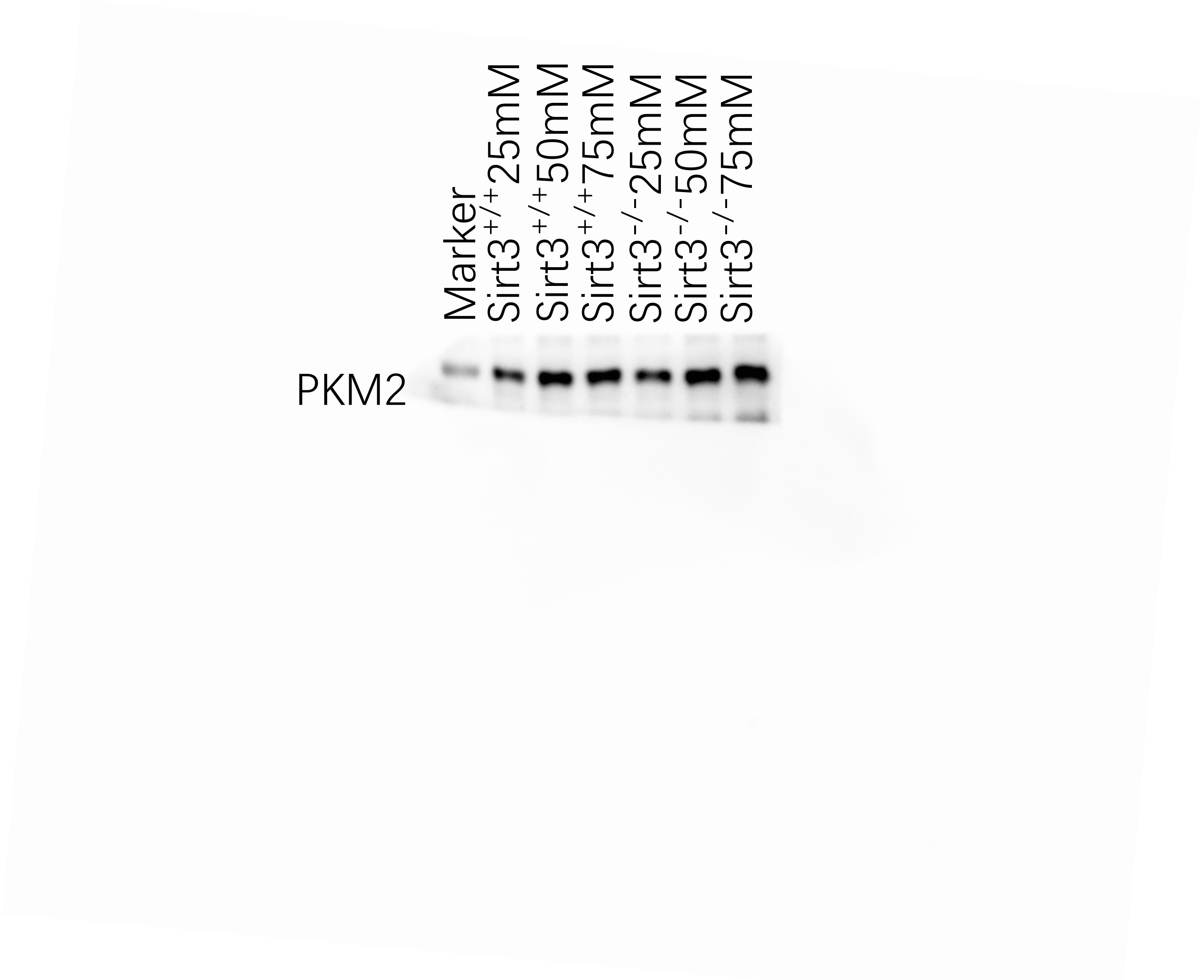
Marker
Sirt3+/+25mM
Sirt3+/+50mM
Sirt3+/+75mM
Sirt3-/-25mM
Sirt3-/-50mM
Sirt3-/-75mM
PKM2

Fig.4e

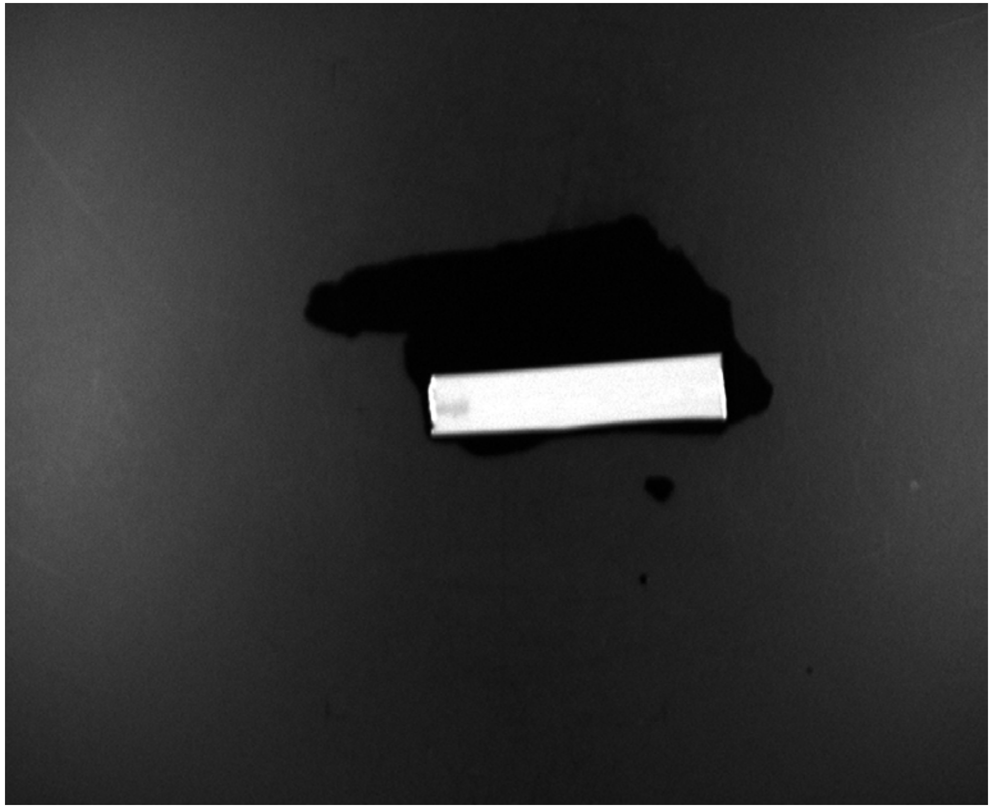

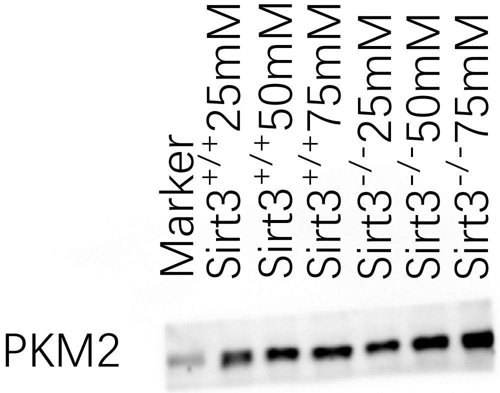
Marker
Sirt3+/+25mM
Sirt3+/+50mM
Sirt3+/+75mM
Sirt3-/-25mM
Sirt3-/-50mM
Sirt3-/-75mM
PKM2

Fig.4e

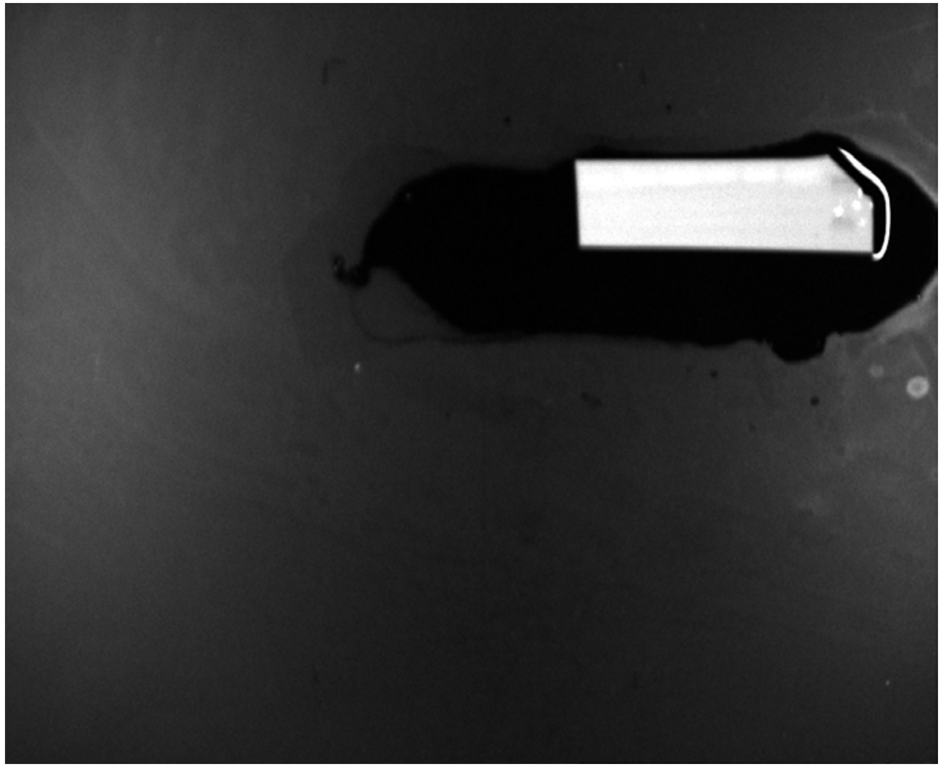

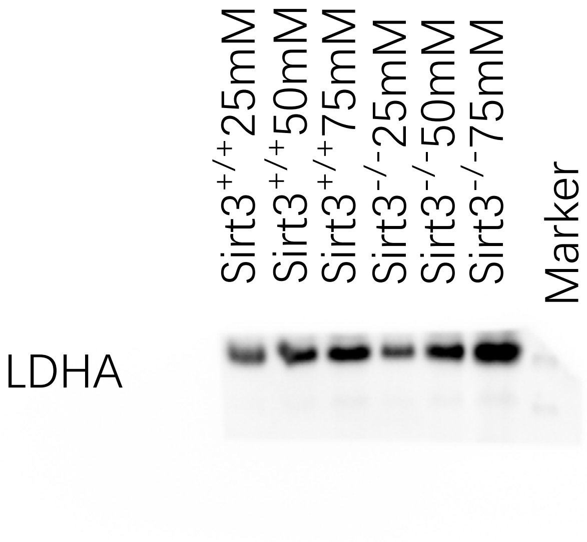

Sirt3+/+25mM
Sirt3+/+50mM
Sirt3+/+75mM
Sirt3-/-25mM
Sirt3-/-50mM
Sirt3-/-75mM
Marker
LDHA

Fig.4e

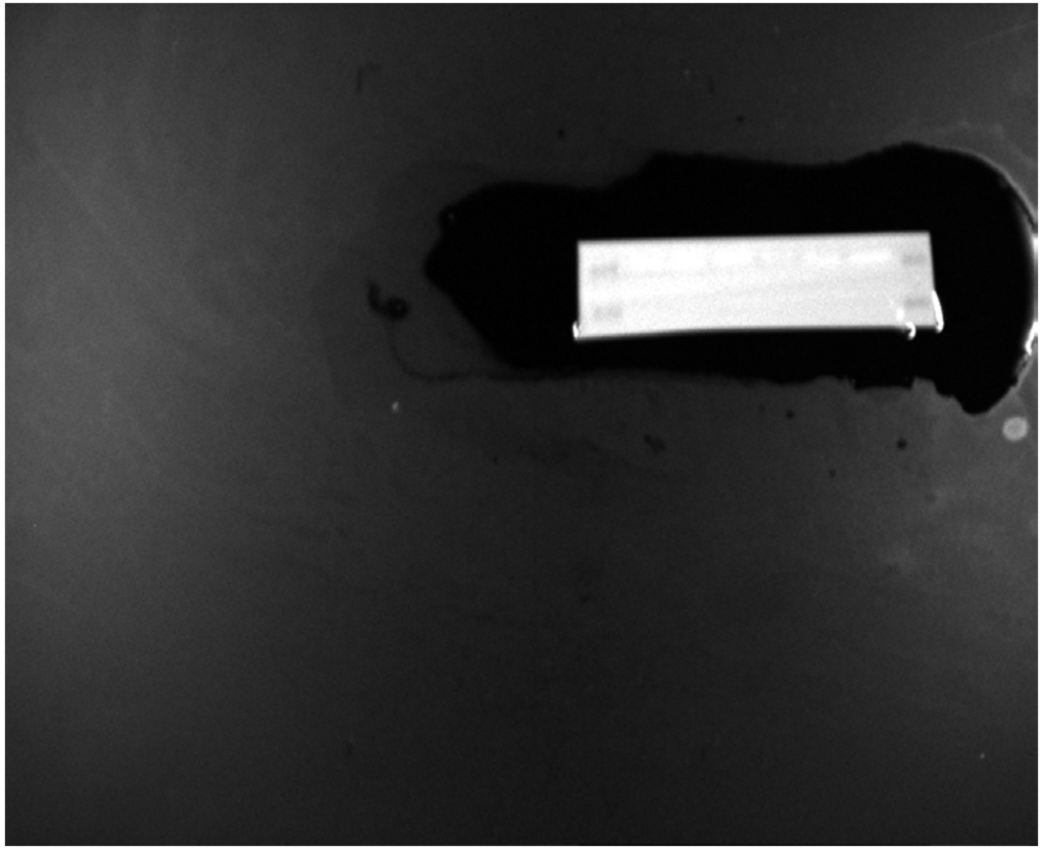

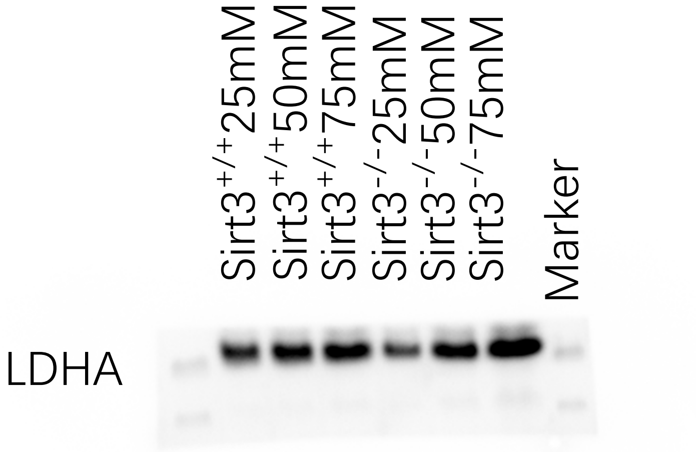
Sirt3+/+25mM
Sirt3+/+50mM
Sirt3+/+75mM
Sirt3-/-25mM
Sirt3-/-50mM
Sirt3-/-75mM
Marker
LDHA

Fig.4e

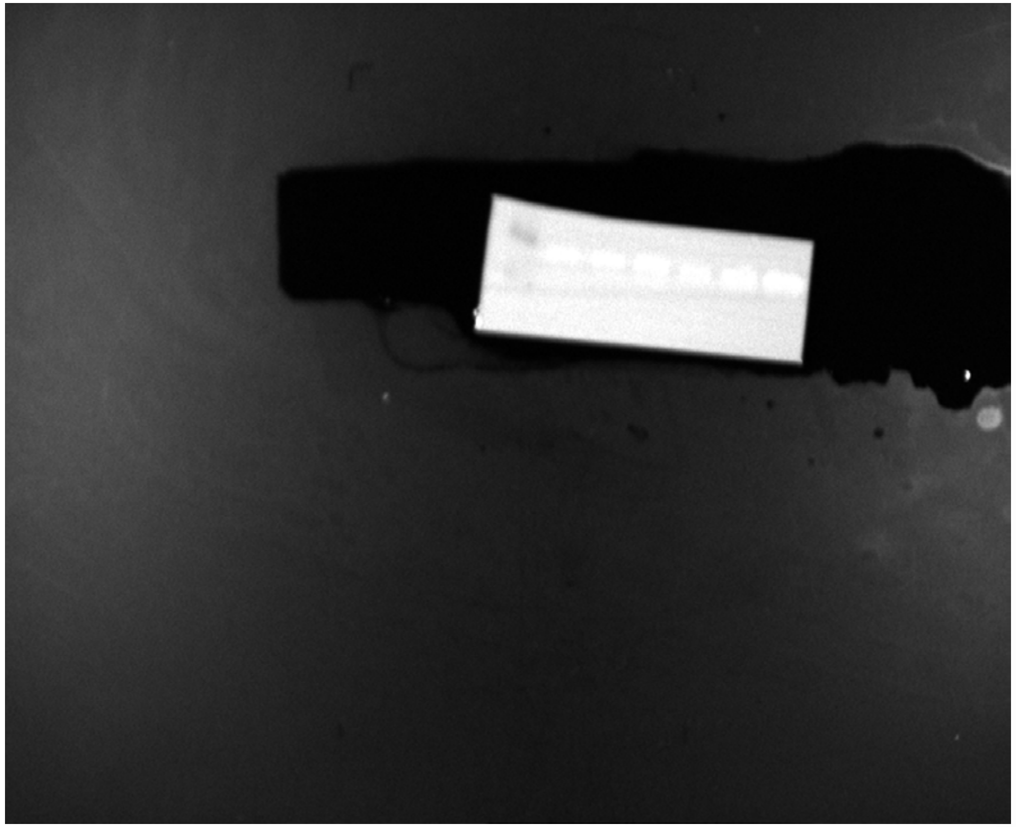

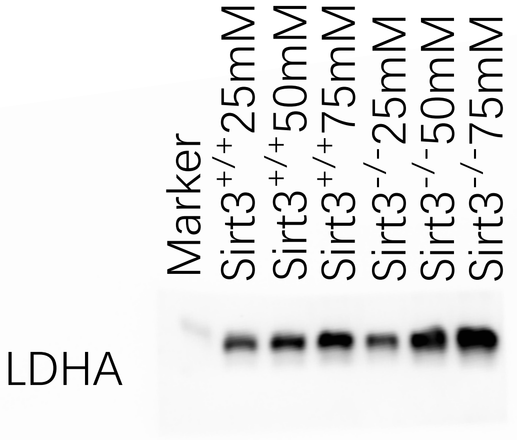

Marker
Sirt3+/+25mM
Sirt3+/+50mM
Sirt3+/+75mM
Sirt3-/-25mM
Sirt3-/-50mM
Sirt3-/-75mM
LDHA

Fig.4e

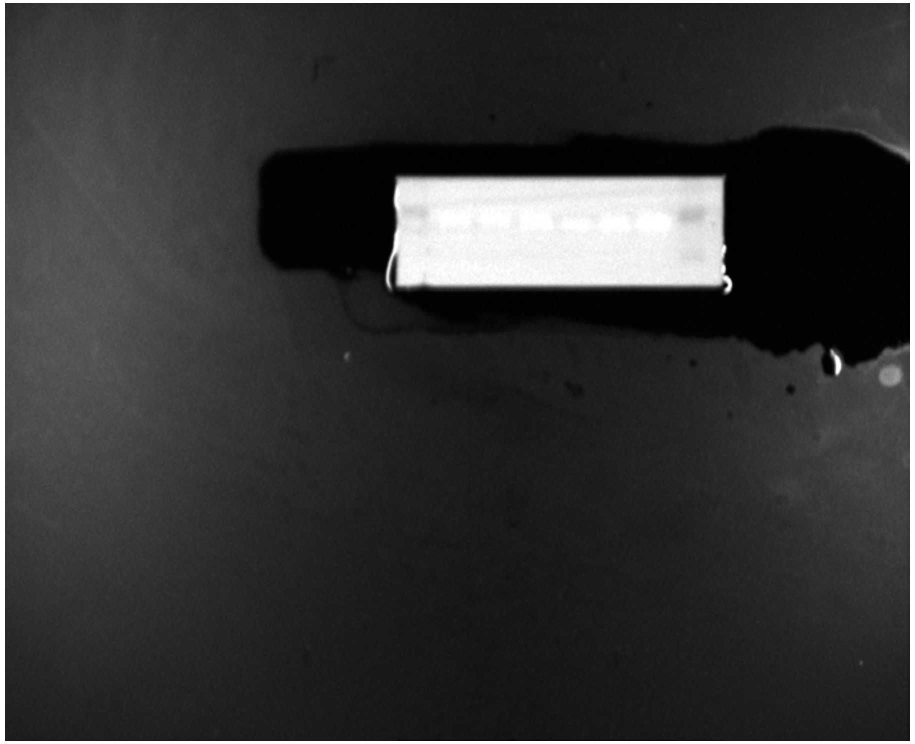

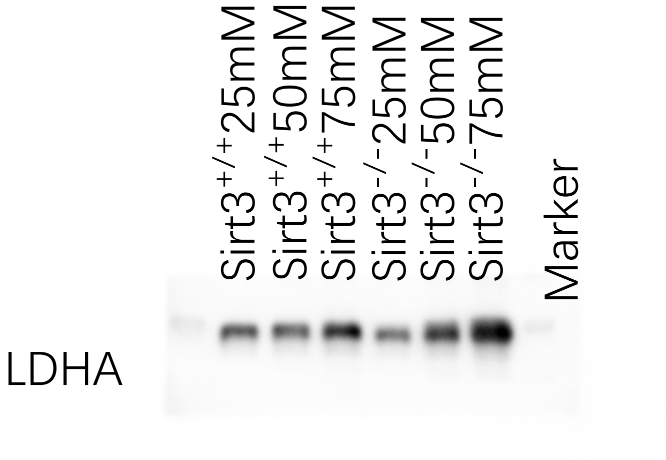
Sirt3+/+25mM
Sirt3+/+50mM
Sirt3+/+75mM
Sirt3-/-25mM
Sirt3-/-50mM
Sirt3-/-75mM
Marker
LDHA

Fig.5a

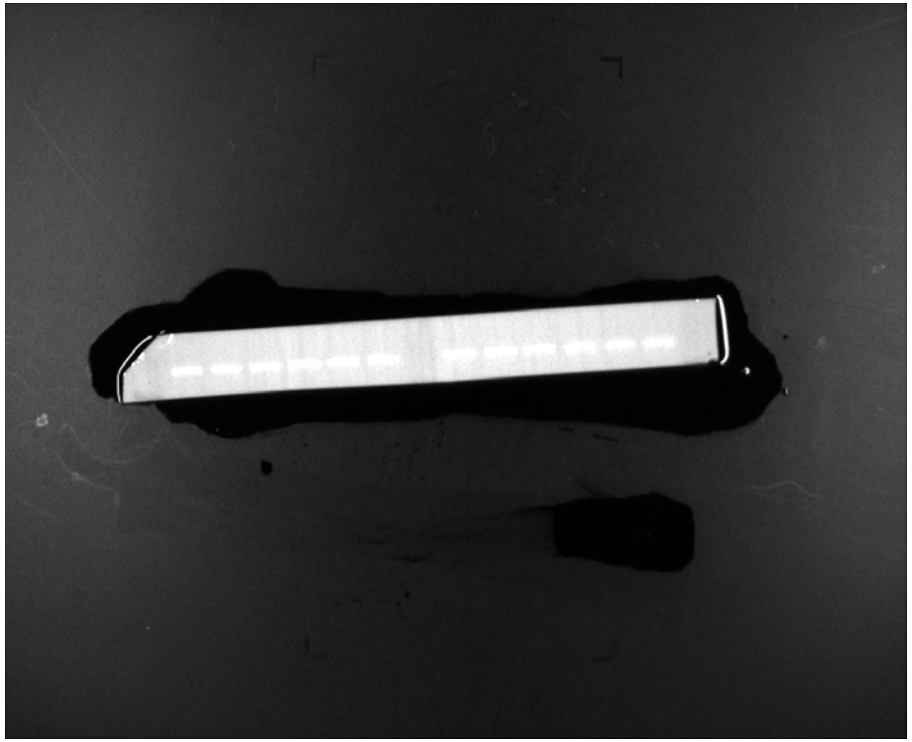

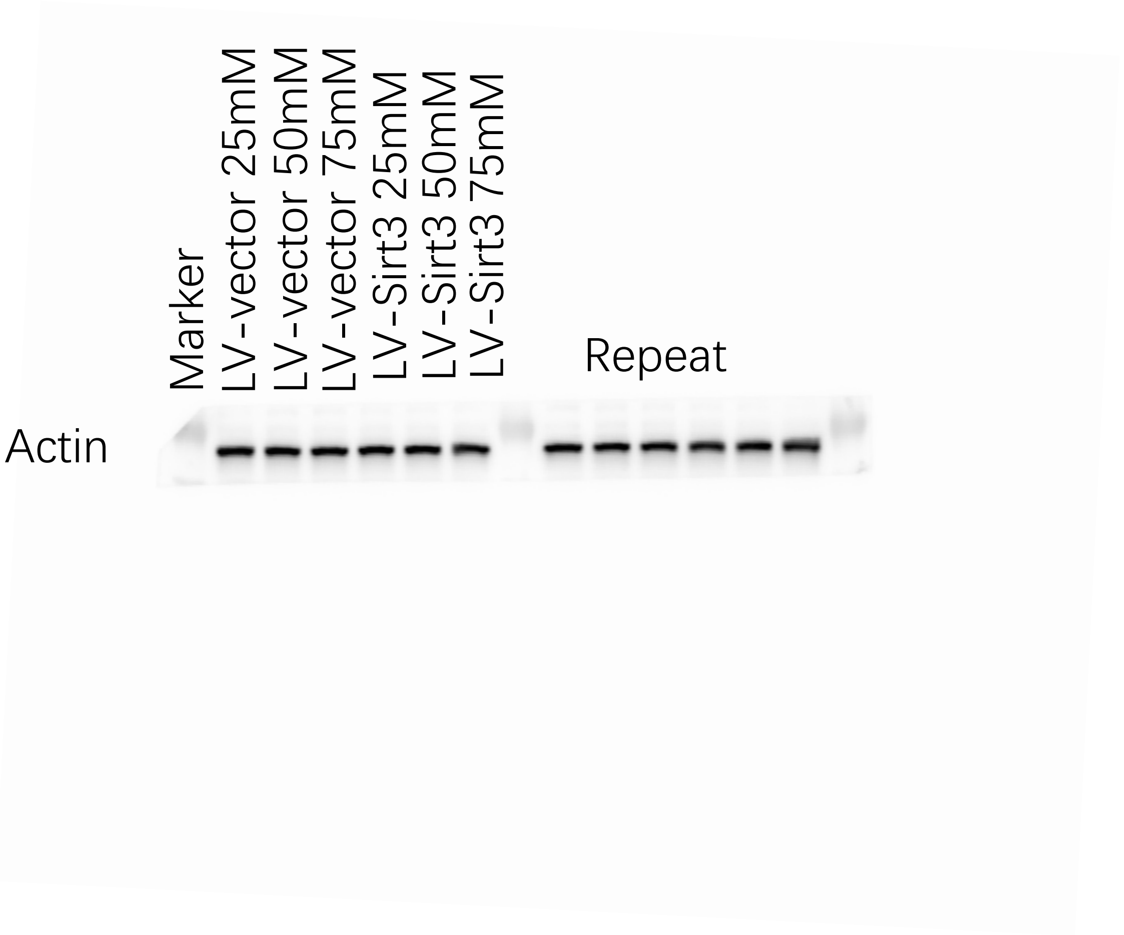
Marker
LV-vector 25mM
LV-vector 50mM
LV-vector 75mM
LV-Sirt3 25mM
LV-Sirt3 50mM
LV-Sirt3 75mM
Repeat
Actin

Fig.5a

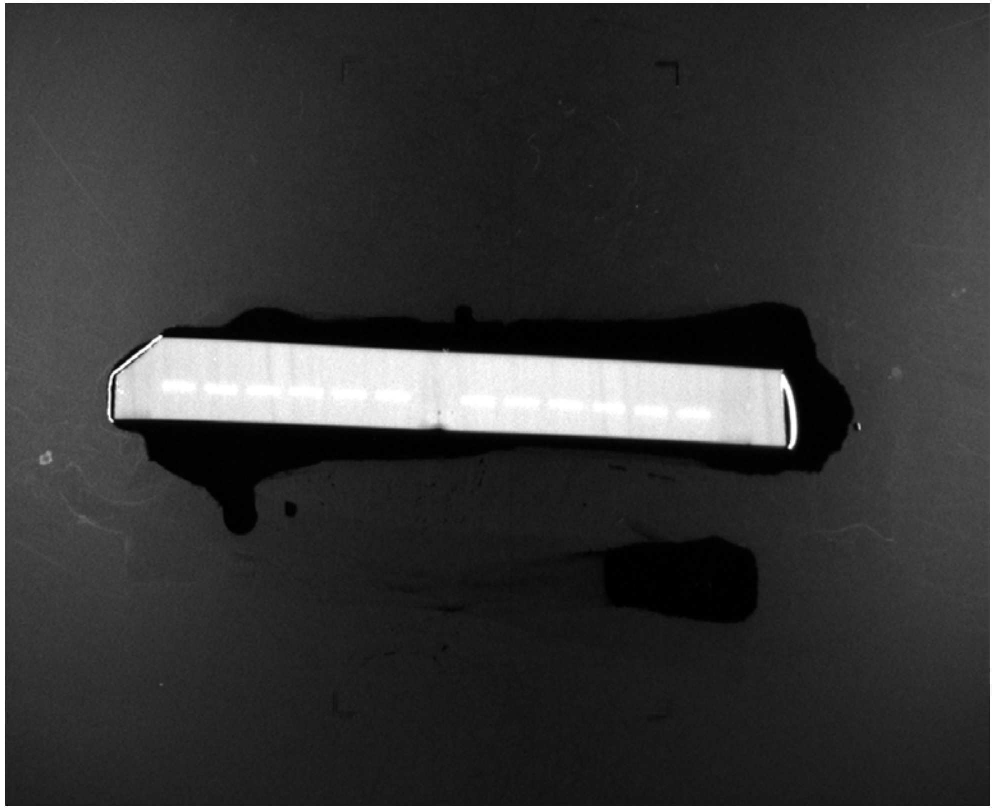

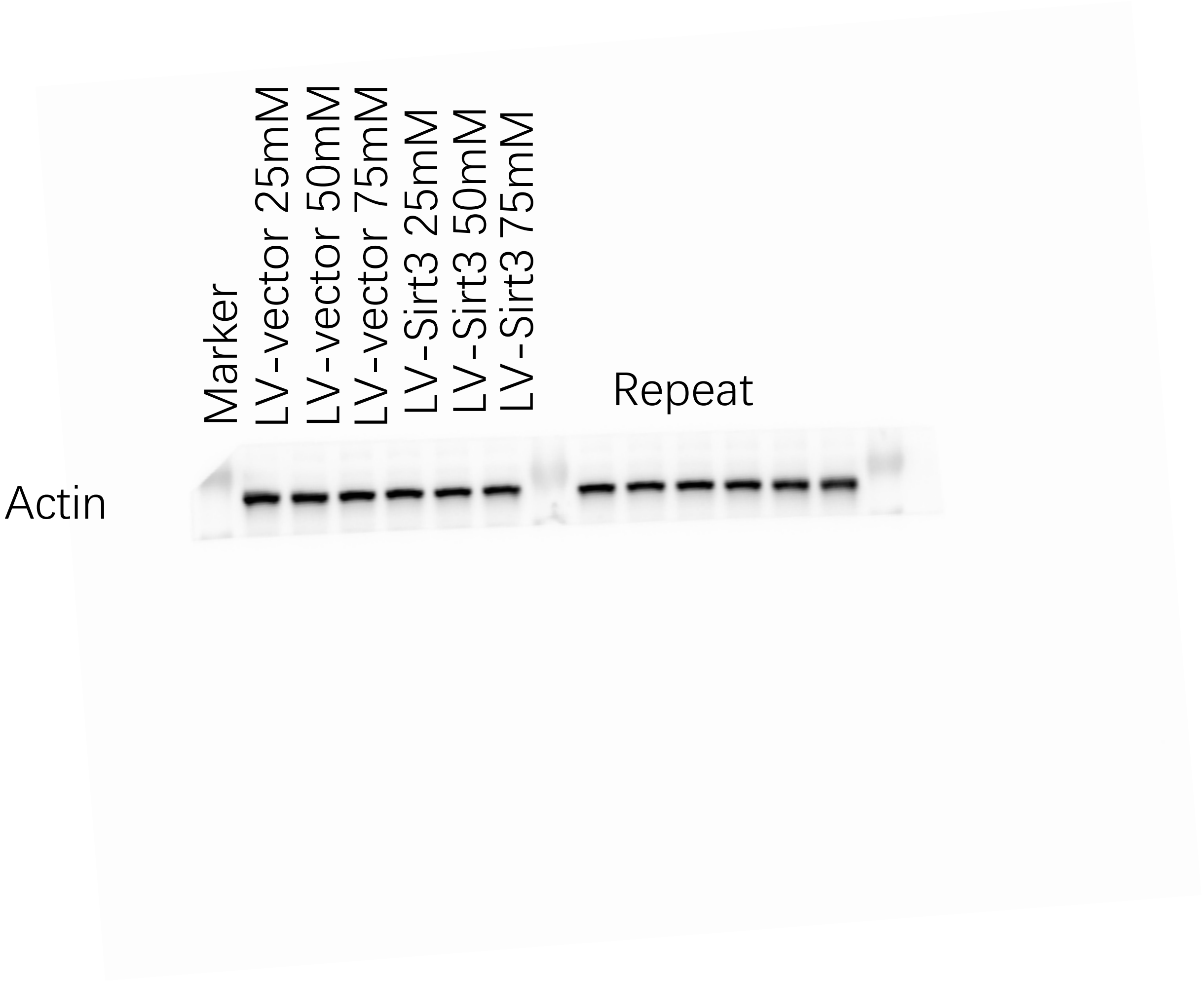
Marker
LV-vector 25mM
LV-vector 50mM
LV-vector 75mM
LV-Sirt3 25mM
LV-Sirt3 50mM
LV-Sirt3 75mM
Repeat
Actin

Fig.5a

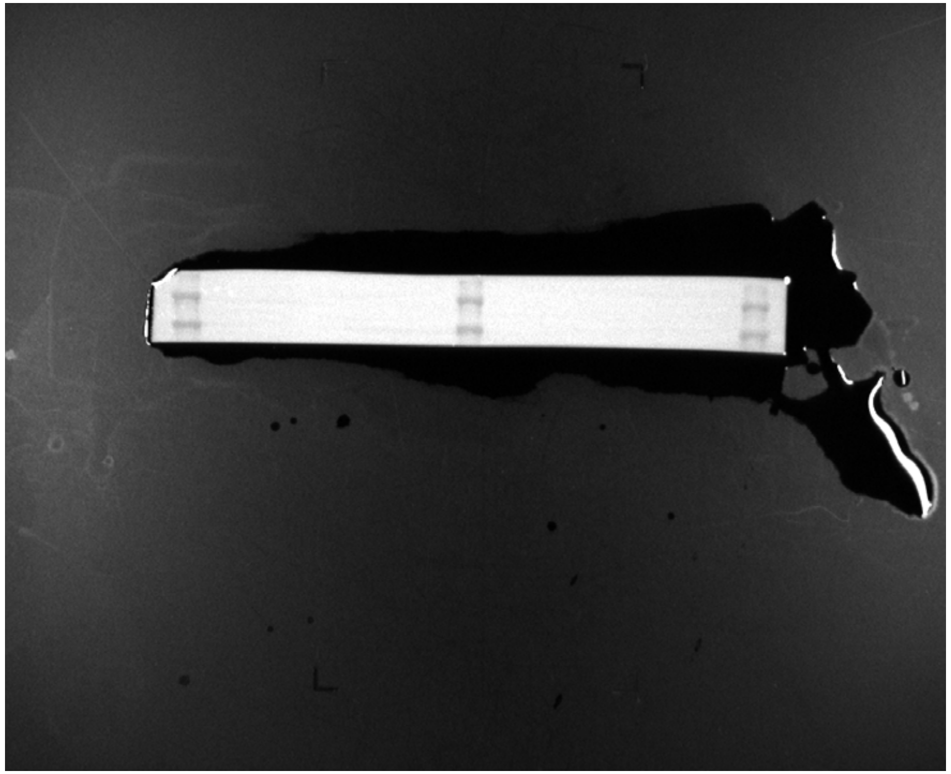

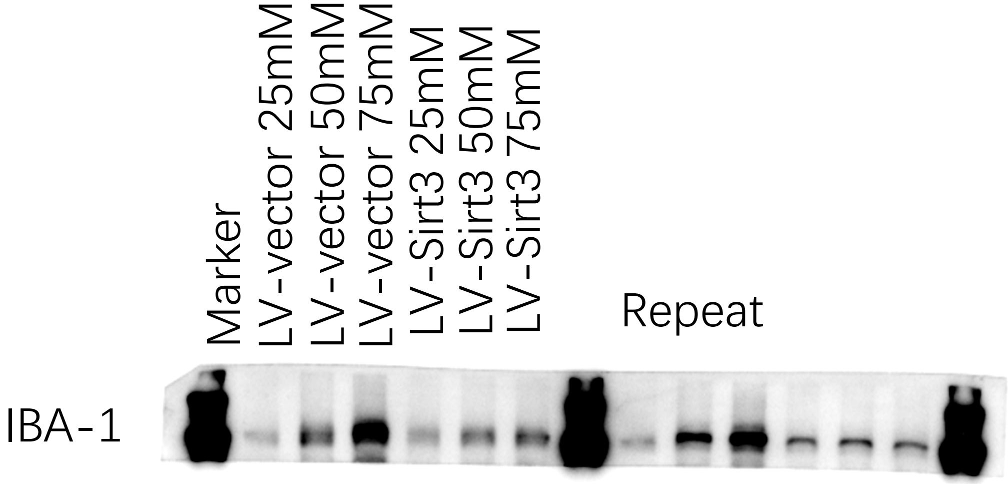
Marker
LV-vector 25mM
LV-vector 50mM
LV-vector 75mM
LV-Sirt3 25mM
LV-Sirt3 50mM
LV-Sirt3 75mM
Repeat
IBA-1

Fig.5a

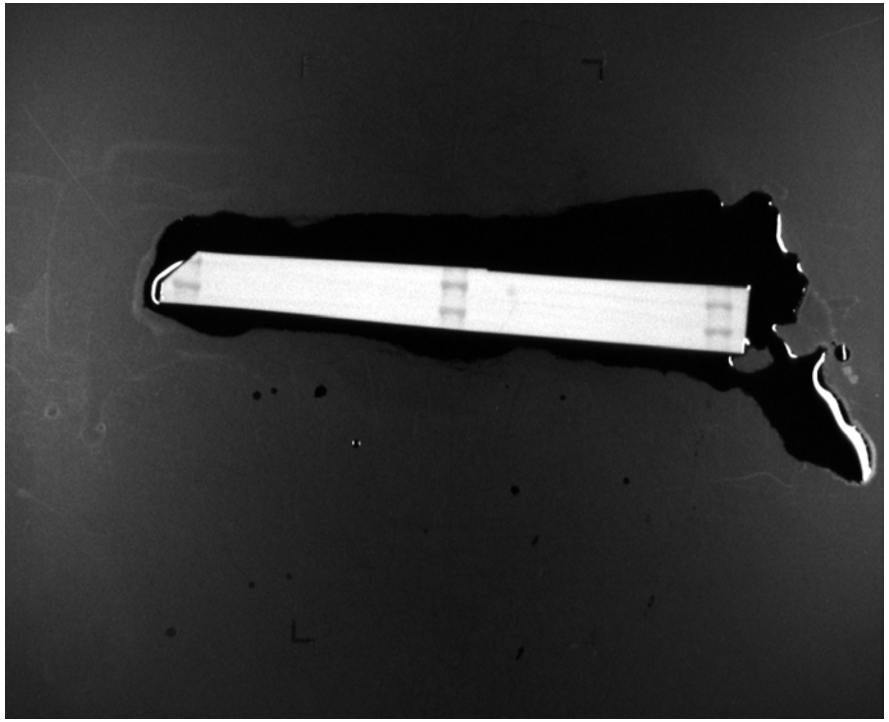

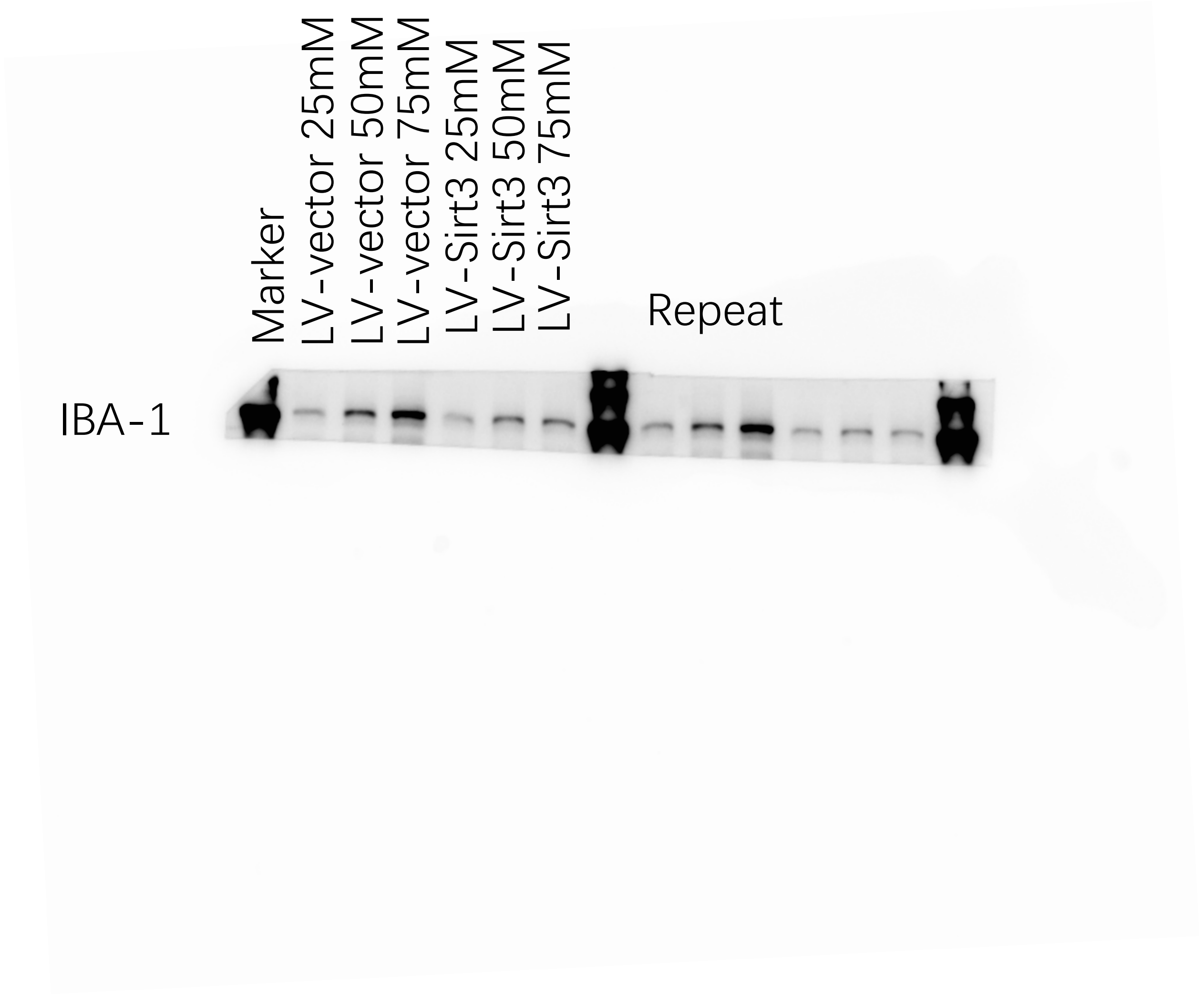
Marker
LV-vector 25mM
LV-vector 50mM
LV-vector 75mM
LV-Sirt3 25mM
LV-Sirt3 50mM
LV-Sirt3 75mM
Repeat
IBA-1

Fig.5c

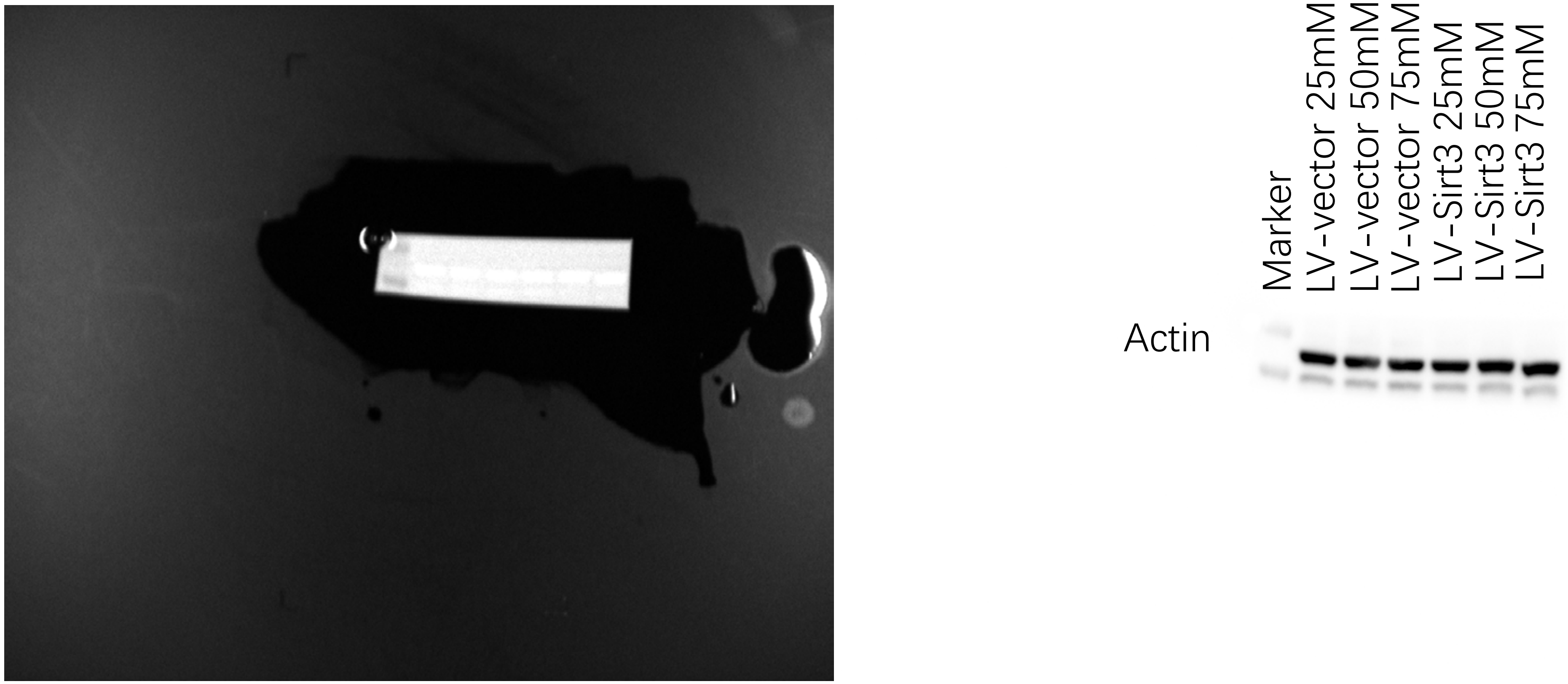
Marker
LV-vector 25mM
LV-vector 50mM
LV-vector 75mM
LV-Sirt3 25mM
LV-Sirt3 50mM
LV-Sirt3 75mM
Actin

Fig.5c

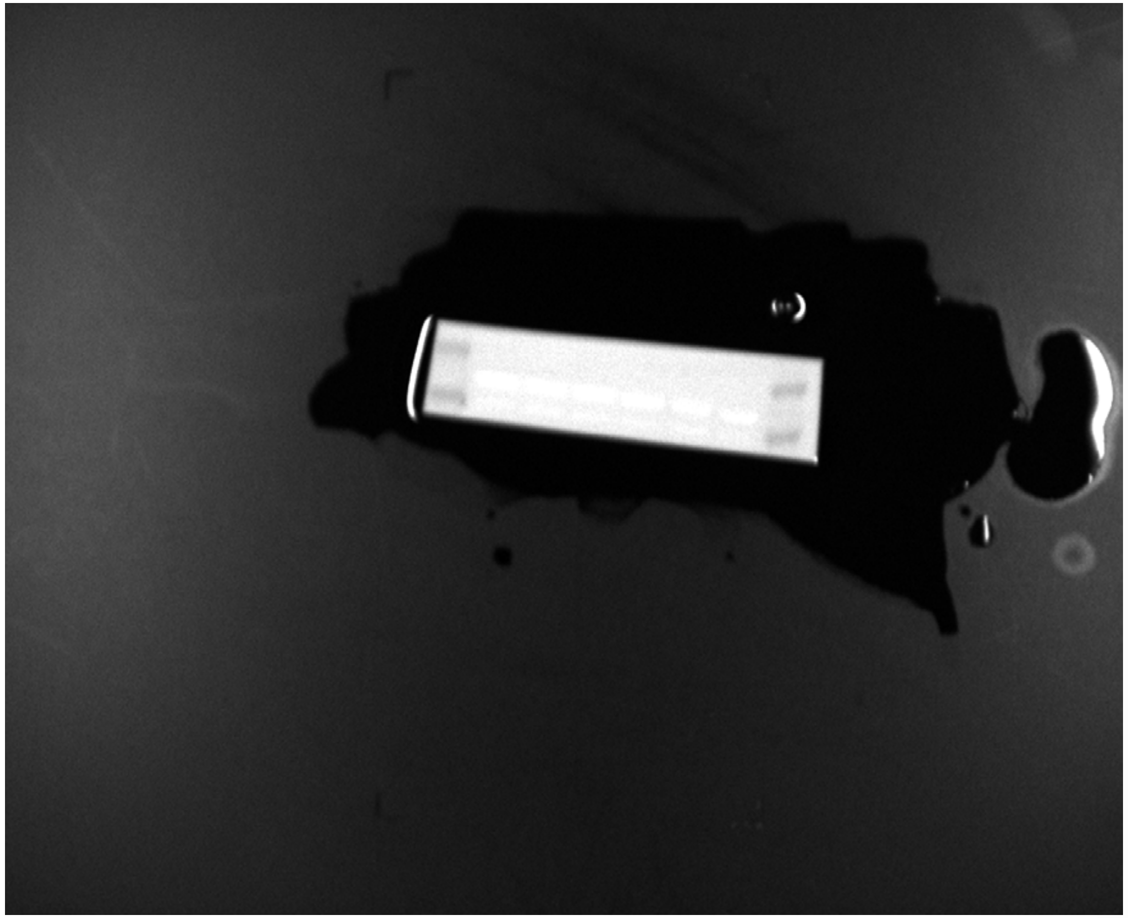

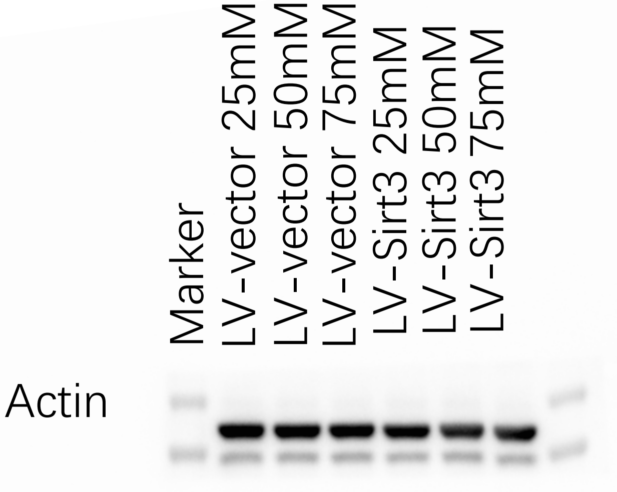
Marker
LV-vector 25mM
LV-vector 50mM
LV-vector 75mM
LV-Sirt3 25mM
LV-Sirt3 50mM
LV-Sirt3 75mM
Actin

Fig.5c

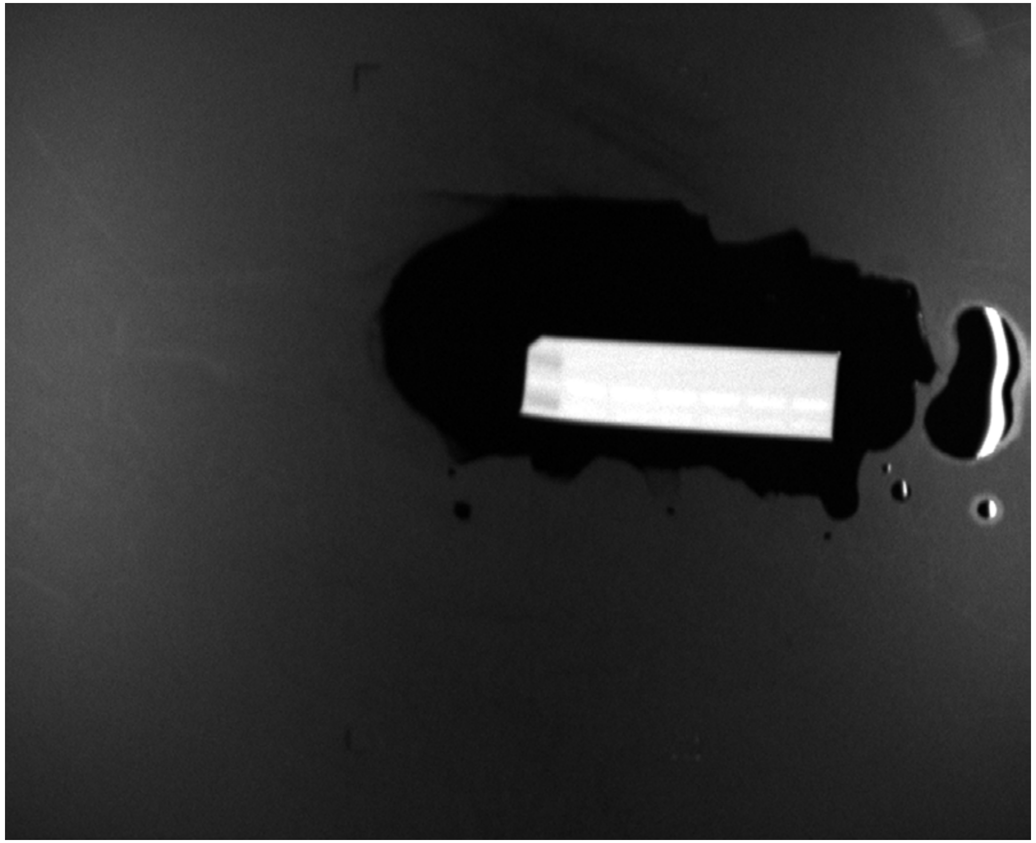

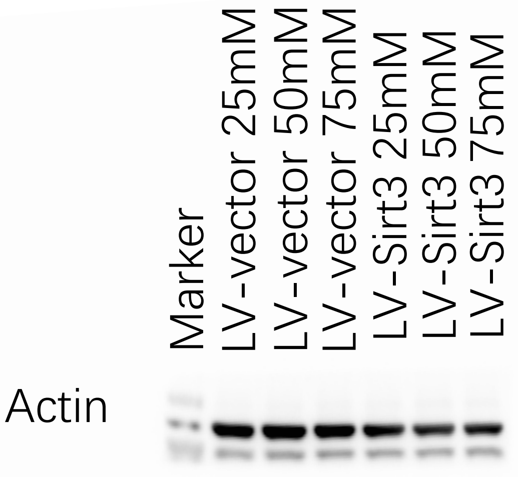
Marker
LV-vector 25mM
LV-vector 50mM
LV-vector 75mM
LV-Sirt3 25mM
LV-Sirt3 50mM
LV-Sirt3 75mM
Actin

Fig.5c

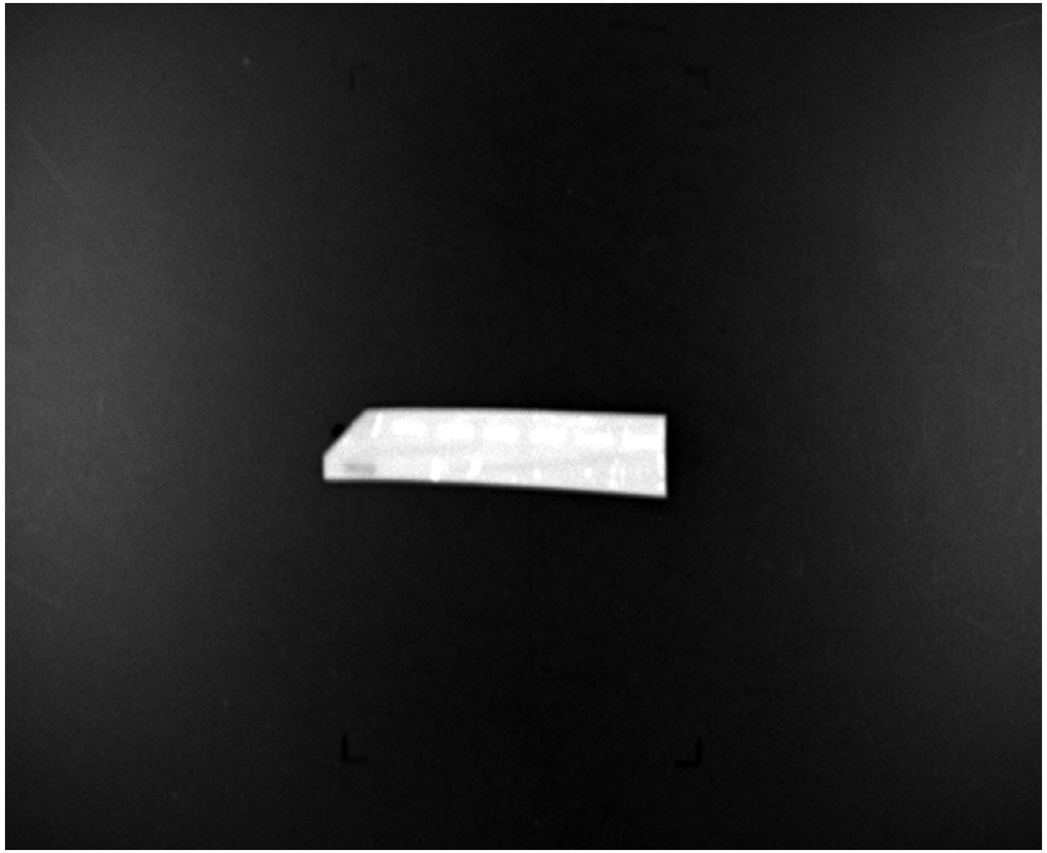

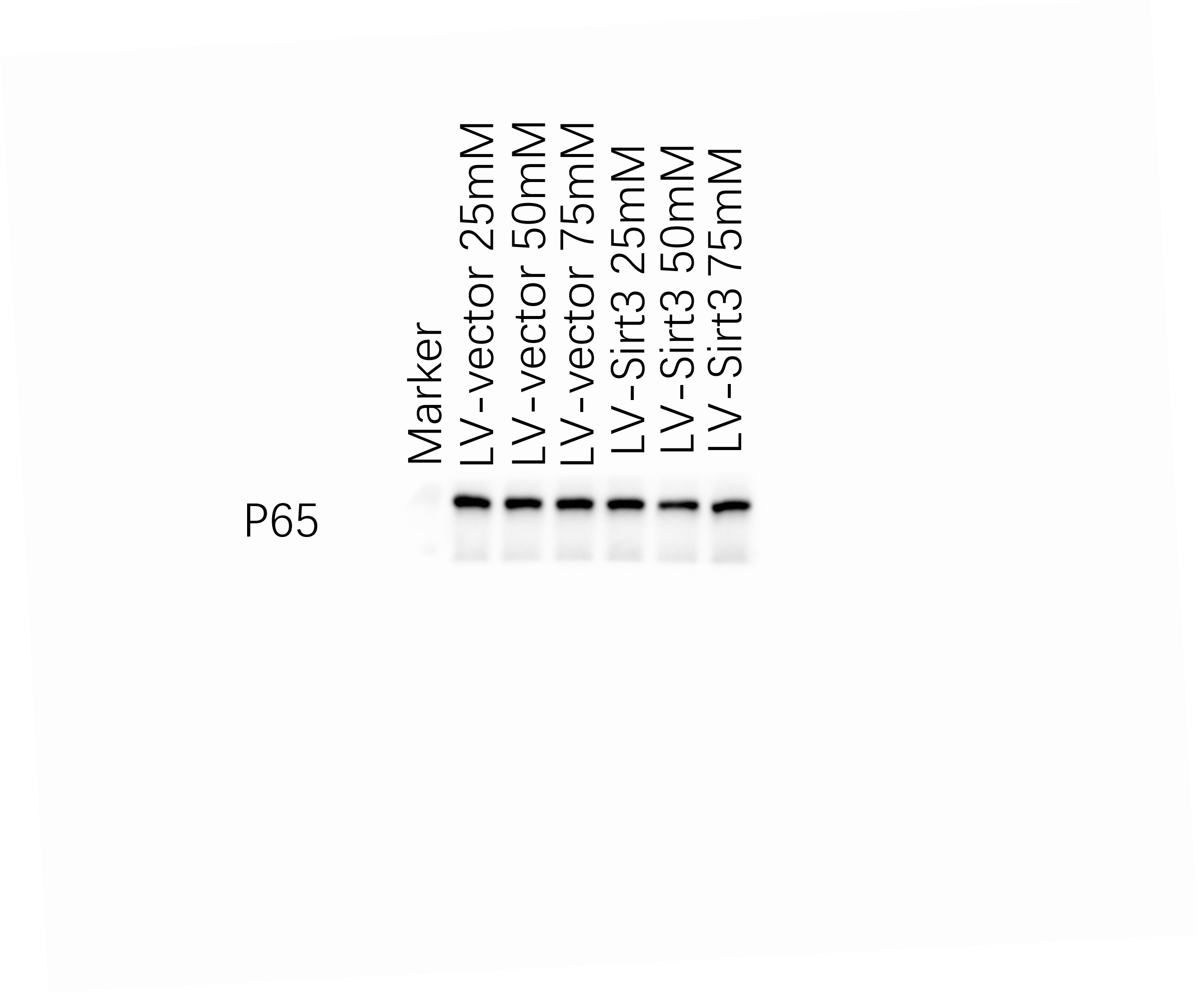
Marker
LV-vector 25mM
LV-vector 50mM
LV-vector 75mM
LV-Sirt3 25mM
LV-Sirt3 50mM
LV-Sirt3 75mM
P65

Fig.5c

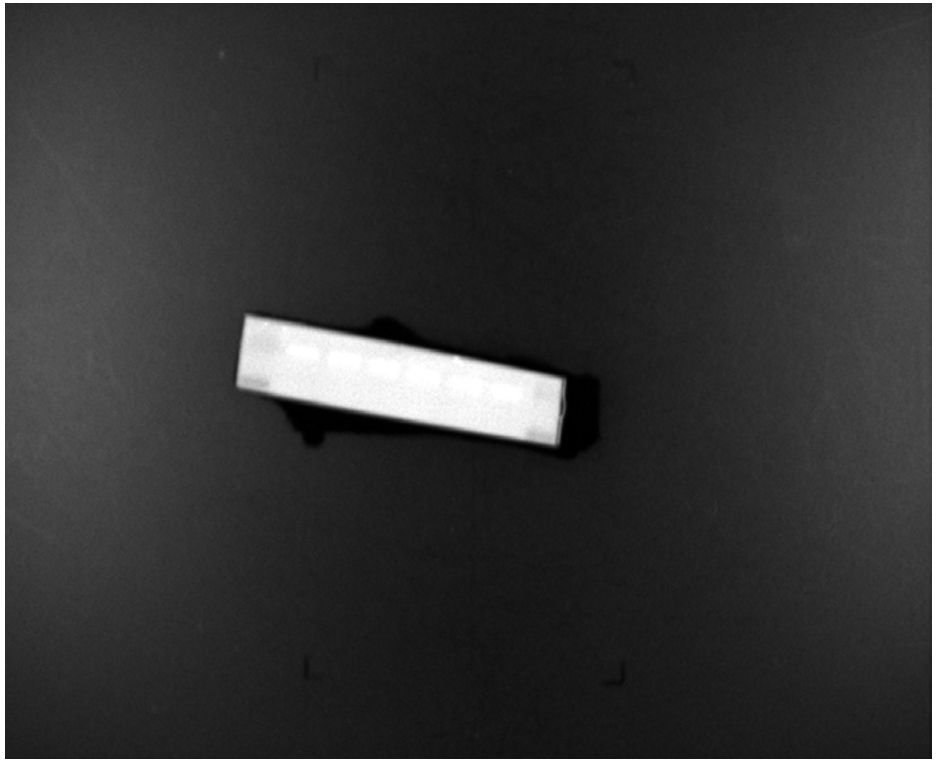

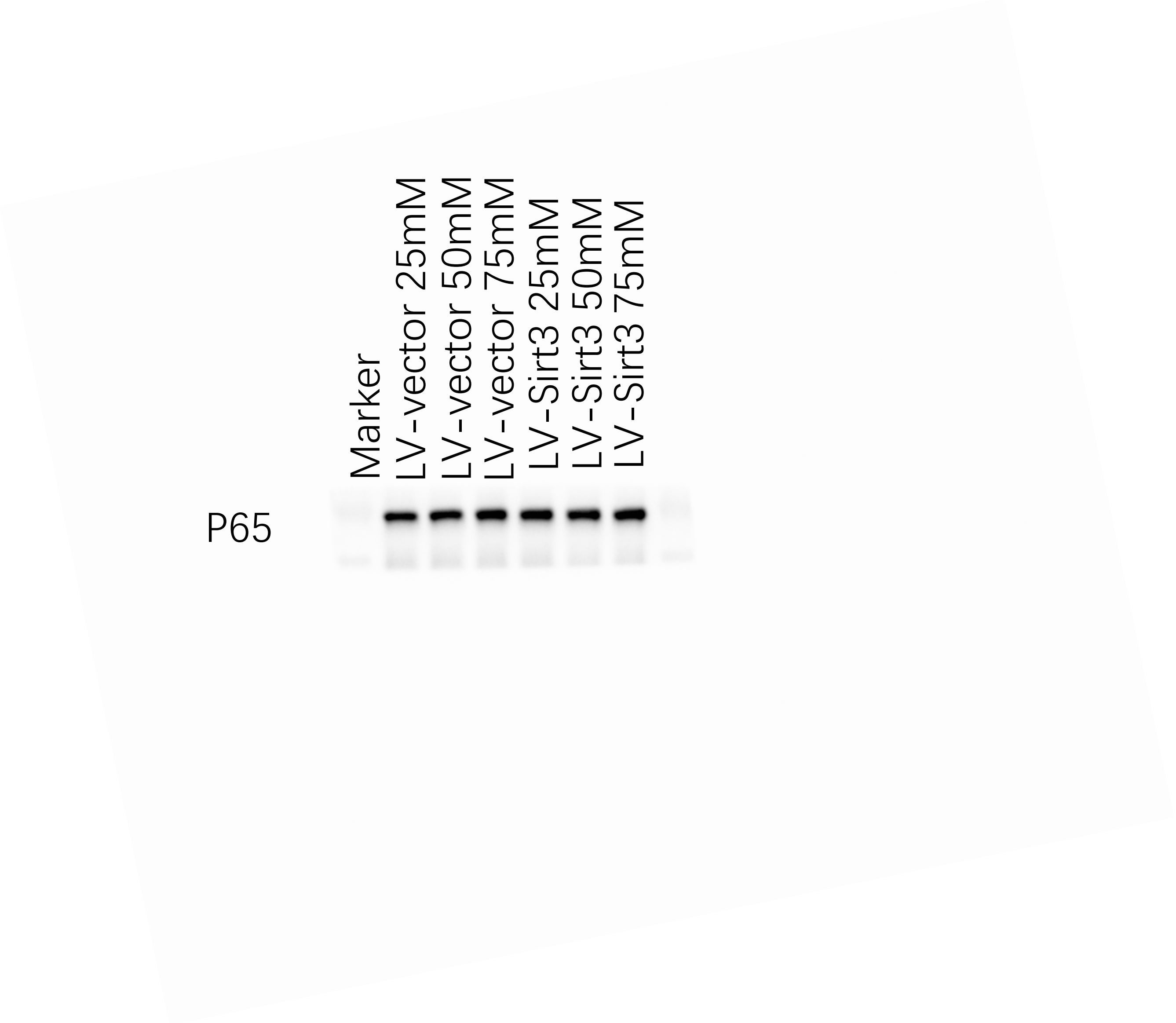
Marker
LV-vector 25mM
LV-vector 50mM
LV-vector 75mM
LV-Sirt3 25mM
LV-Sirt3 50mM
LV-Sirt3 75mM
P65

Fig.5c

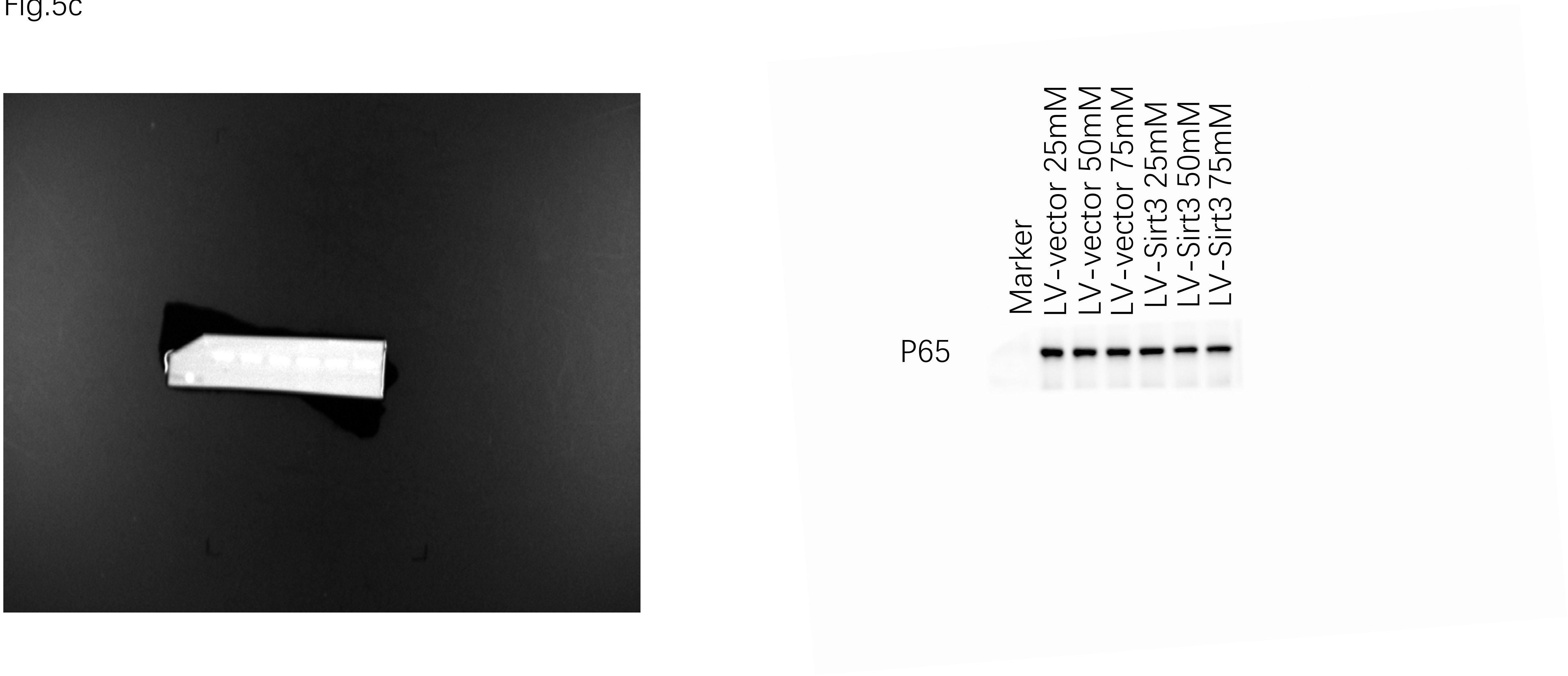
Marker
LV-vector 25mM
LV-vector 50mM
LV-vector 75mM
LV-Sirt3 25mM
LV-Sirt3 50mM
LV-Sirt3 75mM
P65

Fig.5c

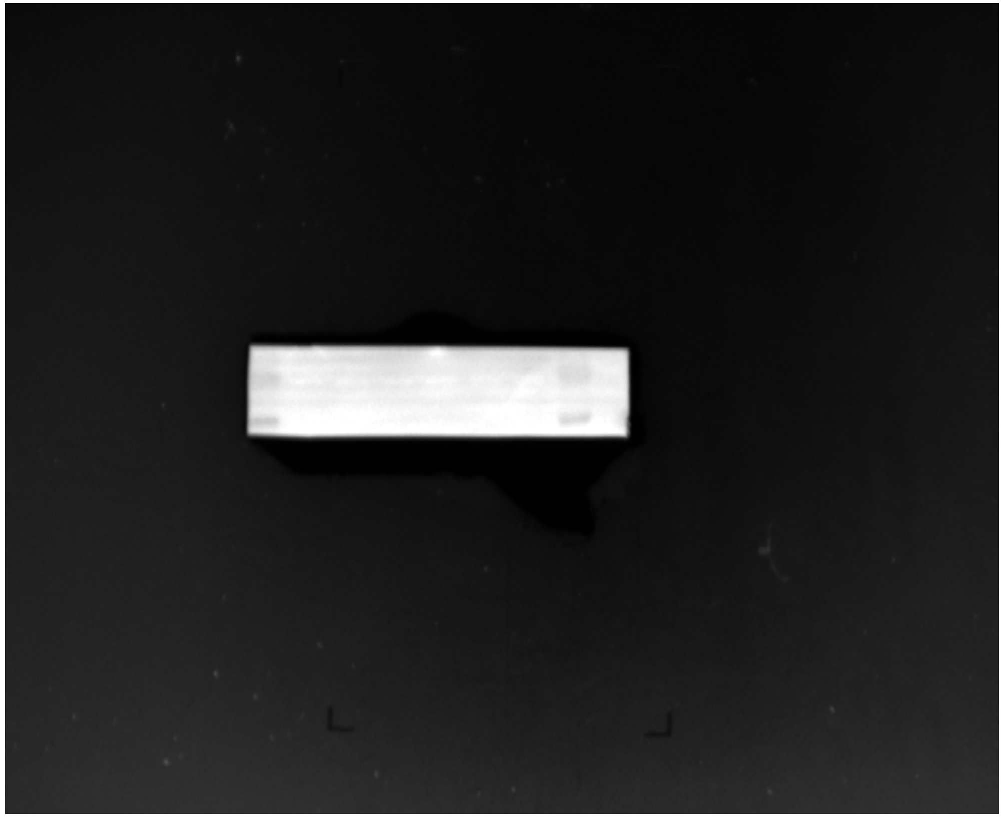

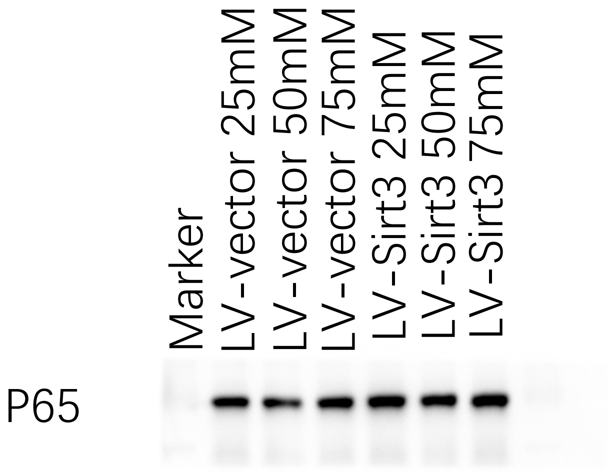
Marker
LV-vector 25mM
LV-vector 50mM
LV-vector 75mM
LV-Sirt3 25mM
LV-Sirt3 50mM
LV-Sirt3 75mM
P65

Fig.5c

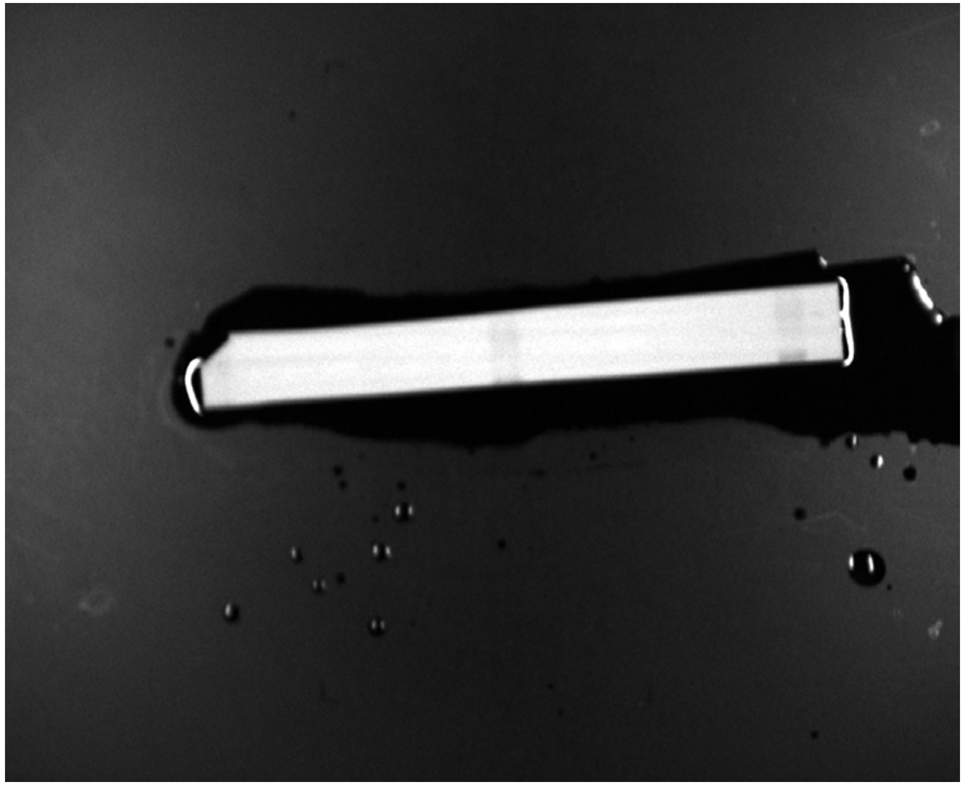

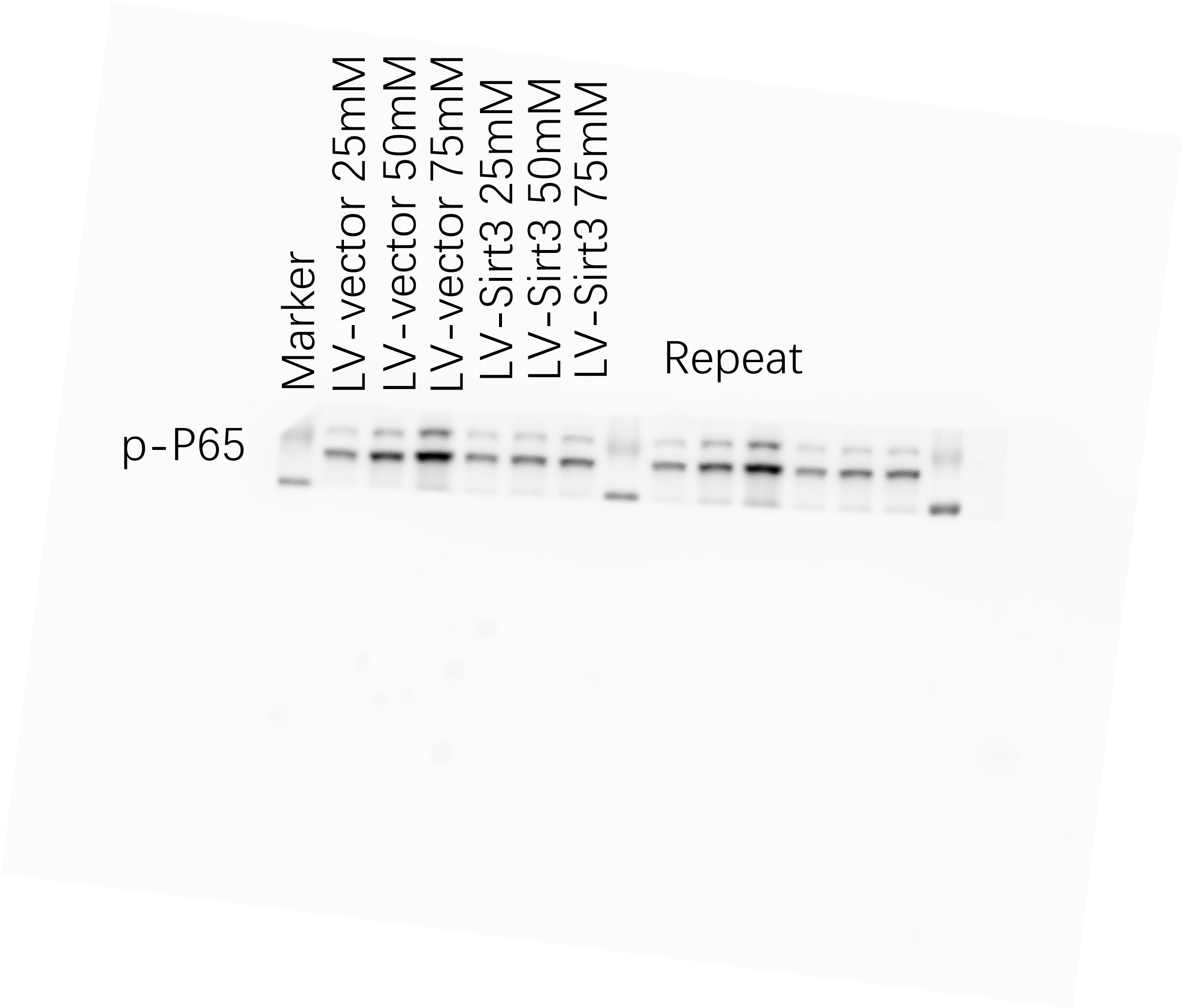
Marker
LV-vector 25mM
LV-vector 50mM
LV-vector 75mM
LV-Sirt3 25mM
LV-Sirt3 50mM
LV-Sirt3 75mM
Repeat
p-P65

Fig.5c

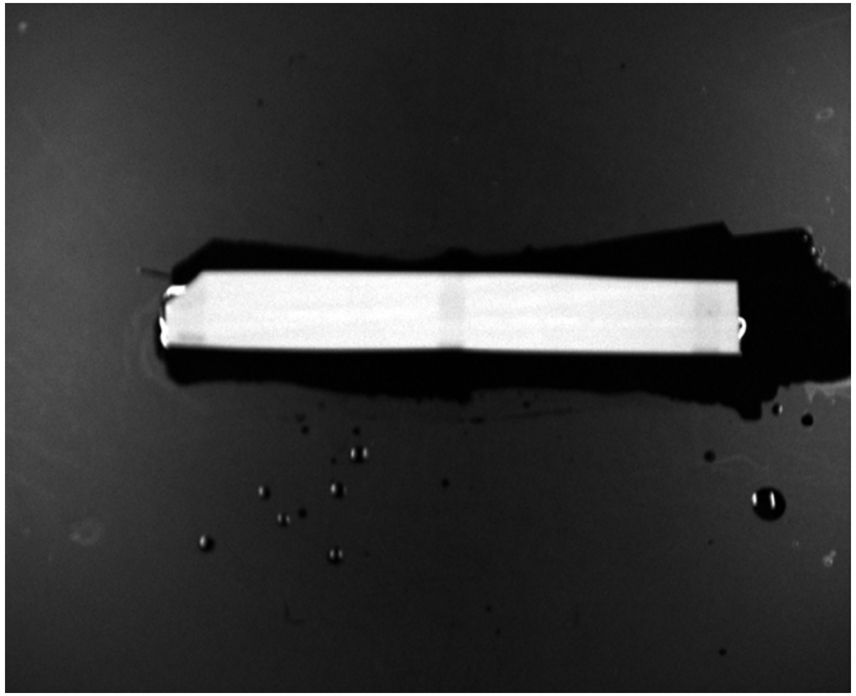

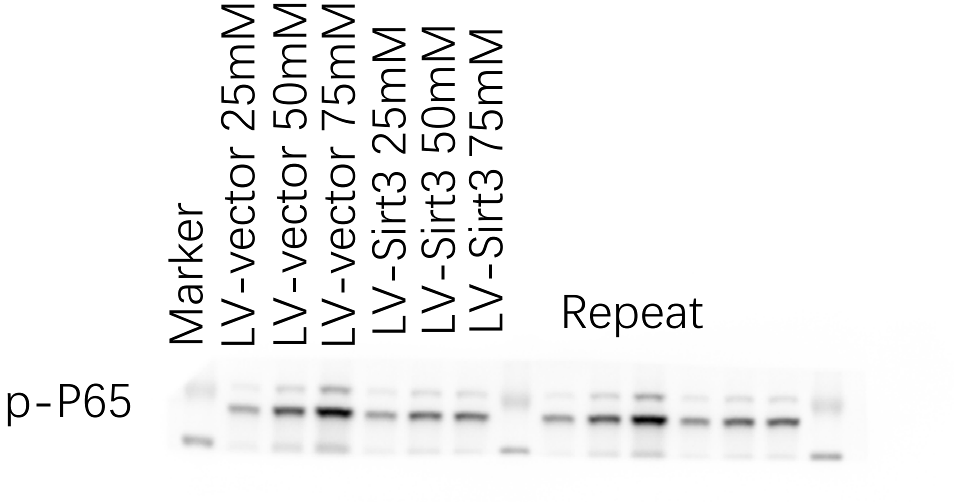
Marker
LV-vector 25mM
LV-vector 50mM
LV-vector 75mM
LV-Sirt3 25mM
LV-Sirt3 50mM
LV-Sirt3 75mM
Repeat
p-P65

Fig.5d

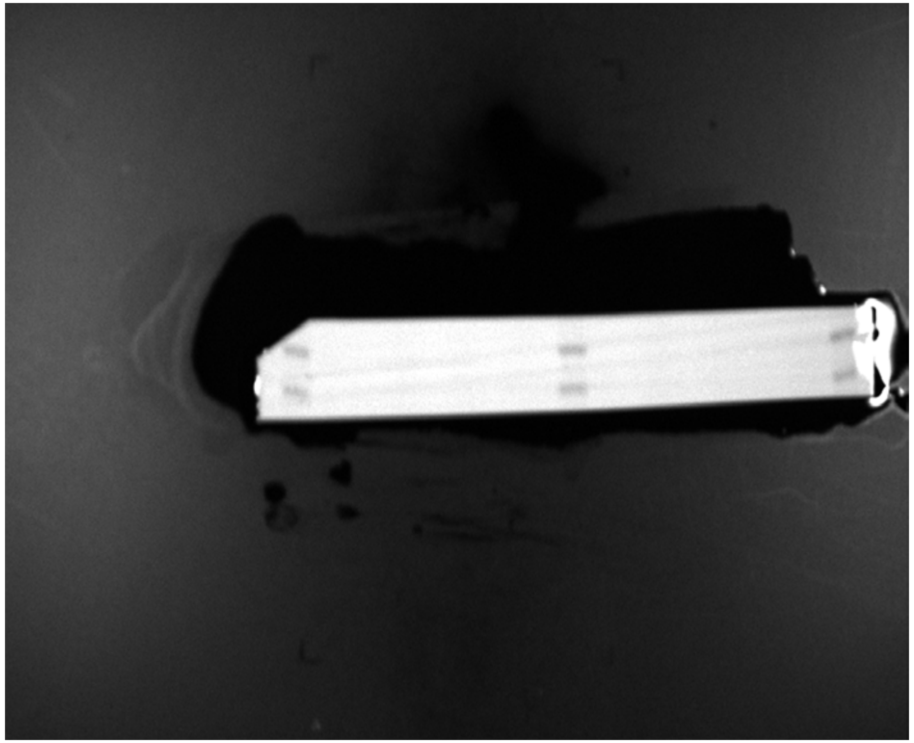

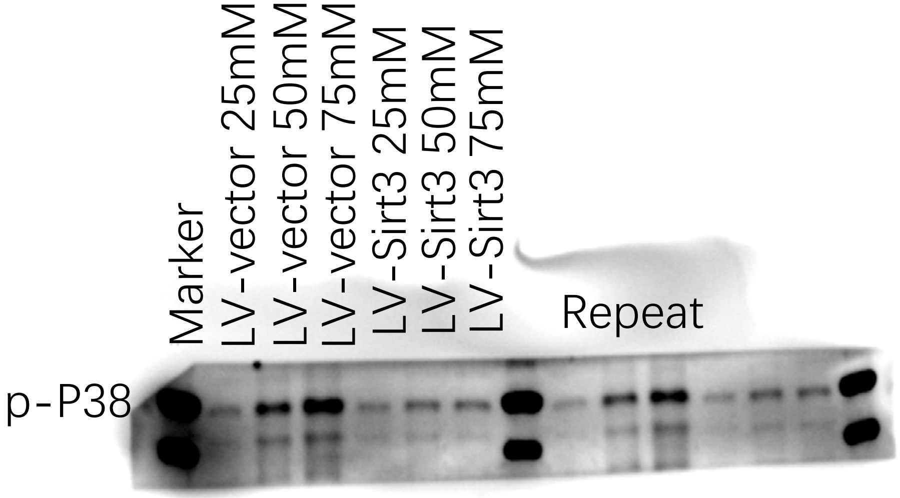
Marker
LV-vector 25mM
LV-vector 50mM
LV-vector 75mM
LV-Sirt3 25mM
LV-Sirt3 50mM
LV-Sirt3 75mM
Repeat
p-P38

Fig.5d

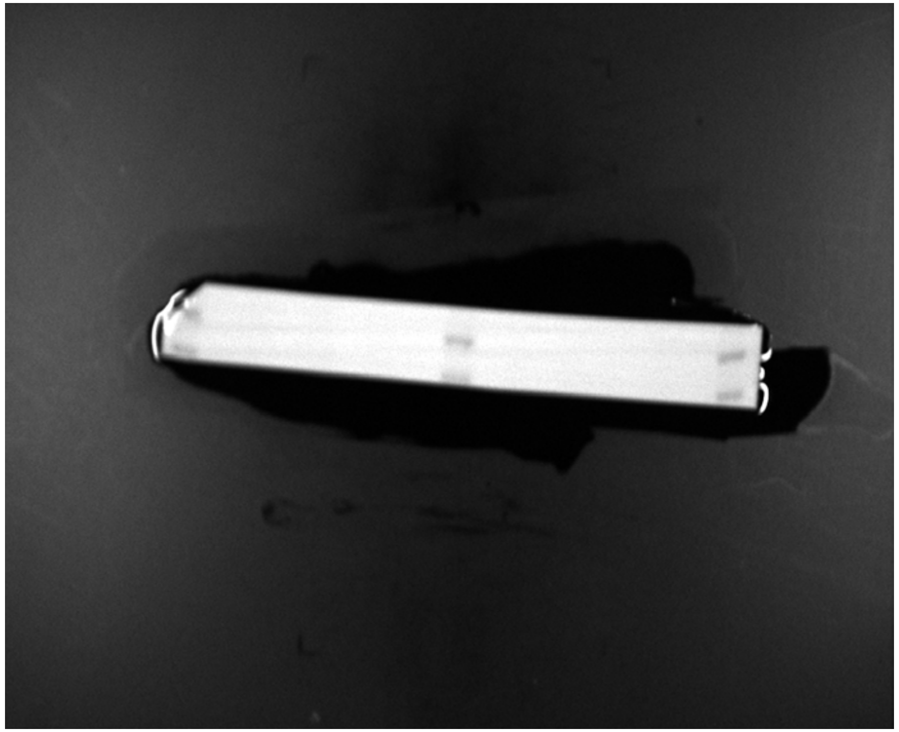

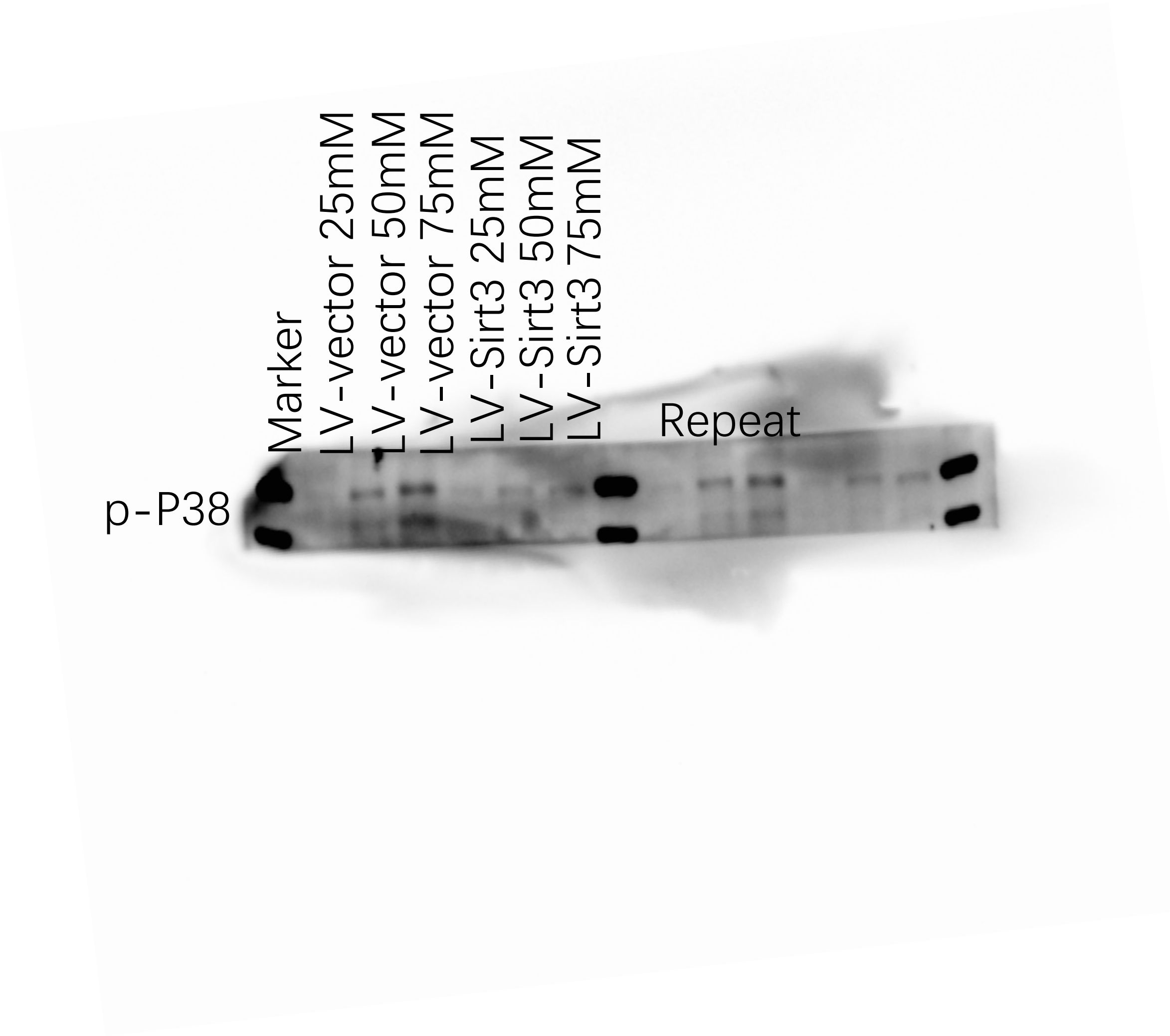
Marker
LV-vector 25mM
LV-vector 50mM
LV-vector 75mM
LV-Sirt3 25mM
LV-Sirt3 50mM
LV-Sirt3 75mM
Repeat
p-P38

Fig.5d

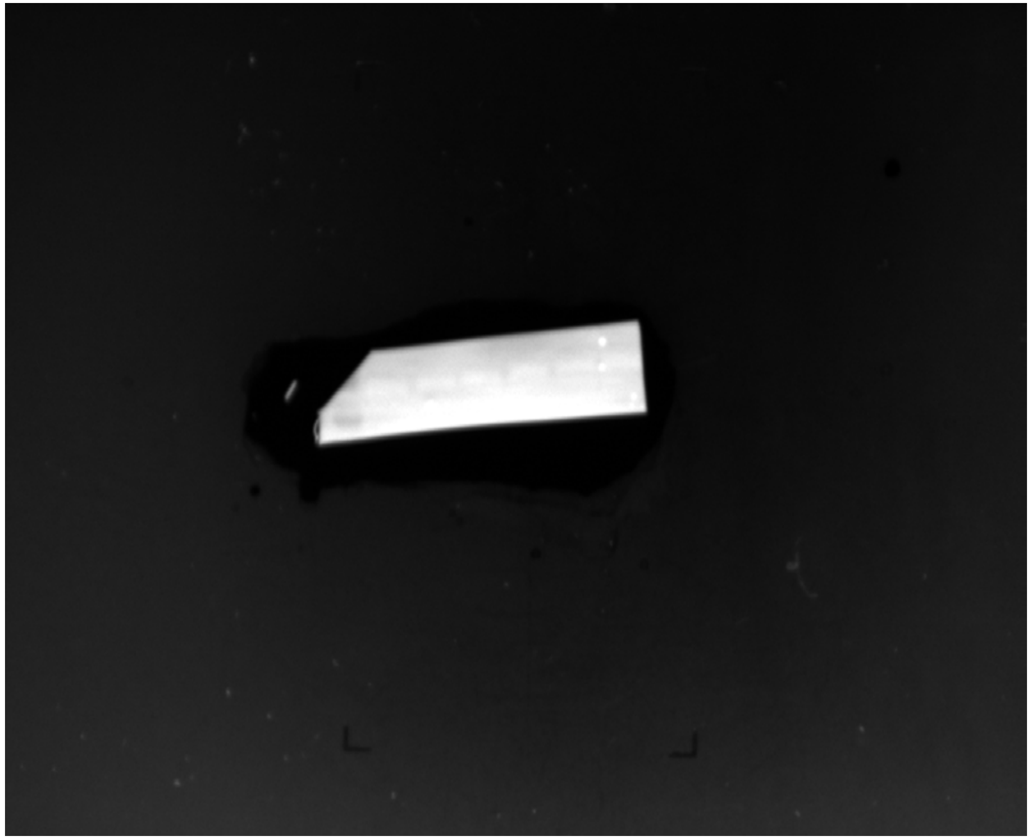

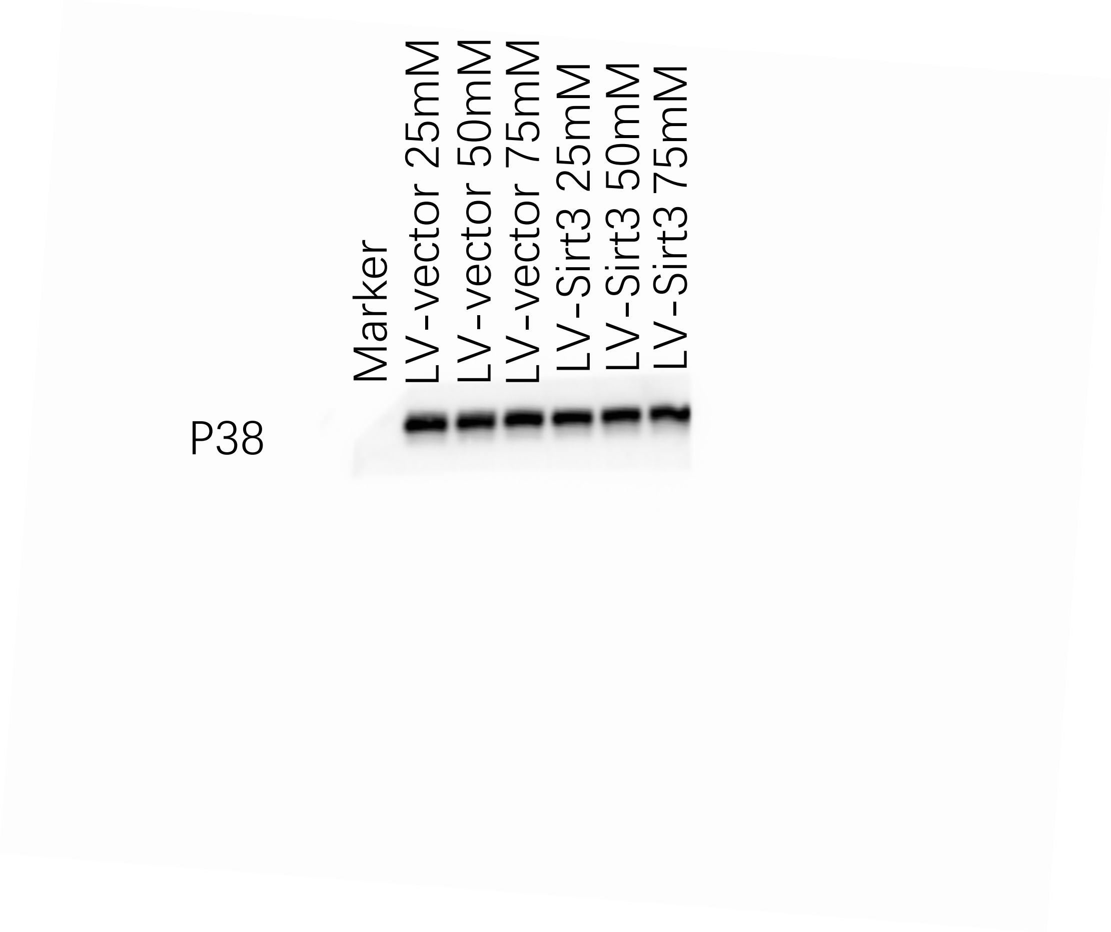
Marker
LV-vector 25mM
LV-vector 50mM
LV-vector 75mM
LV-Sirt3 25mM
LV-Sirt3 50mM
LV-Sirt3 75mM
P38

Fig.5d

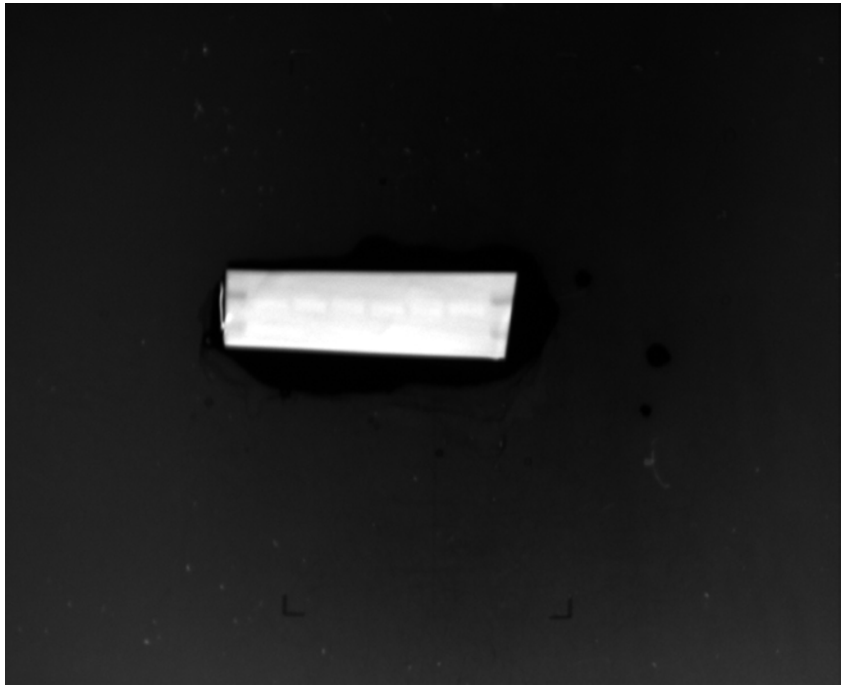

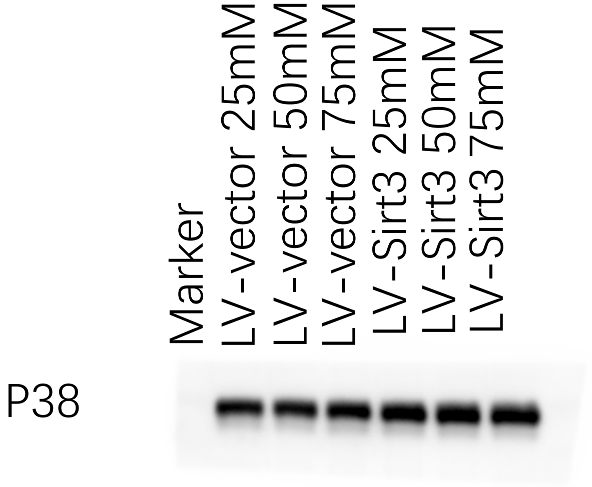
Marker
LV-vector 25mM
LV-vector 50mM
LV-vector 75mM
LV-Sirt3 25mM
LV-Sirt3 50mM
LV-Sirt3 75mM
P38

Fig.5d

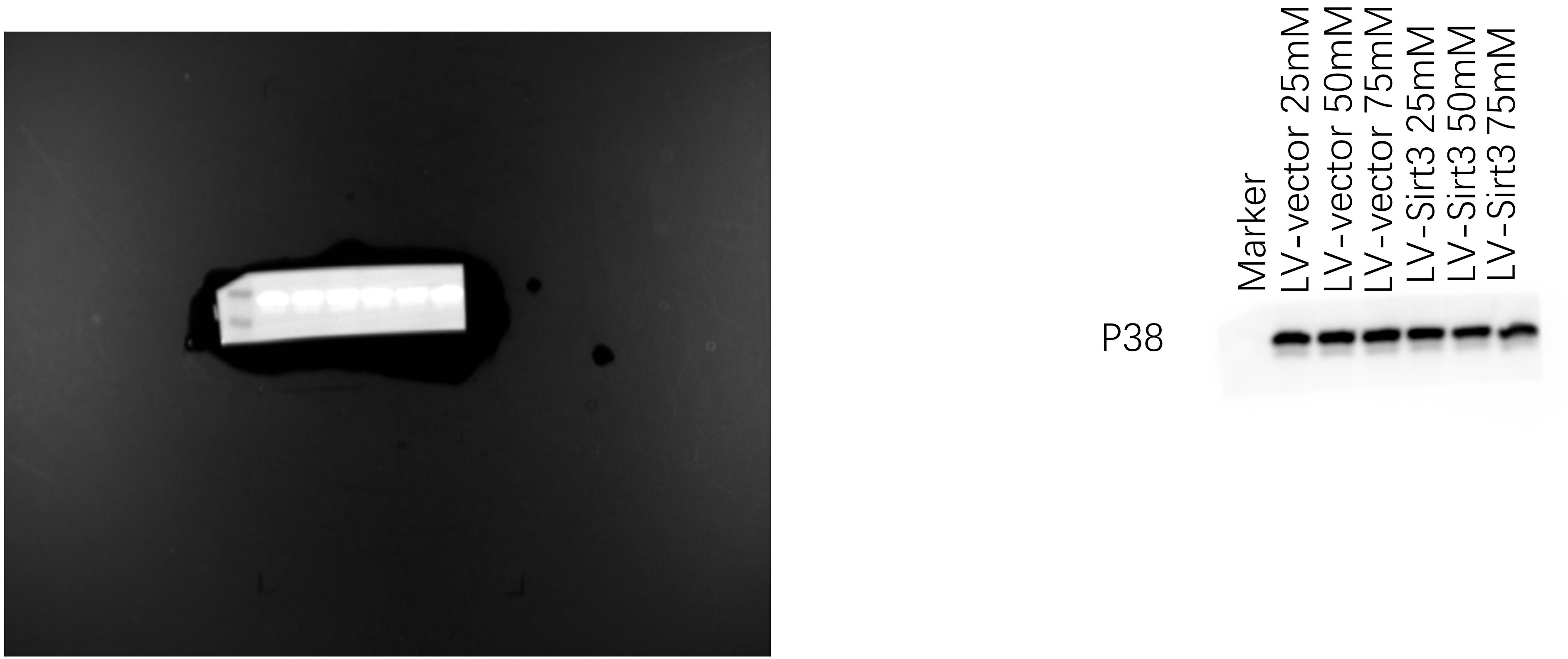
Marker
LV-vector 25mM
LV-vector 50mM
LV-vector 75mM
LV-Sirt3 25mM
LV-Sirt3 50mM
LV-Sirt3 75mM
P38

Fig.5d

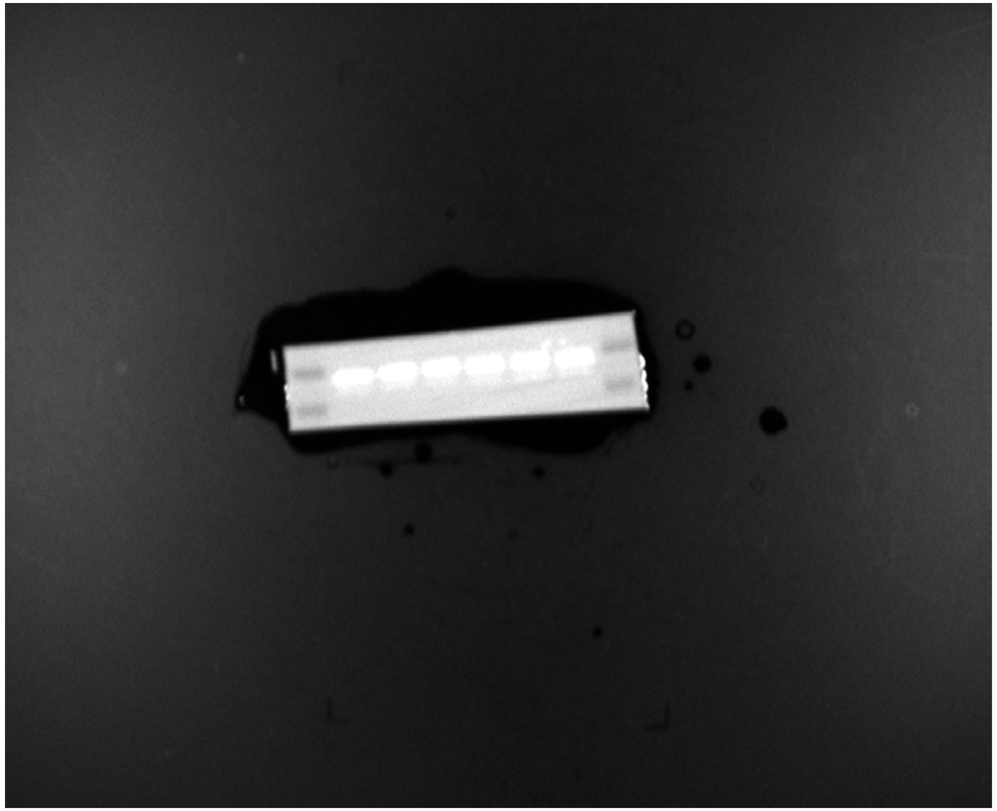

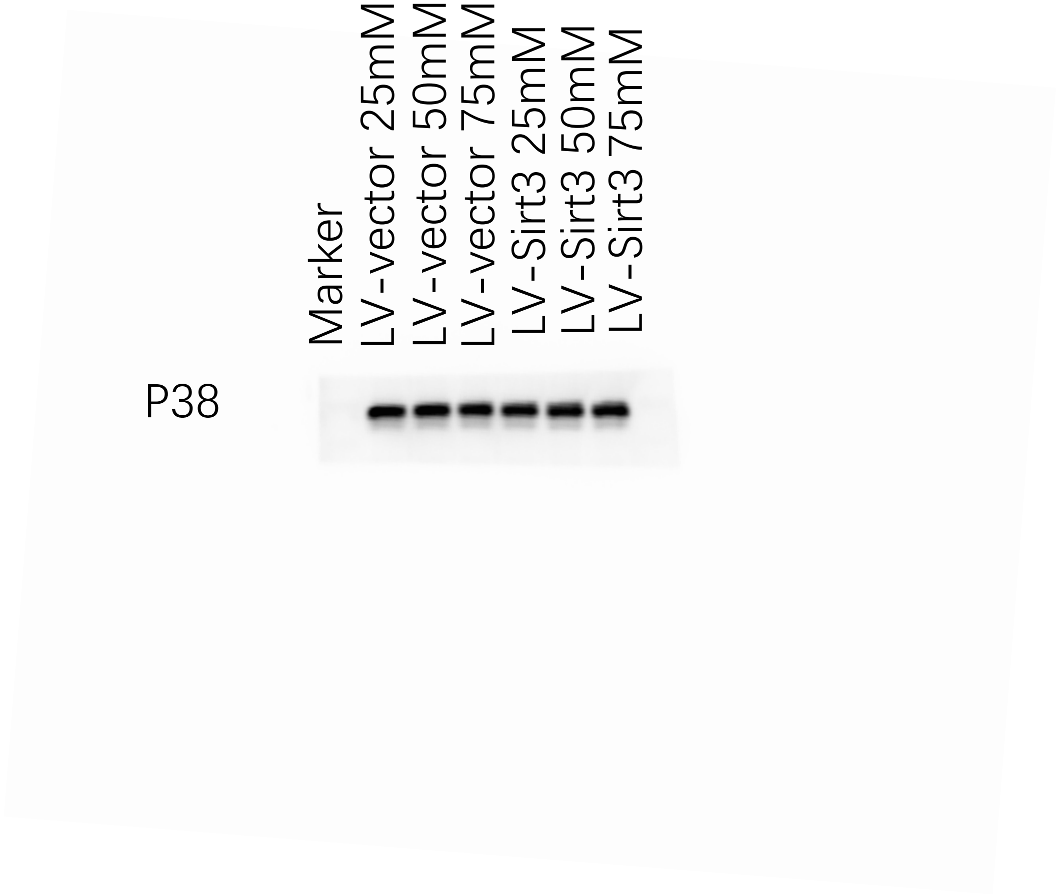
Marker
LV-vector 25mM
LV-vector 50mM
LV-vector 75mM
LV-Sirt3 25mM
LV-Sirt3 50mM
LV-Sirt3 75mM
P38

Fig.5d

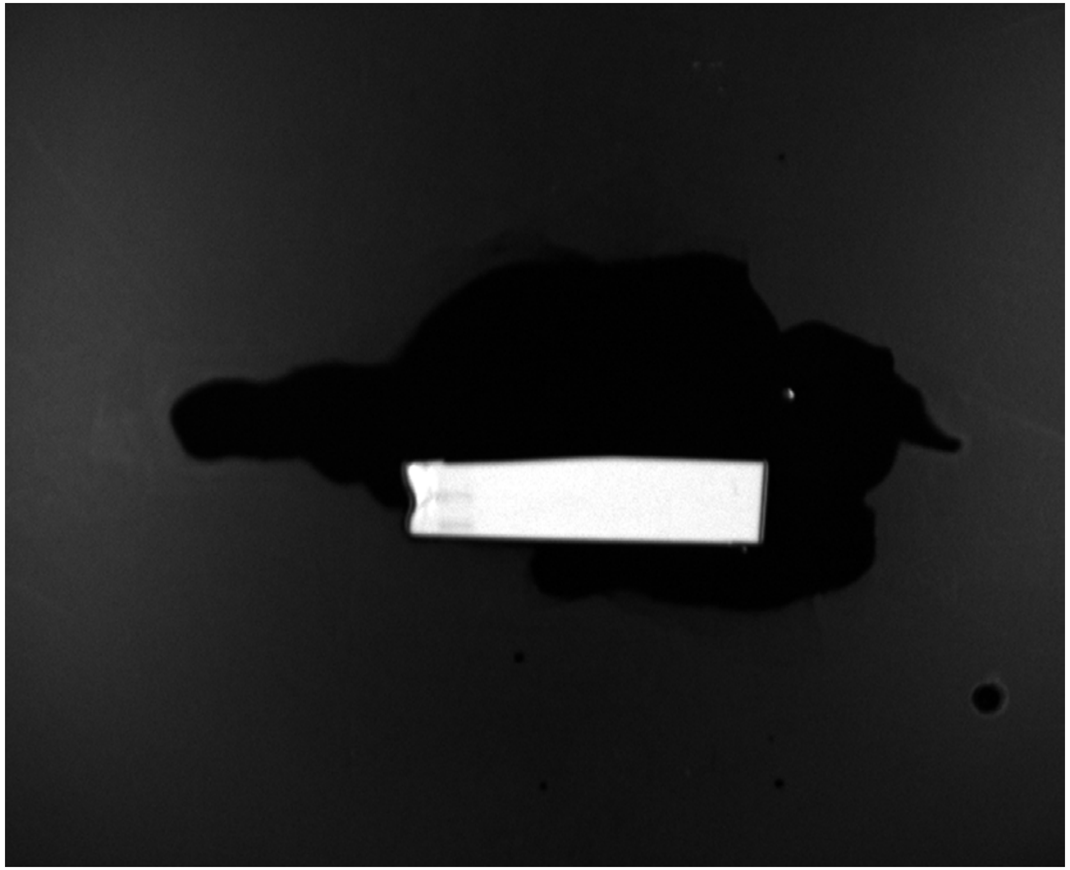

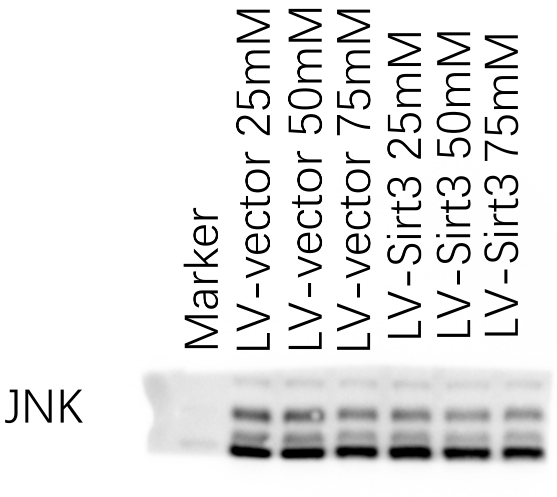
Marker
LV-vector 25mM
LV-vector 50mM
LV-vector 75mM
LV-Sirt3 25mM
LV-Sirt3 50mM
LV-Sirt3 75mM
JNK

Fig.5d

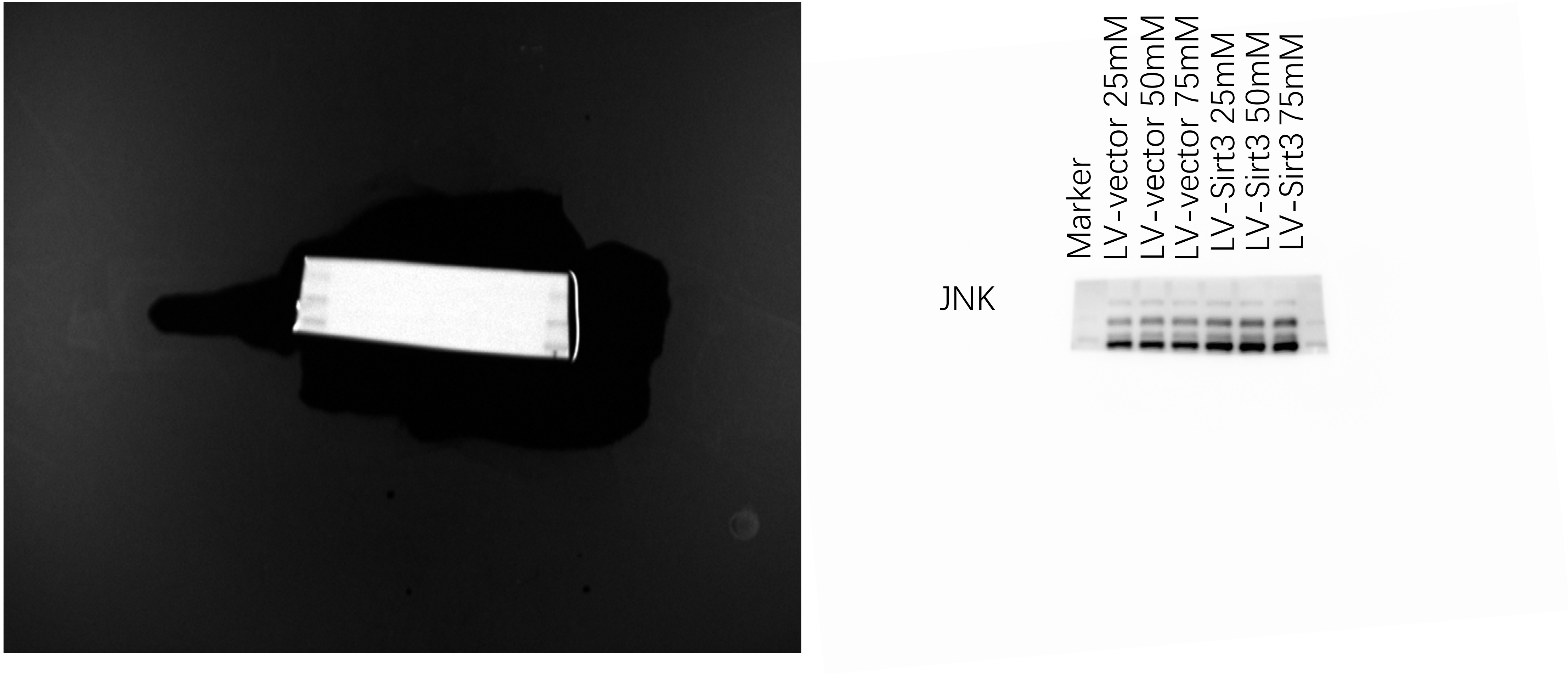
Marker
LV-vector 25mM
LV-vector 50mM
LV-vector 75mM
LV-Sirt3 25mM
LV-Sirt3 50mM
LV-Sirt3 75mM
JNK

Fig.5d

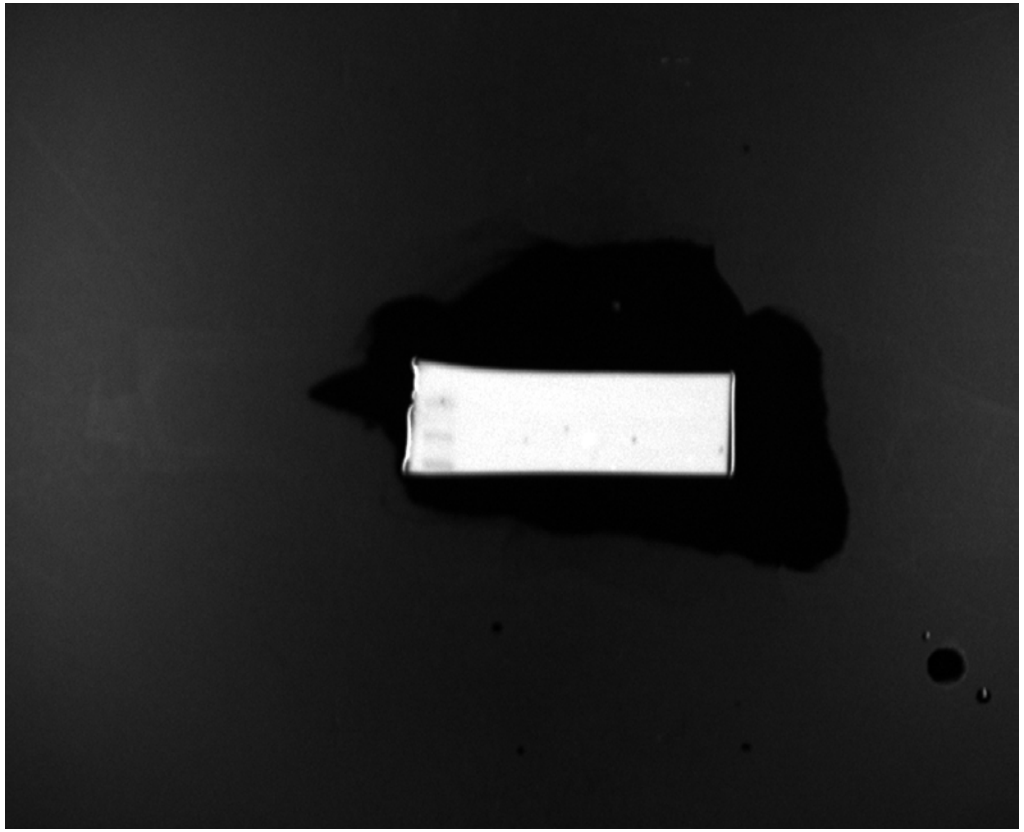

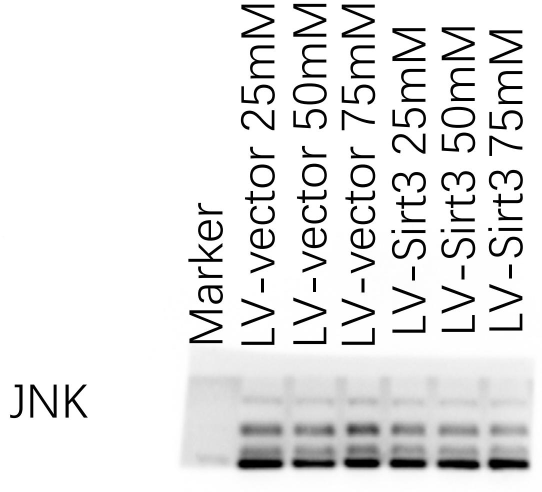
Marker
LV-vector 25mM
LV-vector 50mM
LV-vector 75mM
LV-Sirt3 25mM
LV-Sirt3 50mM
LV-Sirt3 75mM
JNK

Fig.5d

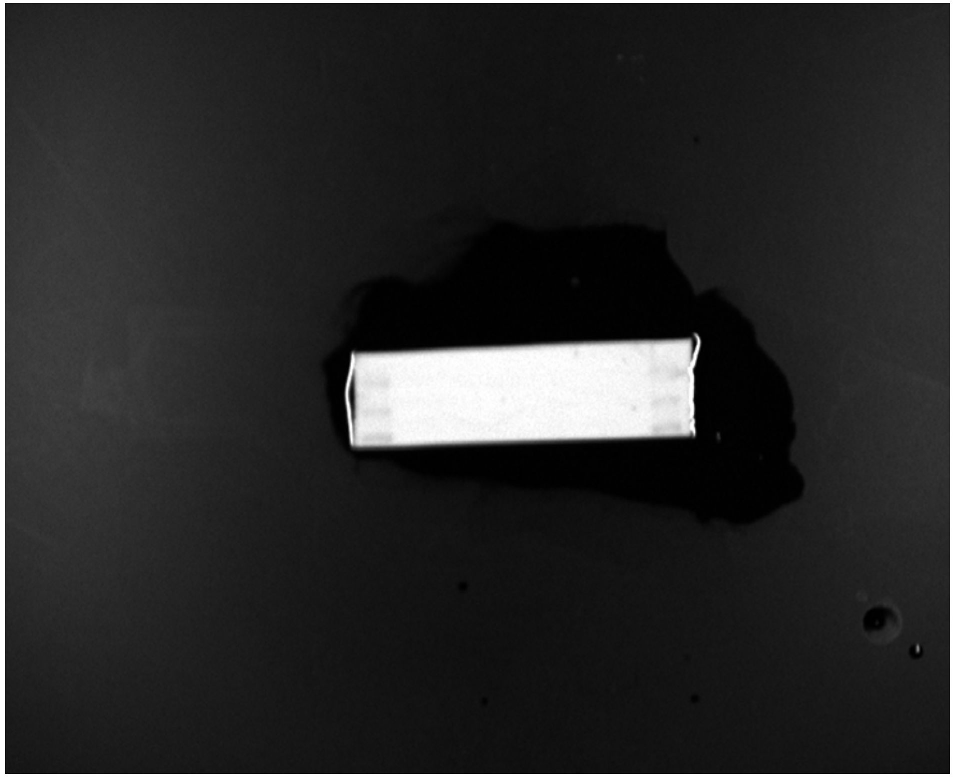

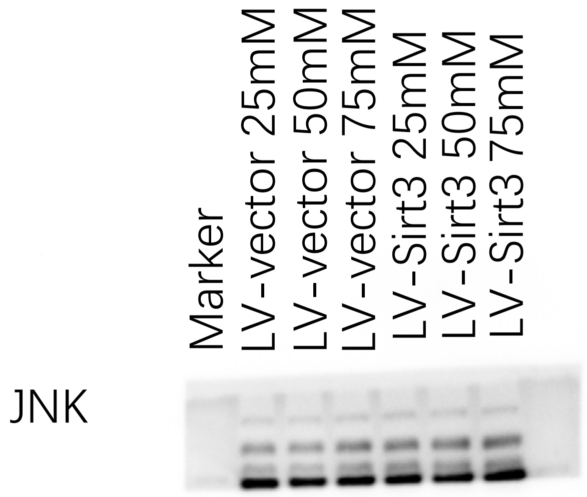
JNK
Marker
LV-vector 25mM
LV-vector 50mM
LV-vector 75mM
LV-Sirt3 25mM
LV-Sirt3 50mM
LV-Sirt3 75mM

Fig.5d

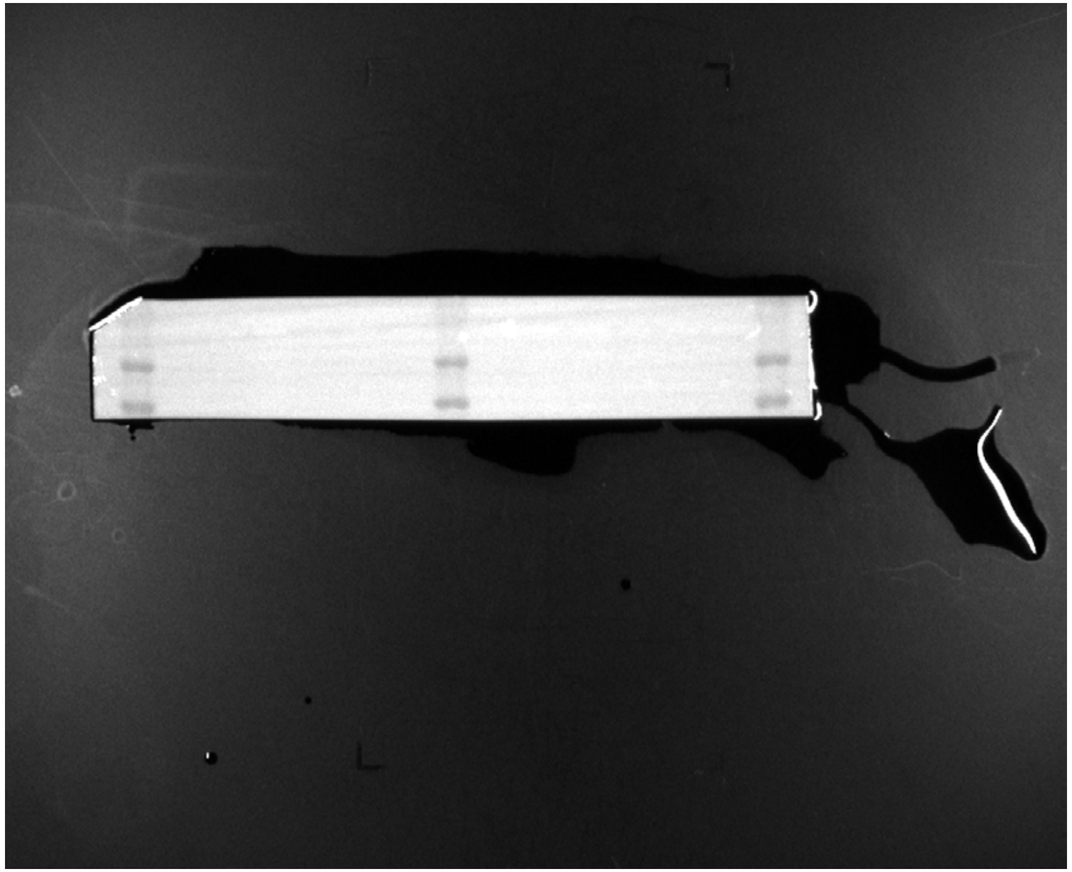

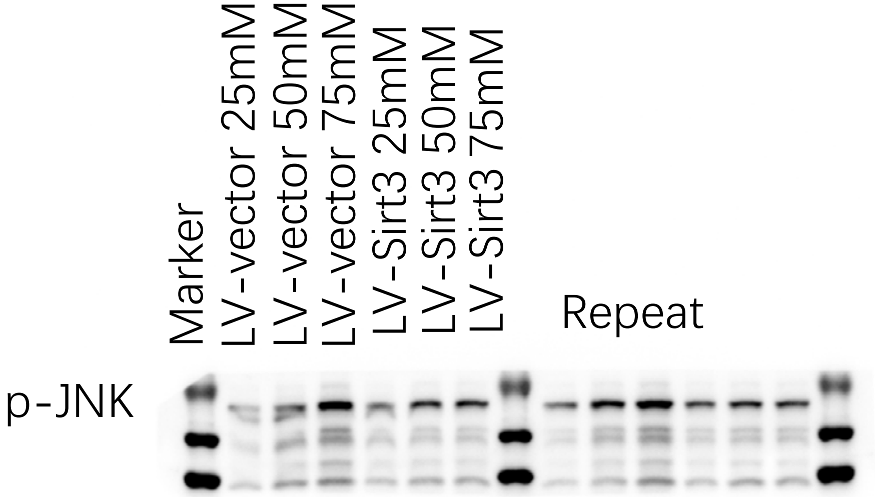
Marker
LV-vector 25mM
LV-vector 50mM
LV-vector 75mM
LV-Sirt3 25mM
LV-Sirt3 50mM
LV-Sirt3 75mM
Repeat
p-JNK

Fig.5d

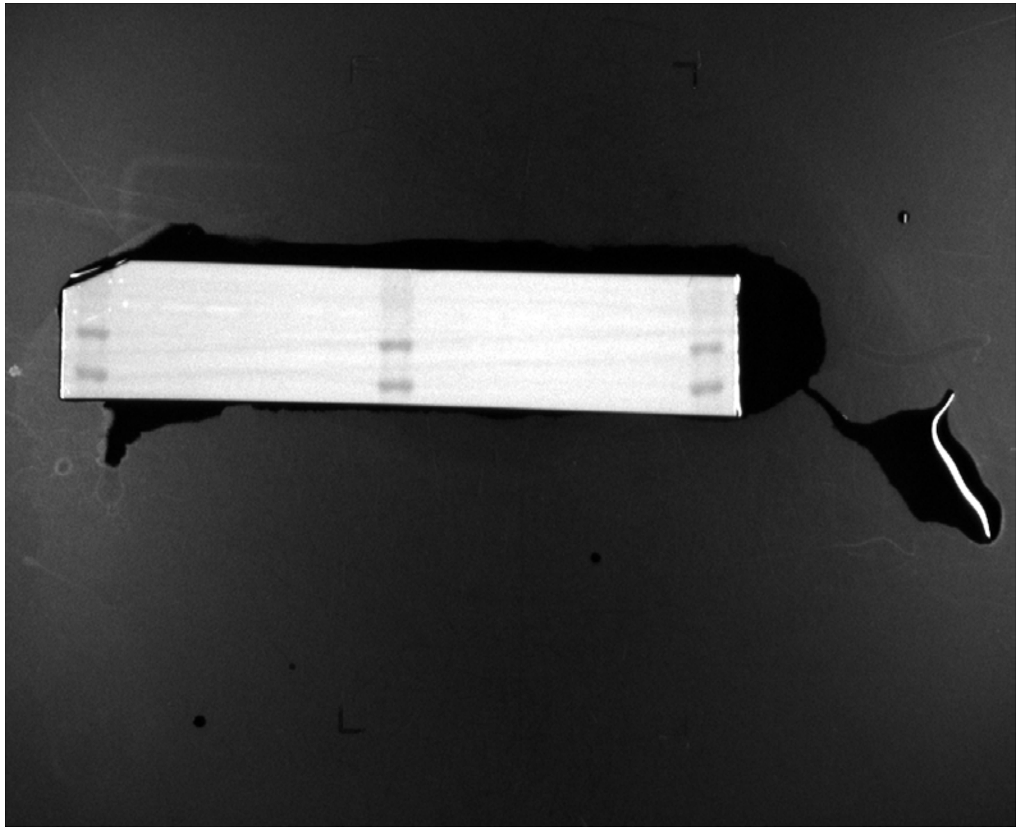

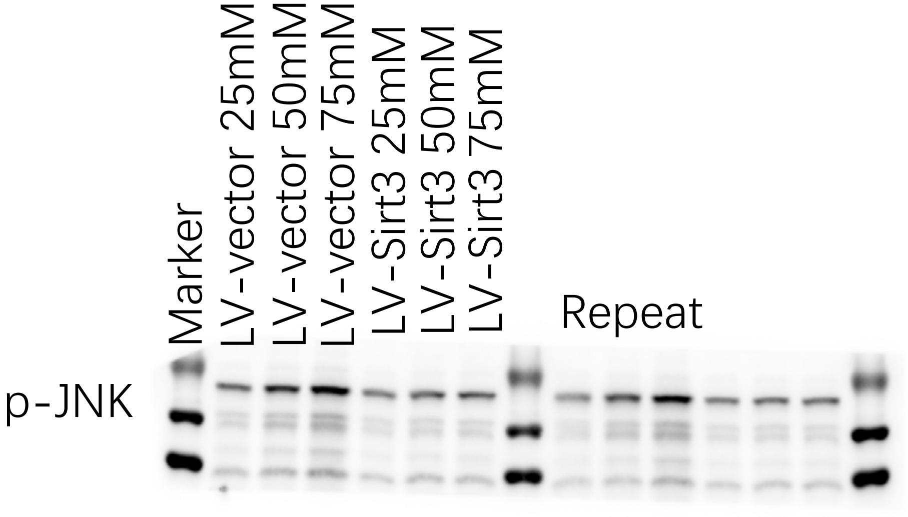
Marker
LV-vector 25mM
LV-vector 50mM
LV-vector 75mM
LV-Sirt3 25mM
LV-Sirt3 50mM
LV-Sirt3 75mM
Repeat
p-JNK

Fig.5d

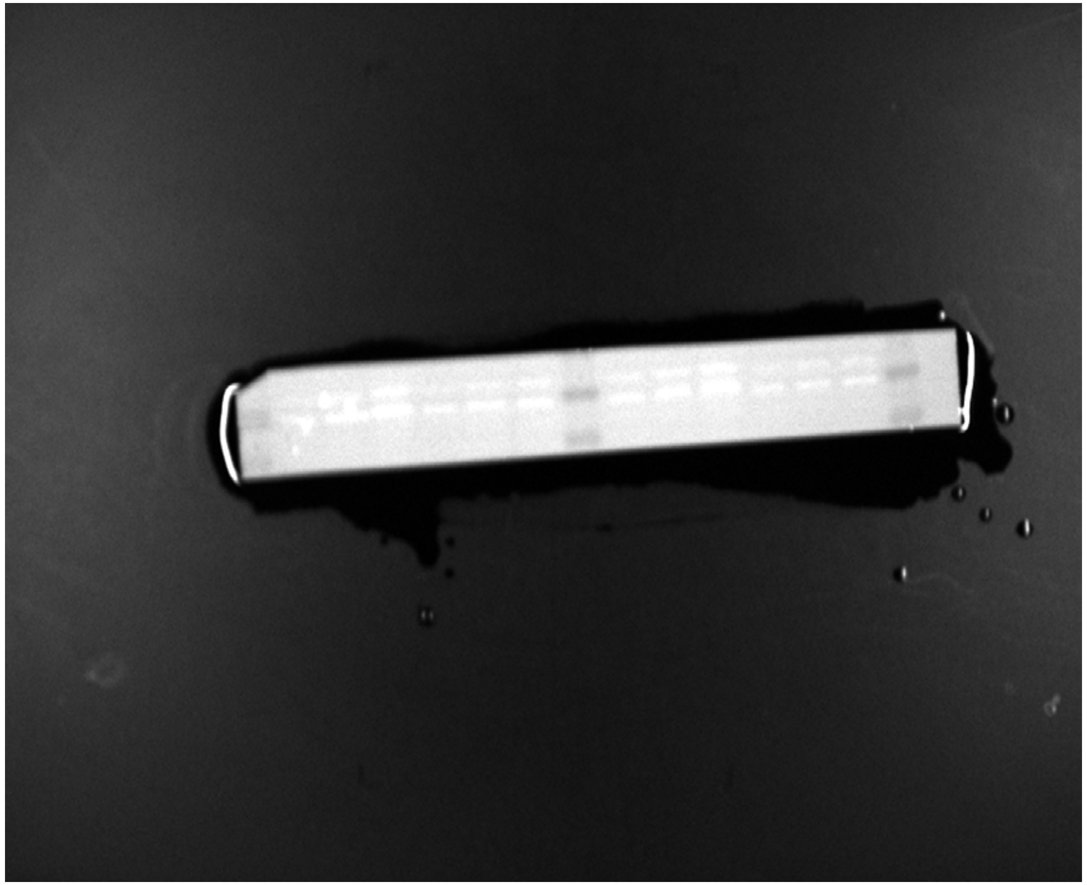

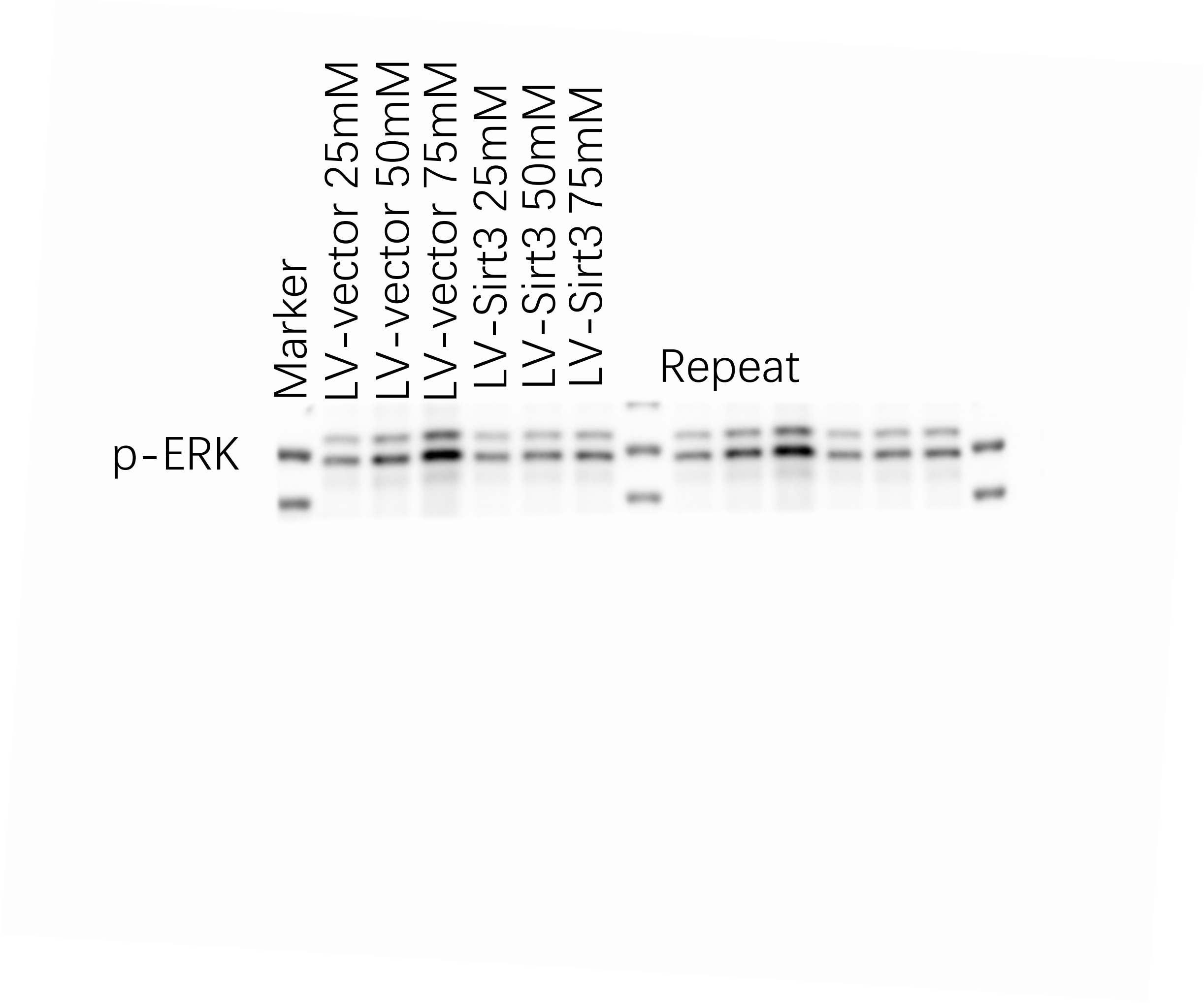
Marker
LV-vector 25mM
LV-vector 50mM
LV-vector 75mM
LV-Sirt3 25mM
LV-Sirt3 50mM
LV-Sirt3 75mM
Repeat
p-ERK

Fig.5d

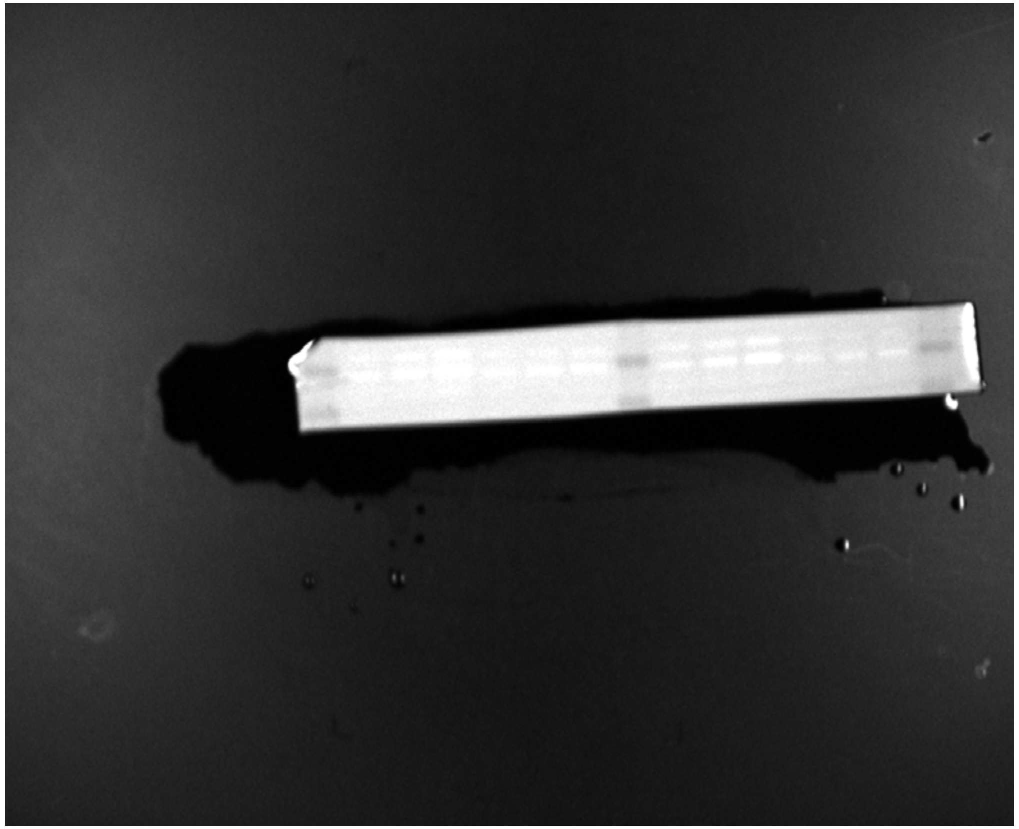

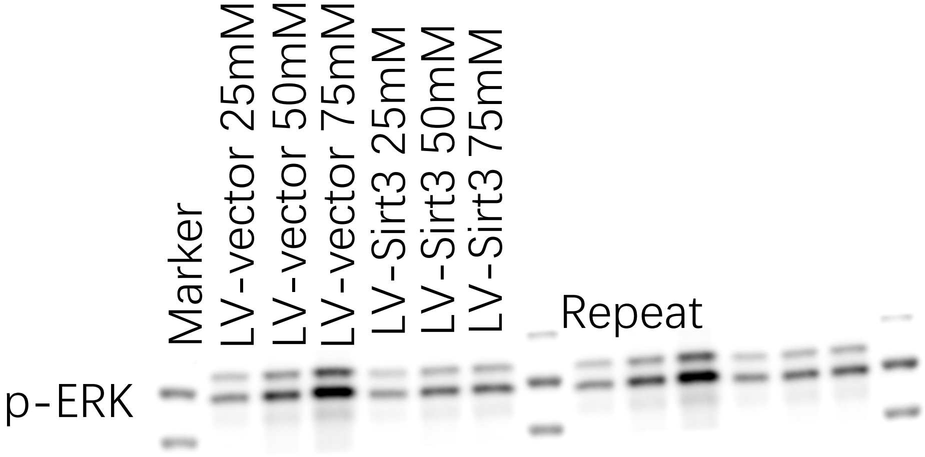
Marker
LV-vector 25mM
LV-vector 50mM
LV-vector 75mM
LV-Sirt3 25mM
LV-Sirt3 50mM
LV-Sirt3 75mM
Repeat
p-ERK

Fig.5d

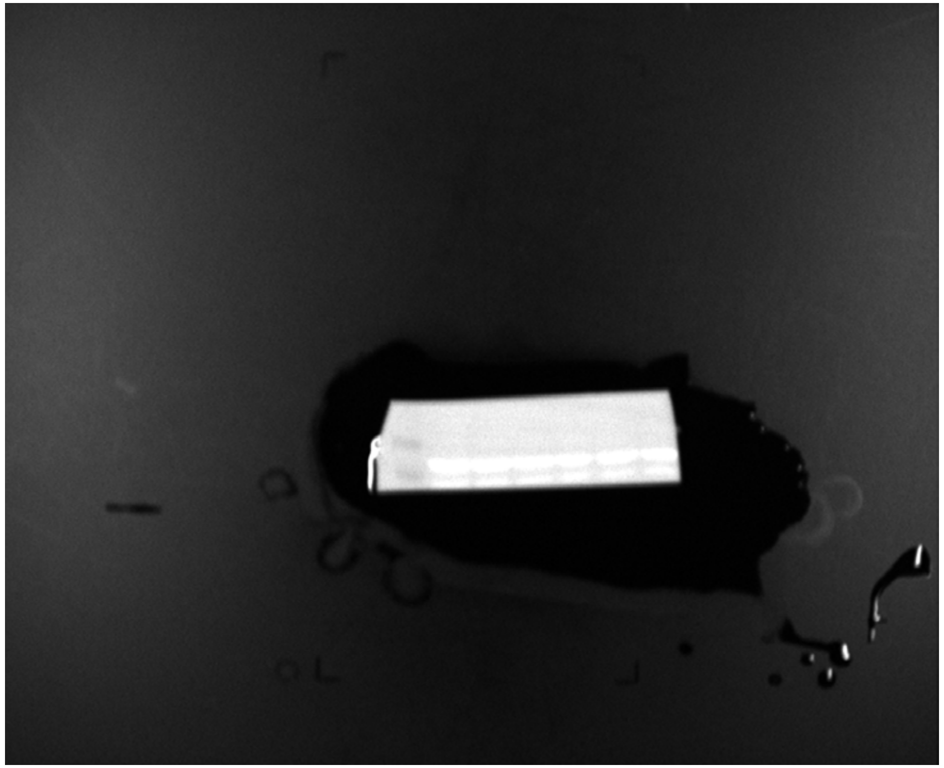

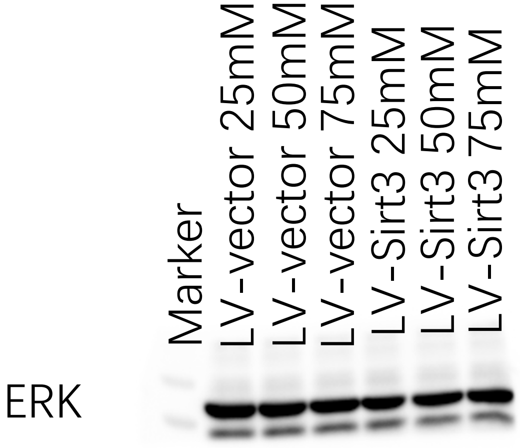
Marker
LV-vector 25mM
LV-vector 50mM
LV-vector 75mM
LV-Sirt3 25mM
LV-Sirt3 50mM
LV-Sirt3 75mM
ERK

Fig.5d

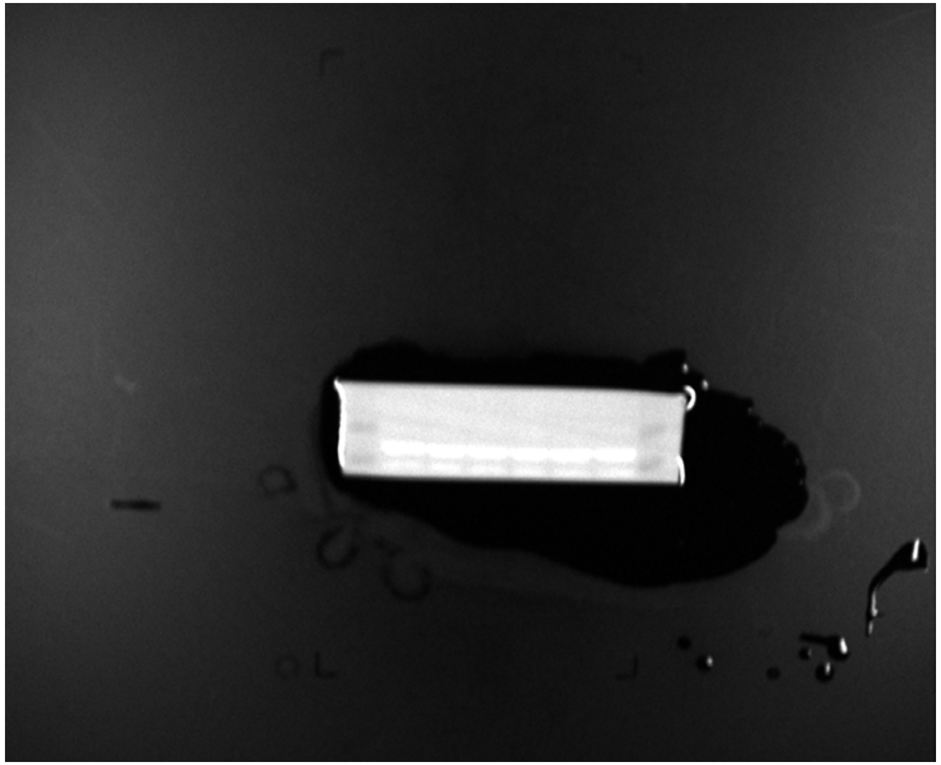

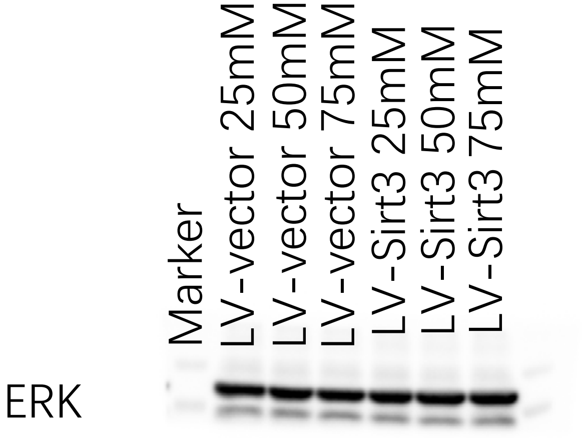

Marker
LV-vector 25mM
LV-vector 50mM
LV-vector 75mM
LV-Sirt3 25mM
LV-Sirt3 50mM
LV-Sirt3 75mM
ERK

Fig.5d

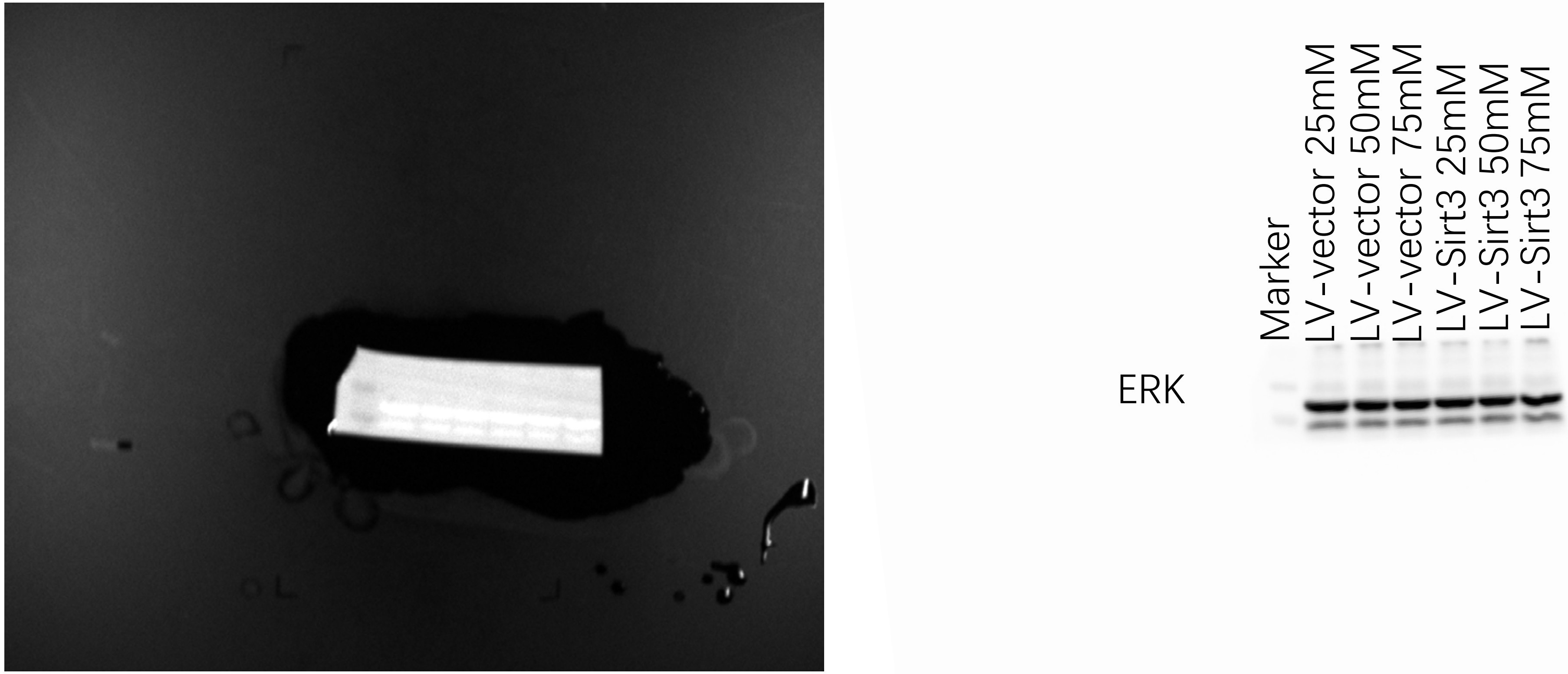
Marker
LV-vector 25mM
LV-vector 50mM
LV-vector 75mM
LV-Sirt3 25mM
LV-Sirt3 50mM
LV-Sirt3 75mM
ERK

Fig.5d

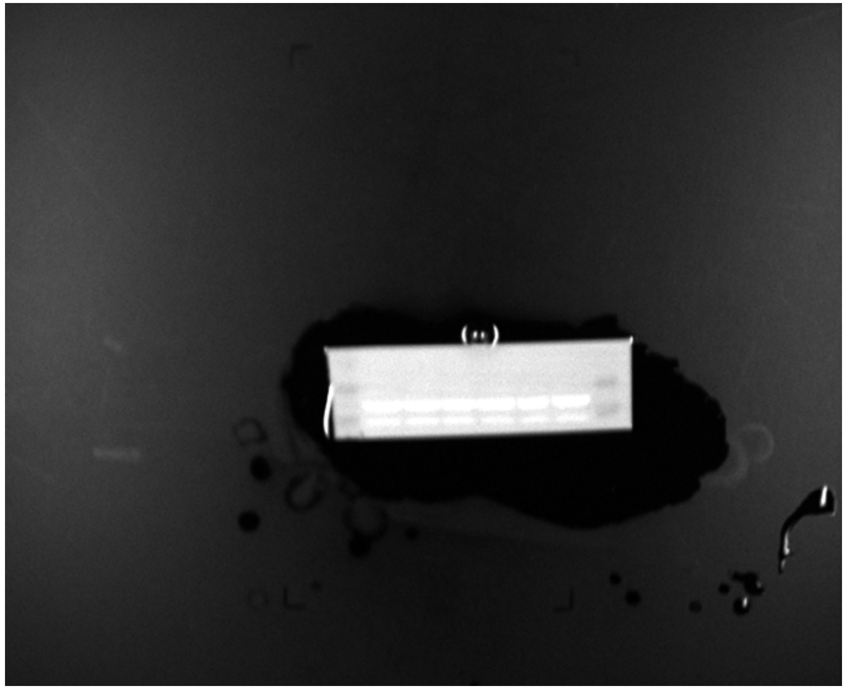

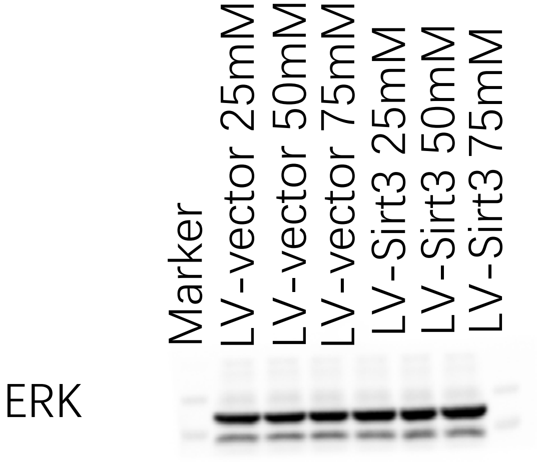
Marker
LV-vector 25mM
LV-vector 50mM
LV-vector 75mM
LV-Sirt3 25mM
LV-Sirt3 50mM
LV-Sirt3 75mM
ERK

Fig.5f

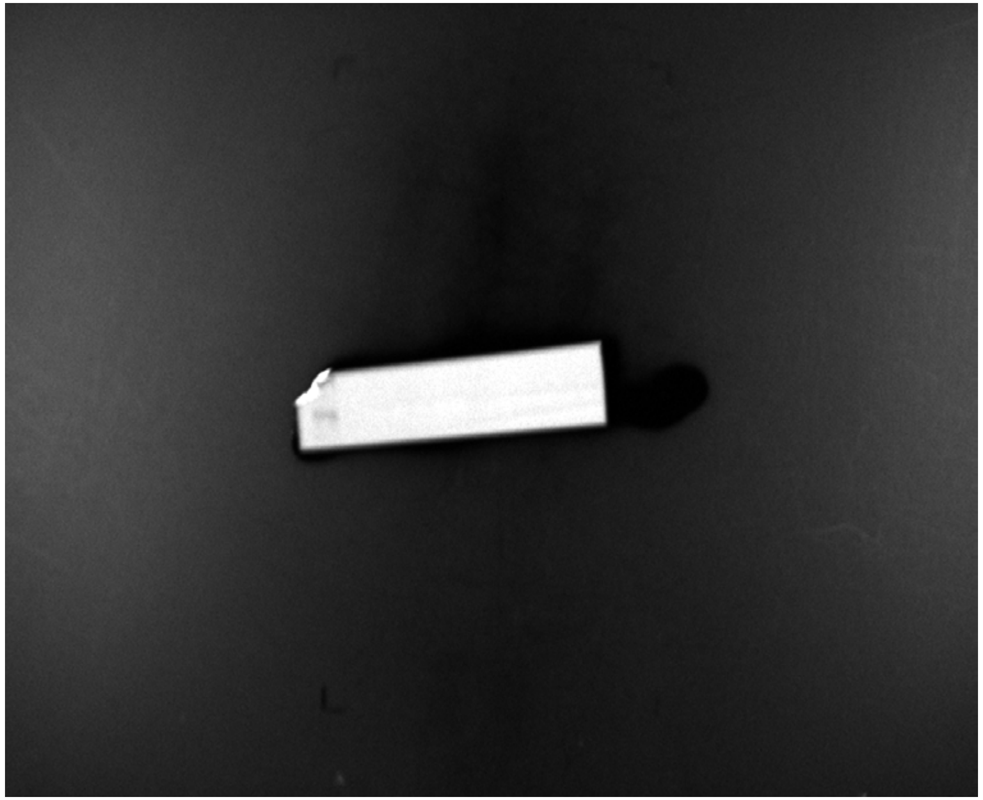

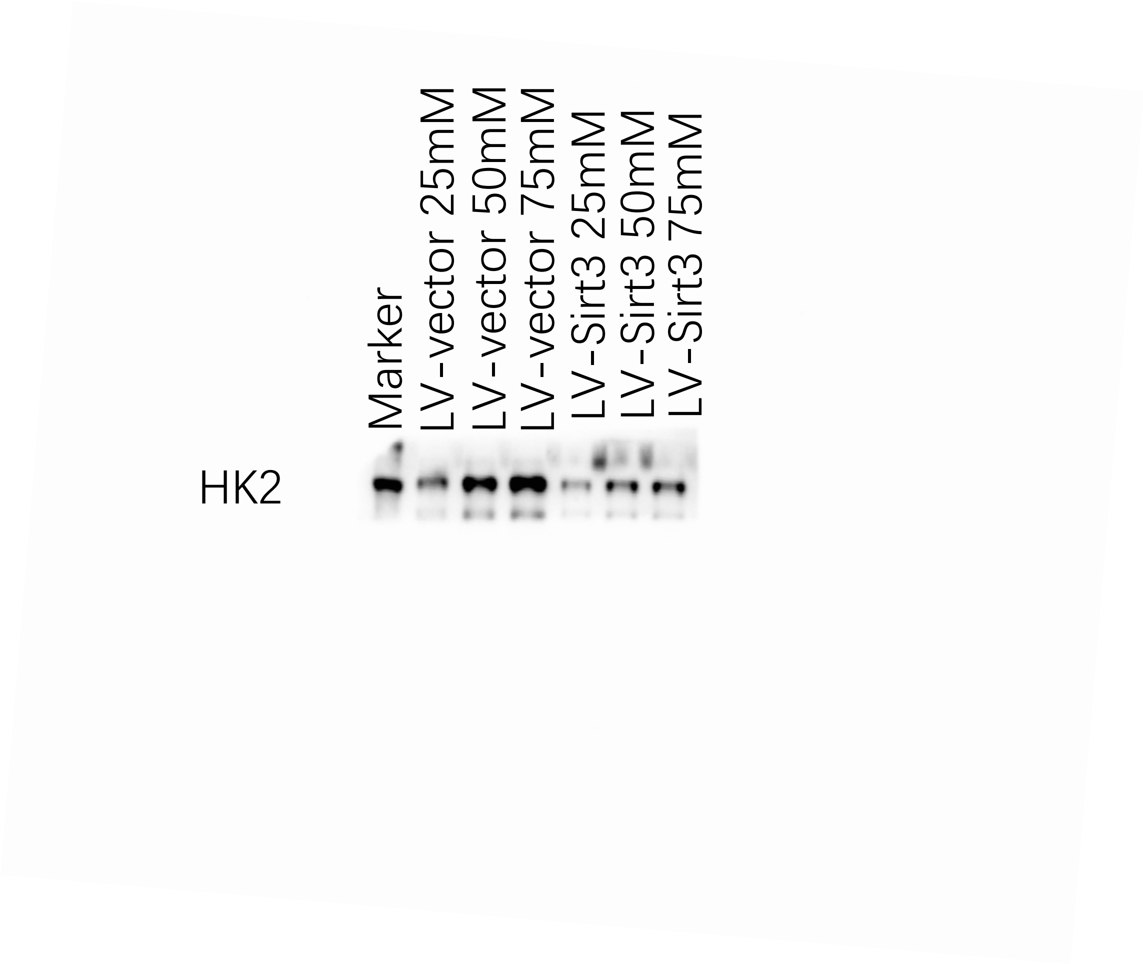
Marker
LV-vector 25mM
LV-vector 50mM
LV-vector 75mM
LV-Sirt3 25mM
LV-Sirt3 50mM
LV-Sirt3 75mM
HK2

Fig.5f

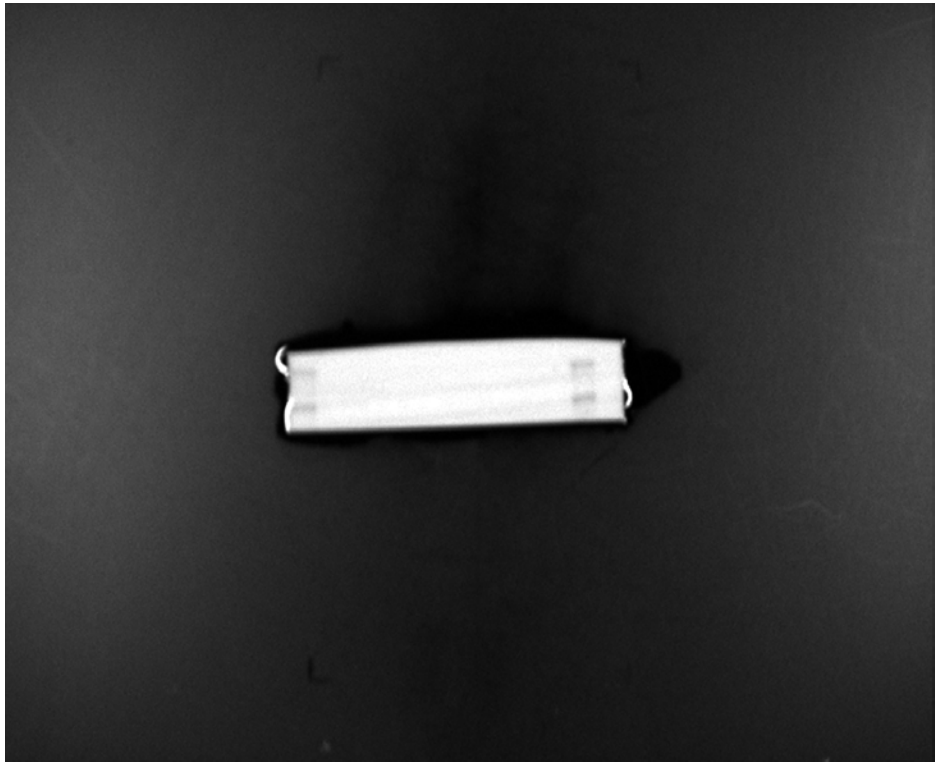

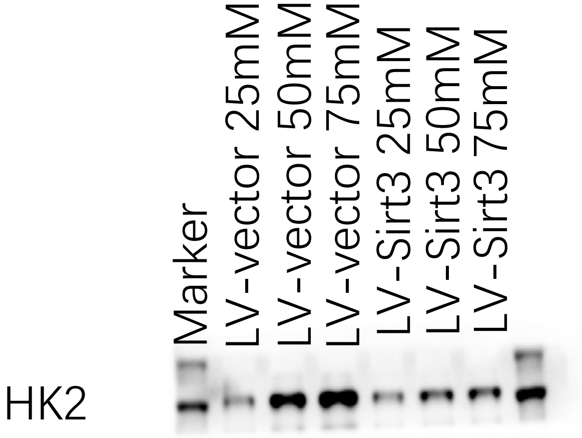
Marker
LV-vector 25mM
LV-vector 50mM
LV-vector 75mM
LV-Sirt3 25mM
LV-Sirt3 50mM
LV-Sirt3 75mM
HK2

Fig.5f

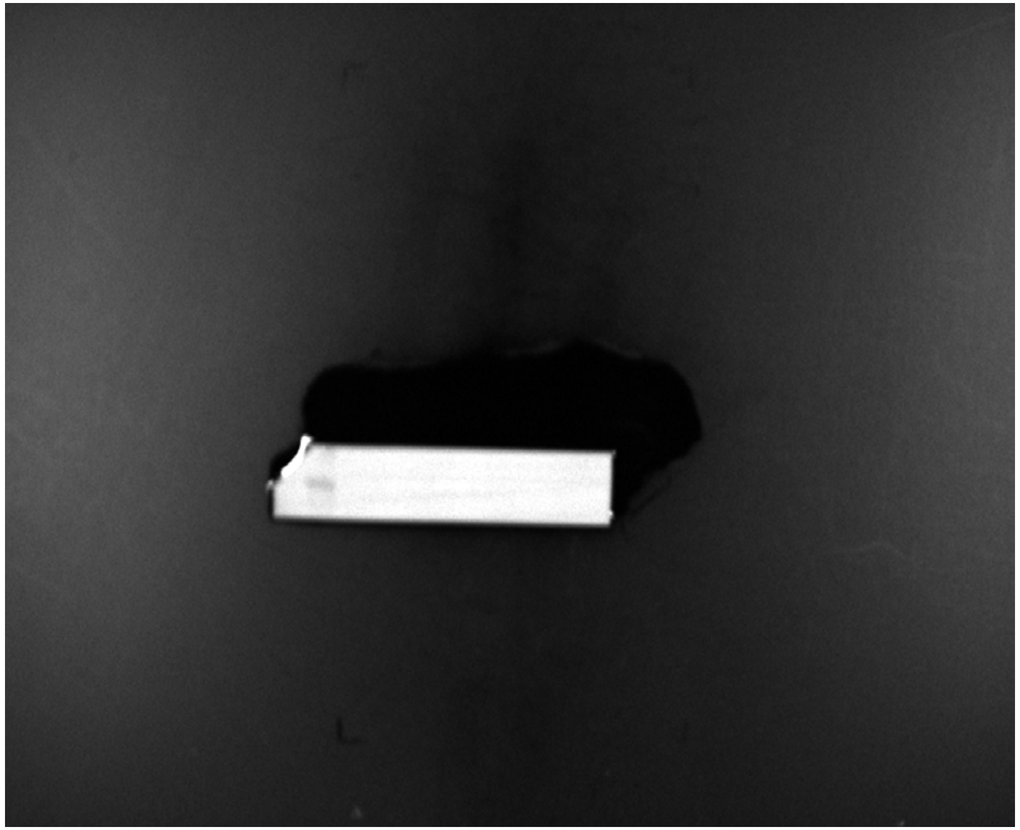

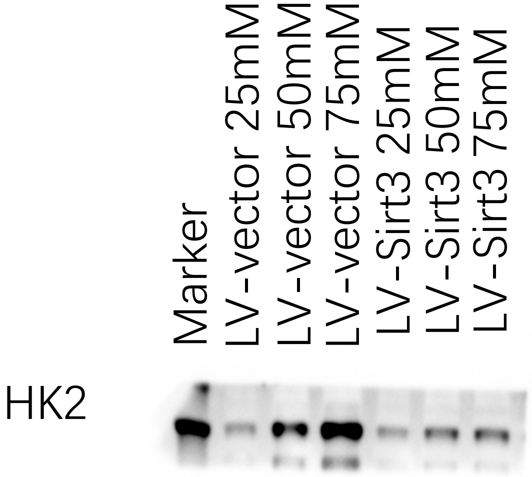
Marker
LV-vector 25mM
LV-vector 50mM
LV-vector 75mM
LV-Sirt3 25mM
LV-Sirt3 50mM
LV-Sirt3 75mM
HK2

Fig.5f

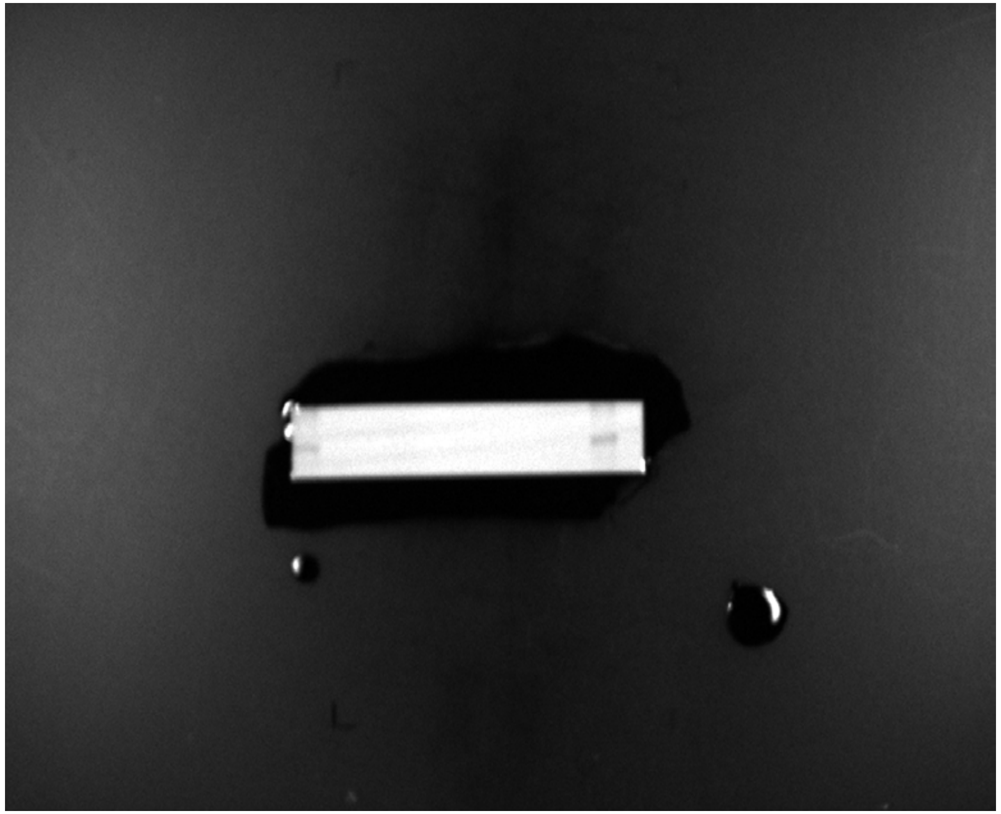

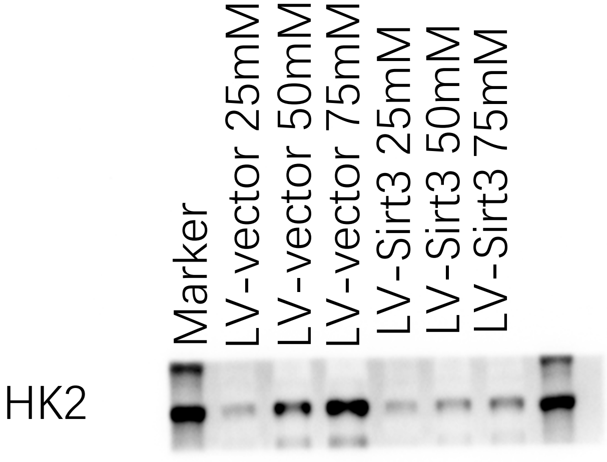
Marker
LV-vector 25mM
LV-vector 50mM
LV-vector 75mM
LV-Sirt3 25mM
LV-Sirt3 50mM
LV-Sirt3 75mM
HK2

Fig.5f

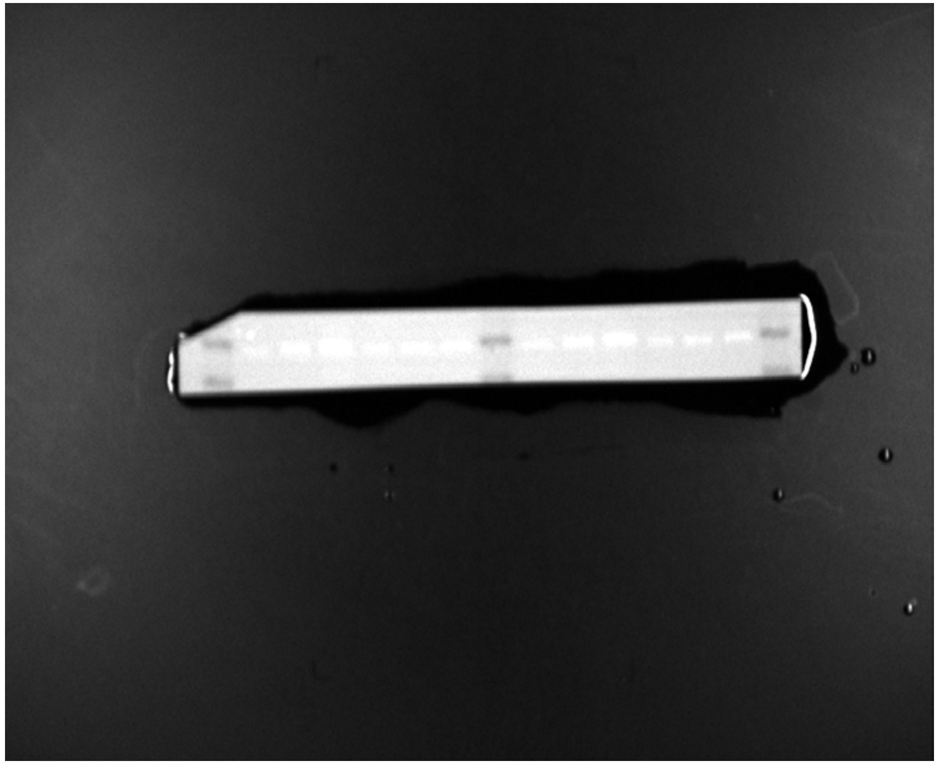

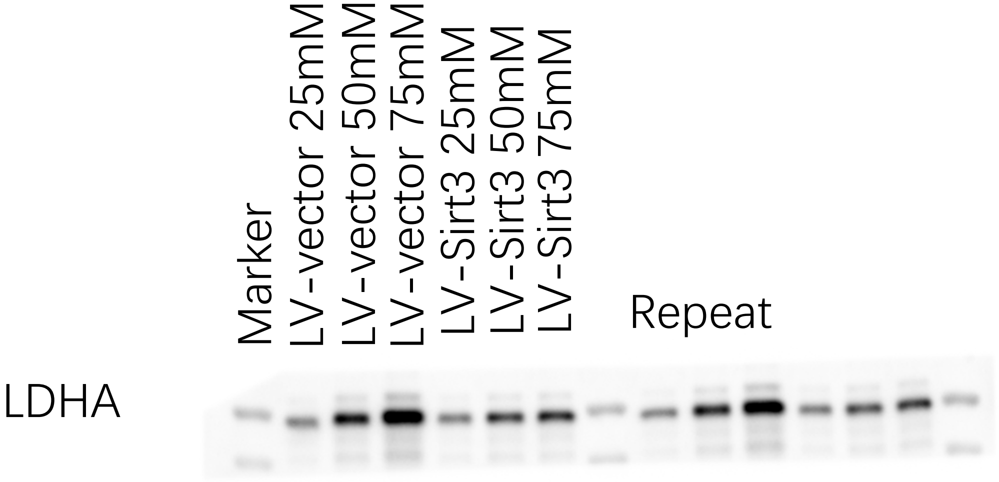
Marker
LV-vector 25mM
LV-vector 50mM
LV-vector 75mM
LV-Sirt3 25mM
LV-Sirt3 50mM
LV-Sirt3 75mM
Repeat
LDHA

Fig.5f

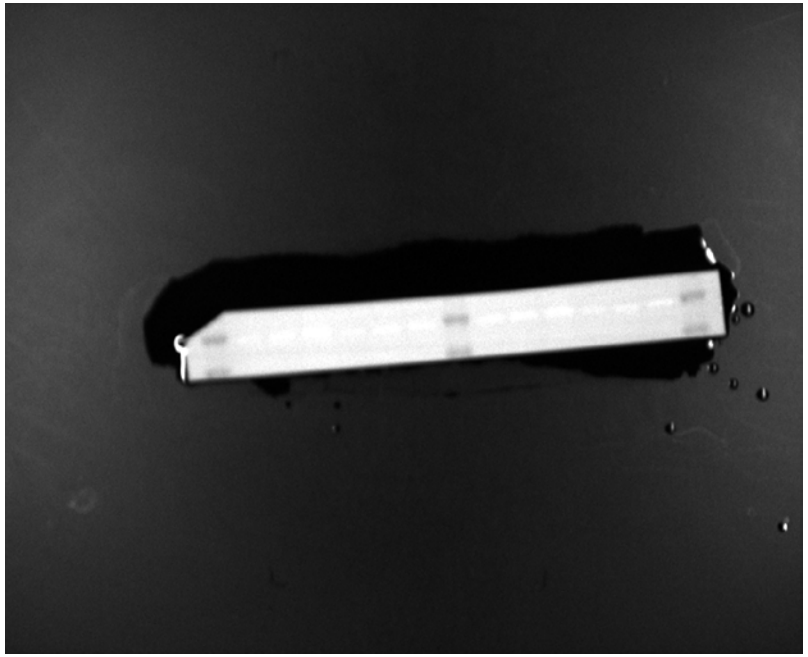

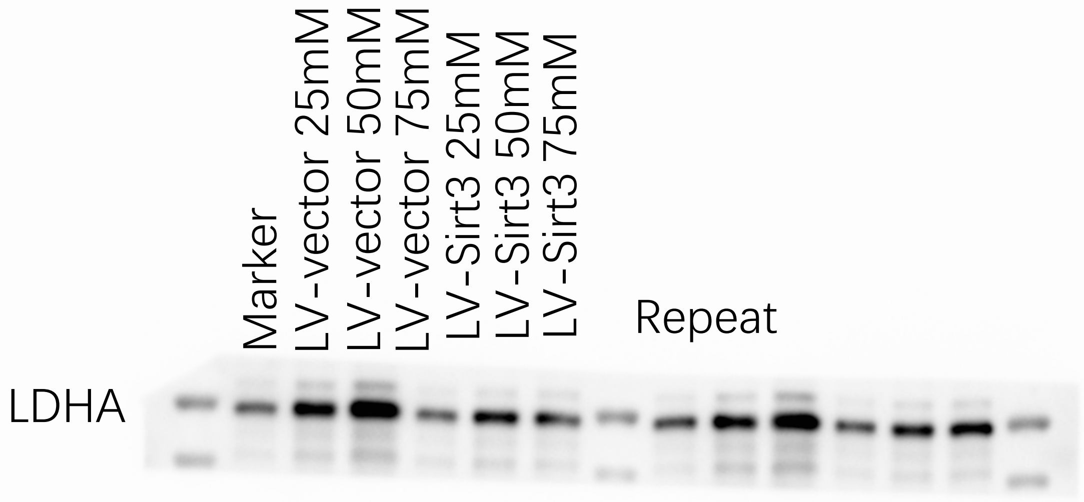
Marker
LV-vector 25mM
LV-vector 50mM
LV-vector 75mM
LV-Sirt3 25mM
LV-Sirt3 50mM
LV-Sirt3 75mM
Repeat
LDHA

Fig.5f

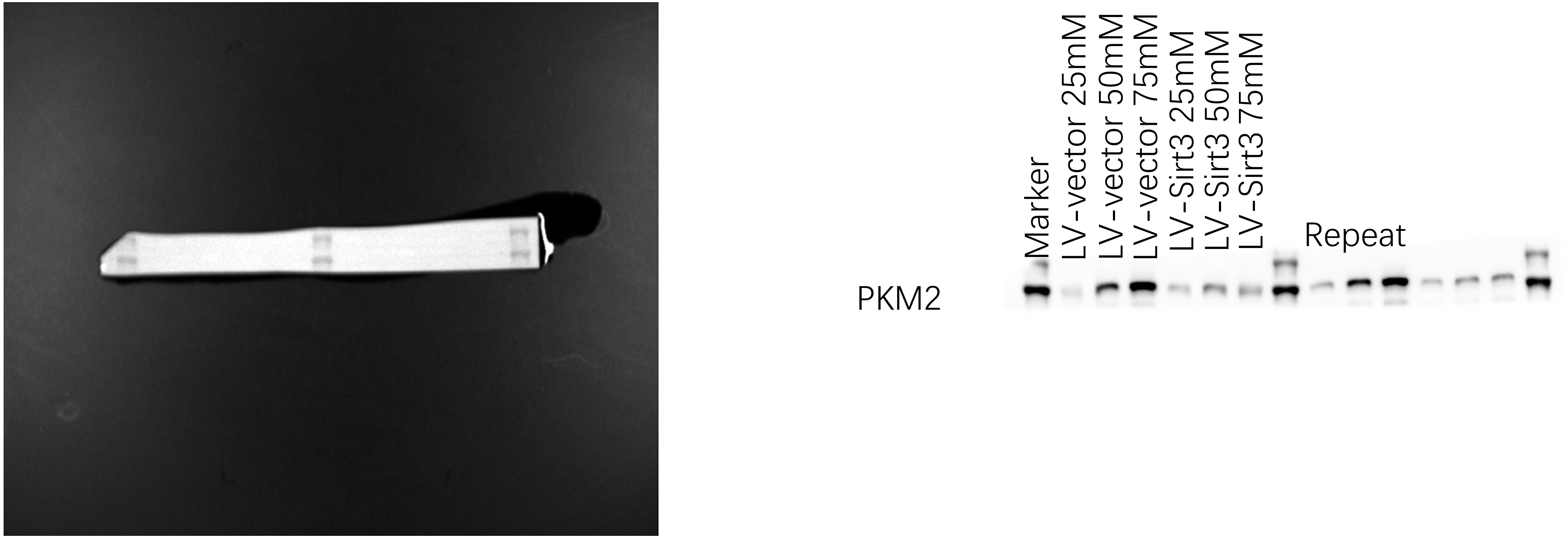
Marker
LV-vector 25mM
LV-vector 50mM
LV-vector 75mM
LV-Sirt3 25mM
LV-Sirt3 50mM
LV-Sirt3 75mM
Repeat
PKM2

Fig.5f

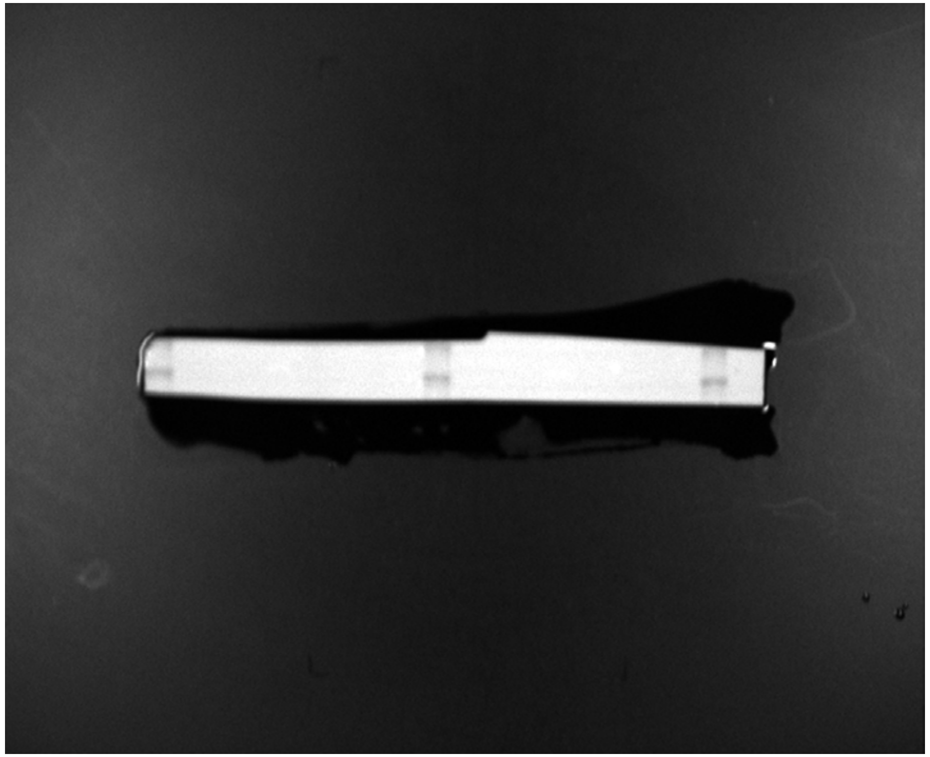

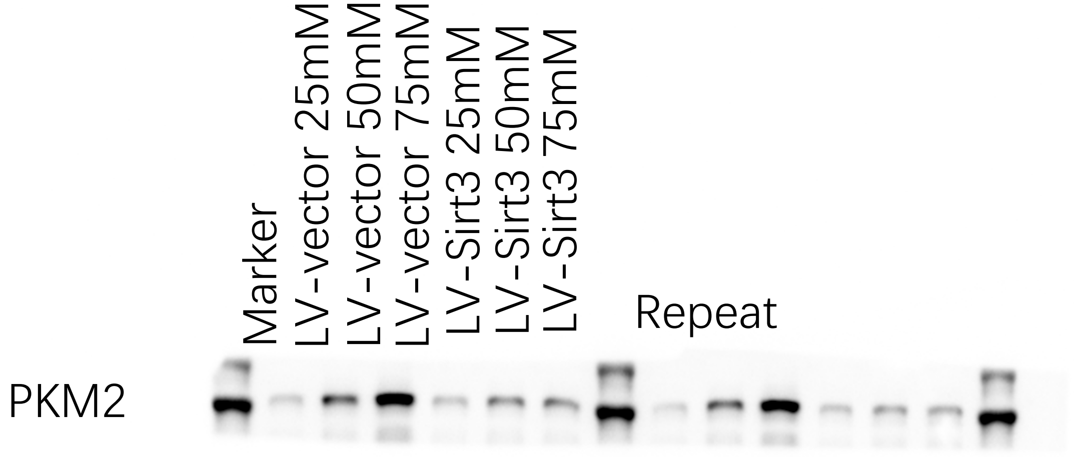
Marker
LV-vector 25mM
LV-vector 50mM
LV-vector 75mM
LV-Sirt3 25mM
LV-Sirt3 50mM
LV-Sirt3 75mM
Repeat
PKM2

Fig.6e

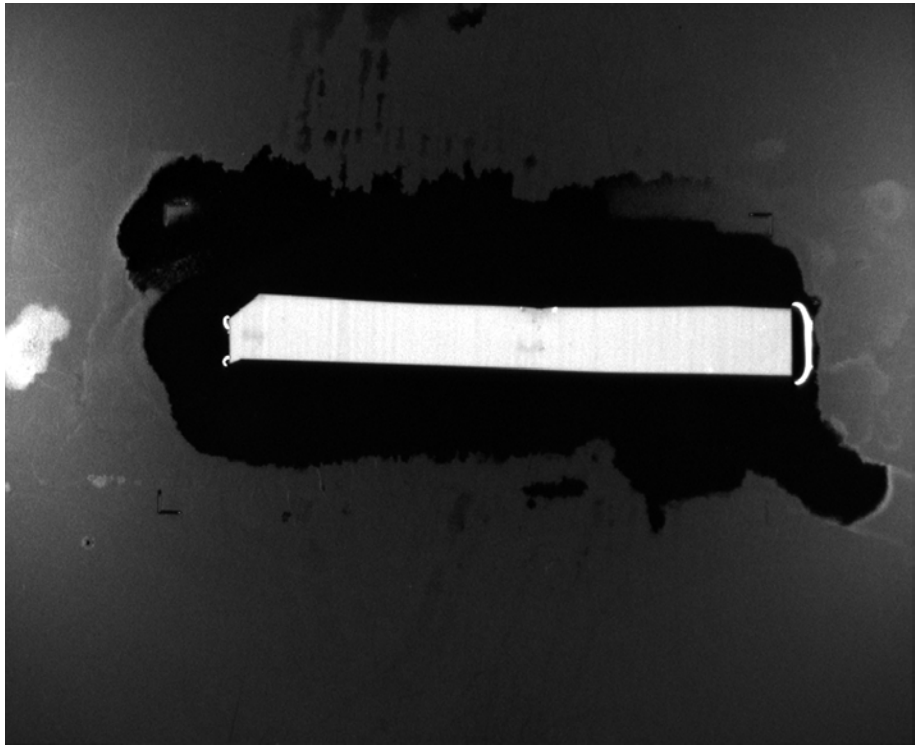

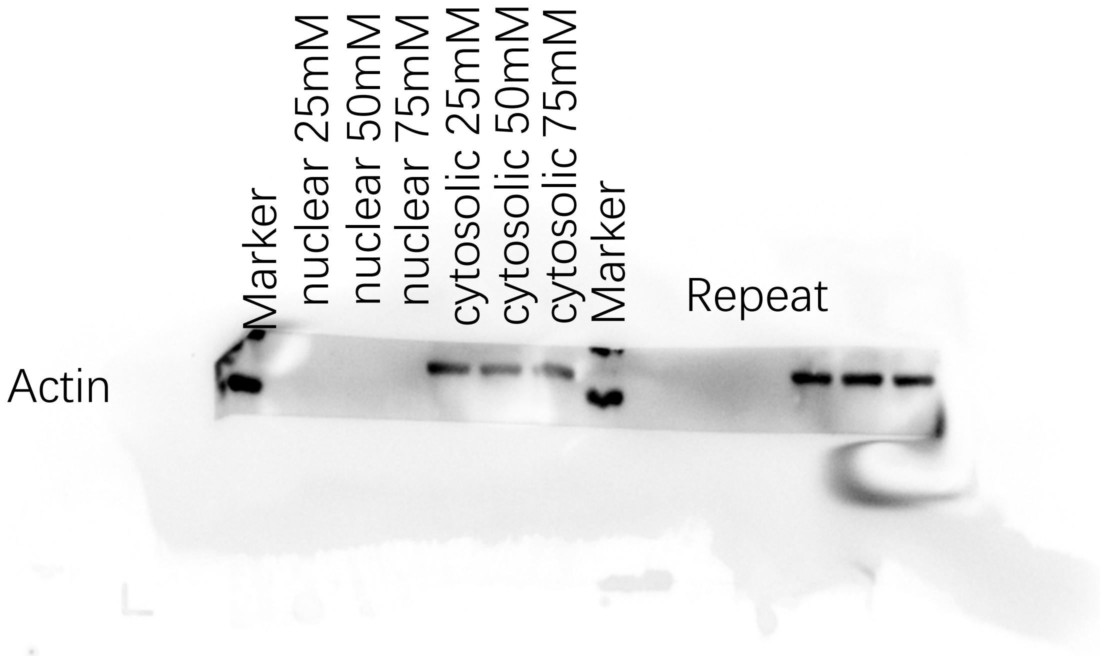

Marker
nuclear 25mM
nuclear 50mM
nuclear 75mM
cytosolic 25mM
cytosolic 50mM
cytosolic 75mM
Marker
Repeat
Actin

Fig.6e

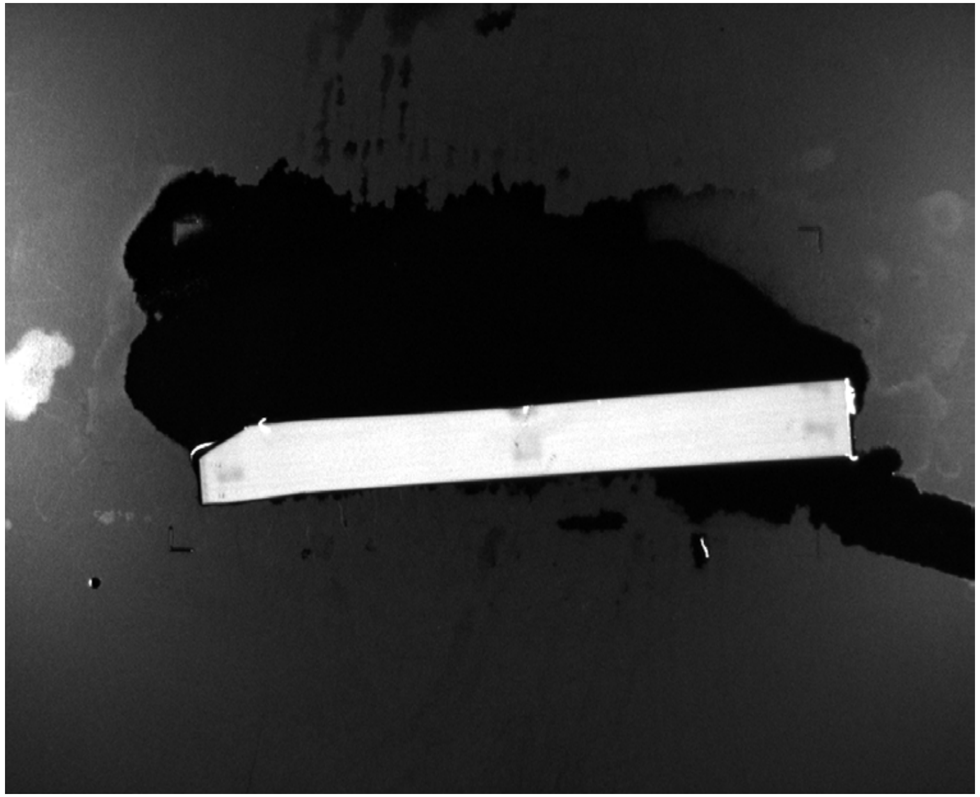

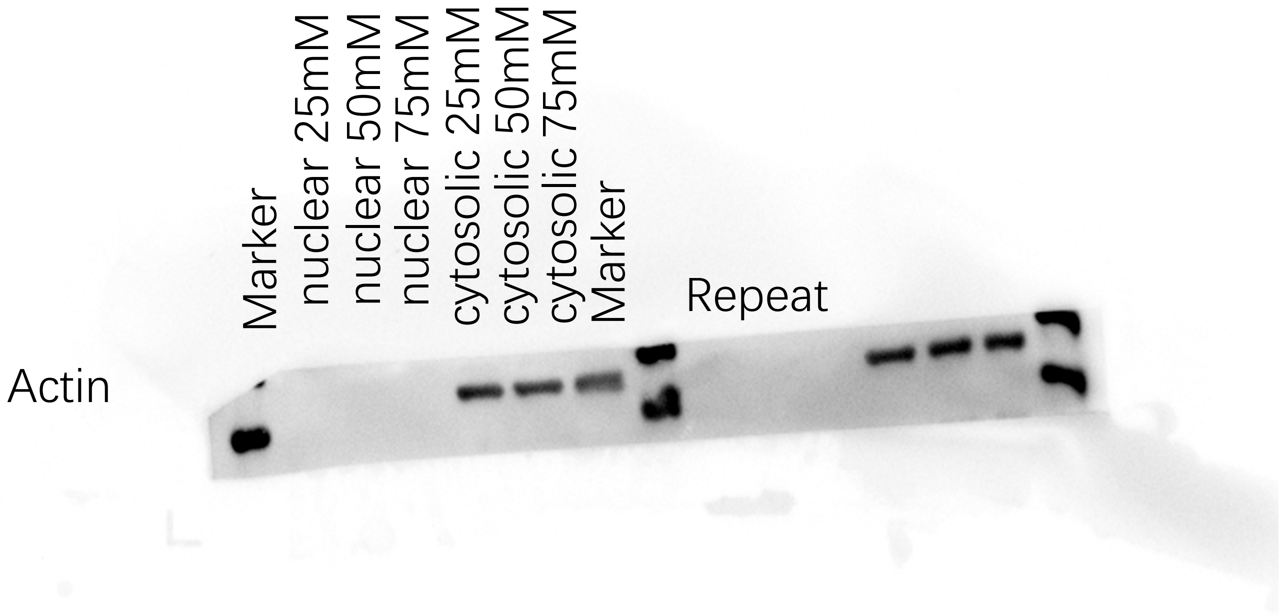
Marker
nuclear 25mM
nuclear 50mM
nuclear 75mM
cytosolic 25mM
cytosolic 50mM
cytosolic 75mM
Marker
Repeat
Actin

Fig.6e

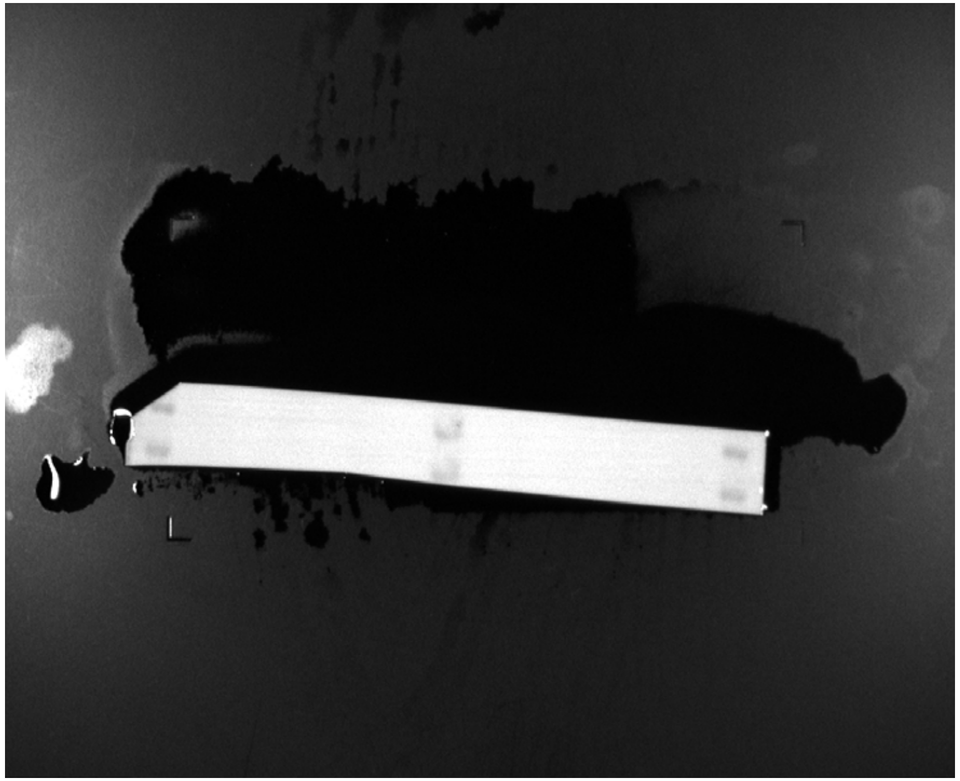

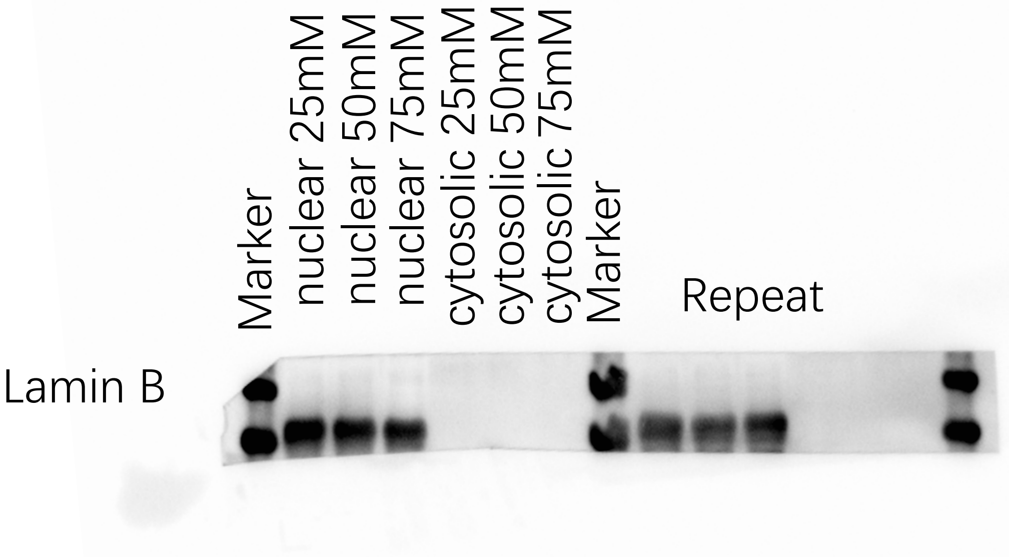
Marker
nuclear 25mM
nuclear 50mM
nuclear 75mM
cytosolic 25mM
cytosolic 50mM
cytosolic 75mM
Marker
Repeat
Lamin B

Fig.6e

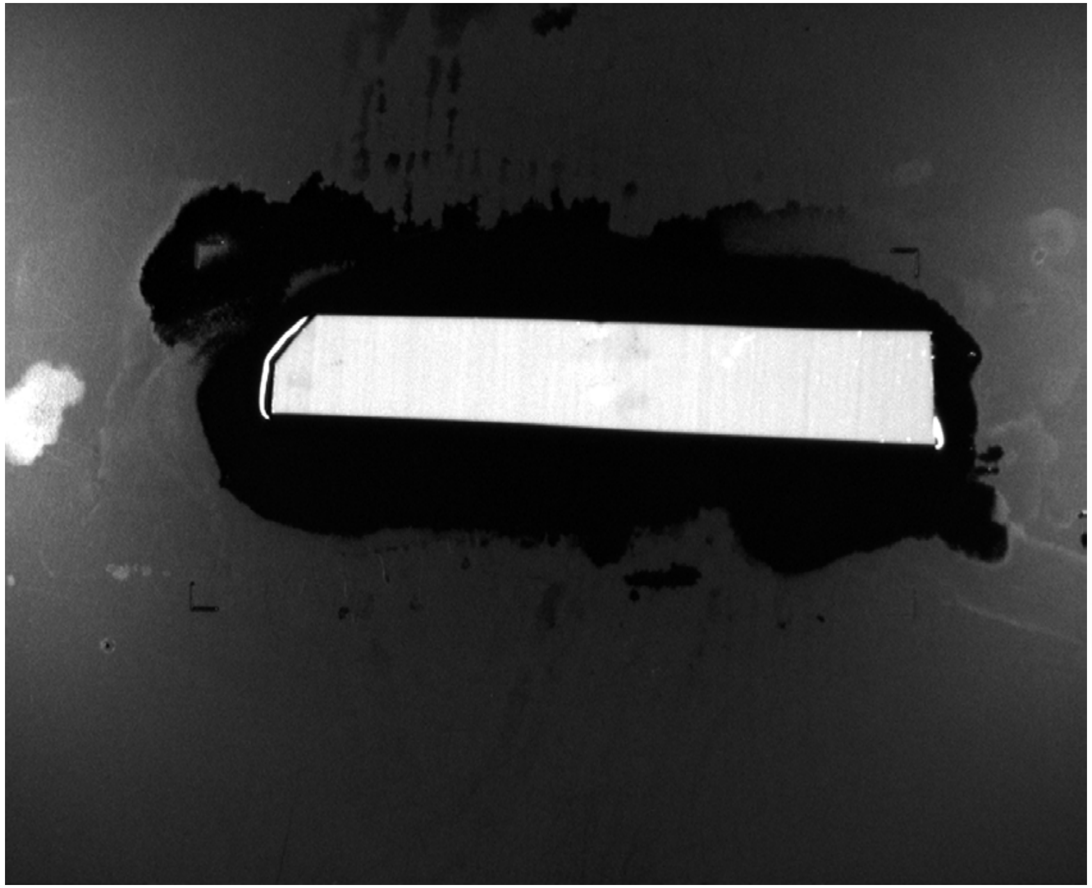

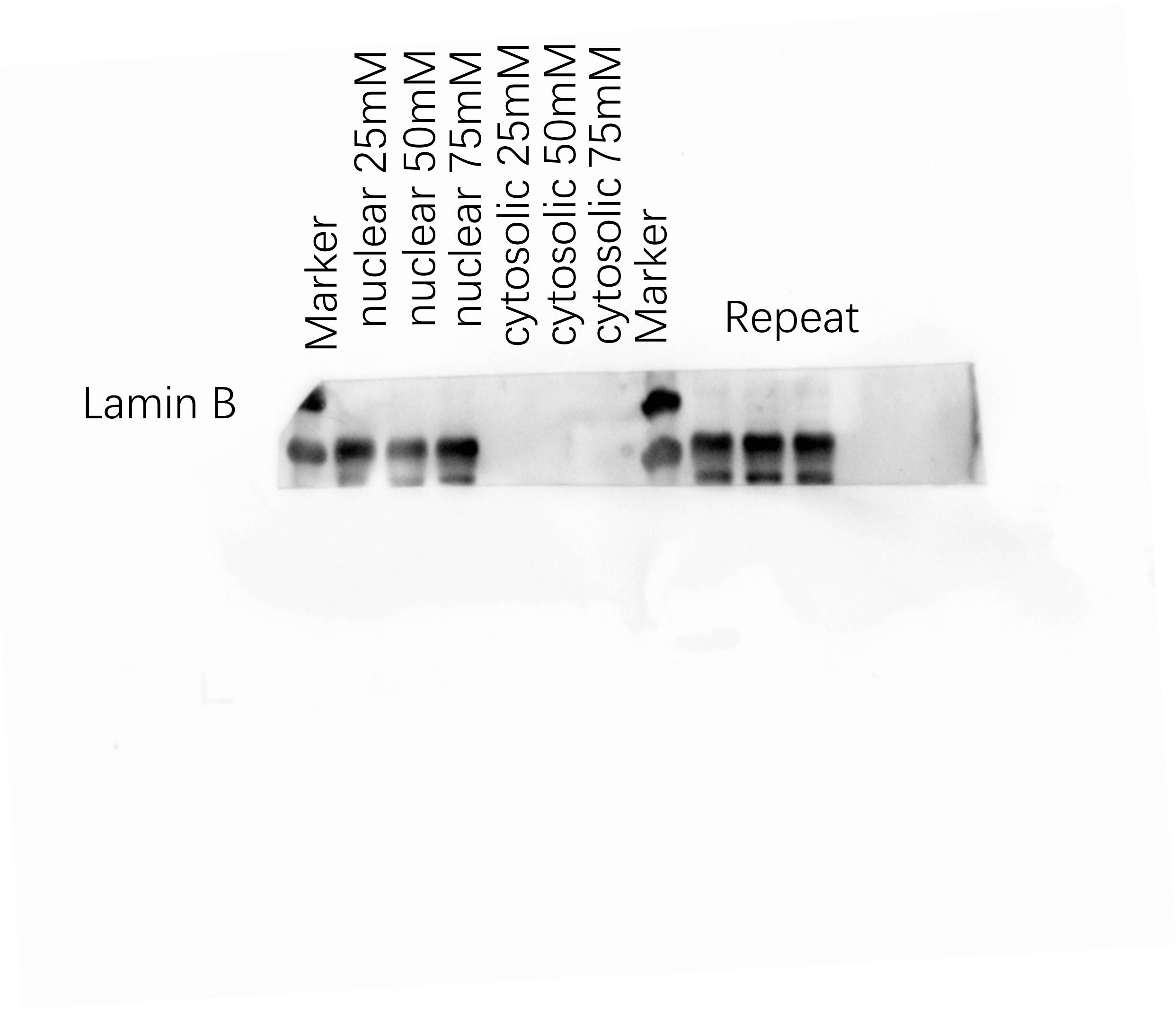
Marker
nuclear 25mM
nuclear 50mM
nuclear 75mM
cytosolic 25mM
cytosolic 50mM
cytosolic 75mM
Marker
Repeat
Lamin B

Fig.6e

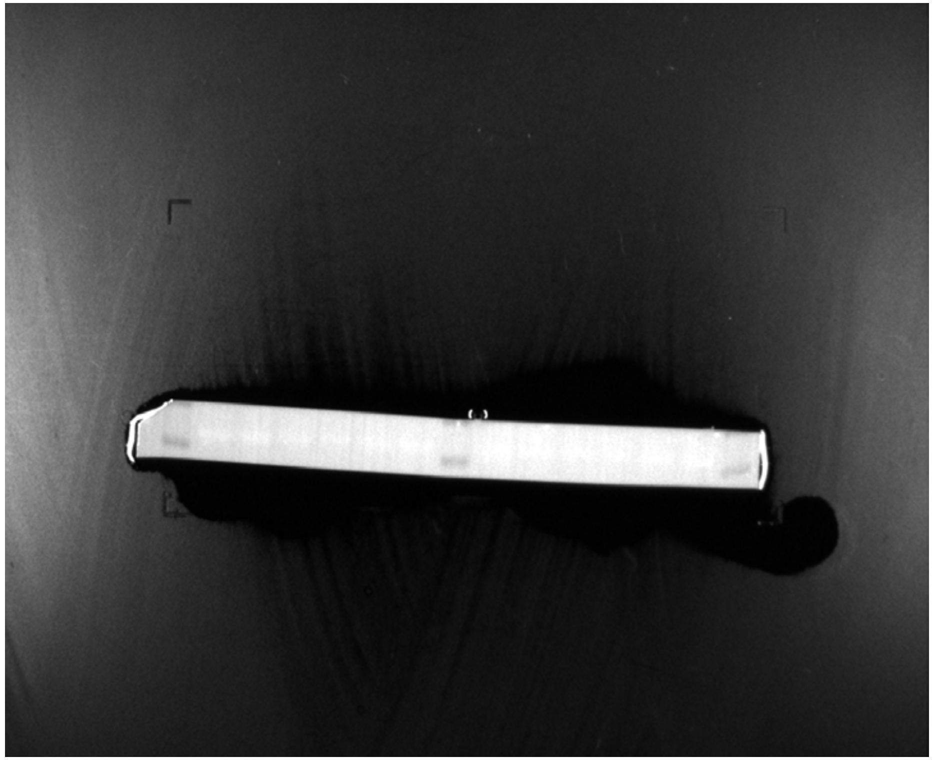

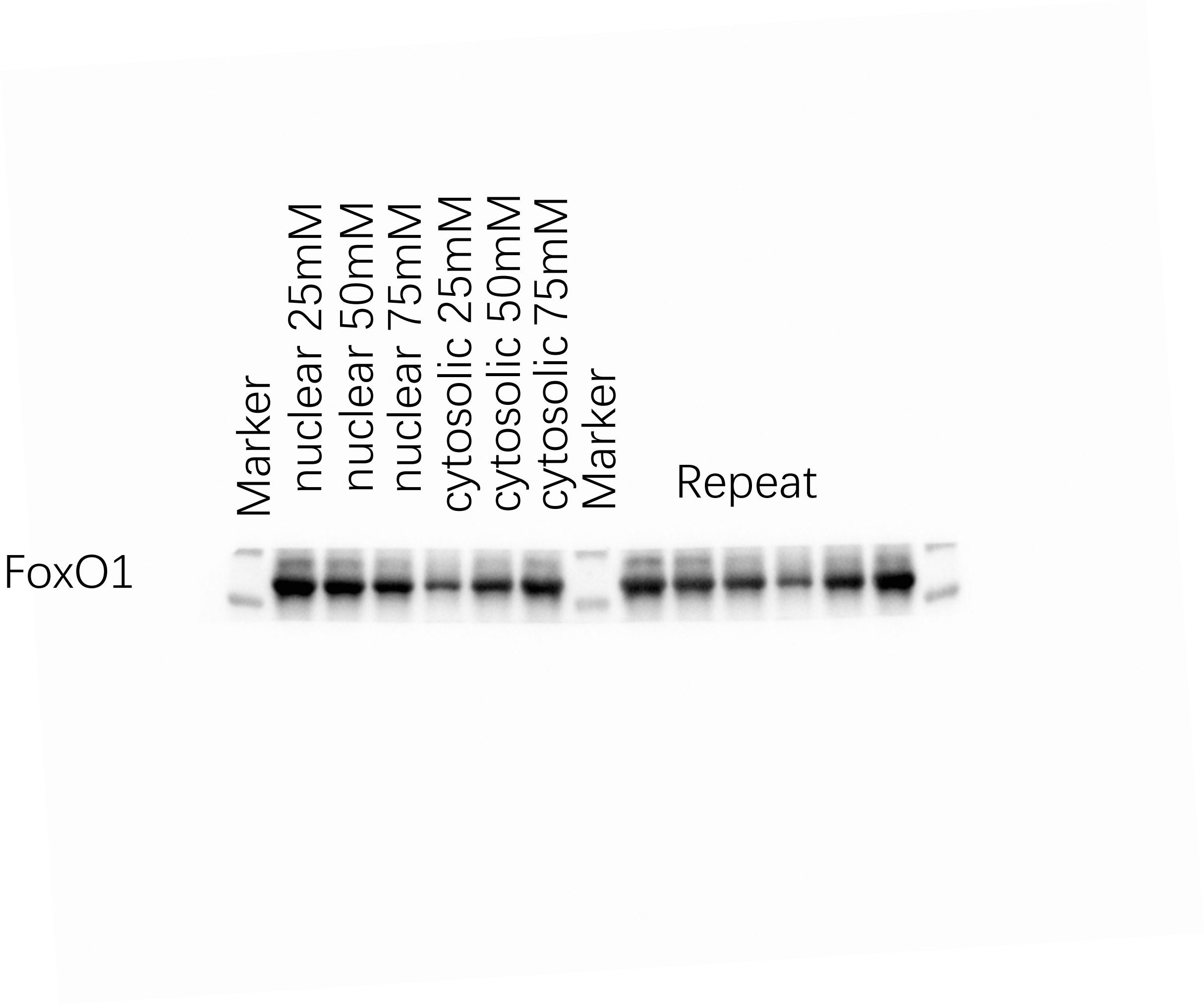
Marker
nuclear 25mM
nuclear 50mM
nuclear 75mM
cytosolic 25mM
cytosolic 50mM
cytosolic 75mM
Marker
Repeat
FoxO1

Fig.6e

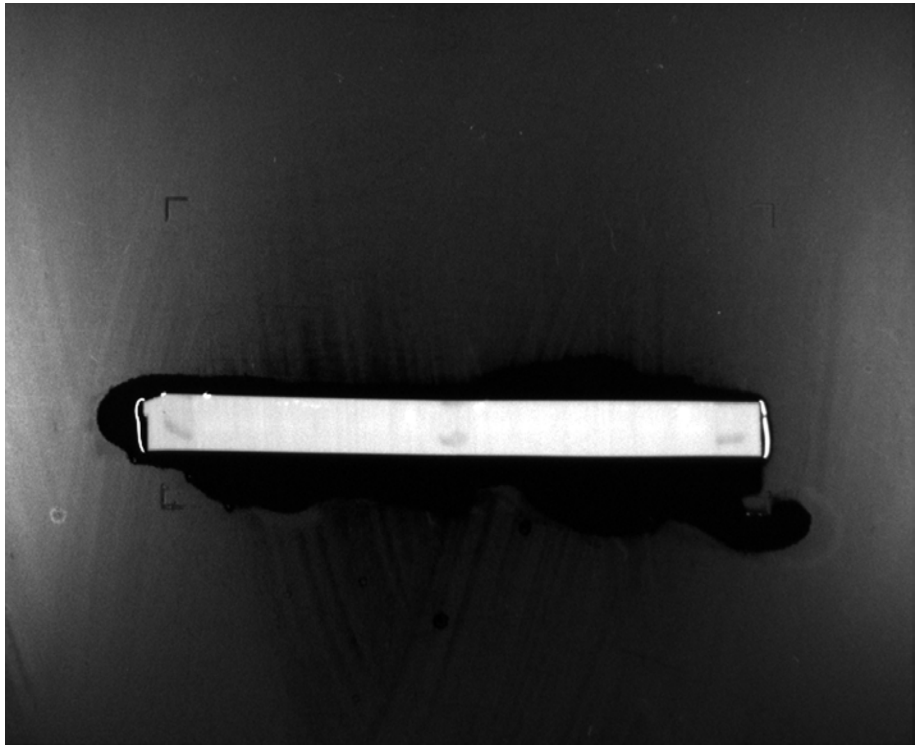

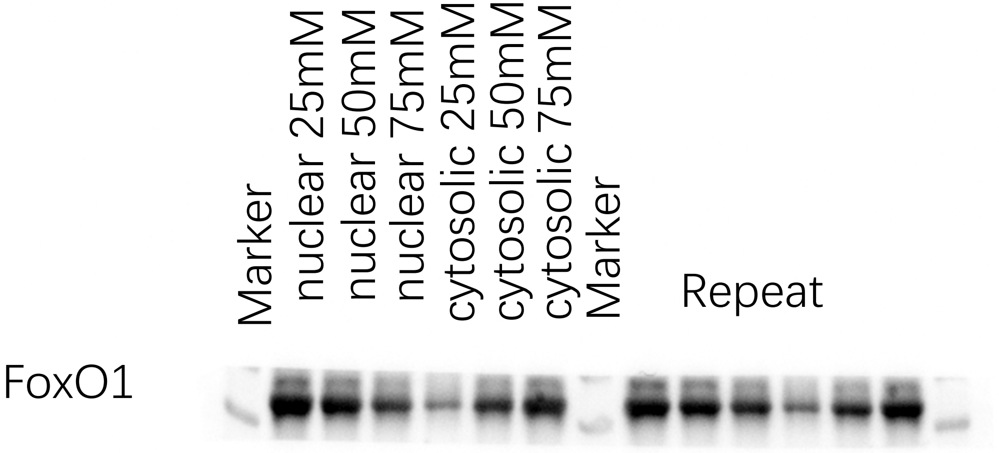

Marker
nuclear 25mM
nuclear 50mM
nuclear 75mM
cytosolic 25mM
cytosolic 50mM
cytosolic 75mM
Marker
Repeat
FoxO1

Fig.6f

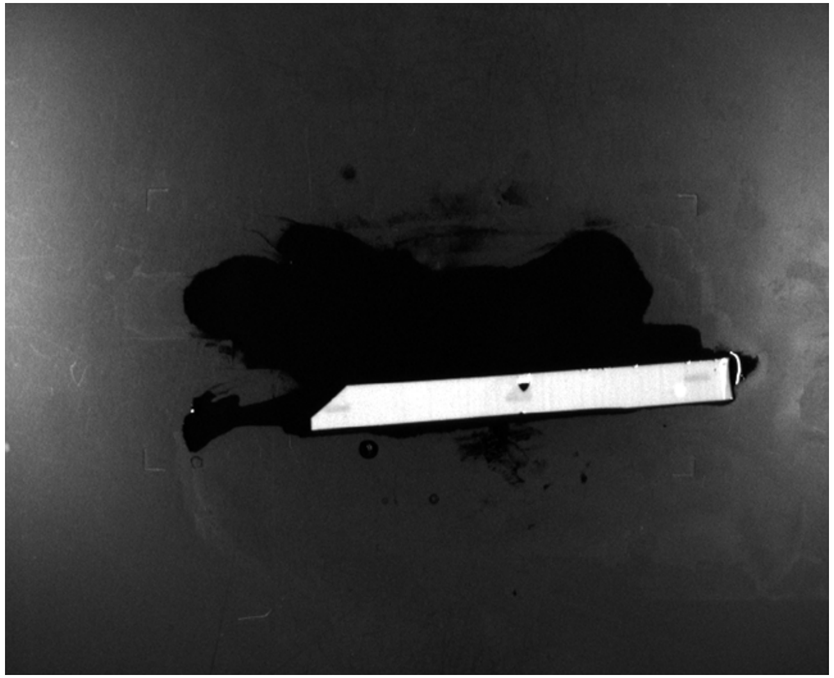

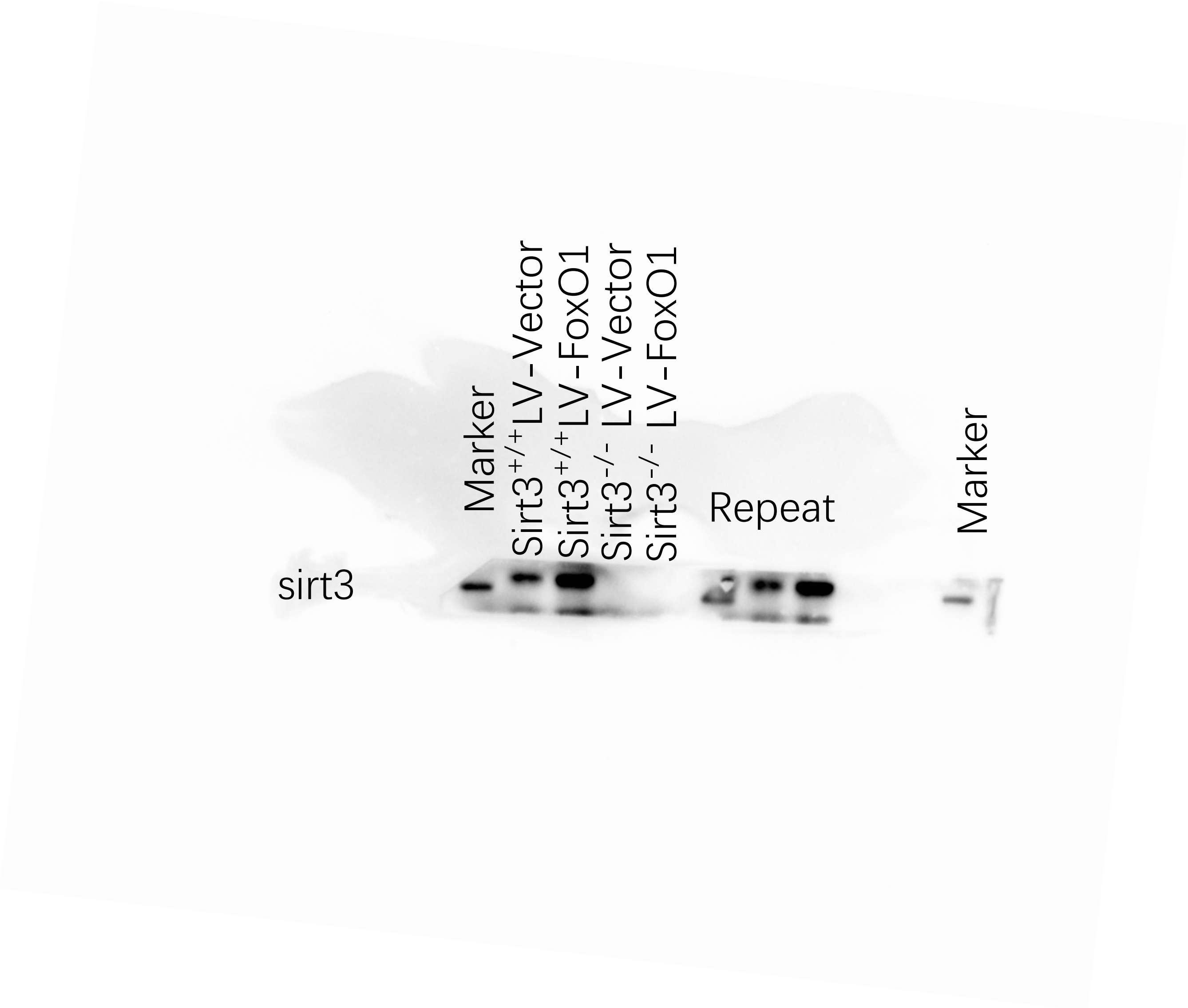
Marker
Sirt3+/+ LV-Vector
Sirt3+/+ LV-FoxO1
Sirt3-/- LV-Vector
Sirt3-/- LV-FoxO1
Repeat
Marker
sirt3

Fig.6f

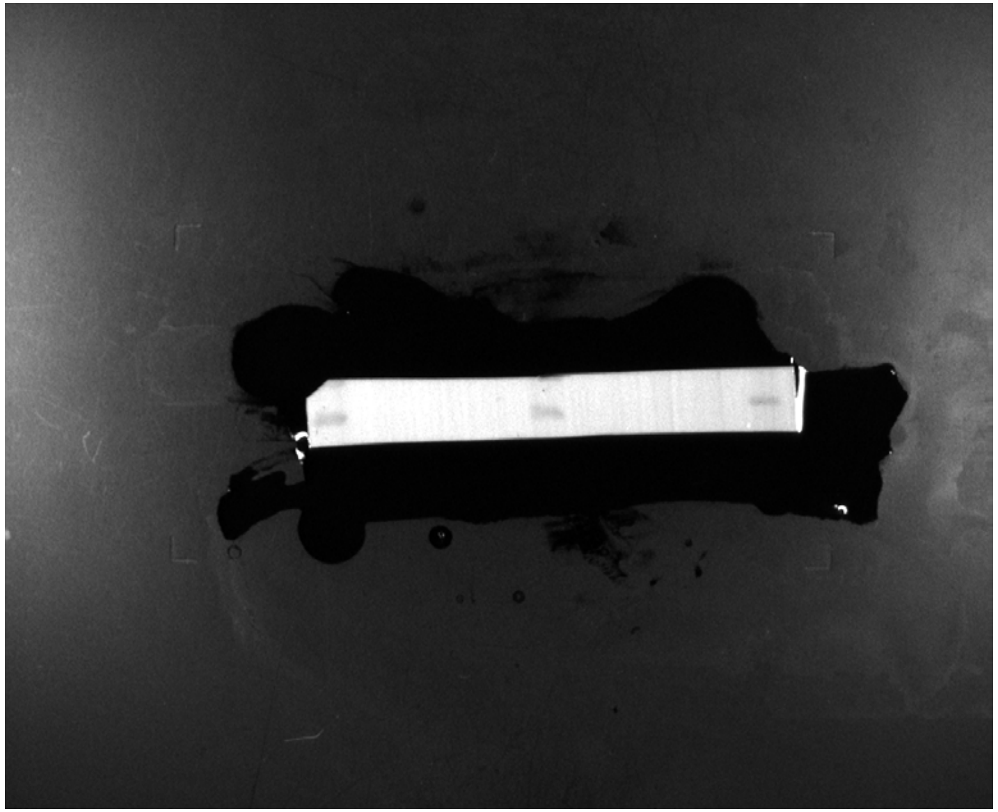

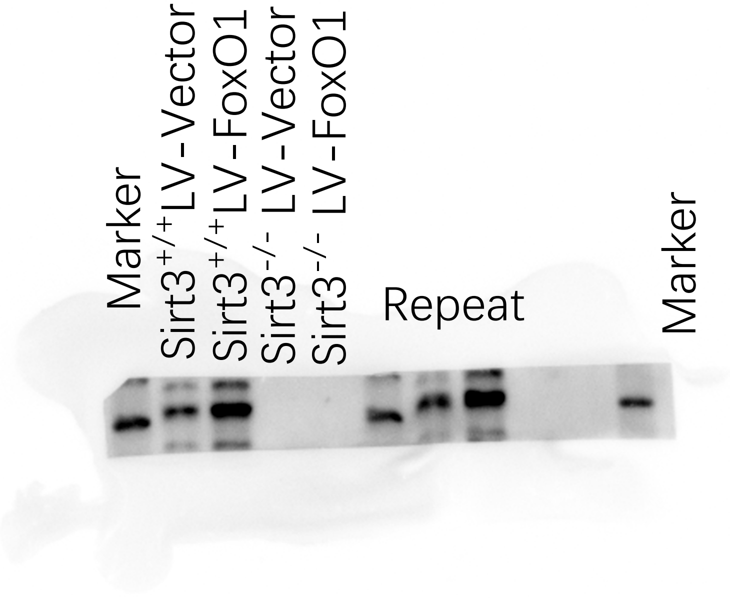
Marker
Sirt3+/+LV-Vector
Sirt3+/+LV-FoxO1
Sirt3-/- LV-Vector
Sirt3-/- LV-FoxO1
Repeat
Marker
sirt3

Fig.6f

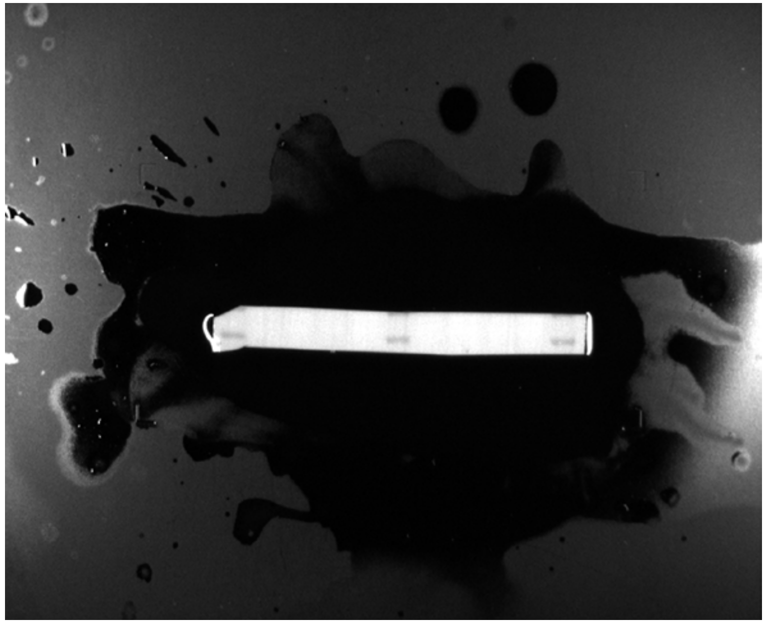

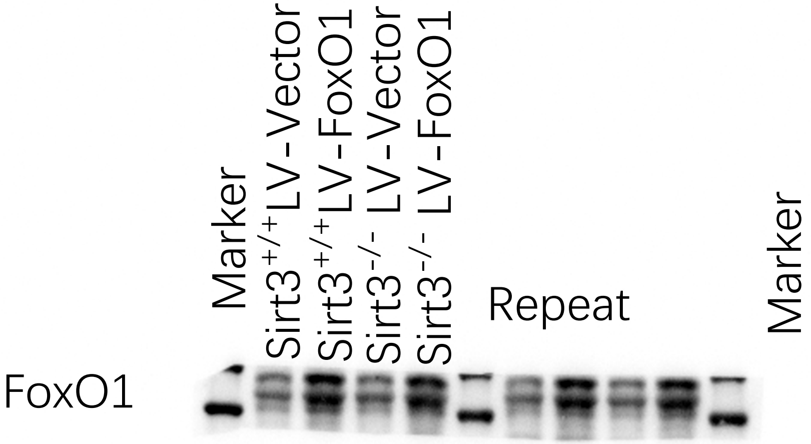
FoxO1
Marker
Sirt3+/+LV-Vector
Sirt3+/+LV-FoxO1
Sirt3-/- LV-Vector
Sirt3-/- LV-FoxO1
Repeat
Marker

Fig.6f

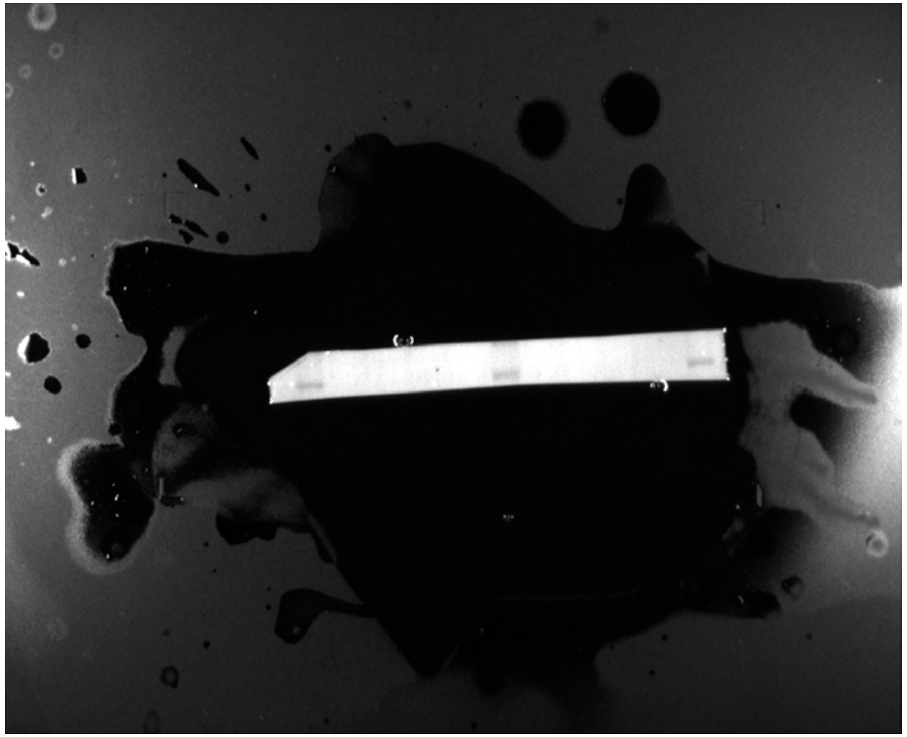

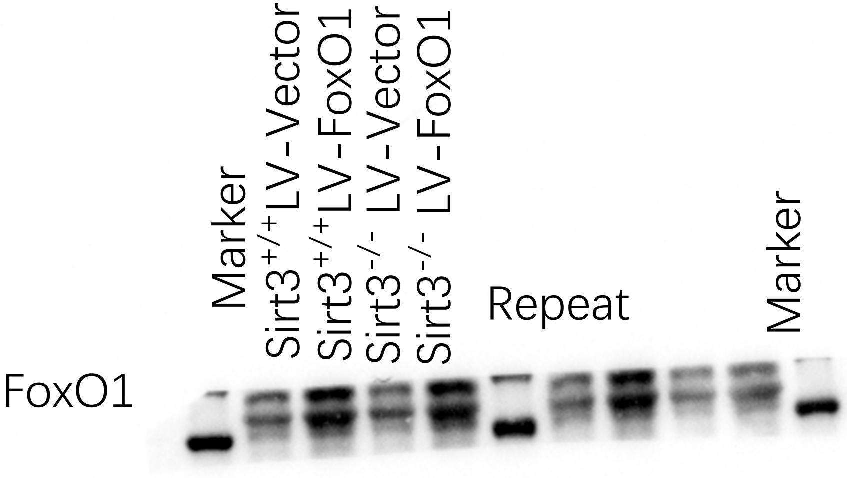
Marker
Sirt3+/+ LV-Vector
Sirt3+/+ LV-FoxO1
Sirt3-/- LV-Vector
Sirt3-/- LV-FoxO1
Repeat
Marker
FoxO1

Fig.6f

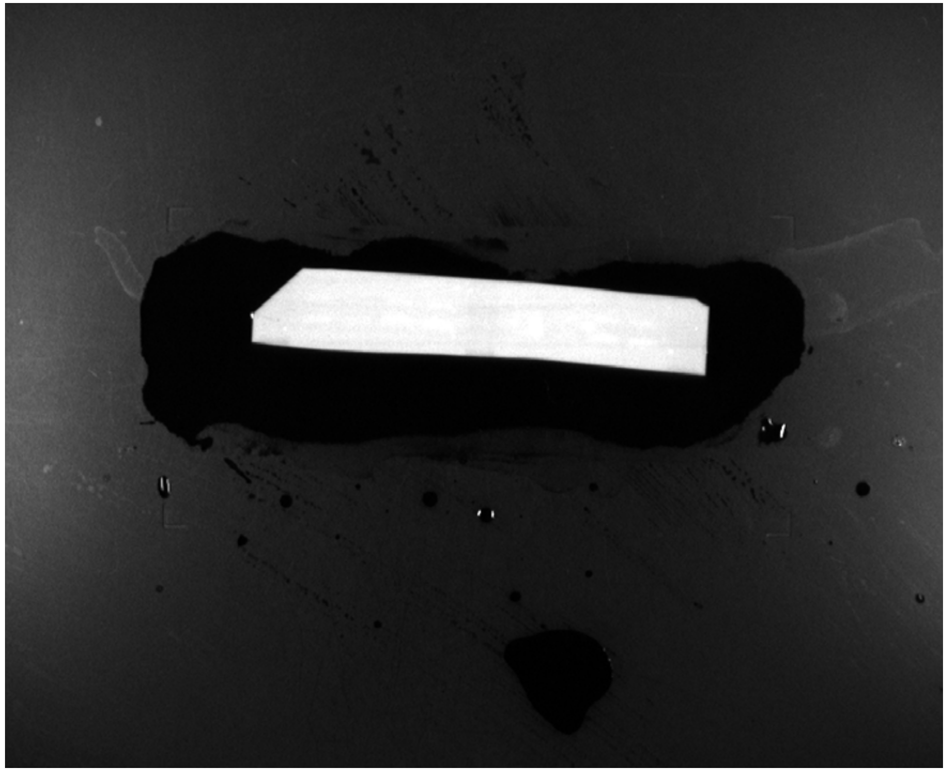

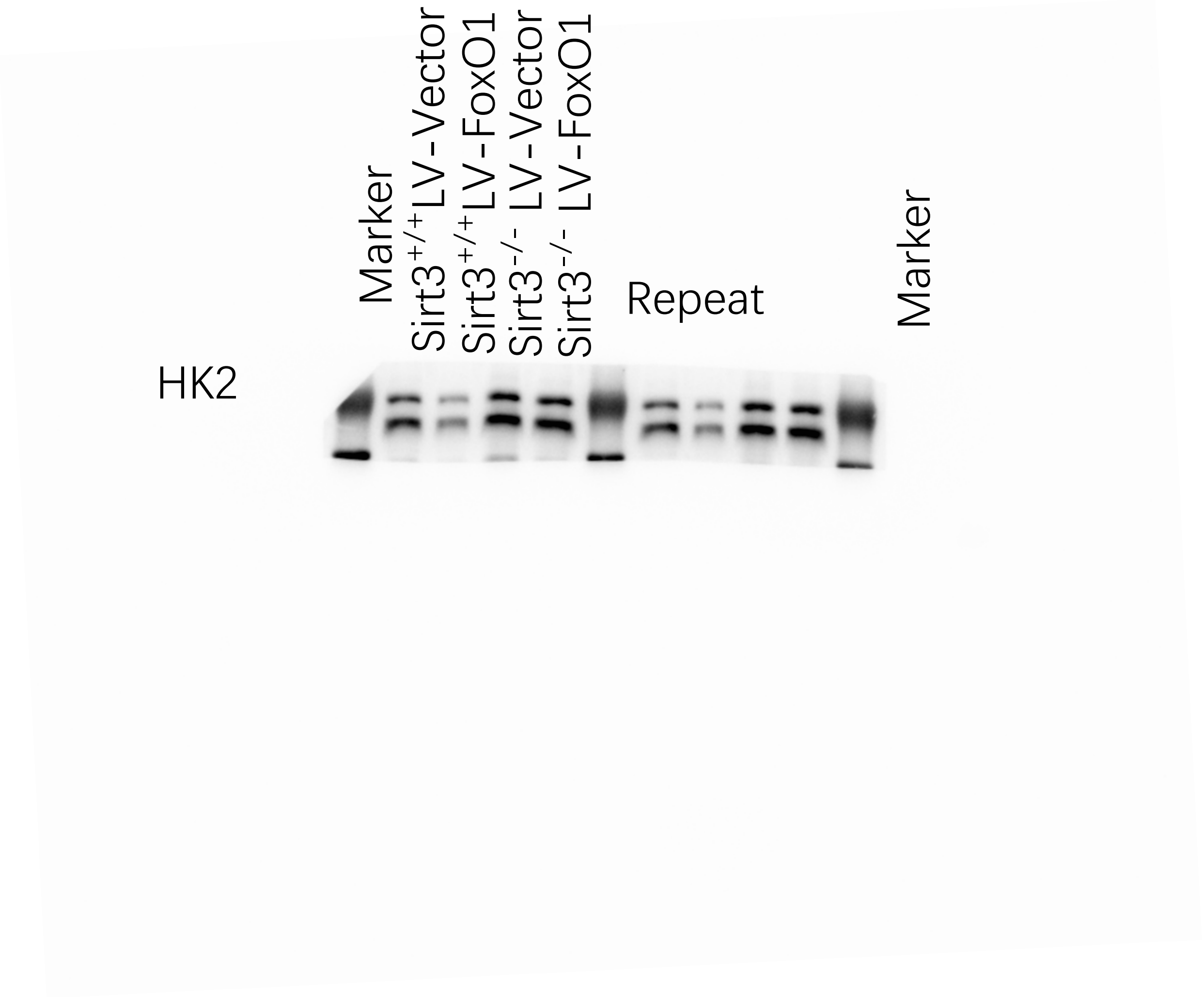
Marker
Sirt3+/+ LV-Vector
Sirt3+/+ LV-FoxO1
Sirt3-/- LV-Vector
Sirt3-/- LV-FoxO1
Repeat
Marker
HK2

Fig.6f

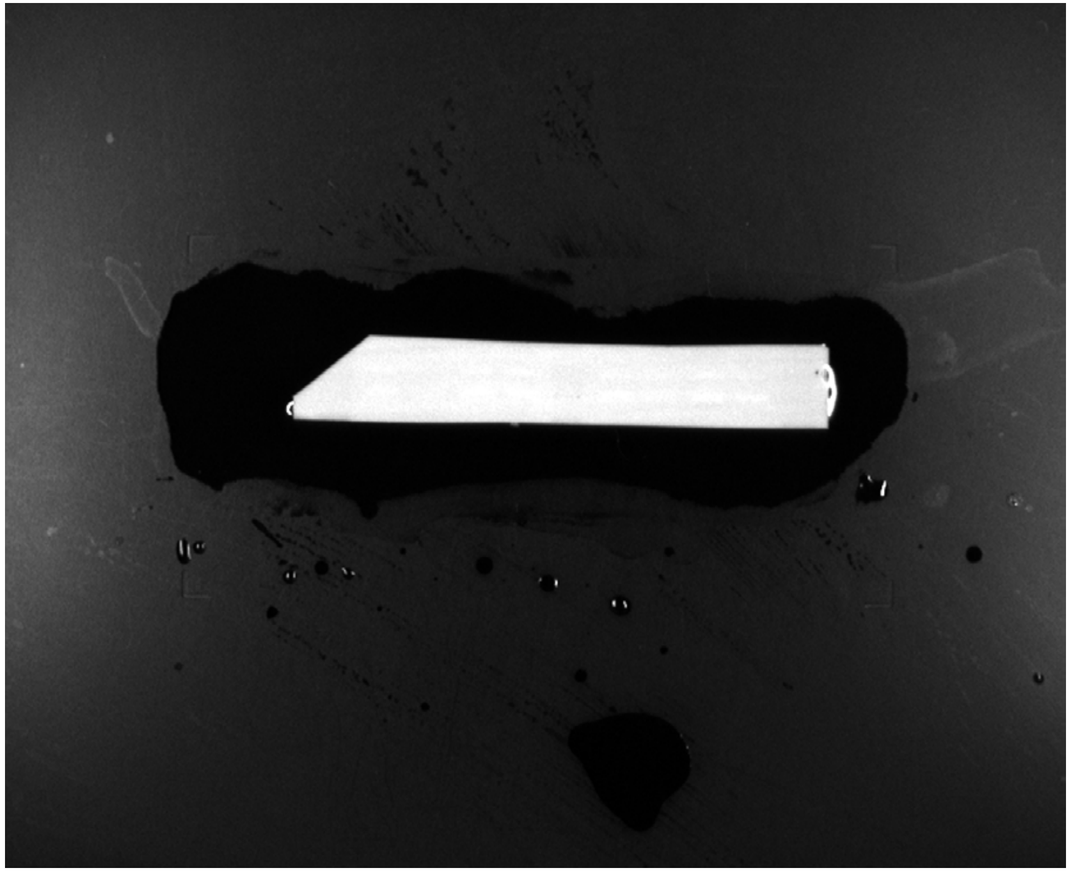

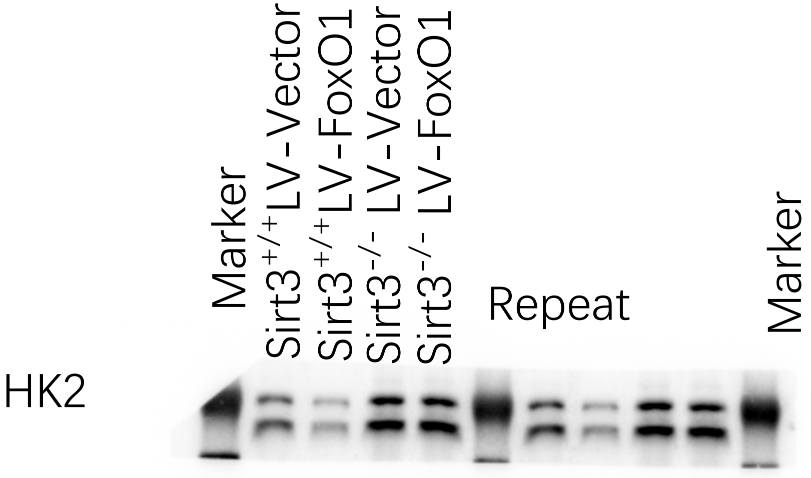
HK2
Marker
Sirt3$^{+/+}$LV-Vector
Sirt3$^{+/+}$LV-FoxO1
Sirt3$^{-/-}$ LV-Vector
Sirt3$^{-/-}$ LV-FoxO1
Repeat
Marker

Fig.6f

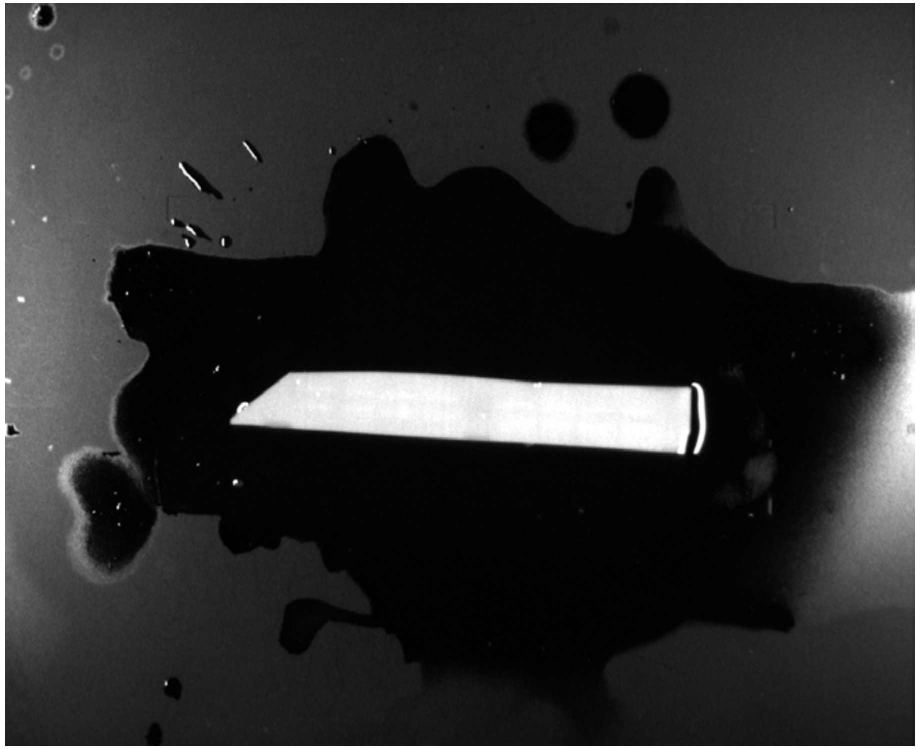

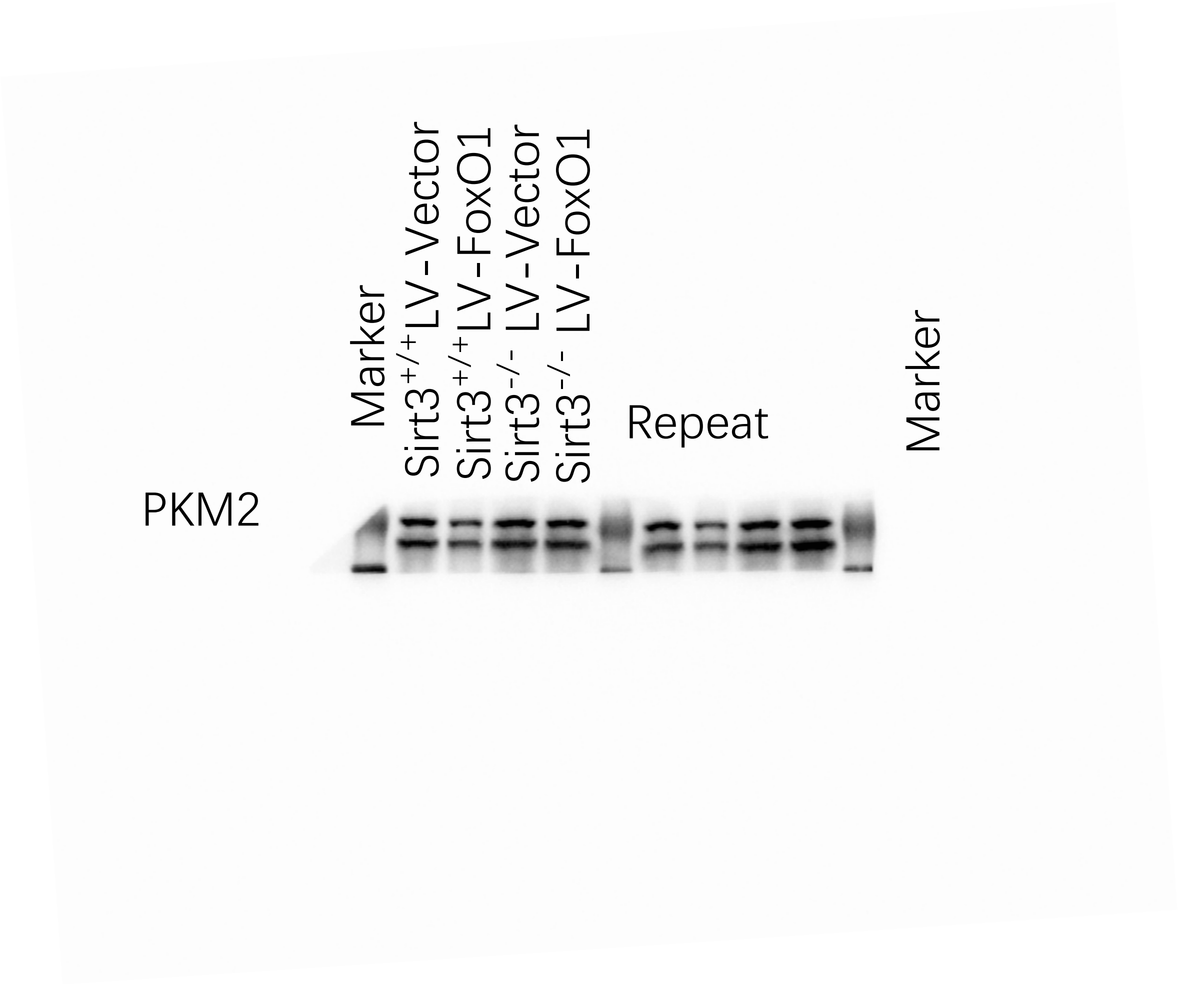
Marker
Sirt3+/+LV-Vector
Sirt3+/+LV-FoxO1
Sirt3-/- LV-Vector
Sirt3-/- LV-FoxO1
Repeat
Marker
PKM2

Fig.6f

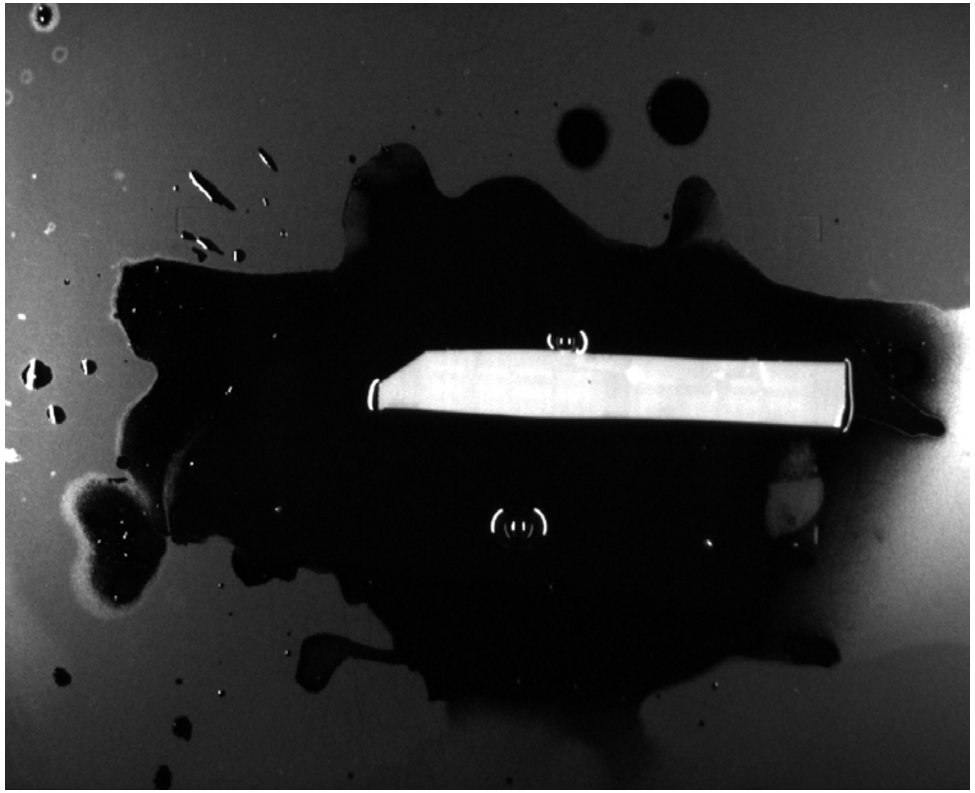

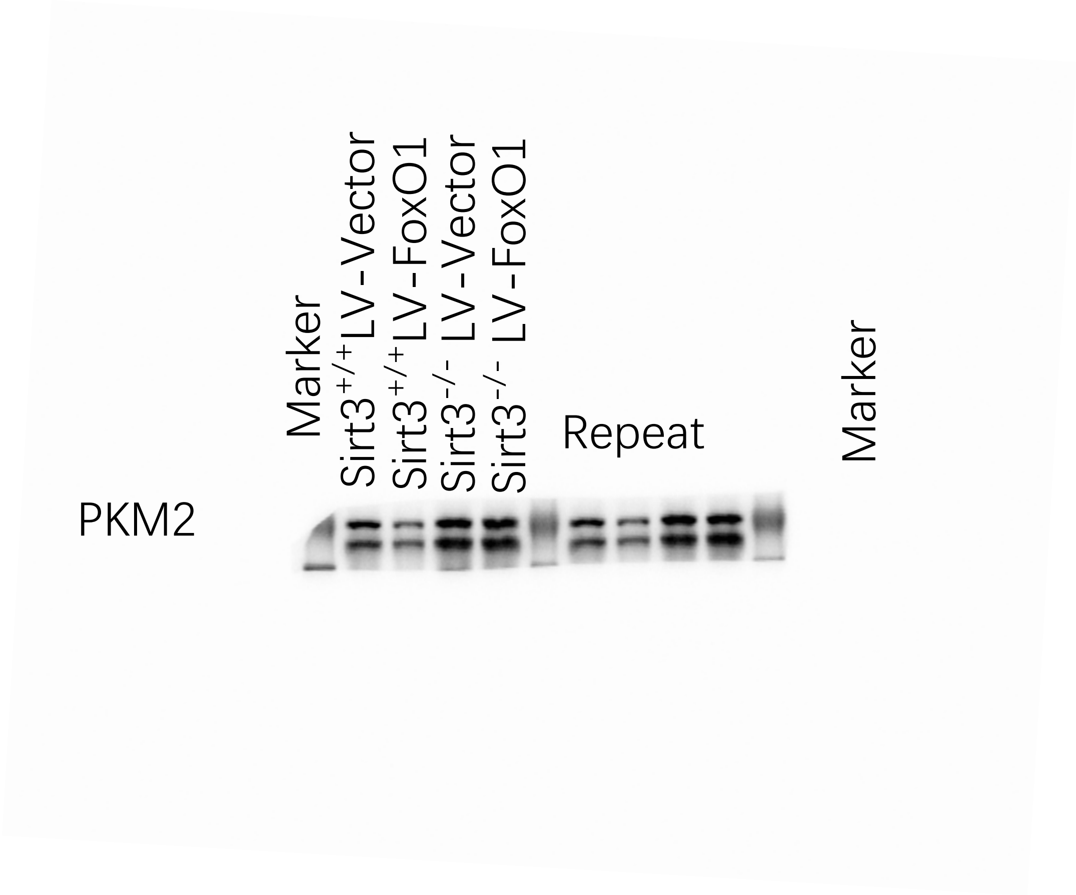
Marker
Sirt3+/+LV-Vector
Sirt3+/+LV-FoxO1
Sirt3-/- LV-Vector
Sirt3-/- LV-FoxO1
Repeat
Marker
PKM2

Fig.6f

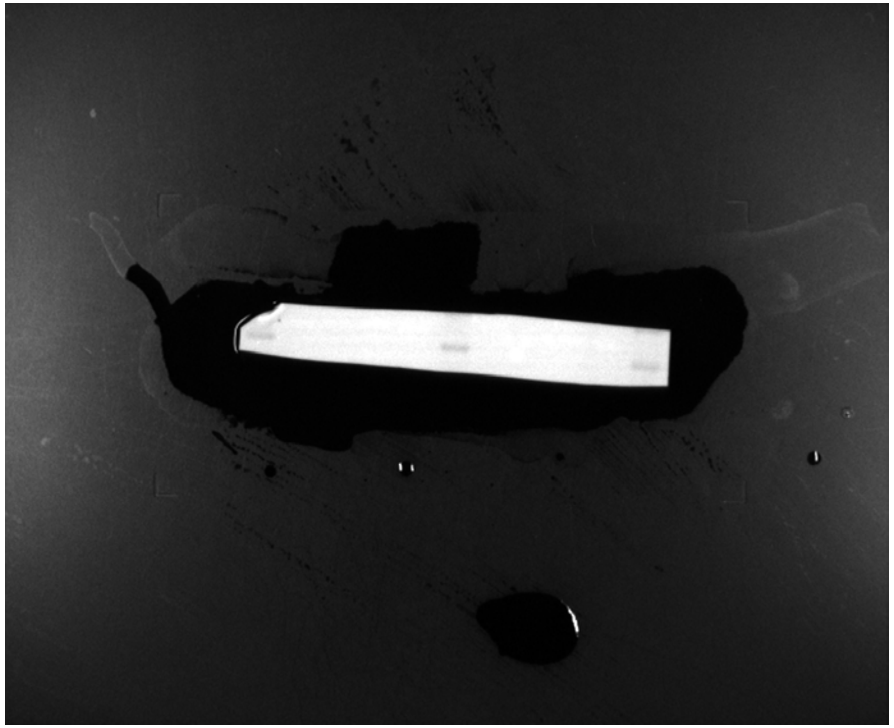

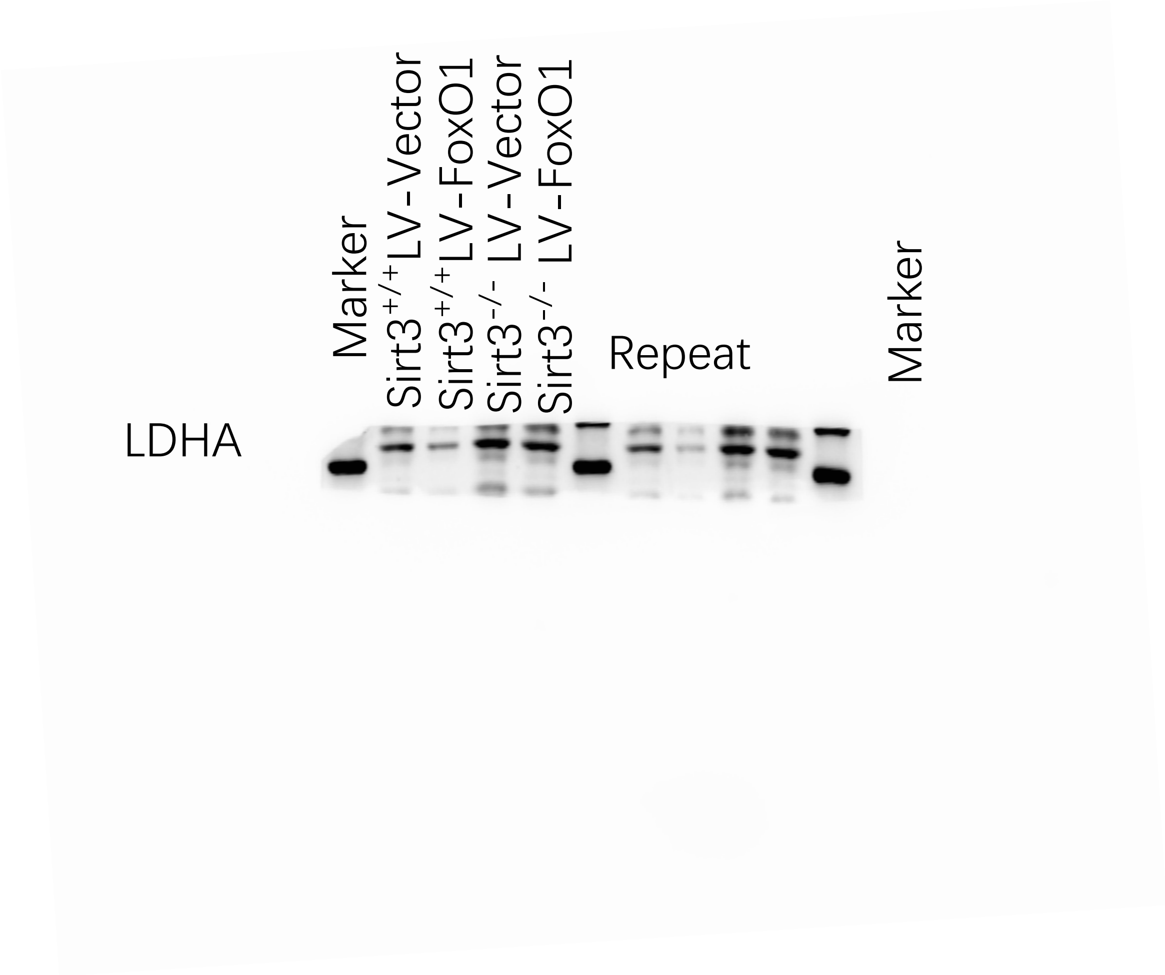

LDHA
Marker
Sirt3+/+ LV-Vector
Sirt3+/+ LV-FoxO1
Sirt3-/- LV-Vector
Sirt3-/- LV-FoxO1
Repeat
Marker

Fig.6f

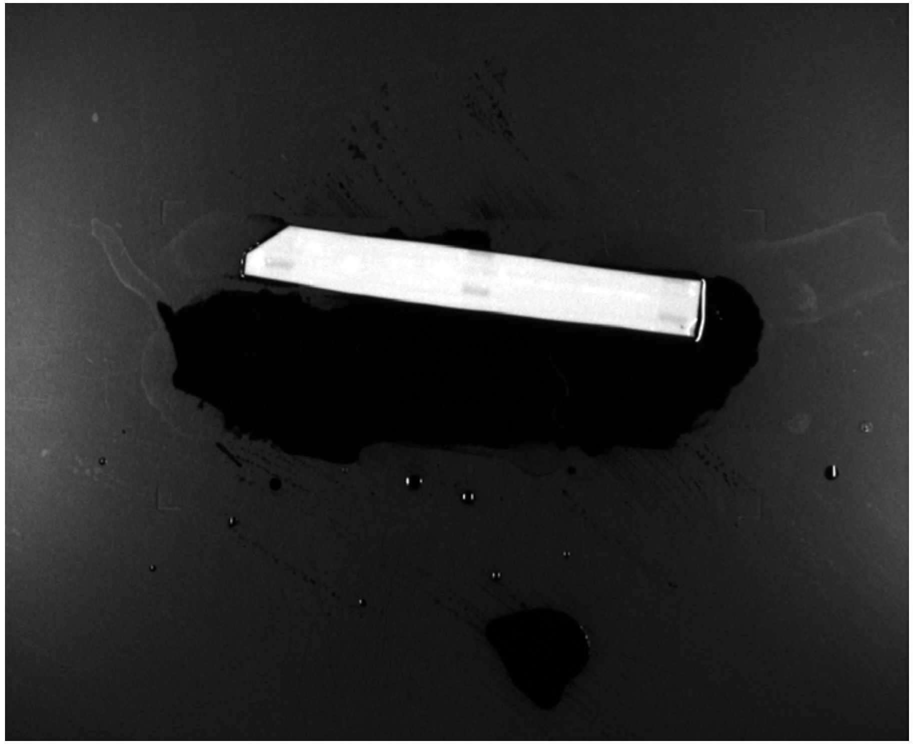

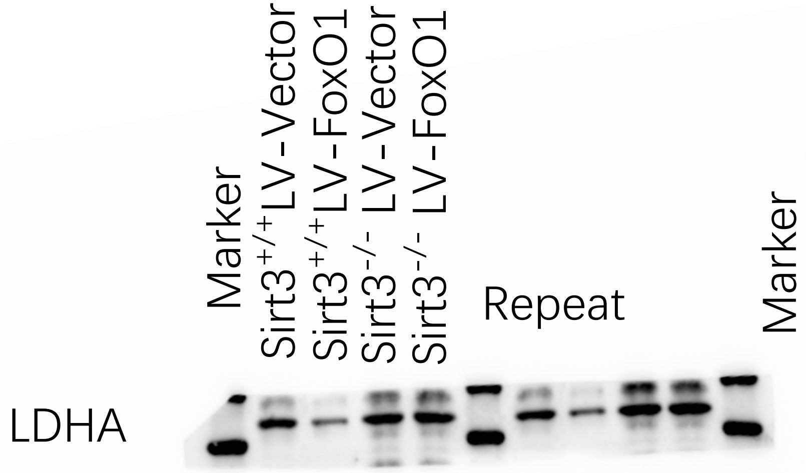

Marker
Sirt3+/+LV-Vector
Sirt3+/+LV-FoxO1
Sirt3-/- LV-Vector
Sirt3-/- LV-FoxO1
Repeat
Marker
LDHA

Fig.6h

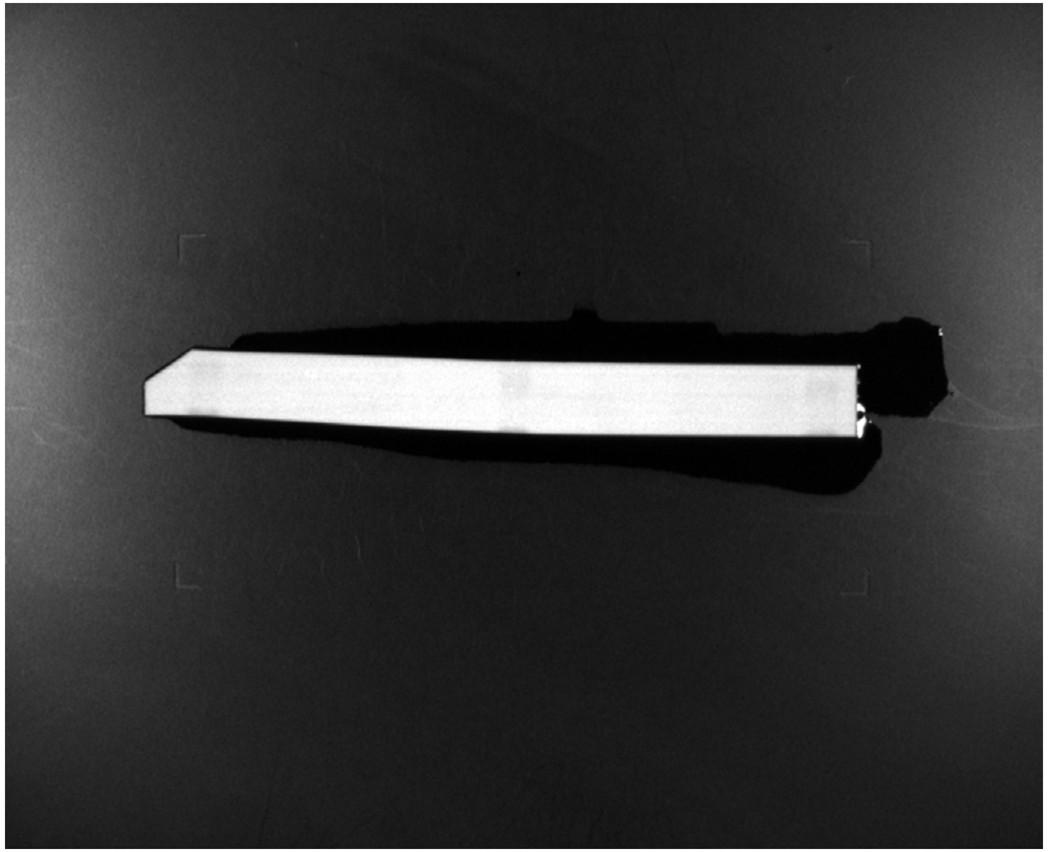

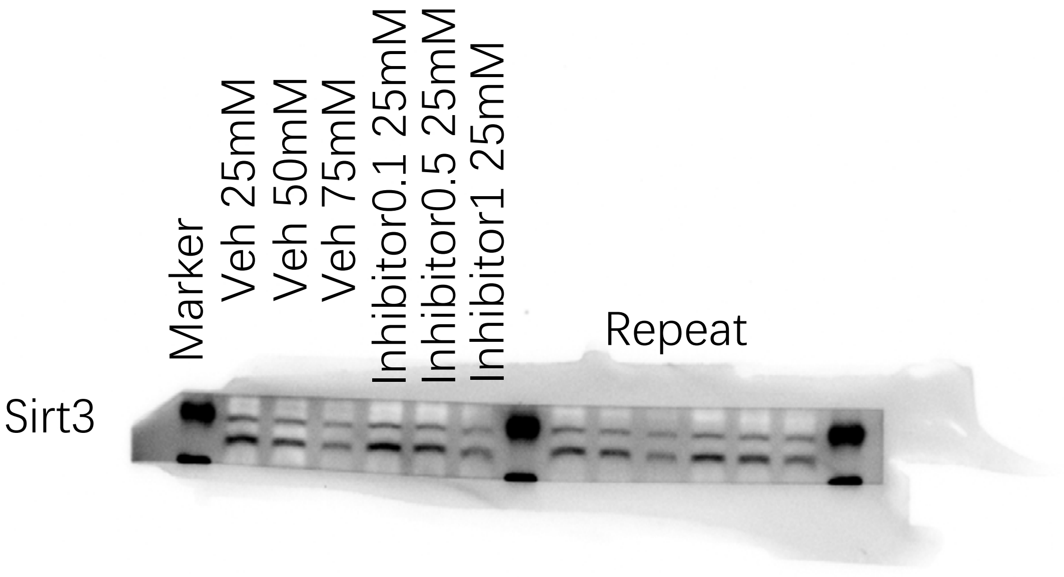
Marker
Veh 25mM
Veh 50mM
Veh 75mM
Inhibitor0.1 25mM
Inhibitor0.5 25mM
Inhibitor1 25mM
Repeat
Sirt3

Fig.6h

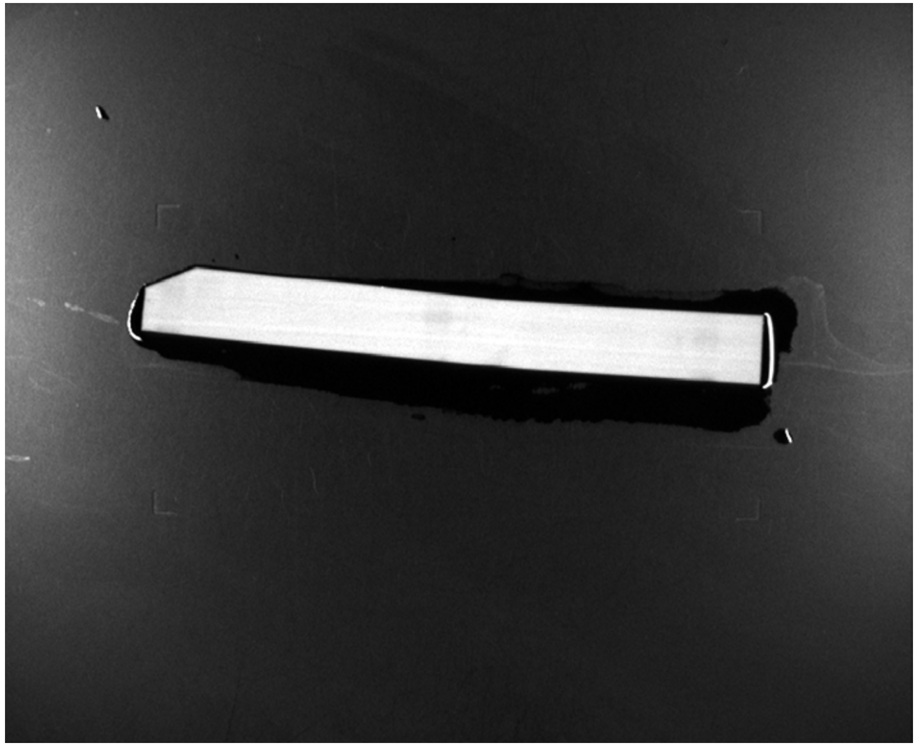

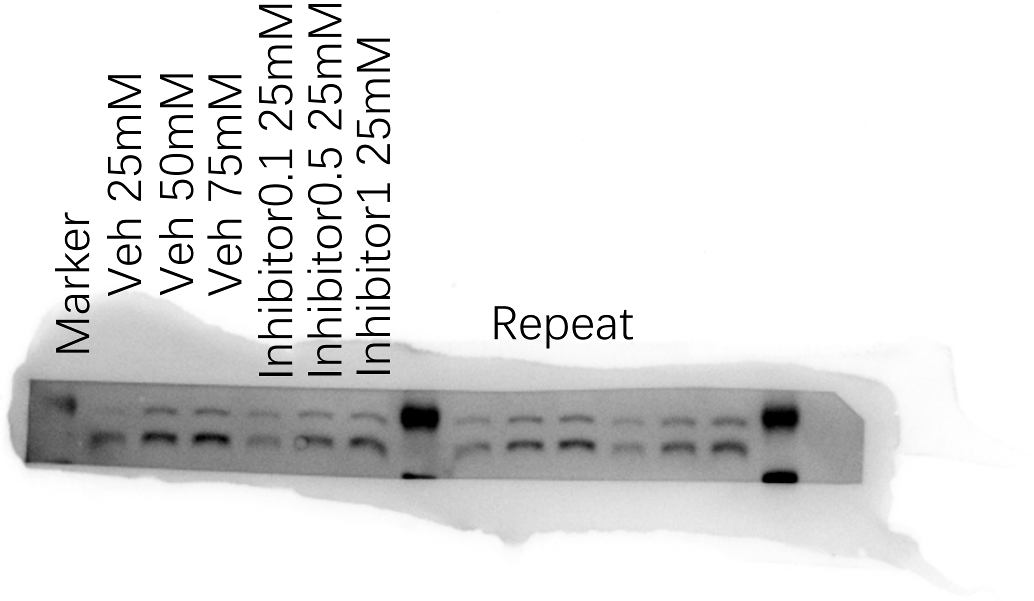
Marker
Veh 25mM
Veh 50mM
Veh 75mM
Inhibitor0.1 25mM
Inhibitor0.5 25mM
Inhibitor1 25mM
Repeat
Sirt3

Fig.6h

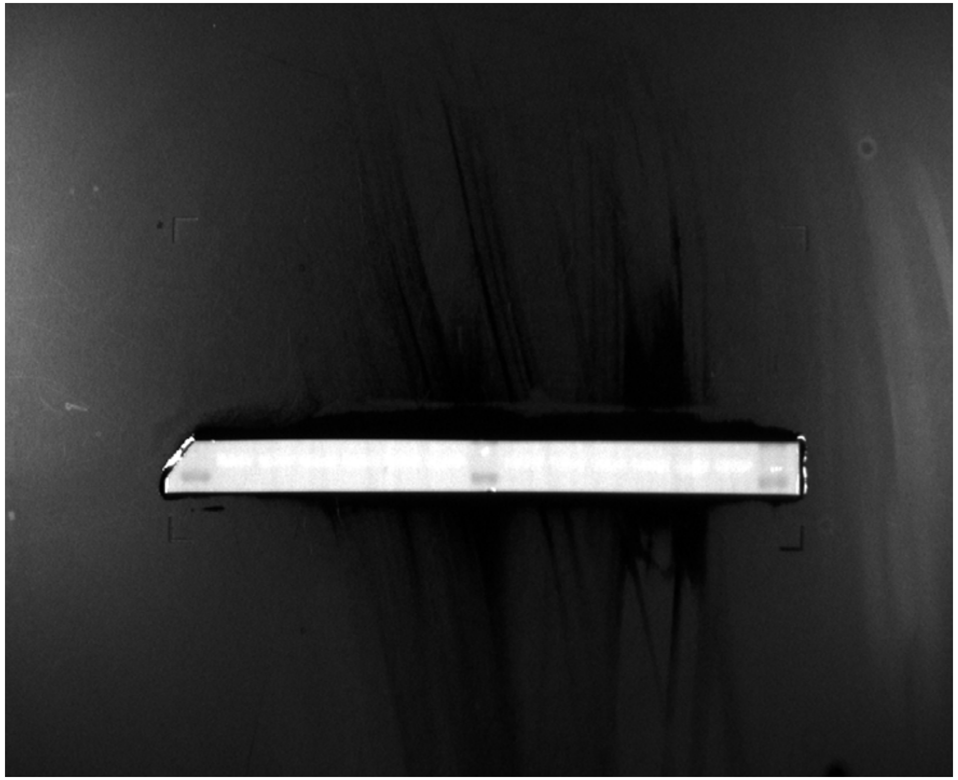

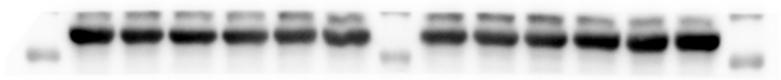
Marker
Veh 25mM
Veh 50mM
Veh 75mM
Inhibitor0.1 25mM
Inhibitor0.5 25mM
Inhibitor1 25mM
Repeat
Actin

Fig.6h

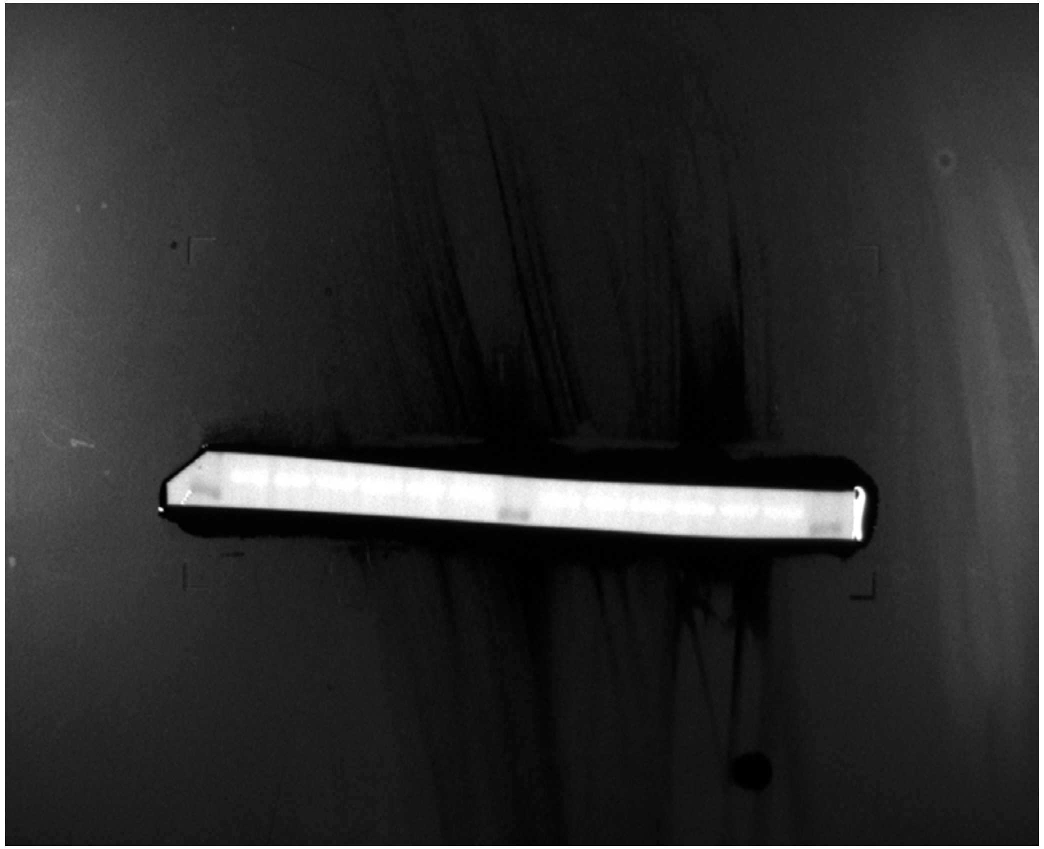

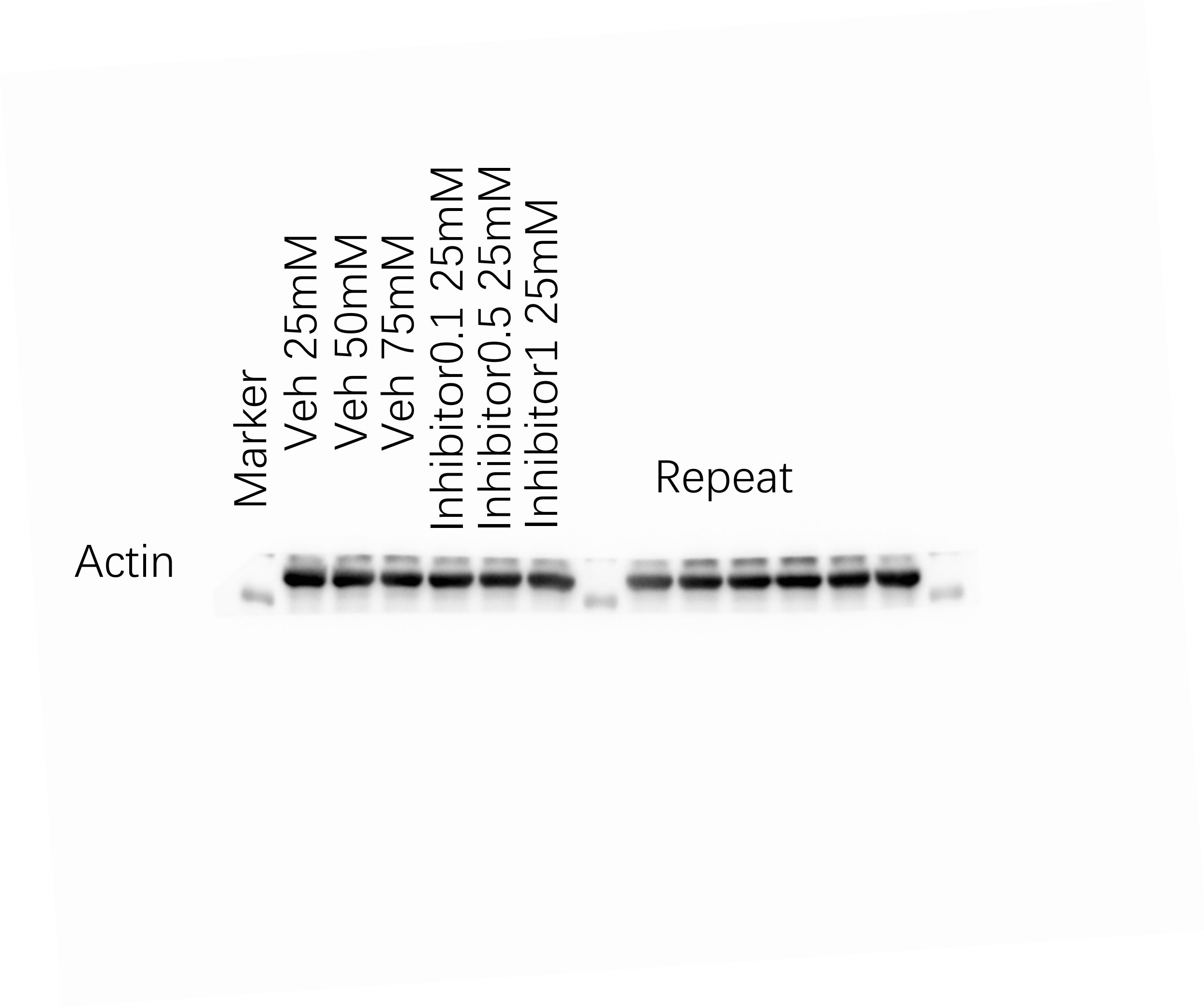
Marker
Veh 25mM
Veh 50mM
Veh 75mM
Inhibitor0.1 25mM
Inhibitor0.5 25mM
Inhibitor1 25mM
Repeat
Actin

Fig.7a

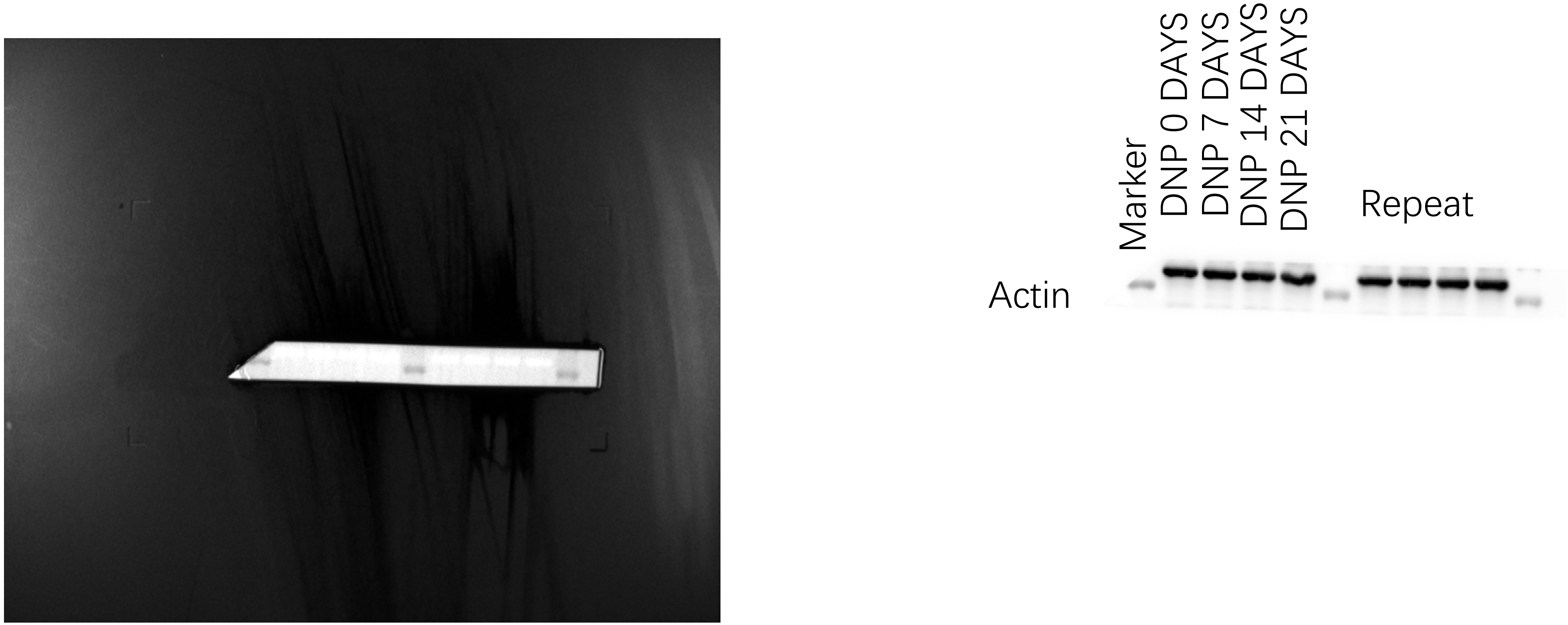
Marker
DNP 0 DAYS
DNP 7 DAYS
DNP 14 DAYS
DNP 21 DAYS
Repeat
Actin

Fig.7a

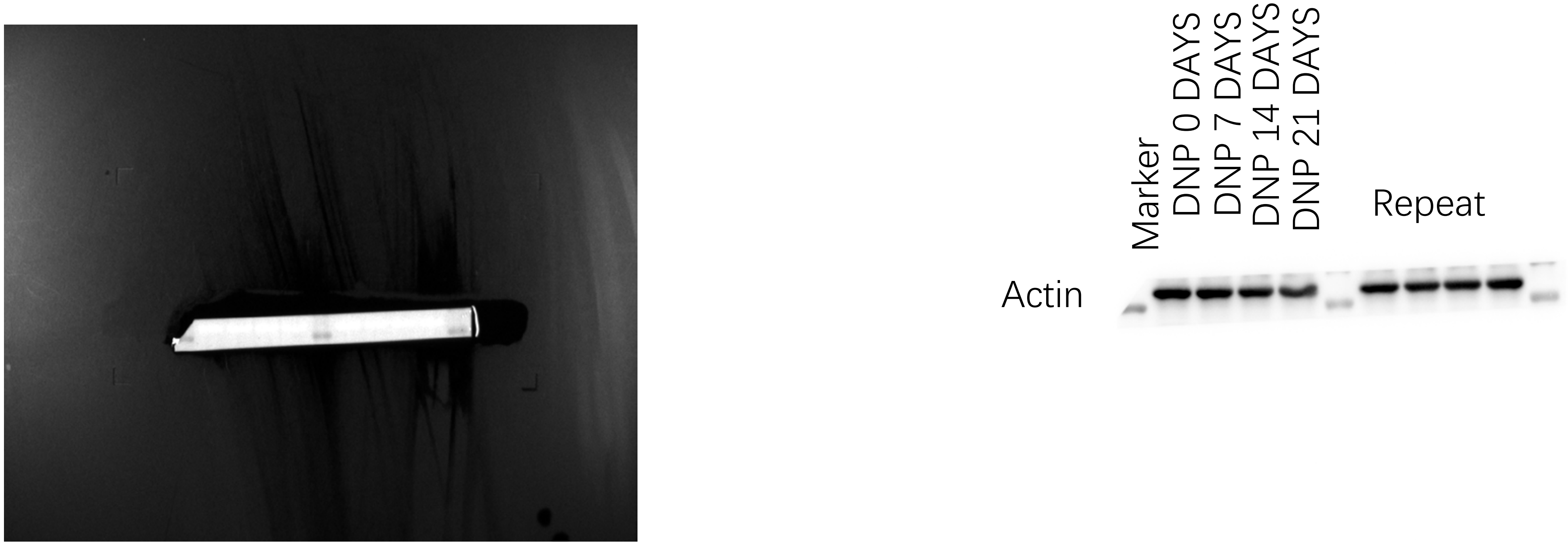
Marker
DNP 0 DAYS
DNP 7 DAYS
DNP 14 DAYS
DNP 21 DAYS
Repeat
Actin

Fig.7a

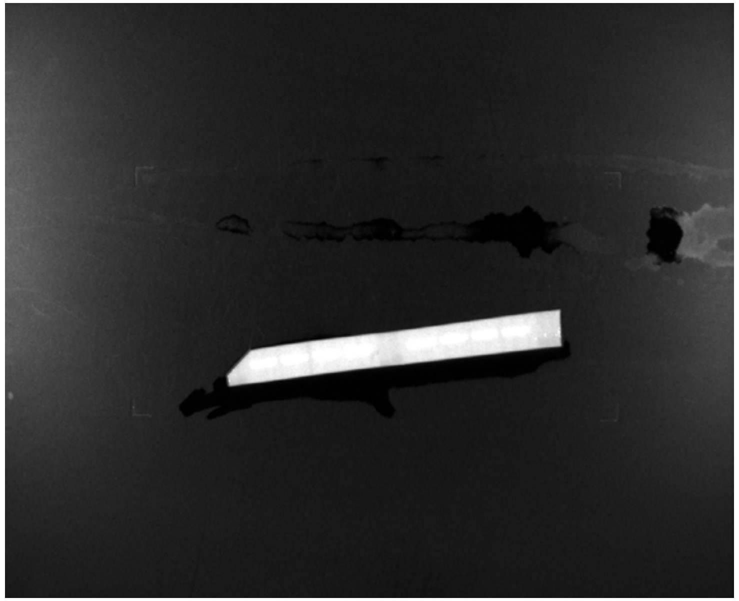

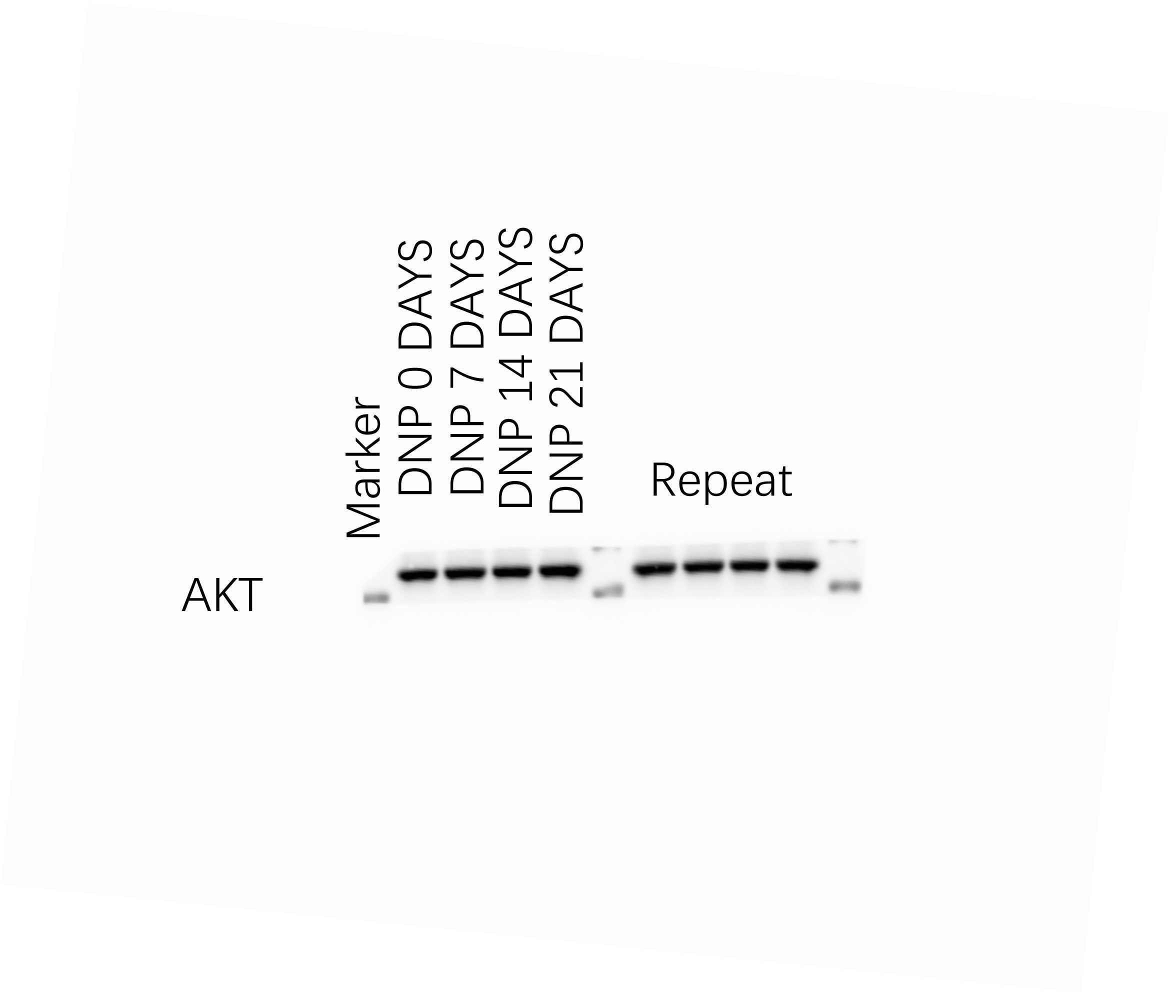

Fig.7a

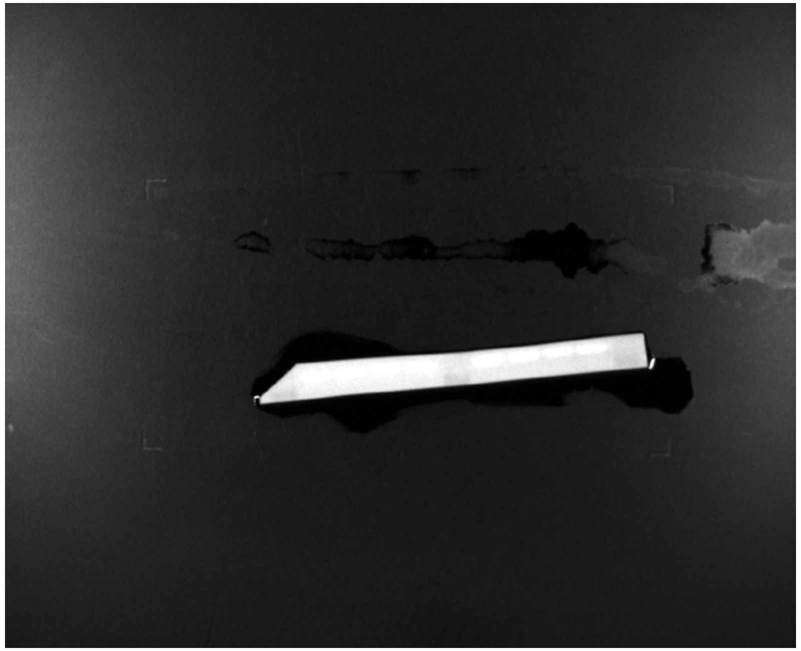

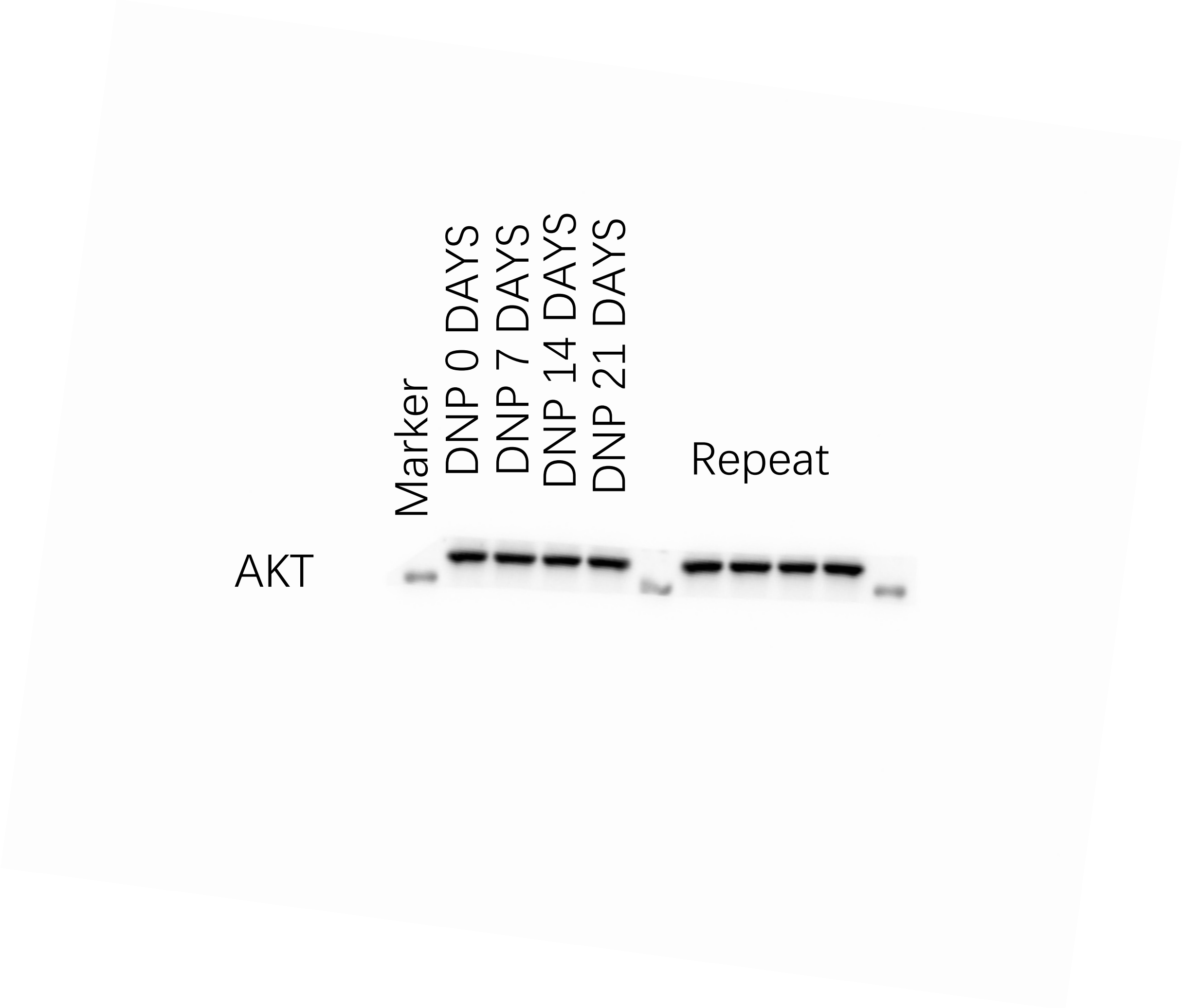

Fig.7a

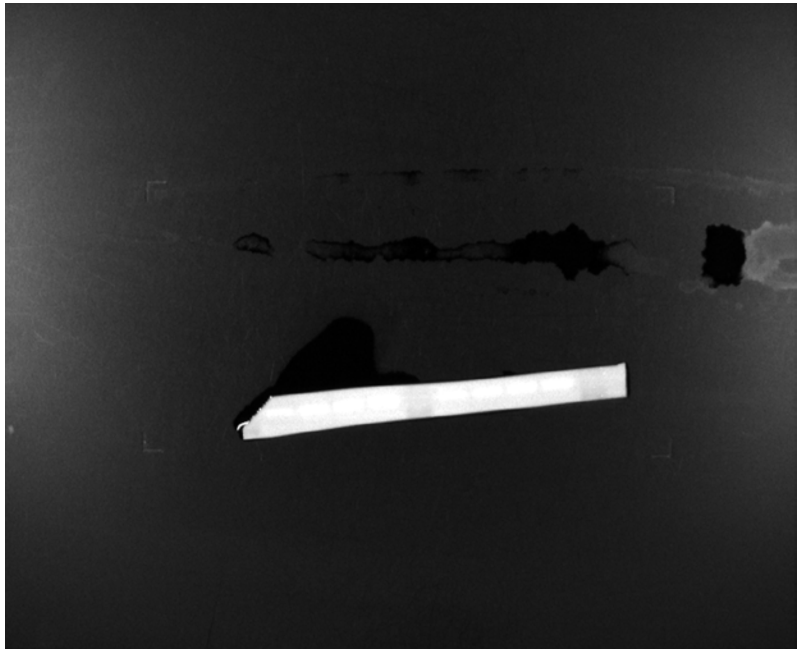

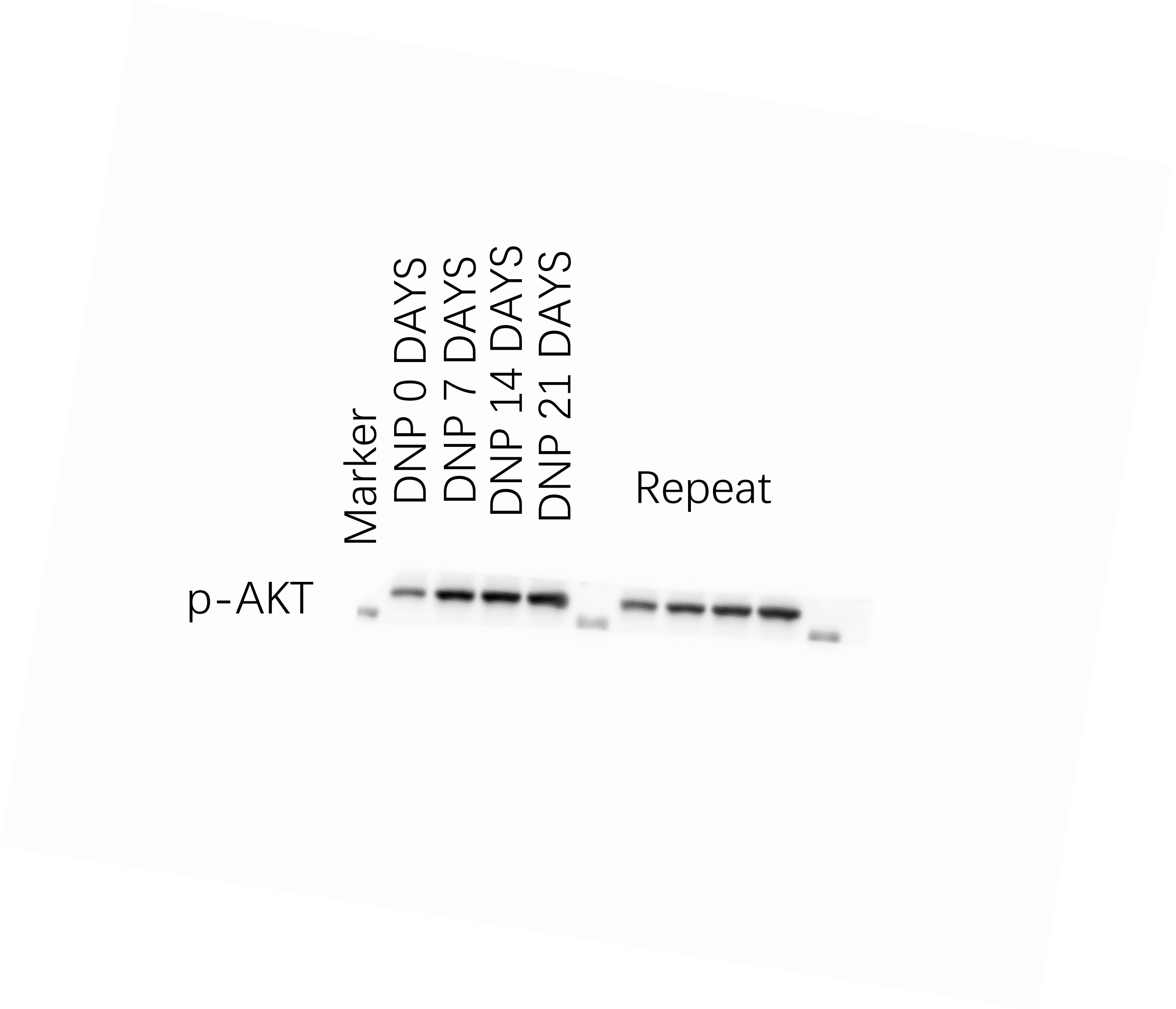
Marker
DNP 0 DAYS
DNP 7 DAYS
DNP 14 DAYS
DNP 21 DAYS
Repeat
p-AKT

Fig.7a

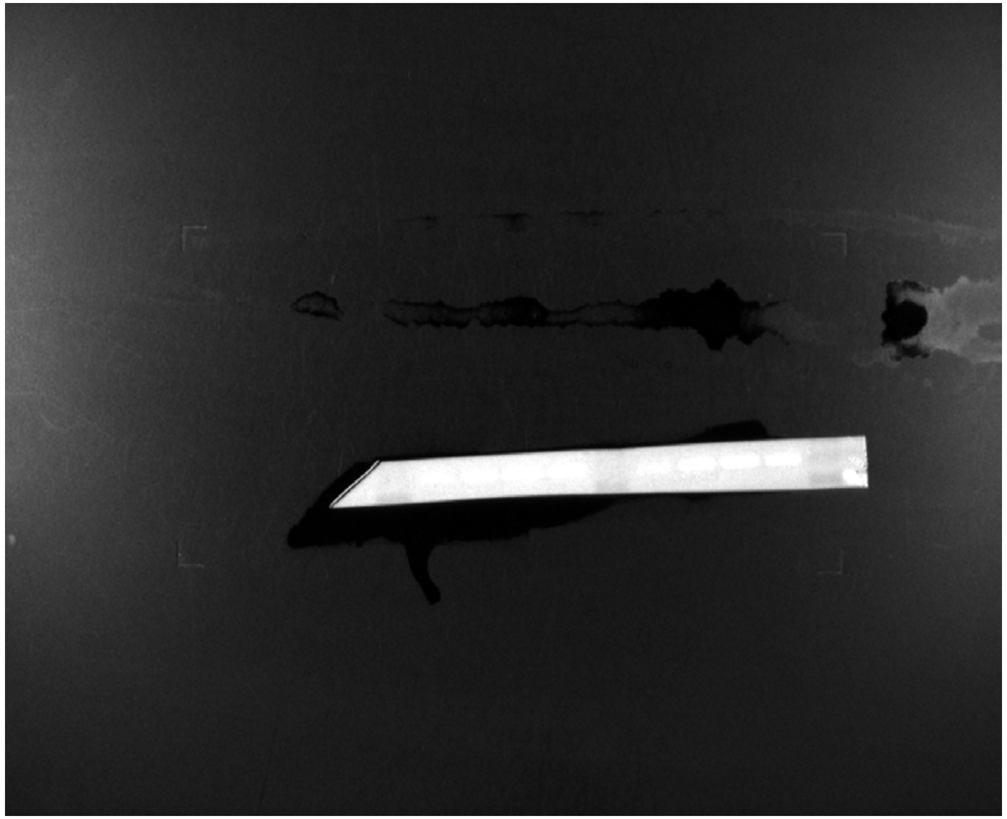

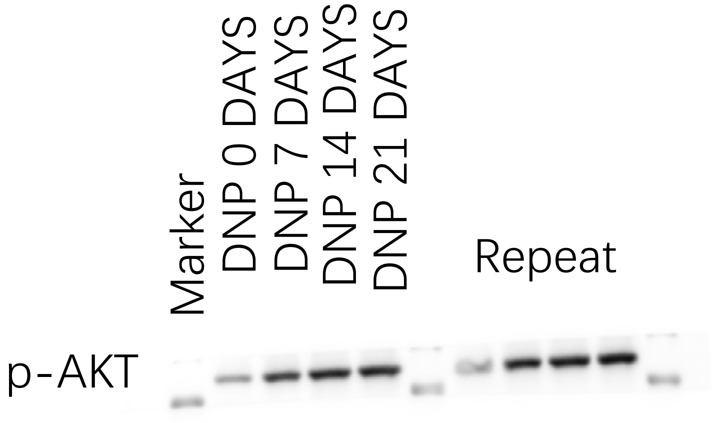
Marker
DNP 0 DAYS
DNP 7 DAYS
DNP 14 DAYS
DNP 21 DAYS
Repeat
p-AKT

Fig.7a

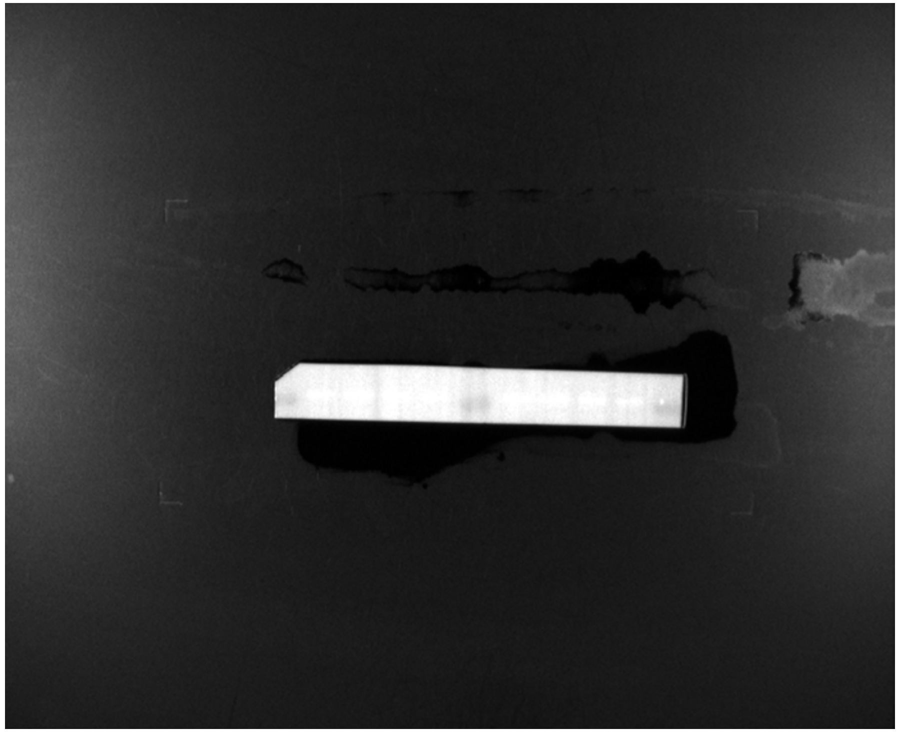

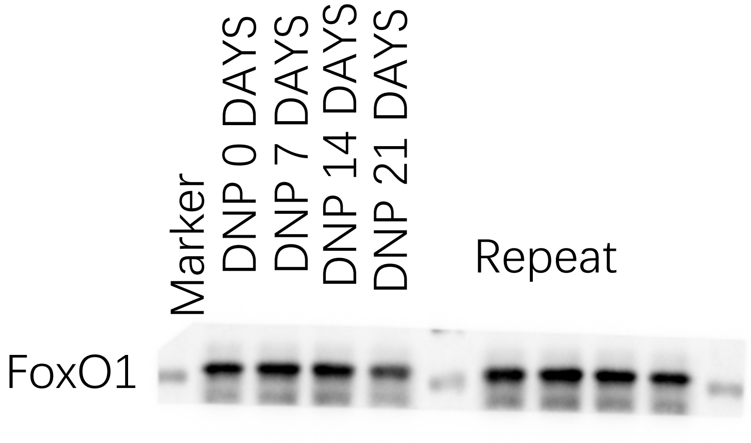
Marker
DNP 0 DAYS
DNP 7 DAYS
DNP 14 DAYS
DNP 21 DAYS
Repeat
FoxO1

Fig.7a

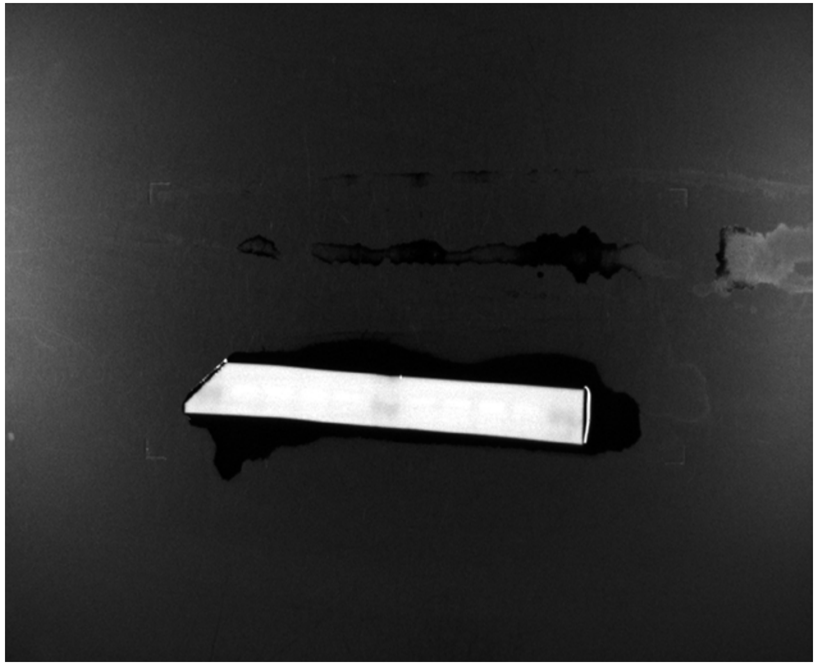

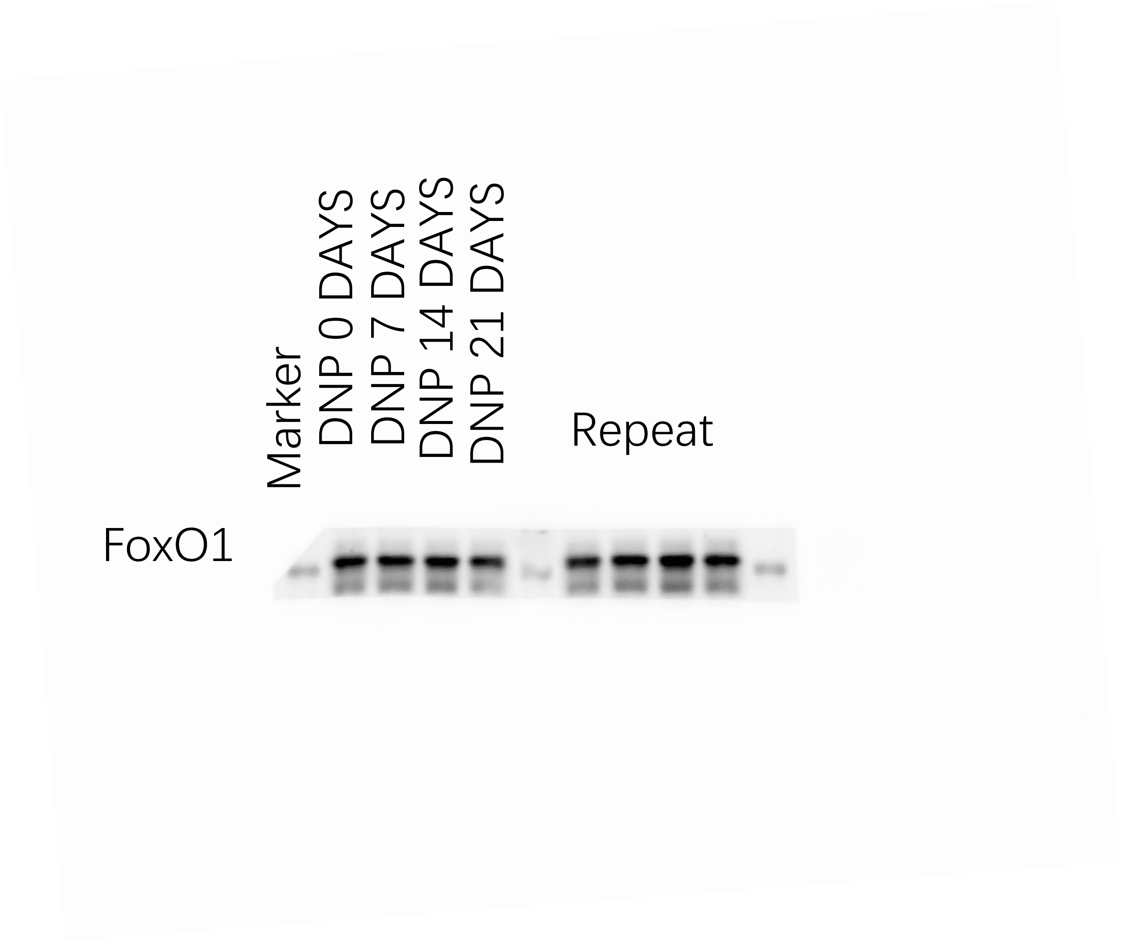
Marker
DNP 0 DAYS
DNP 7 DAYS
DNP 14 DAYS
DNP 21 DAYS
Repeat
FoxO1

Fig.7a

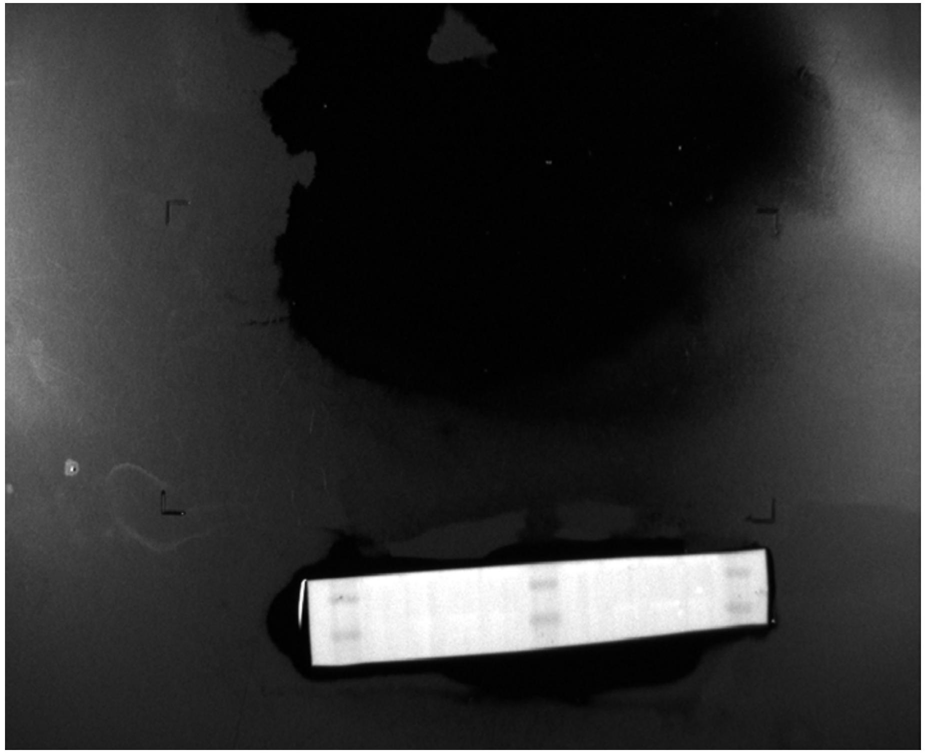

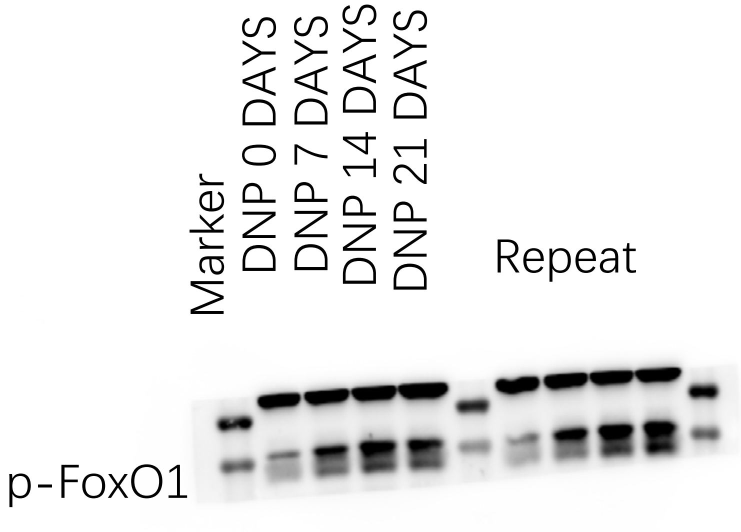
Marker
DNP 0 DAYS
DNP 7 DAYS
DNP 14 DAYS
DNP 21 DAYS
Repeat
p-FoxO1

Fig.7a

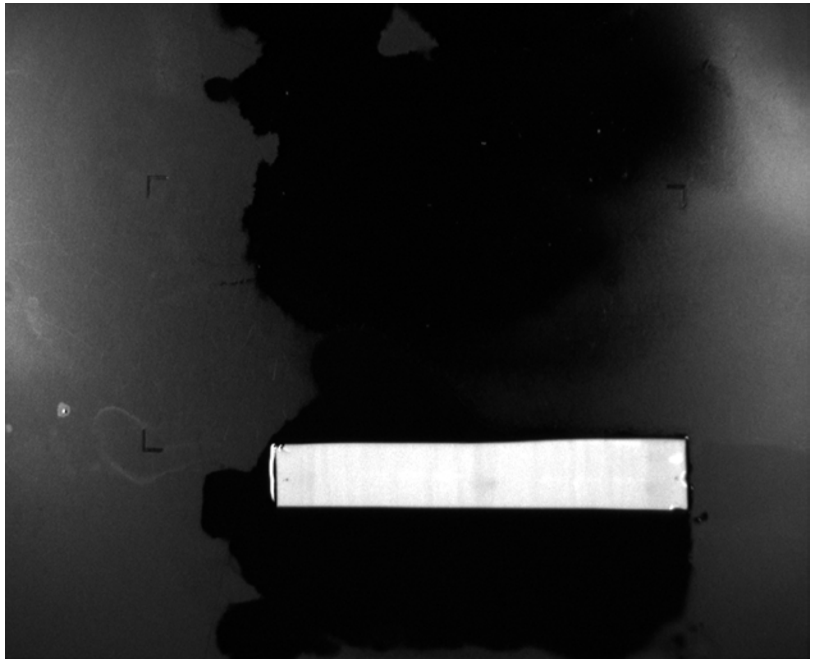

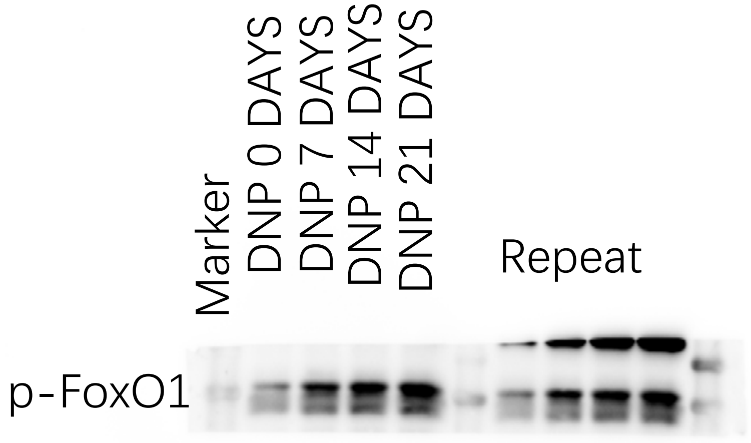
Marker
DNP 0 DAYS
DNP 7 DAYS
DNP 14 DAYS
DNP 21 DAYS
Repeat
p-FoxO1

Fig.7b

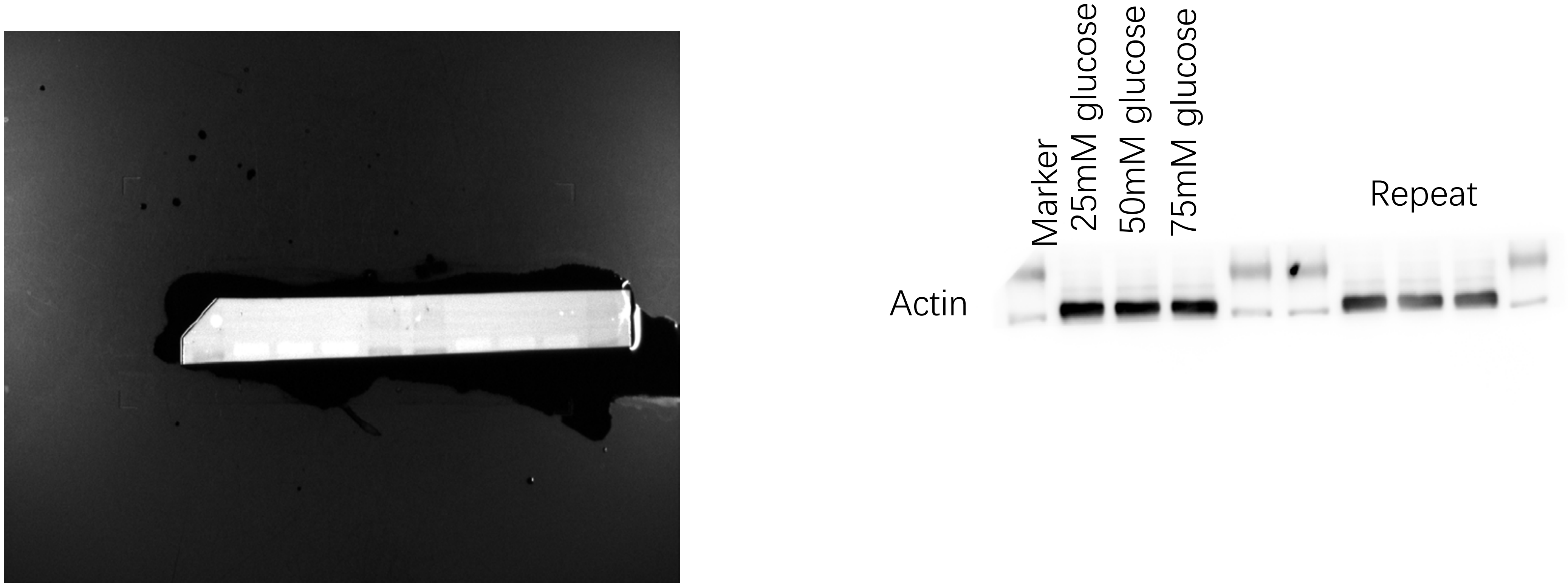
Marker
25mM glucose
50mM glucose
75mM glucose
Repeat
Actin

Fig.7b

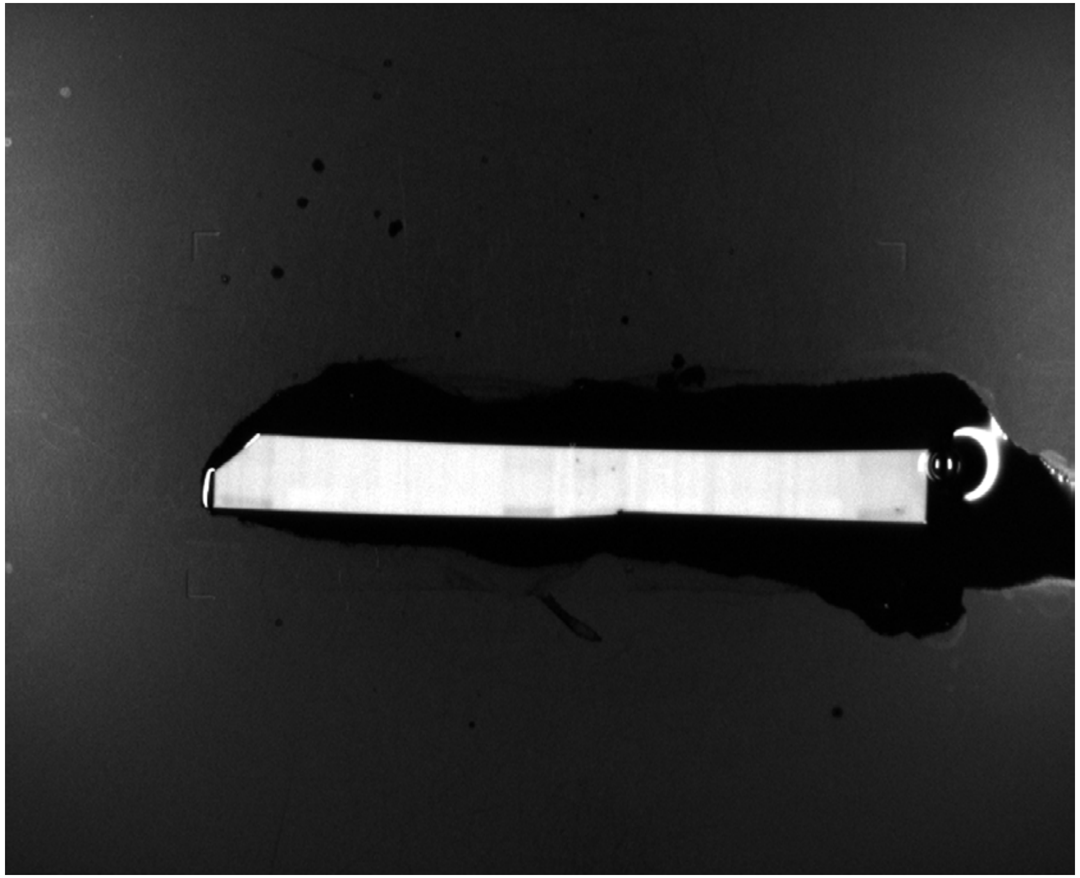

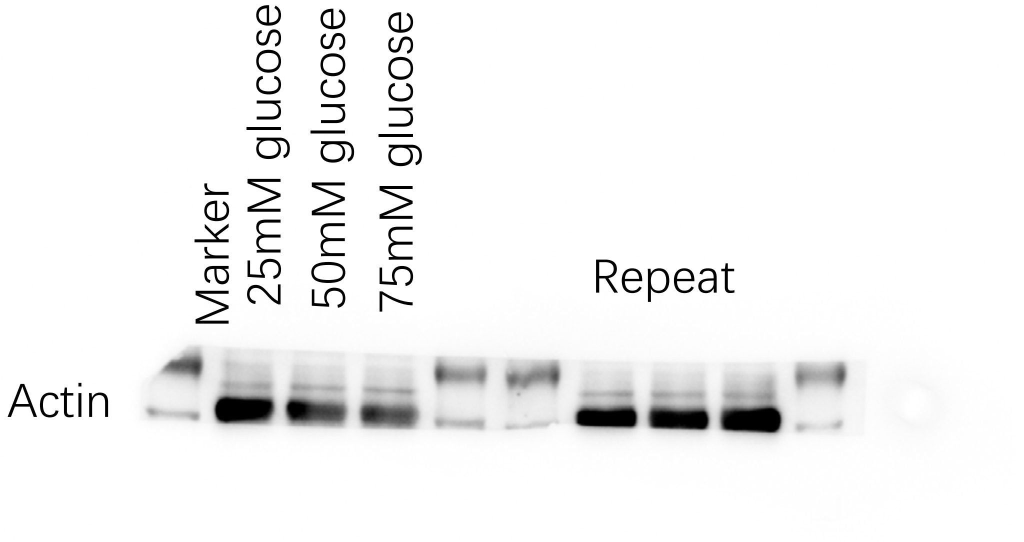

Marker
25mM glucose
50mM glucose
75mM glucose
Repeat
Actin

Fig.7b

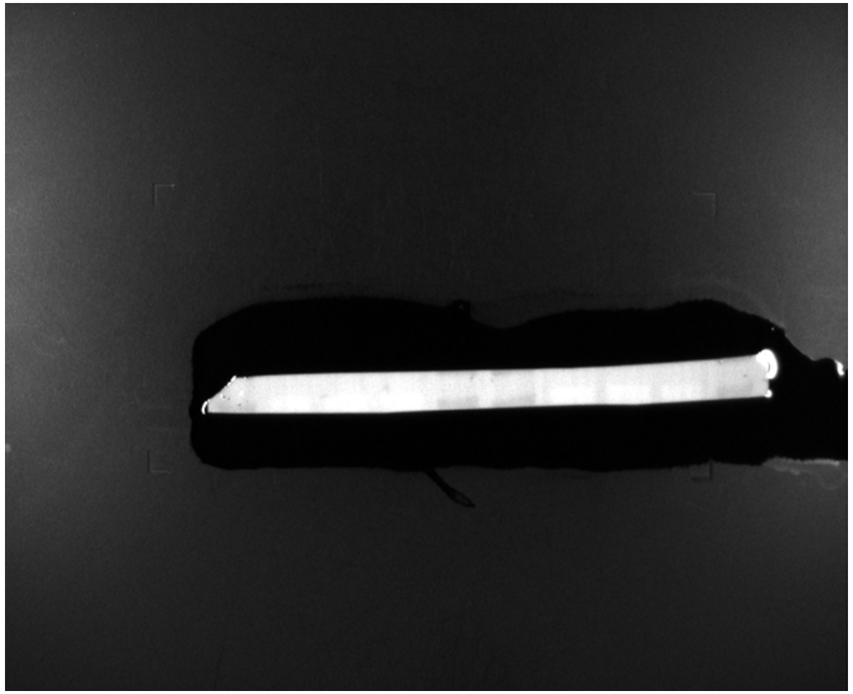

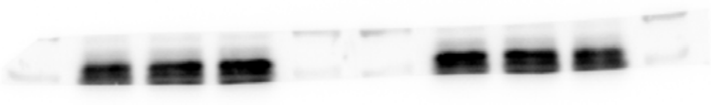

Marker
25mM glucose
50mM glucose
75mM glucose
Repeat
FoxO1

Fig.7b

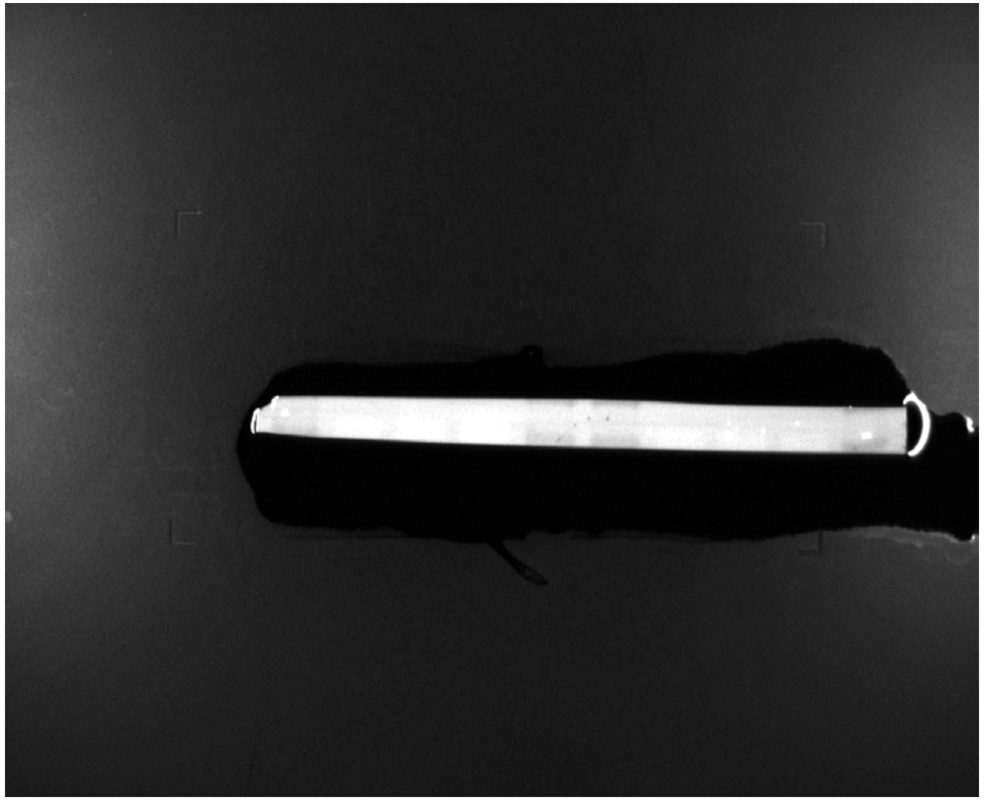

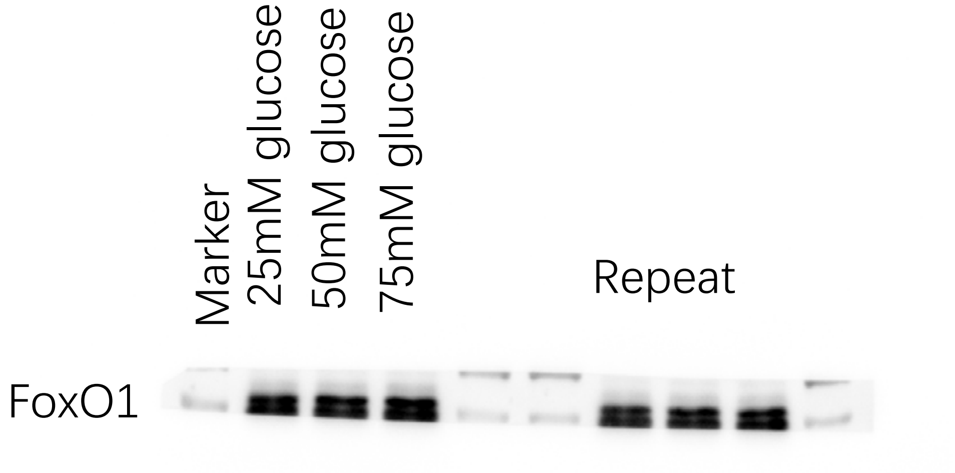
Marker
25mM glucose
50mM glucose
75mM glucose
Repeat
FoxO1

Fig.7b

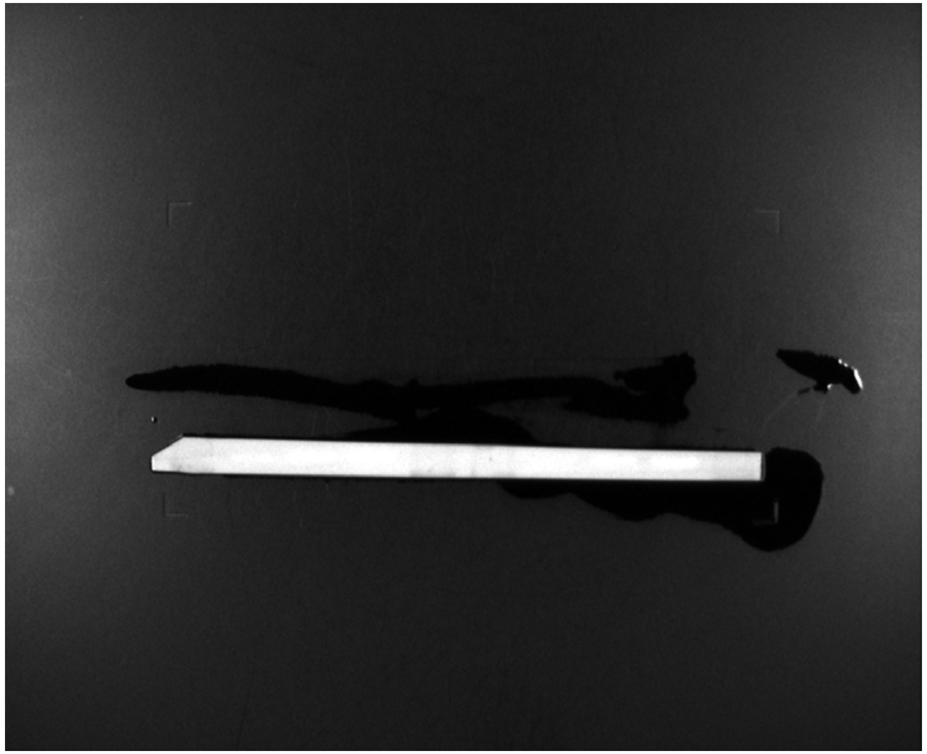

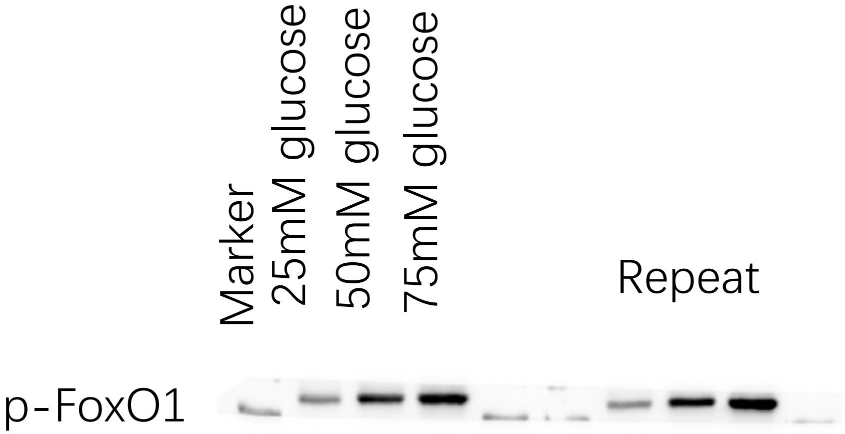
Marker
25mM glucose
50mM glucose
75mM glucose
Repeat
p-FoxO1

Fig.7b

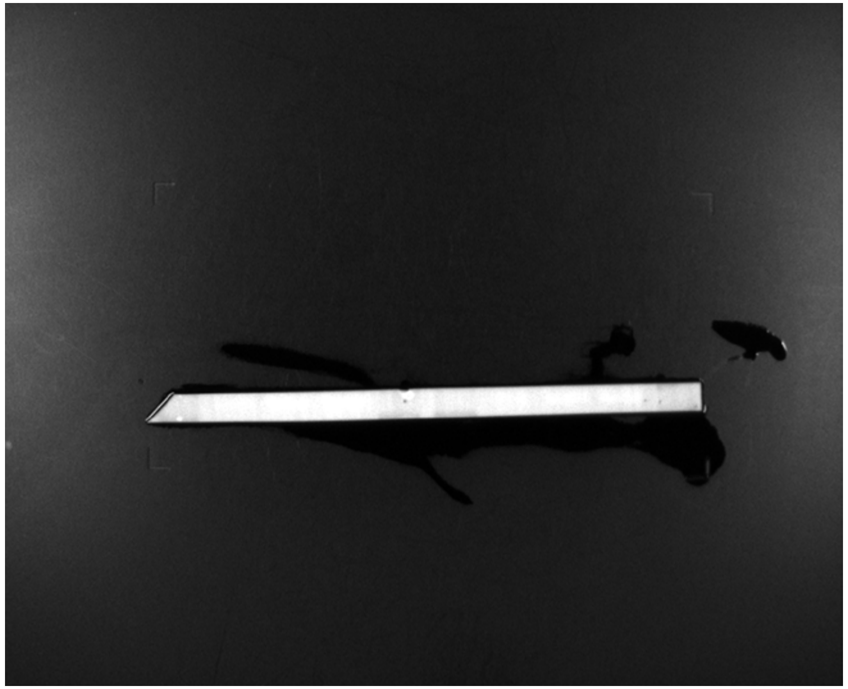

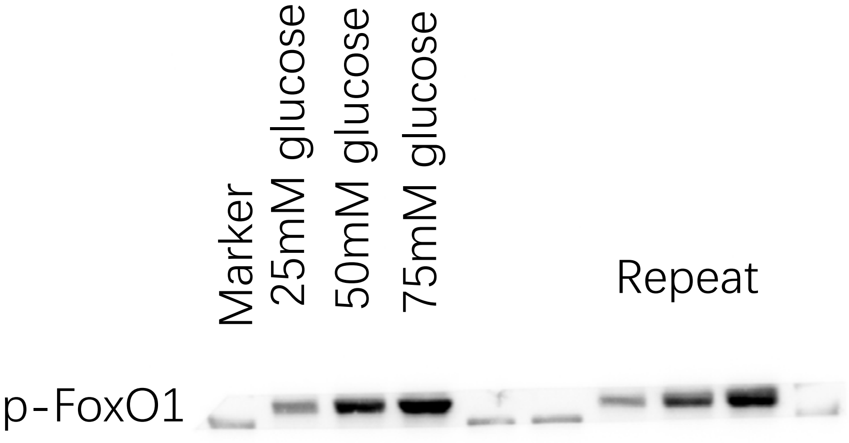
Marker
25mM glucose
50mM glucose
75mM glucose
Repeat
p-FoxO1

Fig.7b

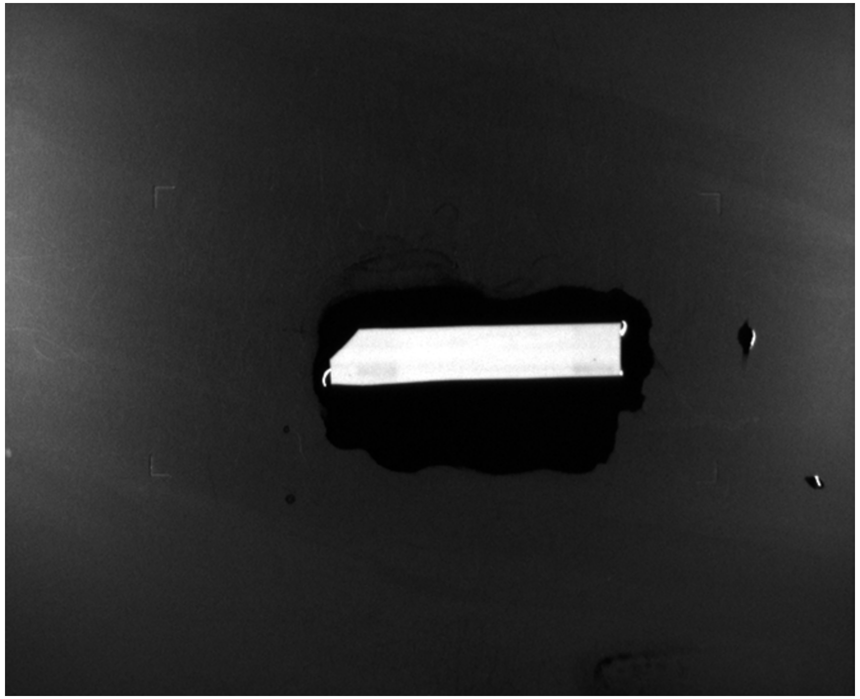

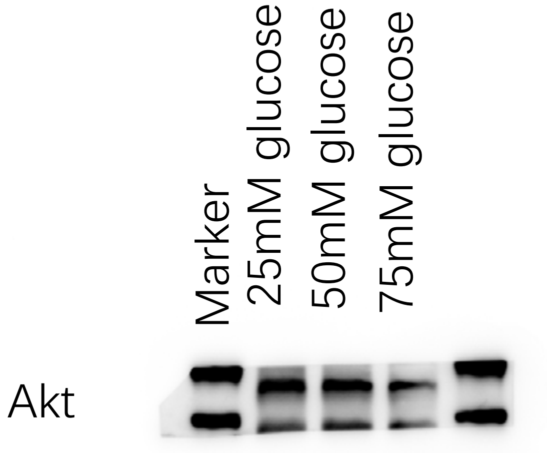

Fig.7b

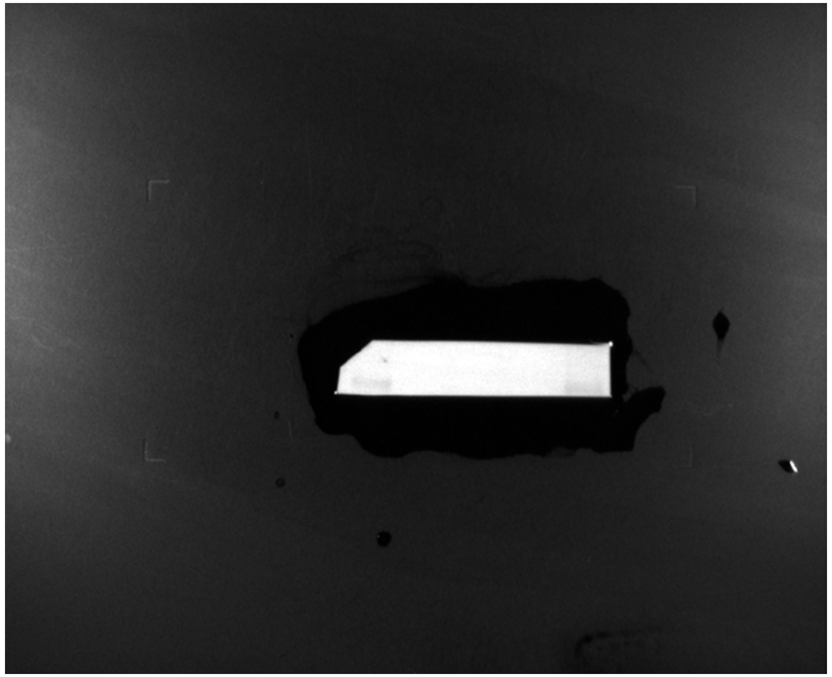

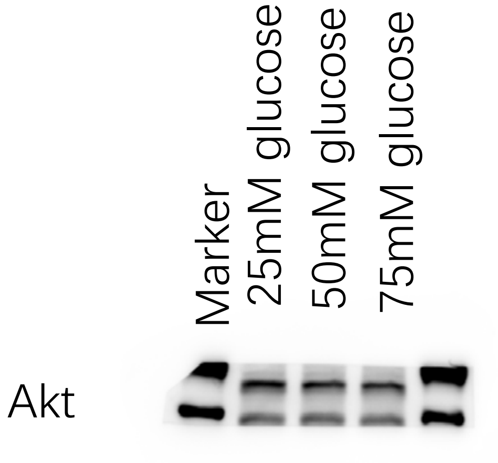

Fig.7b

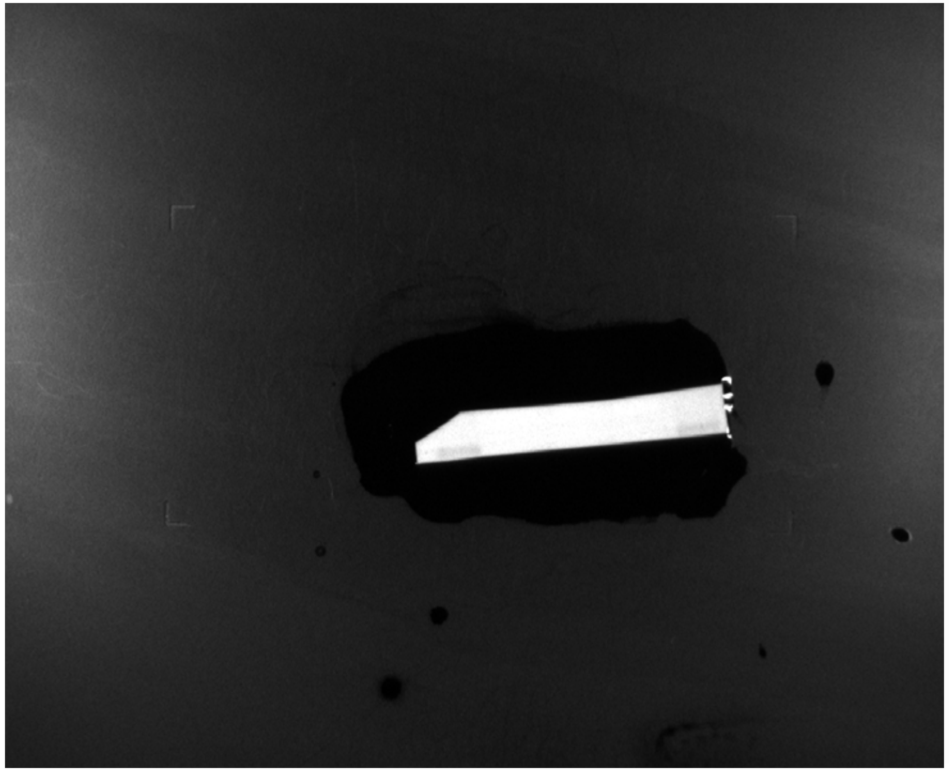

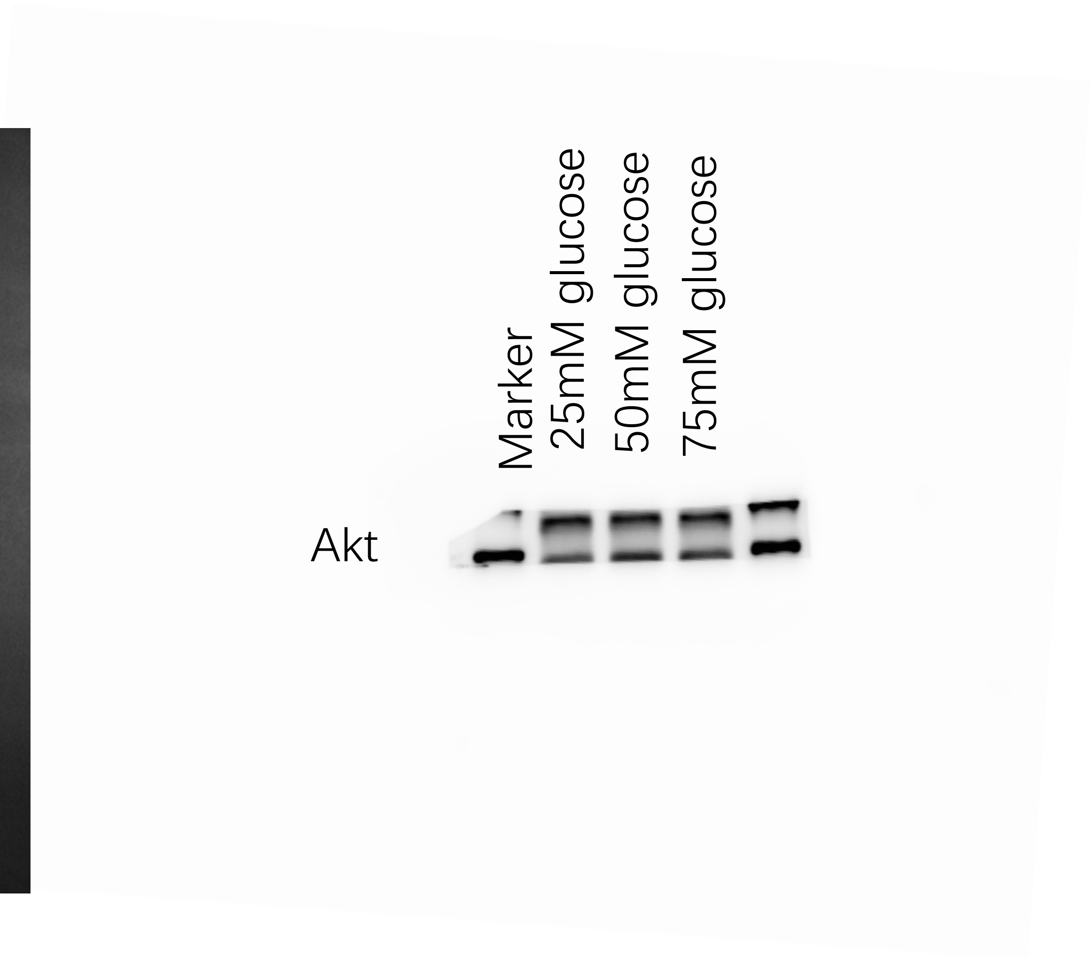

Fig.7b

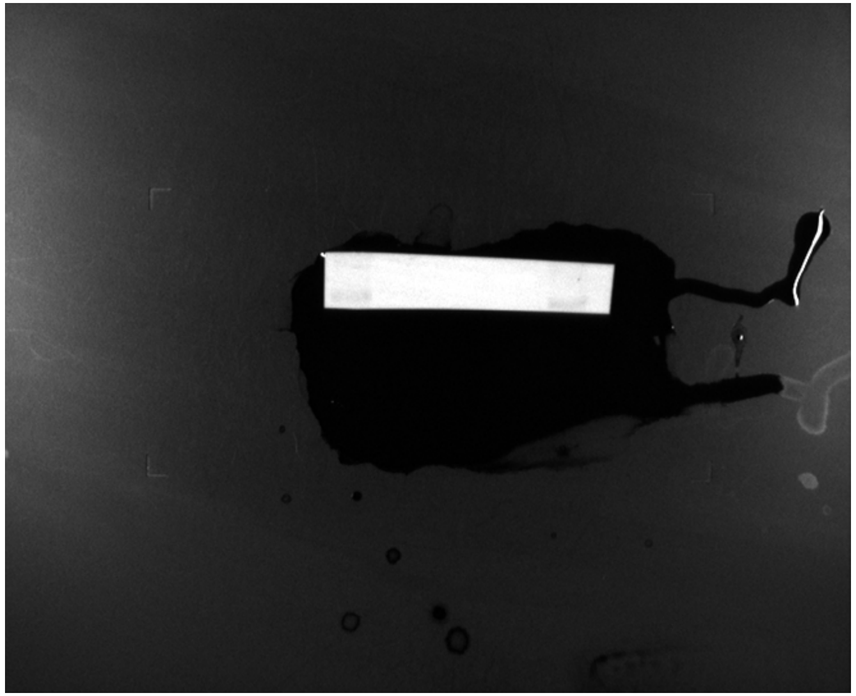

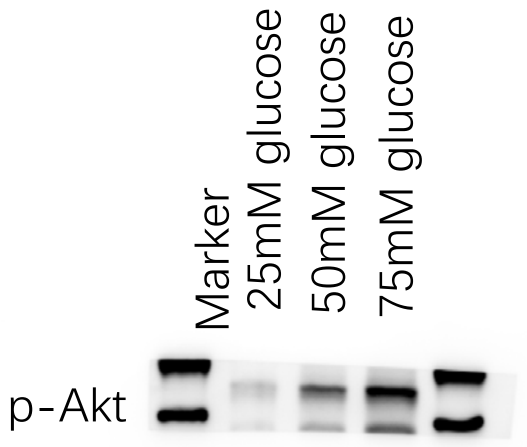

Fig.7b

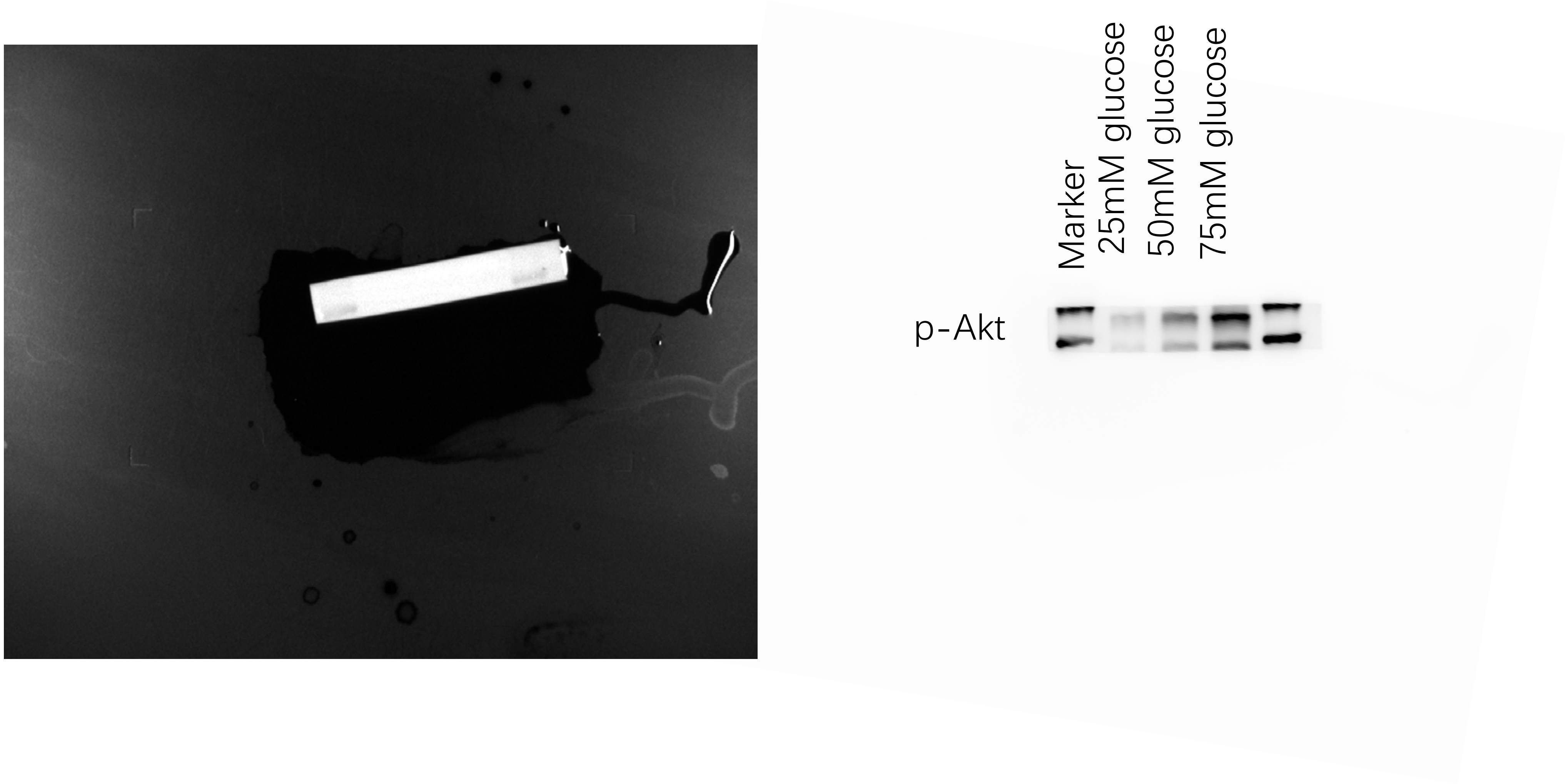

Fig.7b

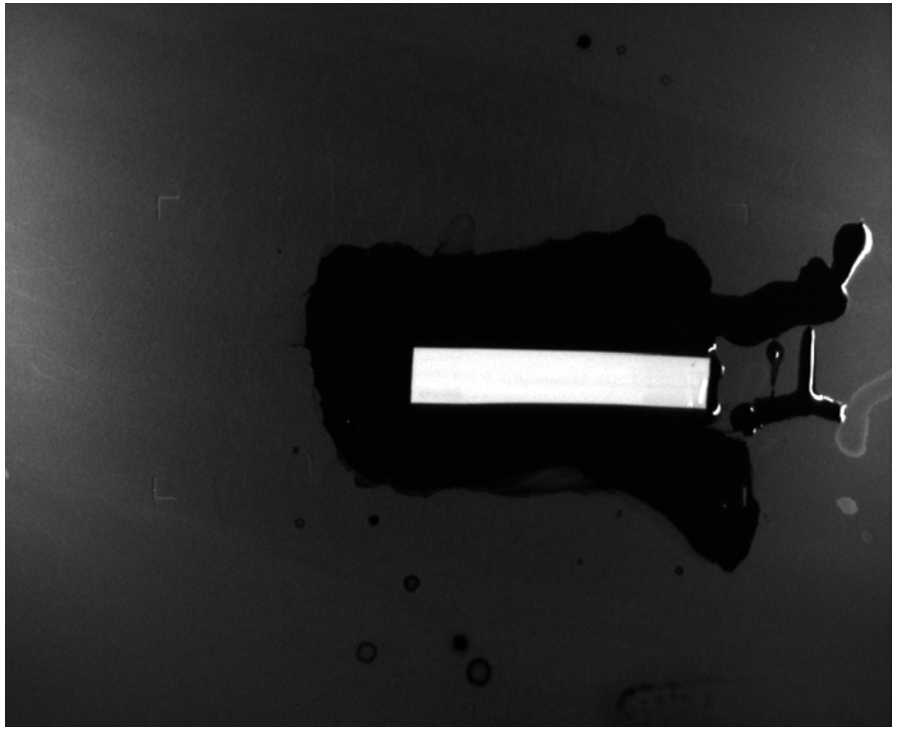

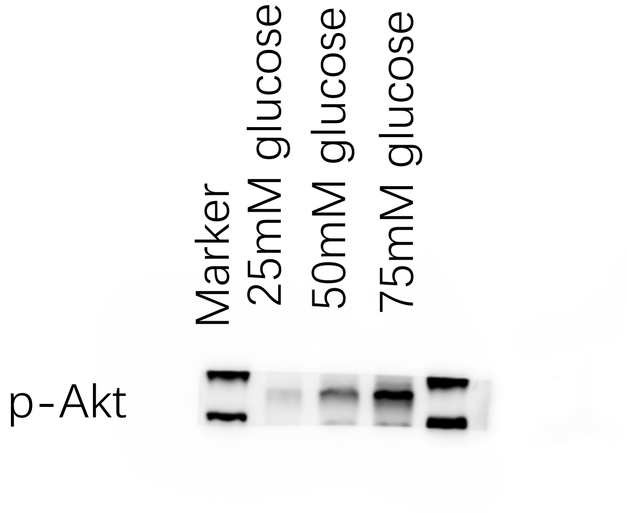

Fig.7d

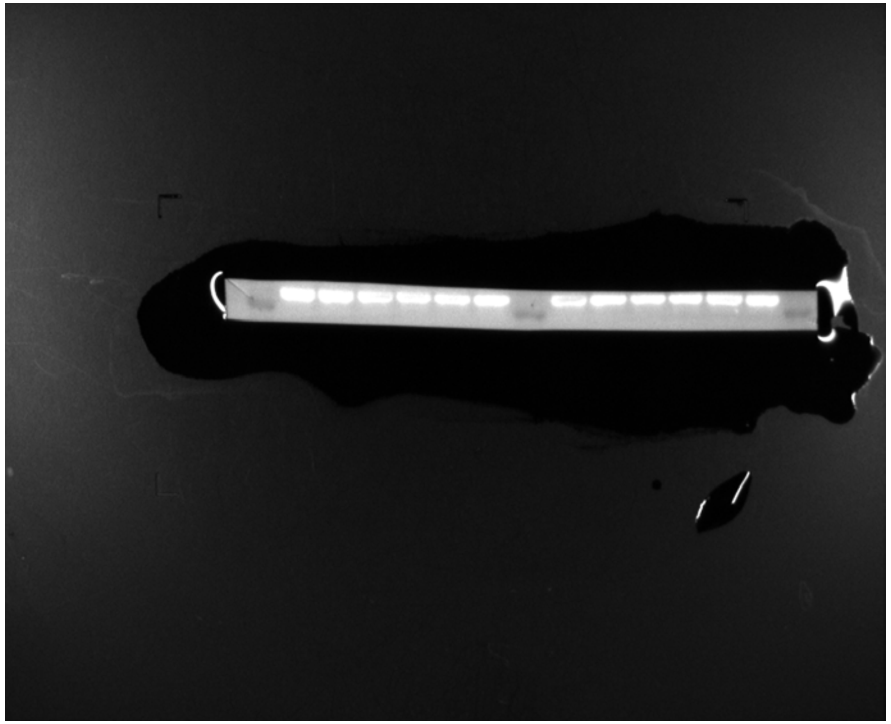

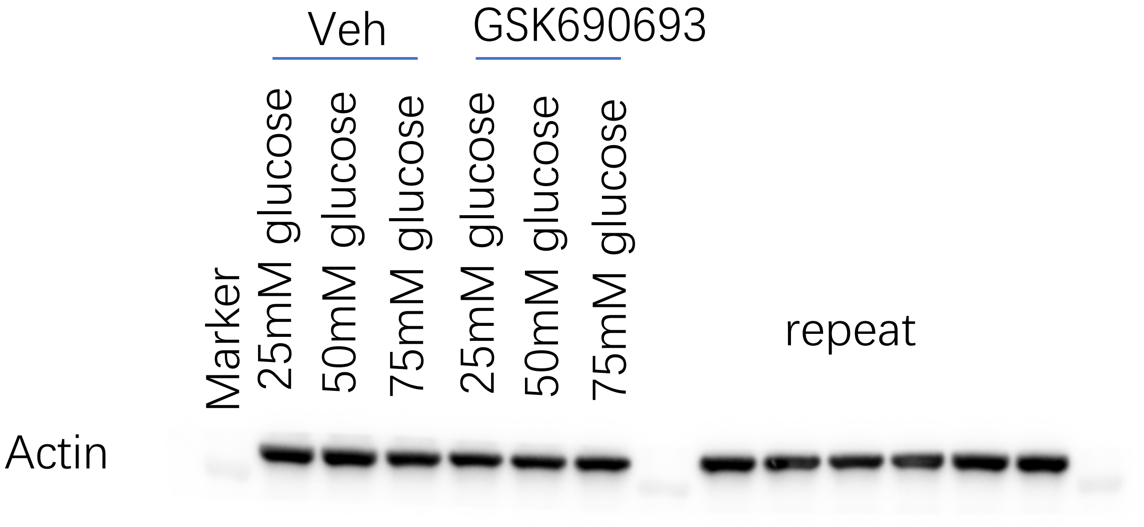

Veh
GSK690693
Marker
25mM glucose
50mM glucose
75mM glucose
25mM glucose
50mM glucose
75mM glucose
repeat
Actin

Fig.7d

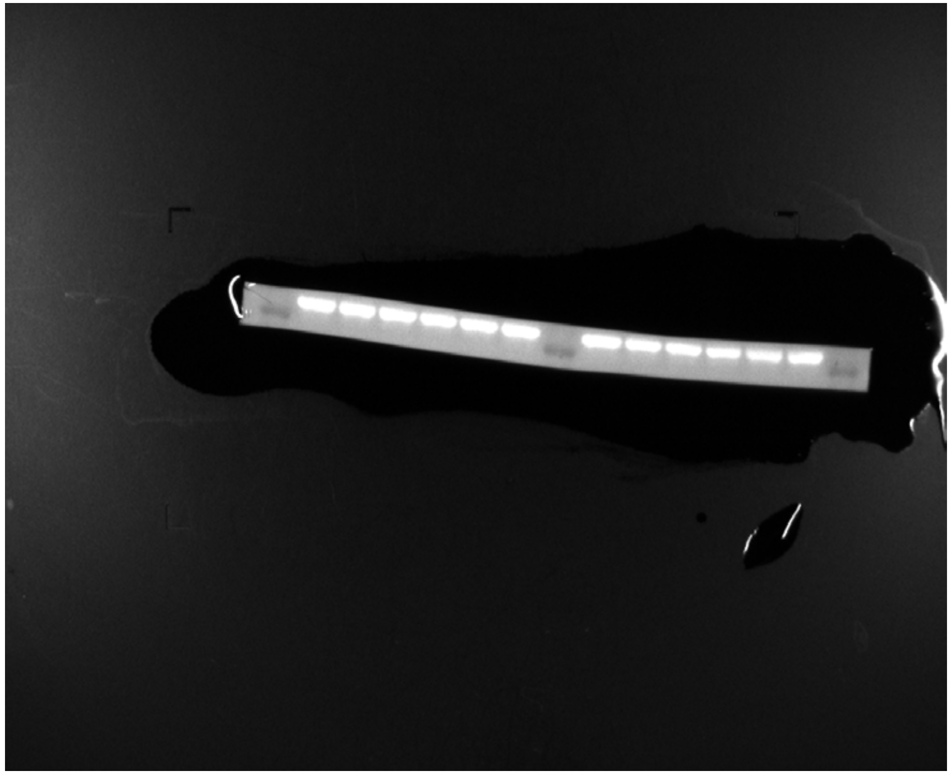

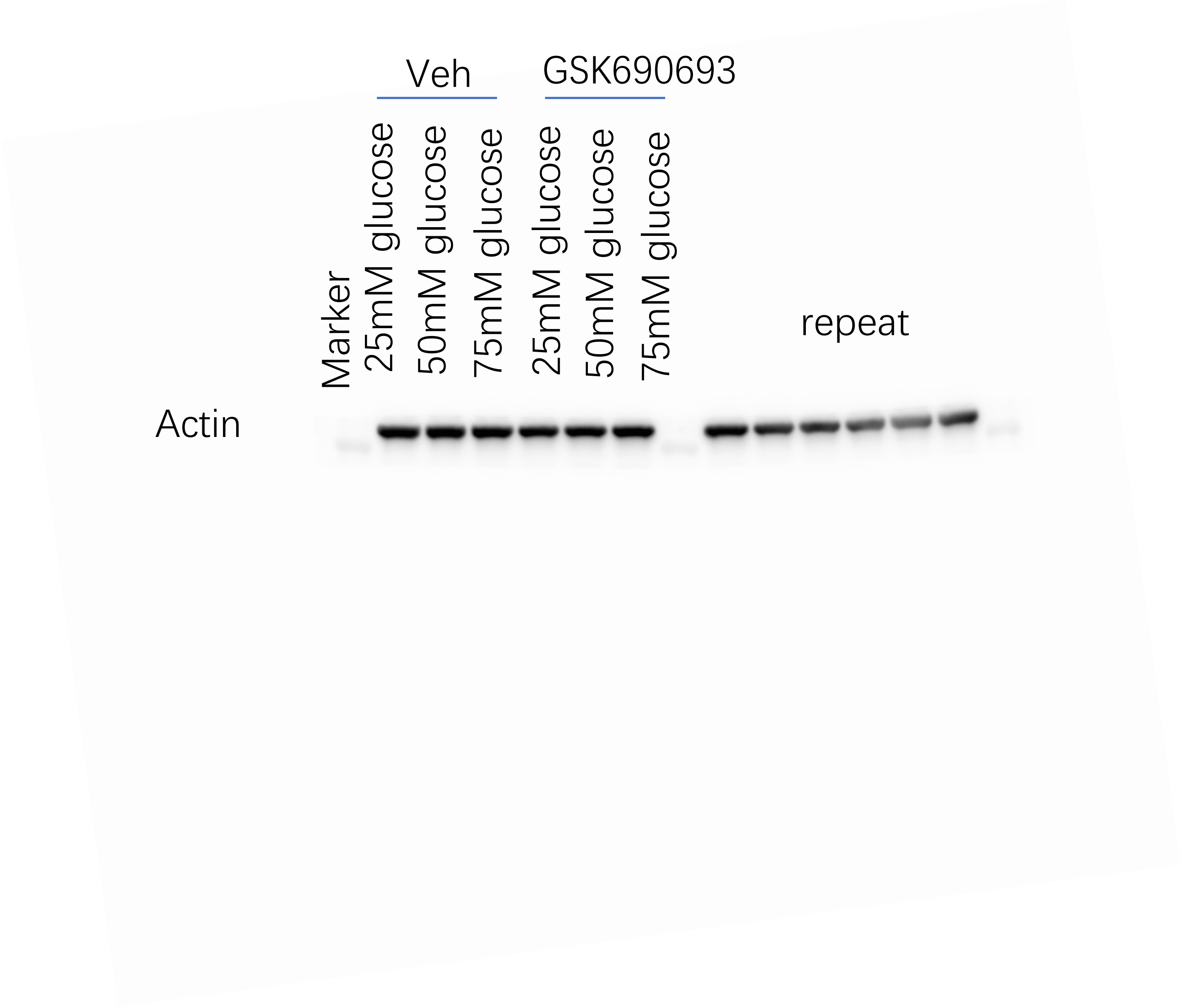
Veh
GSK690693
Marker
25mM glucose
50mM glucose
75mM glucose
25mM glucose
50mM glucose
75mM glucose
repeat
Actin

Fig.7d

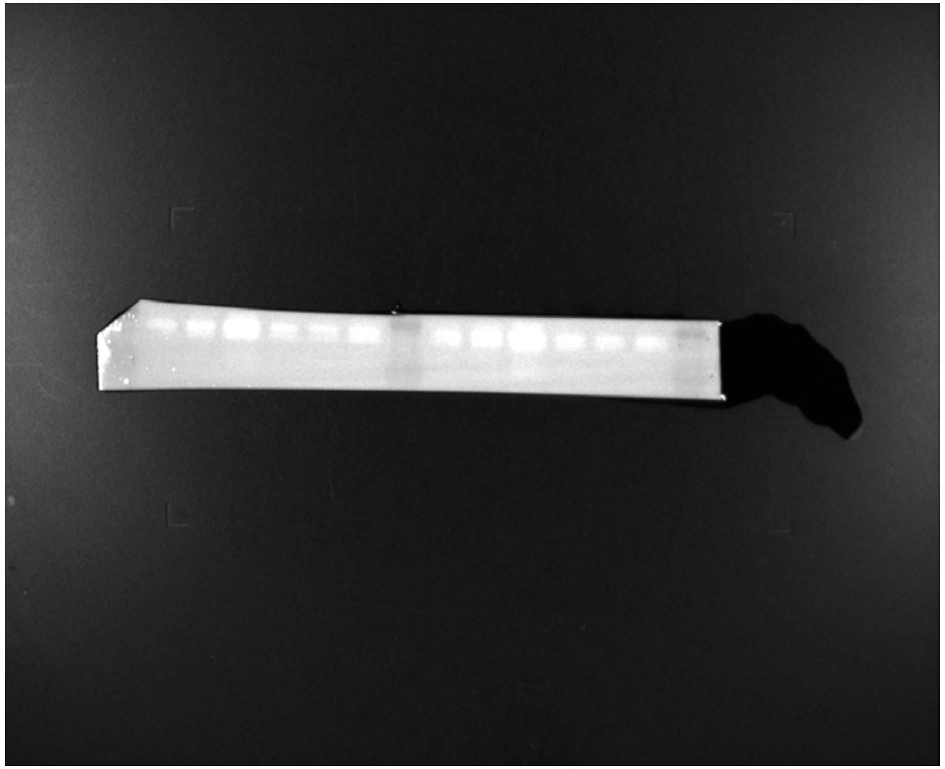

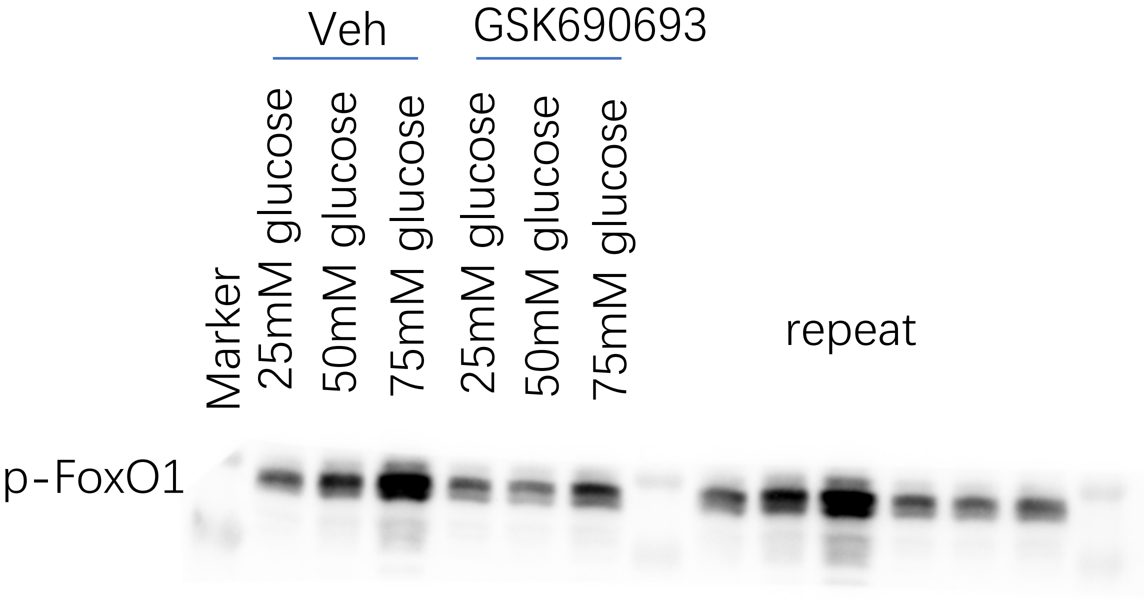
Veh
GSK690693
Marker
25mM glucose
50mM glucose
75mM glucose
25mM glucose
50mM glucose
75mM glucose
repeat
p-FoxO1

Fig.7d

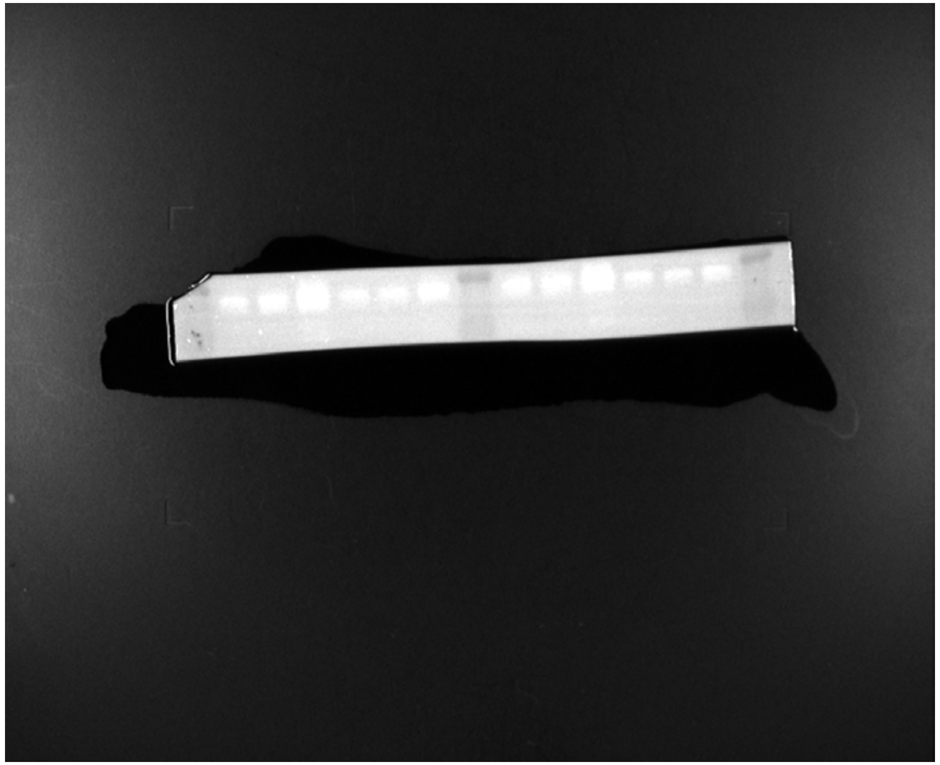

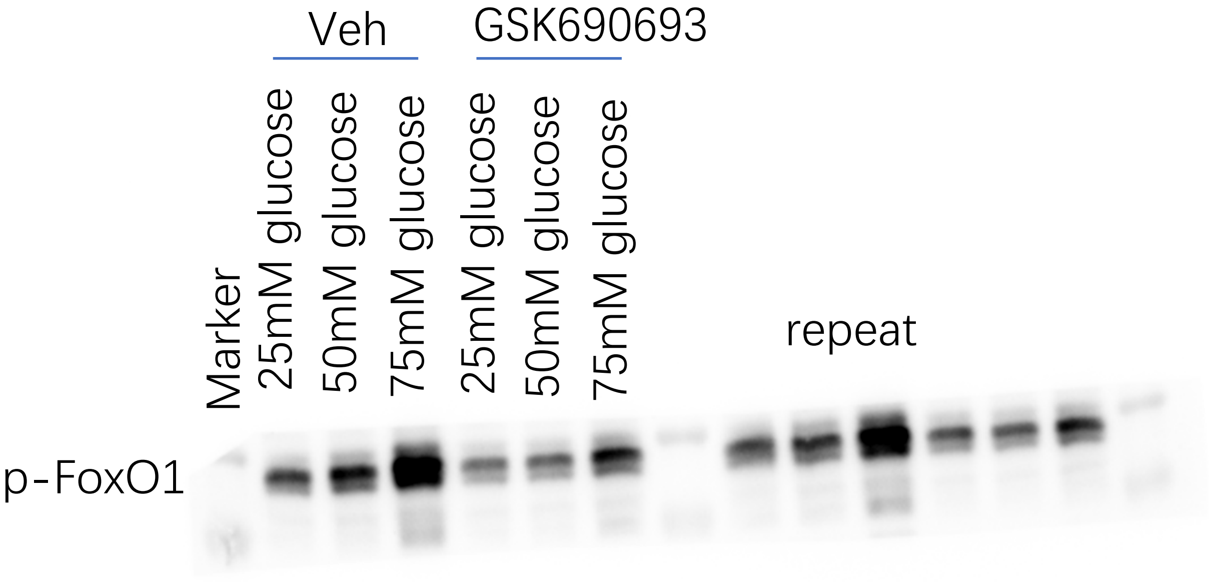

Veh
GSK690693
Marker
25mM glucose
50mM glucose
75mM glucose
25mM glucose
50mM glucose
75mM glucose
repeat
p-FoxO1

Fig.7d

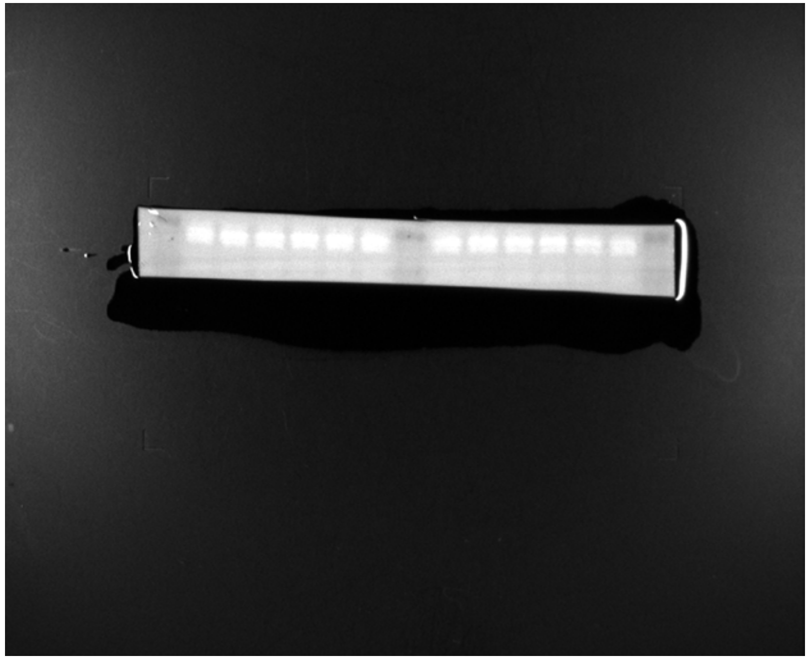

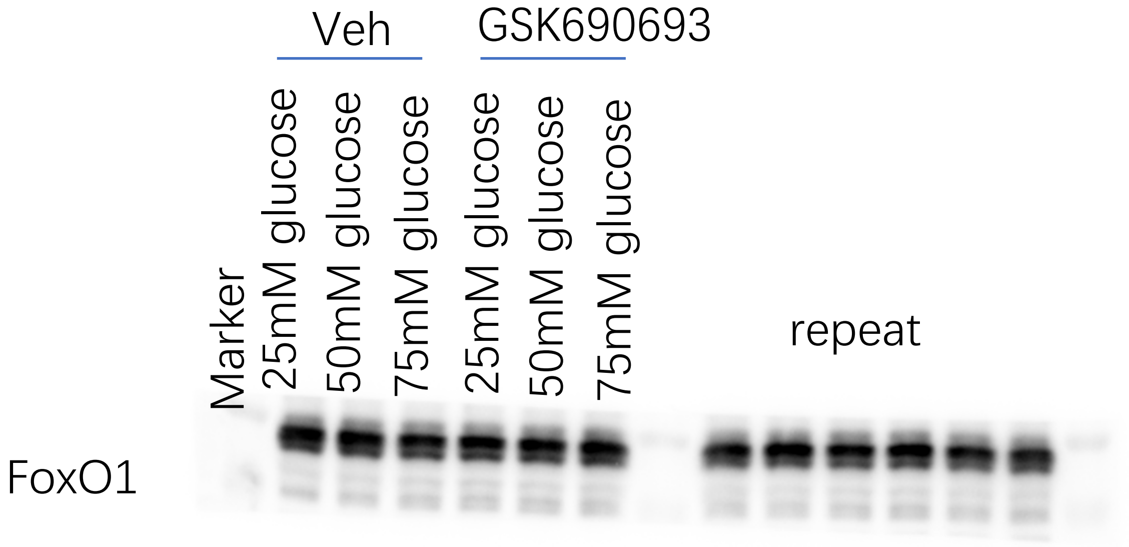
Veh
GSK690693
Marker
25mM glucose
50mM glucose
75mM glucose
25mM glucose
50mM glucose
75mM glucose
repeat
FoxO1

Fig.7d

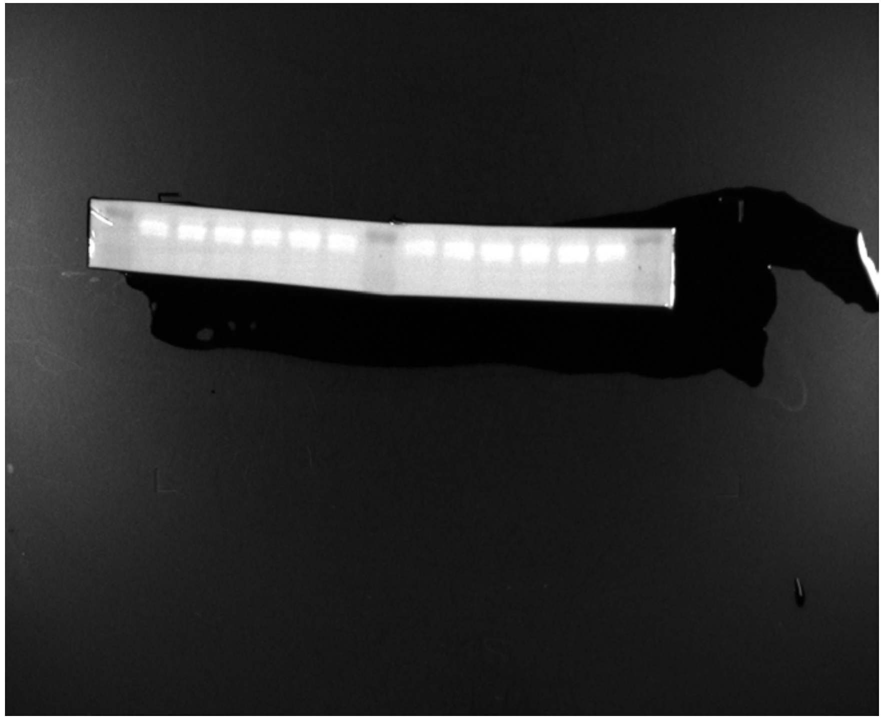

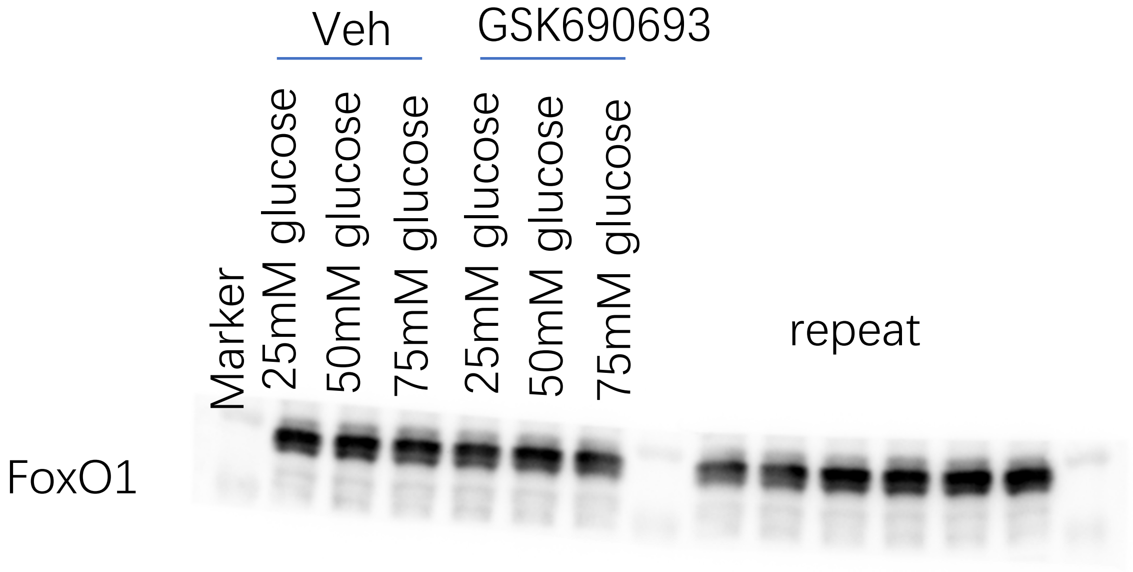
Veh
GSK690693
Marker
25mM glucose
50mM glucose
75mM glucose
25mM glucose
50mM glucose
75mM glucose
repeat
FoxO1

Fig.7d

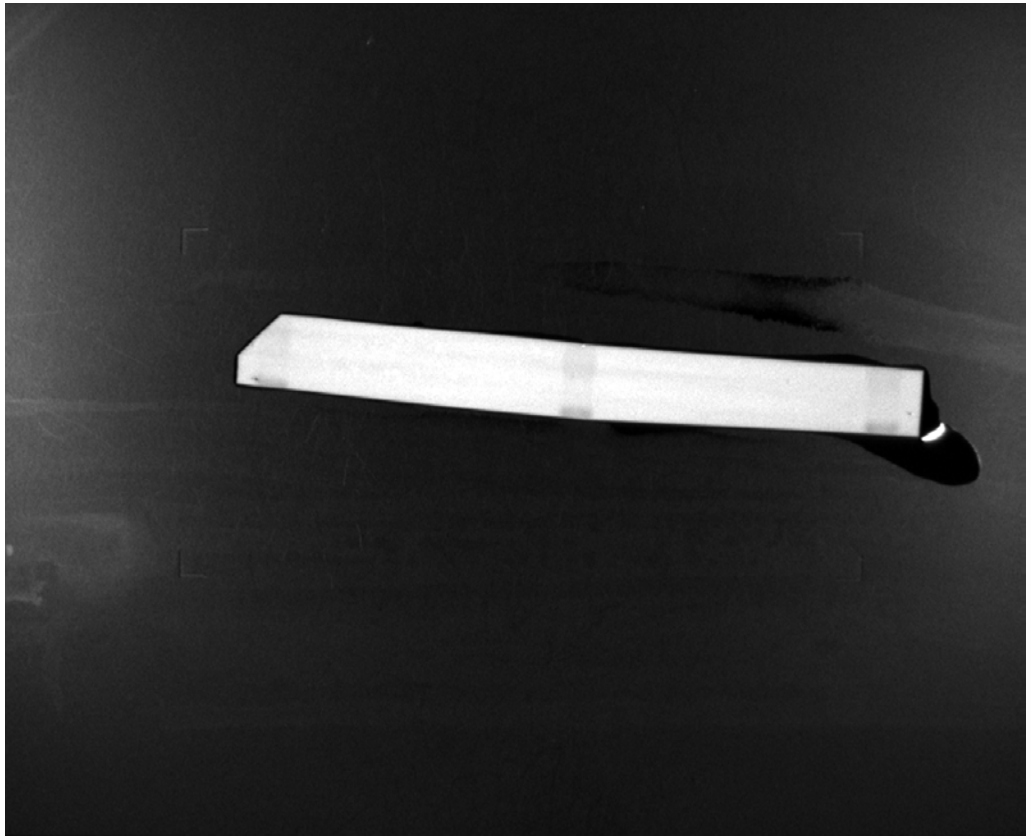

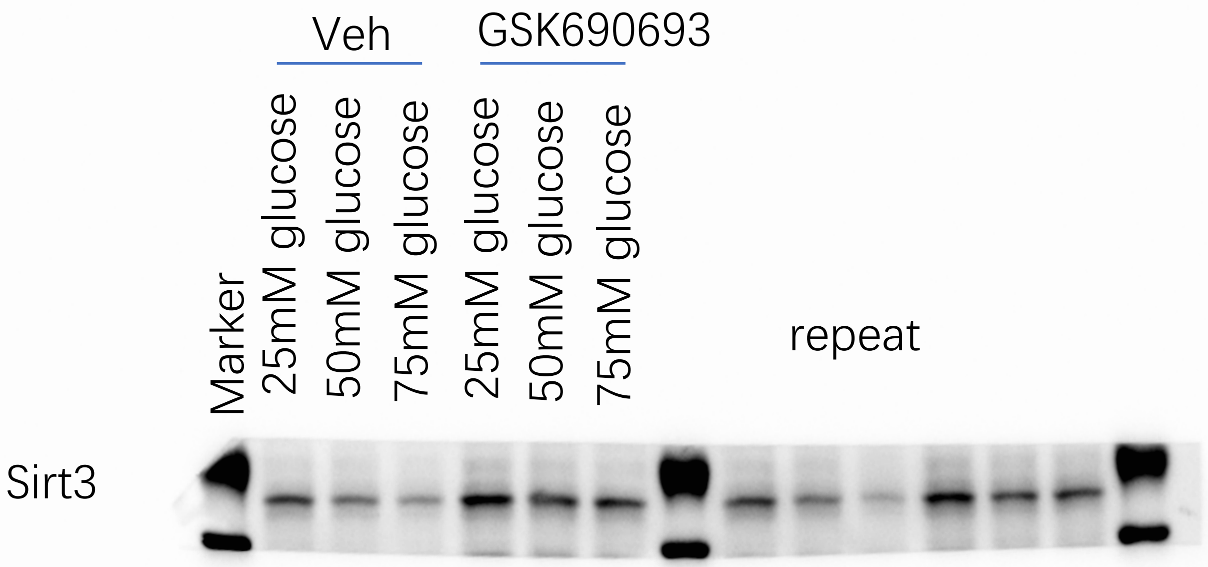
Veh
GSK690693
Marker
25mM glucose
50mM glucose
75mM glucose
25mM glucose
50mM glucose
75mM glucose
repeat
Sirt3

Fig.7d

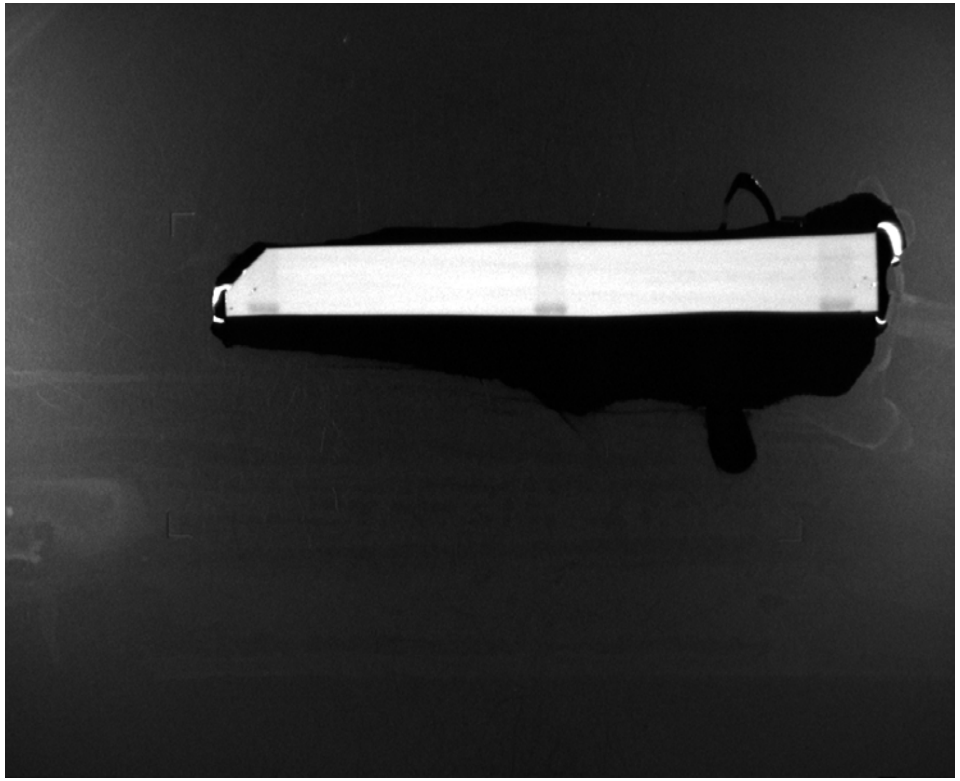

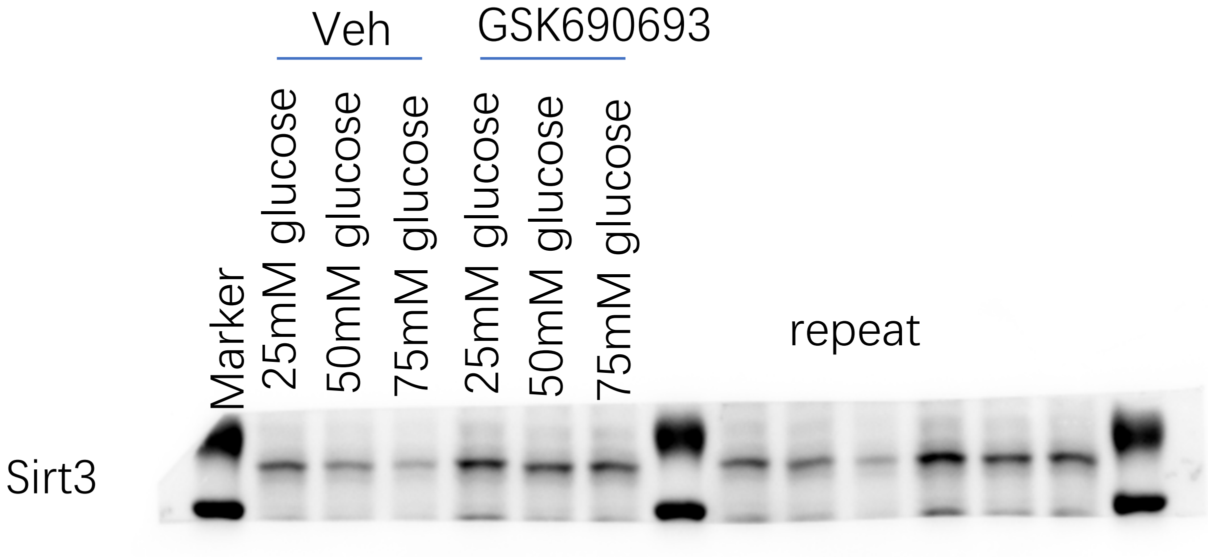
Veh
GSK690693
Marker
25mM glucose
50mM glucose
75mM glucose
25mM glucose
50mM glucose
75mM glucose
repeat
Sirt3

Fig.7f

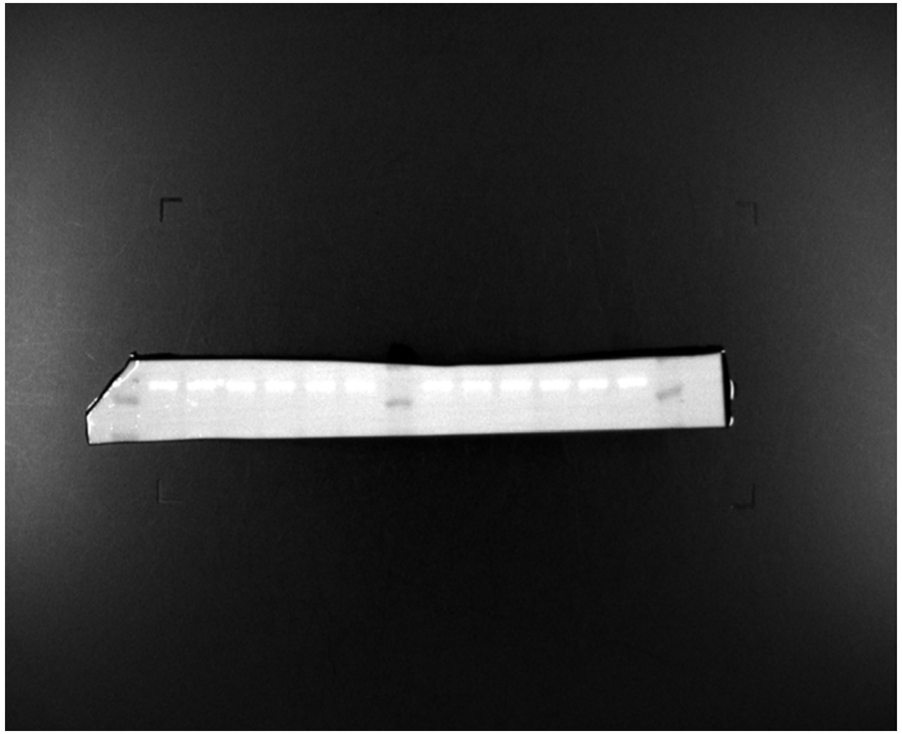

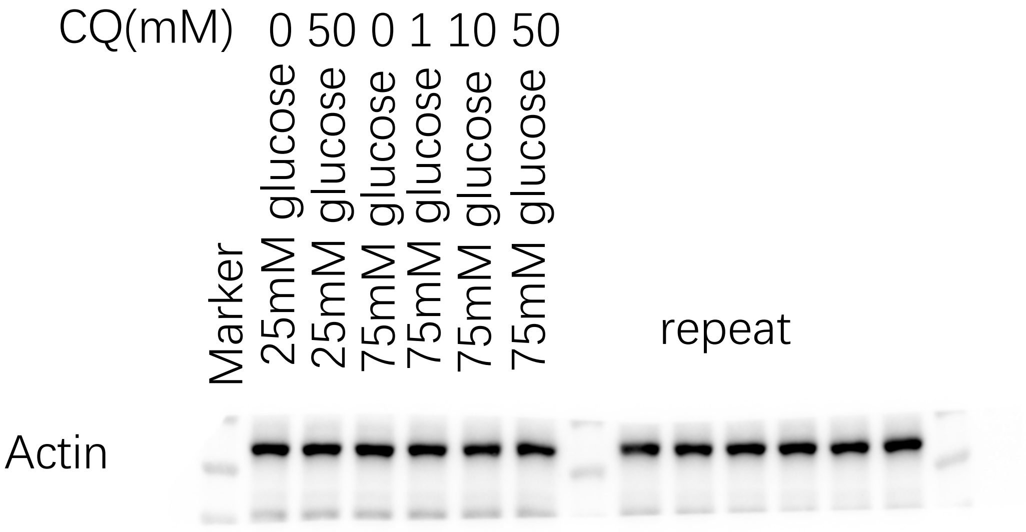
CQ(mM) 0 50 0 1 10 50
Marker
25mM glucose
25mM glucose
75mM glucose
75mM glucose
75mM glucose
75mM glucose
repeat
Actin

Fig.7f

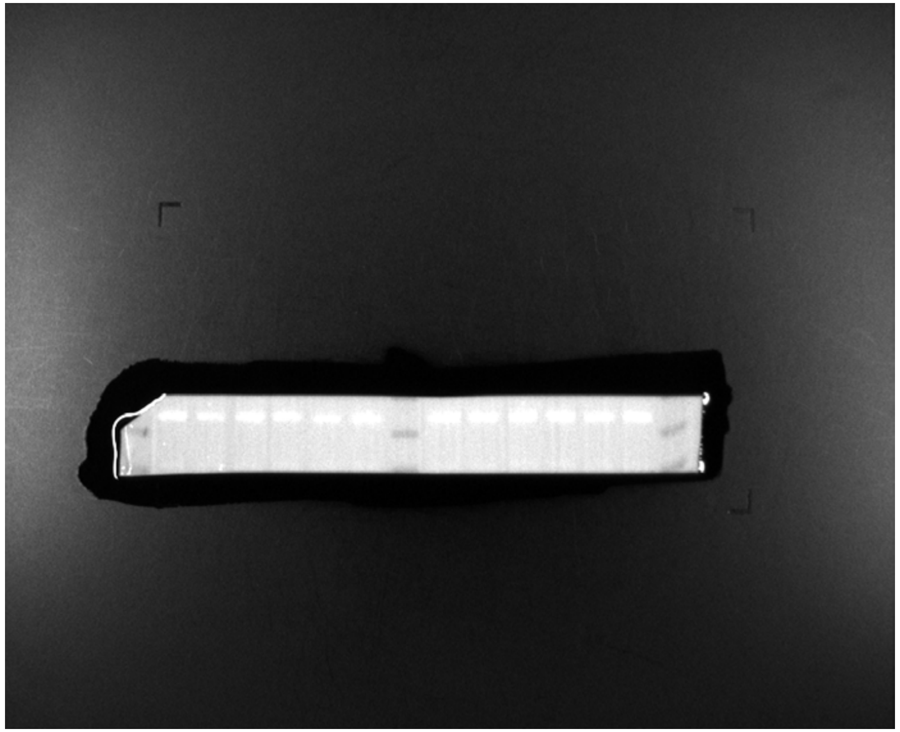

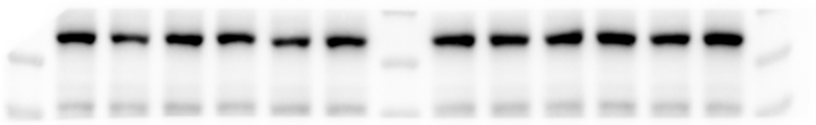
CQ(mM) 0 50 0 1 10 50
Marker
25mM glucose
25mM glucose
75mM glucose
75mM glucose
75mM glucose
75mM glucose
repeat
Actin

Fig.7f

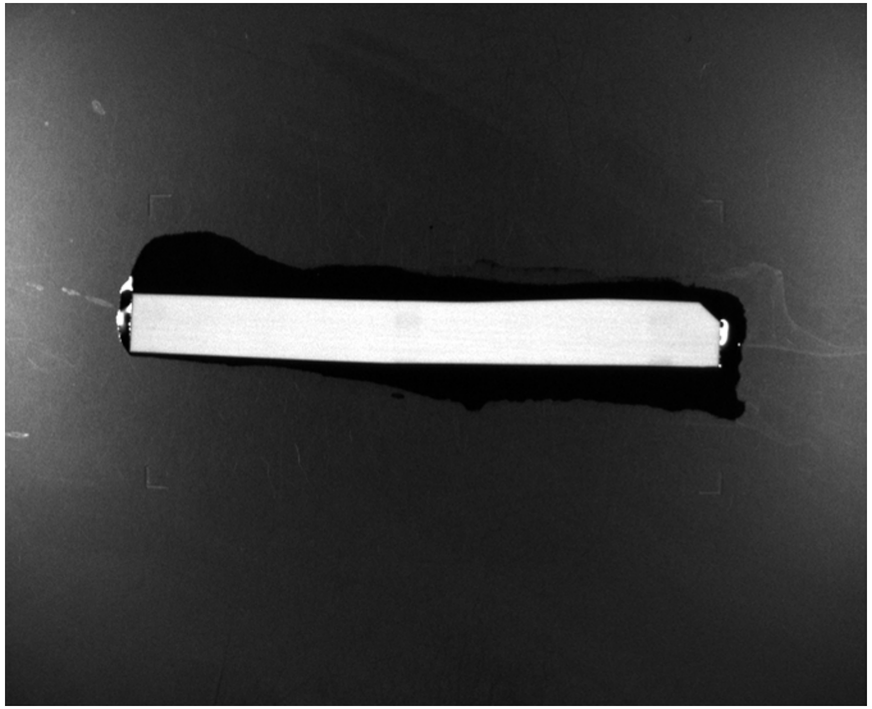

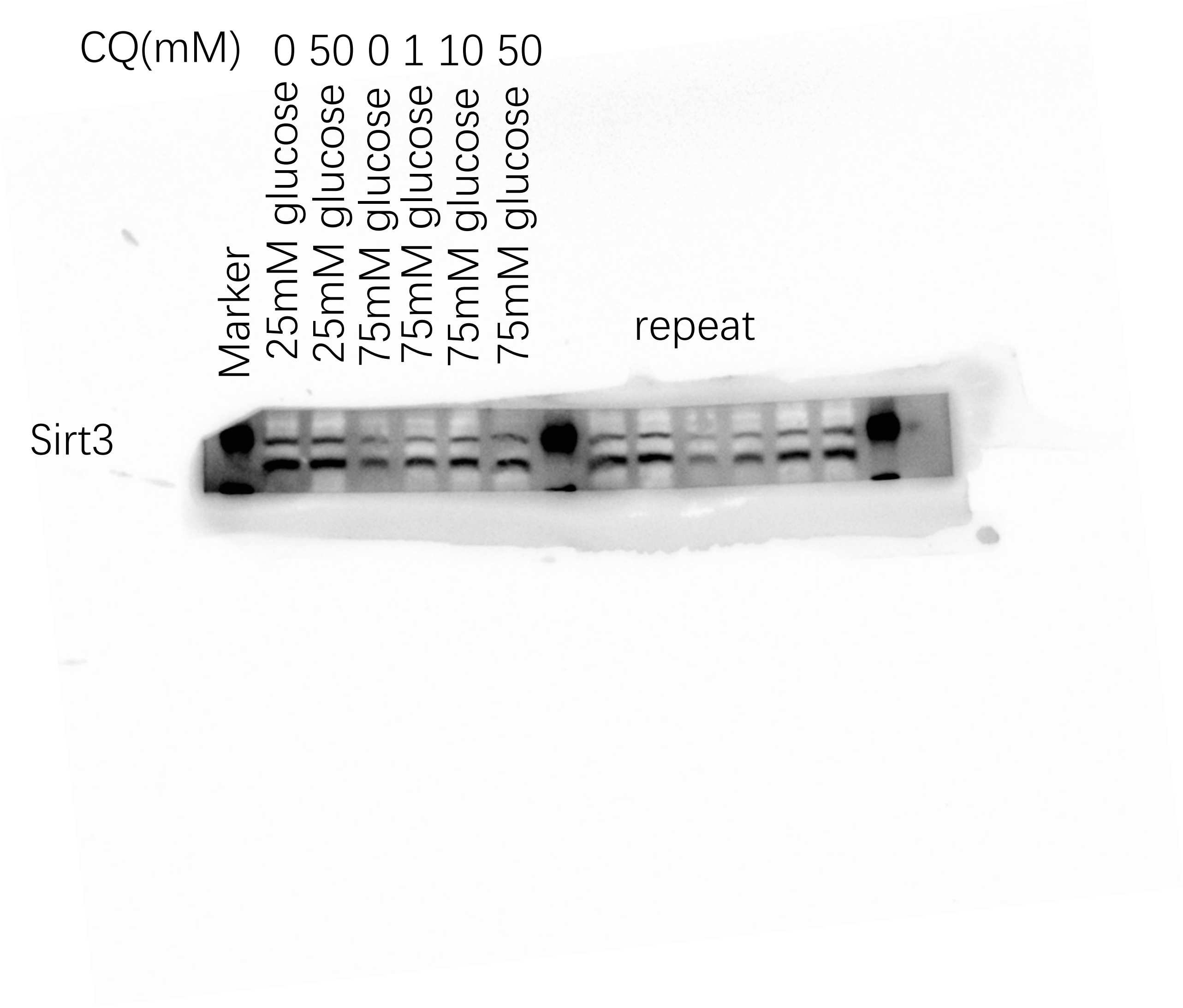
CQ(mM)
0 50 0 1 10 50
Marker
25mM glucose
25mM glucose
75mM glucose
75mM glucose
75mM glucose
75mM glucose
repeat
Sirt3

Fig.7f

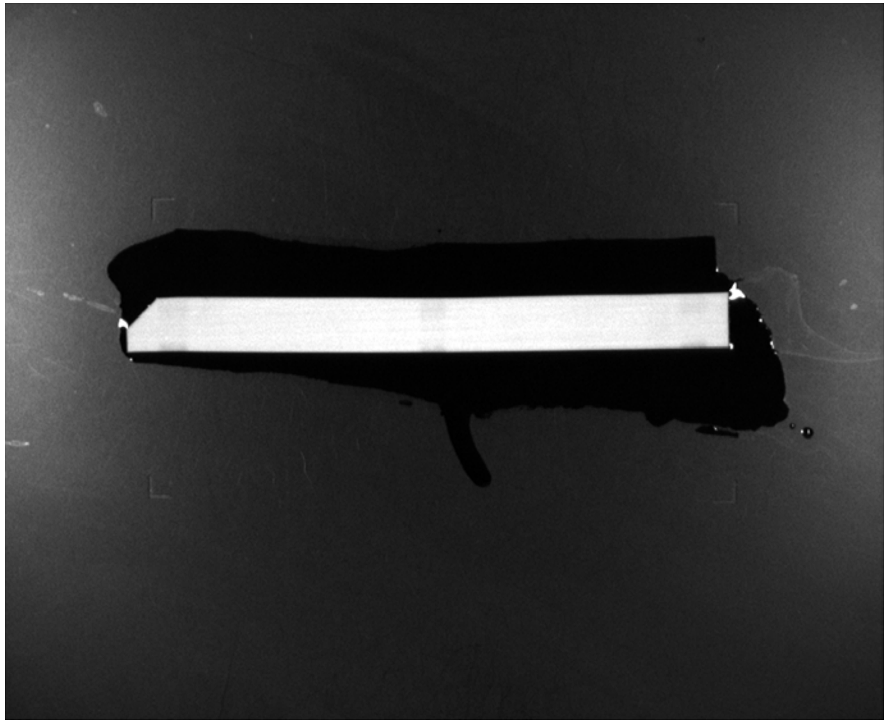

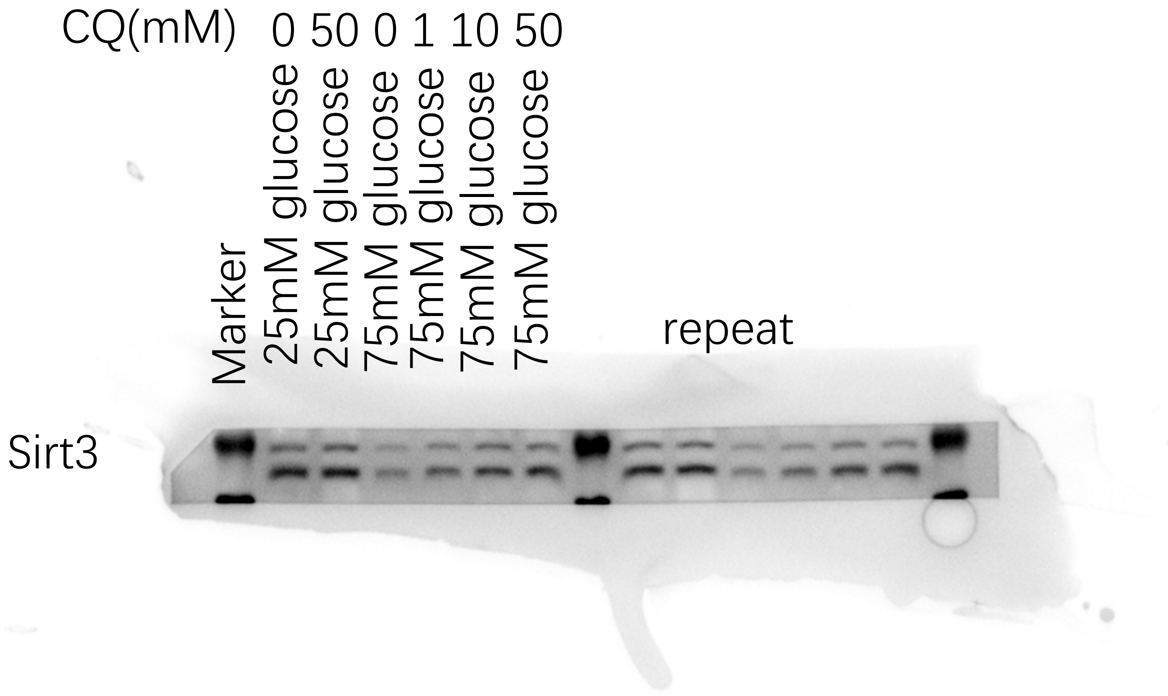
CQ(mM) 0 50 0 1 10 50
Marker
25mM glucose
25mM glucose
75mM glucose
75mM glucose
75mM glucose
75mM glucose
repeat
Sirt3

Fig.7g

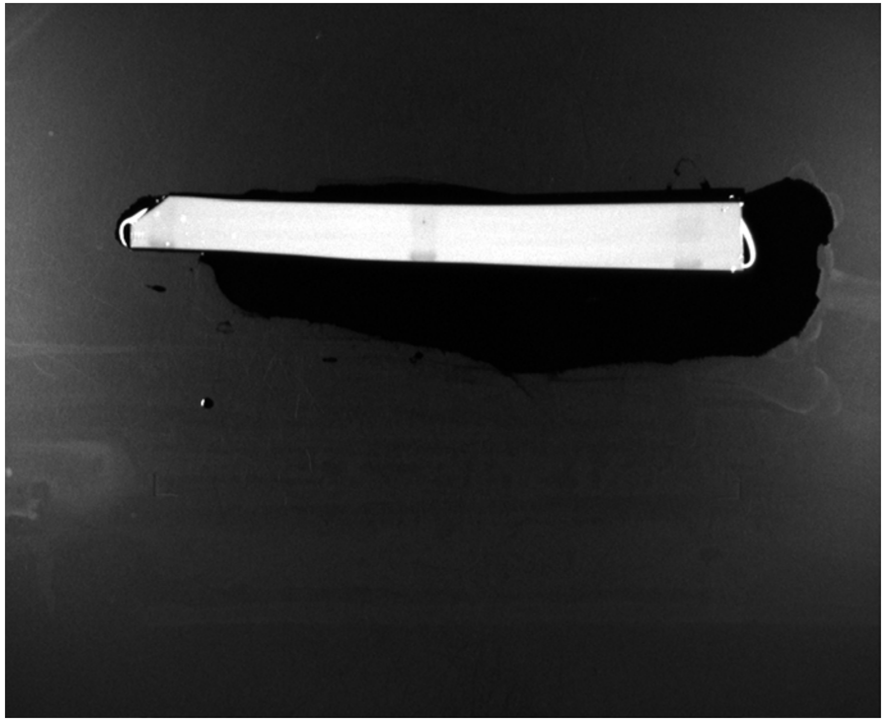

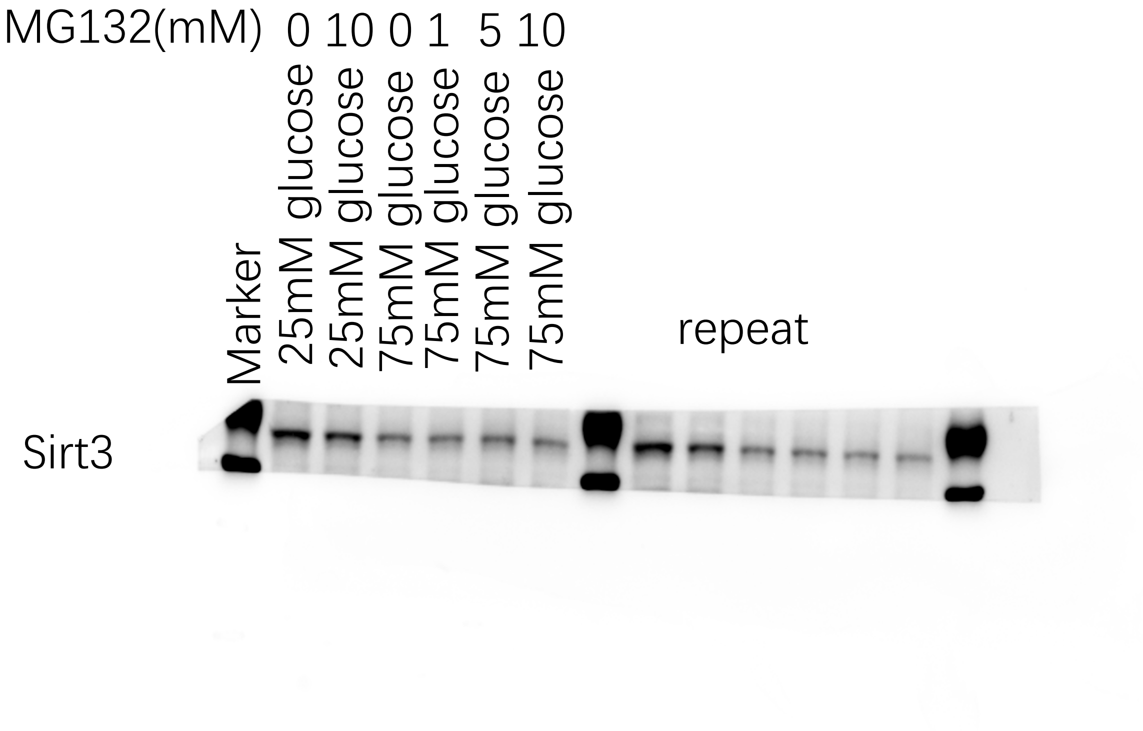
MG132(mM) 0 10 0 1 5 10
Marker
25mM glucose
25mM glucose
75mM glucose
75mM glucose
75mM glucose
75mM glucose
repeat
Sirt3

Fig.7g

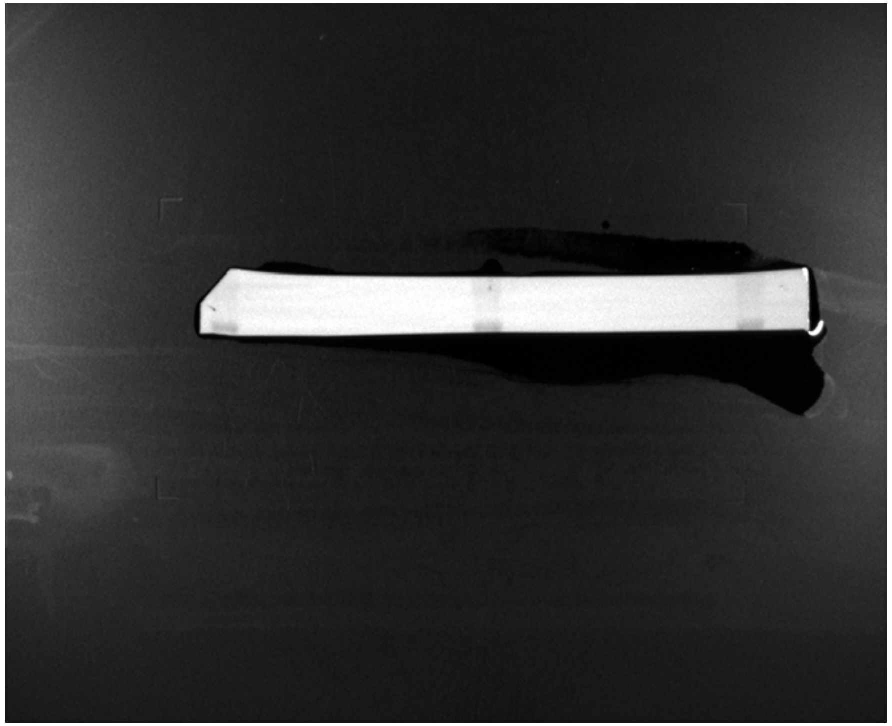

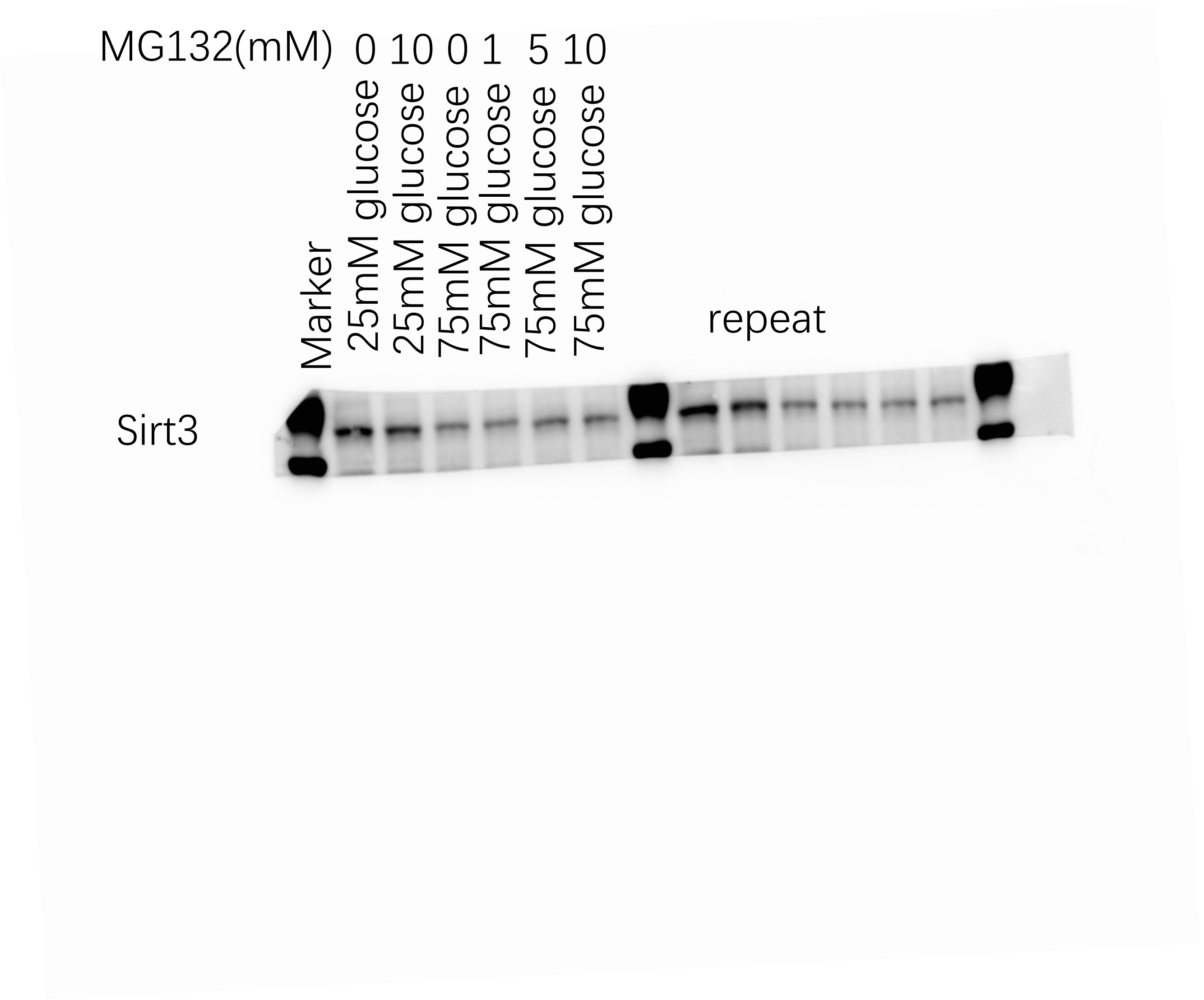
MG132(mM) 0 10 0 1 5 10
Marker
25mM glucose
25mM glucose
75mM glucose
75mM glucose
75mM glucose
75mM glucose
repeat
Sirt3

Fig.7g

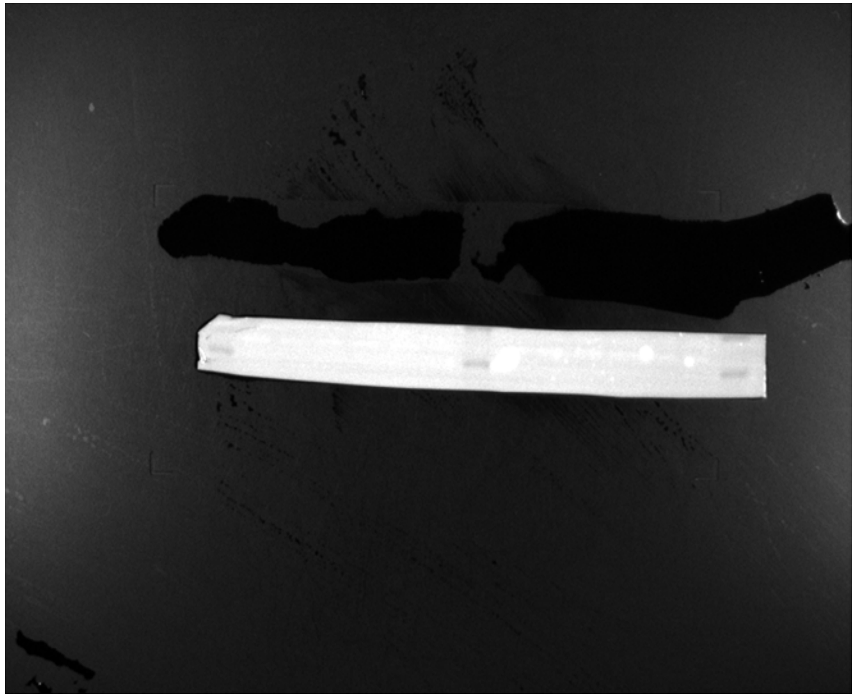

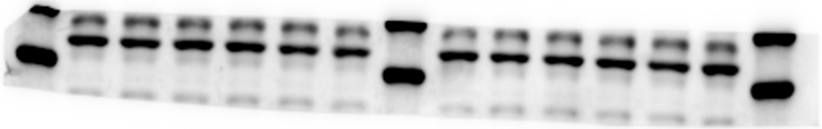
MG132(mM) 0 10 0 1 5 10
Marker
25mM glucose
25mM glucose
75mM glucose
75mM glucose
75mM glucose
75mM glucose
repeat
Actin

Fig.7g

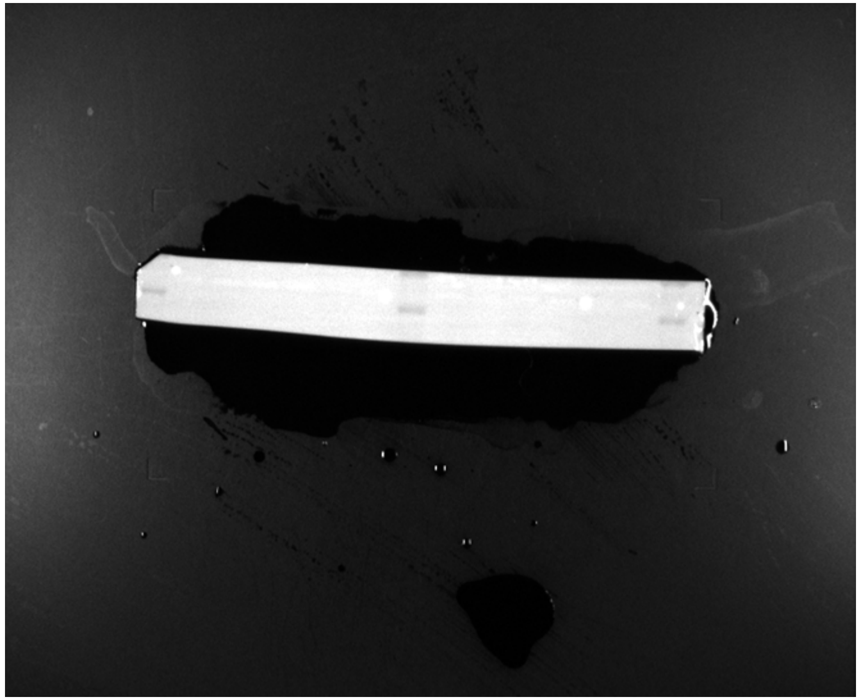

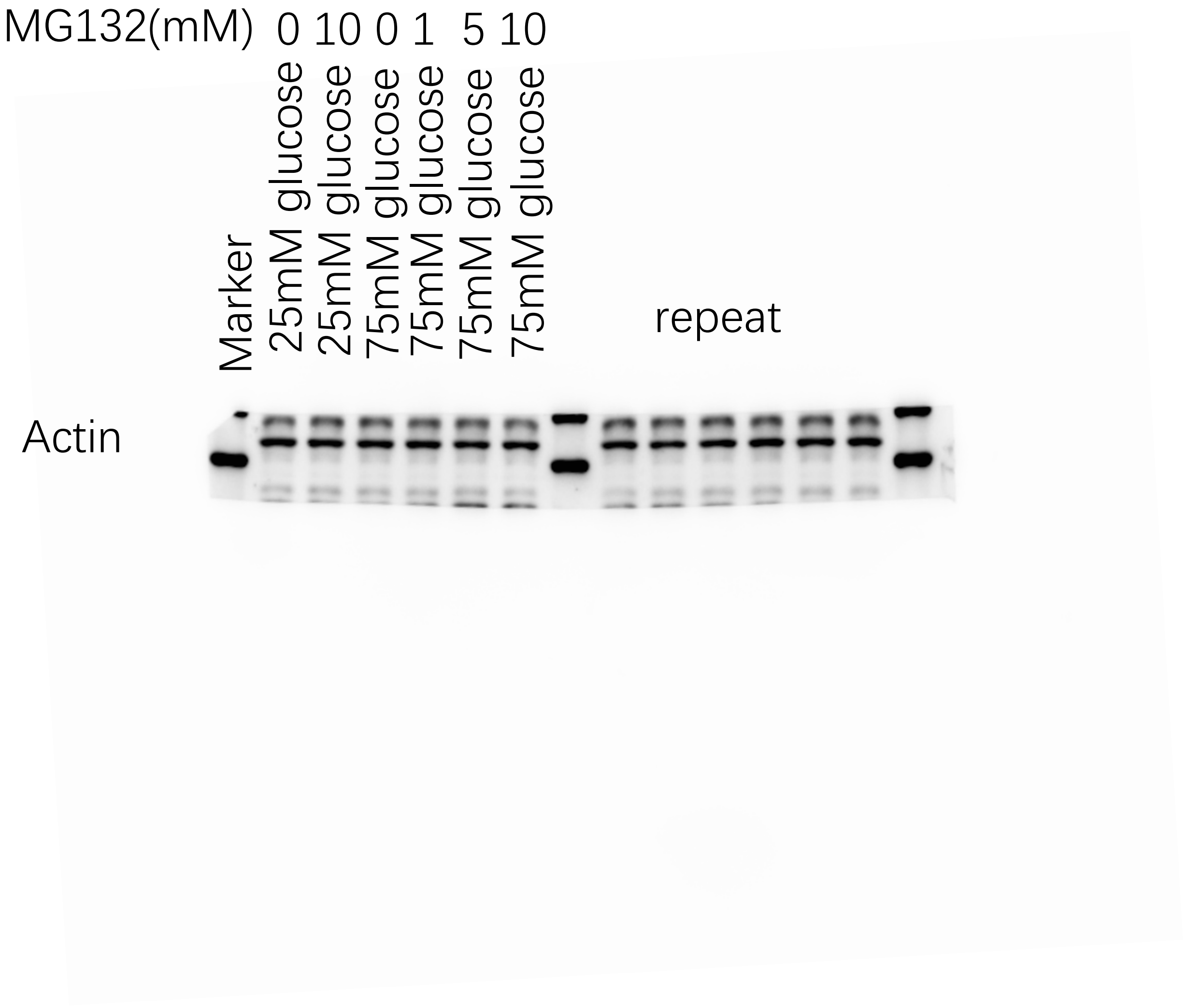

MG132(mM) 0 10 0 1 5 10
Marker
25mM glucose
25mM glucose
75mM glucose
75mM glucose
75mM glucose
75mM glucose
repeat
Actin

Fig.7h

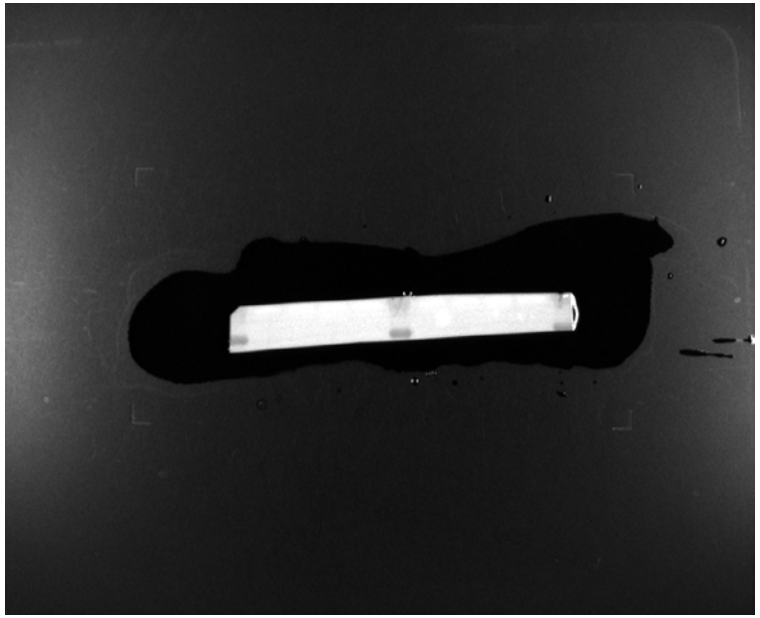

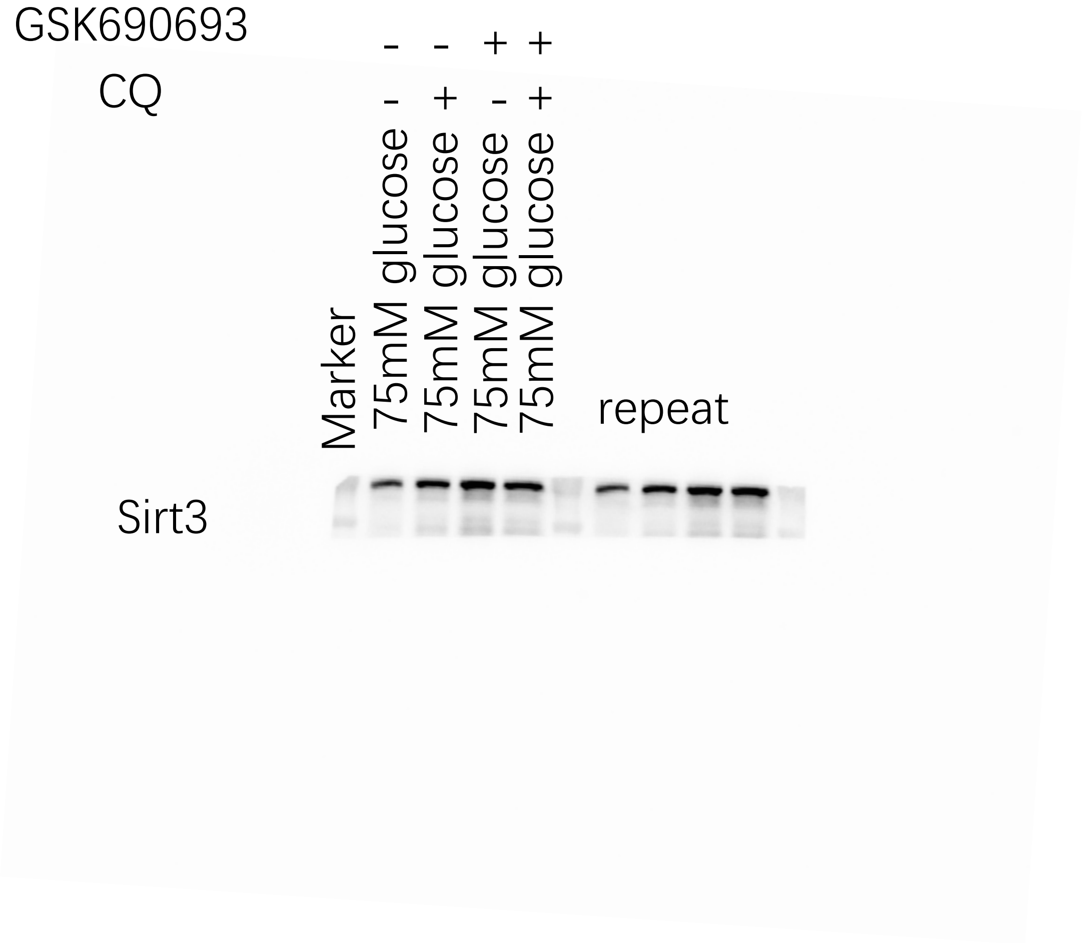
GSK690693
CQ
- - + +
- + - +
Marker
75mM glucose
75mM glucose
75mM glucose
75mM glucose
repeat
Sirt3

Fig.7h

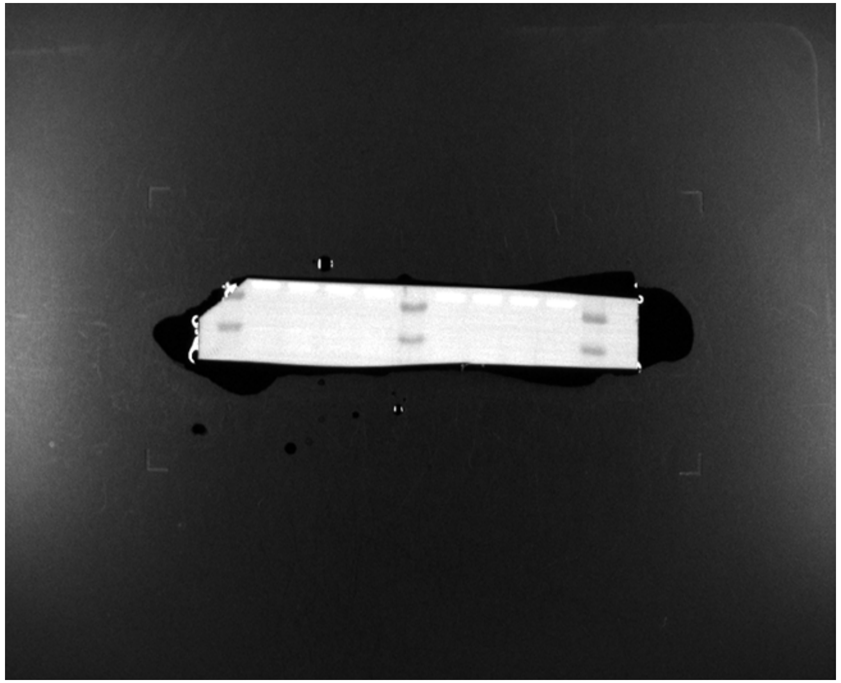

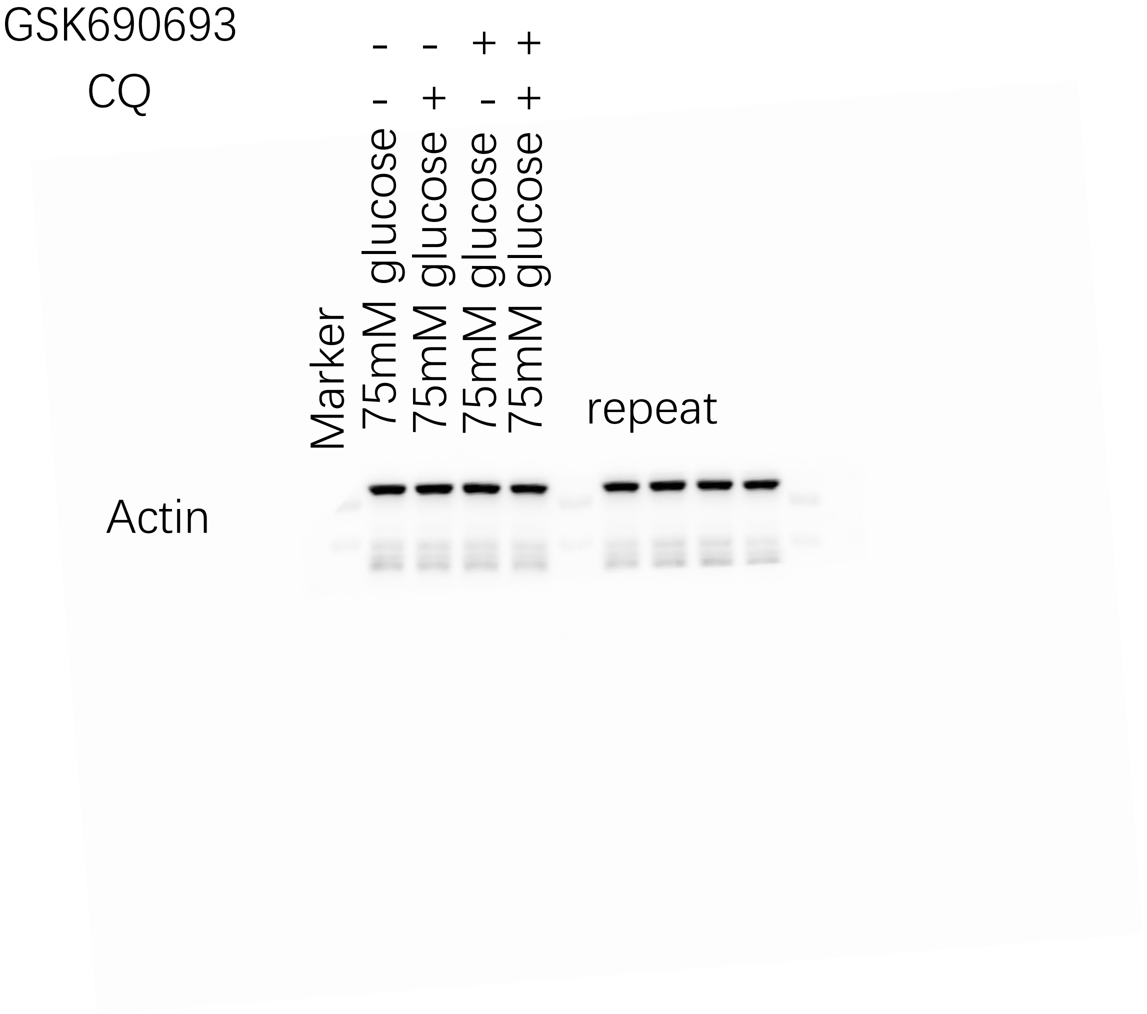
GSK690693
CQ
- - + +
- + - +
Marker
75mM glucose
75mM glucose
75mM glucose
75mM glucose
repeat
Actin

Fig.7j

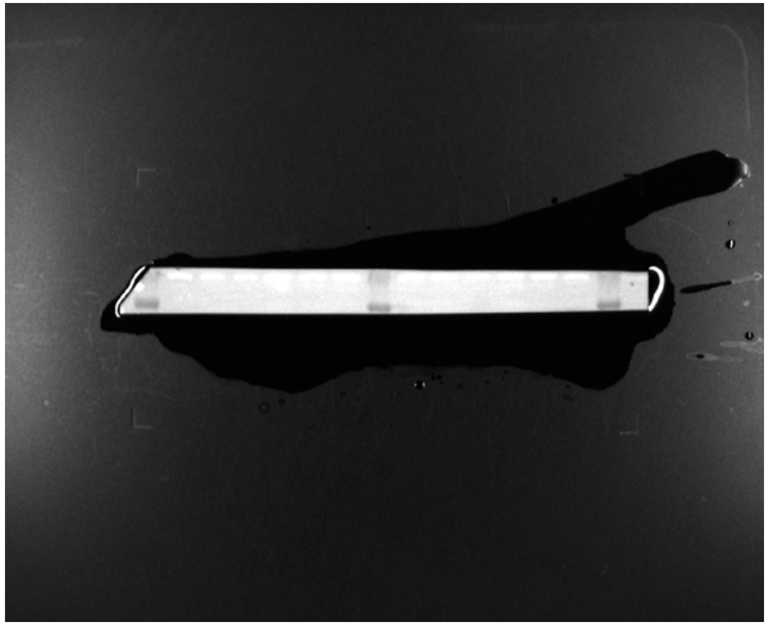

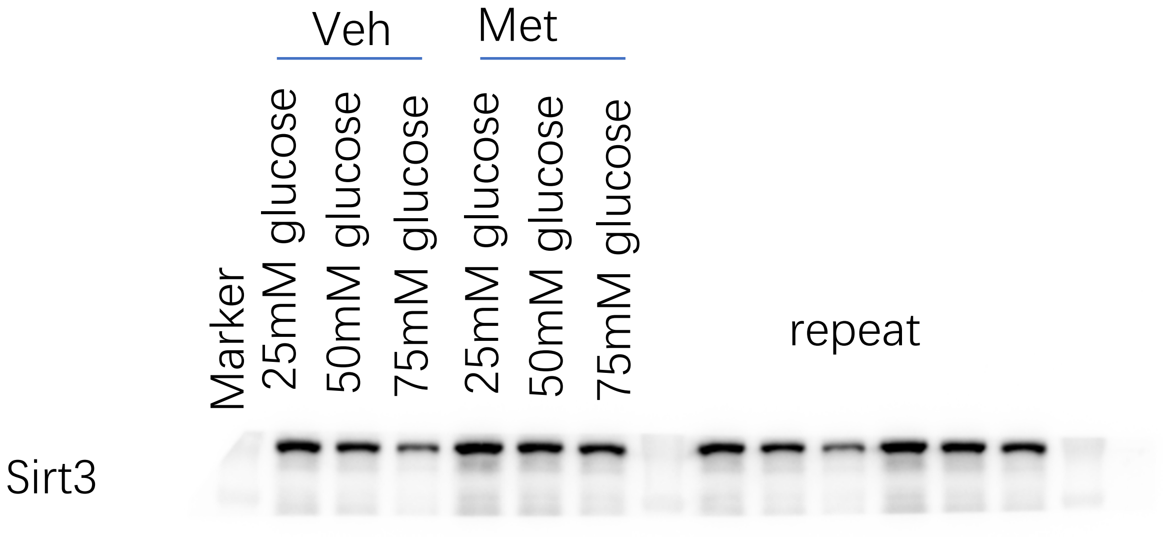
Veh
Met
Marker
25mM glucose
50mM glucose
75mM glucose
25mM glucose
50mM glucose
75mM glucose
repeat
Sirt3

Fig.7j

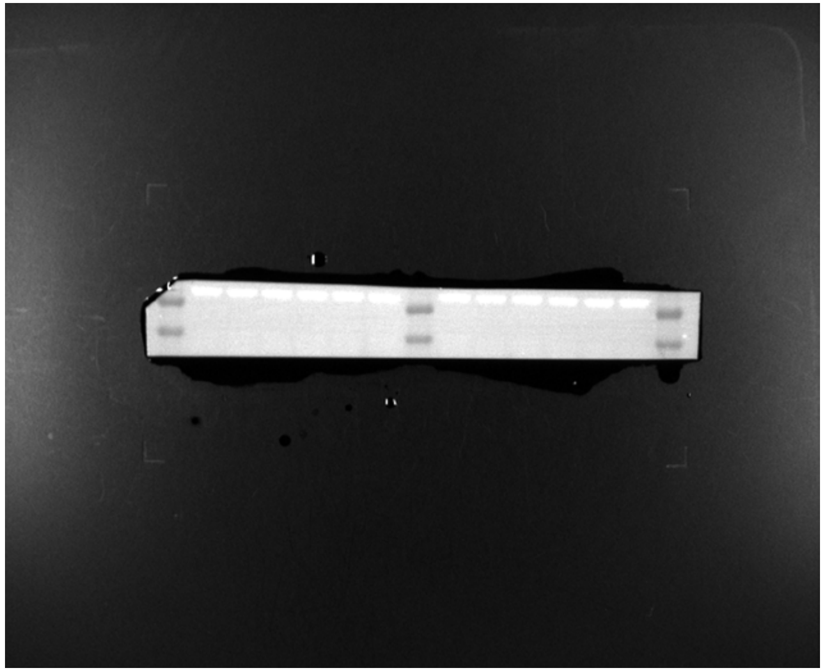

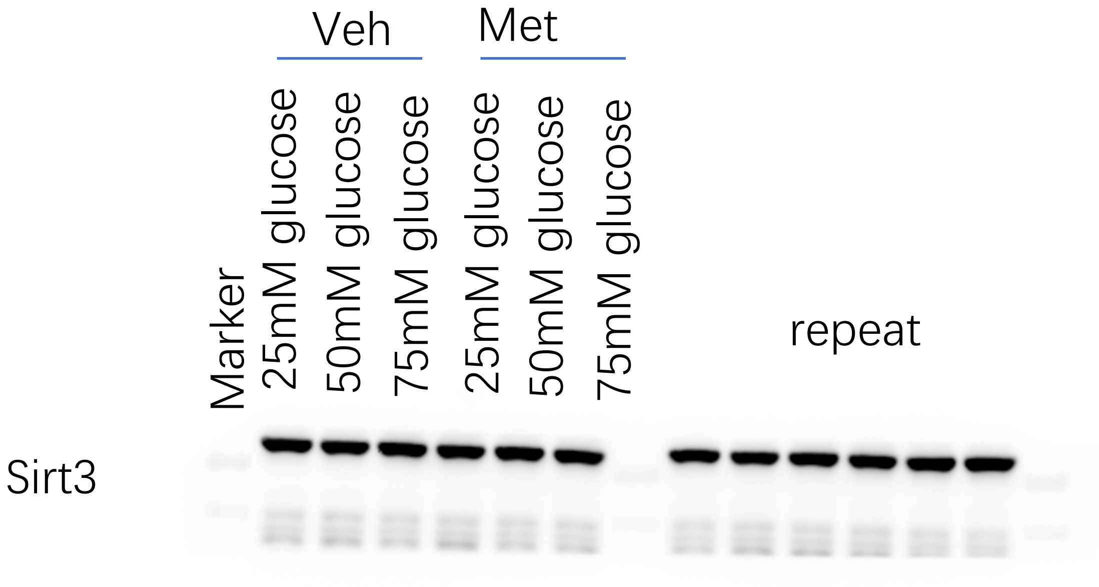
Veh
Met
Marker
25mM glucose
50mM glucose
75mM glucose
25mM glucose
50mM glucose
75mM glucose
repeat
Sirt3

Fig.8f

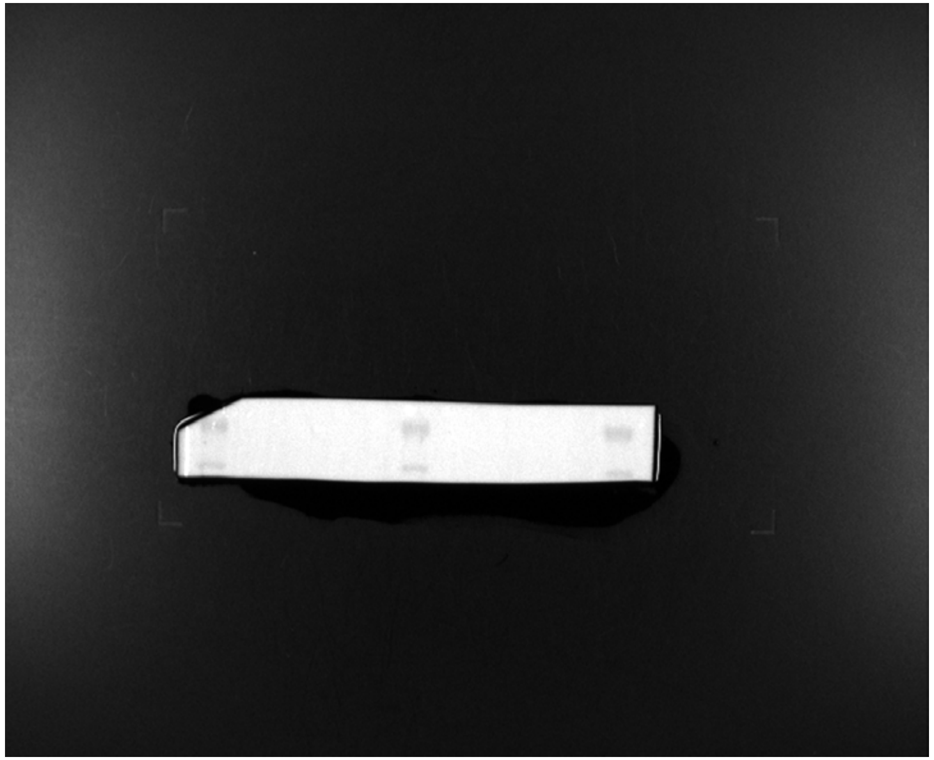

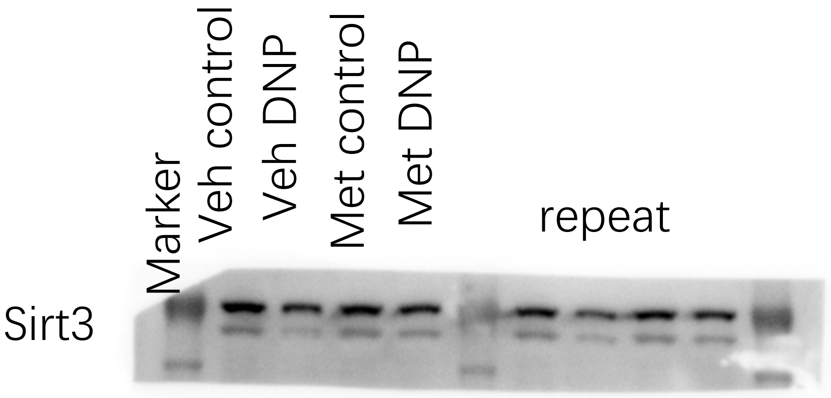

Fig.8f

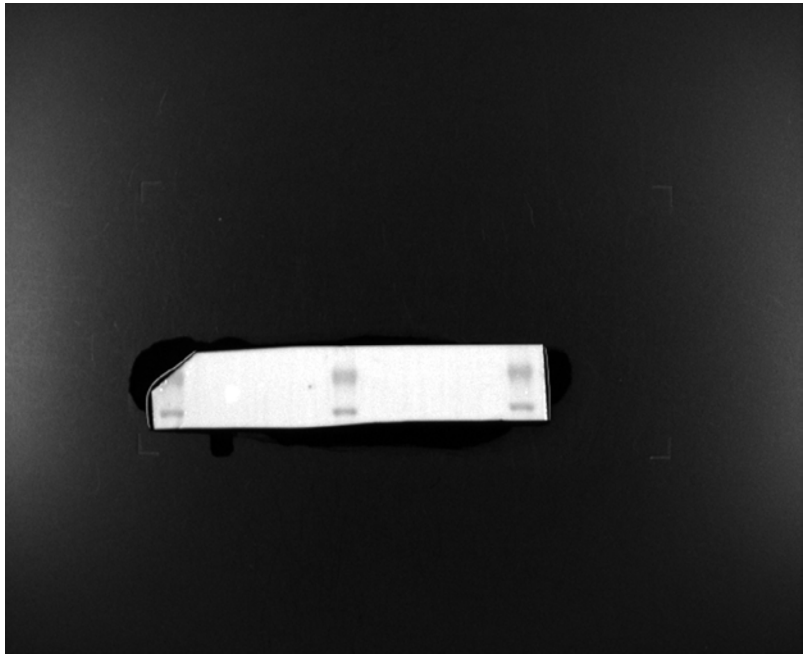

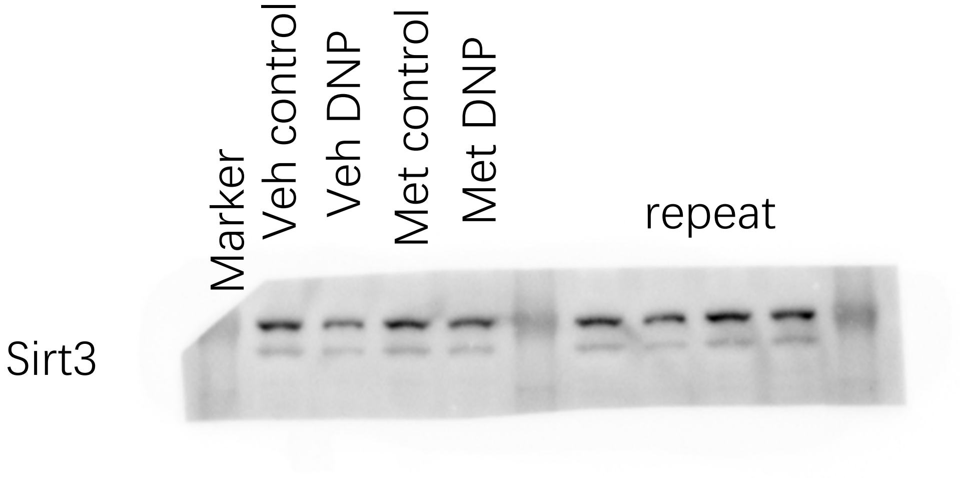

Fig.8f

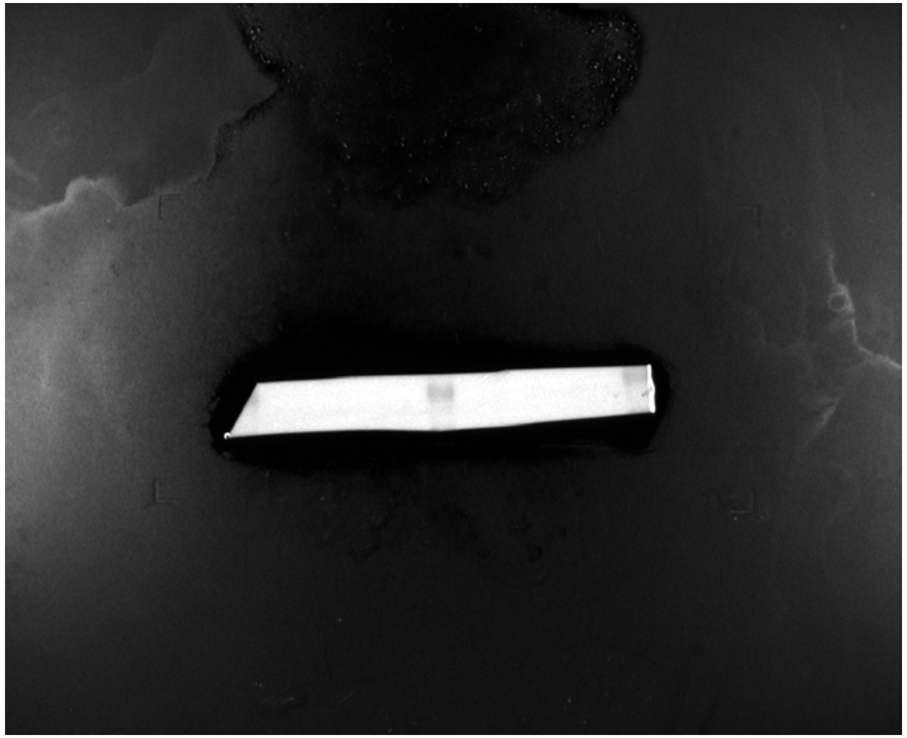

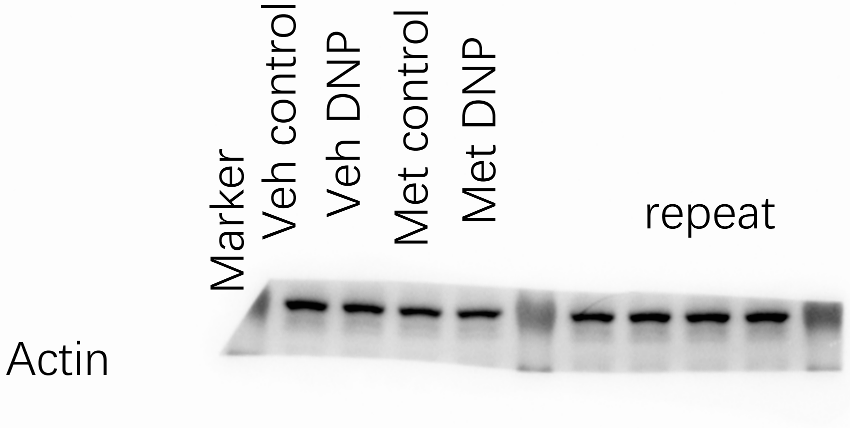

Fig.8f

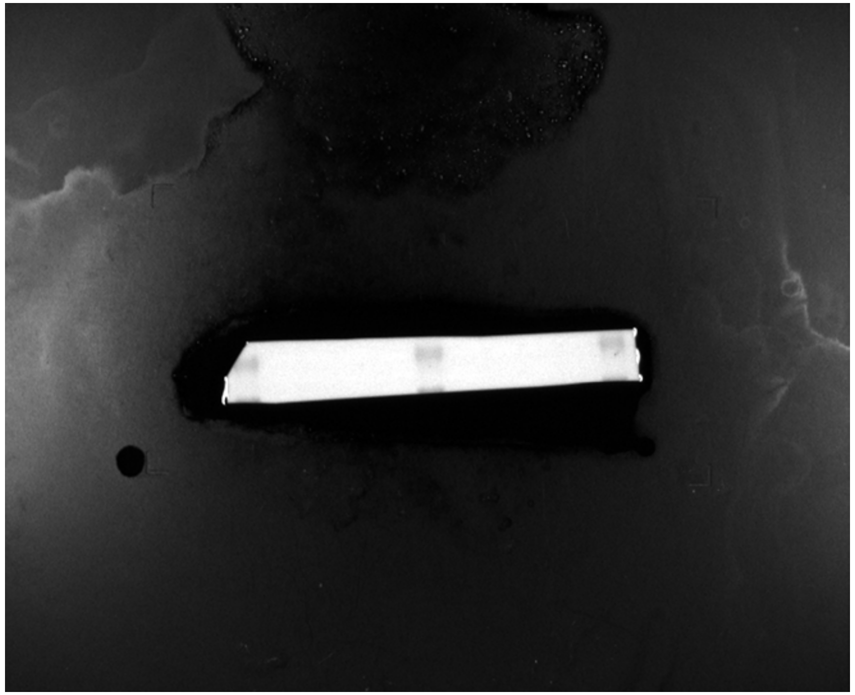

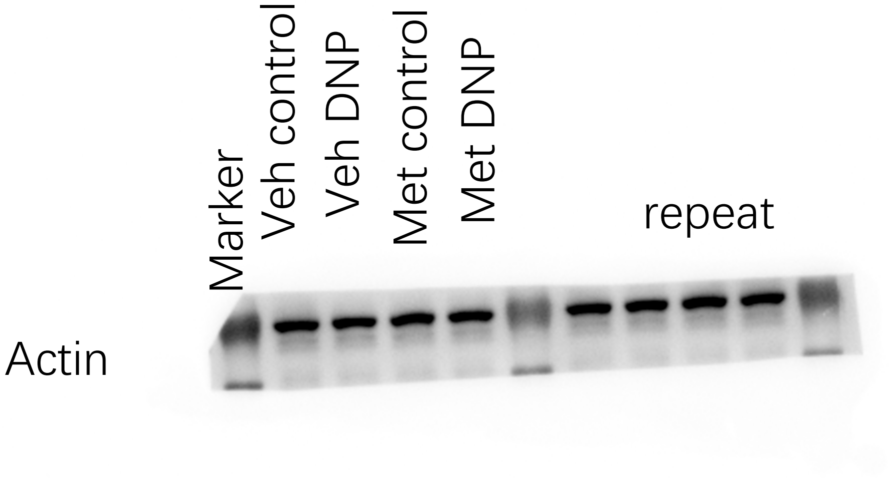

Fig.8h

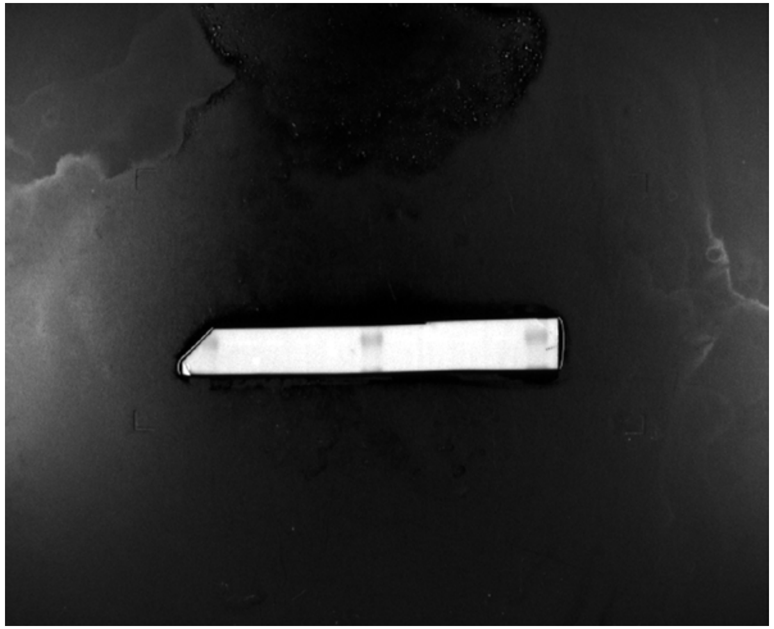

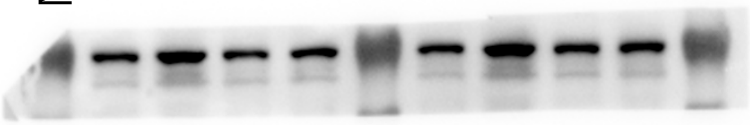

Fig.8h

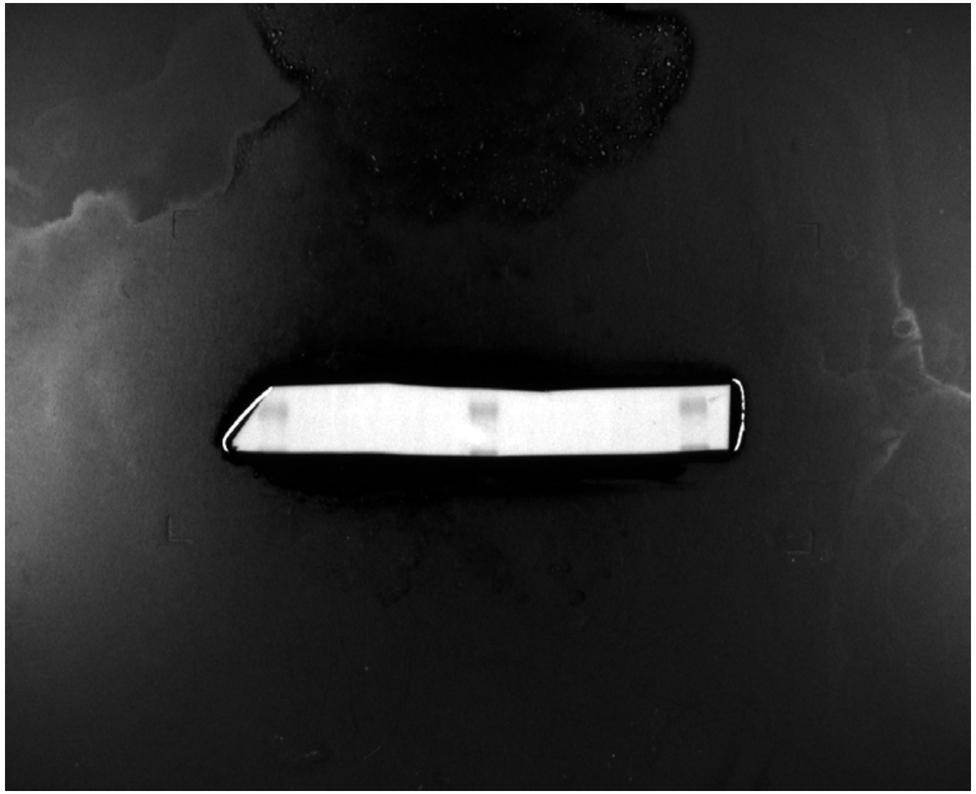

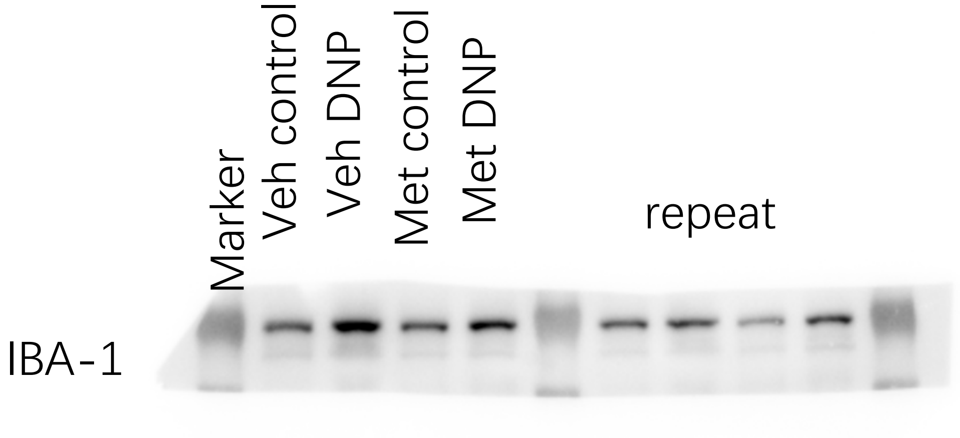

Fig.8h

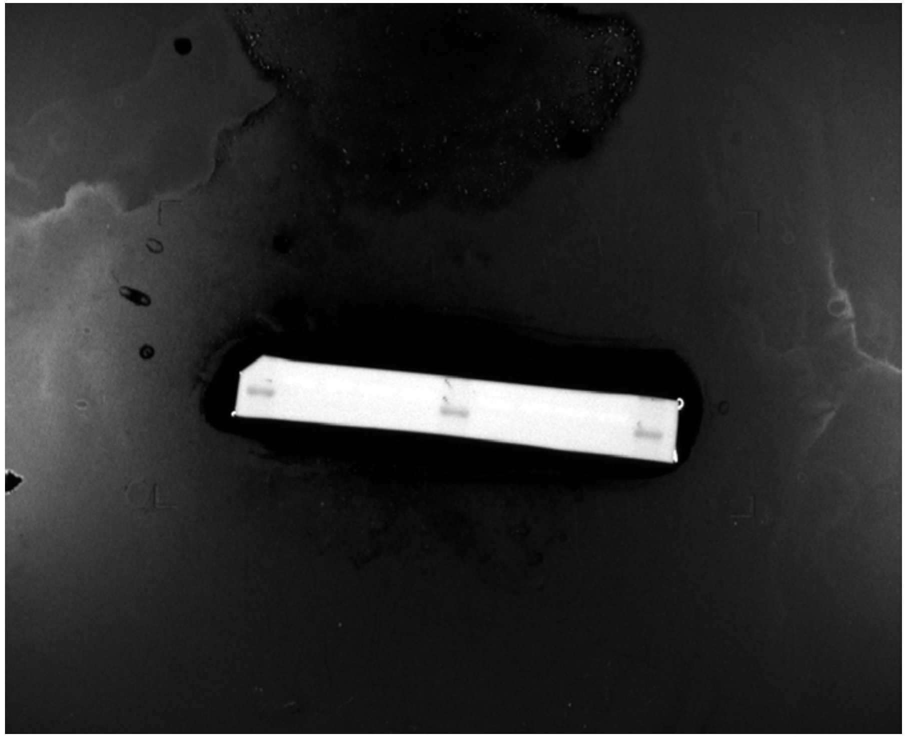

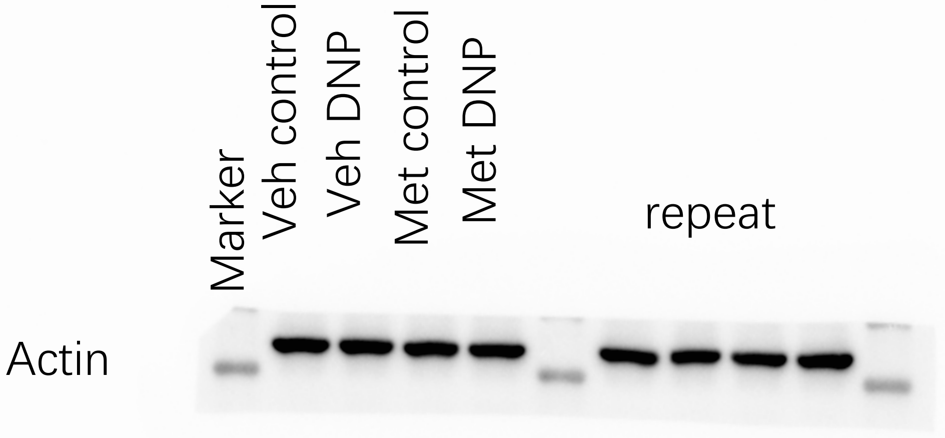

Fig.8h

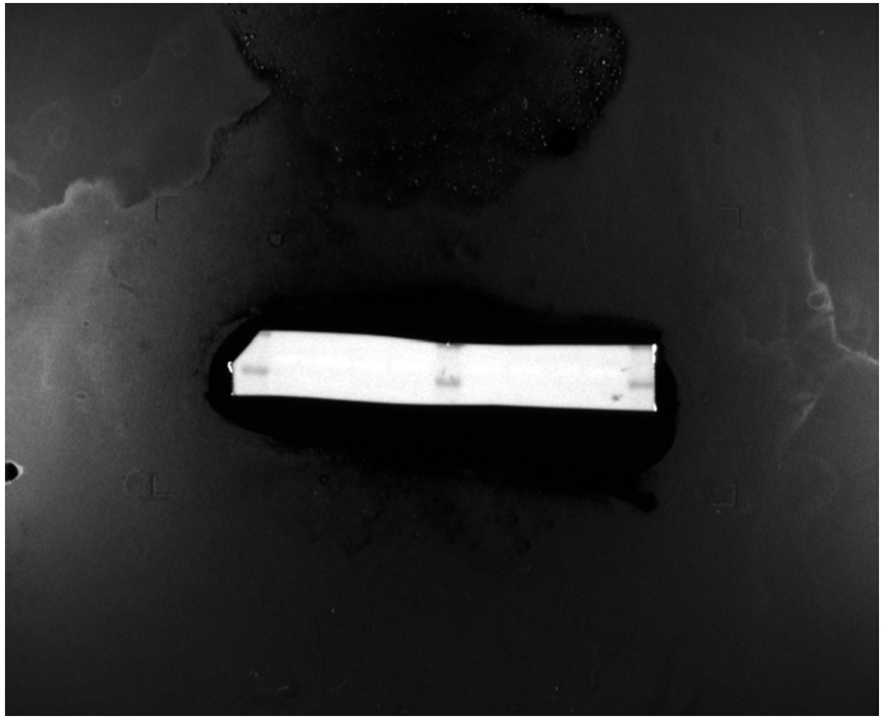

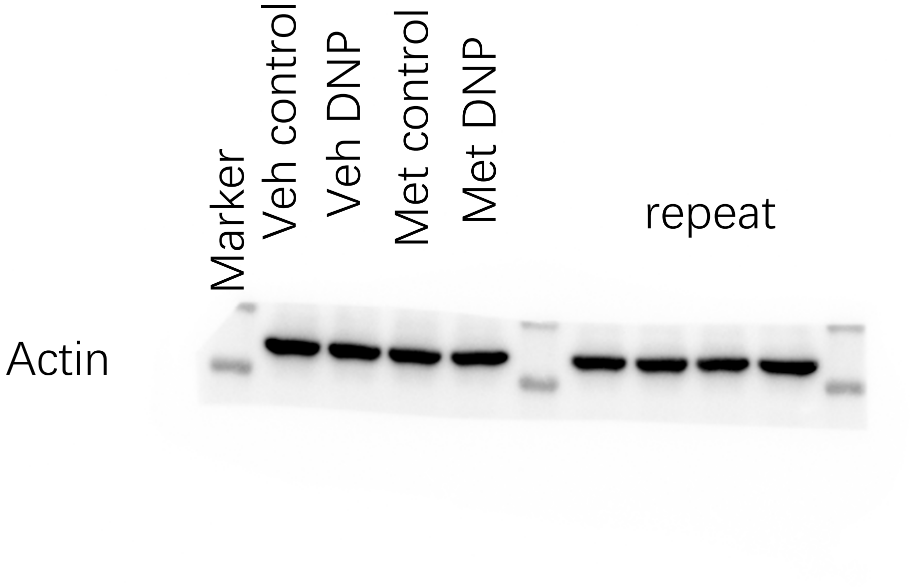

Supplement: Supplementary file 3 — Data S1. [file CNS-30-e14913-s001.pdf]
